# AAV9/*MFSD8* gene therapy is effective in preclinical models of neuronal ceroid lipofuscinosis type 7 disease

**DOI:** 10.1172/JCI146286

**Published:** 2022-03-01

**Authors:** Xin Chen, Thomas Dong, Yuhui Hu, Frances C. Shaffo, Nandkishore R. Belur, Joseph R. Mazzulli, Steven J. Gray

**Affiliations:** 1 Department of Pediatrics, University of Texas Southwestern (UTSW) Medical Center, Dallas, Texas, USA.; 2 The Ken and Ruth Davee Department of Neurology, Northwestern University Feinberg School of Medicine, Chicago, Illinois, USA.

**Keywords:** Neuroscience, Gene therapy, Genetic diseases, Neurodegeneration

## Abstract

Neuronal ceroid lipofuscinosis type 7 (CLN7) disease is a lysosomal storage disease caused by mutations in the facilitator superfamily domain containing 8 (*MFSD8*) gene, which encodes a membrane-bound lysosomal protein, MFSD8. To test the effectiveness and safety of adeno-associated viral (AAV) gene therapy, an in vitro study demonstrated that AAV2/*MFSD8* dose dependently rescued lysosomal function in fibroblasts from a CLN7 patient. An in vivo efficacy study using intrathecal administration of AAV9/*MFSD8* to *Mfsd8^–^^/–^* mice at P7–P10 or P120 with high or low dose led to clear age- and dose-dependent effects. A high dose of AAV9/*MFSD8* at P7–P10 resulted in widespread *MFSD8* mRNA expression, tendency of amelioration of subunit c of mitochondrial ATP synthase accumulation and glial fibrillary acidic protein immunoreactivity, normalization of impaired behaviors, doubled median life span, and extended normal body weight gain. In vivo safety studies in rodents concluded that intrathecal administration of AAV9/*MFSD8* was safe and well tolerated. In summary, these results demonstrated that the AAV9/*MFSD8* vector is both effective and safe in preclinical models.

## Graphical abstract

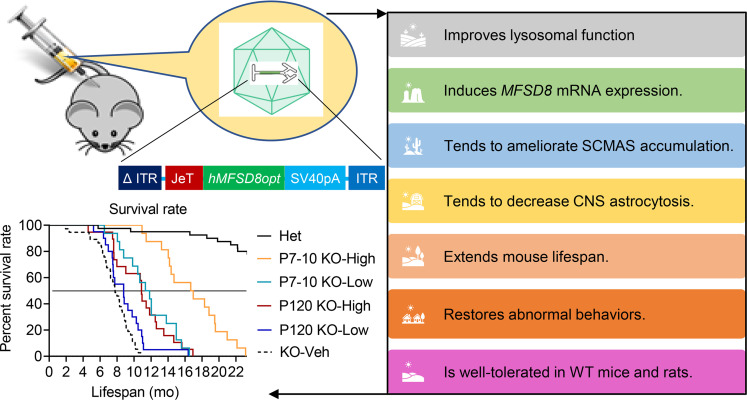

## Introduction

The variant late infantile neuronal ceroid lipofuscinosis type 7 (vLINCL7 or CLN7) disease is a lysosomal storage disease (LSD) caused by a mutation in the gene named major facilitator superfamily domain containing 8 (*MFSD8*). The *MFSD8* gene encodes a 518–amino acid polytopic lysosomal transmembrane protein with 12 membrane-spanning domains (1). Since the initial identification of a mutation in the gene in 2007, a total of 38 different *MFSD8* mutations and 2 sequence variations have been reported in populations throughout the world (2). The types of mutations include missense, splice site, nonsense, frame shift, and sequence deletion or insertion. The autosomal recessive condition in children is inherited from healthy, carrier parents, each contributing a defective allele.

Although the genetic mutations in the *MFSD8* gene resulting in CLN7 disease are well documented and the MFSD8 protein is a member of the major facilitator superfamily (MFS), the function, nature of metabolite(s) transported by MFSD8 protein (3), and therefore disease mechanisms of this progressive neurodegenerative disease are unknown. Histopathology indicates there is progressive loss in neuronal cells in the cerebral cortex layer V, complete loss of the granule cell layer in the cerebellum, age-dependent progressive losses in cerebellar Purkinje cells, and degeneration of photoreceptors in the retina (4). Loss of vision from progressive degeneration of the retina and neuroinflammation in the cerebellar and cerebral cortical regions of human patients are key features of the disease. Defective lysosomal function and dysregulation of autophagy have been suggested as potential contributors to the neurodegenerative mechanism of CLN7 disease (5, 6).

Pathobiology of CLN7 disease is not completely understood. Human *MFSD8* mRNA is ubiquitously expressed in the CNS, heart, placenta, liver, skeletal muscle, and pancreas (1). *Mfsd8* mRNA is expressed throughout the rat brain, with increased levels in the granular layer of the cerebellum and pyramidal layers of the hippocampus (4). The protein is localized to lysosomal membranes (4, 5, 7, 8). MFS proteins are solute transporters and a conserved family function for the MFSD8 protein, and its localization suggests its conserved putative function as a transporter in the lysosomal membrane. Dysfunction of the MFSD8 protein results in the accumulation of lysosomal storage material or autofluorescent ceroid lipopigments in neuronal and peripheral tissues, an important feature of CLN7 disease (4, 5, 9). CLN7 patients with mutations in the *MFSD8* gene were shown to exhibit massive accumulation of subunit c of mitochondrial ATP synthase (SCMAS) in the brain and peripheral organs (10). The ultrastructure of the neuronal storage material in CLN7 patients consists of rectilinear complexes and fingerprint profiles (1, 11, 12). There is elevated expression of lysosomal proteins, including *CTSD*, *CTSB*, and *CTSZ*, in the CLN7 storage phenotype due to enhanced transcription (6). The buildup of storage material in CLN7 disease is thought to lead to the destabilization and increased permeability of the lysosomal membrane, potentially resulting in apoptosis and neurodegeneration (13, 14). A common finding in LSD is a generalized neuroinflammatory response to neuronal storage, as seen by astrogliosis marker glial fibrillary acidic protein (GFAP) staining (5).

The incidence and prevalence of CLN7 disease are unknown, although more than 70 cases have been identified in people around the world (https://medlineplus.gov/genetics/condition/cln7-disease/#frequency). The clinical presentation of CLN7 disease can vary from a mild, late-onset presentation with nonsyndromic visual deficits to a severe, early onset version that manifests as neurological signs with progressive deterioration in intellectual and motor capabilities, seizures, muscle spasms, and visual deficits, culminating in premature death (1, 11, 12, 15–24). Although a recent paper reported some amelioration of CLN7 disease phenotypes by tamoxifen in cellular and murine models (25), there is no approved treatment for patients suffering from CLN7 disease. Management of the condition is limited to symptomatic intervention to treat seizures, dystonia, anxiety, sleep disorders, and spasms (26). Surgery may be required in patients with difficulty swallowing (19). Since all aspects of CLN7 disease stem from the loss of *MFSD8* gene function, *MFSD8* gene–replacement therapy represents a reasonable and promising approach to providing a meaningful and long-term therapeutic benefit for this patient population. Nevertheless, there have been, to our knowledge, no publications describing preclinical gene therapy (GT) studies for CLN7 disease.

Over the last two decades, there have been numerous viral vector–based GT approaches tested for other disorders. Collective evidence has shown that GT can be clinically therapeutic and well tolerated, resulting in therapies for rare inherited diseases and, in some cases, resolving the majority of symptoms (27). Recombinant adeno-associated viral vector type 9 (AAV9) has particularly been shown to be a safe and efficacious neurotropic vector for delivering transgenes to the CNS (28). These recombinant AAV vectors are nonpathogenic, nonreplicating, and transduce both dividing and nondividing cells. Importantly, they are incapable of coding viral proteins and are primarily nonintegrating, making them an ideal vector for gene delivery to the CNS (29). AAV9 mediates broad gene transfer across the entire CNS in a way that translates from mice to larger animal models (28–38). Furthermore, AAV9 can be purified in large quantities at high concentrations for potential use in delivering a functional copy of a gene to cells with aberrant, disease-causing mutations directly to the CNS using intrathecal (i.t.) administration (28–38). AAV9 is also the vector utilized in US FDA–approved GT by an i.v. route, Zolgensma, for infants with spinal muscular atrophy (SMA). The AAV9 vector is also being used for ongoing GT clinical trials, including an AAV9 vector for giant axonal neuropathy (GAN) that was developed by our group and is being used in the first human i.t.-administered AAV trial at the US NIH Clinical Center (30).

In this study, we utilized an approach similar to that of GAN GT to evaluate the efficacy and safety of *MFSD8* gene transfer in vitro in fibroblasts from a CLN7 patient as well as in KO mouse models to investigate whether this would show a potential benefit to pediatric patients suffering from CLN7 disease. To inform the relative risks associated with this approach, we conducted a dose-ranging, 1-year non–good laboratory practice (non-GLP) toxicology study in WT C57BL/6J mice by i.t. administration and performed a comprehensive histopathological assessment at termination. We further conducted a dose-ranging, 3-month GLP toxicology study in WT Sprague Dawley rats by i.t. administration. Here, we present all collected preclinical data in rodents to support clinical evaluation of i.t.-administered AAV9/*MFSD8* as a potential GT for CLN7 patients. This AAV9-based GT strategy can be broadly applied to correct other loss-of-function mutations that lead to CNS disorders.

## Results

### AAV2/MFSD8 vector rescues lysosomal function in primary fibroblasts from a CLN7 patient.

**Figure 1:**
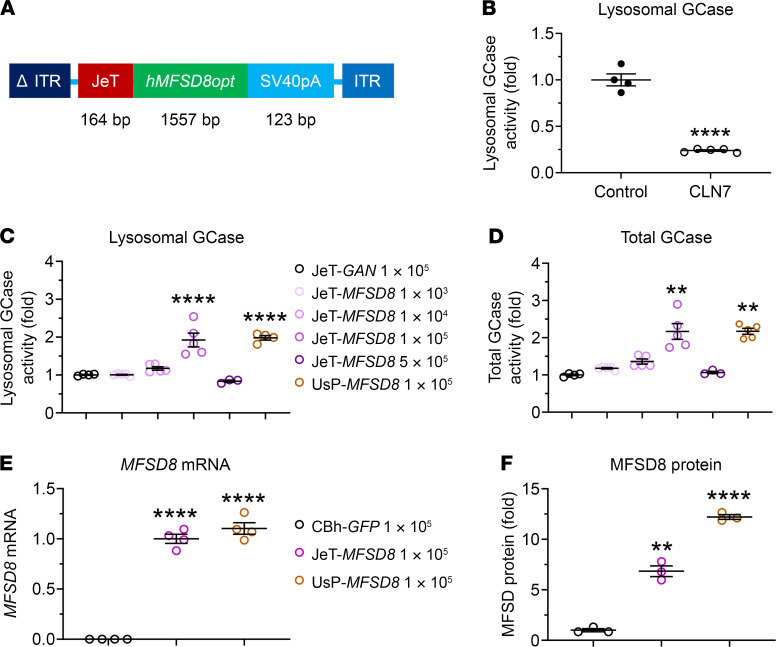
AAV2/*MFSD8* vector construct expressing human *MFSD8* and its rescue of lysosomal function in primary fibroblasts from a CLN7 patient. (**A**) Schematic diagram of AAV2/*MFSD8* construct comprising a mutant AAV2 ITR with the D element deleted (ΔITR), the JeT promoter, the human *MFSD8* codon-optimized coding sequence (*hMFSD8opt*), the synthetic polyadenylation SV40pA signal, and WT AAV2 ITR. (**B**) Lysosomal GCase activity (*n* = 4–5) was measured in fibroblasts from age-matched healthy control and a CLN7 patient. GCase activity was normalized to the cell volume. (**C** and **D**) Lysosomal and total GCase activity (*n* = 3–5) was measured following AAV2-mediated transduction of JeT-*GAN* (negative control), JeT-*MFSD8* (therapeutic transgene at increasing doses), or UsP-*MFSD8* (therapeutic transgene with stronger promotor). The fold differences in lysosomal (**C**) and total (**D**) GCase activity were normalized to the cell volume and to cohorts transfected with JeT-*GAN*. (**E** and **F**) *MFSD8* mRNA and MFSD8 protein (*n* = 3–4) were assayed following AAV2-mediated transduction of CBh-*GFP* (negative control), JeT-*MFSD8*, or UsP-*MFSD8*. A ROUT test was used first to remove any outlier. All data in **B**–**F** are presented as mean ± SEM, with the scatter plot representing measurements from individual culture wells. Data sets that passed tests for normality or homogeneity of variance were analyzed using unpaired *t* test or 1-way ANOVA with α set at 0.05 and Dunnett’s correction for relevant pairwise comparisons. Data sets that did not pass tests for normality or homogeneity of variance were analyzed using the Kruskal-Wallis test with α set at 0.05 and Dunn’s correction for relevant pairwise comparisons. **P < 0.01; ****P < 0.0001, compared with control.

To determine whether AAV-mediated expression of WT *MFSD8* could rescue the function of the lysosomal system in fibroblasts from a CLN7 patient, we created a self-complementary (sc) AAV2/*MFSD8* vector, which is packaged with an expression cassette comprising a mutant AAV2 inverted terminal repeat (ITR) with the D element deleted (ΔITR), the low-expressing JeT promoter (30, 39), the human *MFSD8* codon-optimized coding sequence (*hMFSD8opt*), simian virus 40 polyadenylation (SV40pA) signal, and WT AAV2 ITR (Figure 1A).

Peripheral tissue biopsies taken from human CLN7 patients show accumulation of storage material typical of the disease in the lysosomal compartments, indicating a compromised function (19, 21). There is also elevated expression of lysosomal cathepsins such as *CTSB* in the CLN7 storage phenotype (6). Since the precise function of CLN7 in the lysosome is not known, a functional lysosomal assay (40, 41) that measures lysosomal β-glucocerebrosidase (GCase) was used as a surrogate to measure lysosomal function in patient fibroblast cell cultures. Consistent with our previous report (42), there was a significant reduction in lysosomal GCase activity compared with that of a healthy age-matched individual (Figure 1B), suggesting that CLN7 deficiency compromises general lysosomal function.

As an initial proof-of-concept for human *MFSD8* GT, AAV2/*MFSD8* efficacy at improving lysosomal function in cultured fibroblasts from a CLN7 patient was tested. These assays used an AAV2 vector to deliver the *MFSD8* expression cassette to assess the function of the *hMFSD8opt* transgene expression, as these cells are not readily transduced by AAV9. An AAV2 vector carrying the gigaxonin (*GAN*) transgene driven by the JeT promoter was used as a negative control. In addition to the JeT promoter, a stronger UsP promoter was used to test for a potential additional benefit from higher *MFSD8* transgene expression. Note that the JeT and UsP promoters are identical, except UsP contains an intron that boosts expression. The AAV2/JeT-*MFSD8* titers tested consisted of 1 × 10^3^, 1 × 10^4^, 1 × 10^5^, and 5 × 10^5^ vector genomes (vg)/cell. The AAV2/JeT-*GAN* and the AAV2/UsP-*MFSD8* titers used consisted of 1 × 10^5^ vg/cell.

The enzymatic activity in the fibroblasts transduced with AAV2/JeT-*GAN* was considered the baseline to which activity in test cohorts was compared. There was a dose-dependent increase in the lysosomal function with AAV2/JeT-*MFSD8* titers of 1 × 10^4^ and 1 × 10^5^ vg/cell (Figure 1, C and D). There was about a 2-fold increase in lysosomal and total GCase activity at the 1 × 10^5^ vg/cell multiplicity of infection (MOI). At the highest MOI of 5 × 10^5^ vg/cell, there was no significant improvement in lysosomal GCase activity (Figure 1, C and D). Although toxicity from increased *hMFSD8opt* expression or high AAV doses is a possibility, a general cell-staining assay that measures total cell volume did not demonstrate any significant changes compared with other conditions tested (Supplemental Figure 1, A and B; supplemental material available online with this article; https://doi.org/10.1172/JCI146286DS1). The fold changes in enzymatic activity with the JeT promoter–driven *MFSD8* at the 1×10^5^ vg/cell MOI and the stronger UsP promoter at the 1×10^5^ vg/cell MOI were similar (Figure 1, C and D), suggesting that there is no additional benefit to rescuing lysosomal function by using the stronger promoter at this dose. Similar patterns were seen in total and lysosomal *CTSB* activity (Supplemental Figure 1, E and F).

Further evaluations were performed at a fixed titer of 1 × 10^5^ vg/cell to compare the JeT promoter and UsP promoter in terms of their relative abilities to drive expression of *MFSD8* mRNA and protein as well as to confirm the lysosome rescue results. While both promoters drove similar levels of *MFSD8* mRNA expression, the UsP promoter drove more MFSD8 protein expression compared with the JeT promoter, suggesting that there is a saturating level of transcript/protein above which additional rescue is not achieved (Figure 1, E and F, and Supplemental Figure 1G). In terms of rescuing lysosomal function, the data from 2 additional independent experiments showed increased GCase enzymatic activity with AAV2/JeT-*MFSD8* treatment relative to AAV2/JeT-*GAN* (Supplemental Figure 1, C and D). However, as originally assessed, the 1 × 10^5^ vg/cell dose with a stronger UsP promoter did not result in a significant increase in total and lysosomal GCase activity above that seen with the JeT promoter at the same titer (Supplemental Figure 1, C and D). Taken together, the data from Figure 1 and Supplemental Figure 1 indicate JeT-driven *MFSD8* expression at the vector dose of 1 × 10^5^ vg/cell rescues the lysosomal function in fibroblasts from a CLN7 patient, and there is no observed additional benefit to overexpressing *MFSD8* with a stronger promoter.

### AAV9/MFSD8 GT in KO mice rescues GCase activity, induces MFSD8 mRNA expression, and confers trends of decreased SCMAS accumulation and GFAP immunoreactivity.

**Figure 2:**
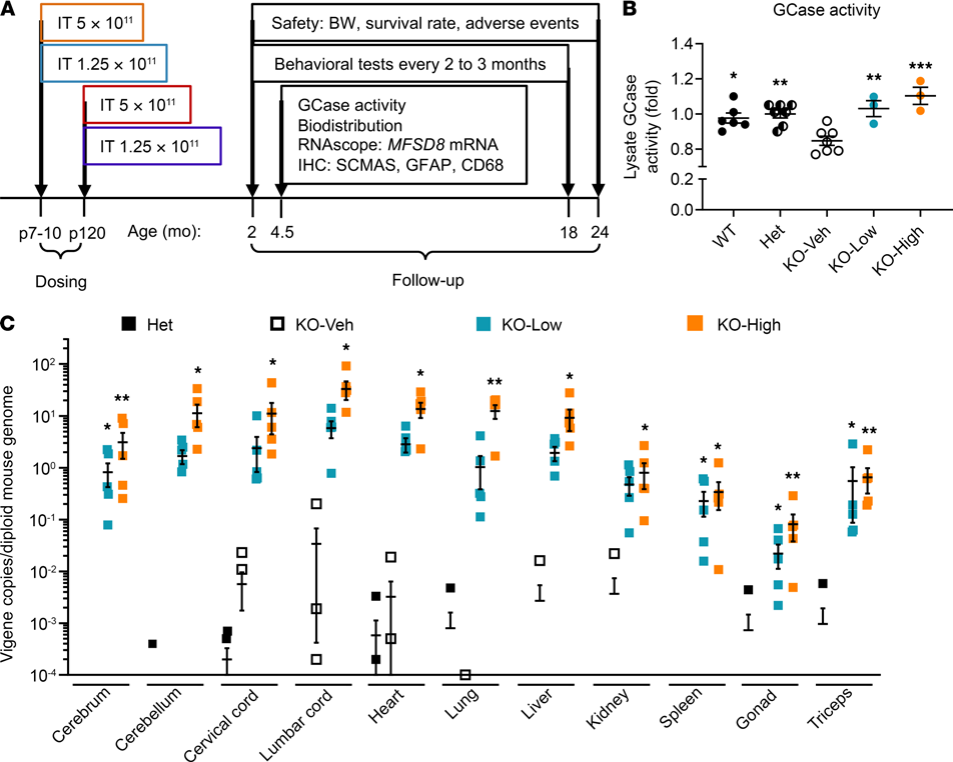
Experimental design for in vivo efficacy study, GCase activity in mouse brain lysate, and vector biodistribution in central and periphery organs. (**A**) High (5 × 10^11^ vg/mouse) or low (1.25 × 10^11^ vg/mouse) dose of AAV9/*MFSD8* vector was administered i.t. to equal numbers of male and female mice at P7–P10 (presymptomatic) or P120 (early symptomatic). Study readouts at each time point at specified age are listed from left to right. (**B**) GCase activity was measured in brain lysates of mice treated at P7–P10 and harvested at 4.5 months old (*n* = 3–7). (**C**) Vector biodistribution was measured in central and periphery organs from mice treated at P7–P10 and harvested at 4.5 months old (*n* = 3–7). A ROUT test was used first to remove any outlier. Data in **B** were normalized to Het mice. All data in **B** and **C** are presented as mean ± SEM, with the scatter plot representing measurements from individual mice. Data sets in **B** and **C** that passed tests for normality or homogeneity of variance were analyzed using 1-way ANOVA with α set at 0.05 and Dunnett’s correction for relevant pairwise comparisons. Data sets that did not pass tests for normality or homogeneity of variance were analyzed using the Kruskal-Wallis test with α set at 0.05 and Dunn’s correction for relevant pairwise comparisons. *P < 0.05; **P < 0.01; ***P < 0.001, compared with KO-Veh.

**Figure 3:**
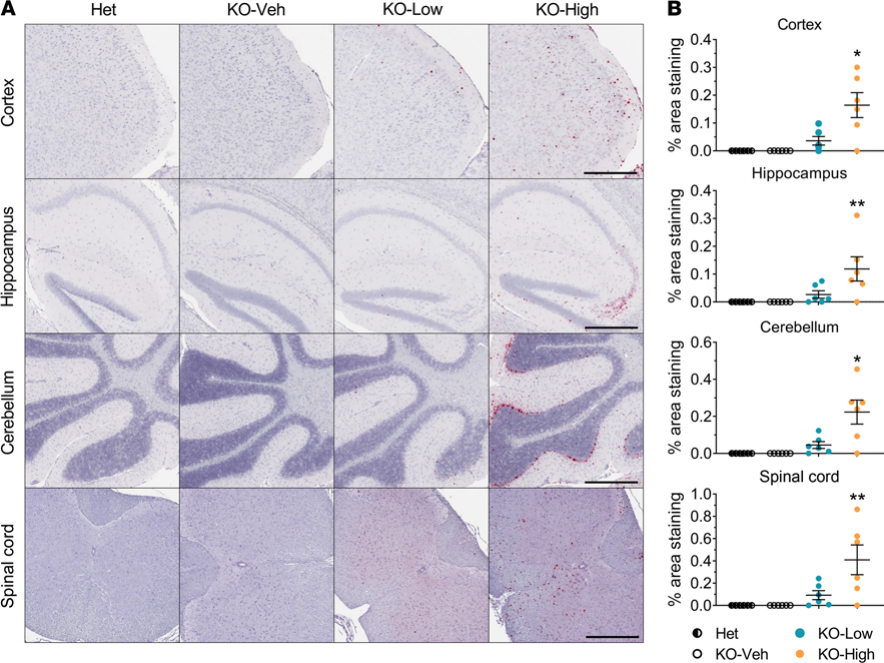
AAV9/*MFSD8* GT dose dependently induces *hMFSD8opt* mRNA expression in the CNS of KO mice. High (5 × 10^11^ vg/mouse) or low (1.25 × 10^11^ vg/mouse) dose of AAV9/*MFSD8* vector was administered i.t. to equal numbers of male and female mice at P7–P10. At 4.5 months old, mouse brain and spinal cord were harvested for RNAscope staining to detect *hMFSD8opt* mRNA (**A**). Histology images with 1 section/animal were digitized with a ScanScope slide scanner and analyzed using custom analysis settings in HALO Image Analysis Platform. Results are presented as percentage of area staining positive for *hMFSD8opt* mRNA by tissue region (**B**). A ROUT test was used first to remove any outlier. Each data point represents a measurement from an individual animal (*n* = 5–6), with lines representing the mean measurement ± SEM. Data sets that passed tests for normality or homogeneity of variance were analyzed using 1-way ANOVA with α set at 0.05 and Dunnett’s correction for relevant pairwise comparisons. Data sets that did not pass tests for normality or homogeneity of variance were analyzed using the Kruskal-Wallis test with α set at 0.05 and Dunn’s correction for relevant pairwise comparisons. *P < 0.05; **P < 0.01, compared with KO-Veh. Scale bars: 500 μm.

**Figure 4:**
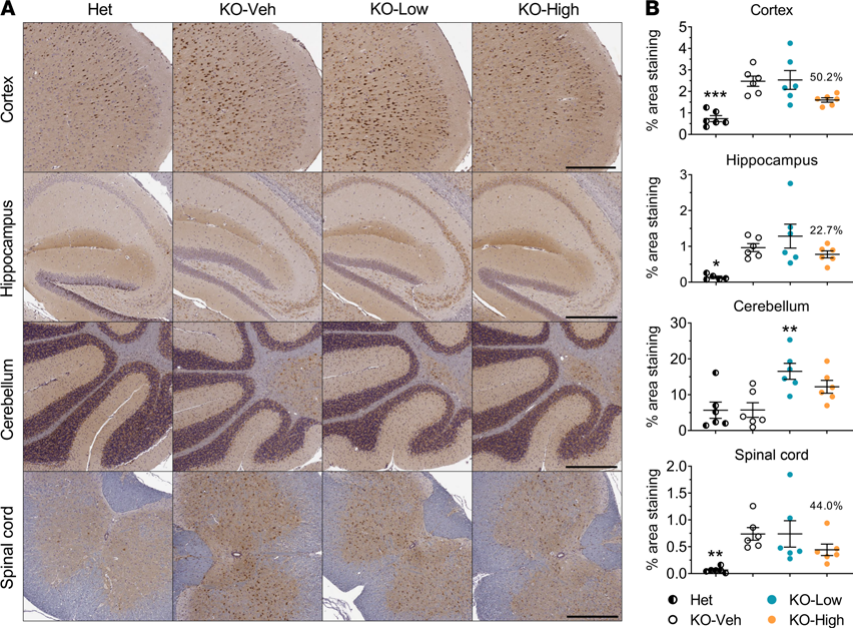
High dose of AAV9/*MFSD8* GT tends to ameliorate SCMAS accumulation in the brain and spinal cord of KO mice. High (5 × 10^11^ vg/mouse) or low (1.25 × 10^11^ vg/mouse) dose of AAV9/*MFSD8* vector was administered intrathecally to equal numbers of male and female mice at P7–P10. At 4.5 months of age, mouse brain and spinal cord were harvested for IHC staining to detect SCMAS (**A**). Histology images with 1 section/animal were digitized with a ScanScope slide scanner and analyzed using custom analysis settings in the HALO Image Analysis Platform. Results are presented as the percentage of area staining positive for SCMAS by tissue region (**B**). A ROUT test was used first to remove any outlier. Each data point represents a measurement from an individual animal (*n* = 5–6), with lines representing the mean measurement ± SEM. Percentages above the KO-High data points indicate the reduction in the mean compared with KO-Veh. Data sets that passed tests for normality or homogeneity of variance were analyzed using 1-way ANOVA with α set at 0.05 and Dunnett’s correction for relevant pairwise comparisons. Data sets that did not pass tests for normality or homogeneity of variance were analyzed using the Kruskal-Wallis test with α set at 0.05 and Dunn’s correction for relevant pairwise comparisons. *P < 0.05; **P < 0.01; ***P < 0.001, compared with KO-Veh. Scale bars: 300 μm (cortex); 500 μm (hippocampus, cerebellum, spinal cord).

**Figure 5:**
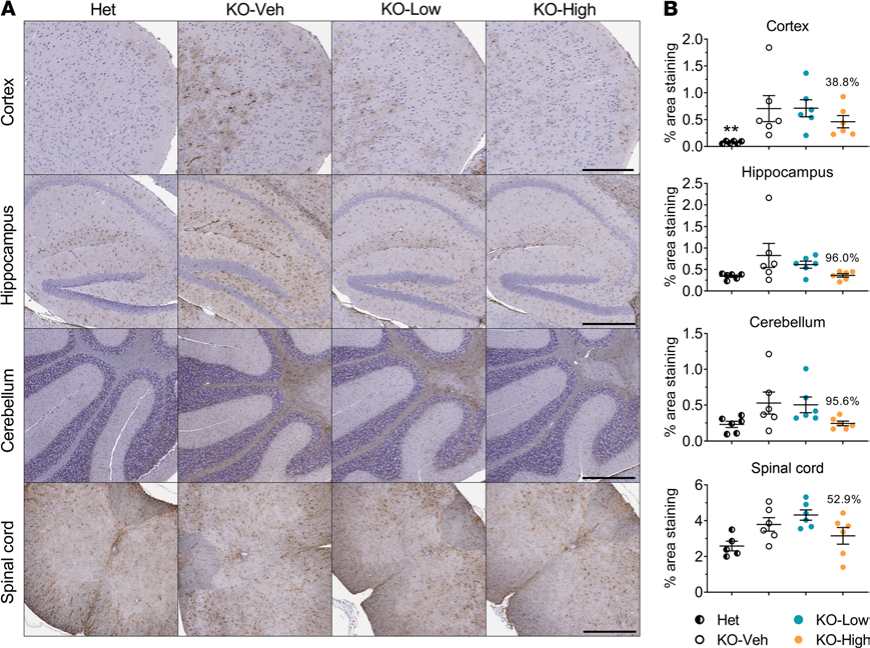
High dose of AAV9/*MFSD8* GT tends to decrease GFAP immunoreactivity in the brain and spinal cord of KO mice. High (5 × 10^11^ vg/mouse) or low (1.25 × 10^11^ vg/mouse) dose of AAV9/*MFSD8* vector was administered i.t. to equal numbers of male and female mice at P7–P10. At 4.5 months of age, mouse brain and spinal cord were harvested for IHC staining to detect GFAP (**A**). Histology images with 1 section/animal were digitized with a ScanScope slide scanner and analyzed using custom analysis settings in the HALO Image Analysis Platform. Results are presented as percentage of area staining positive for GFAP by tissue region (**B**). A ROUT test was used first to remove any outlier. Each data point represents measurement from an individual animal (*n* = 5–6), with lines representing the mean measurement ± SEM. Percentages above the KO-High data points indicate the reduction in the mean compared with KO-Veh. Data sets that passed tests for normality or homogeneity of variance were analyzed using 1-way ANOVA with α set at 0.05 and Dunnett’s correction for relevant pairwise comparisons. Data sets that did not pass tests for normality or homogeneity of variance were analyzed using the Kruskal-Wallis test with α set at 0.05 and Dunn’s correction for relevant pairwise comparisons. **P < 0.01, compared with KO-Veh. Scale bars: 300 μm (cortex); 500 μm (hippocampus, cerebellum, spinal cord).

To determine whether the AAV9/*MFSD8* vector rescues the phenotypes in *Mfsd8^–/–^* (KO) mice, groups with equal numbers of male and female KO mice were injected i.t. at P7–P10 (presymptomatic cohorts) or P120 (early symptomatic cohorts) with a single high (5 × 10^11^ vg/mouse) or low (1.25 × 10^11^ vg/mouse) dose of AAV9/*MFSD8* vector (Figure 2A). At 4.5 months of age, 3 males and 3 females from each dose or control group treated at P7–P10 were taken down to evaluate GCase activity (Figure 2B), vector biodistribution (Figure 2C), *MFSD8* mRNA expression (Figure 3), and early histological signs (Figure 4, Figure 5, and Supplemental Figures 2 and 3) of treatment efficacy. Compared with WT or Het control mice, there was significantly reduced GCase activity in brain lysates of KO mice receiving vehicle treatment (KO-Veh), which was fully rescued by the low (KO-Low) or high (KO-High) dose of AAV9/*MFSD8* vector (Figure 2B). The i.t. delivery of AAV9/*MFSD8* vector resulted in a dose-dependent increase of *MFSD8* vector DNA across the CNS (brain and spinal cord) and peripheral organs (heart, lung, liver, kidney, spleen, gonad, and triceps). The *MFSD8* vector DNA was concentrated closest to the injection site in the spinal cord and detected at lower levels in multiple brain regions. In the peripheral organs, similar high amounts of *MFSD8* DNA persisted in heart, lung, and liver and to a lesser extent in kidney, spleen, gonad, and triceps (Figure 2C). Animals receiving either the low or high dose of AAV9/*MFSD8* vector had detectable levels of transgene *hMFSD8*opt mRNA in all tissues and brain regions assessed (Figure 3). The KO-High group had significantly higher mRNA levels than the KO-Veh group.

In CLN7 disease, there is progressive neurodegeneration accompanied by neuroinflammation evidenced by astrocytosis and microgliosis (43). Histopathology of CLN7 tissue shows similar changes in the brain tissue of patients (4) and in mouse models (6). IHC with GFAP and ionized calcium binding adaptor molecule 1 (Iba1) antibodies identify pronounced astrogliosis and microgliosis, respectively, in CLN7-deficient mice (6). SCMAS and sphingolipid activator proteins (saposins A and D) are components of the autofluorescent storage material retained in the lysosomes of neuronal tissue in LSDs (5, 6, 24).

IHC with primary antibody against SCMAS was used to assay the accumulation in neuronal tissue isolated from the mice at the age of 4.5 months. The increase in SCMAS can be observed as higher amounts of brown stain in these tissue sections (Figure 4A). Accumulation of SCMAS was evident in KO-Veh animals compared with Het controls in the cortex, hippocampus, and spinal cord, but not in the cerebellum (Figure 4B). The low dose of AAV9/*MFSD8* had minimal effect on SCMAS staining in these same tissue regions. It should be noted that in the cerebellum and hippocampus, variable and sometimes high background staining made the automated image analysis inconsistent at times, which may explain the apparent increase of SCMAS upon low-dose treatment in the cerebellum. Our opinion, given qualitatively and without being blind to treatment or genotype, is that there is not an increase in SCMAS staining in the cerebellum. In contrast, the high dose of AAV9/*MFSD8* showed trends of reduced SCMAS accumulation by up to 50.2% in the brain and spinal cord of KO mice, although the differences compared with that of vehicle-injected KO mice were not statistically significant (Figure 4B and Supplemental Table 1).

GFAP immunoreactivity was significantly increased in the cortex of KO-Veh group mice compared with Het controls at the age of 4.5 months, and the high dose of AAV9/*MFSD8* tended to reduce GFAP immunoreactivity by 38.8% in the cortex of KO mice (Figure 5 and Supplemental Table 1). Similar trends of increased GFAP immunoreactivity were observed in the hippocampus, cerebellum, and spinal cord of the KO-Veh group, which showed consistent trends of reduction upon treatment with AAV9/*MFSD8*, but none of the differences were statistically significant (Figure 5 and Supplemental Table 1).

Cluster of differentiation 68 (CD68) immunoreactivity is an alternative marker to Iba1 for microglia, but this was not significantly increased in any brain region analyzed in any group compared with KO-Veh at the age of 4.5 months (Supplemental Figure 2 and Supplemental Table 1). NeuN^+^, total cell numbers, and the NeuN^+^/total cell ratio were not significantly changed in any brain region analyzed in any group compared with KO-Veh at the age of 4.5 months (Supplemental Figure 3 and Supplemental Table 1). Taken together, these data suggest that the AAV9/*MFSD8* GT is effective at slowing or preventing histological signs of disease progression, but that these effects are dose dependent and require a high dose to be effective.

### AAV9/MFSD8 GT improves survival rate, extends median life span, and maintains normal body weight longer in KO mice.

**Figure 6:**
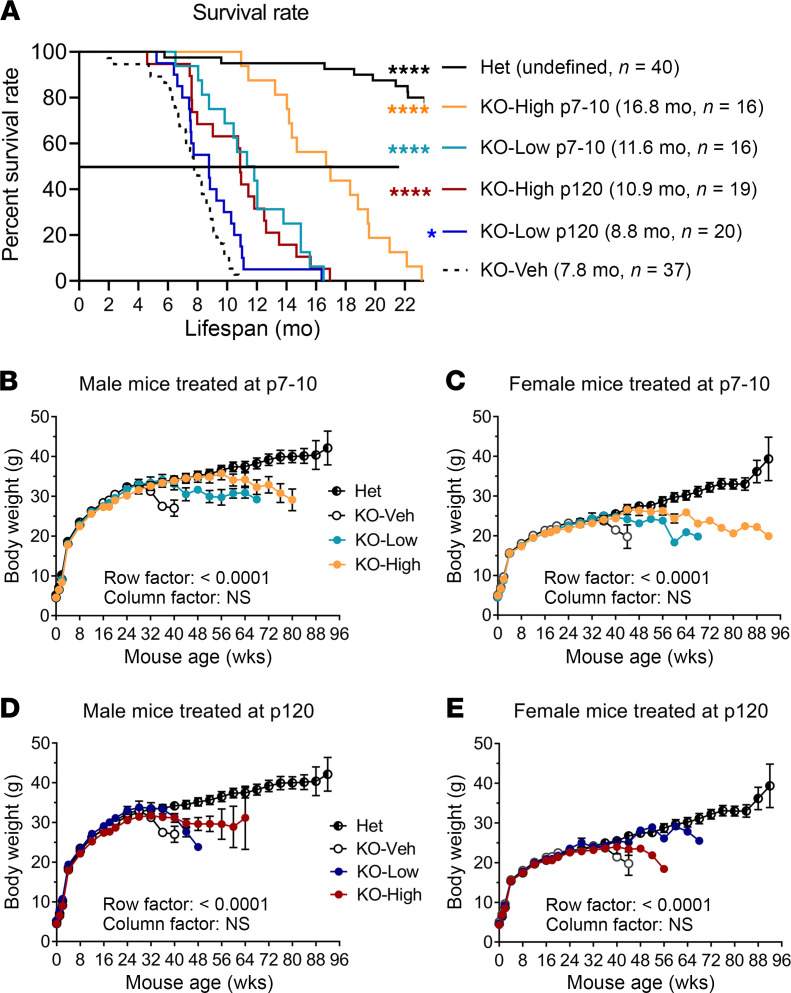
AAV9/*MFSD8* GT improves survival rate, extends median life span, and maintains normal BW longer in KO mice. High (5 × 10^11^ vg/mouse) or low (1.25 × 10^11^ vg/mouse) dose of AAV9/*MFSD8* vector was administered i.t. to equal numbers of male and female mice at P7–P10 or P120. (**A**) Kaplan-Meier survival curve shows the survival over time with median survival and mouse number enrolled in parentheses. Data were compared with log-rank (Mantel-Cox) test. *P < 0.05; ****P < 0.0001 compared with KO-Veh. (**B**–**E**) BW of animal treated at P7–P10 (**B** and **C**) or P120 (**D** and **E**). All data in **B**–**E** are presented as mean ± SEM (*n* = 16–40). Two-way ANOVA with repeated measures was used for significance analyses.

There is increased mortality in KO mice along with associated behavioral deficits (6). We found that KO-Veh animals started experiencing mortality after 2 months of age, and survival drastically decreased between 6 and 11 months of age (Figure 6A). AAV9/*MFSD8* administration had a significant effect on survival in both an age-dependent and dose-dependent manner, with the early treatment and high-dose group showing a larger increase in survival, whereas the late-treatment and low-dose groups showed moderately increased survival (Figure 6A). The i.t. high dose of AAV9/*MFSD8* at P7–P10 resulted in a greater than doubling of median life span (16.8 months versus 7.8 months in KO-Veh mice; Figure 6A). There was no survival benefit when KO mice were treated at 6 months of age with the high i.t. dose (Supplemental Figure 4), further supporting that CLN7 needs to be treated early for treatment to be effective. There was a notable drop in body weight (BW) as KO mice approached median survival age across all dose cohorts (Figure 6, B–E). All KO-Veh mice lost BW rapidly from approximately 6 months of age, whereas AAV9/*MFSD8*-treated KO mice showed an age- and dose-dependent maintenance of normal BW for a longer time. No neurologic symptoms or general malaise related to the treatment was observed. These results suggest that AAV9/*MFSD8* GT is effective and safe in this preclinical disease model.

### AAV9/MFSD8 GT restores impaired behavioral phenotypes in KO mice.

**Figure 7:**
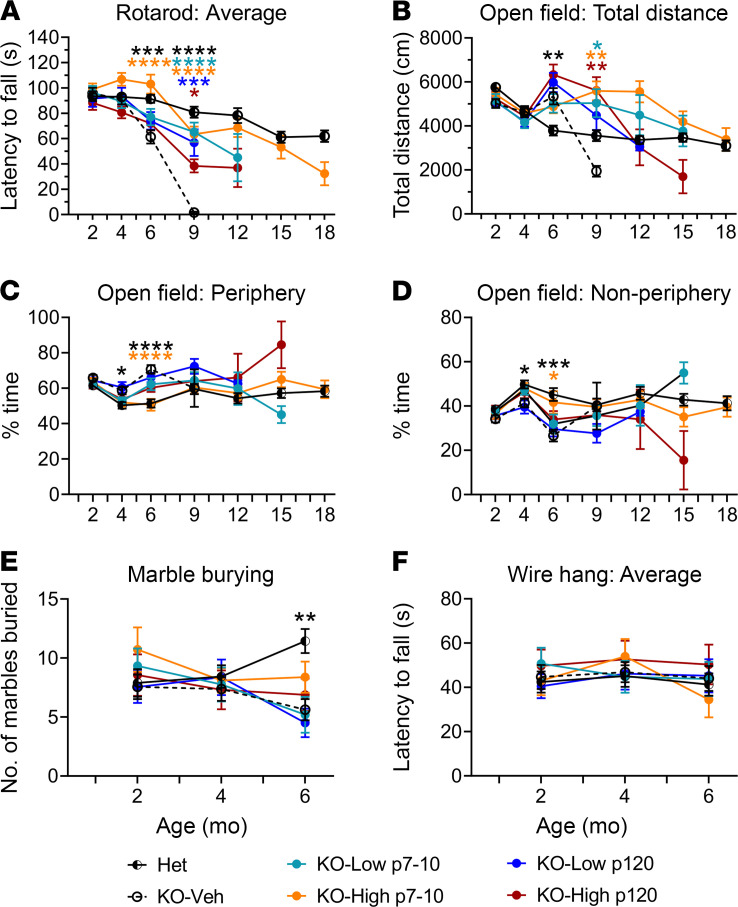
AAV9/*MFSD8* GT restores impaired behavioral phenotypes in KO mice. High (5 × 10^11^ vg/mouse) or low (1.25 × 10^11^ vg/mouse) dose of AAV9/*MFSD8* vector was administered i.t. to equal numbers of male and female mice at P7–P10 or P120. The mice were allowed to complete rotarod (**A**), open-field (**B**–**D**), marble-burying (**E**), and wire-hang (**F**) tests. All data are presented as mean ± SEM (*n* = 16–40). Data sets of each time point that passed tests for normality or homogeneity of variance were analyzed using 1-way ANOVA with α set at 0.05 and Dunnett’s correction for relevant pairwise comparisons. Data sets that did not pass tests for normality or homogeneity of variance were analyzed using the Kruskal-Wallis test with α set at 0.05 and Dunn’s correction for relevant pairwise comparisons. *P < 0.05; **P < 0.01; ***P < 0.001; ****P < 0.0001, compared with KO-Veh.

There are motor deficits, including hind-limb paralysis, tremors, and epilepsies, in the KO mice (6) that correlate with the neurodegenerative manifestation in CLN7 patient populations (23, 24). To determine whether AAV9/*MFSD8* GT ameliorates these deficits, mouse cohorts underwent a battery of behavioral testing that included rotarod, open-field, marble-burying, and wire-hang tests (Figure 7). These tests were selected based on known or suspected deficits in the KO mouse model as well as for their ability to be repeated longitudinally. Performance of mice on the rotarod reflects motor coordination capabilities (44). The cohorts were tested for their ability to walk forward without falling on a horizontal rod rotating on its long axis at an accelerating speed. Latency to fall was recorded over multiple trials per mouse. This assay quantifies loss or improvements in the motor-coordination differences between untreated and treated KO mice. The open-field test in mice is a tool to assess novel environment exploration, anxiety-related behavior, and general locomotor activity (45). Performance of the KO mice on these tests had not been previously reported; thus, this testing will serve to assess both the utility of the open-field test for the CLN7-specific phenotype and for potential therapeutic rescue. Additionally, mice were tested for marble burying, a behavioral assay that utilizes the natural digging behavior of mice. Increased digging/marble burying can be observed in multiple models of psychiatric disease (46). Wire hang was also used to test grip strength.

No or minimal behavioral deficits were observed between Het control animals and KO-Veh or KO-AAV9/*MFSD8* dosed animals at 2 or 4 months of age (Figure 7). At 6 months of age, behavioral deficits were observed in the KO-Veh group compared with Het controls in multiple assays including rotarod, open field, and marble burying, with deficit rescue observable in the P7–P10 high-dose group. KO-Veh animals performed poorly on the rotarod compared with Het controls, with a significantly shortened latency to fall across 8 trials (Figure 7A). This deficit was ameliorated in the P7–P10 high-dose group, which performed significantly better than the KO-Veh group and was not significantly different from the Het control group. In the open field, KO-Veh animals were hyperactive, traveling a greater total distance during the testing period, and spent significantly more time on the periphery of the testing arena compared with Het controls (Figure 7, B–D). Spending more time in the periphery of the testing arena in KO-Veh animals is indicative of anxiety-like behavior and was normalized in the P7–P10 high-dose group. In marble burying, KO-Veh mice buried significantly fewer marbles than Het controls, as did both KO low-dose groups (Figure 7E). This was an unexpected finding, since mouse models of anxiety and psychiatric disorders show an increase in digging/marble burying; however, studies have also shown that hippocampal lesions can reduce these natural behaviors (47). While there was a trend toward normalization of marble-burying behavior in the high-dose treatment groups, it was not statistically significant. There were no significant differences between any groups in wire-hang performance (Figure 7F) at 6 months of age.

Only 6 KO-Veh animals survived to the 9-month testing, and these animals were unable to stay on the rotarod testing apparatus for more than a few seconds (Figure 7A). All treated animals performed better on the rotarod than KO-Veh animals, with early treatment achieving better benefits. In the open field, there were only significant differences in the total distance traveled (Figure 7, B–D). The P7–P10 and P120 high-dose–treated animals remained hyperactive compared with Het controls and KO-Veh animals.

All KO-Veh animals died by 12 months of age, and only 1 P120 low-dose animal survived to the 12-month testing (Figure 6A). Thus, the behavior tests were mainly performed on other groups, including Het controls, P7–P10 low dose, P7–P10 high dose, and P120 high dose (Figure 7, A–D). The majority of treated animals were able to perform on the rotarod, with only the P7–P10 high-dose group having a latency to fall comparable to that of the Het controls (Figure 7A). In the open field, the P7–P10 treated animals remained hyperactive (Figure 7B).

There were only a few treated KO animals alive by 15 months of age and no animals alive by 18 months of age in other treatment groups. Remaining animals were either Het controls or KOs treated with P7–P10 high dose (Figure 6A). The remaining KO animals treated with P7–P10 high dose were still able to perform well on the rotarod and open field, with performance comparable to that of Het controls (Figure 7, A–D).

Taking these data together, there were notable behavioral deficits starting from 6 months of age in the KO mice, and some deficits seen on the rotarod and open field were completely restored in the P7–P10 high-dose group, with a trend toward improvement in some other treatment groups. All these results indicate positive treatment effects of AAV9/*MFSD8* GT in both survival and quality of life.

### AAV9/MFSD8 GT is safe and well tolerated in WT mice in a non-GLP study.

**Figure 8:**
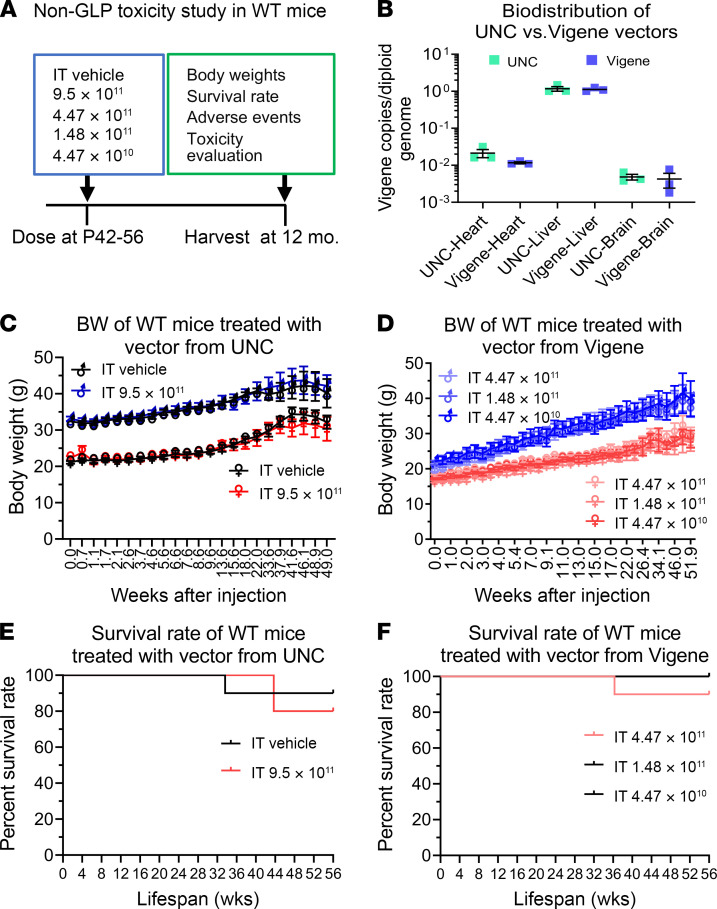
AAV9/*MFSD8* GT does not significantly affect BW or survival rate of WT mice in non-GLP toxicity study. (**A**) Experimental design of the non-GLP toxicity study in WT mice. (**B**) In vivo equivalence of preclinical lots of AAV9/*MFSD8* vectors from UNC and Vigene. WT Mice (*n* = 3) in each group were injected with the vector via tail vein in a 200 μL bolus of 2 × 10^11^ vg/mouse. Mouse heart, liver, and brain were harvested a week later for biodistribution analysis. Data sets that passed tests for normality or homogeneity of variance were analyzed using unpaired *t* test with α set at 0.05. Data sets that did not pass tests for normality or homogeneity of variance were analyzed using Mann-Whitney *U* test with α set at 0.05. No significance was observed. (**C** and **D**) BW of WT mice (*n* = 5/group/sex) treated with vector from UNC (**C**) or Vigene (**D**). Two-way ANOVA with repeated measures was used for statistical analysis, and no interaction significance was observed. All data in **B**–**D** are presented as mean ± SEM. (**E** and **F**) Survival rate of WT mice (*n* = 5/group/sex) treated with vector from UNC (**E**) or Vigene (**F**). Data shown in Kaplan-Meier survival curve were compared with log-rank (Mantel-Cox) test. No significance was observed.

To demonstrate the long-term safety of the AAV9/*MFSD8* vector, WT C57BL/6J mice were injected i.t. with the AAV9/*MFSD8* vector in a non-GLP study (Figure 8A). The mice were randomized to different groups, injected i.t. with 5 μL of vehicle or different doses of AAV9/*MFSD8* vectors from University of North Carolina–Vector Core (UNC) or Vigene Biosciences, and monitored up to 1 year following injection for BW, survival, adverse events, and histopathology evaluation. The UNC- and Vigene-produced vectors were retitered in parallel, then evaluated for equivalency by conducting a limited biodistribution analysis after i.v. injection in mice at the same dose. Biodistribution results demonstrated that they were functionally equivalent (Figure 8B). In this non-GLP study, there was no significant difference in BW between groups in male or female mice at any point of assessment and any dose tested (Figure 8, C and D). There were no obvious signs of morbidity in the adult WT mice dosed with AAV9/*MFSD8* at doses up to 9.50 × 10^11^ vg/mouse over the duration of the study. There were 4 unexpected deaths in this study: 1 animal found dead in the control group injected with vehicle with no obvious reason, 1 found dead in the treated group injected with 4.47 × 10^11^ vg/mouse with no obvious reason, 1 found dead in the treated group injected with 9.50 × 10^11^ vg/mouse that was most likely caused by overgrooming-induced severe back injury, and 1 euthanized for animal welfare in the treated group injected with 9.50 × 10^11^ vg/mouse because of overgrooming-induced severe back and leg injury. Therefore, there were no significant differences in survival rates between groups in male or female mice (Figure 8, E and F). At the end of the experiment, the tissues of the 46 surviving mice were sent out for a blinded histopathology evaluation that concluded that none of the microscopic findings were suggestive of adverse effects related to vector administration in these mice (Supplemental Toxicology Report 1, UTSW). Taken together, all these results demonstrate that doses up to 9.50 × 10^11^ vg/mouse are well tolerated in WT C57BL/6J mice up to 12 months following treatment.

### AAV9/MFSD8 GT is safe and well tolerated in WT rats in a GLP study.

**Figure 9:**
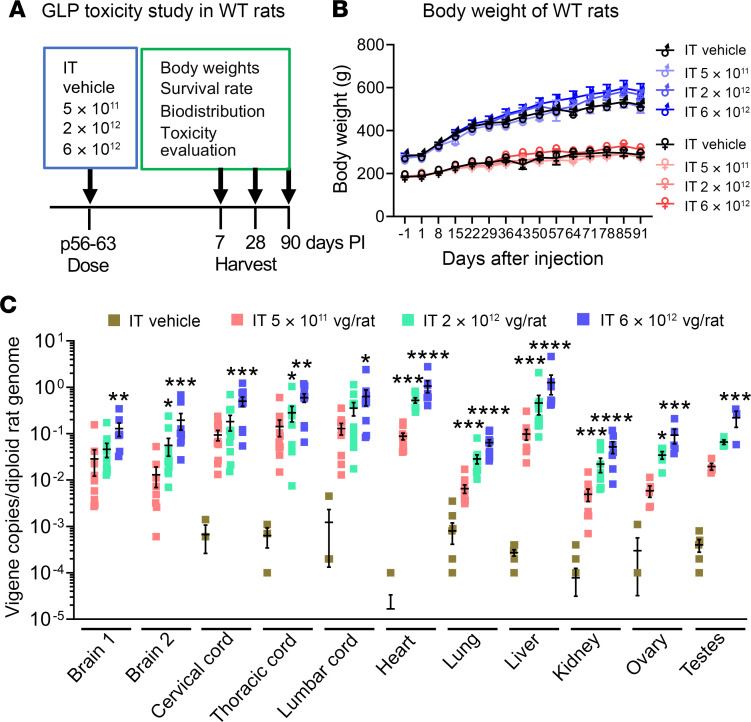
AAV9/*MFSD8* GT dose dependently increases *MFSD8* biodistribution in all rat organs tested, but does not significantly affect BW or cause severe adverse effects in WT rats in a GLP toxicity study. (**A**) Experimental design of the GLP toxicity study in WT rats. (**B**) BW of WT rats treated with vector from Vigene. Two-way ANOVA with repeated measures was used for statistical analysis, and no interaction significance was observed. (**C**) *MFSD8* biodistribution of organs harvested at 28 days following the administration of AAV9/*MFSD8* vector. Data sets that passed tests for normality or homogeneity of variance were analyzed using 1-way ANOVA with α set at 0.05 and Dunnett’s correction for relevant pairwise comparisons. Data sets that did not pass tests for normality or homogeneity of variance were analyzed using the Kruskal-Wallis test with α set at 0.05 and Dunn’s correction for relevant pairwise comparisons. All data in **B** and **C** are presented as mean ± SEM (*n* = 5/group/sex).

To further demonstrate the safety and biodistribution pattern of AAV9/*MFSD8* vector, WT Crl:CD(SD) (CD) rats were injected with AAV9/*MFSD8* vector in a GLP study (Figure 9A). The animal study was performed by Charles River Laboratories Inc. Male and female CD rats were randomized into cohorts, with 5 males and 5 females per cohort, and dosed by a qualified laboratory technician. At the initiation of dosing, the animals assigned to the study were approximately 56 to 63 days old and injected i.t. once in each animal with a dose of 5 × 10^11^, 2 × 10^12^, or 6 × 10^12^ vg/rat. All animals were monitored up to 90 days following the injection for BW, survival, biodistribution, rotarod performance, and toxicology, including blood chemistry and histopathology. Rats were sacrificed on day 7, 28, or 90 after injection, and tissues were collected for biodistribution and toxicity evaluation. In this GLP rat study, there was no significant difference in BW between groups in male or female rats at any point of assessment and any dose tested (Figure 9B). There were no obvious signs of morbidity or mortality in the adult WT rats dosed with AAV9/*MFSD8* at doses up to 6 × 10^12^ vg/rat over the duration of the study. Total genomic DNA was purified from tissue samples collected at necropsy day 28, using a QIAGEN QIAcube HT. Quantitative PCR (qPCR) was used to determine the quantity of the *MFSD8* transgene per diploid rat genome (Figure 9C). Consistent with the results in mice (Figure 2C), i.t. delivery of AAV9/*MFSD8* vector resulted in a dose-dependent increase of *MFSD8* vector DNA across the CNS (brain and spinal cord) and peripheral organs (heart, lung, liver, kidney, ovary, and testes). The *MFSD8* vector DNA was concentrated closest to the injection site in the spinal cord and detected at lower levels in multiple brain regions. In the peripheral organs, similar high amounts of *MFSD8* DNA persisted in liver and heart and to a lesser extent in testes, ovary, lung, and kidney. The pattern of CLN7 biodistribution in this study is consistent with that expected from AAV9 and observed in a previous study from our laboratory where a similar vector, scAAV9/JeT-*hGANopt*-SpA, was injected i.t. into WT rats at a dose of 6.6 × 10^11^ vg/rat (our unpublished findings). Collectively, i.t. delivery of AAV9/*MFSD8* resulted in broad *MFSD8* biodistribution across the rat body, which is considered to portray the normal biodistribution pattern expected for an AAV9 vector in rats, with vector biodistribution increasing linearly with dose. Moreover, there was no indication of reduced vector biodistribution compared with that expected, suggesting a lack of vector loss due to cellular toxicity. Clinical pathology revealed a minor immune or inflammatory stimulus, including increases in lymphocyte and leukocyte count, increases in fibrinogen and globulin concentration, and decreases in triglyceride concentration (Supplemental Table 2), which lacked definitive microscopic correlates and were mostly resolved by the end of the observation period. All animals survived to their scheduled terminal necropsies. At the end of the experiment, the tissues of all enrolled rats were sent out for blinded comprehensive histopathology evaluation, which concluded that there were no scAAV9/*MFSD8*-related macroscopic findings or microscopic changes directly attributable to the administration of the test article, except increased thymus weights in males at the high dose. Microscopically, only minimal and multifocal hemorrhages were seen in the thymus. The increased thymus weights had no clear histopathological correlation and therefore were considered to have unclear toxicological significance (Supplemental Toxicology Report 2, Charles River Laboratories). Taking these data together, administration of the scAAV9/*MFSD8* vector up to 6 × 10^12^ vg/rat resulted in dose-dependent increase of *MFSD8* vector DNA across the rat body and was not associated with any mortality, clinical observations, altered rotarod performance, BW, or food-consumption changes, further demonstrating the safety of the AAV9/*MFSD8* vector.

## Discussion

Recombinant AAV9-mediated GT has been extensively used in preclinical and clinical studies for the treatment of CNS disorders. Efficacy with AAV9 has been demonstrated in numerous preclinical models of CNS disorders and in some clinical studies using an i.t. route of administration. In addition, efficacy with AAV9 has also been demonstrated in multiple Batten diseases, including CLN3, CLN6, and CLN8, using other routes of administration (48–50). Here, we tested the feasibility and efficacy of an AAV9-based strategy to deliver the codon-optimized human *MFSD8* gene in fibroblasts from a CLN7 patient in vitro as well as in a CLN7 mouse model to investigate whether this would predict a benefit to pediatric patients with CLN7 disease.

Preclinical in vivo studies were initiated in our laboratory at the UNC Gene Therapy Center and completed at UTSW Medical Center. Administration of AAV9/*MFSD8* i.t. in KO mice, a severe mouse model of the human disease, conferred a consistent but nonsignificant trend of decreased accumulation of SCMAS and astrogliosis, major hallmarks of the underlying disease pathology. There were no findings of behavioral deficits at 2 or 4 months of age in KO-Veh animals compared with Het controls; however, there were notable behavioral deficits at 6 months of age. This is consistent with previously published data on this mouse model, in which behavioral deficits were seen starting at 6 months of age (5, 6). Some deficits in rotarod and open field were completely restored in the P7–P10 high-dose group, with a trend toward improvement in some other treatment groups (i.e., lower dose or later age of treatment). The extended life span in all treatment groups demonstrates that AAV9/*MFSD8* treatment can be effective at extending life in this animal model of Batten disease. Further, surviving treated animals could still perform similarly to Het controls on a rotarod beyond 9 months of age, suggesting that treatment can protect quality of life in addition to extending survival. Considering both the age- and dose-dependent increase in survival, along with the behavioral phenotypic rescue especially in the P7–P10 high-dose group, it is evident that a high dose at an earlier age provides the largest benefit for survival and quality of life. Previous preclinical studies of CLN6 and CLN8 diseases utilized an i.c.v. route of administration in newborn mice (48, 49). Our preclinical studies have proven the concept of i.t. AAV9/*MFSD8* gene replacement as a viable treatment strategy for the treatment of CLN7 disease when it is administered at later postnatal ages that better model intervention ages relevant to human treatment.

Studies in mice and NHPs using an AAV9 vector carrying the reporter gene GFP have demonstrated that widespread distribution of the transgene across the spinal cord, dorsal root ganglia (DRG), and brain are achievable after a single i.t. injection of the vector (34, 51). Compared with an i.v. route of administration, the i.t. route favorably directs a greater percentage of vector biodistribution to the CNS (34–38, 51–53). Notably, AAV9 does distribute to peripheral organs following an i.t. injection, but to a considerably lesser extent compared with following an i.v. route. The i.t. approach is scalable to humans, avoids anti-AAV9–neutralizing antibodies in the blood, and reduces the risk of transgene overexpression in peripheral organs. Biodistribution of AAV9/*MFSD8* in rats and qualitative mRNA expression analysis in KO mouse models following i.t. AAV9/*MFSD8* administration suggest that the distribution and expression of transgene is comparable to what has been observed with other past i.t. studies, including those with AAV9/*GAN*, which used the same JeT promoter (30). A limitation of our approach is that we would not expect meaningful biodistribution of the AAV9 vector to photoreceptors in the eye by the i.t. route. Although we did not investigate the potential for vision rescue, we would not expect this treatment approach to prevent photoreceptor degeneration and eventual vision loss. As we did not directly compare the i.t. route to an i.v. administration in this study, we cannot speculate whether additional biodistribution to peripheral organs could have led to additional treatment benefits. Combining the i.t. dose with an ocular GT, a combined i.v. administration, optimized vector administration approaches, and/or an improved capsid all represent avenues to increasing treatment efficacy. Conversely, we must consider that the lack of complete rescue of the mouse model at the high dose and early intervention we used may indicate the edge of AAV9’s ability to fully rescue a disorder when delivering a gene that acts in a cell-autonomous manner unless further steps are taken to improve the gene-transfer technology.

The non-GLP toxicology studies were conducted on juvenile WT mice, showing that the AAV9/*MFSD8* vector does not affect BW or body condition over a 1-year period of longitudinal monitoring. Further, a comprehensive blinded histopathology assessment did not find any evidence of toxicity due to AAV9/*MFSD8* at 1 year after injection. Collectively, i.t. AAV9/*MFSD8* doses up to 9.50 × 10^11^ vg/mouse were deemed safe and well tolerated in WT mice. There were no toxicities observed in either the in-life portion of the study or after microscopic examination of major tissues. If scaled to humans by cerebrospinal fluid (CSF) volume (assuming 35 μL CSF volume in mice and 140 mL CSF volume in humans), the mouse dose of 9.5 × 10^11^ vg in 5 μL would translate to a human dose of 3.8 × 10^15^ vg in a volume of 20 mL. This highest dose injected in the mice is a 3.8-fold higher titer than the highest dose proposed for humans (1 × 10^15^ total vg), in twice the volume proposed for humans. Thus, the maximum tolerated dose in mice up to 1 year after injection provides a further safety margin above what is proposed in humans.

The pivotal GLP toxicology study was conducted in normal juvenile rats by Charles River Laboratories. This GLP rat toxicology study found no significant test article–related effects on study parameters, with the exception of increased thymus weights in males at the high dose, suggesting AAV9/*MFSD8* was overall well tolerated with the i.t. doses up to 6 × 10^12^ vg/rat in WT rats. If scaled to humans by CSF volume (assuming 250 μL CSF volume in rat and 140 mL CSF volume in humans), the rat dose of 6 × 10^12^ vg in 60 μL would translate to a human dose of 3.4 × 10^15^ vg in a volume of 33.6 mL. This highest dose injected in the rats is a 3.4-fold higher titer than the highest dose proposed in humans, in over 3 times the volume proposed in humans. Thus, the maximum tolerated dose in rats up to 3 months after injection provides a further safety margin above what is proposed in humans. Based on the mouse and rat studies combined, we report a no-observed-adverse-effect level (NOAEL) for i.t. AAV9/*MFSD8* as 3.8 × 10^15^ vg (scaled human dose), although it could be higher considering that higher doses were not tested.

An identified risk of inflammatory damage to DRG is an emerging safety concern of CNS-directed AAV GT that has been reported from recent nonclinical studies in NHPs as well as piglets (54–56). In a study assessing i.t. administration of AAV across over 200 NHPs assessed in one center, minimal to mild DRG histopathology was a consistent finding across most animals, independent of the transgene (55). However, that same study noted minimal correlations to any adverse behavioral symptoms or physiological biomarkers such as altered nerve conduction. In a follow-up report, incorporation of an miRNA-binding site specific to DRG resolved this histopathology, suggesting that high transgene expression was driving the toxicity in DRG (56). Considering this putative link between transgene overexpression and DRG toxicity, there is value in our initial in vitro experiment comparing promoters, in which we concluded that the minimal synthetic JeT promoter was sufficient to rescue cellular phenotypes with no additional value in overexpressing *MFSD8* (Figure 1). We performed a long-term safety study in mice and a short-term safety study in rats; however, a notable limitation of rodent models is that they have not been demonstrated to model the DRG toxicity that has been observed in pigs and NHPs. However, our group has observed DRG histopathology in rats following i.t. administration of an unrelated AAV9 vector and transgene in another GLP safety study, suggesting that rats may be capable of modeling this DRG histopathology (data not shown). Nonetheless, after careful examination, there was no sign of DRG toxicity in the mouse and rat safety studies for AAV9/*MFSD8*.

In conclusion, the results achieved in these studies demonstrate that AAV9/*MFSD8* is both effective and well tolerated in preclinical models, providing strong proof-of-concept evidence that AAV9/*MFSD8* GT should be considered for human translation. The results presented herein and as supplemental information represent the pivotal nonclinical safety and efficacy data that supported the approval of FDA investigational new drug (IND) application 19766 and subsequent initiation of a phase I clinical trial to evaluate AAV9/CLN7 in patients with CLN7 Batten disease. Since the CSF volume of humans is relatively static after 3 years of age (57) whereas CNS cells degenerate as CLN7 disease deteriorates, we suggest a final target dose of 1 × 10^15^ vg total vg in 10 mL in CLN7 patients more than 3 years old. Higher doses could be considered and may be justified from our preclinical data, if the vector could be concentrated above 1 × 10^14^ vg/mL or if a higher injection volume could be utilized. Our data also emphasize the value of intervening as early in the disease course as possible to maximize treatment benefit. If patients 3 years old or younger were treated, it might be appropriate to scale doses lower to account for reduced brain and CSF volume. It is worth noting that while CLN7 is an LSD, MFSD8 is a membrane-bound protein, and this disease is not thought to benefit from cross correction. Thus, the conclusions from this study may translate to other AAV9-based gene replacement strategies for related neurological disorders involving genes with cell-autonomous effects.

## Methods

### Plasmid design and development.

We designed and developed the JeT-*hMFSD8opt*-SV40pA plasmid (Figure 1A) containing the transgene of a human *MFSD8* codon-optimized construct (*hMFSD8opt*). The transgene consists of a human *MFSD8* codon-optimized DNA coding sequence of 1557 bp between a 164 bp JeT promoter (30, 39) and a 123 bp SV40pA polyadenylation signal. The JeT promoter and SV40pA are utilized for their small sizes, allowing for packaging into an scAAV vector as well as mediating the minimal amount of expression needed.

### scAAV2/MFSD8 and scAAV9/MFSD8 vector preparation.

The established plasmid was packaged into scAAV2 and scAAV9 vectors (58) which are 10 to 100 times more efficient at transduction compared with traditional single-stranded AAV (ssAAV) vectors (59, 60). The scAAV2 vector was produced at the UNC Vector Core. For the cAAV9 vector, 3 lots of vectors were produced by either the UNC Vector Core or Vigene Biosciences Inc. Both research-grade vectors by 2 vendors showed equivalent in vivo biodistribution patterns, indicating similar biopotency (Figure 8B). Vigene also produced a toxicology lot of AAV9/*MFSD8* that was used in the cohorts of KO mice to assess efficacy of the GT and in WT rats to assess safety of the GT in the GLP toxicology study. The AAV2 vectors and the research-grade AAV9 vector made at UNC were titered by qPCR and confirmed by silver stain (61). The vectors produced at Vigene underwent quality control release testing. The quality control summaries of the scAAV9/*MFSD8* vectors are included in the Supplemental Information.

### In vitro CLN7 patient fibroblast culture and treatment.

An assay to measure lysosomal function in patient fibroblasts was developed previously (40, 41, 62). The fibroblasts were obtained from peripheral tissue biopsies of a human CLN7 patient with compound mutations of c.1102G→C (p.Asp368His) and i6SVA insertion (42) that were treated with the AAV2 vectors at the indicated MOIs for 5 days and loaded with cascade blue dextran at 1 mg/mL (D-1976, Life Technologies) for 1 day before the measurement of enzyme activity. All enzymatic assays were performed with *n* = 4 to 6 separate culture wells and in 2 to 3 different passages of fibroblasts. The slight difference in *n* number between total and lysosomal activity is due to occasional cell attachment/clumping issues on the bafA1-treated side, leading to exclusion of those samples.

### Mfsd8^–/–^ (KO) mice.

Two different KO mouse models (tm1a and tm1d alleles) were generated and characterized by S. Storch’s laboratory (University of Hamburg, Hamburg, Germany) (5, 6). The phenotype of the tm1a allele is much milder than the typical human disease, while the phenotype of the tm1d allele is comparable to the human CLN7 clinical presentation. Therefore, the tm1d allele mouse model was used in our preclinical studies. The tm1d-KO mice were generated by targeted deletion of exon 2 in the *Mfsd8* gene, which recapitulates key features of human CLN7 disease (6). tm1d-KO mice were identified by toe tattooing at P7–P10 and then randomized into treatment groups based on the ID numbers assigned to them at genotyping.

### Efficacy study plan in KO mice.

The experimental design for the in vivo efficacy study is summarized in Figure 2A. In brief, equal numbers of male and female KO mice were injected i.t. at P7–P10 (presymptomatic cohorts) or P120 (early symptomatic cohorts). For i.t., 5 μL of high (5 × 10^11^ vg/mouse) or low (1.25 × 10^11^ vg/mouse) dose of scAAV9/*MFSD8* vector was administrated via lumber puncture. All mice were weighed weekly up to 4 weeks of age and monthly thereafter, as well as observed for overt signs of adverse effects at the times of weighing. The survival rate was calculated, and cause of mortality was investigated by a veterinary staff whenever possible. Behavioral testing was performed blindly on all study cohorts at 2, 4, 6, 9, 12, 15, and 18 months of age. At 4.5 months of age, 3 males and 3 females from each cohort treated at P7–P10 were sacrificed to evaluate GCase activity, vector biodistribution, *MFSD8* mRNA expression by RNAscope, and early histological signs of treatment efficacy by IHC. All remaining mice were maintained to evaluate long-term survival, behavioral phenotypes, and safety until they required humane euthanasia or reached 24 months of age, the planned end point of the experiment.

### Tissue preparation for GCase activity, vector biodistribution, RNAscope, and IHC staining.

At necropsy, animals were deeply anesthetized via an i.p. injection of a 2.5% avertin solution in normal saline. Animals were perfused for 5 minutes with 1× PBS containing 1 U/mL heparin. The right halves of tissues were harvested for GCase activity in brain lysates (40, 41) and vector biodistribution in all tissues collected (63). The left halves of tissues were harvested and fixed in 10% neutral-buffered formalin (NBF) for 24 hours and transferred to 70% ethanol. Tissues were then processed, embedded in paraffin, and cut into 5 μm sections. Separate sections were used for RNAscope to detect *MFSD8* mRNA or IHC to stain for SCMAS, GFAP (64), CD68, and NeuN.

### Image analysis.

All stained slides with 1 section for each animal were digitized with a ScanScope slide scanner (Aperio Technologies). Scanned slides were viewed with the ImageScope software package (version 10.0, Aperio Technologies) and analyzed using custom analysis settings in HALO Image Analysis Platform (Halo2.2, Indica Labs). A region of interest (ROI) was hand drawn on each image to allow for analysis by tissue region. Within the brain, regions were drawn around the whole brain, cortex, hippocampus, cerebellum, subcortex, and brain stem. For spinal cord samples, the entire tissue area was analyzed. A threshold for each stain was set using positive and negative control images, and the same analysis settings were applied for every image of the same stain. Percentages of area staining for each marker of interest were recorded for each tissue and ROI. Fiji ImageJ (NIH) software was used to count NeuN^+^ cells and hematoxylin-stained nuclei. Total cells (NeuN^+^ cells plus hematoxylin-stained nuclei) and the NeuN^+^/total cells ratio were calculated. Analysis was done with the observer blinded to the treatment group of each sample.

### Behavioral tests.

Animals were assessed in a battery of behavioral tests repeated at 2, 4, 6, 9, 12, 15, and 18 months of age. Rotarod and open-field tests were performed at 2 to 18 months of age, while marble-burying and wire-hang tests were performed at 2 to 6 months of age. All behavioral tests were conducted by personnel blind to the genotype and treatment of the mice.

### Non-GLP safety study in WT BL/6J mice.

The non-GLP studies presented in Figure 8A were designed to identify any long-term safety issues of the experimental therapy. The mice were randomized to different groups and injected i.t. with 5 μL of vehicle or different doses of AAV9/*MFSD8* vectors. Two lots of research-grade AAV9/*MFSD8* vectors were made by the UNC Vector Core or Vigene, and both were used in parallel safety studies. Mice were monitored following the treatment, and appropriate supportive or therapeutic interventions were offered. A detailed necropsy was performed to investigate the reason or reasons for the ailments. Terminal tissue samples including brain, heart, liver, lung, gonad, spleen, kidney, eyeball, sciatic nerve, cervical spinal cord, and lumbar spinal cord at 12 months following the treatment were collected for histopathological assessment. The final histopathological evaluation on collected tissue samples was performed and reported by Mary Wight-Carter (Supplemental Information).

### Safety study in WT CD rats in a GLP study.

This animal study was performed by Charles River Laboratories. Male and female CD rats were randomized into cohorts, with 5 males and 5 females per cohort, and dosed as shown in Figure 9A. At the initiation of dosing, the animals assigned to study were approximately 56 to 63 days old and weighed between 165 g and 328 g. AAV9/*MFSD8* vector was injected i.t. once in each animal by a qualified laboratory technician, in a volume of 20 or 60 μL at a final dose of 5 × 10^11^, 2 × 10^12^, or 6 × 10^12^ vg/rat. All animals were monitored up to 90 days following the injection. Rats were sacrificed on day 7, 28, or 90 after injection, and tissues were collected for biodistribution and toxicity evaluation. For biodistribution, total genomic DNA was purified from tissue samples collected at necropsy day 28, using a QIAGEN QIAcube HT. qPCR was used to determine the quantity of the *MFSD8* transgene per diploid rat genome. Details of this study are provided in Charles River Laboratories’ final report, provided as supplemental material.

### Statistics.

All quantitative data in this paper were presented as mean ± SEM, analyzed, and graphed using GraphPad Prism Software (version 9.2.0). A ROUT test was used first to remove any outlier. Only 2 outliers were excluded from calculation based on the ROUT test (Supplemental Table 1). Data were then tested for normal distribution (Shapiro-Wilk normality test) and homogeneity of variance (Brown-Forsythe test). Data sets that passed these 2 tests were analyzed using Student’s 2-tailed, unpaired *t* test for 2-group comparison or 1-way ANOVA for equal or more than 3-group comparison with Dunnett’s correction for relevant pairwise comparisons. Data sets that did not pass tests for normality or homogeneity of variance were analyzed using the Mann-Whitney *U* test for 2-group comparison or the Kruskal-Wallis test with Dunn’s correction for relevant pairwise comparisons. For survival analysis, data shown in the Kaplan-Meier survival curve were compared with the log-rank (Mantel-Cox) test. Two-way ANOVA with repeated measures was used for BW analyses. *P* < 0.05 was considered significant for all statistical analyses.

For more detailed information, refer to Supplemental Methods.

### Study approval.

All studies on mice were approved by the IACUC of the UNC at Chapel Hill or the UTSW Medical Center. The in-life GLP toxicity study was performed at Charles River Laboratory and was approved by their IACUC. CLN7 fibroblast patient consent information has been described previously (42)

## Author contributions

XC and SJG designed the experiments. XC, FCS, and SJG coordinated studies with collaborators and core facilities and wrote the manuscript. XC, TD, YH, FCS, NRB, and JRM performed the experiments. XC, TD, and FCS analyzed all data and prepared all figures for the manuscript. YH, NRB, and JRM helped prepare the manuscript. SJG oversaw all activities related to the project and acquired all funding for the work.

## Supplementary Material

Supplemental data

Supplemental Toxicology Report 1

Supplemental Toxicology Report 2

## Version 1 01/13/2022

In-Press Preview

## Version 2 03/01/2022

Electronic publication
