# Supplementary material for: AAV9/MFSD8 gene therapy is effective in preclinical models of neuronal ceroid lipofuscinosis type 7 disease
Source: J Clin Invest. 2022 Mar 1;132(5):e146286. doi: 10.1172/JCI146286 (PMC8884910; doi:10.1172/JCI146286)
Supplement: Supplemental data [file jci-132-146286-s024.pdf]

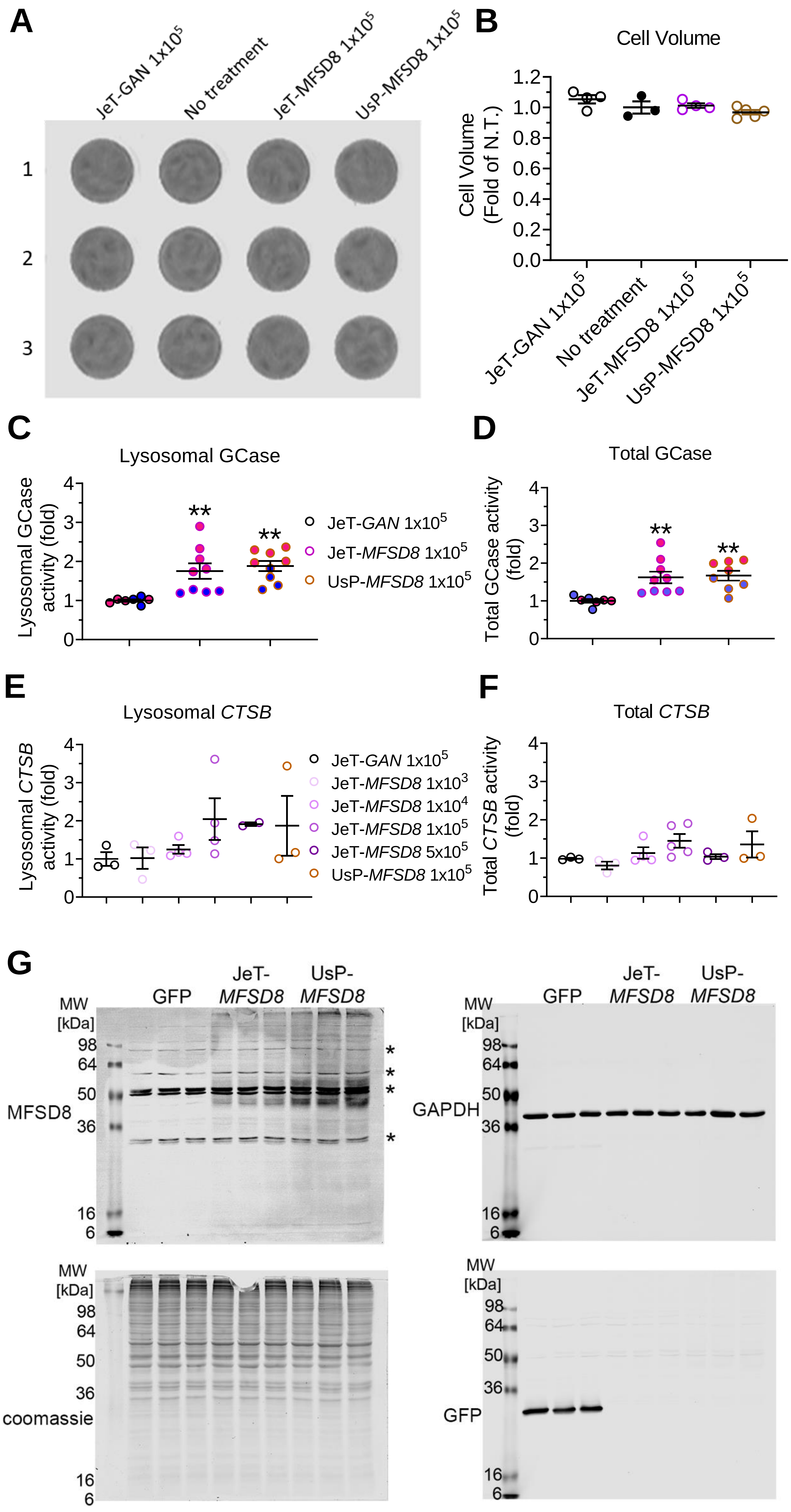

Supplemental Figure 1. The effects of AAV2/*MFSD8* vector on cell viability (A-B), GCase activity (C-D), *CTSB* activity (E-F), and MFSD8 protein (G) in primary fibroblasts from CLN7 patient. In **A** and **B,** fibroblasts were not infected, infected with Jet-*GAN*, Jet-*MFSD8*, or UsP-*MFSD8*, fixed, and stained with a cell volume indicator (CellTag700) at 6 days post infection. Representative image of cultures analyzed in a 96 well format is shown (**A**). Quantification of cell volume is represented as fold change of no treatment cells (**B**). In **C** and **D,** two independent experiments are represented by red or blue fill. Lysosomal and total GCase activity (**C** and **D**, n=7-9) or lysosomal and total *CTSB* activity (**E** and **F**, n=2-5) were measured in fibroblasts from a CLN7 patient following AAV2-mediated transduction of JeT-*GAN* (negative control), JeT-*MFSD8* (therapeutic transgene at increasing doses), or UsP-*MFSD8* (therapeutic transgene with stronger promotor). Both GCase and *CTSB* activities were normalized to the cell volume and to cohorts transfected with Jet-*GAN*. A ROUT test was used first to remove any outlier. All data in **B**-**F** are presented as mean ± SEM with the scatter plot representing measurements from individual culture well. Data sets that passed tests for normality or homogeneity of variance were analyzed using one-way ANOVA with α set at 0.05 and Dunnett's correction for relevant pairwise comparisons. Data sets that did not pass tests for normality or homogeneity of variance were analyzed using Kruskal-Wallis test with α set at 0.05 and Dunn's correction for relevant pairwise comparisons. **$p<0.01$ compared to control. In **G**, Western blot analysis of cell lysates (n=3) from cell culture treated with CBh-*GFP* (negative control), JeT-*MFSD8*, or UsP-*MFSD8*. Asterisks indicate putative non-specific bands and were not used in quantification. The same membrane that was probed for MFSD8 protein was sequentially probed for GAPDH and GFP using fluorescent-conjugated secondary antibodies. The coomassie stain shows total protein loaded from the corresponding gel used for the MFSD8 blot. GCase, beta-glucocerebrosidase; *CTSB*, Cathepsin B; GAPDH, Glyceraldehyde-3-phosphate dehydrogenase; GFP, Green fluorescent protein.

Supplemental Table 1. Raw data of RNAscope for *MFSD8* mRNA, and IHC staining for SCMAS, GFAP, and CD68

		RNAscope for MFSD8 mRNA expression					IHC for SCMAS					IHC for GFAP					IHC for CD68			
Treatment	mouse ID	Whole brain	Cortex	Hippocampus	Cerebellum	Spinal cord	Whole brain	Cortex	Hippocampus	Cerebellum	Spinal cord	Whole brain	Cortex	Hippocampus	Cerebellum	Spinal cord	Whole brain	Cortex	Hippocampus	Cerebellum
Het	CLN71D 28.57	0.0001	0.0002	0.0000	0.0000	0.0000	0.3961	0.6204	0.0803	1.4049	0.0597	0.2365	0.0607	0.3835	0.2719	*8.2397**	1.0101	0.0008	0.1267	1.6527
	CLN71D 28.60	0.0000	0.0000	0.0001	0.0000	0.0001	0.7632	0.3539	0.0738	2.3555	0.0619	0.2215	0.1002	0.4023	0.2403	2.3899	0.6118	0.0059	0.3823	4.0041
	CLN71D 28.79	0.0001	0.0000	0.0002	0.0000	0.0001	2.2236	1.0555	0.1712	6.9533	0.0088	0.1432	0.0963	0.2915	0.0944	1.9955	1.3459	0.0011	0.0683	3.2287
	CLN71D 35.03	0.0001	0.0001	0.0000	0.0002	0.0000	0.6658	0.5584	0.2565	2.0139	0.1616	0.1688	0.0507	0.2293	0.3068	2.8586	0.6916	0.0029	0.9711	1.9321
	CLN71D 35.06	0.0000	0.0000	0.0000	0.0000	0.0000	1.5346	0.5565	0.1057	5.1853	0.0411	0.1582	0.0500	0.3650	0.1074	2.1775	2.1094	0.0012	0.1252	3.4501
	CLN71D 48.63	0.0000	0.0001	0.0000	0.0000	0.0000	7.6354	1.2512	*0.9084**	16.1151	0.0600	0.2359	0.1027	0.3849	0.3586	3.4992	1.2751	0.0028	0.1546	3.2654
	**Mean**	**0.0001**	**0.0001**	**0.0001**	**0.0000**	**0.0000**	**2.2031**	**0.7326**	**0.1375**	**5.6713**	**0.0655**	**0.1940**	**0.0768**	**0.3427**	**0.2299**	**2.5841**	**1.1740**	**0.0025**	**0.3047**	**2.9222**
KO-Veh	CLN71D 28.55	0.0000	0.0000	0.0000	0.0000	0.0000	1.0037	1.9165	0.7419	2.5871	0.8578	1.6235	1.8419	2.1634	1.2134	3.8522	1.1584	0.0013	0.0299	2.4342
	CLN71D 28.59	0.0001	0.0004	0.0000	0.0000	0.0000	1.2136	2.7898	1.3183	2.9802	0.6152	0.5876	0.8440	0.5787	0.4447	5.0736	0.8188	0.0093	0.0119	2.0580
	CLN71D 28.78	0.0000	0.0001	0.0000	0.0000	0.0005	0.5236	1.7896	0.8279	0.9477	0.6931	0.4216	0.3800	0.6326	0.3662	3.4563	0.4994	0.0036	0.0118	0.7264
	CLN71D 35.05	0.0001	0.0000	0.0000	0.0000	0.0006	1.6045	2.6176	1.0051	3.8860	1.2568	0.3657	0.4777	0.2584	0.6713	4.6006	1.4969	0.0007	0.4467	2.6723
	CLN71D 49.73	0.0000	0.0001	0.0000	0.0000	0.0004	3.4469	2.3660	0.6510	10.8562	0.5212	0.2269	0.2134	0.8859	0.1404	3.1857	0.5372	0.0007	0.1244	3.0316
	CLN71D. 52.84	0.0000	0.0000	0.0001	0.0000	0.0004	4.3548	3.3586	1.2424	13.1285	0.4855	0.5214	0.4762	0.4417	0.3327	2.5669	0.6997	0.0018	0.3058	0.4546
	**Mean**	**0.0000**	**0.0001**	**0.0000**	**0.0000**	**0.0003**	**2.0245**	**2.4730**	**0.9644**	**5.7309**	**0.7383**	**0.6245**	**0.7055**	**0.8268**	**0.5281**	**3.7892**	**0.8684**	**0.0029**	**0.1551**	**1.8962**
KO-Low	CLN71D 28.56	0.0552	0.0662	0.0751	0.0375	0.1741	4.6698	2.1641	1.5322	14.4983	1.0333	0.9954	1.3660	0.8399	1.0086	3.5546	0.9362	0.0043	0.0870	2.4883
	CLN71D 28.61	0.0326	0.0209	0.0125	0.0714	0.2420	3.1596	1.8058	0.5358	9.5496	0.3843	0.6371	0.6820	0.7568	0.6028	5.3193	0.7228	0.0013	0.0116	1.9807
	CLN71D 28.80	0.0216	0.0178	0.0107	0.0285	0.0067	3.8975	2.2442	0.8280	13.3540	0.4760	0.4277	0.5394	0.6396	0.3575	4.9073	0.7161	0.0034	0.2209	1.3369
	CLN71D 48.65	0.0083	0.0142	0.0007	0.0106	0.0000	8.2354	4.2406	2.7566	25.3060	0.4152	0.4697	0.5928	0.5381	0.4154	3.6668	0.4792	0.0002	0.1091	2.5572
	CLN71D 48.70	0.0000	0.0000	0.0000	0.0000	0.0955	6.1258	3.3677	1.3562	19.2712	1.8437	0.5234	0.8868	0.6403	0.3188	4.4080	1.1211	0.0006	0.3473	2.1590
	CLN71D. 52.85	0.1030	0.0983	0.0610	0.1227	0.0350	5.2631	1.3643	0.6991	17.0779	0.2782	0.2122	0.2055	0.2634	0.3181	4.0349	1.5246	0.0021	0.0558	3.5780
	**Mean**	**0.0368**	**0.0362**	**0.0267**	**0.0451**	**0.0922**	**5.2252**	**2.5311**	**1.2847**	**16.5095**	**0.7384**	**0.5443**	**0.7121**	**0.6130**	**0.5035**	**4.3151**	**0.9167**	**0.0020**	**0.1386**	**2.3500**
KO-High	CLN71D 28.58	0.3110	0.2609	0.3112	0.4546	0.2477	3.1365	1.9333	0.6619	6.9668	0.3518	0.2285	0.2266	0.4230	0.1662	3.8516	0.6974	0.0019	0.0811	2.4147
	CLN71D 28.77	0.1623	0.1817	0.0647	0.2742	0.6220	3.4584	1.2655	0.4048	10.6433	0.1788	0.2351	0.2331	0.3571	0.2151	3.3645	0.7278	0.0012	0.0251	1.3583
	CLN71D 35.01	0.1864	0.1496	0.1515	0.2771	0.8623	4.9854	1.6526	0.6974	14.7292	0.4030	0.2958	0.6509	0.2789	0.3721	4.4300	0.9264	0.0162	0.0987	1.5386
	CLN71D 48.66	0.0863	0.0936	0.0782	0.0926	0.1535	3.2165	1.3634	0.8944	9.4734	0.3165	0.3263	0.4375	0.4547	0.3021	3.6979	1.3213	0.0057	0.0644	3.2308
	CLN71D 49.71	0.2031	0.3002	0.1060	0.2399	0.5698	4.1587	1.7378	0.9155	12.0425	0.4625	0.4189	0.9285	0.4473	0.2373	2.1651	1.3689	0.0008	0.3119	2.7156
	CLN71D.52.86	0.0000	0.0000	0.0000	0.0000	0.0000	7.3646	1.6452	1.0840	19.3647	0.9399	0.1568	0.2919	0.2112	0.1660	1.3981	1.0785	0.0014	0.3667	2.1023
	**Mean**	**0.1582**	**0.1643**	**0.1186**	**0.2231**	**0.4092**	**4.3867**	**1.5996**	**0.7763**	**12.2033**	**0.4421**	**0.2769**	**0.4614**	**0.3620**	**0.2431**	**3.1512**	**1.0201**	**0.0045**	**0.1580**	**2.2267**
						Mean KO-Veh - Het		1.7404	0.8269	0.0596	0.6728		0.6288	0.4840	0.2982	1.2051				
						Mean KO-Low - Het		1.7985	1.1472	10.8382	0.6729		0.6353	0.2703	0.2737	1.7310				
						Mean KO-High - Het		0.8670	0.6388	6.5320	0.3766		0.3847	0.0193	0.0133	0.5670				
						X=(Mean KO-High - Het)*100/(Mean KO-Veh - Het)		49.8	77.3	10955.6	56.0		61.2	4.0	4.4	47.1				
						Y=100-X		50.2	22.7	-10855.6	44.0		38.8	96.0	95.6	52.9				

Note: the blue font and asterisk in this table are the two outliers based on ROUT test and therefore excluded from the calculation.

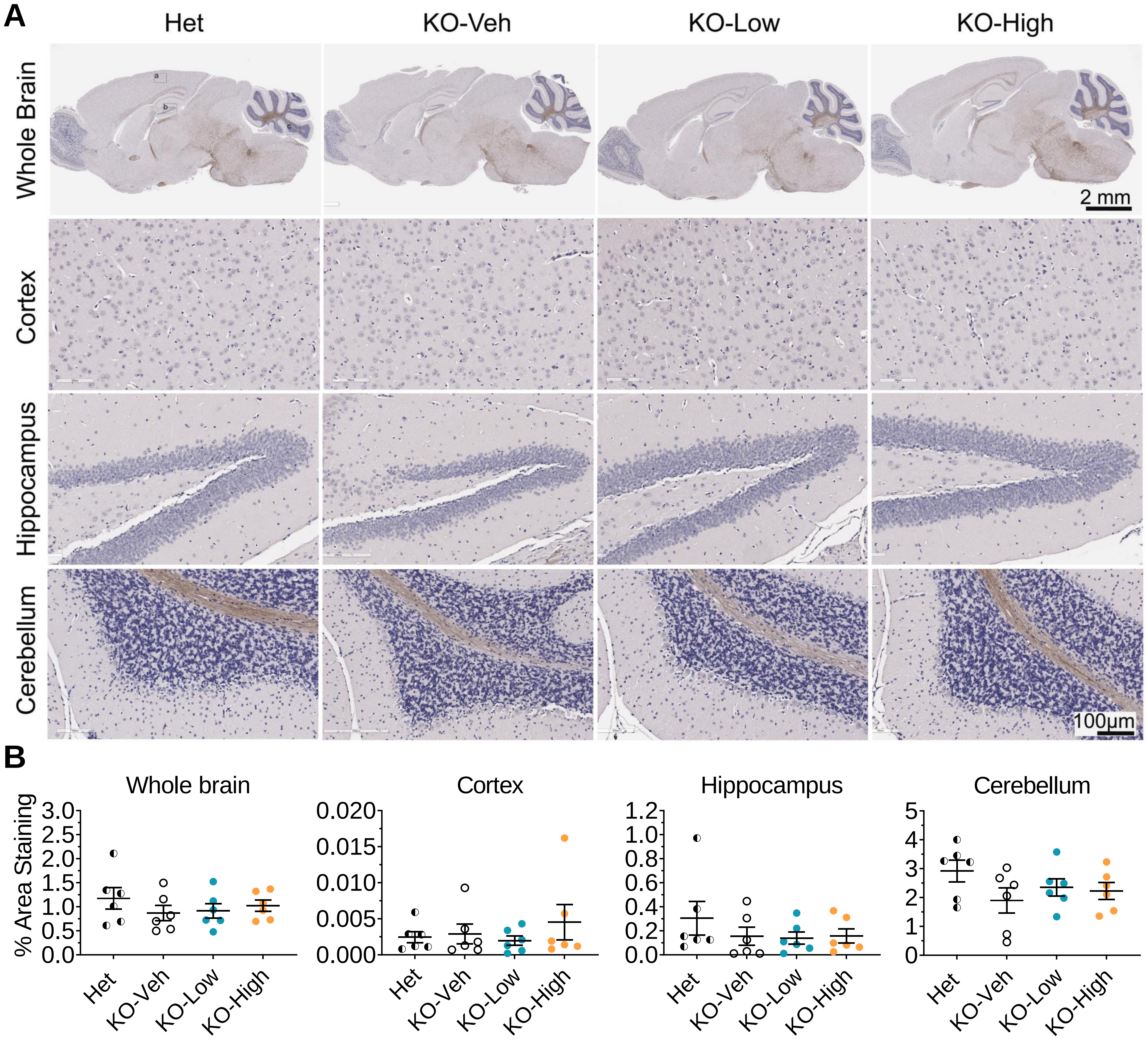

Supplemental Figure 2. No increased IHC staining of CD68 in brains of *Mfsd8*$^{-/-}$ mice at the age of 4.5 months old. High (5x10^{11} vg/mouse) or low (1.25x10^{11} vg/mouse) dose of AAV9/*MFSD8* vector was administered intrathecally to balanced male and female mice at postnatal day p7-10. At 4.5 months old, mouse brains were harvested for IHC staining to detect CD68 (**A**). Histology images with 1 section/animal were digitized with a ScanScope slide scanner and analyzed using custom analysis settings in HALO™ Image Analysis Platform. Results are presented as % area staining positive for CD68 by brain region (**B**). A ROUT test was used first to remove any outlier. Each data point represents measurement from an individual animal (n=5-6), with lines representing the mean measurement ± SEM. Data sets that passed tests for normality or homogeneity of variance were analyzed using one-way ANOVA with α set at 0.05 and Dunnett's correction for relevant pairwise comparisons. Data sets that did not pass tests for normality or homogeneity of variance were analyzed using Kruskal-Wallis test with α set at 0.05 and Dunn's correction for relevant pairwise comparisons. No significance was observed. KO-Veh, KO mice receiving vehicle; KO-Low, KO mice receiving low dose; KO-High, KO mice receiving high dose; IHC, immunohistochemistry; CD68, cluster of differentiation 68. Scale bars for Whole brain, 2mm; Scale bars for Cortex, Hippocampus, and Cerebellum, 100µm.

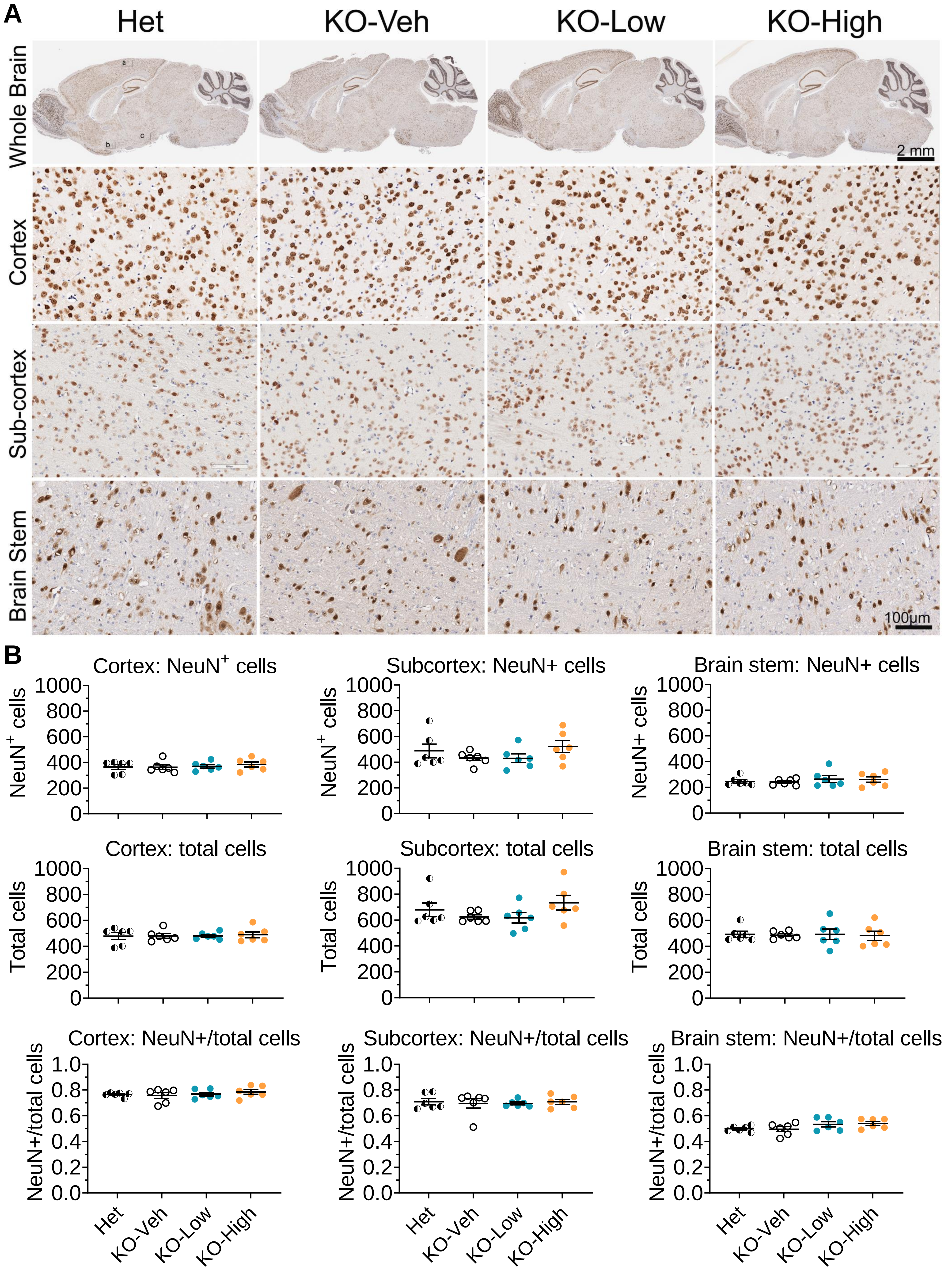

Supplemental Figure 3. No decreased cell numbers in brains of *Mfsd8*$^{-/-}$ mice at the age of 4.5 months old. A high ($5x10^{11}$ vg/mouse) or low ($1.25x10^{11}$ vg/mouse) dose of AAV9/*MFSD8* vector was administered intrathecally to balanced male and female mice at postnatal day p7-10. At 4.5 months old, mouse brains were harvested for IHC staining for NeuN (**A**). Histology images with 1 section/animal were digitized with a ScanScope slide scanner and cell numbers were counted with image J. Results are presented as NeuN^{+} cells, total cells, and NeuN^{+}/total cells (**B**). A ROUT test was used first to remove any outlier. Each data point represents measurement from an individual animal (n=5-6), with lines representing the mean measurement ± SEM. Data sets that passed tests for normality or homogeneity of variance were analyzed using one-way ANOVA with α set at 0.05 and Dunnett's correction for relevant pairwise comparisons. Data sets that did not pass tests for normality or homogeneity of variance were analyzed using Kruskal-Wallis test with α set at 0.05 and Dunn's correction for relevant pairwise comparisons. No significance was observed. KO-Veh, KO mice receiving vehicle; KO-Low, KO mice receiving low dose; KO-High, KO mice receiving high dose; IHC, immunohistochemistry. Scale bars for Whole brain, 2mm; Scale bars for Cortex, Sub-cortex, and Brain Stem, 100μm.

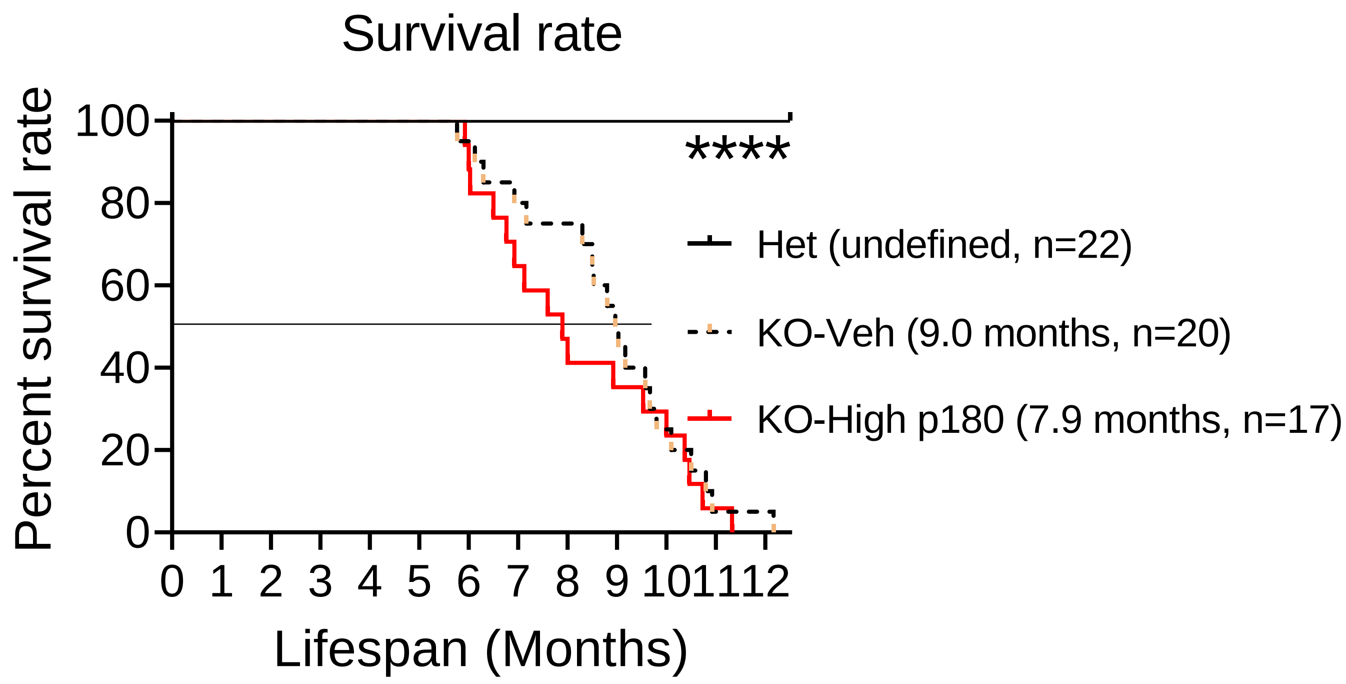

Supplemental Figure 4. AAV9/*MFSD8* GT does not extend lifespan of *Mfsd8*$^{-/-}$ mice when treated at the age of 6 months old. High ($5x10^{11}$ vg/mouse) dose of AAV9/*MFSD8* vector was administered intrathecally to *Mfsd8*$^{-/-}$ mice at p180 (n=17-22). Kaplan-Meier survival curve shows the survival over time with median survival and mice number enrolled in parenthesis. Data were compared with Log-rank (Mantel-Cox) test. ****p<0.0001 compared to KO-Veh. KO-Veh, KO mice receiving vehicle; KO-High, KO mice receiving high dose.

Supplemental Table 2. The minimal adverse effects of treatment in WT rats in this GLP toxicity study.

	Male			Female		
	$5x10^{11}$	$2x10^{12}$	$6x10^{12}$	$5x10^{11}$	$2x10^{12}$	$6x10^{12}$
↑ lymphocyte by 16~71%		Yes[a]	Yes[a]		Yes[a]	Yes
↑ leukocyte by10~60%		Yes[a]	Yes[a]		Yes[a]	Yes
↑ fibrinogen by ~25%			Yes[b]			
↑ globulin by 5~12%		Yes[a]	Yes	Yes[a]	Yes[a]	Yes
↓ triglyceride by 18~50%	Yes[b]	Yes[b]	Yes[b]			

a: partially resolved by the end of the observation period.
b: resolved by the end of the observation period.

COA of UNC-VC product: LAV45 lot

UNIVERSITY OF NORTH CAROLINA
VECTOR CORE

Quality Control Summary

Lot #	LAV45-final

Test by qPCR

Test #	Titer, vg/mL	Analyst	Date	File
1	1.87E+14	PZ	11/01/2017	20171101-1531-ghbh-pz

PAGE analysis

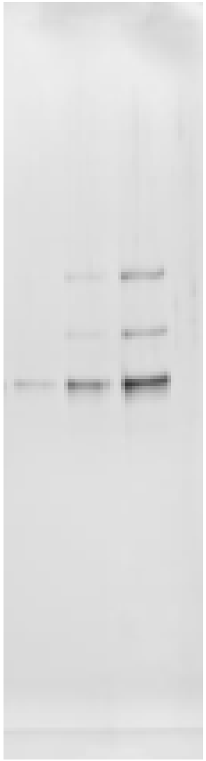

Loaded 5.00E+09 vg   4980E std   2e9vg   5e9vg   1e10vg
Calculated 4.00E+09 vg

Analyst	Ping Zhang
Date	11/03/2017
Reference #	20171103-silver

UNIVERSITY OF NORTH CAROLINA
VECTOR CORE

SEM (before concentration)

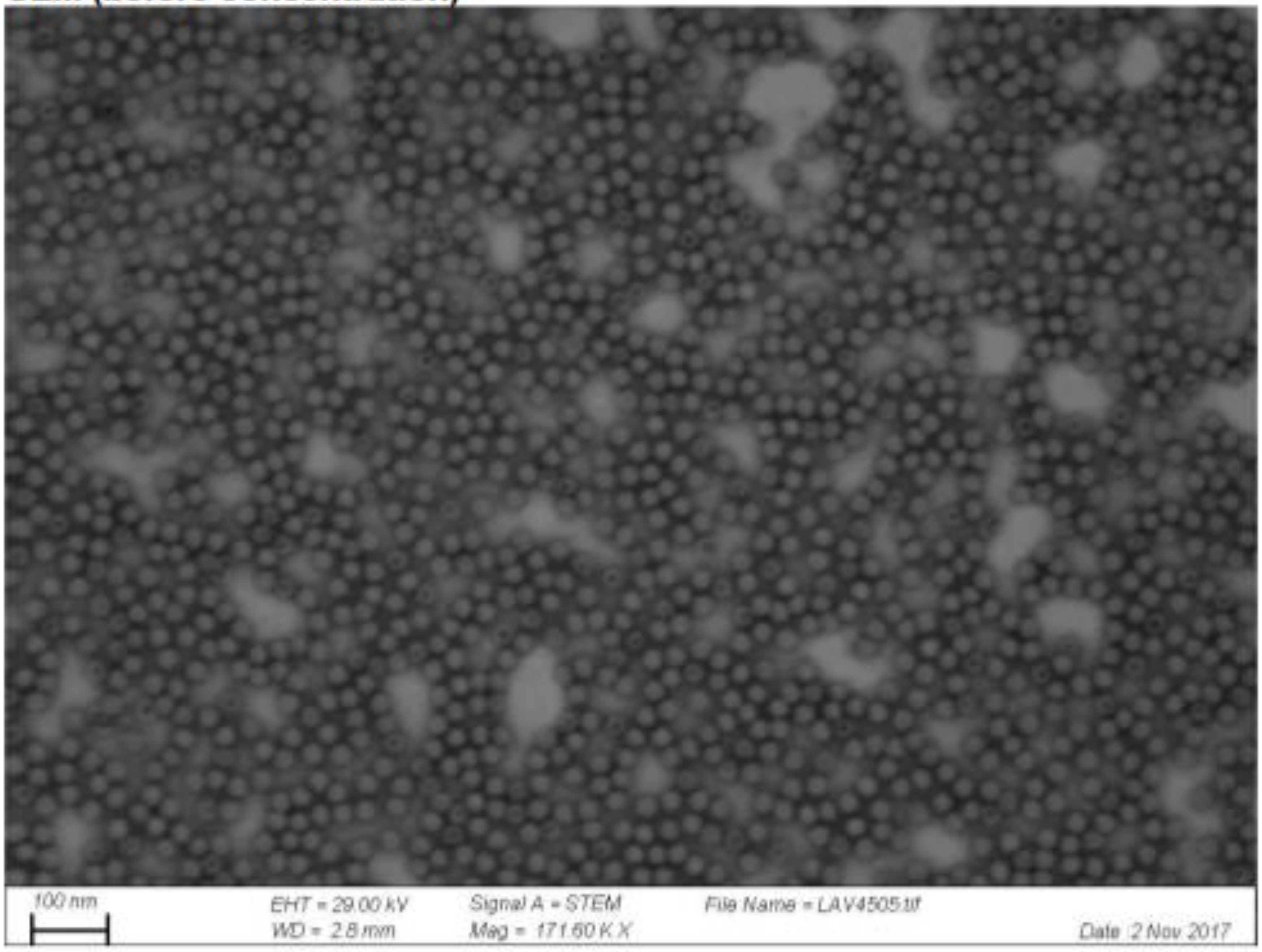

85% full

Analyst	Ping Zhang
Date	11/02/2017
Reference #	20171102-lav45-05

COA of Vigene product : Research Grade lot

12111 Parklawn Drive
Rockville, MD 20852
(301)251-6638
www.vigenebio.com

Vigene Biosciences
Excellence in Gene Delivery

PRODUCT INFORMATION & CERTIFICATE OF ANALYSIS

PRODUCT INFORMATION

Research Grade scAAV9-CLN7 virus production and purification

Date: September 13th 2017

SHELF LIFE: 2 years from date of receipt under proper storage conditions

SHIPMENT SPECIFICATION & HANDLING INSTRUCTION

Quantity	Description	Volume/titer
**1**	**AAV9/CLN7**	**4X 250µl, 9.80 1E13 gc/ml**

FORMULATION BUFFER

PBS, 5% Sorbitol, pH 7.4 containing 0.001%F-68

SHIPPING&STORAGE CONDITIONS

Product shipped on dry ice. Upon receiving, please store at -80 degrees for long-term storage.

HANDLING INSTRUCTION & PRODUCT MANUAL

For detailed information regarding the vectors and product manual for the corresponding product, please visit our website at http://www.vigenebio.com.

Senior PD Director Man-shiow Jiang, Ph.D.

12111 Parklawn Drive
Rockville, MD 20852
(301)251-6638
www.vigenebio.com

Vigene Biosciences
Excellence in Gene Delivery

NOTE TO PURCHASE

Vigene Biosciences' products are to be used for research purpose only. They may not be used for any other purposes, including, but not limited to, in vitro diagnostic purposes, therapeutics, or in humans. **Vigene Biosciences'** products may not be transferred to any third parties, resold, modified for resale, or used to manufacture commercial products or to provide a service to other third parties without prior written approval from Vigene Biosciences, Inc.
Your use of this product is also subject to compliance with the licensing requirements listed above and described on the product's web page at http://www.vigenebio.com. It is your responsibility to review, understand and adhere to any restrictions imposed by these statements. ≥

Senior PD Director Man-shiow Jiang, Ph.D.

COA of Vigene product: Tox Lot

12111 Parklawn Drive
Rockville, MD 20852
(301)251-6638
www.vigenebio.com

Vigene Biosciences
Excellence in Gene Delivery

PRODUCT INFORMATION & CERTIFICATE OF ANALYSIS

PRODUCT INFORMATION

Tox Lot scAAV9-CLN7 virus production and purification

Date: September 8th 2018

SHELF LIFE: 2 years from date of receipt under proper storage conditions

SHIPMENT SPECIFICATION & HANDLING INSTRUCTION

Quantity	Description	Volume/titer
**50**	**AAV9/CLN7**	**200µl X 50<br>1.09+14GC /mL**

FORMULATION BUFFER

PBS, 5% Sorbitol, pH 7.4 containing 0.001%F-68

Test	Specification	Result	Unit
**Viral genome titer (qPCR)**	**≥1E+14 GC/mL**	**1.09E+14 (±1.76E+13)**	**GC/mL**
**Residual Host Cell Protein**	**Report result**	**<LOD**	**ng/mL**
**Residual HCD**	**Report result**	**369±96**	**ng/mL**
**Residual Benzonase**	**Report result**	**<LOD (0.7)**	**ng/mL**
**Endotoxin**	**<10**	**2.7±0.8**	**ng/mL**
**pH**	**7.4±0.3**	**7.4**	
**Appearance**	**Clear**	**Clear**	

Senior PD Director: MAN-SHIOW JIANG, PH.D.

12111 Parklawn Drive
Rockville, MD 20852
(301)251-6638
www.vigenebio.com

Vigene Biosciences
Excellence in Gene Delivery

PRODUCT LABEL

CLN-7 Vial#01/50

P/N: TOX-CLN-7, L/N: MSJ-18-01-16-2

0.2mL/vial, ≥1.0E14VG/mL

Store ≤-70°C DOM: 07 Sep 2018

Prepared for BHF by Vigene Biosciences,

Caution: R&D Use Only

SHIPPING&STORAGE CONDITIONS

Product shipped on dry ice. Upon receiving, please store at -80 degrees for long-term storage.

HANDLING INSTRUCTION & PRODUCT MANUAL

For detailed information regarding the vectors and product manual for the corresponding product, please visit our website at http://www.vigenebio.com.

Senior PD Director: MAN-SHIOW JIANG, PH.D.

12111 Parklawn Drive
Rockville, MD 20852
(301)251-6638
www.vigenebio.com

Vigene Biosciences
Excellence in Gene Delivery

NOTE TO PURCHASE

Vigene Biosciences' products are to be used for research purpose only. They may not be used for any other purposes, including, but not limited to, in vitro diagnostic purposes, therapeutics, or in humans**. Vigene Biosciences'** products may not be transferred to any third parties, resold, modified for resale, or used to manufacture commercial products or to provide a service to other third parties without prior written approval from Vigene Biosciences, Inc.
Your use of this product is also subject to compliance with the licensing requirements listed above and described on the product's web page at http://www.vigenebio.com. It is your responsibility to review, understand and adhere to any restrictions imposed by these statements. ≥

Senior PD Director: MAN-SHIOW JIANG, PH.D.
