# Supplementary material for: AAV9/MFSD8 gene therapy is effective in preclinical models of neuronal ceroid lipofuscinosis type 7 disease
Source: J Clin Invest. 2022 Mar 1;132(5):e146286. doi: 10.1172/JCI146286 (PMC8884910; doi:10.1172/JCI146286)
Supplement: Supplemental Toxicology Report 1 [file jci-132-146286-s025.pdf]

REPORT

A Single Dose Toxicity Study of AAV9/CLN7 Vectors Administered by Intrathecal Injection in WT C57BL/6J Mice

CONTENTS

Abbreviations 2
Introduction 3
Materials and methods 3
Study Design 3
Results and Discussion 4
Body Weight 4
Clinical Signs or Adverse Events 5
Survival Rate 5
Histopathology 6
Conclusions 26
Appendix 27
UNC-VC Vector Certificate of Analysis 27
Vigene Vector Certificate of Analysis 29

List of Figures

Figure 1. In vivo equivalence of UNC and Vigene preclinical lots of AAV9/CLN7 vectors 4
Figure 2. No significant difference of body weight between groups within male or female mice. 5
Figure 3. No significant difference of survival rate between groups within male or female mice. 5

List of Tables

Table 1. Summary of the non-GLP cohorts 3
Table 2. Summary of mouse sex, group, and ID number 7
Table 3. Mean and individual body weight (g) 9
Table 4. Histopathology of mice injected IT with vehicle 16
Table 5. Histopathology of mice injected IT with $9.50x10^{11}$ vg/mouse of AAV9/CLN7 vectors 18
Table 6. Histopathology of mice injected IT with $4.47x10^{11}$ vg/mouse of AAV9/CLN7 vectors 20
Table 7. Histopathology of mice injected IT with $1.48x10^{11}$ vg/mouse of AAV9/CLN7 vectors 22
Table 8. Histopathology of mice injected IT with $4.47x10^{10}$ vg/mouse of AAV9/CLN7 vectors 24

ABBREVIATIONS

AAV9	adeno-associated virus serotype 9
CLN7	ceroid lipofuscinosis, neuronal type 7 (The variant late infantile neuronal ceroid lipofuscinosis, vLINCL)
IT	intrathecal
UNC-VC	University of North Carolina – Vector Core
vg	vector genomes
WT	wild type

INTRODUCTION

The objective of this study was to characterize the toxicity and gene expression of scAAV9/Jet-hCLN7opt-SV40pA (AAV9/CLN7) following a single intrathecal (IT) injection in wild type (WT) CH57BL/6J mice. The AAV9/CLN7 is being developed for treatment of the neurodegenerative Batten disease (CLN7 subtype).

MATERIALS AND METHODS

Study Design

WT CH57BL/6J mice from Jackson Laboratories were assigned to the study as indicated in Table 1.

Table 1. Summary of the non-GLP cohorts

Group	Route*	Age (weeks)	Body weight (g)**		Dose (vg/mouse)	Vector manufacturer	Endpoint (12 months post injection)
			Male (n=5)	Female (n=5)			
1	IT	8	32.4±1.1	21.1±0.8	Vehicle	UNC-VC	Body weights, Clinical signs, Adverse events, Mortality, and Histopathology
2			33.1±1.2	22.3±1.1	$9.50x10^{11}$		
3	IT	6	20.3±1.0	17.6±0.7	$4.47x10^{11}$	Vigene	
4			21.7±0.6	16.4±0.3	$1.48x10^{11}$		
5			22.2±0.6	17.2±0.2	$4.47x10^{10}$		

GLP = Good Laboratory Practice; IT = intrathecal; UNC-VC = University of North Carolina – Vector Core; vg = vector genome
*IT injections were via lumbar puncture, a 5 µL dose in vehicle (350mM phosphate-buffered saline, 5% sorbitol).
**Mean ± standard deviation

The non-GLP studies presented in Table 1 were designed to identify any long-term safety issues of the experimental therapy. The mice were randomized to different groups and injected IT with 5 µL of vehicle or different doses of AAV9/CLN7 vectors. Two lots of AAV9/CLN7 vectors were made by UNC-VC (University of North Carolina - Vector Core) or Vigene (Vigene Biosciences, Inc.). Both vectors were titered in parallel at UNC. Sodium dodecyl sulfate–polyacrylamide gel electrophoresis (SDS-PAGE) and silver stain analysis showed no visible contaminating protein in the UNC-VC lot, but the Vigene lot had host cell protein contamination (not shown). The equivalence of the two AAV9/CLN7 vector lots made at UNC-VC and Vigene was assessed by injecting the same doses of each vector in WT mice and measuring the biodistribution of the vectors to the heart, liver, and brain after 1 week. As shown in Figure 1, both vectors showed equivalent in vivo biodistribution patterns, indicating similar biopotency, and therefore were employed in this toxicity study.

Figure 1. In vivo equivalence of UNC and Vigene preclinical lots of AAV9/CLN7 vectors.

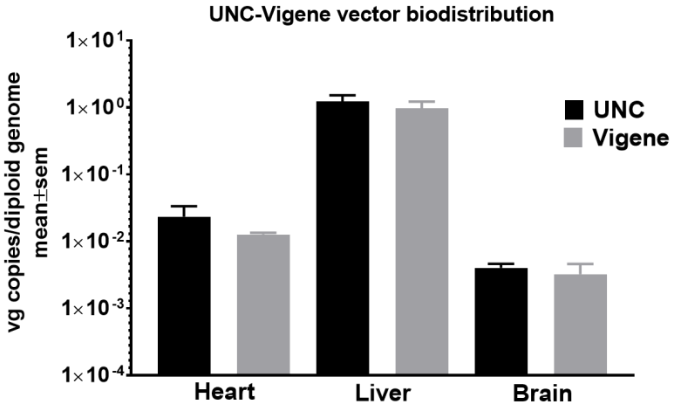

Mice (n=3) in each group were injected with the vector via tail vein in a 200 µL volume. The dose administered was 2×10^{11} vg/mouse and tissues were collected a week later for biodistribution analysis.

Mice were monitored for changes in body weight, clinical signs, adverse events, and mortality following the treatment. All mice were weighed twice weekly for the first 2 months and then biweekly thereafter. Any clinical signs or adverse events including neurological symptoms were investigated, evaluated, and recorded. Appropriate supportive or therapeutic interventions were offered per Institutional Animal Care and Use Committee (IACUC) and veterinary guidance. Blood and tissue samples were collected from mice that are euthanized for humane reasons. Where possible, a detailed necropsy was performed to investigate or identify the reason for the ailment by a trained technician or veterinary staff. Terminal tissue samples at 12 months following the treatment were collected for histopathological assessment. The final histopathological evaluation on collected tissue samples was performed and reported by Dr. Mary Wight-Carter, DVM, DACVP, Veterinary Pathologist at Animal Research Center, University of Texas Southwestern Medical Center.

RESULTS AND DISCUSSION

Body Weight

IT AAV9/CLN7 doses up to 9.50×10^{11} vg/mouse were administered in WT C57BL/6J mice to assess the safety of IT dosing and *hCLN7* overexpression (Table 1). The vector manufactured by UNC-VC (dosed 21 December 2017) and Vigene (dosed 19 or 23 October 2017) were tested in separate cohorts. Body weight difference was monitored to assess the overall health of the animals. There was no significant difference in body weight between groups within male or female mice at any point of assessment (Figure 2 and Table 3), demonstrating that doses up to 9.50×10^{11} vg/mouse are well tolerated in the WT C57BL/6J mice up to 12 months following the treatment.

Figure 2. No significant difference of body weight between groups within male or female mice.

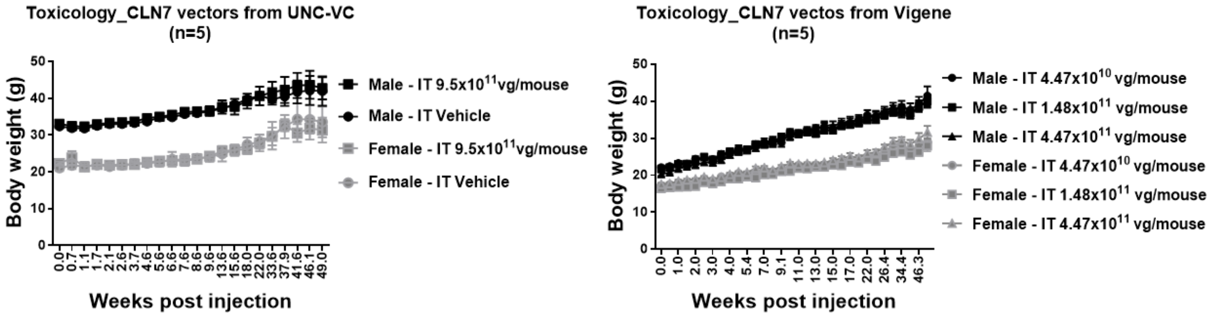

WT CH57BL/6J mice (n=5 per group/sex) were dosed with vehicle or AAV9/CLN7 via IT injection. Left panel: UNC manufactured AAV9/CLN7 was dosed at 9.50×10^{11} vg/mouse at 8 weeks of age. Right panel: Vigene manufactured AAV9/CLN7 was dosed at 4.47×10^{10}, 1.48×10^{11}, or 4.47×10^{11} vg/mouse at 6 weeks of age. Mice were weighed twice weekly for the first two months following the treatment and then biweekly thereafter.

Clinical Signs or Adverse Events

No outward signs of toxicity were noted over the duration of the study.

Survival Rate

There were no obvious clinical signs of morbidity in the adult WT mice dosed with AAV9/CLN7 at doses up to 9.50×10^{11} vg/mouse (Table 1). There were four unexpected death in this study: one found death in the control group injected with vehicle with no obvious reason, one found death in the treated group injected with 4.47×10^{11} vg/mouse with no obvious reason, one found death in the treated group injected with 9.50×10^{11} vg/mouse which was most likely caused by overgrooming-induced severe back injury, and one euthanized death for animal welfare in the treated group injected with 9.50×10^{11} vg/mouse because of overgrooming-induced severe back and leg injury. There was no significant difference of survival rates between groups within male or female mice (Figure 3), further demonstrating that doses up to 9.50×10^{11} vg/mouse are well tolerated in WT C57BL/6J mice up to 12 months following the treatment.

Figure 3. No significant difference of survival rate between groups within male or female mice.

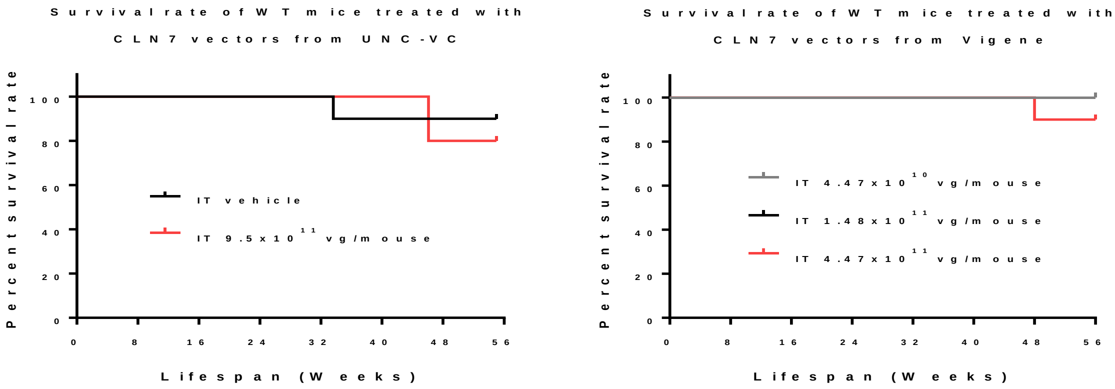

WT CH57BL/6J mice (n=5 per group/sex) were dosed with vehicle or AAV9/CLN7 via IT injection. Left panel: UNC manufactured AAV9/CLN7 was dosed at 9.50×10^{11} vg/mouse at 8 weeks of age. Right panel: Vigene manufactured AAV9/CLN7 was dosed at 4.47×10^{10}, 1.48×10^{11}, or 4.47×10^{11} vg/mouse at 6 weeks of age.

Histopathology

At the end of the experiment, 46 survival mice were perfused with phosphate-buffered saline containing 1 U/mL heparin and tissues were fixed in 10% formalin for 3 days. The tissues were then transferred to 70% ethanol and sent out for histopathology evaluation by Dr. Mary Wight-Carter, DVM, DACVP. Dr. Wight-Carter concluded that the tumors, increased number of inflammatory cell infiltrates, and degenerative lesions within multiple organs that were seen are considered to be common background lesions in aged mice. None of the microscopic findings are suggestive of adverse effects related to vector administration in these mice (see below the detailed report from Dr. Wight-Carter).

University of Texas Southwestern Medical Center
5323 Harry Hines Boulevard
Dallas, Texas 75390
Phone 214-648-1402
Mary.Wight-Carter@UTSouthwestern.edu

FINAL REPORT

Safety Study of scAAV9/CLN7 Vectors in WT C57BL/6J Mice

Report No. 18-088

**Sponsor Name:**	Mila's Miracle Foundation
**Study Director:**	Steven Gray, PhD
**Contract Pathologist:**	Mary Wight-Carter, DVM, DACVP
**Date of Final Report:**	April 15, 2019

[signature] 5-10-19

Mary Wight-Carter, DVM, DACVP — Date

Tissues were stored in 70% ETOH, trimmed into tissue cassettes and sent for processing to IDEXX laboratories. Hematoxylin and eosin stained slides were produced from the cassettes. Tissues and the corresponding slides were labeled with the following IDs (Table 2): 101, 102, 103, 104, 105, 106, 107, 108, 109, 110, 111, 112, 113, 114, 115, 117, 119, WT1, WT2, WT3, WT4, WT5, WT6, WT7, WT8, WT9, WT10, WT11, WT12, WT14, WT15, WT16, WT17, WT18, WT19, WT20, WT21, WT22, WT23, WT24, WT25, WT26, WT27, WT28, WT29, and WT30. Brain, heart, liver, lung, gonad, spleen, kidney, eyeball, sciatic nerve, cervical and lumbar spinal cord were submitted for all animals except for the following instances. The sciatic nerve was not present for animal ID 101, 103, 106, 108, WT1, WT 21, WT22, and WT 23. The gonad was not present for animal ID 110, 111, 114, WT1, WT2, and WT11. The brain was not present for animal ID WT28.

Table 2. Summary of mouse sex, group, and ID number

IT* vg/mouse	Male					Female				
vehicle	106	107	108	109	116	113	114	115	117	119
9.50×10^{11}	101	102	103	104	105	110	111	112	118	120
4.47×10^{11}	WT16	WT17	WT18	WT19	WT20	WT11	WT12	WT13	WT14	WT15
1.48×10^{11}	WT6	WT7	WT8	WT9	WT10	WT1	WT2	WT3	WT4	WT5
4.47×10^{10}	WT26	WT27	WT28	WT29	WT30	WT21	WT22	WT23	WT24	WT25

IT = intrathecal; vg = vector genome
*IT injections were via lumbar puncture, a 5 µL dose in vehicle (350 mM phosphate-buffered saline, 5% sorbitol).

The cerebrum, cerebellum, and olfactory bulb of all the mice were microscopically normal. There were no abnormalities found in the cervical or lumbar cord of the mice. The eyes that were submitted were all normal. There were no abnormalities found in the sciatic nerves in any of the mice.

The seminiferous tubules of animal ID WT27 and 109 had multiple variably sized vacuoles that replaced various levels of the seminiferous epithelium in a few tubules. There was no evidence of accompanying germ cell degeneration. Because very few tubules were affected and there was no accompanying degeneration, it suggests that this was an incidental finding. Animal ID 101 had multiple variably sized vacuoles that replaced various levels of the seminiferous epithelium in multiple tubules. Sertoli cells were present; however, other levels of germ cells were not. No other lesions were present in the mouse testicles.

All of the ovaries that were present were normal and the structures within the ovaries were consistent with various points in the estrus cycle.

The hearts were normal. There was no evidence of heart failure in the other organs.

The kidneys of 107 showed multifocal mild to moderate thickening of the glomerular tufts, multifocal tubular regeneration, mild multifocal interstitial fibrosis, and mild to moderate multifocal interstitial and perivascular infiltrates with mononuclear cells (glomerulonephropathy). The multiple glomerular tufts were thickened with eosinophilic proteinaceous material in kidneys of animal ID 105 (mild glomerulopathy).

There was mild to moderate perivascular infiltrates with small to moderate numbers of mononuclear cells in kidneys of WT9, WT17, WT18, WT15, WT4, and WT6. There were mild multifocal peripelvic infiltrates with small to moderate numbers of mononuclear cells in kidneys of WT1, WT4, WT5, WT6, WT7, WT8, WT9, WT11, WT12, WT14, WT16, WT17, WT19, WT20, WT21, WT22, WT23, WT24, WT25, WT26, WT27, WT28, WT29, WT30, 101, 102, 103, 104, 105, 106, 108, 109, 110, 111, 112, 114, 117, and 119.

The tubules of kidneys of WT8 had a few small areas of mineralization. The renal pelvis had mild to moderate dilation of a few tubules of kidneys of WT12, WT14, WT15, and WT23.

The above described lesions are not uncommon in adult or aged mice and typically are more frequent in male mice.

The livers of the following mice had mild to moderate peribiliary infiltrates with mononuclear cells with no corresponding fibrosis or hepatocellular necrosis: 107, WT1, WT5, WT15, WT17, and WT26. Minimal to moderate peribiliary and perivascular infiltrates are a common finding in mice and increase in incidence as the mice age.

The livers of the following mice contained multifocal infiltrates with small numbers of mixed inflammatory cell infiltrates with hepatocellular necrosis (micro-abscess): 102, 103, 104, 105, 106, 111, 112, 113, 114, WT1, WT4, WT12, WT14, WT15, WT17, and WT25. Areas with 1 to 2 cell hepatocyte necrosis accompanied by inflammatory cells can occur spontaneously in the mouse liver with increased incidence as the mice age.

Animal numbers 101 and 102 had multifocal areas of extramedullary hematopoiesis present in the liver parenchyma. This is less common in rodents as they age and typically occurs in response to increased hematopoietic demand.

Animal 105 had a liver nodule grossly evident that microscopically was morphologically consistent with a hepatocellular adenoma which expanded the parenchyma and compressed the adjacent normal tissue. Animal 106 had a hepatocellular adenoma which expanded the parenchyma and compressed the adjacent normal tissue. This tumor was not grossly evident. Adenomas are common findings in adult B6 mice.

Multifocal hepatocytes throughout the livers from 101, 102, 103, 104, 105, 106, 108, 109, 110, 113, 114, 117, 119, WT3, WT6, WT9, WT16, WT17, WT19, WT20, WT23, WT27, WT29, and WT30 had round, variably sized intracytoplasmic vacuoles that are morphologically consistent with lipidosis.

The lungs from the following mice had mild to moderate perivascular infiltrates with mononuclear cells: 101, 102, 103, 106, 107, WT1, WT10, WT15, WT18, WT20, WT21, and WT24. The lungs from the following mice had mild to moderate peribronchiolar infiltrates with mononuclear cells: 112, 117, WT17, WT18, WT23, WT25, WT26, WT28, WT29, WT3, WT8, and WT9. These infiltrates are commonly seen in the lungs of adult mice.

Mouse 102 had a focal area of mineralization surrounded by a small amount of fibrosis.

The spleen of all mice had variable amounts of extramedullary hematopoiesis and hemosiderin within the macrophages of the red pulp. This is considered normal in older mice. Mouse WT2 had enlarged spleen grossly and the white pulp was expanded with neoplastic small lymphocytes (lymphoma).

Table 3. Mean and individual body weight (g)

ID	*Sex*	*IT*	*Date*	*0 weeks*	*0.7 weeks*	*1.1 weeks*	*1.7 weeks*	*2.1 weeks*	*2.6 weeks*	*3.7 weeks*	*4.6 weeks*	*5.6 weeks*	*6.6 weeks*	*7.6 weeks*	*8.6 weeks*
106	M	Vehicle	12/21/2017	33.9	33.6	33.4	34.0	34.1	34.9	35.4	35.8	37.0	36.6	37.7	37.5
107	M	Vehicle	12/21/2017	31.0	30.9	310	31.4	31.7	31.6	32.5	33.7	34.2	35.2	35.9	35.8
108	M	Vehicle	12/21/2017	31.4	30.7	30.8	31.0	31.7	31.8	31.4	32.1	33.3	33.1	34.3	34.7
109	M	Vehicle	12/21/2017	32.5	31.7	30.8	32.1	33.0	32.7	32.6	32.5	33.8	34.2	34.3	35.9
116	M	Vehicle	12/21/2017	33.2	32.7	33.3	34.4	34.5	34.4	34.6	34.6	36.0	35.3	36.6	36.3
			**Mean**	**32.4**	**31.9**	**31.9**	**32.6**	**33.0**	**33.1**	**33.3**	**33.7**	**34.9**	**34.9**	**35.8**	**36.0**
113	F	Vehicle	12/21/2017	22.4	22.7	22.2	22.2	22.3	23.1	23.8	23.4	22.7	26.8	23.6	25.5
114	F	Vehicle	12/21/2017	20.8	20.9	20.8	22.2	21.8	21.4	22.4	22.6	22.8	23.3	25.6	23.2
115	F	Vehicle	12/21/2017	20.8	21.8	21.3	21.9	21.4	22.8	22.8	22.3	24.6	23.3	23.3	24.2
119	F	Vehicle	12/21/2017	21.4	23.0	23.4	22.8	21.2	20.6	20.8	21.9	22.0	22.0	21.6	23.2
117	F	Vehicle	12/21/2017	20.1	20.7	20.0	20.4	20.7	21.2	21.1	21.6	21.3	21.8	22.0	22.5
			**Mean**	**21.1**	**21.8**	**21.5**	**21.9**	**21.5**	**21.8**	**22.2**	**22.4**	**22.7**	**23.4**	**23.2**	**23.7**
101	M	$9.50x10^{11}$	12/21/2017	34.7	35.1	34.5	35.1	35.4	35.9	36.8	37.8	38.8	39.6	40.5	40.4
102	M	$9.50x10^{11}$	12/21/2017	33.5	32.7	32.3	33.2	33.1	33.2	33.4	34.1	33.9	35.3	35.0	35.5
103	M	$9.50x10^{11}$	12/21/2017	30.9	29.9	30.6	31.3	32.1	32.8	32.6	33.9	34.3	34.7	35.6	35.7
105	M	$9.50x10^{11}$	12/21/2017	33.3	32.7	32.8	32.8	33.6	32.6	32.6	33.4	33.7	33.0	33.9	34.1
104	M	$9.50x10^{11}$	12/21/2017	33.1	31.9	31.6	32.4	32.6	33.1	33.3	34.0	34.5	35.0	36.6	36.4
			**Mean**	**33.1**	**32.5**	**32.4**	**33.0**	**33.4**	**33.5**	**33.7**	**34.6**	**35.0**	**35.5**	**36.3**	**36.4**
117	F	$9.50x10^{11}$	12/21/2017	23.6	23.7	23.9	24.2	23.5	24.8	24.2	24.8	26.2	25.1	25.1	24.9
110	F	$9.50x10^{11}$	12/21/2017	22.5	22.4	21.4	23.4	23.2	22.4	23.1	23.8	24.1	24.1	24.8	25.2
118	F	$9.50x10^{11}$	12/21/2017	22.9	22.2	21.7	220	20.8	21.0	21.0	22.0	21.6	22.1	22.0	22.9
112	F	$9.50x10^{11}$	12/21/2017	20.3	19.9	19.3	19.9	20.4	20.2	20.8	20.9	20.7	21.4	21.3	21.8
120	F	$9.50x10^{11}$	12/21/2017	22.0	20.8	20.4	20.8	21.3	21.7	21.3	21.7	22.1	22.6	22.2	22.6
			**Mean**	**22.3**	**21.8**	**21.3**	**22.1**	**21.8**	**22.0**	**22.1**	**22.6**	**22.9**	**23.1**	**23.1**	**23.5**

ID	*Sex*	*IT*	*Date*	*9.6 weeks*	*13.6 weeks*	*15.6 weeks*	*18.0 weeks*	*22.0 weeks*	*33.6 weeks*	*37.9 weeks*	*41.6 weeks*	*46.1 weeks*	*48.9 weeks*	*49.0 weeks*
106	M	Vehicle	12/21/2017	38.3	39.3	40.4	42.3	43.1	42.3	43.4	44.2	43.9	41.1	39.7
107	M	Vehicle	12/21/2017	36.4	37.7	38.1	39.5	41.1	35.5	32.9	32.5	30.7	31.1	29.2
108	M	Vehicle	12/21/2017	34.9	35.7	37.3	39.2	39.7	40.6	42.9	15.9	48.6	49.2	47.1
109	M	Vehicle	12/21/2017	35.5	36.9	37.5	39.0	38.9	40.7	42.8	44.4	45.5	46.7	44.5
116	M	Vehicle	12/21/2017	37.0	37.0	38.6	38.8	41.1	Dead	Dead	Dead	Dead	Dead	Dead
			**Mean**	**36.4**	**37.3**	**38.4**	**39.8**	**10.8**	**39.8**	**40.5**	**41.8**	**42.2**	**42.0**	**40.1**
113	F	Vehicle	12/21/2017	26.1	28.0	26.0	29.8	30.4	35.6	36.2	37.9	39.3	39.4	37.6
114	F	Vehicle	12/21/2017	24.3	24.4	28.1	26.3	26.8	29.3	33.3	35.5	35.9	34.3	33.0
115	F	Vehicle	12/21/2017	24.2	26.0	25.7	28.7	30.2	31.0	33.7	38.5	35.2	34.4	33.5
119	F	Vehicle	12/21/2017	22.8	23.6	24.8	27.4	27.5	27.7	30.3	31.2	31.4	32.1	30.2
117	F	Vehicle	12/21/2017	23.0	22.7	25.2	24.9	26.3	25.7	28.1	28.6	29.6	27.9	26.6
			**Mean**	**24.1**	**24.9**	**26.0**	**27.4**	**28.2**	**29.9**	**32.3**	**34.3**	**34.3**	**33.6**	**32.2**
101	M	9.50×10^{11}	12/21/2017	41.0	43.9	45.4	45.9	49.2	50.6	52.9	53.5	54.0	50.3	49.4
102	M	9.50×10^{11}	12/21/2017	34.6	34.9	33.9	35.4	36.3	34.7	33.4	35.0	34.2	35.9	35.6
103	M	9.50×10^{11}	12/21/2017	36.4	37.8	37.3	40.3	41.3	42.8	43.6	45.2	47.0	44.1	44.2
105	M	9.50×10^{11}	12/21/2017	33.4	33.6	33.8	34.6	34.8	36.6	35.6	36.8	35.5	35.5	34.9
104	M	9.50×10^{11}	12/21/2017	37.5	38.2	38.2	40.1	41.7	43.8	46.0	47.1	47.8	47.6	46.8
			**Mean**	**36.6**	**37.7**	**37.7**	**39.3**	**40.7**	**41.7**	**42.3**	**43.5**	**43.7**	**42.7**	**42.2**
117	F	9.50×10^{11}	12/21/2017	26.3	29.2	30.1	30.8	33.4	36.5	34.0	27.4	Dead	Dead	Dead
110	F	9.50×10^{11}	12/21/2017	25.5	28.6	28.9	28.2	32.9	33.3	35.4	35.5	35.7	35.5	34.0
118	F	9.50×10^{11}	12/21/2017	23.4	24.5	23.2	23.7	23.3	23.9	24.8	23.8	Dead	Dead	Dead
112	F	9.50×10^{11}	12/21/2017	22.8	22.0	22.4	23.5	23.4	24.9	27.9	29.7	25.8	24.7	23.8
120	F	9.50×10^{11}	12/21/2017	23.4	24.0	25.1	25.0	26.2	29.6	33.6	36.5	34.1	34.0	33.3
			**Mean**	**24.3**	**25.7**	**25.9**	**26.2**	**27.8**	**29.6**	**31.1**	**30.6**	**31.9**	**31.4**	**30.4**

ID	*Sex*	*IT*	*Date*	*0 weeks*	*0.4 weeks*	*1.0 weeks*	*1.3 weeks*	*1.9 weeks*	*2.3 weeks*	*2.9 weeks*	*3.3 weeks*	*3.9 weeks*	*4.9 weeks*	*5.3 weeks*
WT16	M	1.48×10^{11}	10/23/2017	22.0	22.5	23.4	24.6	25.3	26.1	25.8	26.7	27.8	28.5	28.9

WT17	M	4.47x10^{11}	10/23/2017	18.9	19.8	21.2	21.4	22.2	23.2	23.7	24.5	26.3	27.5	27.5
WT18	M	4.47x10^{11}	10/23/2017	20.6	20.8	21.9	22.5	22.2	23.1	22.8	23.4	24.4	25.4	25.9
WT19	M	4.47x10^{11}	10/23/2017	19.7	19.9	20.4	20.8	20.8	21.4	21.1	21.7	22.9	24.6	24.2
WT20	M	4.47x10^{11}	10/23/2017	20.4	20.9	22.0	22.7	23.3	23.8	23.8	24.5	26.1	27.1	27.3
			**Mean**	**20.3**	**20.8**	**21.8**	**22.4**	**22.8**	**23.5**	**23.4**	**24.2**	**25.5**	**26.6**	**26.8**
WT11	F	4.47x10^{11}	10/23/2017	17.9	17.8	18.2	19.2	19.4	19.7	19.2	20.3	21.0	20.9	21.0
WT12	F	4.47x10^{11}	10/23/2017	16.6	16.8	17.2	18.4	18.2	18.7	18.2	18.8	19.5	20.4	20.4
WT13	F	4.47x10^{11}	10/23/2017	16.9	17.2	18.3	17.7	18.3	19.5	18.5	18.8	19.2	20.5	19.8
WT14	F	4.47x10^{11}	10/23/2017	18.3	17.7	18.5	19.1	19.1	19.2	19.6	19.4	20.4	21.0	21.0
WT15	F	4.47x10^{11}	10/23/2017	18.2	19.1	19.8	19.3	19.6	20.4	19.4	19.8	21.1	21.5	21.6
			**Mean**	**17.6**	**17.7**	**18.4**	**18.7**	**18.9**	**19.5**	**19.0**	**19.4**	**20.2**	**20.9**	**20.8**
WT6	M	1.48x10^{11}	10/23/2017	22.7	24.8	25.3	25.2	26.1	27.6	25.6	27.8	27.3	29.2	29.6
WT7	M	1.48x10^{11}	10/23/2017	21.8	22.1	23.7	21.5	24.8	25.9	26.5	26.5	28.8	27.5	28.3
WT8	M	1.48x10^{11}	10/23/2017	21.5	20.5	21.2	21.5	22.5	23.2	22.4	24.0	26.4	25.7	26.6
WT9	M	1.48x10^{11}	10/23/2017	21.7	22.2	23.2	23.2	23.5	24.8	23.9	24.6	25.6	25.5	25.6
WT10	M	1.48x10^{11}	10/23/2017	21.0	21.0	21.5	24.0	21.8	23.1	22.4	23.1	23.7	24.0	24.3
			**Mean**	**21.7**	**22.1**	**23.0**	**23.1**	**23.7**	**24.9**	**24.2**	**25.2**	**26.4**	**26.4**	**26.9**
WT1	F	1.48x10^{11}	10/23/2017	16.4	16.8	16.9	17.2	16.9	18.2	17.5	18.9	18.0	18.8	18.1
WT2	F	1.48x10^{11}	10/23/2017	16.6	17.0	17.1	17.0	17.7	18.4	17.5	18.7	19.3	18.2	19.8
WT3	F	1.48x10^{11}	10/23/2017	16.5	16.8	17.1	17.3	17.5	18.6	19.0	18.0	19.4	20.5	21.4
WT4	F	1.48x10^{11}	10/23/2017	15.9	15.9	16.3	15.8	16.0	17.8	17.4	17.3	18.6	18.4	19.1
WT5	F	1.48x10^{11}	10/23/2017	16.8	16.9	16.7	17.0	16.9	17.4	17.3	18.2	19.4	19.2	19.6
			**Mean**	**16.4**	**16.7**	**16.8**	**16.9**	**17.0**	**18.1**	**17.7**	**18.2**	**18.9**	**19.0**	**19.6**
WT26	M	4.47x10^{10}	10/24/2017	22.5	22.6	23.2	22.8	23.9	24.6	24.1	24.8	26.5	27.3	26.1

WT27	M	4.47x10^{10}	10/24/2017	23.2	23.8	25.1	24.9	26.9	27.5	26.4	28.3	28.6	30.1	29.8
WT28	M	4.47x10^{10}	10/24/2017	22.1	23.0	23.6	23.9	25.0	25.6	25.1	26.6	27.6	27.4	27.9
WT29	M	4.47x10^{10}	10/24/2017	21.4	21.4	21.8	21.4	22.7	22.7	22.3	24.1	24.7	24.6	24.7
WT30	M	4.47x10^{10}	10/24/2017	21.6	21.3	22.6	22.5	23.6	23.9	23.8	24.9	26.1	26.7	26.4
			**Mean**	**22.2**	**22.4**	**23.3**	**23.1**	**24.4**	**24.9**	**24.3**	**25.7**	**26.7**	**27.2**	**27.0**
WT21	F	4.47x10^{10}	10/24/2017	17.3	18.1	18.3	18.0	18.3	18.7	18.8	19.6	20.0	20.6	20.3
WT22	F	4.47x10^{10}	10/24/2017	17.1	17.3	18.1	17.6	17.6	19.1	17.8	18.7	18.8	19.8	19.7
WT23	F	4.47x10^{10}	10/24/2017	17.1	16.7	17.0	18.1	17.9	18.5	18.5	20.0	20.5	20.2	20.6
WT24	F	4.47x10^{10}	10/24/2017	17.5	17.8	18.6	18.1	18.9	19.4	19.2	19.7	20.3	21.2	20.8
WT25	F	4.47x10^{10}	10/24/2017	16.9	17.8	17.8	17.5	18.7	19.0	18.5	19.8	19.7	20.5	19.5
			**Mean**	**17.2**	**17.5**	**18.0**	**17.9**	**18.3**	**18.9**	**18.6**	**19.6**	**19.9**	**20.5**	**20.2**

ID	*Sex*	*IT*	*Date*	*5.9 weeks*	*6.9 weeks*	*7.9 weeks*	*9.0 weeks*	*10.0 weeks*	*10.9 weeks*	*12.0 weeks*	*12.9 weeks*	*13.9 weeks*	*14.9 weeks*	*15.9 weeks*
WT16	M	1.48x10^{11}	10/23/2017	29.3	30.9	31.2	32.6	33.0	33.1	33.4	34.0	34.9	34.9	35.8
WT17	M	4.47x10^{11}	10/23/2017	28.7	29.2	29.2	29.8	30.0	31.0	30.7	31.4	32.4	31.5	32.2
WT18	M	4.47x10^{11}	10/23/2017	26.1	26.9	27.2	27.7	28.3	29.4	29.8	30.2	31.0	31.3	31.5
WT19	M	4.47x10^{11}	10/23/2017	24.6	26.3	26.0	28.0	28.4	29.4	29.9	31.2	32.2	32.3	32.5
WT20	M	4.47x10^{11}	10/23/2017	28.2	29.0	29.3	31.2	33.0	33.2	35.0	34.5	37.0	37.2	38.2
			**Mean**	**27.4**	**28.5**	**28.6**	**29.9**	**30.5**	**31.2**	**31.8**	**32.3**	**33.5**	**33.4**	**34.0**
WT11	F	4.47x10^{11}	10/23/2017	22.8	23.3	21.4	22.8	23.4	22.8	23.0	24.3	23.7	25.3	25.6
WT12	F	4.47x10^{11}	10/23/2017	21.4	20.9	20.9	21.4	22.3	22.2	22.8	22.3	22.9	22.7	23.0
WT13	F	4.47x10^{11}	10/23/2017	20.6	21.0	20.7	22.4	22.7	23.1	22.8	22.7	23.8	23.2	23.5
WT14	F	4.47x10^{11}	10/23/2017	21.3	22.2	21.0	21.6	23.1	23.6	22.6	22.3	22.9	23.6	24.6

WT15	F	$4.47x10^{11}$	10/23/2017	23.3	22.8	22.8	23.6	24.6	23.2	24.5	23.8	23.9	25.4	26.1
			**Mean**	**21.9**	**22.0**	**21.4**	**22.4**	**23.2**	**23.0**	**23.1**	**23.1**	**23.4**	**24.0**	**24.6**
WT6	M	$1.48x10^{11}$	10/23/2017	30.4	29.9	30.6	31.5	32.6	32.6	33.8	32.7	33.8	33.6	35.6
WT7	M	$1.48x10^{11}$	10/23/2017	29.8	30.2	30.3	31.7	32.7	32.3	32.8	32.6	33.5	33.6	35.0
WT8	M	$1.48x10^{11}$	10/23/2017	28.1	28.2	29.2	30.6	31.2	31.1	31.6	30.8	32.4	31.9	32.9
WT9	M	$1.48x10^{11}$	10/23/2017	27.0	27.2	28.0	29.8	30.9	30.9	31.3	30.8	30.8	31.1	32.0
WT10	M	$1.48x10^{11}$	10/23/2017	26.1	26.0	27.0	28.3	29.1	29.0	29.0	29.3	30.2	30.4	31.2
			**Mean**	**28.3**	**28.3**	**29.0**	**30.4**	**31.3**	**31.2**	**31.7**	**31.2**	**32.1**	**32.1**	**33.3**
WT1	F	$1.48x10^{11}$	10/23/2017	18.2	19.5	19.3	20.0	20.4	21.3	21.3	21.4	21.5	22.1	21.6
WT2	F	$1.48x10^{11}$	10/23/2017	19.3	20.0	20.2	21.3	21.2	21.5	22.3	22.8	21.8	21.9	22.7
WT3	F	$1.48x10^{11}$	10/23/2017	20.2	21.0	21.6	21.9	21.9	23.6	22.0	22.6	23.4	24.6	23.6
WT4	F	$1.48x10^{11}$	10/23/2017	19.1	19.7	19.2	20.1	20.2	21.3	21.1	21.6	21.6	22.2	21.9
WT5	F	$1.48x10^{11}$	10/23/2017	19.7	20.6	20.8	21.7	21.9	21.8	22.0	22.7	22.7	22.8	23.3
			**Mean**	**19.3**	**20.2**	**20.2**	**21.0**	**21.1**	**21.9**	**21.7**	**22.2**	**22.2**	**22.7**	**22.6**
WT26	M	$4.47x10^{10}$	10/24/2017	27.6	28.0	28.3	29.2	30.0	28.7	29.8	30.6	31.0	28.8	30.6
WT27	M	$4.47x10^{10}$	10/24/2017	31.2	32.3	32.8	24.8	35.0	34.7	35.4	35.4	36.5	35.8	37.1
WT28	M	$4.47x10^{10}$	10/24/2017	29.4	30.3	30.7	31.5	32.1	31.8	32.4	33.8	34.1	33.5	33.7
WT29	M	$4.47x10^{10}$	10/24/2017	26.2	26.4	27.3	28.6	29.8	30.5	30.7	31.6	31.6	31.9	33.0
WT30	M	$4.47x10^{10}$	10/24/2017	27.4	27.6	28.1	29.9	31.1	30.8	32.2	32.4	32.8	32.3	34.0
			**Mean**	**28.4**	**28.9**	**29.4**	**28.8**	**31.6**	**31.3**	**32.1**	**32.8**	**33.2**	**32.5**	**33.7**
WT21	F	$4.47x10^{10}$	10/24/2017	21.1	22.0	20.9	21.8	22.0	22.7	22.4	22.4	22.5	23.2	23.3
WT22	F	$4.47x10^{10}$	10/24/2017	20.1	20.7	20.3	21.0	21.0	21.7	22.9	21.6	21.8	22.6	23.4
WT23	F	$4.47x10^{10}$	10/24/2017	21.4	21.3	20.7	22.1	22.5	23.2	23.1	24.2	24.0	24.6	25.0

WT24	F	4.47x10^{10}	10/24/2017	21.1	22.1	21.5	22.0	22.1	23.3	22.9	23.3	23.3	23.3	23.9
WT25	F	4.47x10^{10}	10/24/2017	20.4	21.4	21.4	21.7	21.8	22.5	22.0	22.3	22.7	22.9	23.0
			**Mean**	**20.8**	**21.5**	**21.0**	**21.7**	**21.9**	**22.7**	**22.7**	**22.8**	**22.9**	**23.3**	**23.7**

ID	*Sex*	*IT*	*Date*	*16.9 weeks*	*17.9 weeks*	*21.9 weeks*	*23.9 weeks*	*26.3 weeks*	*30.1 weeks*	*34.1 weeks*	*41.7 weeks*	*46.0 weeks*	*49.7 weeks*	*51.9 weeks*
WT16	M	1.48x10^{11}	10/23/2017	36.5	36.2	37.2	37.0	38.2	38.7	36.5	34.5	36.7	38.1	36.5
WT17	M	4.47x10^{11}	10/23/2017	32.7	32.4	32.9	33.4	33.6	34.8	32.8	33.6	34.4	34.2	32.9
WT18	M	4.47x10^{11}	10/23/2017	32.7	33.1	34.5	34.9	37.1	39.1	39.8	39.0	41.5	43.6	40.5
WT19	M	4.47x10^{11}	10/23/2017	33.2	33.5	34.7	36.0	36.8	38.8	40.0	38.4	39.9	42.7	40.5
WT20	M	4.47x10^{11}	10/23/2017	38.3	38.6	38.1	40.1	41.1	41.4	39.5	36.1	40.1	41.5	40.1
			**Mean**	**34.7**	**34.8**	**35.5**	**36.3**	**37.4**	**38.6**	**37.7**	**36.3**	**38.5**	**40.0**	**38.1**
WT11	F	4.47x10^{11}	10/23/2017	25.8	25.8	27.9	28.4	28.8	30.4	30.8	30.4	33.1	33.6	32.0
WT12	F	4.47x10^{11}	10/23/2017	22.6	23.2	23.8	23.8	24.8	25.3	27.0	25.0	26.2	27.5	25.2
WT13	F	4.47x10^{11}	10/23/2017	24.0	23.8	27.2	26.2	28.4	30.2	31.7	Dead	Dead	Dead	Dead
WT14	F	4.47x10^{11}	10/23/2017	26.5	24.1	24.4	25.1	28.5	28.2	27.6	29.1	28.4	30.0	29.2
WT15	F	4.47x10^{11}	10/23/2017	26.9	26.8	26.5	27.3	27.5	31.1	29.9	29.1	31.7	35.3	29.4
			**Mean**	**25.2**	**24.7**	**26.0**	**26.2**	**27.6**	**29.0**	**29.4**	**28.4**	**29.9**	**31.6**	**29.0**
WT6	M	1.48x10^{11}	10/23/2017	36.3	36.3	35.5	36.0	38.8	38.6	40.1	39.6	43.2	44.2	42.3
WT7	M	1.48x10^{11}	10/23/2017	34.2	34.9	36.1	36.3	37.2	37.2	37.5	36.4	37.3	40.0	37.7
WT8	M	1.48x10^{11}	10/23/2017	33.3	33.7	34.2	34.0	35.6	37.2	34.2	33.9	35.1	36.2	34.8
WT9	M	1.48x10^{11}	10/23/2017	33.2	33.5	35.8	35.8	37.8	38.1	39.2	39.8	41.5	43.4	40.9
WT10	M	1.48x10^{11}	10/23/2017	31.2	32.0	32.8	32.7	32.6	33.8	33.6	34.1	36.2	36.6	34.9
			**Mean**	**33.6**	**34.1**	**34.9**	**35.0**	**36.4**	**37.0**	**36.9**	**36.8**	**38.7**	**40.1**	**38.1**

WT1	F	$1.48x10^{11}$	10/23/2017	22.8	22.2	25.0	24.1	24.9	26.6	27.8	25.1	25.7	26.2	27.6
WT2	F	$1.48x10^{11}$	10/23/2017	23.7	23.3	23.3	23.7	24.7	25.8	28.2	26.0	27.2	28.9	28.6
WT3	F	$1.48x10^{11}$	10/23/2017	25.6	25.5	27.6	25.6	25.6	26.5	26.9	27.6	29.6	33.5	31.0
WT4	F	$1.48x10^{11}$	10/23/2017	22.1	22.2	22.5	21.9	24.6	26.0	23.8	24.2	24.0	25.5	24.4
WT5	F	$1.48x10^{11}$	10/23/2017	23.5	23.4	23.6	23.6	24.5	24.7	24.8	24.5	24.9	25.8	26.0
			**Mean**	**23.5**	**23.3**	**24.4**	**23.8**	**24.9**	**25.9**	**26.3**	**25.5**	**26.3**	**28.0**	**27.5**
WT26	M	$4.47x10^{10}$	10/24/2017	31.2	31.6	32.0	32.2	31.7	33.6	33.1	31.4	32.4	32.6	32.1
WT27	M	$4.47x10^{10}$	10/24/2017	38.2	39.1	39.5	38.6	40.0	40.7	41.3	38.5	40.4	43.3	41.6
WT28	M	$4.47x10^{10}$	10/24/2017	33.9	34.8	35.5	35.7	37.0	37.1	38.1	38.9	39.5	41.3	39.7
WT29	M	$4.47x10^{10}$	10/24/2017	33.2	34.0	35.8	35.1	36.7	37.5	37.4	38.1	39.7	41.7	40.4
WT30	M	$4.47x10^{10}$	10/24/2017	34.4	36.0	38.6	38.3	37.8	39.5	42.4	41.8	44.3	48.4	45.9
			**Mean**	**34.2**	**35.1**	**36.3**	**36.0**	**36.6**	**37.7**	**38.5**	**37.7**	**39.3**	**41.5**	**39.9**
WT21	F	$4.47x10^{10}$	10/24/2017	24.1	24.1	24.4	25.1	24.7	27.7	26.4	26.3	27.8	28.2	27.6
WT22	F	$4.47x10^{10}$	10/24/2017	23.4	22.8	23.2	25.1	23.6	26.0	25.3	25.5	26.3	26.3	26.3
WT23	F	$4.47x10^{10}$	10/24/2017	24.0	24.4	26.3	27.2	29.8	31.3	33.8	28.3	29.5	32.2	32.7
WT24	F	$4.47x10^{10}$	10/24/2017	24.5	24.6	25.9	25.6	27.6	28.2	28.1	26.8	28.3	29.0	28.1
WT25	F	$4.47x10^{10}$	10/24/2017	23.4	23.7	25.3	24.6	25.3	26.9	27.4	26.1	26.8	29.4	27.5
			**Mean**	**23.9**	**23.9**	**25.0**	**25.5**	**26.2**	**28.0**	**28.2**	**26.6**	**27.7**	**29.0**	**28.4**

IT = intrathecal dose received

Table 4. Histopathology of mice injected IT with vehicle

**Sex**	**M**	**M**	**M**	**M**	**M**	**F**	**F**	**F**	**F**	**F**
**ID**	**106**	**107**	**108**	**109**	**116[a]**	**113**	**114**	**115**	**117**	**119**
**IT dose (vg/mouse)**	**Vehicle**	**Vehicle**	**Vehicle**	**Vehicle**	**Vehicle**	**Vehicle**	**Vehicle**	**Vehicle**	**Vehicle**	**Vehicle**
Gross finding										
Cerebrum	Normal	Normal	Normal	Normal		Normal	Normal	Normal	Normal	Normal
Cerebellum	Normal	Normal	Normal	Normal		Normal	Normal	Normal	Normal	Normal
Olfactory bulb	Normal	Normal	Normal	Normal		Normal	Normal	Normal	Normal	Normal
Cervical cord	Normal	Normal	Normal	Normal		Normal	Normal	Normal	Normal	Normal
Lumber cord	Normal	Normal	Normal	Normal		Normal	Normal	Normal	Normal	Normal
Sciatic nerve	No data	Normal	No data	Normal		Normal	Normal	Normal	Normal	Normal
Eye	Normal	Normal	Normal	Normal		Normal	Normal	Normal	Normal	Normal
Ovaries						Normal	No data	Normal	Normal	Normal
Testicles: multiple vacuoles, an incidental finding				x						
Heart	Normal	Normal	Normal	Normal		Normal	Normal	Normal	Normal	Normal
Kidney: glomerulonephropathy, not uncommon in adult or aged mice		x								
Kidney: mild glomerulopathy, not uncommon in adult or aged mice										
Kidney: perivascular infiltrates with mononuclear cells, not uncommon in adult or aged mice										
Kidney: peripelvic infiltrates with mononuclear cells, not uncommon in adult or aged mice	x		x	x			x		x	x
Kidney: mineralization in the tubules, not uncommon in adult or aged mice										
Kidney: dilation of the tubules, not uncommon in adult or aged mice										
Liver: peribiliary infiltrates with mononuclear cells, a common finding in mice		x								
Liver: micro-abscess, an increased incidence as the mice age	x					x	x			
Liver: extramedullary hematopoiesis, in response to increased hematopoietic demand										
Liver: hepatocellular adenoma, a common finding in adult B6 mice	x									

**Sex**	**M**	**M**	**M**	**M**	**M**	**F**	**F**	**F**	**F**	**F**
**ID**	**106**	**107**	**108**	**109**	**116[a]**	**113**	**114**	**115**	**117**	**119**
**IT dose (vg/mouse)**	**Vehicle**	**Vehicle**	**Vehicle**	**Vehicle**	**Vehicle**	**Vehicle**	**Vehicle**	**Vehicle**	**Vehicle**	**Vehicle**
Liver: lipidosis	x		x	x		x	x		x	x
Lung: perivascular infiltrates with mononuclear cells, commonly seen in adult mice	x	x								
Lung: peribronchiolar infiltrates with mononuclear cells, commonly seen in adult mice									x	
Lung: focal mineralization										
Spleen	Normal	Normal	Normal	Normal		Normal	Normal	Normal	Normal	Normal

IT = intrathecal

[a] Mouse ID 116 was found dead at the age of 42 weeks with no obvious reason.

Table 5. Histopathology of mice injected IT with 9.50x10^{11} vg/mouse of AAV9/CLN7 vectors

**Sex**	**M**	**M**	**M**	**M**	**M**	**F**	**F**	**F**	**F**	**F**
**ID**	**101**	**102**	**103**	**104**	**105**	**110**	**111**	**112**	**118[a]**	**120[b]**
**IT dose (vg/mouse)**	**9.50x10^{11}**	**9.50x10^{11}**	**9.50x10^{11}**	**9.50x10^{11}**	**9.50x10^{11}**	**9.50x10^{11}**	**9.50x10^{11}**	**9.50x10^{11}**	**9.50x10^{11}**	**9.50x10^{11}**
Gross finding		Liver nodule			Liver nodule					
Cerebrum	Normal	Normal	Normal	Normal	Normal	Normal	Normal	Normal		
Cerebellum	Normal	Normal	Normal	Normal	Normal	Normal	Normal	Normal		
Olfactory bulb	Normal	Normal	Normal	Normal	Normal	Normal	Normal	Normal		
Cervical cord	Normal	Normal	Normal	Normal	Normal	Normal	Normal	Normal		
Lumber cord	Normal	Normal	Normal	Normal	Normal	Normal	Normal	Normal		
Sciatic nerve	No data	Normal	No data	Normal	Normal	Normal	Normal	Normal		
Eye	Normal	Normal	Normal	Normal	Normal	Normal	Normal	Normal		
Ovaries						No data	No data	Normal		
Testicles: multiple vacuoles, an incidental finding	x									
Heart	Normal	Normal	Normal	Normal	Normal	Normal	Normal	Normal		
Kidney: glomerulonephropathy, not uncommon in adult or aged mice										
Kidney: mild glomerulopathy, not uncommon in adult or aged mice					x					
Kidney: perivascular infiltrates with mononuclear cells, not uncommon in adult or aged mice										
Kidney: peripelvic infiltrates with mononuclear cells, not uncommon in adult or aged mice	x	x	x	x	x	x	x	x		
Kidney: mineralization in the tubules, not uncommon in adult or aged mice										
Kidney: dilation of the tubules, not uncommon in adult or aged mice										
Liver: peribiliary infiltrates with mononuclear cells, a common finding in mice										
Liver: micro-abscess, an increased incidence as the mice age		x	x	x	x		x	x		

Sex	M	M	M	M	M	F	F	F	F	F
**ID**	**101**	**102**	**103**	**104**	**105**	**110**	**111**	**112**	**118[a]**	**120[b]**
**IT dose (vg/mouse)**	**$9.50x10^{11}$**	**$9.50x10^{11}$**	**$9.50x10^{11}$**	**$9.50x10^{11}$**	**$9.50x10^{11}$**	**$9.50x10^{11}$**	**$9.50x10^{11}$**	**$9.50x10^{11}$**	**$9.50x10^{11}$**	**$9.50x10^{11}$**
Liver: extramedullary hematopoiesis, in response to increased hematopoietic demand	x	x								
Liver: hepatocellular adenoma, a common finding in adult B6 mice					x					
Liver: lipidosis	x	x	x	x	x	x				
Lung: perivascular infiltrates with mononuclear cells, commonly seen in adult mice	x	x	x							
Lung: peribronchiolar infiltrates with mononuclear cells, commonly seen in adult mice								x		
Lung: focal mineralization		x								
Spleen	Normal	Normal	Normal	Normal	Normal	Normal	Normal	Normal		

IT = intrathecal

[a] Mouse ID 118 was euthanized at the age of 57 weeks due to severe back wound caused by overgrooming.

[b] Mouse ID 120 was found dead at the age of 51 weeks due to severe back and leg wound caused by overgrooming.

Table 6. Histopathology of mice injected IT with $4.47x10^{11}$ vg/mouse of AAV9/CLN7 vectors

**Sex**	**M**	**M**	**M**	**M**	**M**	**F**	**F**	**F**	**F**	**F**
**ID**	**WT16**	**WT17**	**WT18**	**WT19**	**WT20**	**WT11**	**WT12**	**WT13[a]**	**WT14**	**WT15**
**IT dose (vg/mouse)**	**$4.47x10^{11}$**	**$4.47x10^{11}$**	**$4.47x10^{11}$**	**$4.47x10^{11}$**	**$4.47x10^{11}$**	**$4.47x10^{11}$**	**$4.47x10^{11}$**	**$4.47x10^{11}$**	**$4.47x10^{11}$**	**$4.47x10^{11}$**
Gross finding										
Cerebrum	Normal	Normal	Normal	Normal	Normal	Normal	Normal		Normal	Normal
Cerebellum	Normal	Normal	Normal	Normal	Normal	Normal	Normal		Normal	Normal
Olfactory bulb	Normal	Normal	Normal	Normal	Normal	Normal	Normal		Normal	Normal
Cervical cord	Normal	Normal	Normal	Normal	Normal	Normal	Normal		Normal	Normal
Lumber cord	Normal	Normal	Normal	Normal	Normal	Normal	Normal		Normal	Normal
Sciatic nerve	Normal	Normal	Normal	Normal	Normal	Normal	Normal		Normal	Normal
Eye	Normal	Normal	Normal	Normal	Normal	Normal	Normal		Normal	Normal
Ovaries						No data	Normal		Normal	Normal
Testicles: multiple vacuoles, an incidental finding										
Heart	Normal	Normal	Normal	Normal	Normal	Normal	Normal		Normal	Normal
Kidney: glomerulonephropathy, not uncommon in adult or aged mice										
Kidney: mild glomerulopathy, not uncommon in adult or aged mice										
Kidney: perivascular infiltrates with mononuclear cells, not uncommon in adult or aged mice		x	x							x
Kidney: peripelvic infiltrates with mononuclear cells, not uncommon in adult or aged mice	x	x		x	x	x	x		x	
Kidney: mineralization in the tubules, not uncommon in adult or aged mice										
Kidney: dilation of the tubules, not uncommon in adult or aged mice							x		x	x
Liver: peribiliary infiltrates with mononuclear cells, a common finding in mice		x								x
Liver: micro-abscess, an increased incidence as the mice age		x					x		x	x

**Sex**	**M**	**M**	**M**	**M**	**M**	**F**	**F**	**F**	**F**	**F**
**ID**	**WT16**	**WT17**	**WT18**	**WT19**	**WT20**	**WT11**	**WT12**	**WT13**[a]	**WT14**	**WT15**
**IT dose (vg/mouse)**	**$4.47x10^{11}$**	**$4.47x10^{11}$**	**$4.47x10^{11}$**	**$4.47x10^{11}$**	**$4.47x10^{11}$**	**$4.47x10^{11}$**	**$4.47x10^{11}$**	**$4.47x10^{11}$**	**$4.47x10^{11}$**	**$4.47x10^{11}$**
Liver: extramedullary hematopoiesis, in response to increased hematopoietic demand										
Liver: hepatocellular adenoma, a common finding in adult B6 mice										
Liver: lipidosis	x	x		x	x					
Lung: perivascular infiltrates with mononuclear cells, commonly seen in adult mice			x		x					x
Lung: peribronchiolar infiltrates with mononuclear cells, commonly seen in adult mice		x	x							
Lung: focal mineralization										
Spleen	Normal	Normal	Normal	Normal	Normal	Normal	Normal		Normal	Normal

IT = intrathecal

[a] Mouse ID WT13 was found dead at the age of 46 weeks with no obvious reason.

Table 7. Histopathology of mice injected IT with $1.48x10^{11}$ vg/mouse of AAV9/CLN7 vectors

**Sex**	**M**	**M**	**M**	**M**	**M**	**F**	**F**	**F**	**F**	**F**
**ID**	**WT6**	**WT7**	**WT8**	**WT9**	**WT10**	**WT1**	**WT2**	**WT3**	**WT4**	**WT5**
**IT dose (vg/mouse)**	**$1.48x10^{11}$**	**$1.48x10^{11}$**	**$1.48x10^{11}$**	**$1.48x10^{11}$**	**$1.48x10^{11}$**	**$1.48x10^{11}$**	**$1.48x10^{11}$**	**$1.48x10^{11}$**	**$1.48x10^{11}$**	**$1.48x10^{11}$**
Gross finding							splenomegaly			
Cerebrum	Normal	Normal	Normal	Normal	Normal	Normal	Normal	Normal	Normal	Normal
Cerebellum	Normal	Normal	Normal	Normal	Normal	Normal	Normal	Normal	Normal	Normal
Olfactory bulb	Normal	Normal	Normal	Normal	Normal	Normal	Normal	Normal	Normal	Normal
Cervical cord	Normal	Normal	Normal	Normal	Normal	Normal	Normal	Normal	Normal	Normal
Lumber cord	Normal	Normal	Normal	Normal	Normal	Normal	Normal	Normal	Normal	Normal
Sciatic nerve	Normal	Normal	Normal	Normal	Normal	No data	Normal	Normal	Normal	Normal
Eye	Normal	Normal	Normal	Normal	Normal	Normal	Normal	Normal	Normal	Normal
Ovaries						No data	No data	Normal	Normal	Normal
Testicles: multiple vacuoles, an incidental finding										
Heart	Normal	Normal	Normal	Normal	Normal	Normal	Normal	Normal	Normal	Normal
Kidney: glomerulonephropathy, not uncommon in adult or aged mice										
Kidney: mild glomerulopathy, not uncommon in adult or aged mice										
Kidney: perivascular infiltrates with mononuclear cells, not uncommon in adult or aged mice	x			x					x	
Kidney: peripelvic infiltrates with mononuclear cells, not uncommon in adult or aged mice	x	x	x	x		x			x	x
Kidney: mineralization in the tubules, not uncommon in adult or aged mice			x							
Kidney: dilation of the tubules, not uncommon in adult or aged mice										
Liver: peribiliary infiltrates with mononuclear cells, a common finding in mice						x				x
Liver: micro-abscess, an increased incidence as the mice age						x			x	

**Sex**	**M**	**M**	**M**	**M**	**M**	**F**	**F**	**F**	**F**	**F**
**ID**	**WT6**	**WT7**	**WT8**	**WT9**	**WT10**	**WT1**	**WT2**	**WT3**	**WT4**	**WT5**
**IT dose (vg/mouse)**	**$1.48x10^{11}$**	**$1.48x10^{11}$**	**$1.48x10^{11}$**	**$1.48x10^{11}$**	**$1.48x10^{11}$**	**$1.48x10^{11}$**	**$1.48x10^{11}$**	**$1.48x10^{11}$**	**$1.48x10^{11}$**	**$1.48x10^{11}$**
Liver: extramedullary hematopoiesis, in response to increased hematopoietic demand										
Liver: hepatocellular adenoma, a common finding in adult B6 mice										
Liver: lipidosis	x			x				x		
Lung: perivascular infiltrates with mononuclear cells, commonly seen in adult mice					x	x				
Lung: peribronchiolar infiltrates with mononuclear cells, commonly seen in adult mice			x	x				x		
Lung: focal mineralization										
Spleen	Normal	Normal	Normal	Normal	Normal	Normal	Normal	Normal	Normal	Normal

IT = intrathecal

Table 8. Histopathology of mice injected IT with 4.47×10^{10} vg/mouse of AAV9/CLN7 vectors

**Sex**	**M**	**M**	**M**	**M**	**M**	**F**	**F**	**F**	**F**	**F**
**ID**	**WT26**	**WT27**	**WT28**	**WT29**	**WT30**	**WT21**	**WT22**	**WT23**	**WT24**	**WT25**
**IT dose (vg/mouse)**	**4.47×10^{10}**	**4.47×10^{10}**	**4.47×10^{10}**	**4.47×10^{10}**	**4.47×10^{10}**	**4.47×10^{10}**	**4.47×10^{10}**	**4.47×10^{10}**	**4.47×10^{10}**	**4.47×10^{10}**
Gross finding										
Cerebrum	Normal	Normal	No data	Normal	Normal	Normal	Normal	Normal	Normal	Normal
Cerebellum	Normal	Normal	No data	Normal	Normal	Normal	Normal	Normal	Normal	Normal
Olfactory bulb	Normal	Normal	No data	Normal	Normal	Normal	Normal	Normal	Normal	Normal
Cervical cord	Normal	Normal	Normal	Normal	Normal	Normal	Normal	Normal	Normal	Normal
Lumber cord	Normal	Normal	Normal	Normal	Normal	Normal	Normal	Normal	Normal	Normal
Sciatic nerve	Normal	Normal	Normal	Normal	Normal	No data	No data	No data	Normal	Normal
Eye	Normal	Normal	Normal	Normal	Normal	Normal	Normal	Normal	Normal	Normal
Ovaries						Normal	Normal	Normal	Normal	Normal
Testicles: multiple vacuoles, an incidental finding		x								
Heart	Normal	Normal	Normal	Normal	Normal	Normal	Normal	Normal	Normal	Normal
Kidney: glomerulonephropathy, not uncommon in adult or aged mice										
Kidney: mild glomerulopathy, not uncommon in adult or aged mice										
Kidney: perivascular infiltrates with mononuclear cells, not uncommon in adult or aged mice										
Kidney: peripelvic infiltrates with mononuclear cells, not uncommon in adult or aged mice	x	x	x	x	x	x	x	x	x	x
Kidney: mineralization in the tubules, not uncommon in adult or aged mice										
Kidney: dilation of the tubules, not uncommon in adult or aged mice								x		
Liver: peribiliary infiltrates with mononuclear cells, a common finding in mice	x									
Liver: micro-abscess, an increased incidence as the mice age										x

**Sex**	**M**	**M**	**M**	**M**	**M**	**F**	**F**	**F**	**F**	**F**
**ID**	**WT26**	**WT27**	**WT28**	**WT29**	**WT30**	**WT21**	**WT22**	**WT23**	**WT24**	**WT25**
**IT dose (vg/mouse)**	**$4.47x10^{10}$**	**$4.47x10^{10}$**	**$4.47x10^{10}$**	**$4.47x10^{10}$**	**$4.47x10^{10}$**	**$4.47x10^{10}$**	**$4.47x10^{10}$**	**$4.47x10^{10}$**	**$4.47x10^{10}$**	**$4.47x10^{10}$**
Liver: extramedullary hematopoiesis, in response to increased hematopoietic demand										
Liver: hepatocellular adenoma, a common finding in adult B6 mice										
Liver: lipidosis		x		x	x			x		
Lung: perivascular infiltrates with mononuclear cells, commonly seen in adult mice						x			x	
Lung: peribronchiolar infiltrates with mononuclear cells, commonly seen in adult mice	x		x	x				x		x
Lung: focal mineralization										
Spleen	Normal	Normal	Normal	Normal	Normal	Normal	Normal	Normal	Normal	Normal

IT = intrathecal

CONCLUSIONS

IT AAV9/CLN7 doses up to 9.50×10^{11} vg/mouse are safe and well tolerated in WT mice. There were no toxicities observed in either the in-life portion of the study or after microscopic examination of major tissues. If scaled to humans by cerebrospinal fluid (CSF) volume (assuming 35 uL CSF volume in mice and 140 mL CSF volume in humans), the mouse dose of 9.5×10^{11} vg in 5 uL would translate to a human dose of 3.8×10^{15} vg in a volume of 20 mL. This highest dose injected in the mice is a 3.8-fold higher titer than the highest dose proposed in humans, in twice the volume proposed in humans. Thus, the maximum tolerated dose in mice up to one year post-injection provides a further safety margin above what is proposed in humans.

APPENDIX

UNC-VC Vector Certificate of Analysis

UNIVERSITY OF NORTH CAROLINA
VECTOR CORE

Quality Control Summary

Lot #	LAV45-final

Test by qPCR

Test #	Titer, vg/mL	Analyst	Date	File
1	1.87E+14	PZ	11/01/2017	20171101-1531-ghbh-pz

PAGE analysis

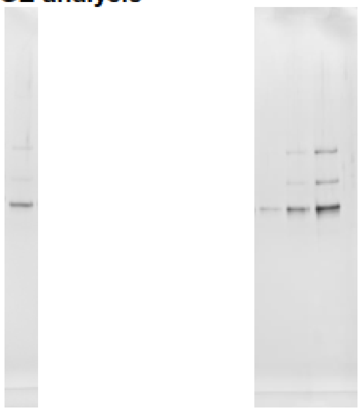

Loaded 5.00E+09 vg   4980E std   2e9vg   5e9vg   1e10vg

Calculated 4.00E+09 vg

Analyst	Ping Zhang
Date	11/03/2017
Reference #	20171103-silver

UNIVERSITY OF NORTH CAROLINA VECTOR CORE

SEM (before concentration)

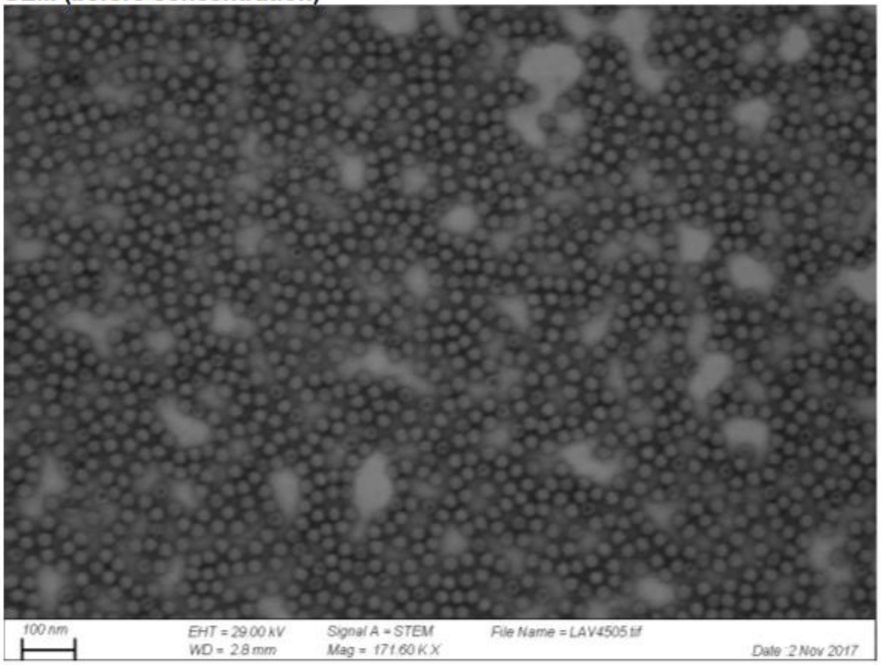

85% full

Analyst	Ping Zhang
Date	11/02/2017
Reference #	20171102-lav45-05

Vigene Vector Certificate of Analysis

12111 Parklawn Drive
Rockville, MD 20852
(301)251-6638
www.vigenebio.com

Vigene Biosciences
Excellence in Gene Delivery

PRODUCT INFORMATION & CERTIFICATE OF ANALYSIS

PRODUCT INFORMATION

Research Grade scAAV9-CLN7 virus production and purification

Date: September 13th 2017

SHELF LIFE: 2 years from date of receipt under proper storage conditions

SHIPMENT SPECIFICATION & HANDLING INSTRUCTION

Quantity	Description	Volume/titer
**1**	**AAV9/CLN7**	**4X 250µl, 9.80 1E13 gc/ml**

FORMULATION BUFFER

PBS, 5% Sorbitol, pH 7.4 containing 0.001%F-68

SHIPPING&STORAGE CONDITIONS

Product shipped on dry ice. Upon receiving, please store at -80 degrees for long-term storage.

HANDLING INSTRUCTION & PRODUCT MANUAL

For detailed information regarding the vectors and product manual for the corresponding product, please visit our website at http://www.vigenebio.com.
