# Supplementary material for: AAV9/MFSD8 gene therapy is effective in preclinical models of neuronal ceroid lipofuscinosis type 7 disease
Source: J Clin Invest. 2022 Mar 1;132(5):e146286. doi: 10.1172/JCI146286 (PMC8884910; doi:10.1172/JCI146286)
Supplement: Supplemental Toxicology Report 2 [file jci-132-146286-s026.pdf]

FINAL REPORT

Testing Facility Study No. 2759-001

Sponsor Reference No. CLN7-001

A Single Dose Toxicity Study of scAAV9/JeT-hCLN7opt-SV40pA Administered by Intrathecal Injection in Rats

SPONSOR:
Foundation for Batten Hope
3963 Maple Avenue, Suite 390
Dallas, TX 75219
USA

TESTING FACILITY:
Charles River Laboratories, Inc.
54943 North Main Street
Mattawan, MI 49071
USA

TABLE OF CONTENTS

Page

TABLE OF CONTENTS 2
REPORT TEXT 4
COMPLIANCE STATEMENT AND REPORT APPROVAL 4
QUALITY ASSURANCE STATEMENT 5
1. RESPONSIBLE PERSONNEL 7
1.1. Testing Facility 7
1.2. Individual Scientists (IS) at Testing Facility 7
1.3. Principal Investigators (PI) 7
2. SUMMARY 8
3. INTRODUCTION 9
3.1. Study Schedule 9
4. MATERIALS AND METHODS 9
4.1. Test Article and Vehicle Information 9
4.2. Test Article and Vehicle Characterization 10
4.3. Reserve Samples 10
4.4. Test Article Inventory and Disposition 10
4.5. Dose Formulation and Analysis 10
4.6. Test System 11
4.7. Experimental Design 12
4.8. In-life Procedures, Observations, and Measurements 13
4.9. Laboratory Evaluations 14
4.10. Terminal Procedures 15
5. STATISTICS 16
5.1. Group Pair-wise Comparisons (General ANOVA) 17
6. COMPUTER SYSTEMS 18
7. RETENTION OF RECORDS, SAMPLES, AND SPECIMENS 18
8. RESULTS 19
8.1. Mortality 19
8.2. In-life Examinations 19
8.3. Clinical Pathology 20
8.4. Terminal Evaluations 22
8.5. Biodistribution Evaluations 27
9. CONCLUSION 28
10. REFERENCES 29
FIGURE 30

TABLE OF CONTENTS

	Page
1. Mean Body Weight Values	30
2. Mean Body Weight Change Values	33
3. Mean Food Consumption Values	36
TABLE	39
1. Summary of Detailed Clinical Observations	39
2. Summary of Body Weight Values	44
3. Summary of Body Weight Change Values	53
4. Summary of Caged Food Consumption Values	62
5. Summary of Rotating Rod Values	69
APPENDIX	72
1. Amended Protocol, Protocol, and Deviations	72
2. Test Article Characterization	131
3. Record of Animal Fate and Disposition	133
4. Individual Detailed Clinical Observations	142
5. Individual Body Weight Values	153
6. Individual Body Weight Change Values	170
7. Individual Caged Food Consumption Values	187
8. Individual Rotating Rod Values	213
9. Clinical Pathology Report	222
10. Pathology Report	340
11. Biodistribution Analysis Report	698

COMPLIANCE STATEMENT AND REPORT APPROVAL

The study was conducted in accordance with the United States (US) Food and Drug Administration (FDA) Good Laboratory Practice (GLP) Regulations, 21 Code of Federal Regulations (CFR) Part 58. Exceptions from the above regulations are listed below.

- The Sponsor has not yet provided documentation of the strength, purity, composition, stability, and other pertinent information for the batch (lot) of vehicle and test article used on study.
- The Fusion v6 Rotating Rod System data was generated by computer software that is not validated.
- The samples for bioanalysis (plasma), CSF, and gene expression (tissues) were not analyzed. If analyzed, the data will be reported separately.

This study was conducted in accordance with the procedures described herein. All deviations authorized/acknowledged by the Study Director are documented in the Study Records. The report represents an accurate and complete record of the results obtained.

DocuSigned by:
Sarah Davis
Signer Name: Sarah Davis
Signing Reason: I approve this document
Signing Time: 30-Mar-2020 | 12:42:14 EDT
CBB533787C4D4D21A7693D214BF98FA5

Sarah Davis, BS, LATG
Study Director

QUALITY ASSURANCE STATEMENT

Below are the inspections conducted by the Quality Assurance Unit and the dates the inspections were reported to the Study Director and Testing Facility Management for Study No. 2759-001.

Date(s) of Inspection	Study Phase Inspected	Date(s) Reported to Study Director and Testing Facility Management
09/09/2018 to 09/10/2018	Protocol	09/10/2018, 09/10/2018
09/14/2018 to 09/14/2018	Test Material Preparation In-process Inspection	09/14/2018, 09/14/2018
09/18/2018 to 09/19/2018	Test Material Administration In-process Inspection	09/19/2018, 09/19/2018
10/22/2018 to 10/22/2018	Protocol Amendment 1	10/24/2018, 01/02/2019
12/12/2018 to 12/13/2018	Protocol Amendment 2	12/13/2018, 12/13/2018
12/12/2018 to 12/14/2018	Fusion Rotating Rod System In-process Inspection	12/14/2018, 01/02/2019
01/28/2019 to 01/28/2019	Postmortem Data Review	01/28/2019, 01/28/2019
01/28/2019 to 01/28/2019	Report Tables	01/28/2019, 02/05/2019
02/06/2019 to 02/12/2019	Postmortem Data Review	02/12/2019, 03/12/2019
02/06/2019 to 02/12/2019	Antemortem Data Review	02/12/2019, 02/26/2019
02/06/2019 to 02/12/2019	Antemortem Report Audit	02/12/2019, 02/26/2019
03/12/2019 to 03/12/2019	Clinical Pathology Report Review	03/12/2019, 03/12/2019
03/12/2019 to 03/22/2019	Postmortem Data Review	03/22/2019, 03/25/2019
04/15/2019 to 04/15/2019	Protocol Amendment 3	04/15/2019, 04/15/2019
02/25/2020 to 02/27/2020	Protocol Amendment 4	02/27/2020, 02/27/2020
03/17/2020 to 03/18/2020	Final Report Review	03/18/2020, 03/27/2020

In addition to the above-mentioned audits, process-based and/or routine facility inspections were also conducted during the course of this study. Inspection findings, if any, specific to this study were reported by Quality Assurance to the Study Director and Management.

DocuSigned by:
Shannon Dodes
Signer Name: Shannon Dodes
Signing Reason: I approve this document
Signing Time: 30-Mar-2020 | 06:51:05 EDT
B74EB5E454BA49C2A573D32C16FD2555

Shannon Dodes, BA, LAT, RQAP-GLP
Quality Assurance Auditor

1. RESPONSIBLE PERSONNEL

1.1. Testing Facility

**Senior Director, Safety Evaluation**	Theodore Baird, PhD, DSP
**Senior Director, Bioanalytical/Analytical Sciences**	Amy Smith, BA
**Senior Director, DMPK**	Clint Rosenfeld, PhD
**Director, Operations**	Kurt Willmont, BS, LAT
**Associate Director, Regulatory Compliance**	Janis Kissel, BS, RQAP-GLP
**Study Director**	Sarah Davis, BS, LATG
**Supervisor, Surgery and Efficacy**	
**Supervisor, Toxicology Services**	Jessica Haynes, BS, LAT
**Executive Director, Attending Veterinarian**	Dale Cooper, DVM, MS, DACLAM
**Report Coordinator**	Michael Westover, BAA, MBA
**Quality Assurance Auditor**	Shannon Dodes, BA, LAT, RQAP-GLP

1.2. Individual Scientists (IS) at Testing Facility

**Staff Veterinarians**	
	Scott Adrian, DVM
	Michael Bradley, BS, DVM
	April George, DVM
	Jennifer Kylie, BScH, BVM&S, DVS, DACLAMc
	Jesse Veenstra, DVM
**Individual Scientist, Clinical Pathology**	Caitlyn Carter, DVM, DACVP

1.3. Principal Investigators (PI)

**Serum, CSF, qPCR, and RT-qPCR Evaluations**	Steven J Gray, PhD<br>University of Texas Southwestern Medical Center-Gray Lab<br>6000 Harry Hines BLVD<br>NA02.508<br>Dallas, TX 75390

Anatomic Pathology

Odete R. Mendes, DVM, PhD, DACVP, DABT
Charles River Laboratories, Inc.
4025 Stirrup Creek Drive
Suite 150
Durham, NC 27703

2. SUMMARY

The objective of this study was to further characterize the toxicity, biodistribution, and gene expression of the test article, scAAV9/JeT-hCLN7opt-SV40pA (also known as CLN-7), following a single intrathecal (IT) injection in CD®[Crl:CD®(SD)] rats. The test article is being developed for treatment of the neurodegenerative Batten Disease (CLN7 subtype). The study design was as follows:

Text Table 1
Experimental Design

Group No.	Dose Level (vg/animal)	Dose Concentration (vg/µL)	Dose Volume (µL)	No. of Animals					
				Necropsy Day 8		Necropsy Day 29		Necropsy Day 91	
				M	F	M	F	M	F
1	0	0	60	5	5	5	5	5	5
2	$5x10^{11}$	$2.5x10^{10}$	20	5	5	5	5	5	5
3	$2x10^{12}$	$1x10^{11}$	20	5	5	5	5	5	5
4	$6x10^{12}$	$1x10^{11}$	60	5	5	5	5	5	5

M-Male
F-Female

The following parameters and end points were evaluated in this study: mortality, clinical observations, body weight, and food consumption, clinical pathology parameters (hematology, coagulation, and clinical chemistry), gross necropsy findings, organ weights, and histopathologic examinations, fusion v6 rotating rod system evaluation, serum and CSF analysis, biodistribution and gene expression analysis by qPCR and RT-qPCR. Exploratory serum and CSF analysis, as well as gene expression analysis, will be reported separately as needed.

Administration of the scAAV9/JeT-hCLN7opt-SV40pA was not associated with any mortality, clinical observations, accelerating rotarod values, bodyweight or food consumption changes. All animals survived to their scheduled terminal necropsies.

Single intrathecal injections of scAAV9/JeT-hCLN7opt-SV40pA to Sprague Dawley rats at Low Dose ($5x10^{11}$ vg/animal), Mid Dose ($2x10^{12}$ vg/animal) or High Dose ($6x10^{12}$ vg/animal) levels on Day 1 with an observation period through Day 91 resulted in test article-related increases in lymphocyte counts in both sexes at the Mid and High dose levels with concomitant increases in total leukocyte counts, which tended to partially resolve by the end of the observation period except in females at the High Dose levels. Additionally, males administered the High Dose level had a test article-related increase in fibrinogen concentration that had resolved by the end of the observation period. Males administered Mid and High Dose levels and females administered Low, Mid, and High Dose levels also had test article-related increases in globulin concentrations, which had partially resolved by the end of the observation period at the Low and Mid Dose

levels but persisted in both sexes at the High Dose level. These changes were collectively suggestive of a minor immune or inflammatory stimulus, which lacked definitive microscopic correlates.

Males administered the Low, Mid, and High Dose levels also had test article-related decreases in triglyceride concentrations that had resolved by the end of the observation period and lacked microscopic correlates.

Intrathecal administration of scAAV9/JeT-hCLN7opt-SV40pA once on Day 1 to rats at doses of 0, $5x10^{11}$, $2x10^{12}$ or $6x10^{12}$ vg/animal with an observation period of up to 91 days resulted in no unscheduled deaths. There were no test article-related macroscopic findings. There were no microscopic changes directly attributable to the administration of scAAV9/JeT-hCLN7opt-SV40pA.

Therefore, within the duration and design of this study the no-observed-adverse-effect-level (NOAEL) was $6x10^{12}$ vg/animal of scAAV9/JeT-hCLN7opt-SV40pA.

3. INTRODUCTION

Due to the acquisition of MPI Research by Charles River, the name of the MPI Research facility in Mattawan, Michigan has been changed to Charles River Laboratories, Inc., 54943 North Main Street, Mattawan, MI 49071, USA. Study documents may contain both names and both names are considered equivalent and may be used as the name of MPI Research transitions to Charles River.

The objective of this study was to further characterize the toxicity, biodistribution, and gene expression of the test article, scAAV9/JeT-hCLN7opt-SV40pA. The test article is being developed for treatment of the neurodegenerative Batten Disease (CLN7 subtype).

The design of this study was based on the current International Council for Harmonisation (ICH) Harmonised Tripartite Guidelines and generally accepted procedures for the testing of pharmaceutical compounds[1] and in accordance with the *Guide for the Care and Use of Laboratory Animals* from the National Research Council.[2] The study protocol, the last amended study protocol, and deviations are presented in Appendix 1.

3.1. Study Schedule

Study Initiation Date<br>(Protocol Signed by Study Director)	07 Sep 2018
Experimental Start Date<br>(First Day of Dose Administration)	14 Sep 2018
Experimental Termination Date<br>(Last Animal Removed from Study)	08 Jan 2019

4. MATERIALS AND METHODS

4.1. Test Article and Vehicle Information

4.1.1. Test Article

Identification: scAAV9/JeT-hCLN7opt-SV40pA (also known as CLN-7)

Batch (Lot) No.: MSJ-18-01-16-2

Receipt Date: 12 Sep 2018

Expiration Date: Not Available

Physical Description: Clear, colorless frozen liquid

Storage Conditions: Frozen at -60°C to -90°C, protected from light

Supplier: Vigene Biosciences Inc

4.1.2. Vehicle

Identification: Placebo

Batch (Lot) No.: MSJ-18-01-17-1

Receipt Date: 12 Sep 2018

Expiration Date: Not Available

Physical Description: Clear, colorless frozen liquid

Storage Conditions: Frozen at -60°C to -90°C, protected from light

Supplier: Vigene Biosciences Inc

4.2. Test Article and Vehicle Characterization

The Sponsor has not provided to the Testing Facility documentation of the identity, strength, purity, composition, and stability for the vehicle or test article. A Certificate of Analysis has not been provided to the Testing Facility and as presented in Appendix 2.

4.3. Reserve Samples

For the batch (lot) of control and test article, a reserve sample was collected and maintained under the appropriate storage conditions by the Testing Facility.

4.4. Test Article Inventory and Disposition

Records of the receipt, distribution, and storage of test and control articles were maintained. With the exception of reserve samples, all unused test article was discarded after completion of the study.

4.5. Dose Formulation and Analysis

4.5.1. Preparation of Vehicle

Fresh vehicle, isotonic phosphate-buffered saline containing 5% w/v sorbitol, was used as received from the Vigene Biosciences, Inc., Rockville, Maryland. The vehicle was removed from frozen storage and allow to thaw at controlled room temperature prior to formulation preparation or dosing, stored refrigerated (2ºC to 8ºC), and used within 2 weeks.

4.5.2. Preparation of Test Article

The test article, scAAV9/JeT-hCLN7opt-SV40pA (also known as CLN-7), was used as received from the Vigene Biosciences, Inc., Rockville, Maryland. No adjustment was made for purity. The high and middle doses were administered neat (undiluted). The test article was removed from frozen storage and allowed to thaw at controlled room temperature prior to formulation preparation or dosing, stored refrigerated (2ºC to 8ºC), and used within 2 weeks after thawing.

Biosafety Level 2 (BSL2) procedures were observed during dose formulation preparation in accordance with Testing Facility BLS2 Policy.

On the day of dosing, using sterile equipment, and aseptic techniques, formulations of test article for low dose, Group 2, were prepared by mixing the appropriate amount of vehicle with the appropriate amount of test article to achieve a nominal concentration of 2.5×10^{10} vg/µL. The low dose was diluted 1:4 (i.e. 1 part test article and 3 parts vehicle). Test article was mixed by inversion 5 times prior to being added to vehicle. The test article formulations were dispensed into polypropylene containers, inverted 10 times, and stored on wet ice under refrigeration prior to use.

On occasion, additional preparations were made due to insufficient amounts dispensed during the course of the study.

4.6. Test System

4.6.1. Receipt

From 07 Sep 2018 to 19 Dec 2018, 138 experimentally naïve CD®[CRL:CD®(SD), rats (69/sex) were received from Charles River Laboratories, Raleigh, North Carolina or Charles River Kingston, Stone Ridge, New York. At the initiation of dosing, the animals assigned to study were approximately 8 to 9 weeks old and weighed between 165 g and 328 g.

4.6.2. Justification for Test System and Number of Animals

The current state of scientific knowledge and the applicable guidelines cited previously in this protocol did not provide acceptable alternatives, in vitro or otherwise, to the use of live animals to accomplish the purpose of this study. “The development of knowledge necessary for the improvement of the health and well-being of humans as well as other animals requires in vivo experimentation with a wide variety of animal species.”[3] “Whole animals are essential in research and testing because they best reflect the dynamic interactions between the various cells, tissues, and organs comprising the human body.”[4]

The rat is the usual rodent model[5] used for evaluating the toxicity of various classes of chemicals and for which there is a large historical database.

4.6.3. Animal Identification

At study assignment, each animal was identified using a subcutaneously implanted electronic identification chip.

4.6.4. Environmental Acclimation

During the 7 to 11-day acclimation period, the animals were observed daily with respect to general health and any signs of disease.

4.6.5. Selection, Assignment, Replacement, and Disposition of Animals

Animals were assigned to study groups by a standard, by weight, randomization procedure designed to achieve similar group mean body weights. Animals assigned to study had body weights within ±20% of the mean body weight for each sex.

4.6.6. Husbandry

4.6.6.1. Housing

The animals were pair or group-housed in solid bottom cages with nonaromatic bedding. The housing was equipped with an automatic watering valve as specified in the *USDA Animal Welfare Act* (9 CFR, Parts 1, 2 and 3) and as described in the *Guide for the Care and Use of Laboratory Animals*.[2] Each cage was clearly labeled with study, group, animal number, and sex.

4.6.6.2. Environmental Conditions

Target temperatures of 68°F to 79°F with a relative target humidity of 30% to 70% were maintained. A 12-hour light/12-hour dark cycle was maintained, except when interrupted for designated procedures. Ten or greater air changes per hour with 100% fresh air (no air recirculation) were maintained in the animal rooms.

4.6.6.3. Food

Lab Diet® (Certified Rodent Diet #5002, PMI Nutrition International, Inc.) was provided ad libitum except during designated procedures. The feed was analyzed by the supplier for nutritional components and environmental contaminants. Results of the analysis are provided by the supplier and are on file at the Testing Facility. It is considered that there are no known contaminants in the feed that would interfere with the objectives of the study.

4.6.6.4. Water

Tap water was available ad libitum to each animal via an automatic watering system (except during designated procedures). Periodic analysis of the water is performed, and results of these analyses are on file at the Testing Facility. It is considered that there are no known contaminants in the water that could interfere with the outcome of the study.

4.6.6.5. Animal Enrichment

Psychological/environmental enrichment was provided according to SOP.

4.6.6.6. Veterinary Care

Veterinary care was available throughout the course of the study, and animals were examined by the veterinary staff as warranted by clinical signs or other changes. All veterinary examinations and recommended therapeutic treatments, if any, were documented in the study records and reviewed by the Study Director. The medical treatments and observations recorded after initiation of dosing are not reported but are maintained in the study file.

4.7. Experimental Design

4.7.1. Administration of Test Materials

The vehicle and test article were administered once on day of dosing via slow bolus intrathecal injection. The dose levels were 0, $5x10^{11}$, $2x10^{12}$, and $6x10^{12}$ vg/animal and administered at a dose volume of 20 or 60 µL. The control group received the vehicle in the same manner as the treated groups. Prior to dosing, vehicle or test article was warmed at controlled room temperature for 15 to 30 minutes and dosed within 2 hours. BLS2 procedures were observed during test article administration.

Prior to the first dose the needle was primed: the syringe (with a 26 gauge Hamilton needle attached) was filled with vehicle or test article. The Hamilton needle was removed, the Lasse needle attached, and the vehicle or test articled flushed through the needle. The syringe was then filled with the dose after the needle was primed.

The animal was anesthetized to effect with isoflurane using a precision vaporizer and placed in ventral recumbency. A single intrathecal dose was administered with a 27 gauge Lasse needle and 100 µL gas tight Hamilton syringe. A small incision was made to assist needle placement. Under fluoroscopic guidance, the needle was inserted into the lumbar cistern. Animals were returned to their cages and allowed to recover following dosing.

4.7.2. Justification of Route and Dose Levels

The dose level was selected by the Sponsor, or in consultation with the Sponsor, on the basis of available data from previous studies and discussion with the FDA in a preIND meeting. The low and middle doses had been tolerated well in pilot mouse studies, and the middle dose was the target dose to model the proposed clinical dose in CLN7 patients. The middle dose had shown sufficient to improve pathological signatures of the disease in mice. Per the discussion with the FDA, we were advised to bracket the “middle” target dose with lower and higher doses to complete the dose-responsive nature of any effect. Intrathecal injection is the intended route of administration of this test article in humans.

4.8. In-life Procedures, Observations, and Measurements

4.8.1. Cage Side Observations

All animals were observed for morbidity, mortality, injury, and the availability of food and water twice daily, once in the morning and once in the afternoon. Animals were not removed from the cage during observation, unless necessary for identification or confirmation of possible findings.

4.8.2. Detailed Clinical Observations

The animals were removed from the cage, and a detailed clinical examination of each animal was performed during the acclimation period (as needed), pretreatment, and weekly during the study (for deviations see Appendix 1). On occasion, clinical observations were recorded at unscheduled intervals. The examinations performed during the acclimation period are not reported but are maintained in the study file. The observations included, but were not limited to, evaluation of the skin, fur, eyes, ears, nose, oral cavity, thorax, abdomen, external genitalia, limbs and feet, respiratory and circulatory effects, autonomic effects such as salivation, and nervous system effects including tremors, convulsions, reactivity to handling, and unusual behavior.

4.8.3. Body Weights

Body weights for all animals were measured and recorded within 3 days of arrival, prior to randomization, Day 1, and weekly during the study (for deviations see Appendix 1). The body weights recorded within 3 days of arrival and prior to randomization are not reported but are maintained in the study file.

4.8.4. Food Consumption

Food consumption (per cage) was measured and recorded weekly throughout the study (for deviations see Appendix 1).

4.8.5. Fusion v6 Rotating Rod System

All animals scheduled for Day 29 and Day 91 necropsy were evaluated using the Acuscan EzRod rotating rod system using Fusion v6 software.

Prior to testing, a training session was conducted. The animal was placed on a rotating rod set at a constant speed of -4 rotations per minute (rpm) for 3 minutes (the negative sign indicates direction, in this case the animal ambulates away from the observer). Animals that fell off during session were placed back on the rotating rod until 3 minutes was completed. Data from the training sessions are not reported but are maintained in the study file.

For testing runs, the apparatus was set to accelerate from -4 rpm to -40 rpm over the course of 3 minutes. The acceleration profile was set up so that at 0.1 seconds after the trial was started (chamber button was pushed), the rod was rotating at -4 rpm and at 180.1 seconds the rotating rod was rotating at -40 rpm. The rod accelerated at a rate of 0.2 revolutions per second for 3 minutes. The elapsed time from the start of the trial to the animal's fall and the speed of the rod at that time was recorded digitally by the instrument. Each animal performed 3 trials with at least 15 minutes between trials.

Testing was initiated by pushing the chamber button then placing the rat on the rotating rod. If the animal fell but failed to trigger the sensors to terminate the run, the observer immediately terminated the run manually and was considered a valid run.

In the event the animal didn't fall but the run was terminated unexpectedly by the apparatus, the run was considered invalid and a re-run phase was initiated to retest the animal immediately. If the animal clung to the rod for two consecutive rotations, the testing session was stopped manually by the observer and was considered a valid run.

4.9. Laboratory Evaluations

4.9.1. Clinical Pathology

Clinical pathology evaluations were conducted on all animals at termination. The materials and methods are described in Appendix 9.

Bone marrow smears were collected and preserved.

4.9.2. Bioanalysis

4.9.2.1. Bioanalytical Sample Collection

Blood samples (approximately 0.6 mL to 1 mL) were collected at termination from all animals via the abdominal vessel vena cava after carbon dioxide inhalation for determination of the serum concentrations of the test article. The animals were not fasted prior to blood collection, with the exception of the intervals that coincided with fasting for clinical pathology collections. For deviations see Appendix 1.

4.9.2.2. Bioanalytical Sample Processing

Blood samples were collected in serum separator tubes and centrifuged at controlled room temperature. The resulting serum was divided into 3 aliquots (approximately 100 µL) in pre-labeled cryovials. On occasion, aliquots were stored on dry ice prior to final frozen storage. All aliquots were stored frozen at -60°C to -90°C within 60 minutes of centrifugation. For deviations see Appendix 1.

4.9.2.3. Bioanalytical Sample Analysis

Samples were shipped on dry ice to University of Texas Southwestern Medical Center-Gray Lab, Dallas, Texas, for possible analysis. If a sample analysis is conducted, the data will be reported separately.

4.9.3. Cerebrospinal Fluid (CSF) Collection and Analysis

CSF samples (maximum attainable) were collected from all animals at scheduled termination via the cisterna magna. CSF samples were collected in 2 approximately equal aliquots in prelabeled cryovials. On occasion, aliquots were stored on dry ice prior to final frozen storage. All aliquots were stored frozen at -60ºC to -90ºC. For deviations see Appendix 1.

Samples were shipped on dry ice to the Laboratory of Dr. Steven Gray, University of Texas Southwestern Medical Center-Gray Lab, Dallas, Texas, for possible future analysis. If a sample analysis is conducted, the data will be reported separately.

4.10. Terminal Procedures

Postmortem study evaluations were performed on animals at the scheduled terminal necropsies.

4.10.1. Macroscopic

Necropsy examinations were performed under procedures approved by a veterinary pathologist. The animals were examined carefully for external abnormalities including palpable masses. The skin was reflected from a ventral midline incision and subcutaneous masses were identified and correlated with antemortem findings. The abdominal, thoracic, and cranial cavities were examined for abnormalities. The organs were removed, examined, and, where required, placed in fixative. The pituitary was fixed in situ. All designated tissues were fixed in neutral buffered formalin, except for the eyes (including the optic nerve) and testes, which were fixed using a modified Davidson's fixative[6] prior to placement in formalin. Tissue samples collected for analysis of the test article concentrations were not placed in fixative. Formalin was infused into the lung via the trachea and into the urinary bladder. A full complement of tissues and organs was collected from all animals.

4.10.2. Organ Weights

Body weights and protocol-designated organ weights were recorded for all surviving animals at the scheduled necropsies and appropriate organ weight ratios were calculated (relative to body and brain weights). Paired organs were weighed together. A combined weight for the thyroid and parathyroid glands was collected. Only the right mandibular salivary gland was weighed.

4.10.3. Microscopic

Fixed hematoxylin and eosin-stained paraffin sections from protocol-designated sections of tissues were processed to slide and all required slides were shipped under ambient condition to Charles River Laboratories, Inc., Durham, North Carolina for microscopic evaluation.

The spinal cord was opened up to remove bilateral DRG's (including nerve roots, when possible) from the cervical, thoracic, and lumber regions of the spinal column. The DRG's were submitted for processing in individual tissue cassettes and left and right orientation maintained. Spinal sections adjacent to the harvested DRG's were trimmed and submitted for processing.

4.10.4. Biodistribution and Gene Expression Evaluations

Tissue samples were collected from the brain (at least 3 coronal cross sections: hindbrain including brainstem and cerebellum, midbrain, and forebrain), heart, kidney, liver, lung, lymph node (mandibular and mesenteric), skeletal muscle (bicep femoris and gastrocnemius), ovary, cervical spinal cord (including DRG and nerve roots), thoracic spinal cord (including DRG and nerve roots), lumbar spinal cord (including DRG and nerve roots), and testes of all animals for analysis of the test article concentrations. Each organ/tissue sample was divided in half; one half for DNA purification and the other half for RNA purification. When possible, duplicated samples were collected (4 total per tissue/organ) as long as each sample was ≥10 mg.

Samples for DNA analysis were collected using strict aseptic techniques and disposable instruments for each tissue or organ. Gloves were changed between collection and dissection of each tissue for PCR analysis. Non-disposable instruments and cutting board were wiped down with 10% bleach solution, rinsed with water, and wiped down with 100% ethanol between each of the specified organs. Samples for PCR were collected prior to sample collection for any other parameters. Samples of organs or tissues collected for qPCR were placed in prelabeled 2 mL microfuge tubes (1 tube/sample), snap-frozen in liquid nitrogen, and store frozen at -60ºC to -90ºC. Samples collected for RT-qPCR analysis of the transgene mRNA were completely immersed into prelabeled 2 mL microfuge prefilled with 1.5 mL RNALater. Samples were stored refrigerated (2ºC to 8ºC) for up to 24 hours, RNALater removed, and stored frozen (-60C to -90C).

Samples were shipped on dry ice to the Laboratory of Dr. Steven Gray, University of Texas Southwestern Medical Center-Gray Lab, Dallas, Texas, for analysis. Samples for gene expression via RT-qPCR were not analyzed. If analyzed, the data will be reported separately. All analytical work will be conducted by the Laboratory of Dr. Steven Gray using an analytical method to be added by amendment if analysis is conducted.

5. STATISTICS

Text Table 2 defines the set of comparisons used in the statistical analyses described in this section.

Text Table 2
Statistical Comparisons

Control Group	Treatment Group
1	2, 3, 4

The raw data were tabulated within each time interval, and the mean and standard deviation were calculated for each endpoint by sex and group. For each endpoint, treatment groups were compared to the control group using the analysis outlined in Text Table 3.

Text Table 3
Statistical Analysis

Endpoints	Type of Analysis
Body Weights	Group Pair-wise Comparisons (General ANOVA)
Body Weight Change	
Food Consumption	
Hematology	
Coagulation	
Clinical Chemistry	
Urinalysis<br>Urine Volume<br>Specific Gravity<br>pH	
Organ Weights	
Absolute Weights	
Relative to Body and Brain Weights	

5.1. Group Pair-wise Comparisons (General ANOVA)

Included below are the details of the statistical routines that were applied to the data, dependent on the data specific assumptions outlined as part of the routine. The actual analysis performed for each endpoint and collection interval is included in the summary tables. The experimental unit for the analysis of food consumption was cage, while for all other endpoints the experimental unit was the individual animal. Food consumption was calculated as described in the Testing Facility SOP.

If the control group had a sample size less than three, no inferential statistics were calculated. If a particular endpoint and/or parameter within a given collection interval had the same value across all experimental units, no inferential statistics were calculated.

For endpoints and/or parameters where all groups with sample sizes of three or greater were included, the normality of the residuals and homogeneity of variances were tested to determine if the data were approximately normal or if a log transformation or rank transformation was required. Levene's test[7] was used to assess homogeneity of group variances and Shapiro-Wilk's[8] test was used to test the normality of the residuals.

For the raw data, if Levene's test was not significant ($p \geq 0.01$) and Shapiro-Wilk's test was not significant ($p \geq 0.01$), then a normal distribution was used. If either the Levene's test was significant ($p < 0.01$) or Shapiro-Wilk's test was significant ($p < 0.01$), normality and homogeneity of variances were tested with a log transformation used on the data.

For the log transformed data, if Levene's test was not significant ($p \geq 0.01$) and Shapiro-Wilk's test was not significant ($p \geq 0.01$), then a log normal distribution was used. If either the Levene's test was significant ($p < 0.01$) or Shapiro-Wilk's test was significant ($p < 0.01$), then a rank transformation was used on the data.

For raw or log transformed data, a one way analysis of variance was used to test each endpoint for the effects of treatment[9]. If the treatment effect was significant ($p < 0.05$), linear contrasts were constructed for a Dunnett's[9] pair-wise comparison of treatment groups as described above.

For rank transformed data, a Kruskal-Wallis test was used to test each endpoint for the effects of treatment[9]. If the treatment effect was significant ($p < 0.05$), a non-parametric Dunn's[9] pair-wise comparison test of each treatment group with the control group was performed.

Results of all pair-wise comparisons are reported at the 0.05 and 0.01 significance levels. All endpoints were analyzed using two-tailed tests.

6. COMPUTER SYSTEMS

Critical computerized systems used in the study are listed below or presented in the appropriate Phase Report. All computerized systems used in the conduct of this study have been validated; when a particular system has not satisfied all requirements, appropriate administrative and procedural controls were implemented to assure the quality and integrity of data.

Text Table 4
Critical Computerized Systems

System Name	Version No.	Description of Data Collected and/or Analyzed
Logbook	5.3	Electronic notebook and data collection system for veterinary communications, observations, and treatments.
MPI Research ExyLIMS	3.0	A comprehensive laboratory information management system used to manage data, including but not limited to: instrumentation, test articles, standards, and samples.
NextDocs®	6.1	Electronic documentation management of Deviation Events and Corrective and Preventative Actions (CAPA).
Provantis™	8.7, 9.4	Client-server, Oracle-based system used for electronic documentation and data management from compound receipt through reporting.
SAS®	9.2, 9.3	The SAS® System is an integrated system of software products that enables a user to perform data entry, retrieval, data management, reporting, graphics, statistical analysis, and applications development.
SAS/STAT®	14.1	Software used for statistical procedures.
R	3.5.1	Software used for statistical procedures.
Siemens Environmental Monitoring	3.11	Environmental monitoring, alarming, and reporting applications.
Niagara Framework® Software System	2.3	
Acuscan EZRod System with Fusion v6 Superflex Edition:	3.0	Software used to record data for the Acuscan EZRod system.

7. RETENTION OF RECORDS, SAMPLES, AND SPECIMENS

All study-specific raw data, documentation, records, protocol, samples, specimens, and final reports from this study were archived at the Testing Facility. At least one year after issue of the draft report, the Sponsor will be contacted. Data and specimens shipped to the Sponsor or Sponsor-designated location for Biodistribution were maintained by University of Texas Southwestern Medical Center-Gray Lab. Details regarding the retention of the materials were provided to the Study Director for inclusion in the Final Report.

8. RESULTS

8.1. Mortality

A record of animal fate and disposition is presented in Appendix 3.

All animals survived to the scheduled necropsy.

8.2. In-life Examinations

8.2.1. Detailed Clinical Observations

Detailed clinical observations are summarized in Table 1. Individual detailed clinical observations are presented in Appendix 4.

There were no scAAV9/JeT-hCLN7opt-SV40pA-related clinical observations. Clinical findings that were observed were not considered to be test article-related. These observations were generally sporadic, low in frequency, lacked dose-dependency, were considered injury or blood collection-related, occurred at similar frequency to controls, and/or were considered incidental as common findings in rats of this strain/age.

On a few occasions, loss of skin elasticity was observed and was due to dehydration from a sipper that was flowing too slowly. This was observed on only a single occasion per affected cage: animal number 1504 and 1505 during week 7, animal number 2005 during week 3, and animal number 3003 during week 9.

On occasion, veterinary observations were conducted during the course of the study for health monitoring purposes. In general, veterinary observations were similar to, and supported by, the detailed clinical observations.

8.2.2. Body Weight

Body weight data are illustrated in Figure 1 and summarized in Table 2. Individual body weight values are presented in Appendix 5. Body weight change data are illustrated in Figure 2 and summarized in Table 3. Individual body weight change values are presented in Appendix 6.

There was no scAAV9/JeT-hCLN7opt-SV40pA -related effect on body weight.

Mean body weight gains in treated males and females were similar to the IT vehicle controls. Occasional differences from controls were noted but were of low magnitude and considered to reflect normal biological variation. In a few instances body weight losses and subsequent gains the following week were noted and were due to dehydration from a sipper that was flowing too slowly. This was observed on only a single occasion per affected cage: Animal No. 1504 and 1505 (cage 8) on Day 43, Animal No. 2004 and 2005 (cage 14) on Day 15, Animal No. 3001 and 3002 (cage 25) on Day 64, and Animal No. 3003, 3004, and 3005 (cage 26) on Day 60.

8.2.3. Food Consumption

Food consumption data are illustrated in Figure 3 and summarized in Table 4. Individual caged food consumption values are presented in Appendix 7.

There was no scAAV9/JeT-hCLN7opt-SV40pA-related effect on food consumption. Food consumption was similar among treatment groups and controls. Occasional differences from controls were noted but were of low magnitude and considered to reflect normal biological variation. In a few instances decreases in food consumption were noted and were due to dehydration from a sipper that was flowing too slowly. This was observed on only a single

occasion per affected cage: Animal No. 1504 and 1505 (cage 8) on Day 43, Animal No. 2004 and 2005 (cage 14) on Day 15, Animal No. 3001 and 3002 (cage 25) on Day 64, and Animals No. 3003, 3004, and 3005 (cage 26) on Day 60.

8.2.4. Fusion v6 Rotating Rod System

Fusion v6 Rotating Rod System data are summarized in Table 5. Fusion v6 Rotating Rod System evaluations are presented in Appendix 8.

No test article-related effects were observed following the administration of scAAV9/JeT-hCLN7opt-SV40pA. Accelerating rotarod values were similar across all groups.

8.3. Clinical Pathology

The Clinical Pathology Report is presented in Appendix 9.

8.3.1. Summary

Single intrathecal injections of scAAV9/JeT-hCLN7opt-SV40pA to Sprague Dawley rats at Low Dose ($5x10^{11}$ vg/animal), Mid Dose ($2x10^{12}$ vg/animal) or High Dose ($6x10^{12}$ vg/animal) levels on Day 1 with an observation period through Day 91 resulted in test article-related increases in lymphocyte counts in both sexes at the Mid and High dose levels with concomitant increases in total leukocyte counts, which tended to partially resolve by the end of the observation period except in females at the High Dose levels. Additionally, males administered the High Dose level had a test article-related increase in fibrinogen concentration that had resolved by the end of the observation period. Males administered Mid and High Dose levels and females administered Low, Mid, and High Dose levels also had test article-related increases in globulin concentrations, which had partially to fully resolved by the end of the observation period at the Low and Mid Dose levels but persisted in both sexes at the High Dose level. These changes were collectively suggestive of a minor immune or inflammatory stimulus, which lacked definitive microscopic correlates.

Males administered the Low, Mid, and High Dose levels also had test article-related decreases in triglyceride concentrations that had resolved by the end of the observation period and lacked microscopic correlates.

8.3.2. Hematology

Table 1 and Appendix 1 of the Clinical Pathology Report.

On Days 8, 29, and 91 in both sexes at the Mid Dose and High Dose there were minimal to mild increases in mean lymphocyte counts with concomitant increases in mean total leukocyte counts, as illustrated in Text Table 4. These changes tended to be most pronounced on Day 29, except in High Dose females that had the most pronounced change on Day 91. These changes were considered test article-related. Along with increases in fibrinogen concentrations (see Section 8.3.3) and globulin concentrations (see Section 8.3.4), these changes were collectively suggestive of a minor immune or inflammatory stimulus, which lacked definitive microscopic correlates.

Text Table 4
scAAV9/JeT-hCLN7opt-SV40pA-Related Hematology Changes

Group<br>Dose Level<br>Sex	2<br>Low Dose<br>M	 <br>F	3<br>Mid Dose<br>M	 <br>F	4<br>High Dose<br>M	 <br>F
**Lymphocytes**						
Day 8	–	–	1.24x	1.18x	1.18x	1.16x
Day 29	–	–	1.54x	1.33x	1.71x	1.20x
Day 91	–	–	1.20x	1.30x	1.25x	1.67x
**Total Leukocytes**						
Day 8	–	–	1.21x	1.18x	1.13x	1.15x
Day 29	–	–	1.48x	1.26x	1.60x	1.15x
Day 91	–	–	1.10x	1.25x	1.17x	1.60x

M = Males F = Females
A dash (—) indicates absence of a test article-related change. Numerical values indicate fold (x) change of the treated group mean value relative to the concurrent control group mean value.

All other fluctuations among individual and mean hematology values, regardless of statistical significance, were not considered test article-related due to one or more of the following: negligible magnitude, inconsistent direction of change, lack of a dose-responsive pattern, and/or relation to expected values for biologic and procedure-related variation.

8.3.3. Coagulation

Table 2 and Appendix 2 of the Clinical Pathology Report.

On Day 29 in males at the High Dose there was a minimal increase in mean fibrinogen concentration (1.25x), which had resolved by Day 91. This increase in fibrinogen concentration was considered test article-related and suggestive of a minor immune or inflammatory stimulus as previously discussed.

All other fluctuations among individual and mean coagulation values, regardless of statistical significance, were not considered test article-related due to one or more of the following: negligible magnitude, inconsistent direction of change, lack of a dose-responsive pattern, and/or relation to expected values for biologic and procedure-related variation.

8.3.4. Clinical Chemistry

Table 3 and Appendix 3 of the Clinical Pathology Report.

As illustrated in Text Table 5, on Day 29 in males at the Mid and High Dose levels and females at all dose levels there were minimal increases in mean globulin concentrations. These changes had partially to fully resolved by Day 91 at the Low and Mid Dose levels, but persisted at the High Dose. These changes were considered test article-related and suggestive of a minor immune or inflammatory stimulus, as previously discussed.

On Day 8 males at all dose levels had mild decreases in mean triglyceride concentrations, which had partially resolved by Day 29 and fully resolved by Day 91. These decreases in triglyceride concentrations were considered test article-related. No microscopic correlates were observed.

Text Table 5
scAAV9/JeT-hCLN7opt-SV40pA-Related Clinical Chemistry Changes

Group<br>Dose Level<br>Sex	2<br>Low Dose<br>M	 <br>F	3<br>Mid Dose<br>M	 <br>F	4<br>High Dose<br>M	 <br>F
**Globulin**						
Day 8	–	–	–	–	–	–
Day 29	–	1.10x	1.12x	1.10x	1.08x	1.09x
Day 91	–	1.05x	1.07x	–	1.09x	1.08x
**Triglycerides**						
Day 8	0.53x	–	0.57x	–	0.50x	–
Day 29	0.77x	–	0.82x	–	0.82x	–
Day 91	–	–	–	–	–	–

M = Males F = Females
A dash (—) indicates absence of a test article-related change. Numerical values indicate fold (x) change of the treated group mean value relative to the concurrent control group mean value.

All other fluctuations among individual and mean clinical chemistry values, regardless of statistical significance, were not considered test article-related due to one or more of the following: negligible magnitude, inconsistent direction of change, lack of a dose-responsive pattern, and/or relation to expected values for biologic and procedure-related variation.

8.4. Terminal Evaluations

The Pathology Report is presented in Appendix 10.

8.4.1. Mortality

There were no main study unscheduled deaths during this study.

8.4.2. Gross Pathology

8.4.2.1. Terminal Euthanasia Animals (Day 8)

Table 1 and Appendix 5 of the Pathology Report.

No gross (macroscopic) findings were noted for male or female animals at this interval.

8.4.2.2. Terminal Euthanasia Animals (Day 29)

Table 2 and Appendix 6 of the Pathology Report.

No test article-related gross findings were noted. The gross findings observed were considered incidental, of the nature commonly observed in this strain and age of rat and/or were of similar incidence in control and treated animals and, therefore, were considered unrelated to administration of scAAV9/JeT-hCLN7opt-SV40pA.

8.4.2.3. Terminal Euthanasia Animals (Day 91)

Table 3 and Appendix 7 of the Pathology Report.

No test article-related gross findings were noted. The gross findings observed were considered incidental, of the nature commonly observed in this strain and age of rat and/or were of similar

incidence in control and treated animals and, therefore, were considered unrelated to administration of scAAV9/JeT-hCLN7opt-SV40pA.

8.4.3. Organ Weights

8.4.3.1. Terminal Euthanasia Animals (Day 8)

Table 4 and Appendix 2 of the Pathology Report.

No test article-related organ weight changes were noted. There were isolated organ weight values that were different from their respective controls. There were, however, no patterns, trends, or correlating data to suggest these values were toxicologically relevant. Thus, the organ weight differences observed were considered incidental and unrelated to administration of scAAV9/JeT-hCLN7opt-SV40pA.

8.4.3.2. Terminal Euthanasia Animals (Day 29)

Table 5 and Appendix 3 of the Pathology Report.

Potential test article-related organ weight changes noted at the end of Day 29 and are summarized in Text Table 6.

Text Table 6
Summary of Organ Weight Data – Terminal Euthanasia (Day 29)

	Males		
**Group**	**2**	**3**	**4**
**Dose (vg/day)**	**$5x10^{11}$**	**$2x10^{12}$**	**$6x10^{12}$**
**No. Animals per Group**	**5**	**5**	**5**
**Thyroid (No. Weighed)[a]**	**5**	**5**	**5**
Absolute value	-11.7	**-22.5**	**-22.3**
% of body weight	-12.7	**-20.0**	**-23.1**
% of brain weight	-11.3	**-21.7**	**-23.4**

[a] All values expressed as percent difference of control group means.

Based upon statistical analysis of group means, values highlighted in bold are significantly different from control group – $P \leq 0.01$; refer to data tables for actual significance levels and tests used.

Decreased thyroid weights in male animals were statistically significant at $\geq 2x10^{12}$ vg/animal. As per protocol, the thyroid gland was not evaluated microscopically.

No other test article-related organ weight changes were noted. There were other isolated organ weight values that were statistically different from their respective controls. There were, however, no patterns, trends, or correlating data to suggest these values were toxicologically relevant. Thus, other organ weight differences observed were considered incidental and unrelated to administration of scAAV9/JeT-hCLN7opt-SV40pA.

8.4.3.3. Terminal Euthanasia Animals (Day 91)

Table 6 and Appendix 4 of the Pathology Report.

Potential test article-related organ weight changes noted at the Day 91 terminal euthanasia and potential test article-related organ weight changes noted at the Day 29 terminal euthanasia that were not observed at the end of Day 91 are summarized in Text Table 7.

Text Table 7
Summary of Organ Weight Data – Terminal Euthanasia (Day 91)

	Males		
**Group**	**2**	**3**	**4**
**Dose (vg/animal)**	**$5x10^{11}$**	**$2x10^{12}$**	**$6x10^{12}$**
**No. Animals per Group**	**5**	**5**	**5**
**Thymus (No. Weighed)[a]**	**5**	**5**	**5**
Absolute value	13.0	46.9	**64.6**
% of body weight	14.6	36.2	**56.0**
% of brain weight	16.2	52.7	**70.1**
**Thyroid (No. Weighed)**	**5**	**5**	**5**
Absolute value	-7.5	15.6	8.1
% of body weight	-6.9	6.8	9.4
% of brain weight	-6.0	19.2	10.4

[a] All values expressed as percent difference of control group means.

Based upon statistical analysis of group means, values highlighted in bold are significantly different from control group – $P \leq 0.05$; refer to data tables for actual significance levels and tests used.

Increased thymus weights were observed in males in a dose dependent manner and became statistically significant at the highest dose. Increased thymus weights had no histopathological correlate and were considered to be of little toxicological significance.

Potential dose dependent decreases in thyroid weight that were observed in males on Day 29 were not observed on Day 91 and actual increases in thyroid weights were observed at $6x10^{12}$ vg/animal. These data, together with the small magnitude and transiency of the changes observed, support an unlikely relationship to treatment and no toxicological significance.

A statistically significant increase was observed for the spleen of females treated at $6x10^{12}$ vg/animal. Spleen weight increases were of small magnitude, were not in a dose dependent manner and did not have a relevant correlating microscopic effect and were thus considered within expected biological variability for this organ and not related to test article administration.

No other test article-related organ weight changes were noted. There were other isolated organ weight values that were statistically different from their respective controls. There were, however, no patterns, trends, or correlating data to suggest these values were toxicologically relevant. Thus, other organ weight differences observed were considered incidental and unrelated to administration of scAAV9/JeT-hCLN7opt-SV40pA.

8.4.4. Histopathology

8.4.4.1. Terminal Euthanasia (Day 8)

Table 7 and Appendix 5 of the Pathology Report.

Microscopic findings are summarized in Text Table 8.

Text Table 8
Summary of Microscopic Findings – Terminal Euthanasia (Day 8)

	Males				Females			
**Group**	1	**2**	**3**	**4**	**1**	**2**	**3**	**4**
**Dose (vg/animal)**	**0**	**5x10^11^**	**2x10^12^**	**6x10^12^**	**0**	**5x10^11^**	**2x10^12^**	**6x10^12^**
**No. Animals Examined**	**5**	**5**	**5**	**5**	**5**	**5**	**5**	**5**
**Dorsal Root Ganglia, Lumbar right (No. Examined)**	(5)	(5)	(5)	(5)	(5)	(5)	(5)	(5)
Infiltration, mononuclear perivascular								
Minimal	0	0	1	1	1	1	1	0
**Dorsal Root Ganglia, Lumbar left (No. Examined)**	(5)	(5)	(5)	(5)	(4)	(5)	(5)	(5)
Infiltration, mononuclear perivascular								
Minimal	1	1	0	1	0	1	0	1
**Spleen (No. Examined)**	(5)	(5)	(5)	(5)	(5)	(5)	(5)	(5)
Depletion, marginal zone								
Minimal	0	0	0	0	0	0	0	3
Mild	0	0	0	0	0	0	0	1
Marked	0	0	0	0	0	0	0	1
**Spinal Cord, Lumbar (No. Examined)**	(5)	(5)	(5)	(5)	(5)	(5)	(5)	(5)
Gliosis								
Minimal	0	0	0	0	0	0	2	0
Infiltration, mononuclear perivascular								
Minimal	0	0	0	0	0	0	2	0
**Spinal Cord, Thoracic (No. Examined)**	(5)	(5)	(5)	(5)	(5)	(5)	(5)	(5)
Gliosis								
Minimal	0	0	0	0	0	0	1	0
Infiltration, mononuclear perivascular								
Minimal	0	0	0	0	0	0	1	0

Minimal to marked depletion of the splenic marginal zone was observed in females treated at $6x10^{12}$ vg/animal. Decrease in B lymphocytes (main component) of the marginal zone have been associated with stress related response in rodent preclinical studies.[10] Additionally, this finding was not observed at latter time points and was interpreted likely a transient secondary effect to test article administration and therefore of limited toxicological relevance.

Minimal perivascular mononuclear infiltrates in the right and/or left lumbar ganglia were observed with a comparable incidence across treatment groups, these were attributed to the administration procedure and not directly to test article administration.

Likewise, focal minimal gliosis and minimal perivascular infiltrates were observed in the lumbar and/or the thoracic spinal cord in individual females treated at $2x10^{12}$ vg/animal. These were secondary reactivity to the administration procedure and were not considered to be directly attributable to a test article effect.

Other microscopic findings observed were considered incidental, of the nature commonly observed in this strain and age of rat, and/or were of similar incidence and severity in control and treated animals and, therefore, were considered unrelated to administration of scAAV9/JeT-hCLN7opt-SV40pA.

8.4.4.2. Terminal Euthanasia (Day 29)

Table 8 and Appendix 6 of the Pathology Report.

Microscopic findings noted at the end of Day 29 and are summarized in Text Table 9.

Text Table 9
Summary of Microscopic Findings – Terminal Euthanasia (Day 29)

	Males				Females			
**Group**	1	**2**	**3**	**4**	**1**	**2**	**3**	**4**
**Dose (vg/animal)**	**0**	**5x10^11^**	**2x10^12^**	**6x10^12^**	**0**	**5x10^11^**	**2x10^12^**	**6x10^12^**
**No. Animals Examined**	**5**	**5**	**5**	**5**	**5**	**5**	**5**	**5**
**Dorsal Root Ganglia, Lumbar right (No. Examined)**	(5)	(5)	(5)	(5)	(5)	(5)	(5)	(5)
Infiltration, mononuclear perivascular								
Minimal	0	1	1	2	1	1	0	1
Mild	0	1	0	0	0	0	0	0
**Dorsal Root Ganglia, Lumbar left (No. Examined)**	(5)	(5)	(5)	(5)	(5)	(5)	(5)	(5)
Infiltration, mononuclear perivascular								
Minimal	0	1	1	1	0	1	2	2

Minimal perivascular mononuclear infiltrates in the right and/or left lumbar ganglia were observed with an overall comparable incidence across treatment groups and were attributed to the administration procedure and not directly to test article administration. In the groups administered the test article, there was a potential very slight trend, that had no clear dose response, for increased finding incidence and severity that could possibly be attributable to reactivity to the presence of the test article.

Other microscopic findings observed were considered incidental, of the nature commonly observed in this strain and age of rat, and/or were of similar incidence and severity in control and treated animals and, therefore, were considered unrelated to administration of scAAV9/JeT-hCLN7opt-SV40pA.

8.4.4.3. Terminal Euthanasia (Day 91)

Table 9 and Appendix 7 of the Pathology Report.

Microscopic findings noted at the end of Day 91 and are summarized in Text Table 10.

Text Table 10
Summary of Microscopic Findings – Terminal Euthanasia (Day 91)

	Males				Females			
**Group**	1	**2**	**3**	**4**	**1**	**2**	**3**	**4**
**Dose (vg/animal)**	**0**	**5x10^11^**	**2x10^12^**	**6x10^12^**	**0**	**5x10^11^**	**2x10^12^**	**6x10^12^**
**No. Animals Examined**	**5**	**5**	**5**	**5**	**5**	**5**	**5**	**5**
**Dorsal Root Ganglia, Lumbar right (No. Examined)**	(5)	(5)	(5)	(5)	(5)	(5)	(5)	(5)
Infiltration, mononuclear perivascular								
Minimal	0	0	0	0	0	0	3	0
**Dorsal Root Ganglia, Lumbar left (No. Examined)**	(5)	(5)	(5)	(5)	(5)	(5)	(5)	(5)
Infiltration, mononuclear perivascular								
Minimal	0	1	0	0	1	0	2	0

Minimal perivascular mononuclear infiltrates in the right and/or left lumbar ganglia were observed with an overall comparable incidence across treatment groups, that decreased in severity and incidence by Day 91. These were attributed to the administration procedure and not directly to test article administration.

Other microscopic findings observed were considered incidental, of the nature commonly observed in this strain and age of rat, and/or were of similar incidence and severity in control and treated animals and, therefore, were considered unrelated to administration of scAAV9/JeT-hCLN7opt-SV40pA.

8.5. Biodistribution Evaluations

The Biodistribution Report is presented in Appendix 11.

IT delivery of AAV9/CLN7 vector results in dose dependent increase of CLN7 vector DNA across the central nervous system (brain and spinal cord) and peripheral organs (heart, lung, liver, kidney, ovary, and testes). The CLN7 vector DNA is concentrated closest to the injection site in the spinal cord and detected at lower levels in multiple brain regions. In the peripheral organs, similar high amounts of CLN7 DNA persist in liver and heart and to the less extent in testes, ovary, lung, and kidney. The pattern of CLN7 biodistribution in this study is consistent with that expected from AAV9 and observed in a previous study from Dr. Gray's laboratory where a similar vector, scAAV9/JeT-hGANopt-spA, was injected IT to WT rats at a dose of 6.6×10^{11} vg per rat (Gray lab, unpublished findings). Collectively, IT delivery of AAV9/CLN7 results in broad CLN7 biodistribution across rat body.

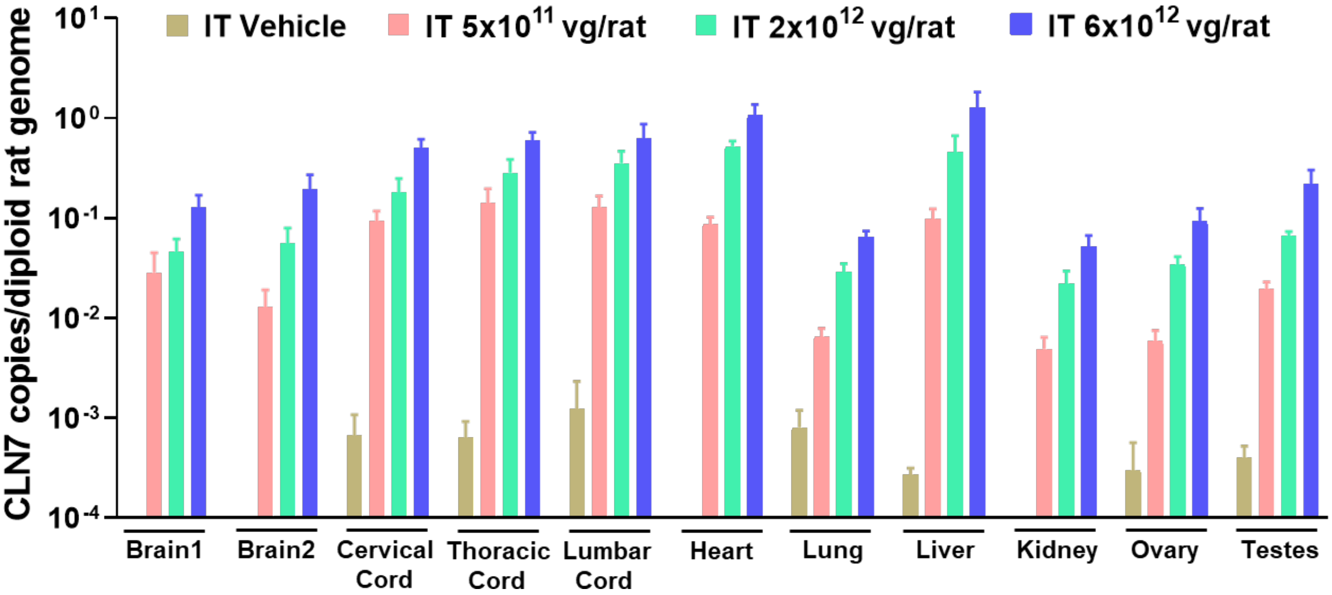

Figure 1. AAV9/CLN7 biodistribution in normal rats.

9. CONCLUSION

The objective of this study, to further characterize the toxicity and biodistribution of the test article, scAAV9/JeT-hCLN7opt-SV40pA, following a single intrathecal (IT) injection in CD®[Crl:CD®(SD)] rats, was met. Administration of the scAAV9/JeT-hCLN7opt-SV40pA was not associated with any mortality, clinical observations, accelerating rotarod values, bodyweight or food consumption changes. All animals survived to their scheduled terminal necropsies.

Single intrathecal injections of scAAV9/JeT-hCLN7opt-SV40pA to Sprague Dawley rats at Low Dose ($5x10^{11}$ vg/animal), Mid Dose ($2x10^{12}$ vg/animal) or High Dose ($6x10^{12}$ vg/animal) levels on Day 1 with an observation period through Day 91 resulted in test article-related increases in lymphocyte counts in both sexes at the Mid and High dose levels with concomitant increases in total leukocyte counts, which tended to partially resolve by the end of the observation period except in females at the High Dose levels. Additionally, males administered the High Dose level had a test article-related increase in fibrinogen concentration that had resolved by the end of the observation period. Males administered Mid and High Dose levels and females administered Low, Mid, and High Dose levels also had test article-related increases in globulin concentrations, which had partially resolved by the end of the observation period at the Low and Mid Dose levels but persisted in both sexes at the High Dose level. These changes were collectively suggestive of a minor immune or inflammatory stimulus, which lacked definitive microscopic correlates.

Males administered the Low, Mid, and High Dose levels also had test article-related decreases in triglyceride concentrations that had resolved by the end of the observation period and lacked microscopic correlates.

Intrathecal administration of scAAV9/JeT-hCLN7opt-SV40pA once on Day 1 to rats at doses of 0, $5x10^{11}$, $2x10^{12}$ or $6x10^{12}$ vg/animal with an observation period of up to 91 days resulted in no unscheduled deaths. There were no test article-related macroscopic findings. There were no microscopic changes directly attributable to the administration of scAAV9/JeT-hCLN7opt-SV40pA.

Therefore, within the duration and design of this study the no-observed-adverse-effect-level (NOAEL) was $6x10^{12}$ vg/animal of scAAV9/JeT-hCLN7opt-SV40pA.

10. REFERENCES

1. Guidance on non-clinical safety studies for the conduct of human clinical trials and marketing authorization for pharmaceuticals. ICH M3(R2), 2009 June 11 (ICH Implementation Date).
2. Guide for the Care and Use of Laboratory Animals. 8th ed., Washington, D.C., National Academies Press, 2011.
3. Principles for the utilization and care of vertebrate animals used in testing, research, and training. Federal Register, 1985 May 20; 50(97).
4. Position statement on the use of animals in research. NIH Guide, 1993 Feb 26; 22(8).
5. Guidance for industry, investigators, and reviewers: exploratory IND studies. U.S. F.D.A. Center for Drug Evaluation and Research (CDER), 2006 Jan.
6. Latendresse JR, Warbrittion AR, Jonassen H, Creasy DM. Fixation of testes and eyes using a modified Davidson’s fluid: comparison with Bouin’s fluid and conventional Davidson’s fluid. Toxicol Pathol. 2002 Jul Aug;30(4):524 33.
7. Milliken GA, Johnson DE. Analysis of messy data. London: Chapman and Hall: 1992.
8. Royston, JP. Approximating the Shapiro-Wilk W Test for Nonnormality. Statistics and Computing 1992; 2:117–119.
9. Zar JH. Biostatistical analysis, 4th ed. New Jersey: Prentice Hall; 1999.
10. Everds NE, Snyder PW, Bailey KL, Bolon B, Creasy DM, Foley GL, Rosol TJ, Sellers T., Interpreting stress responses during routine toxicity studies: a review of the biology, impact, and assessment., Toxicol Pathol. 2013;41(4):560-614.

Figure 1
Mean Body Weight Values

2759-001
A Single Dose Toxicity Study of Test Article Administered by Intrathecal Injection in Rats

Mean Body Weight Values

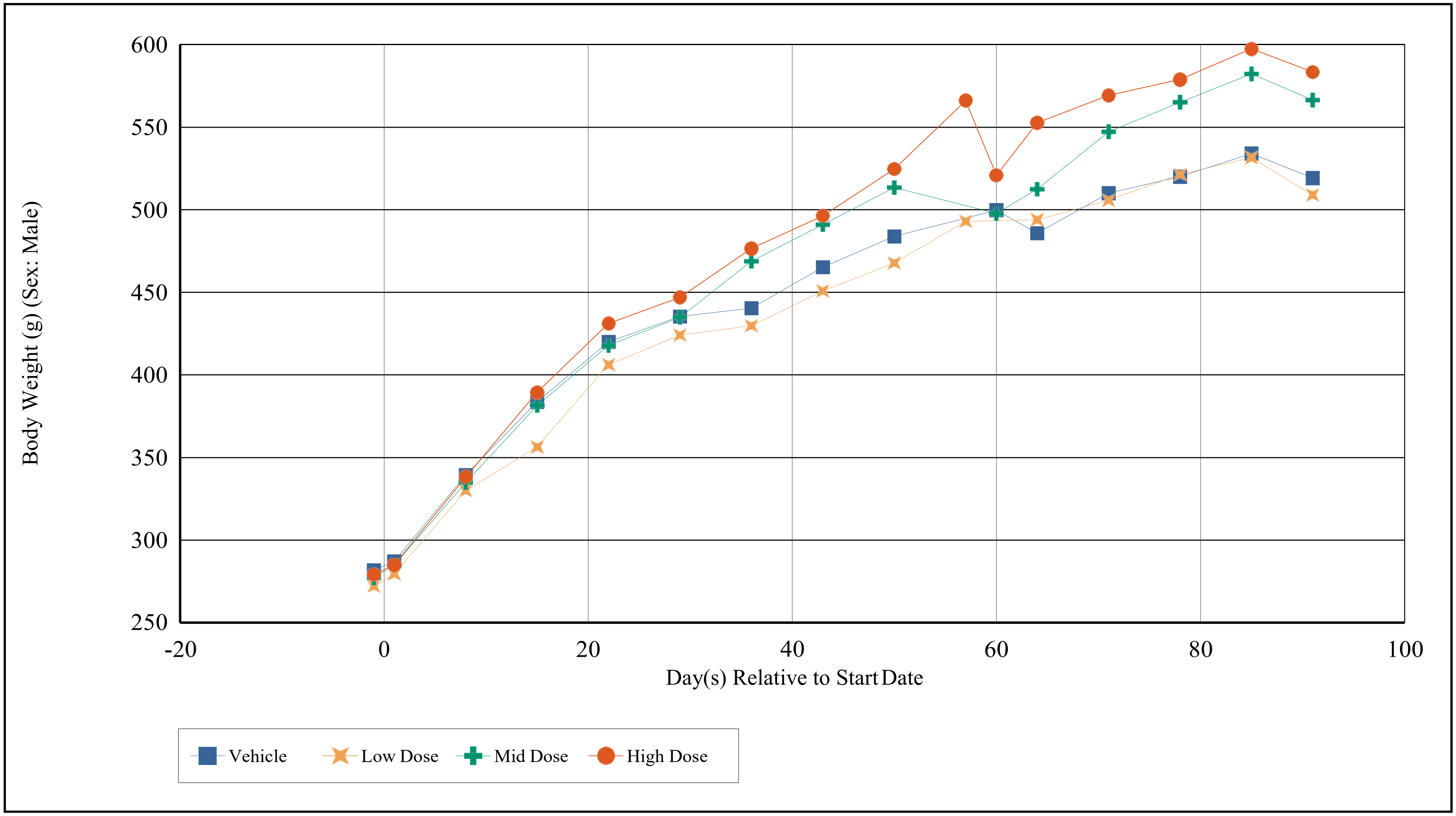

Day 8 body weights for several Day 8 Necropsy animals were fasted, necropsy body weights. See Appendix 6 for individual body weights.

2759-001
A Single Dose Toxicity Study of Test Article Administered by Intrathecal Injection in Rats

Mean Body Weight Values

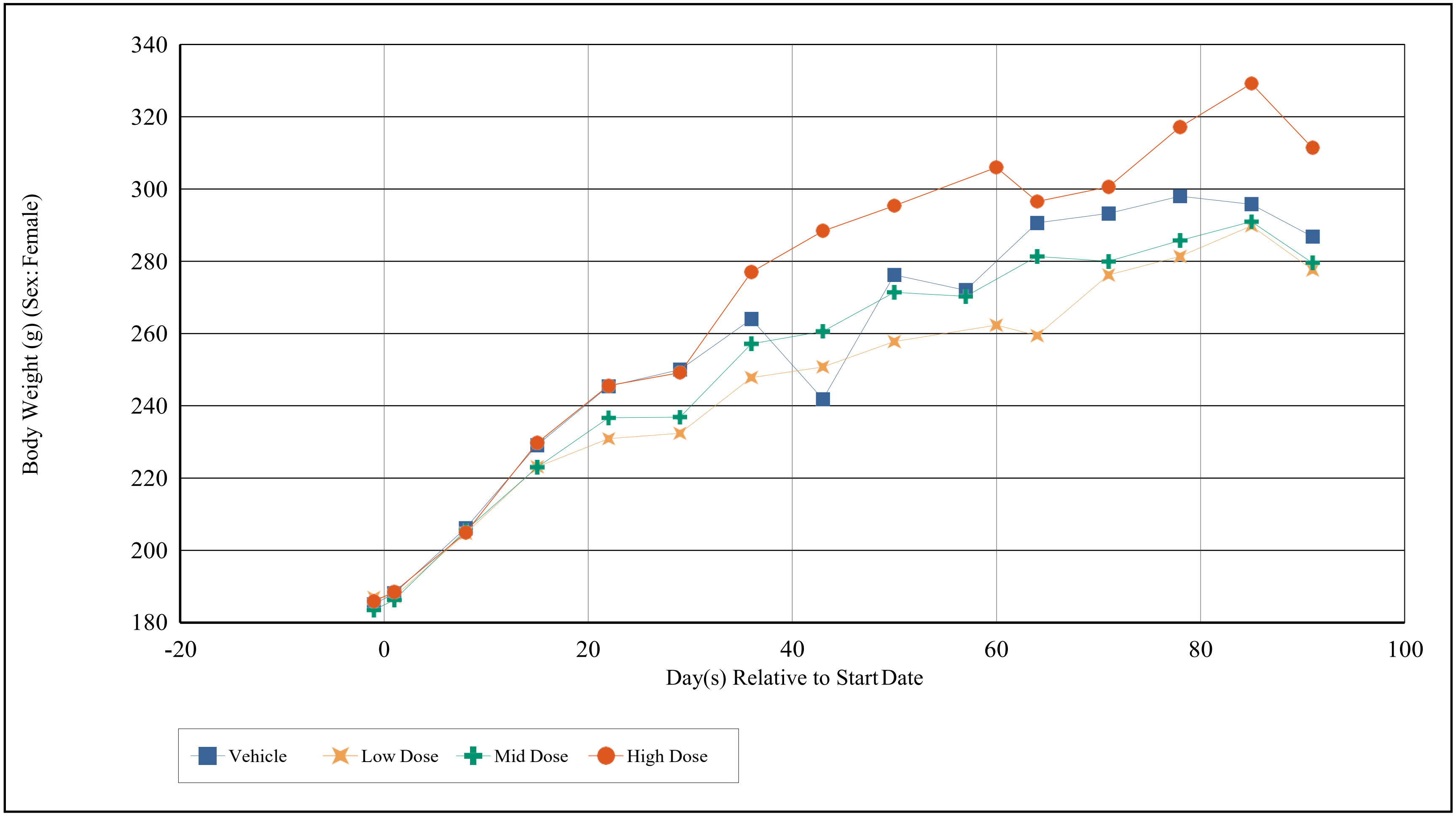

Day 8 body weights for several Day 8 Necropsy animals were fasted, necropsy body weights. See Appendix 6 for individual body weights.

Figure 2
Mean Body Weight Change Values

2759-001
A Single Dose Toxicity Study of Test Article Administered by Intrathecal Injection in Rats

Mean Body Weight Change Values, g

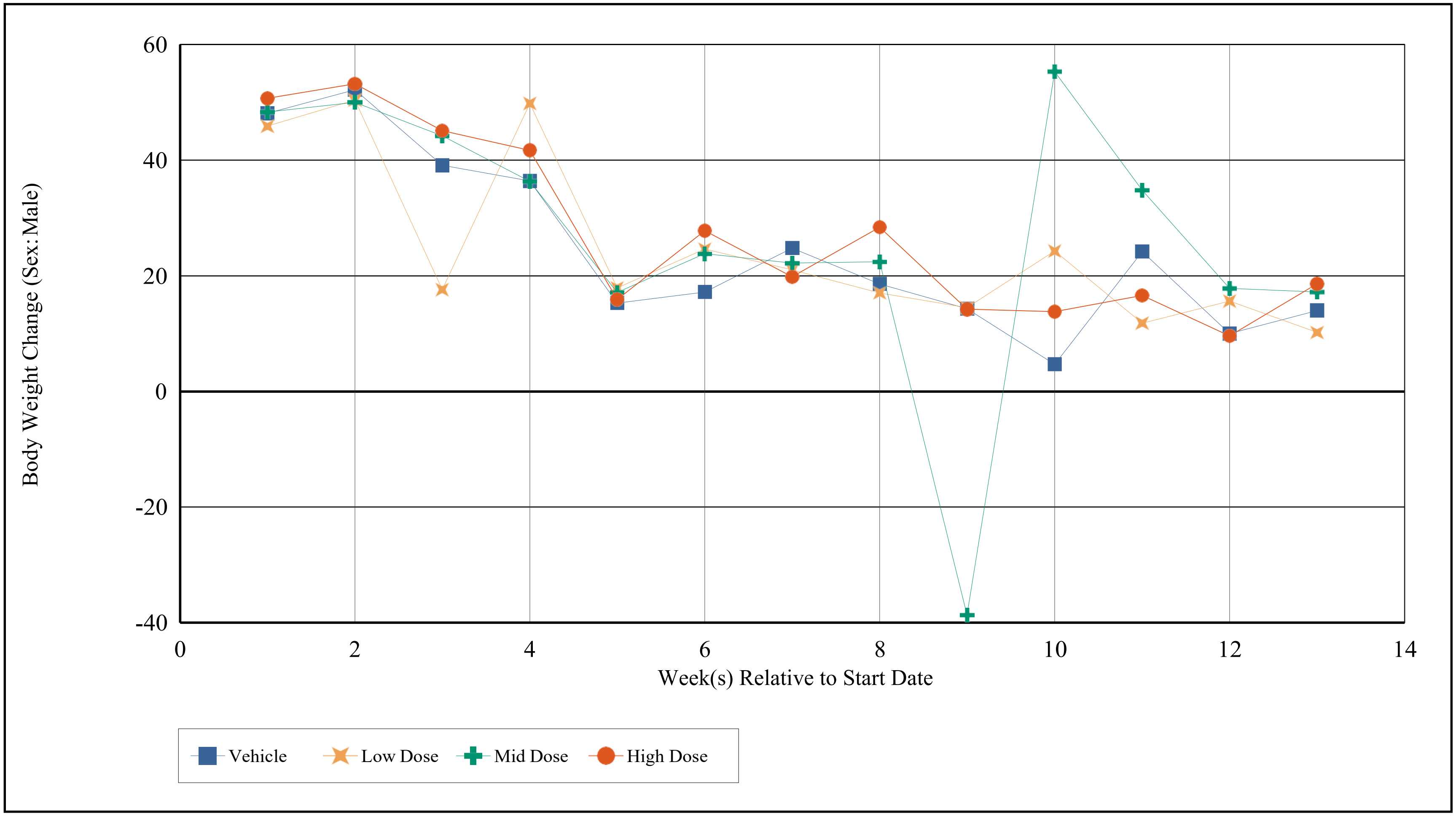

Day 8 body weights for several Day 8 Necropsy animals were fasted, necropsy body weights. See Appendix 6 for individual body weights.

2759-001
A Single Dose Toxicity Study of Test Article Administered by Intrathecal Injection in Rats

Mean Body Weight Change Values, g

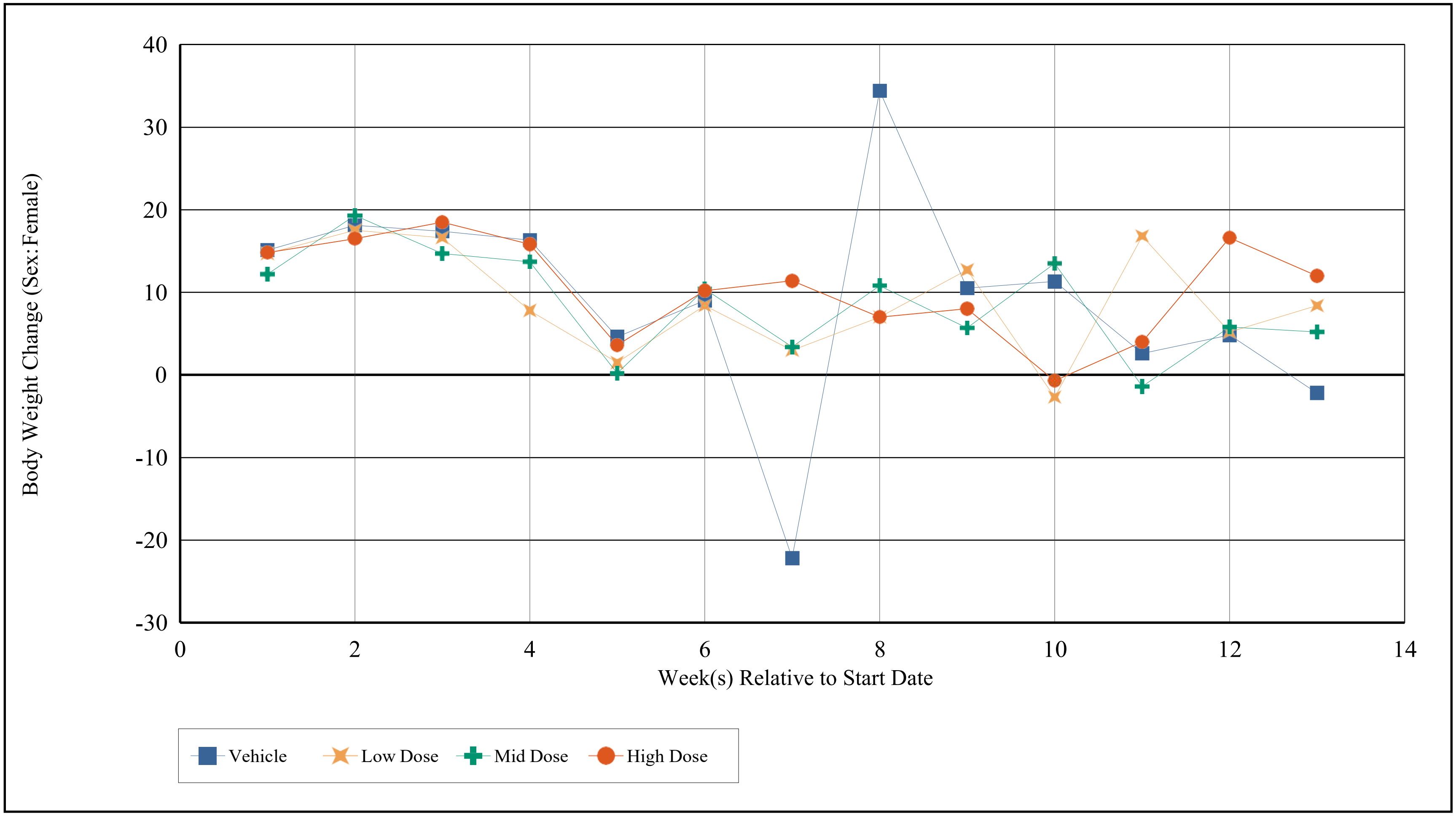

Day 8 body weights for several Day 8 Necropsy animals were fasted, necropsy body weights. See Appendix 6 for individual body weights.

Figure 3
Mean Caged Food Consumption Values

2759-001
A Single Dose Toxicity Study of Test Article Administered by Intrathecal Injection in Rats

Mean Caged Food Consumption Values

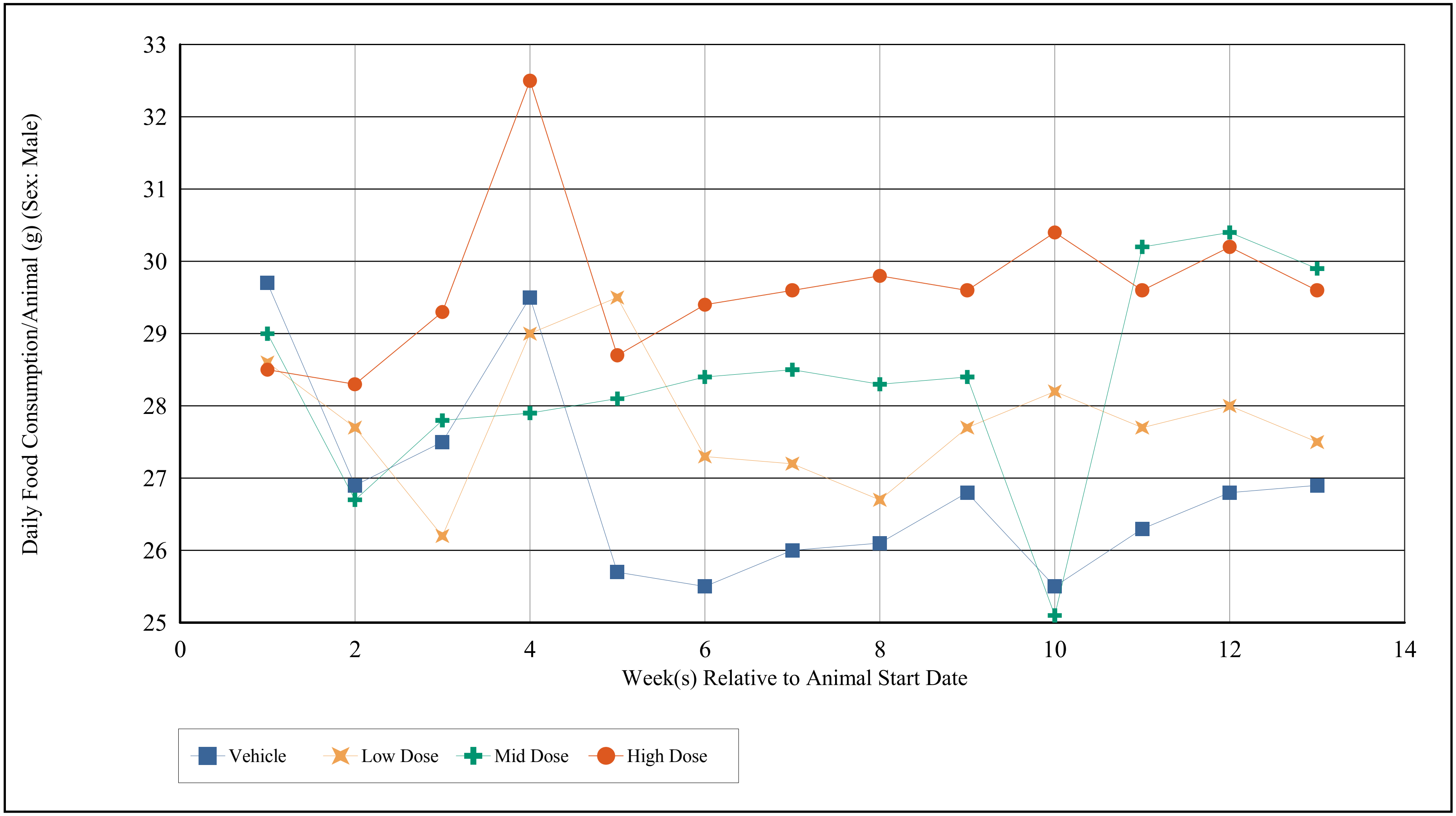

2759-001
A Single Dose Toxicity Study of Test Article Administered by Intrathecal Injection in Rats

Mean Caged Food Consumption Values

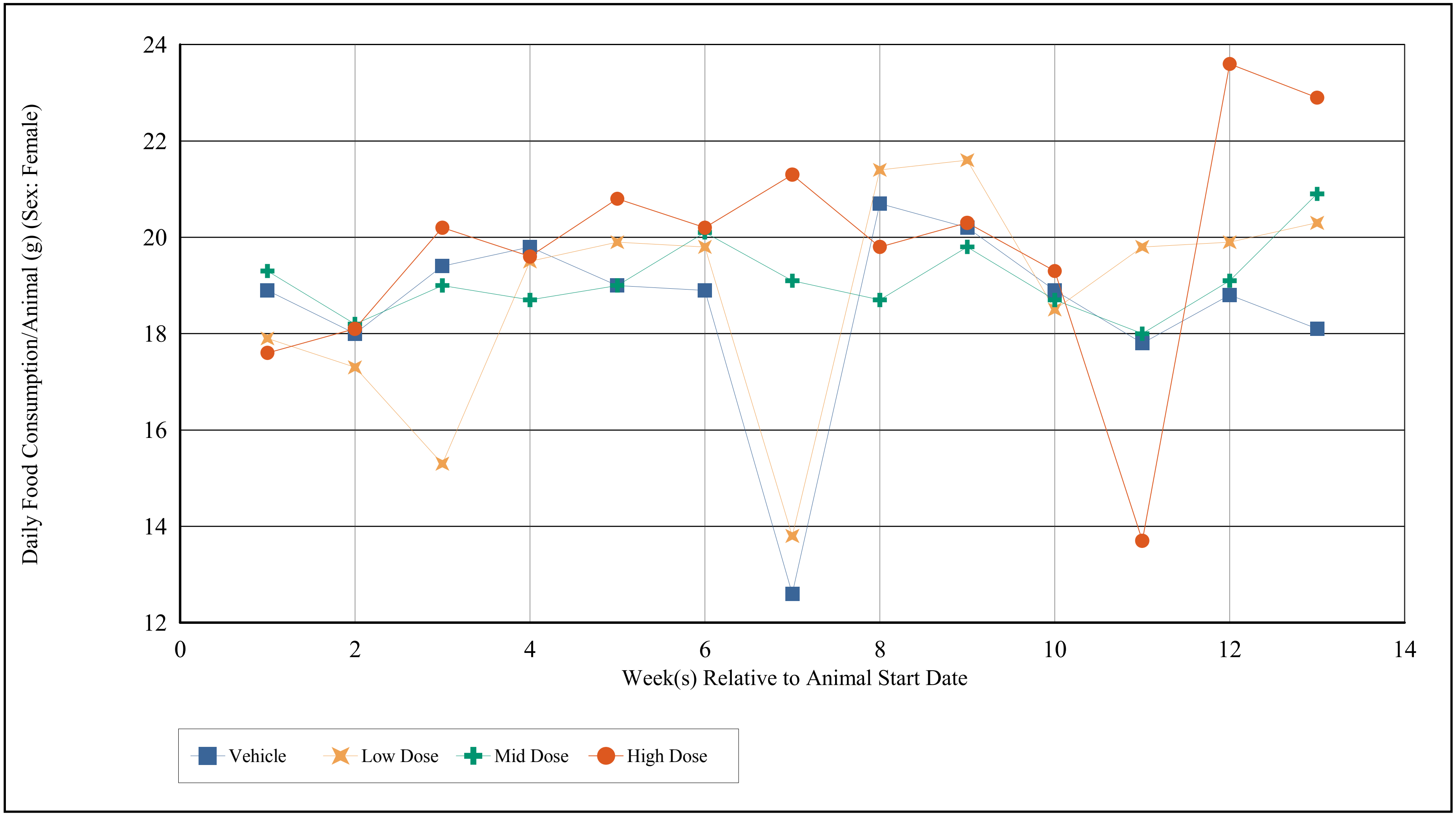

Table 1
Summary of Detailed Clinical Observations

2759-001
A Single Dose Toxicity Study of Test Article Administered by Intrathecal Injection in Rats

Summary of Detailed Clinical Observations

Observation Type: Routine	Male			
From Week -1 (Start Date) to 13 (Start Date)	Vehicle	Low Dose	Mid Dose	High Dose
Total Number of Animals:	15	15	15	15
**Normal**				
Number of Times Recorded	80	92	101	99
Number of Animals Affected	14	15	15	15
**-BEHAVIOR/ACTIVITY**				
**Stereotypy**				
Number of Times Recorded	0	0	0	0
**Vocalization**				
Number of Times Recorded	0	0	0	0
**-EXTERNAL APPEARANCE**				
**Ear/portion of ear missing**				
Number of Times Recorded	0	4	6	8
Number of Animals Affected	-	2	2	1
**Tail bent**				
Number of Times Recorded	25	0	0	0
Number of Animals Affected	2	-	-	-
**-PELAGE/SKIN**				
**Hair discolored, Red**				
Number of Times Recorded	3	0	5	4
Number of Animals Affected	1	-	1	1
**Hair discolored, Brown**				
Number of Times Recorded	6	0	0	4
Number of Animals Affected	1	-	-	1
**Hair sparse**				
Number of Times Recorded	17	17	10	16
Number of Animals Affected	3	4	1	3
**Loss of skin elasticity**				
Number of Times Recorded	0	1	1	0
Number of Animals Affected	-	1	1	-
**Nodule, 5-20 mm**				
Number of Times Recorded	0	0	0	0
**Scabbed area**				
Number of Times Recorded	9	17	10	8
Number of Animals Affected	3	3	1	3

2759-001
A Single Dose Toxicity Study of Test Article Administered by Intrathecal Injection in Rats

Summary of Detailed Clinical Observations

Observation Type: Unscheduled	Male			
From Week -1 (Start Date) to 13 (Start Date)	Vehicle	Low Dose	Mid Dose	High Dose
Total Number of Animals:	15	15	15	15
**-EXTERNAL APPEARANCE**				
**Discharge, Red**				
Number of Times Recorded	0	-	0	-
**Ear/portion of ear missing**				
Number of Times Recorded	0	-	1	-
Number of Animals Affected	-	-	1	-
**Swelling**				
Number of Times Recorded	0	-	0	-
**Tail bent**				
Number of Times Recorded	1	-	0	-
Number of Animals Affected	1	-	-	-
**-PELAGE/SKIN**				
**Hair sparse**				
Number of Times Recorded	2	-	1	-
Number of Animals Affected	2	-	1	-
**Scabbed area**				
Number of Times Recorded	2	-	1	-
Number of Animals Affected	2	-	1	-

2759-001
A Single Dose Toxicity Study of Test Article Administered by Intrathecal Injection in Rats

Summary of Detailed Clinical Observations

Observation Type: Routine	Female			
From Week -1 (Start Date) to 13 (Start Date)	Vehicle	Low Dose	Mid Dose	High Dose
Total Number of Animals:	15	15	15	15
**Normal**				
Number of Times Recorded	109	101	104	108
Number of Animals Affected	15	15	15	15
**-BEHAVIOR/ACTIVITY**				
**Stereotypy**				
Number of Times Recorded	0	0	0	6
Number of Animals Affected	-	-	-	1
**Vocalization**				
Number of Times Recorded	0	0	1	1
Number of Animals Affected	-	-	1	1
**-EXTERNAL APPEARANCE**				
**Ear/portion of ear missing**				
Number of Times Recorded	0	0	0	0
**Tail bent**				
Number of Times Recorded	0	0	0	0
**-PELAGE/SKIN**				
**Hair discolored, Red**				
Number of Times Recorded	14	17	10	7
Number of Animals Affected	1	4	4	3
**Hair discolored, Brown**				
Number of Times Recorded	0	5	0	0
Number of Animals Affected	-	1	-	-
**Hair sparse**				
Number of Times Recorded	2	0	7	0
Number of Animals Affected	1	-	2	-
**Loss of skin elasticity**				
Number of Times Recorded	2	0	0	0
Number of Animals Affected	2	-	-	-
**Nodule, 5-20 mm**				
Number of Times Recorded	0	0	5	0
Number of Animals Affected	-	-	1	-
**Scabbed area**				
Number of Times Recorded	0	0	3	1
Number of Animals Affected	-	-	3	1

2759-001
A Single Dose Toxicity Study of Test Article Administered by Intrathecal Injection in Rats

Summary of Detailed Clinical Observations

Observation Type: Unscheduled	Female			
From Week -1 (Start Date) to 13 (Start Date)	Vehicle	Low Dose	Mid Dose	High Dose
Total Number of Animals:	15	15	15	15
**-EXTERNAL APPEARANCE**				
**Discharge, Red**				
Number of Times Recorded	-	-	1	-
Number of Animals Affected	-	-	1	-
**Ear/portion of ear missing**				
Number of Times Recorded	-	-	0	-
**Swelling**				
Number of Times Recorded	-	-	2	-
Number of Animals Affected	-	-	2	-
**Tail bent**				
Number of Times Recorded	-	-	0	-
**-PELAGE/SKIN**				
**Hair sparse**				
Number of Times Recorded	-	-	0	-
**Scabbed area**				
Number of Times Recorded	-	-	0	-

Table 2
Summary of Body Weight Values

2759-001
A Single Dose Toxicity Study of Test Article Administered by Intrathecal Injection in Rats

Summary of Body Weight Values

Body Weight (g)

Sex: Male<br>Day(s) Relative to Start Date		Vehicle	Low Dose	Mid Dose	High Dose
-1	Mean	281.6	271.9	277.1	279.0
	SD	21.12	25.13	24.90	24.57
	N	15	15	15	15
1	Mean	287.1	279.5	284.8	284.9
	SD	21.33	25.64	24.35	22.82
	N	15	15	15	15
8	Mean	339.3	329.9	334.8	338.1
	SD	24.16	28.76	26.69	30.02
	N	15	15	15	15
15	Mean	383.6	356.4	381.5	389.4
	SD	25.18	59.20	37.07	45.82
	N	10	10	10	10
22	Mean	420.0	406.2	417.8	431.1
	SD	28.96	38.61	42.03	55.36
	N	10	10	10	10

Day 8 body weights for several Day 8 Necropsy animals were fasted, necropsy body weights. See Appendix 6 for individual body weights.

Anova & Dunnett

2759-001
A Single Dose Toxicity Study of Test Article Administered by Intrathecal Injection in Rats

Summary of Body Weight Values

Body Weight (g)

Sex: Male<br>Day(s) Relative to Start Date		Vehicle	Low Dose	Mid Dose	High Dose
29 [g]	Mean	435.3	424.1	434.9	447.0
	SD	19.55	28.93	44.25	62.46
	N	10	10	10	10
36 [g]	Mean	440.4	429.8	468.8	476.4
	SD	22.83	33.03	35.86	53.70
	N	5	5	5	5
43 [g]	Mean	465.2	450.8	491.0	496.2
	SD	17.38	36.18	33.50	54.37
	N	5	5	5	5
50 [g]	Mean	483.8	467.8	513.4	524.6
	SD	20.24	41.92	38.51	61.13
	N	5	5	5	5
57 [I]	Mean	-	493.0 n	-	566.0 n
	SD	-	67.88	-	48.08
	N	-	2	-	2

[g] - Anova & Dunnett
[I] - n - Inappropriate for statistics

2759-001
A Single Dose Toxicity Study of Test Article Administered by Intrathecal Injection in Rats

Summary of Body Weight Values

Body Weight (g)

Sex: Male<br>Day(s) Relative to Start Date		Vehicle	Low Dose	Mid Dose	High Dose
60	Mean	499.7	-	497.7	520.7
	SD	8.08	-	30.09	77.22
	N	3	-	3	3
64	Mean	485.8	494.0	512.4	552.6
	SD	30.68	53.09	61.55	69.58
	N	5	5	5	5
71	Mean	510.0	505.8	547.2	569.2
	SD	19.04	57.14	45.04	75.00
	N	5	5	5	5
78	Mean	520.0	521.4	565.0	578.8
	SD	17.28	60.04	45.95	79.54
	N	5	5	5	5
85	Mean	534.0	531.6	582.2	597.4
	SD	21.41	56.16	45.47	79.09
	N	5	5	5	5

Anova & Dunnett

2759-001
A Single Dose Toxicity Study of Test Article Administered by Intrathecal Injection in Rats

Summary of Body Weight Values

Body Weight (g)

Sex: Male<br>Day(s) Relative to Start Date		Vehicle	Low Dose	Mid Dose	High Dose
91	Mean	519.2	509.0	566.4	583.4
	SD	20.58	61.38	49.44	77.72
	N	5	5	5	5

Anova & Dunnett

2759-001
A Single Dose Toxicity Study of Test Article Administered by Intrathecal Injection in Rats

Summary of Body Weight Values

Body Weight (g)

Sex: Female<br>Day(s) Relative to Start Date		Vehicle	Low Dose	Mid Dose	High Dose
-1	Mean	185.1	186.9	183.5	185.9
	SD	9.74	11.33	9.58	9.15
	N	15	15	15	15
1	Mean	188.1	187.2	186.3	188.5
	SD	11.25	11.18	10.50	8.08
	N	15	15	15	15
8	Mean	206.1	204.7	205.5	204.9
	SD	14.53	12.44	11.22	16.20
	N	15	15	15	15
15	Mean	229.1	223.1	223.0	229.8
	SD	7.65	16.02	14.89	14.66
	N	10	10	10	10
22	Mean	245.4	230.9	236.7	245.6
	SD	11.86	17.62	16.96	20.41
	N	10	10	10	10

Day 8 body weights for several Day 8 Necropsy animals were fasted, necropsy body weights. See Appendix 6 for individual body weights.

Anova & Dunnett

2759-001
A Single Dose Toxicity Study of Test Article Administered by Intrathecal Injection in Rats

Summary of Body Weight Values

Body Weight (g)

Sex: Female<br>Day(s) Relative to Start Date		Vehicle	Low Dose	Mid Dose	High Dose
29 [g]	Mean	250.0	232.4	236.9	249.2
	SD	16.22	16.93	21.66	25.60
	N	10	10	10	10
36 [g]	Mean	264.0	247.8	257.2	277.0
	SD	21.40	14.38	12.11	14.16
	N	5	5	5	5
43 [g1]	Mean	241.8	250.8	260.6	288.4
	SD	48.26	14.53	10.26	13.96
	N	5	5	5	5
50 [g]	Mean	276.2	257.8	271.4	295.4
	SD	21.32	19.36	12.34	16.04
	N	5	5	5	5
57 [I]	Mean	272.0 n	-	270.3 n	-
	SD	43.84	-	3.79	-
	N	2	-	3	-

[g] - Anova & Dunnett
[g1] - Kruskal-Wallis & Dunn
[I] - n - Inappropriate for statistics

2759-001
A Single Dose Toxicity Study of Test Article Administered by Intrathecal Injection in Rats

Summary of Body Weight Values

Body Weight (g)

Sex: Female<br>Day(s) Relative to Start Date		Vehicle	Low Dose	Mid Dose	High Dose
60 [l]	Mean	-	262.3 n	-	306.0 n
	SD	-	20.98	-	14.73
	N	-	3	-	3
64 [g]	Mean	290.6	259.4	281.4	296.6
	SD	25.34	15.03	16.95	19.86
	N	5	5	5	5
71 [g]	Mean	293.2	276.2	280.0	300.6
	SD	30.10	20.02	15.57	15.81
	N	5	5	5	5
78 [g]	Mean	298.0	281.4	285.8	317.2
	SD	29.33	23.16	16.72	20.91
	N	5	5	5	5
85 [g]	Mean	295.8	289.8	291.0	329.2
	SD	32.44	27.63	19.89	21.90
	N	5	5	5	5

[g] - Anova & Dunnett
[l] - n - Inappropriate for statistics

2759-001
A Single Dose Toxicity Study of Test Article Administered by Intrathecal Injection in Rats

Summary of Body Weight Values

Body Weight (g)

Sex: Female<br>Day(s) Relative to Start Date		Vehicle	Low Dose	Mid Dose	High Dose
91	Mean	286.8	277.6	279.6	311.4
	SD	23.55	19.93	17.91	22.07
	N	5	5	5	5

Anova & Dunnett

Table 3
Summary of Body Weight Change Values

2759-001
A Single Dose Toxicity Study of Test Article Administered by Intrathecal Injection in Rats

Summary of Body Weight Change Values, g

Body Weight Change

Sex: Male<br>Day(s) Relative to Start Date		Vehicle	Low Dose	Mid Dose	High Dose
-1 → 1 [g]	Mean	5.5	7.6	7.7	5.9
	SD	4.70	3.48	4.95	5.47
	N	15	15	15	15
1 → 8 [g]	Mean	52.2	50.4	50.0	53.2
	SD	10.07	11.09	9.97	13.29
	N	15	15	15	15
8 → 15 [g1]	Mean	39.1	17.6	44.2	45.1
	SD	5.70	50.20	10.66	13.14
	N	10	10	10	10
15 → 22 [g1]	Mean	36.4	49.8	36.3	41.7
	SD	6.22	33.90	8.65	11.89
	N	10	10	10	10
22 → 29 [g]	Mean	15.3	17.9	17.1	15.9
	SD	14.24	16.75	18.02	15.93
	N	10	10	10	10

Day 8 body weights for several Day 8 Necropsy animals were fasted, necropsy body weights. See Appendix 6 for individual body weights.

[g] - Anova & Dunnett
[g1] - Kruskal-Wallis & Dunn

2759-001
A Single Dose Toxicity Study of Test Article Administered by Intrathecal Injection in Rats

Summary of Body Weight Change Values, g

Body Weight Change

Sex: Male<br>Day(s) Relative to Start Date		Vehicle	Low Dose	Mid Dose	High Dose
29 → 36 [g]	Mean	17.2	24.6	23.8	27.8
	SD	6.72	9.13	5.45	6.91
	N	5	5	5	5
36 → 43 [g]	Mean	24.8	21.0	22.2	19.8
	SD	7.09	7.91	4.32	2.68
	N	5	5	5	5
43 → 50 [g]	Mean	18.6	17.0	22.4	28.4
	SD	6.07	8.37	7.23	13.07
	N	5	5	5	5
50 → 57 [l]	Mean	-	14.5 n	-	19.5 n
	SD	-	4.95	-	3.54
	N	-	2	-	2
50 → 60 [g]	Mean	14.3	-	-38.7 b	10.7
	SD	6.35	-	4.16	11.85
	N	3	-	3	3

[g] - Anova & Dunnett: b = p < 0.01
[l] - n - Inappropriate for statistics

2759-001
A Single Dose Toxicity Study of Test Article Administered by Intrathecal Injection in Rats

Summary of Body Weight Change Values, g

Body Weight Change

Sex: Male<br>Day(s) Relative to Start Date		Vehicle	Low Dose	Mid Dose	High Dose
60 → 64 [g]	Mean	4.7	-	55.3 b	8.0
	SD	3.06	-	1.15	1.73
	N	3	-	3	3
57 → 64 [l]	Mean	-	14.5 n	-	22.5 n
	SD	-	2.12	-	4.95
	N	-	2	-	2
50 → 64 [l]	Mean	-23.5 n	24.3 n	-27.5 n	-
	SD	6.36	22.94	6.36	-
	N	2	3	2	-
64 → 71 [g1]	Mean	24.2	11.8	34.8	16.6
	SD	20.90	7.46	18.59	5.59
	N	5	5	5	5
71 → 78 [g]	Mean	10.0	15.6	17.8	9.6
	SD	5.52	5.98	7.40	7.27
	N	5	5	5	5

[g] - Anova & Dunnett: b = p < 0.01
[l] - n - Inappropriate for statistics
[g1] - Anova & Dunnett(Log)

2759-001
A Single Dose Toxicity Study of Test Article Administered by Intrathecal Injection in Rats

Summary of Body Weight Change Values, g

Body Weight Change

Sex: Male<br>Day(s) Relative to Start Date		Vehicle	Low Dose	Mid Dose	High Dose
78 → 85	Mean	14.0	10.2	17.2	18.6
	SD	5.79	13.79	3.11	5.86
	N	5	5	5	5
85 → 91	Mean	-14.8	-22.6	-15.8	-14.0
	SD	3.96	8.02	6.38	6.67
	N	5	5	5	5

Anova & Dunnett

2759-001
A Single Dose Toxicity Study of Test Article Administered by Intrathecal Injection in Rats

Summary of Body Weight Change Values, g

Body Weight Change

Sex: Female<br>Day(s) Relative to Start Date		Vehicle	Low Dose	Mid Dose	High Dose
-1 → 1 [g]	Mean	3.0	0.3	2.8	2.5
	SD	5.58	2.09	5.39	3.46
	N	15	15	15	15
1 → 8 [g1]	Mean	18.1	17.5	19.3	16.5
	SD	12.53	8.54	9.25	13.55
	N	15	15	15	15
8 → 15 [g1]	Mean	17.4	16.6	14.7	18.5
	SD	6.11	4.12	7.15	5.50
	N	10	10	10	10
15 → 22 [g1]	Mean	16.3	7.8	13.7	15.8
	SD	7.42	6.66	6.36	10.00
	N	10	10	10	10
22 → 29 [g1]	Mean	4.6	1.5	0.2	3.6
	SD	12.39	13.39	9.64	14.89
	N	10	10	10	10

Day 8 body weights for several Day 8 Necropsy animals were fasted, necropsy body weights. See Appendix 6 for individual body weights.

[g] - Kruskal-Wallis & Dunn
[g1] - Anova & Dunnett

2759-001
A Single Dose Toxicity Study of Test Article Administered by Intrathecal Injection in Rats

Summary of Body Weight Change Values, g

Body Weight Change

Sex: Female<br>Day(s) Relative to Start Date		Vehicle	Low Dose	Mid Dose	High Dose
29 → 36 [g]	Mean	9.0	8.4	10.4	10.2
	SD	6.04	8.62	11.84	9.28
	N	5	5	5	5
36 → 43 [g1]	Mean	-22.2	3.0	3.4	11.4
	SD	40.48	7.31	8.17	11.33
	N	5	5	5	5
43 → 50 [g1]	Mean	34.4	7.0	10.8	7.0
	SD	33.92	9.97	8.14	12.77
	N	5	5	5	5
50 → 57 [I]	Mean	10.5 n	-	5.7 n	-
	SD	12.02	-	7.64	-
	N	2	-	3	-
50 → 60 [I]	Mean	-	12.7 n	-	8.0 n
	SD	-	14.43	-	5.57
	N	-	3	-	3

[g] - Anova & Dunnett
[g1] - Kruskal-Wallis & Dunn
[I] - n - Inappropriate for statistics

2759-001
A Single Dose Toxicity Study of Test Article Administered by Intrathecal Injection in Rats

Summary of Body Weight Change Values, g

Body Weight Change

Sex: Female<br>Day(s) Relative to Start Date		Vehicle	Low Dose	Mid Dose	High Dose
60 → 64 [l]	Mean	-	-2.7 n	-	-0.7 n
	SD	-	7.57	-	3.21
	N	-	3	-	3
57 → 64 [l]	Mean	8.5 n	-	2.0 n	-
	SD	2.12	-	8.00	-
	N	2	-	3	-
50 → 64 [l]	Mean	11.3 n	-11.0 n	13.5 n	-8.0 n
	SD	1.15	4.24	4.95	4.24
	N	3	2	2	2
64 → 71 [g]	Mean	2.6	16.8	-1.4	4.0
	SD	11.10	15.11	6.58	5.39
	N	5	5	5	5
71 → 78 [g]	Mean	4.8	5.2	5.8	16.6 a
	SD	4.44	4.71	2.17	11.87
	N	5	5	5	5

[l] - n - Inappropriate for statistics
[g] - Anova & Dunnett: a = p < 0.05

2759-001
A Single Dose Toxicity Study of Test Article Administered by Intrathecal Injection in Rats

Summary of Body Weight Change Values, g

Body Weight Change

Sex: Female<br>Day(s) Relative to Start Date		Vehicle	Low Dose	Mid Dose	High Dose
78 → 85 [g]	Mean	-2.2	8.4	5.2	12.0
	SD	5.17	9.13	4.60	11.98
	N	5	5	5	5
85 → 91 [g1]	Mean	-9.0	-12.2	-11.4	-17.8
	SD	11.85	8.87	3.58	4.44
	N	5	5	5	5

[g] - Anova & Dunnett
[g1] - Kruskal-Wallis & Dunn

Table 4
Summary of Caged Food Consumption Values

2759-001
A Single Dose Toxicity Study of Test Article Administered by Intrathecal Injection in Rats

Summary of Caged Food Consumption Values

Daily Food Consumption/Animal (g)

Sex: Male<br>Week(s) Relative to Animal Start Date		Vehicle	Low Dose	Mid Dose	High Dose
1 [I]	Mean	29.7 n	28.6 n	29.0 n	28.5 n
	SD	0.02	0.73	0.04	1.36
	N	2	2	2	2
1 → 2 [g]	Mean	26.9	27.7	26.7	28.3
	SD	1.98	3.33	0.59	3.37
	N	4	4	4	4
2 → 3 [g]	Mean	27.5	26.2	27.8	29.3
	SD	1.74	8.27	0.80	4.62
	N	4	4	4	4
4 [I]	Mean	29.5 n	29.0 n	27.9 n	32.5 n
	SD	1.47	0.98	1.20	7.37
	N	2	2	2	2
3 → 4 [g]	Mean	27.0	28.6	27.6	30.5
	SD	3.03	4.09	0.75	4.97
	N	4	4	4	4
4 → 5 [I]	Mean	25.7 n	29.5 n	28.1 n	28.7 n
	SD	0.81	1.80	1.30	2.00
	N	2	2	2	2

[I] - n - Inappropriate for statistics
[g] - Anova & Dunnett

2759-001
A Single Dose Toxicity Study of Test Article Administered by Intrathecal Injection in Rats

Summary of Caged Food Consumption Values

Daily Food Consumption/Animal (g)

Sex: Male<br>Week(s) Relative to Animal Start Date		Vehicle	Low Dose	Mid Dose	High Dose
5 → 6	Mean	25.5 n	27.3 n	28.4 n	29.4 n
	SD	1.75	4.26	0.51	3.27
	N	2	2	2	2
6 → 7	Mean	26.0 n	27.2 n	28.5 n	29.6 n
	SD	1.57	3.32	0.91	3.92
	N	2	2	2	2
7 → 8	Mean	26.1 n	26.7 n	28.3 n	29.8 n
	SD	2.22	3.10	1.40	3.27
	N	2	2	2	2
8 → 9	Mean	26.8 n	27.7 n	28.4 n	29.6 n
	SD	1.14	2.74	1.13	4.18
	N	2	2	2	2
9 → 10	Mean	25.5 n	28.2 n	25.1 n	30.4 n
	SD	4.19	3.60	0.25	2.26
	N	2	2	2	2
10 → 11	Mean	26.3 n	27.7 n	30.2 n	29.6 n
	SD	0.89	2.71	1.25	3.96
	N	2	2	2	2

n - Inappropriate for statistics

2759-001
A Single Dose Toxicity Study of Test Article Administered by Intrathecal Injection in Rats

Summary of Caged Food Consumption Values

Daily Food Consumption/Animal (g)

Sex: Male<br>Week(s) Relative to Animal Start Date		Vehicle	Low Dose	Mid Dose	High Dose
11 → 12	Mean	26.8 n	28.0 n	30.4 n	30.2 n
	SD	1.26	2.49	0.10	4.53
	N	2	2	2	2
13	Mean	26.7 n	27.5 n	29.9 n	29.6 n
	SD	2.45	0.19	1.72	3.51
	N	2	2	2	2
12 → 13	Mean	26.9 n	25.3 n	29.8 n	28.9 n
	SD	2.00	0.45	1.48	3.20
	N	2	2	2	2

n - Inappropriate for statistics

2759-001
A Single Dose Toxicity Study of Test Article Administered by Intrathecal Injection in Rats

Summary of Caged Food Consumption Values

Daily Food Consumption/Animal (g)

Sex: Female<br>Week(s) Relative to Animal Start Date		Vehicle	Low Dose	Mid Dose	High Dose
1 [l]	Mean	18.9 n	17.9 n	19.3 n	17.6 n
	SD	0.35	1.85	1.20	1.67
	N	2	2	2	2
1 → 2 [g]	Mean	18.0	17.3	18.2	18.1
	SD	1.14	1.07	0.83	0.76
	N	4	4	4	4
2 → 3 [g1]	Mean	19.4	15.3	19.0	20.2
	SD	0.67	6.39	1.23	0.95
	N	4	4	4	4
4 [l]	Mean	19.8 n	17.2 n	17.8 n	17.5 n
	SD	0.88	1.55	0.75	1.89
	N	2	2	2	2
3 → 4 [g]	Mean	19.5	19.5	18.7	19.6
	SD	1.38	3.46	0.74	0.65
	N	4	4	4	4
4 → 5 [l]	Mean	19.0 n	19.9 n	19.0 n	20.8 n
	SD	0.76	1.43	1.01	0.19
	N	2	2	2	2

[l] - n - Inappropriate for statistics
[g] - Anova & Dunnett
[g1] - Kruskal-Wallis & Dunn

2759-001
A Single Dose Toxicity Study of Test Article Administered by Intrathecal Injection in Rats

Summary of Caged Food Consumption Values

Daily Food Consumption/Animal (g)

Sex: Female<br>Week(s) Relative to Animal Start Date		Vehicle	Low Dose	Mid Dose	High Dose
5 → 6	Mean	18.9 n	19.8 n	20.1 n	20.2 n
	SD	0.12	1.35	0.59	0.13
	N	2	2	2	2
6 → 7	Mean	12.6 n	13.8 n	19.1 n	21.3 n
	SD	8.40	6.97	0.51	2.17
	N	2	2	2	2
7 → 8	Mean	20.7 n	21.4 n	18.7 n	19.8 n
	SD	3.20	3.32	1.01	0.30
	N	2	2	2	2
8 → 9	Mean	20.2 n	21.6 n	19.8 n	20.3 n
	SD	1.78	0.56	0.07	0.62
	N	2	2	2	2
9 → 10	Mean	18.9 n	18.5 n	18.7 n	19.3 n
	SD	0.42	0.79	0.32	0.25
	N	2	2	2	2
10 → 11	Mean	17.8 n	19.8 n	18.0 n	13.7 n
	SD	0.86	1.08	0.57	8.37
	N	2	2	2	2

n - Inappropriate for statistics

2759-001
A Single Dose Toxicity Study of Test Article Administered by Intrathecal Injection in Rats

Summary of Caged Food Consumption Values

Daily Food Consumption/Animal (g)

Sex: Female<br>Week(s) Relative to Animal Start Date		Vehicle	Low Dose	Mid Dose	High Dose
11 → 12	Mean	18.8 n	19.9 n	19.1 n	23.6 n
	SD	0.93	0.54	0.47	6.87
	N	2	2	2	2
13	Mean	18.1 n	19.3 n	20.9 n	20.3 n
	SD	2.00	1.06	0.87	2.97
	N	2	2	2	2
12 → 13	Mean	16.8 n	20.3 n	18.8 n	22.9 n
	SD	0.39	0.76	1.45	4.01
	N	2	2	2	2

n - Inappropriate for statistics

Table 5
Summary of Rotating Rod Values

2759-001
A Single Dose Toxicity Study of Test Article Administered by Intrathecal Injection in Rats

Summary of Rotating Rod Values

Fall Time (sec)

Sex: Male<br><br>Day(s) Relative to Start Date		Vehicle Control	Low Dose	Mid Dose	High Dose
-1 [g]	Mean	43.88	56.40	46.78	52.55
	SD	15.24	22.26	18.96	23.23
	N	10	10	10	10
28 [g]	Mean	62.57	64.40	41.57	48.18
	SD	8.38	10.83	20.03	23.98
	N	5	5	5	5
90 [g]	Mean	28.29	39.60	42.60	42.58
	SD	22.43	19.78	33.92	8.35
	N	5	5	5	5

[g] - Anova & Dunnett

2759-001
A Single Dose Toxicity Study of Test Article Administered by Intrathecal Injection in Rats

Summary of Rotating Rod Values

Fall Time (sec)

Sex: Female<br><br>Day(s) Relative to Start Date		Vehicle Control	Low Dose	Mid Dose	High Dose
-1 [g1]	Mean	81.98	67.04	68.59	72.57
	SD	36.79	12.94	23.91	19.47
	N	10	10	10	10
28 [g]	Mean	64.99	92.88	70.52	82.89
	SD	14.66	37.17	21.19	18.97
	N	5	5	5	5
90 [g]	Mean	62.04	82.22	46.47	76.80
	SD	19.72	27.81	25.80	40.67
	N	5	5	5	5

[g1] - Anova & Dunnett (Log)
[g] - Anova & Dunnett

Appendix 1
Amended Protocol, Protocol, and Deviations

PROTOCOL AMENDMENT NO. 4

Testing Facility Study No. 2759-001

Sponsor Reference No. CLN7-001

A Single Dose Toxicity Study of ~~Test Article~~ scAAV9/JeT-hCLN7opt-SV40pA Administered by Intrathecal Injection in Rats

SPONSOR:
Foundation for Batten Hope
3963 Maple Ave., Suite 390
Dallas, TX 75219
USA

TESTING FACILITY:
Charles River Laboratories, Inc.
54943 North Main Street
Mattawan, MI 49071
USA

SUMMARY OF CHANGES AND JUSTIFICATIONS

Note: When applicable, additions are indicated in bold underlined text and deletions are indicated in bold strikethrough text in the affected sections of the document.

**Item or Section(s)**	**Justification**
**Amendment 1**	**Effective Date of Change: 19 October 2018**
1.2. Proposed Study Schedule	Date updated to match the study schedule.
2.2. Dose Formulation Details	Added BSL2 wording based upon IBC review of the test article.
3.3. Test System Receipt	The number of animals ordered was updated to match the intended number due to the staggered study start. This will provide an appropriate number of spares to meet the study needs.
4.2. Administration	Added BSL2 wording based upon IBC review of the test article.
5.5. Fusion v6 Rotating Rod System	Updated for clarification.
6.1. Animal Disposition	Details added for clarification which were originally omitted in error.
6.2. Postmortem Evaluations	Clarification of the trimming and histology procedures of the spinal cord and DRGs.
6.3. qPCR and RT-qPCR Sample Collection and Analysis	Details added for clarification of the intended collection procedure.
8. STUDY REPORTS	Updated for clarification.
**Amendment 2**	**Effective Date of Change: 10 December 2018**
1.3. Contact Information	Updated to include pathology assignment based upon project scheduling.
6.2. Postmortem Evaluations	Clarification of the trimming and histology procedures of the spinal cord and DRGs. The procedure for trimming was inadvertently not included in amendment 1, however was listed on the summary of changes. Updated pathology evaluation details and added slide shipment due to the pathologist assignment.
**Amendment 3**	**Effective Date of Change: 12 April 2019**
Title page	Updated to reflect legal name change.
1.3. Contact information	Updated email addresses to current CRL emails.
6.4. Organs or Tissues to be Weighed, Preserved, and Microscopically Examined	Histopathological examination of the thymus is added for all animals based upon initial pathology results.
**Amendment 4**	**Effective Date of Change: 25 February 2020**
Study Title	Addition of test article name to study title.

TABLE OF CONTENTS

1. INTRODUCTION 4
2. DOSE FORMULATION AND PREPARATION 7
3. TEST SYSTEM 9
4. STUDY DESIGN 11
5. ANTEMORTEM STUDY EVALUATIONS 12
6. ANIMAL DISPOSITION AND POSTMORTEM STUDY EVALUATIONS 16
7. STATISTICS 25
8. STUDY REPORTS 27
9. DATA AND SPECIMEN RETENTION 28
10. APPROVAL 29

1. INTRODUCTION

1.1. Study Objective

The objective of this study is to further characterize the toxicity, biodistribution, and gene expression of the test article. The test article is being developed for treatment of the neurodegenerative Batten Disease (CLN7 subtype).

1.2. Proposed Study Schedule

The following table contains proposed dates for study activities. Actual dates will be listed in the study report.

Study Initiation Date:	Date Study Director signs Study Approval in this protocol
Experimental Start Date:<br>*(First day of dosing)*	14 September 2018
Experimental Termination Date:<br>*(Last animal removed from study)*	8 January 2019
Draft Report Mail Date:	Approximately 10 weeks after final necropsy

1.3. Contact Information

Study Director:	Sarah Davis, BS, LATG<br>Tel: 269-668-3336 ext. 1250<br>Fax: 269-668-4151<br>E-mail: sarah.davis2@crl.com
Alternate Contact:	Scott T. Wilson, BS, LAT<br>Tel: 269-668-3336 ext. 1610<br>Fax: 269-668-4151<br>E-mail: scott.wilson@crl.com
Sponsor Representative:	Gina Hann<br>Tel: 520-331-6283<br>E-mail: gina.hann@gmail.com
Alternate Sponsor Representative:	Steven Gray, PhD<br>Tel: 214-648-0670

	E-mail: steven.gray@UTSouthwestern.edu
Principal Investigator for Serum, CSF, qPCR, and RT-qPCR Sample Analysis:	Steven J Gray, PhD<br>University of Texas Southwestern Medical Center - Gray Lab<br>6000 Harry Hines Blvd<br>NA02.508<br>Dallas, TX 75390<br>Office: 214-648-0670<br>Lab: 214-648-0671<br>E-mail: steven.gray@UTSouthwestern.edu
Principal Investigator for Anatomic Pathology:	Odete R. Mendes, DVM, PhD, DACVP, DABT<br>Charles River Laboratories, Inc.<br>4025 Stirrup Creek Drive<br>Suite 150<br>Durham, NC 27703<br>Tel: 267-532-3958<br>E-mail: Odete.Mendes@crl.com
Lead Quality Assurance:	Janis Kissel, BS, RQAP-GLP<br>Tel: 269-668-3336, ext. 3170<br>Fax: 269-668-4151<br>E-mail: janis.kissel@crl.com

1.4. Regulatory Compliance, Animal Welfare Standards, and Study Guidelines

Quality Assurance:

- Will assure compliance with the protocol, Testing Facility Standard Operating Procedures (SOPs) and the GLP regulations.
- Will make available inspection records to the Sponsor Representatives during visits to Testing Facility.

GLP Regulation:

- United States Food and Drug Administration (FDA) Good Laboratory Practice (GLP) Regulations, 21 Code of Federal Regulations (CFR) Part 58

Animal Welfare Standards:	• Guide for the Care and Use of Laboratory Animals[1]
Guidelines:	• Current International Council for Harmonisation (ICH) Harmonised Tripartite Guidelines and generally accepted procedures for the testing of pharmaceutical compounds.[2]

1.5. Alteration of Design

Changes to this approved protocol may be made as the study progresses, and all reasonable efforts will be made to discuss necessary changes, in advance, with the Sponsor. Due to the urgent or temporary nature of some protocol changes, the Study Director may authorize a change using a Planned Event form. The form will describe the change, the rationale for the change, and the effective date (or date range, in the instance of a temporary modification) of the change. Such situations will be considered planned deviations until the protocol amendment is prepared and signed, when applicable. In the instance of temporary modifications where a permanent change to the protocol will not be made, the situation will be considered a planned deviation and no protocol amendment will be issued. Resulting protocol amendments will be signed and dated by the Study Director and will identify the date of Sponsor approval, either written or verbal. Signed protocols and protocol amendments will be issued to the Sponsor, including the Sponsor Representative(s), the Sponsor Monitor(s), and the Principal Investigator(s), in addition to other key contact personnel listed in the protocol, and appropriate Testing Facility personnel.

1.6. Proposed Computer Systems

The following are the proposed computer systems to be used during the conduct of this study and their primary function. The actual systems and versions used will be documented in the final report.

Computer System Name	Description
Logbook:	Electronic notebook and data collection system for veterinary communications, observations, and treatments.
Testing Facility ExyLIMS:	A comprehensive laboratory information management system used to manage data, including but not limited to: instrumentation, test articles, standards, and samples.

[1] Guide for the Care and Use of Laboratory Animals. 8th ed., Washington, D.C., National Academies Press, 2011.
[2] Guidance on non-clinical safety studies for the conduct of human clinical trials and marketing authorization for pharmaceuticals. ICH M3(R2), 2009 June 11 (ICH Implementation Date).

Computer System Name	Description
NextDocs®:	Electronic documentation management of Deviation Events and Corrective and Preventative Actions (CAPA).
Provantis™:	Client-server, Oracle-based system used for electronic documentation and data management from compound receipt through reporting.
SAS®:	An integrated system of software products that enables a user to perform data entry, retrieval, data management, reporting, graphics, statistical analysis, and applications development.
Siemens Environmental Monitoring and Niagara Framework® Software System:	Environmental monitoring, alarming, and reporting applications.

2. DOSE FORMULATION AND PREPARATION

2.1. Test Article and Vehicle Information

2.1.1. Test Article Information

Test Article Name: scAAV9/JeT-hCLN7opt-SV40pA

Storage Conditions: Frozen (-60 to -90°C)

Description: A description, lot number, storage conditions, expiration date, safe handling procedures, physical properties, as well as other relevant information will be documented in the study data.

Characterization: The Sponsor will provide documentation on the strength, purity, composition, stability, and other pertinent information on each batch of test article, unless otherwise noted. Note, if the Sponsor does not supply the above information (e.g. certificate of analysis), this will be listed on the Statement of Compliance in the final report.

Reserve Sample: A reserve sample from each lot of test and control articles used in this study will be collected and stored at Testing Facility in a secure area with the appropriate environmental controls. If multiple studies are conducted with the same test article, a common reserve sample may be taken and labeled and stored appropriately.

Test Article Contact: Gina Hann

Tel: 520-331-6283

E-mail: gina.hann@gmail.com

Test Article Disposition: The Sponsor will be contacted for proper disposition of materials (retain/ship/discard) after completion of the in-life phase of the study, and following confirmation that these materials are not assigned to other studies.

2.1.2. Vehicle Information

Vehicle Name: Isotonic phosphate-buffered saline containing 5% w/v sorbitol (provided by the sponsor)

Storage Conditions: The vehicle will be stored frozen (-60 to -90°C) until initial use. Once thawed, the vehicle should be stored refrigerated (2 to 8°C) and used within 2 weeks.

Characterization: The Sponsor will provide documentation on the strength, purity, composition, stability, and other pertinent information on each batch of control article, unless otherwise noted. Note, if the Sponsor does not supply the above information (e.g. certificate of analysis), this will be listed as a GLP exception in the final report.

2.2. Dose Formulation Details

Biosafety Level 2 (BSL2) procedures will be observed during any dose formulations in accordance with Testing Facility BSL2 Policy.

Preparation: Test article will be dispensed as instructed by the Sponsor. The high and middle doses will be used as supplied (without dilution). The low dose will be diluted 1:4 (i.e., 1 part test article and 3 parts vehicle) prior to injection.

Frequency: Dispensed on dosing days

Storage Conditions: Once thawed, the test article should be stored refrigerated (2 to 8°C), released on wet ice for administration, and used within 2 weeks.

3. TEST SYSTEM

3.1. Test System Details

Species:	Rat
Strain:	CD® [Crl:CD®(SD)]
Source:	Charles River Laboratories

3.2. Justification of Test System

The current state of scientific knowledge and the applicable guidelines cited previously in this protocol do not provide acceptable alternatives, *in vitro* or otherwise, to the use of live animals to accomplish the purpose of this study. "The development of knowledge necessary for the improvement of the health and well-being of humans as well as other animals requires *in vivo* experimentation with a wide variety of animal species."[3] "Whole animals are essential in research and testing because they best reflect the dynamic interactions between the various cells, tissues, and organs comprising the human body."[4]

The rat is the usual rodent model[5] used for evaluating the toxicity of various classes of chemicals and for which there is a large historical database.

3.3. Test System Receipt

Expected Age at Arrival:	Ordered to be eight weeks of age at administration
Expected Weight at Arrival:	Commensurate with age; males will generally weigh 215 to 260g and females will generally weigh 165 to 210g, as measured within three days of arrival. The actual range will be documented in the data.

Number Ordered:

Male: 69

Female: 69

Number on Study:

Male: 60

[3] "Principles for the Utilization and Care of Vertebrate Animals Used in Testing, Research, and Training", Federal Register, 1985 May 20; 50(97).
[4] "Position Statement on the Use of Animals in Research", 1993 Feb 26; NIH Guide 22(8).
[5] Guidance for Industry, Investigators, and Reviewers: Exploratory IND Studies, U.S. F.D.A. Center for Drug Evaluation and Research (CDER), 2006 Jan.

Female: 60

Acclimation Duration: At least one week from initial receipt of animals to Testing Facility.

Acclimation Details: During this acclimation period, all animals will be observed daily for any clinical signs of disease, and all animals will be given a detailed clinical examination prior to selection for study.

3.4. Selection for Study

Body Weight Range: ±20% of the mean body weight for each sex

Randomization: By sex, into treatment groups using a standard, by weight, randomization procedure.

All animals with any evidence of disease or physical abnormalities will not be selected for study.

3.5. Method of Identification

Each animal will be assigned an animal number to be used in Provantis™ and will be implanted with a microchip bearing a unique identification number. The individual animal number, implant number, and the Testing Facility study number will comprise a unique identification for each animal. The animal cage will be identified by the study number, animal number, group number, and sex.

3.6. Husbandry

Housing: Pair-housed, when possible (animals may be housed two to three/cage during acclimation and in-life depending on study design)

Caging: Solid bottom cages with nonaromatic bedding. The bedding will be from an approved supplier and documented in the study data.

Temperature and Humidity: Temperature and humidity will be maintained according to Testing Facility SOP.

Lighting: Fluorescent lighting will be provided via an automatic timer for approximately 12 hours per day. On occasion, the dark cycle may be interrupted intermittently due to study-related activities.

Water: Tap water will be supplied *ad libitum* to all animals via an automatic water system unless otherwise indicated.

Diet: The basal diet will be block Lab Diet® Certified Rodent Diet #5002, PMI Nutrition International, Inc. This diet will be available *ad libitum* unless designated otherwise. Each lot number used will be identified in the study records.

Water and Diet Contaminants: There are no known contaminants in the food or water that would interfere with this study. The drinking water used will be monitored for specified contaminants at periodic intervals according to Testing Facility SOP.

Supplemental Enrichment: Animal enrichment will be provided according to Testing Facility SOP.

4. STUDY DESIGN

Group	Treatment	Dose Level (vg/animal)	Dose Volume (µL)	Dose Concentration (vg/µL)	Number of Animals Necropsy Day 8 M	Necropsy Day 8 F	Necropsy Day 29 M	Necropsy Day 29 F	Necropsy Day 91 M	Necropsy Day 91 F
1	Vehicle	0	60 µL	0	5	5	5	5	5	5
2	Test Article Low Dose	$5x10^{11}$	20 µL	$2.5x10^{10}$	5	5	5	5	5	5
3	Test Article Middle Dose	$2x10^{12}$	20 µL	$1x10^{11}$	5	5	5	5	5	5
4	Test Article High Dose	$6x10^{12}$	60 µL	$1x10^{11}$	5	5	5	5	5	5
**Total Number of Animals:**					20	20	20	20	20	20

4.1. Justification of Dose Levels

The dose level was selected by the Sponsor, or in consultation with the Sponsor, on the basis of available data from previous studies and discussion with the FDA in a preIND meeting. The low and middle doses have been tolerated well in pilot mouse studies, and the middle dose is the target dose to model the proposed clinical dose in CLN7 patients. The middle dose has shown sufficient to improve pathological signatures of the disease in mice. Per the discussion with the FDA, we were advised to bracket the “middle” target dose with lower and higher doses to complete the dose-responsive nature of any effect.

4.2. Administration

Biosafety Level 2 (BSL2) procedures will be observed during any test article administration procedures in accordance with Testing Facility BSL2 Policy.

Route:	Intrathecal (IT) injection
Frequency/Duration:	The test article and vehicle will be administered once on Day 1.
Details:	Animals will be anesthetized with Isoflurane and placed in a ventral recumbency and the lumbar cistern will be accessed via a percutaneous needle stick. The location of the needle may be verified using fluoroscopy. Once the needle is in place, the test article will be injected and the needle removed. Animals will then be allowed to recover. The test article will be left at room temperature for 15 to 30 minutes prior to dosing but no more than 2 hours.

4.3. Justification for Route of Administration

Intrathecal injection is the intended route of administration of this test article in humans.

5. ANTEMORTEM STUDY EVALUATIONS

5.1. Cageside Observations

Frequency:	At least twice daily
Details:	Animals will be observed for morbidity, mortality, injury, and availability of food and water. Any animals in poor health will be identified for further monitoring and possible euthanasia.

5.2. Detailed Clinical Observations

Frequency:	Weekly
Details:	Observations will include, but will not be limited to, evaluation of the skin, fur, eyes, ears, nose, oral cavity, thorax, abdomen, external genitalia, limbs and feet, respiratory and circulatory effects, autonomic effects such as salivation, nervous system effects including tremors, convulsions, reactivity to handling, unusual behavior.

5.3. Body Weight

Frequency:	Within three days of arrival, at least once prior to randomization and/or Day -1, and once weekly thereafter.

5.4. Food Consumption

Frequency:	Weekly

5.5. Fusion v6 Rotating Rod System

Frequency:	Animals scheduled for Day 29 and Day 91 necropsy only: predose and prior to their respective necropsy.
Details:	The apparatus is set to accelerate from -4 rpm to -40 rpm (the negative sign indicates direction, in this case it means the animal will ambulate away from the observer) over the course of 3 minutes. This indicates that the rod will be accelerating at a rate of 0.2 revolutions per second. The elapsed time from the start of the trial to the animal's fall and the speed of the rod at the time of the fall is recorded digitally by the instrument. Each animal will perform 3 consecutive trials with at least 15 minutes between trials. The average of all 3 trials will be reported for each animal.<br><br>The morning of each testing a training session will be conducted. The training session will consist of the animal being placed on a rotating rod which is set to a constant speed of -4 rpm for 3 minutes. Animals that fall off during training are immediately placed back on the rotating rod until the 3 minutes is completed. Data from the training sessions will not be reported.<br><br>The computer software is not validated and this will be listed as a GLP exception in the final report.

5.6. Clinical Pathology Sample Collection and Analysis

Fasting Requirements:	Free access to drinking water but fasted overnight prior to blood collection.	
Blood Sample Volume:	Hematology:	0.5 to 1mL
	Coagulation:	1.2mL
	Clinical Chemistry:	0.8 to 1.3mL
Site of Blood Sample Collection:	Vena cava after carbon dioxide inhalation	
Anticoagulant:	Hematology:	K_2EDTA
	Coagulation:	Sodium Citrate
	Clinical Chemistry:	Serum Separator
Sample Disposition:	All samples will be disposed of per Testing Facility SOP.	

The following clinical pathology tests will be conducted on available samples and as survival allows.

Sample Type	Number of Animals	Intervals	Parameters Evaluated
**Hematology**	All	Termination	• leukocyte count (total and absolute differential)<br>• erythrocyte count<br>• hemoglobin<br>• hematocrit<br>• mean corpuscular hemoglobin, mean corpuscular volume, mean corpuscular hemoglobin concentration (calculated)<br>• absolute reticulocytes<br>• platelet count<br>• RDW<br>• blood smear preserve and stain*<br><br>*blood smear review may be performed on select animals per Testing Facility SOP.
**Coagulation**	All	Termination	• prothrombin time<br>• activated partial thromboplastin time<br>• fibrinogen
**Clinical Chemistry**	All	Termination	• alkaline phosphatase<br>• total bilirubin (with direct bilirubin if total bilirubin exceeds 1 mg/dL)<br>• aspartate aminotransferase<br>• alanine aminotransferase<br>• urea nitrogen<br>• creatinine<br>• total protein<br>• albumin<br>• globulin and A/G (albumin/globulin) ratio (calculated)<br>• glucose<br>• total cholesterol<br>• triglycerides<br>• electrolytes (sodium, potassium, chloride)<br>• calcium<br>• phosphorus<br>• sample quality

5.7. Serum Sample Collection and Analysis

5.7.1. Sample Collection

Group(s)	Animals/Sex/Interval	Interval
All	All	Termination

Fasting Requirements:	The animals will not be fasted before blood collection unless concurrent with fasting for other procedures.
Collection Site:	Vena cava after carbon dioxide inhalation
Whole Blood Volume/Sample:	Blood: 0.6 to 1 mL
Anticoagulant:	Serum separator
Whole Blood Storage:	Room Temperature
Additional Container Label Requirements:	Testing Facility study number, relative study day, animal number, and the date and time interval of collection.
Sample Requirements:	Serum: 3 aliquots, 100 µL each. If the total volume of serum is less than 300 µL, then lower equal aliquots are acceptable.
Special Requirements:	The serum may be initially stored on dry ice.
Final Storage Temperature:	Processed samples will be placed on dry ice or in frozen (-60 to -90°C) storage within 60 minutes of centrifugation.

5.7.2. Sample Handling and Analysis

5.7.2.1. Sample Handling

Shipping Conditions:	Frozen on dry ice
Primary Contact for Sample Shipment:	Erik Lykken<br>Attn: Gray Lab<br>6000 Harry Hines Blvd<br>NA02.508<br>University of Texas Southwestern Medical Center<br>Dallas, TX 75390<br>Tel: 214-648-0671<br>E-mail: Erik.Lykken@UTSouthwestern.edu

Shipping Details: Samples will be shipped on Monday through Wednesday for next day delivery. The primary contact will be notified prior to each shipment.

5.7.2.2. Serum Analysis

Analysis Performed by: The analytical work will be conducted by a Sponsor-contracted laboratory, the Laboratory of Dr. Steven Gray, using an analytical method to be added by amendment if sample analysis is conducted. Samples may be retained for possible future analysis.

Regulatory Requirements: The work performed in conjunction with this study will not be conducted in compliance with GLPs. A final report will be prepared and submitted to the Sponsor and Test Facility for inclusion as an appendix in the main study final report., if sample analysis is conducted.

Sample Disposition: Following analysis, any residual samples or backup samples will be retained by the Test Facility (The Laboratory of Dr. Steven Gray) as per their respective SOPs. Any unshipped back up samples remaining at Testing Facility will be archived by Testing Facility.

6. ANIMAL DISPOSITION AND POSTMORTEM STUDY EVALUATIONS

6.1. Animal Disposition

Moribundity: Moribund animals will be subject to Testing Facility SOP criteria and procedures. If possible, the samples below will be collected from animals euthanized *in extremis*, following veterinary consultation. A veterinary consultation is not required if the samples are collected following anesthesia or euthanasia. Blood collection methods utilized for animals euthanized *in extremis* may include suitable methods other than those presented in the respective blood collection section(s) of this protocol.

Sample Type	Groups	Volume	Anticoagulant
Hematology	All	1mL	K_2EDTA
Coagulation		1.2mL	Citrate
Clinical Chemistry		1.3mL	Serum Separator
Serum	All	0.6 to 1 mL	Serum Separator
CSF	All	Max attainable	None

Necropsy examinations will be performed seven days a week. Animals that are found dead or euthanized *in extremis* after regular working hours will be refrigerated overnight and necropsies performed at the start of the next day. Any animal found dead or euthanized *in extremis* will undergo

a gross necropsy examination and macroscopic observations will be recorded. Protocol designated tissues will be saved from these animals.

Method of Euthanasia: Euthanasia will be by carbon dioxide inhalation followed by an Testing Facility SOP approved method to ensure death, e.g. exsanguination.

Disposition of Animals Not Assigned: Extra animals obtained for this study, but not placed on study, will be either transferred to a Testing Facility stock or training colony, or euthanized and discarded. The final disposition of each animal will be documented in the study records.

Disposition of Study Animals: All main study animals will be euthanized and subjected to a complete necropsy examination, performed under procedures approved by a veterinary pathologist in accordance with Testing Facility SOP.

6.2. Postmortem Evaluations

Necropsy Schedule: Terminal: Day 8, Day 29, and Day 91

External Evaluations: The animals will be examined carefully for external abnormalities including palpable masses.

Macroscopic Evaluations: The skin will be reflected from a ventral midline incision, and any subcutaneous masses will be identified and correlated with antemortem findings. The abdominal, thoracic, and cranial cavities will be examined for abnormalities and the organs removed, examined, and, where required, placed in the appropriate fixative.

Tissue Fixation: All tissues will be placed in a neutral buffered formalin, except that the eyes, including the optic nerves, and testes will be fixed using a modified Davidson's fixative[6]. Eyes and testes will be placed into formalin after fixation. Formalin will be infused into the lung via the trachea and into the urinary bladder.

Organ Weights: Body weight and the organ weights identified in the organ list table will be recorded for all animals at the scheduled necropsies, and appropriate organ weight ratios will be calculated (relative to body and brain weights). Paired organs will be weighed together. Organs will not be weighed for animals found dead or euthanized *in extremis*.

[6] Latendresse JR, Warbrittion AR, Jonassen H, Creasy DM. Fixation of testes and eyes using a modified Davidson's fluid: comparison with Bouin's fluid and conventional Davidson's fluid. Toxicol Pathol. 2002 Jul Aug;30(4):524 33.

A combined weight of the thyroid gland with parathyroid glands will be obtained, and the right mandibular/sublingual salivary glands will be weighed together. The thyroid/parathyroid gland and pituitary gland will be weighed following fixation.

Microscopic Evaluation:	Microscopic examination of fixed hematoxylin and eosin-stained paraffin sections will be performed on sections of tissues and from the groups and/or intervals identified in the following table and all animals found dead or euthanized *in extremis*.
Handling of Specific Tissues:	Bone marrow smears will not be prepared for animals found dead but will be prepared for animals euthanized *in extremis* (with the exception of euthanized animals requiring refrigeration prior to necropsy).<br>The spinal cord and DRG (cervical, lumbar, thoracic) will be collected in situ. The samples will be decalcified and embedded in paraffin with cross-sections that include the spinal cord, nerve roots, and DRG.<br>At the time of trimming, the spinal cord will be opened up to remove bilateral DRGs (to include nerve roots, when possible) from the cervical, thoracic, and lumbar regions of the spinal column. The DRGs will each be submitted for processing in individual tissue cassettes, and left/right orientation will be maintained. Spinal cord sections adjacent to the harvested DRGs will be trimmed and submitted for processing.
Additional Testing:	Evaluation of the bone marrow smears will be performed at the discretion of the Study Director and/or Sponsor.<br>The pathologist may use special stains and techniques as needed to aid in the diagnosis of specific lesions. If after routine sectioning, a tissue is missed, one recut will be requested either by re-sectioning the tissue in the block or ordering a wet tissue recut. If the tissue is still missing, the block will not be re-sectioned unless the missing tissue is determined to be a potential target organ. In this case, the tissue will be re-sectioned until located or until it is determined that it is not present in the block or in wet tissue. Non-protocol-required tissues that are coincidentally within the plane of a protocol-required tissue will be examined by the study pathologist, and any findings that impact the interpretation of the study will be documented. In some instances, tissues may be collected and processed to paraffin block or glass slide stage prior to defining the tissues required for microscopic examination (e.g., intermediate and/or recovery groups). In these cases, only protocol-required tissues will be examined microscopically and other tissues present on the slide will not be examined.

Digital images for illustrative purposes may be taken. These images will not be used for data generation or interpretation, and will not be archived or included in the final report.

Pathology Evaluation: All required slides will be shipped under ambient conditions to Charles River Laboratories, Inc., for microscopic evaluation.

Primary Contact for Sample Shipment
Odete R. Mendes, DVM, PhD, DACVP, DABT<br>Charles River Laboratories, Inc.<br>4025 Stirrup Creek Drive<br>Suite 150<br>Durham, NC 27703<br>Tel: 267-532-3958<br>E-mail: Odete.Mendes@crl.com

Any handling of slides performed by Charles River Laboratories, Inc. in conjunction with this study will be conducted in compliance with GLPs, in accordance with Charles River Laboratories, Inc. SOP's.

Microscopic findings will be directly entered into the Testing Facility's Provantis database via remote access and will be performed in accordance with Testing Facility SOP's. Testing Facility will provide all necessary tables to the Anatomic Pathology Principal Investigator for interpretation of the macroscopic and organ weight findings. The microscopic tables will be generated by the Test Site in the Testing Facility's Provantis database via remote access. The Pathology Principal Investigator Report will be prepared by the Test Site and audited by the Test Site Quality Assurance Unit.

All slides, all other study related materials, and data will be sent to Testing Facility for archival when the request to finalize the Pathology Principal Investigator Report is received.

6.3. qPCR and RT-qPCR Sample Collection and Analysis

qPCR Sample Collection: Each organ/tissue sample will be divided in half; one part used for DNA purification and the other part used for RNA purification.

When possible, duplicate samples will be collected (4 total per tissue/organ) as long as each sample is ≥10 mg.

Samples of the organs or tissues collected for qPCR will be placed in labeled 2-mL microfuge tubes (one tube/sample), snap-frozen into liquid nitrogen, and then stored at -60°C to -90°C until shipped to the Sponsor designated laboratory for analysis. Samples collected for RT-qPCR analysis of the transgene mRNA will be completely immersed into labeled 2 mL microfuge tubes prefilled with 1.5 mL RNALater. Samples will be stored refrigerated (2 to 8ºC) for up to 24 hours and then stored frozen (-60 to -90ºC) with the RNALater removed until shipped to the Sponsor designated laboratory for analysis.

The samples for DNA analysis will be collected using strict aseptic techniques and disposable instruments for each tissue or organ. For paired organs, one complete organ will be saved for microscopic examination and a portion of the second organ or the entire organ will be collected for qPCR and RT-qPCR. Care will be given to ensure that cross contamination between tissues does not occur. Gloves will be changed between collection and dissection of each tissue for PCR analysis. In addition, non-disposable instruments will be wiped down with a 10% bleach solution, rinsed with water followed by a wipe down of 100% ethanol between groups. The cutting board will be wiped down with a 10% bleach solution, rinsed with water followed by a wipe down of 100% ethanol between each of the specified organs or sterile trays may be used. The tissues for PCR analysis will be collected prior to sample collection for any other parameters. Samples for qPCR and RT-qPCR will not collected for animals found dead or euthanized *in extremis*.

<u>qPCR and RT-qPCR Sample analysis:</u> If deemed necessary by the sponsor, qPCR and/or RT-qPCR analysis will be conducted. Purified DNA will be used as template for qPCR to detect the transgene in each sample relative to a genomic DNA reference, with a sensitivity of ≤50 copies of vector DNA per microgram of rat genomic DNA. Purified RNA will be used for cDNA synthesis, and the cDNA of the optimized human CLN7 will be quantified relative to endogenous rat GAPDH cDNA and rat CLN7 cDNA levels. Only tissues found positive for vector presence at ≥ 50 double-stranded copy of vector DNA per 1 μg rat DNA will be tested for gene expression by RNA analysis.

6.3.1. Sample Handling and Analysis

6.3.1.1. Sample Handling

Shipping Conditions: Frozen on dry ice

Primary Contact for Sample Shipment:

Erik Lykken
Attn: Gray Lab
6000 Harry Hines Blvd
NA02.508
University of Texas Southwestern Medical Center
Dallas, TX 75390
Tel: 214-648-0671
E-mail: Erik.Lykken@UTSouthwestern.edu

Shipping Details: Samples will be shipped on Monday through Wednesday for next day delivery. The primary contact will be notified prior to each shipment.

6.3.1.2. qPCR and RT-qPCR Sample Analysis

Analysis Performed by: If needed, the analytical work will be conducted by a Sponsor-contracted laboratory, the Laboratory of Dr. Steven Gray, using an analytical method to be added by amendment if sample analysis is conducted. Samples may be retained for possible future analysis.

Regulatory Requirements: The work performed in conjunction with this study will not be conducted in compliance with GLPs. A final report will be prepared and submitted to the Sponsor and Test Facility for inclusion as an appendix in the main study final report, if sample analysis is conducted.

Sample Disposition: Following analysis, any residual samples or backup samples will be retained by the Test Facility (The Laboratory of Dr. Steven Gray) as per their respective SOPs. Any unshipped back up samples remaining at Testing Facility will be archived by Testing Facility.

6.4. Organs or Tissues to be Weighed, Preserved, and Microscopically Examined

Organ	Weigh	Collect at Necropsy	Histology Processing	Histopathology Evaluation Groups[2] All Groups	Biodistribution/Gene Expression All Groups
Animal ID	-	X	-	-	
Artery, aorta	-	X	-	-	
Body cavity, nasal	-	X	-	-	

Organ	Weigh	Collect at Necropsy	Histology Processing	Histopathology Evaluation Groups[2] All Groups	Biodistribution/Gene Expression All Groups
Bone marrow	-	X	-	-	
Bone marrow smear	-	X[1]	-	-	
Bone, femur (one)	-	X	-	-	
Bone, sternum	-	X	-	-	
Brain- at least 3 coronal cross sections (hindbrain including brainstem and cerebellum, midbrain, and forebrain)	X	X	X	X	X
Cervix	-	X	-	-	
Epididymis (both)	X	X	-	-	
Esophagus	-	X	-	-	
Eye (both)	-	X	-	-	
Gland, adrenal (both)	X	X	-	-	
Gland, clitoral (both)	-	X	-	-	
Gland, lacrimal (both, extra-orbital)	-	X	-	-	
Gland, Harderian	-	X (both)	-	-	
Gland, mammary	-	X	-	-	
Gland, parathyroid (both)	-	X	-	-	
Gland, pituitary	X	X	-	-	
Gland, preputial (both)	-	X	-	-	
Gland, prostate	X	X	-	-	
Gland, salivary	X (right mandibular/ sublingual salivary glands)	X (both) sub-mandibular, sublingual, parotid	-	-	
Gland, seminal vesicle (both)	-	X	-	-	
Gland, thyroid (both)	X	X	-	-	
Gland, Zymbal’s (both)	-	X	-	-	
Gut-associated lymphoid tissue	-	X	-	-	
Heart	X	X	X	X	X
Joint, femorotibial (one)	-	X	-	-	-
Kidney (both)	X	X	X	X	X
Large intestine, cecum	-	X	-	-	-
Large intestine, colon	-	X	-	-	-
Large intestine, rectum	-	X	-	-	-
Larynx	-	X	-	-	-
Liver	X	X	X	X	X
Lung	X	X	X	X	X
Lymph node, mandibular	-	X (both)	X (one)	X (one)	X
Lymph node, mesenteric	-	X	X	X	X

Organ	Weigh	Collect at Necropsy	Histology Processing	Histopathology Evaluation Groups[2] All Groups	Biodistribution/Gene Expression All Groups
Macroscopic abnormalities	-	X	X	X	-
Muscle, skeletal (bicep femoris and gastrocnemius)	-	X	X	X	X
Nasopharynx	-	X	-	-	-
Nerve, optic (both)	-	X	-	-	-
Nerve, sciatic	-	X (both)	-	-	-
Ovary (both)	X	X	X	X	X
Oviduct (both)	X	X	-	-	-
Pancreas	-	X	-	-	-
Skin	-	X	-	-	-
Small intestine, duodenum	-	X	-	-	-
Small intestine, ileum	-	X	-	-	-
Small intestine, jejunum	-	X	-	-	-
Spinal cord, cervical (to include DRG and nerve roots)	-	X	X	X	X
Spinal cord, thoracic (to include DRG and nerve roots)	-	X	X	X	X
Spinal cord, lumbar (to include DRG and nerve roots)	-	X	X	X	X
Spleen	X	X	X	X	-
Stomach	-	X	-	-	-
Testis (both)	X	X	X	X	X
Thymus	X	X	X	X	-
Tongue	-	X	-	-	-
Trachea	-	X	-	-	-
Ureter (both)	-	X	-	-	-
Urinary bladder	-	X	-	-	-
Uterus	X	X	-	-	-
Vagina	-	X	-	-	-

X = Procedure to be conducted; - = Not applicable.

[1] Two bone marrow smears will be collected from the femur at scheduled and unscheduled necropsies (for possible examination). Smears will not be collected from animals that are found dead or from animals that were euthanized moribund and then stored in the refrigerator prior to necropsy. Bone marrow smears are allowed to air dry and are not fixed in formalin.

[2] At the discretion of the Study Pathologist, findings for extraneous tissues (non-protocol tissues that may be present on a slide as a result of collection of protocol tissues) will also be recorded when observed.

6.5. Cerebrospinal Fluid (CSF) Sample Collection and Analysis

6.5.1. Sample Collection

Frequency:	All animals at termination and animals euthanized *in extremis*
CSF Volume/Sample:	Max attainable
Anticoagulant:	None
Additional Container Label Requirements:	Testing Facility study number, relative study day, animal number, and the date and time interval of collection.
Sample Requirements:	2 approximately equal aliquots
Final Storage Temperature:	Frozen (-60 to -90°C)

6.5.2. Sample Handling and Analysis

6.5.2.1. Sample Handling

Shipping Conditions:	Frozen on dry ice
Primary Contact for Sample Shipment:	Erik Lykken, PhD<br>Attn: Gray Lab<br>6000 Harry Hines Blvd<br>NA02.508<br>University of Texas Southwestern Medical Center<br>Dallas, TX 75390<br>Tel: 214-648-0671<br>E-mail: Erik.Lykken@UTSouthwestern.edu
Shipping Details:	Samples will be shipped on Monday through Wednesday for next day delivery. The primary contact will be notified prior to each shipment.

6.5.2.2. CSF Analysis

Analysis Performed by:	If needed, the analytical work will be conducted by a Sponsor-contracted laboratory, the Laboratory of Dr. Steven Gray, using an analytical method to be added by amendment if sample analysis is conducted. Samples may be retained for possible future analysis.

Regulatory Requirements: The work performed in conjunction with this study will not be conducted in compliance with GLPs. A final report will be prepared and submitted to the Sponsor and Test Facility for inclusion as an appendix in the main study final report, if sample analysis is conducted.

Sample Disposition: Following analysis, any residual samples or backup samples will be retained by the Test Facility (The Laboratory of Dr. Steven Gray) as per their respective SOPs. Any unshipped back up samples remaining at Testing Facility will be archived by Testing Facility.

7. STATISTICS

The following presents a proposed statistical analysis plan. Statistical plans are data dependent, and this analysis plan may require modification if standard data assumptions are not met. Other conceptually equivalent statistical testing routines may also be employed at the discretion of the statistician. The actual analysis plan will be documented in the final report.

The raw data will be tabulated within each time interval, and the appropriate summary statistics will be calculated for each endpoint, sex, and group. For each endpoint, treatment groups will be compared to the control group using the analysis outlined below. Data for some endpoints, as indicated, will be transformed by either a log or rank transformation prior to conducting the specified analysis.

7.1. Statistical Comparisons

Control Group	Comparison Group(s)
1	2, 3, 4

7.2. Group Pair-wise Comparison (General ANOVA)

Endpoints:

- Body Weight and Body Weight Change
- Food Consumption
- Hematology
- Coagulation
- Clinical Chemistry
- Organ Weights
  - Absolute Weights
  - Relative to Body and Brain Weights
- Urinalysis
  - Urine Volume
  - Specific Gravity
  - pH

Description: The experimental unit for the analysis of food consumption will be cage, while for all other endpoints the experimental unit will be the individual

animal. Food consumption will be calculated as described in Testing Facility SOP.

If the control group has a sample size less than three, no inferential statistics will be calculated. If a particular endpoint and/or parameter within a given collection interval have the same value across all experimental units, no inferential statistics will be calculated.

Otherwise, for endpoints and/or parameters where all groups with sample sizes of three or greater are included, the system will test the normality of the residuals and homogeneity of variances to see whether the data is approximately normal or whether a log transformation or rank transformation should be used. Levene's test[7] will be used to assess homogeneity of group variances and Shapiro-Wilk's[8] test will be used to test the normality of the residuals.

On the raw data, if Levene's test is not significant ($p \geq 0.01$) and Shapiro-Wilk's test is not significant ($p \geq 0.01$), then a normal distribution will be used. If either the Levene's test is significant ($p < 0.01$) or Shapiro-Wilk's test is significant ($p < 0.01$), normality and homogeneity of variances will be tested with a log transformation used on the data.

On the log transformed data, if Levene's test is not significant ($p \geq 0.01$) and Shapiro-Wilk's test is not significant ($p \geq 0.01$), then a log normal distribution will be used. If either the Levene's test is significant ($p < 0.01$) or Shapiro-Wilk's test is significant ($p < 0.01$), then a rank transformation will be used on the data.

Raw or Log Transformed data:

A one way analysis of variance will be used to test each endpoint for the effects of treatment.[9]

If the treatment effect is significant ($p < 0.05$), linear contrasts will be constructed for a Dunnett's pair-wise comparison of treatment groups as described above.

Rank transformed data:

A Kruskal-Wallis test will be used to test each endpoint for the effects of treatment.[9]

---

[7] Milliken GA, Johnson DE., Analysis of messy data. London: Chapman and Hall; 1992.
[8] Royston, J. P., "Approximating the Shapiro-Wilk W Test for Nonnormality." Statistics and Computing 2:117–119; 1992.
[9] Zar JH., Biostatistical analysis. 4th ed. New Jersey: Prentice Hall; 1999.

If the treatment effect is significant ($p<0.05$), a non-parametric Dunn's pair-wise comparison test of each treatment group with the control group.

Results of all pair-wise comparisons will be reported at the 0.05 and 0.01 significance levels. All endpoints will be analyzed using two-tailed tests unless indicated otherwise.

8. STUDY REPORTS

Progress/Study Updates:	As Requested
Report Format:	Comprehensive
Tabulated Summary:	Unaudited tabulated summary will be compiled from the report, in accordance with ICH guidelines, for inclusion in the Common Technical Document (CTD).
Copies Issued:	1 Regulatory Compliant PDF
Electronic Data:	Electronic datasets created following the Standard for Exchange of Nonclinical Data Implementation Guide (SENDIG)[10] will be provided after study finalization. When work in support of this study is conducted at a Test Site (*i.e.*, Bioanalysis, TK modeling, etc.), an electronic version of all data should be provided to Testing Facility. Failure to do so may result in lengthened SEND dataset completion timelines.
Special Considerations:	A signed version of the pathology contributing scientist report (CSR) will be issued with or subsequent to the issuance of the audited draft report. Until the signed pathology CSR is issued, the unsigned pathology CSR will be marked 'Not Intended for Regulatory Submission' and will be listed on the Statement of Compliance in the audited draft report relating to the unsigned nature of this CSR. After the pathology CSR is signed, any subsequent revisions will be made in the form of an amended CSR, which will delineate the changes, as well as the reasons for those changes.<br>Six months after issuance of the draft report (overall report), if no requested revisions or instructions to finalize have been communicated by the Sponsor, the draft report may be issued as a final report, signed by the Study Director, and submitted to the Sponsor. Any modifications or

[10] Standard for Exchange of Nonclinical Data Implementation Guide: Nonclinical Studies, CDISC Standard for Exchange of Nonclinical Data Team.

changes to the draft report requested six months after issuance of the draft will be performed at additional cost to the Sponsor.

9. DATA AND SPECIMEN RETENTION

Materials:	All raw data, documentation, records, protocol, specimens (or samples if archived), and the final report.
Storage Location:	Testing Facility or an Testing Facility contracted archive facility.
Length of Retention:	One year following issuance of the audited draft report.
Disposal:	The Sponsor will be contacted annually by Testing Facility archive staff regarding the retained material and will be responsible for the incurred costs for the return, disposal, or continued storage of any study generated material retained after that time.
Special Considerations:	Data generated at the Sponsor's designated lab will be retained by that facility for at least the period specified above.<br>It is the responsibility of the Sponsor to notify the Study Director of any data generated from tests outside of the scope of this protocol using samples/specimens shipped back to the Sponsor for possible inclusion into the final study report.<br>Data/specimens shipped to the Sponsor or Sponsor-designated location (excluding wet specimens obtained from blood, urine/feces and/or biological fluids), will be archived by the Sponsor.

10. APPROVAL

10.1. Sponsor Approval

24 February 2020
Date of Sponsor Approval

10.2. Study Director Approval

DocuSigned by:

Signer Name: Sarah Davis
Signing Reason: I approve this document
Signing Time: 25-Feb-2020 | 11:07:24 EST
CBB533787C4D4D21A7693D214BF98FA5

Study Director/Date

FINAL PROTOCOL

Testing Facility Study No. 2759-001

Sponsor Reference No. CLN7-001

A Single Dose Toxicity Study of Test Article Administered by Intrathecal Injection in Rats

SPONSOR:
Foundation for Batten Hope
3963 Maple Ave., Suite 390
Dallas, TX 75219
USA

TESTING FACILITY:
MPI Research
54943 North Main Street
Mattawan, MI 49071
USA

TABLE OF CONTENTS

1. INTRODUCTION 3
2. DOSE FORMULATION AND PREPARATION 6
3. TEST SYSTEM 7
4. STUDY DESIGN 10
5. ANTEMORTEM STUDY EVALUATIONS 11
6. ANIMAL DISPOSITION AND POSTMORTEM STUDY EVALUATIONS 15
7. STATISTICS 22
8. STUDY REPORTS 24
9. DATA AND SPECIMEN RETENTION 25
10. APPROVAL 27

1. INTRODUCTION

1.1. Study Objective

The objective of this study is to further characterize the toxicity, biodistribution, and gene expression of the test article. The test article is being developed for treatment of the neurodegenerative Batten Disease (CLN7 subtype).

1.2. Proposed Study Schedule

The following table contains proposed dates for study activities. Actual dates will be listed in the study report.

Study Initiation Date:	Date Study Director signs Study Approval in this protocol
Experimental Start Date:<br>*(First day of dosing)*	14 September 2018
Experimental Termination Date:<br>*(Last animal removed from study)*	17 December 2018
Draft Report Mail Date:	Approximately 10 weeks after final necropsy

1.3. Contact Information

Study Director:	Sarah Davis, BS, LATG<br>Tel: 269-668-3336 ext. 1250<br>Fax: 269-668-4151<br>E-mail: sarah.davis@mpiresearch.com
Alternate Contact:	Scott T. Wilson, BS, LAT<br>Tel: 269-668-3336 ext. 1610<br>Fax: 269-668-4151<br>E-mail: scott.wilson@mpiresearch.com
Sponsor Representative:	Gina Hann<br>Tel: 520-331-6283<br>E-mail: gina.hann@gmail.com
Alternate Sponsor Representative:	Steven Gray, PhD<br>Tel: 214-648-0670

	E-mail: steven.gray@UTSouthwestern.edu
Principal Investigator for Serum, CSF, qPCR, and RT-qPCR Sample Analysis:	Steven J Gray, PhD<br>University of Texas Southwestern Medical Center - Gray Lab<br>6000 Harry Hines Blvd<br>NA02.508<br>Dallas, TX 75390<br>Office: 214-648-0670<br>Lab: 214-648-0671<br>E-mail: steven.gray@UTSouthwestern.edu
Lead Quality Assurance:	Janis Kissel, BS, RQAP-GLP<br>Tel: 269-668-3336, ext. 3170<br>Fax: 269-668-4151<br>E-mail: janis.kissel@mpiresearch.com

1.4. Regulatory Compliance, Animal Welfare Standards, and Study Guidelines

Quality Assurance:

- Will assure compliance with the protocol, Testing Facility Standard Operating Procedures (SOPs) and the GLP regulations.
- Will make available inspection records to the Sponsor Representatives during visits to Testing Facility.

GLP Regulation:

- United States Food and Drug Administration (FDA) Good Laboratory Practice (GLP) Regulations, 21 Code of Federal Regulations (CFR) Part 58

Animal Welfare Standards:

- Guide for the Care and Use of Laboratory Animals[1]

Guidelines:

- Current International Council for Harmonisation (ICH) Harmonised Tripartite Guidelines and generally accepted procedures for the testing of pharmaceutical compounds.[2]

1.5. Alteration of Design

Changes to this approved protocol may be made as the study progresses, and all reasonable efforts will be made to discuss necessary changes, in advance, with the Sponsor. Due to the

[1] Guide for the Care and Use of Laboratory Animals. 8th ed., Washington, D.C., National Academies Press, 2011.
[2] Guidance on non-clinical safety studies for the conduct of human clinical trials and marketing authorization for pharmaceuticals. ICH M3(R2), 2009 June 11 (ICH Implementation Date).

urgent or temporary nature of some protocol changes, the Study Director may authorize a change using a Planned Event form. The form will describe the change, the rationale for the change, and the effective date (or date range, in the instance of a temporary modification) of the change. Such situations will be considered planned deviations until the protocol amendment is prepared and signed, when applicable. In the instance of temporary modifications where a permanent change to the protocol will not be made, the situation will be considered a planned deviation and no protocol amendment will be issued. Resulting protocol amendments will be signed and dated by the Study Director and will identify the date of Sponsor approval, either written or verbal. Signed protocols and protocol amendments will be issued to the Sponsor, including the Sponsor Representative(s), the Sponsor Monitor(s), and the Principal Investigator(s), in addition to other key contact personnel listed in the protocol, and appropriate Testing Facility personnel.

1.6. Proposed Computer Systems

The following are the proposed computer systems to be used during the conduct of this study and their primary function. The actual systems and versions used will be documented in the final report.

Computer System Name	Description
Logbook:	Electronic notebook and data collection system for veterinary communications, observations, and treatments.
Testing Facility ExyLIMS:	A comprehensive laboratory information management system used to manage data, including but not limited to: instrumentation, test articles, standards, and samples.
NextDocs®:	Electronic documentation management of Deviation Events and Corrective and Preventative Actions (CAPA).
Provantis™:	Client-server, Oracle-based system used for electronic documentation and data management from compound receipt through reporting.
SAS®:	An integrated system of software products that enables a user to perform data entry, retrieval, data management, reporting, graphics, statistical analysis, and applications development.

Computer System Name	Description
Siemens Environmental Monitoring and Niagara Framework® Software System:	Environmental monitoring, alarming, and reporting applications.

2. DOSE FORMULATION AND PREPARATION

2.1. Test Article and Vehicle Information

2.1.1. Test Article Information

Test Article Name: scAAV9/JeT-hCLN7opt-SV40pA

Storage Conditions: Frozen (-60 to -90°C)

Description: A description, lot number, storage conditions, expiration date, safe handling procedures, physical properties, as well as other relevant information will be documented in the study data.

Characterization: The Sponsor will provide documentation on the strength, purity, composition, stability, and other pertinent information on each batch of test article, unless otherwise noted. Note, if the Sponsor does not supply the above information (e.g. certificate of analysis), this will be listed on the Statement of Compliance in the final report.

Reserve Sample: A reserve sample from each lot of test and control articles used in this study will be collected and stored at Testing Facility in a secure area with the appropriate environmental controls. If multiple studies are conducted with the same test article, a common reserve sample may be taken and labeled and stored appropriately.

Test Article Contact: Gina Hann

Tel: 520-331-6283

E-mail: gina.hann@gmail.com

Test Article Disposition: The Sponsor will be contacted for proper disposition of materials (retain/ship/discard) after completion of the in-life phase of the study, and following confirmation that these materials are not assigned to other studies.

2.1.2. Vehicle Information

Vehicle Name: Isotonic phosphate-buffered saline containing 5% w/v sorbitol (provided by the sponsor)

Storage Conditions: The vehicle will be stored frozen (-60 to -90°C) until initial use. Once thawed, the vehicle should be stored refrigerated (2 to 8°C) and used within 2 weeks.

Characterization: The Sponsor will provide documentation on the strength, purity, composition, stability, and other pertinent information on each batch of control article, unless otherwise noted. Note, if the Sponsor does not supply the above information (e.g. certificate of analysis), this will be listed as a GLP exception in the final report.

2.2. Dose Formulation Details

Preparation: Test article will be dispensed as instructed by the Sponsor. The high and middle doses will be used as supplied (without dilution). The low dose will be diluted 1:4 (i.e., 1 part test article and 3 parts vehicle) prior to injection.

Frequency: Dispensed on dosing days

Storage Conditions: Frozen (-60 to -90°C) until initial use. Once thawed, the test article should be stored refrigerated (2 to 8°C) and used within 2 weeks.

3. TEST SYSTEM

3.1. Test System Details

Species: Rat

Strain: CD® [Crl:CD®(SD)]

Source: Charles River Laboratories

3.2. Justification of Test System

The current state of scientific knowledge and the applicable guidelines cited previously in this protocol do not provide acceptable alternatives, *in vitro* or otherwise, to the use of live animals to accomplish the purpose of this study. "The development of knowledge necessary for the improvement of the health and well-being of humans as well as other animals requires *in vivo*

experimentation with a wide variety of animal species."[3] "Whole animals are essential in research and testing because they best reflect the dynamic interactions between the various cells, tissues, and organs comprising the human body."[4]

The rat is the usual rodent model[5] used for evaluating the toxicity of various classes of chemicals and for which there is a large historical database.

3.3. Test System Receipt

Expected Age at Arrival:	Ordered to be eight weeks of age at administration
Expected Weight at Arrival:	Commensurate with age; males will generally weigh 215 to 260g and females will generally weigh 165 to 210g, as measured within three days of arrival. The actual range will be documented in the data.
Number Ordered:	
Male:	67
Female:	67
Number on Study:	
Male:	60
Female:	60
Acclimation Duration:	At least one week from initial receipt of animals to Testing Facility.
Acclimation Details:	During this acclimation period, all animals will be observed daily for any clinical signs of disease, and all animals will be given a detailed clinical examination prior to selection for study.

3.4. Selection for Study

Body Weight Range:	±20% of the mean body weight for each sex

[3] "Principles for the Utilization and Care of Vertebrate Animals Used in Testing, Research, and Training", Federal Register, 1985 May 20; 50(97).
[4] "Position Statement on the Use of Animals in Research", 1993 Feb 26; NIH Guide 22(8).
[5] Guidance for Industry, Investigators, and Reviewers: Exploratory IND Studies, U.S. F.D.A. Center for Drug Evaluation and Research (CDER), 2006 Jan.

Randomization:	By sex, into treatment groups using a standard, by weight, randomization procedure.<br>All animals with any evidence of disease or physical abnormalities will not be selected for study.

3.5. Method of Identification

Each animal will be assigned an animal number to be used in Provantis™ and will be implanted with a microchip bearing a unique identification number. The individual animal number, implant number, and the Testing Facility study number will comprise a unique identification for each animal. The animal cage will be identified by the study number, animal number, group number, and sex.

3.6. Husbandry

Housing:	Pair-housed, when possible (animals may be housed two to three/cage during acclimation and in-life depending on study design)
Caging:	Solid bottom cages with nonaromatic bedding. The bedding will be from an approved supplier and documented in the study data.
Temperature and Humidity:	Temperature and humidity will be maintained according to Testing Facility SOP.
Lighting:	Fluorescent lighting will be provided via an automatic timer for approximately 12 hours per day. On occasion, the dark cycle may be interrupted intermittently due to study-related activities.
Water:	Tap water will be supplied *ad libitum* to all animals via an automatic water system unless otherwise indicated.
Diet:	The basal diet will be block Lab Diet® Certified Rodent Diet #5002, PMI Nutrition International, Inc. This diet will be available *ad libitum* unless designated otherwise. Each lot number used will be identified in the study records.
Water and Diet Contaminants:	There are no known contaminants in the food or water that would interfere with this study. The drinking water used will be monitored for specified contaminants at periodic intervals according to Testing Facility SOP.
Supplemental Enrichment:	Animal enrichment will be provided according to Testing Facility SOP.

4. STUDY DESIGN

Group	Treatment	Dose Level (vg/animal)	Dose Volume (µL)	Dose Concentration (vg/µL)	Number of Animals					
					Necropsy Day 8		Necropsy Day 29		Necropsy Day 91	
					M	F	M	F	M	F
1	Vehicle	0	60 µL	0	5	5	5	5	5	5
2	Test Article Low Dose	$5x10^{11}$	20 µL	$2.5x10^{10}$	5	5	5	5	5	5
3	Test Article Middle Dose	$2x10^{12}$	20 µL	$1x10^{11}$	5	5	5	5	5	5
4	Test Article High Dose	$6x10^{12}$	60 µL	$1x10^{11}$	5	5	5	5	5	5
**Total Number of Animals:**					20	20	20	20	20	20

4.1. Justification of Dose Levels

The dose level was selected by the Sponsor, or in consultation with the Sponsor, on the basis of available data from previous studies and discussion with the FDA in a preIND meeting. The low and middle doses have been tolerated well in pilot mouse studies, and the middle dose is the target dose to model the proposed clinical dose in CLN7 patients. The middle dose has shown sufficient to improve pathological signatures of the disease in mice. Per the discussion with the FDA, we were advised to bracket the "middle" target dose with lower and higher doses to complete the dose-responsive nature of any effect.

4.2. Administration

Route: Intrathecal (IT) injection

Frequency/Duration: The test article and vehicle will be administered once on Day 1.

Details: Animals will be anesthetized with Isoflurane and placed in a ventral recumbency and the lumbar cistern will be accessed via a percutaneous needle stick. The location of the needle may be verified using fluoroscopy. Once the needle is in place, the test article will be injected and the needle removed. Animals will then be allowed to recover. The test article will be left at room temperature for 15 to 30 minutes prior to dosing but no more than 2 hours.

4.3. Justification for Route of Administration

Intrathecal injection is the intended route of administration of this test article in humans.

5. ANTEMORTEM STUDY EVALUATIONS

5.1. Cageside Observations

Frequency: At least twice daily

Details: Animals will be observed for morbidity, mortality, injury, and availability of food and water. Any animals in poor health will be identified for further monitoring and possible euthanasia.

5.2. Detailed Clinical Observations

Frequency: Weekly

Details: Observations will include, but will not be limited to, evaluation of the skin, fur, eyes, ears, nose, oral cavity, thorax, abdomen, external genitalia, limbs and feet, respiratory and circulatory effects, autonomic effects such as salivation, nervous system effects including tremors, convulsions, reactivity to handling, unusual behavior.

5.3. Body Weight

Frequency: Within three days of arrival, at least once prior to randomization and/or Day -1, and once weekly thereafter.

5.4. Food Consumption

Frequency: Weekly

5.5. Fusion v6 Rotating Rod System

Frequency: Animals scheduled for Day 29 and Day 91 necropsy only: predose and prior to necropsy.

Details: The apparatus is set to accelerate from -4 rpm to -40 rpm (the negative sign indicates direction, in this case it means the animal will ambulate away from the observer) over the course of 3 minutes. This indicates that the rod will be accelerating at a rate of 0.2 revolutions per second. The elapsed time from the start of the trial to the animal's fall and the speed of the rod at the time of the fall is recorded digitally by the instrument. Each animal will perform 3 consecutive trials with at least 15 minutes between trials. The average of all 3 trials will be reported for each animal.

The morning of each testing a training session will be conducted. The training session will consist of the animal being placed on a rotating rod

which is set to a constant speed of -4 rpm for 3 minutes. Animals that fall off during training are immediately placed back on the rotating rod until the 3 minutes is completed. Data from the training sessions will not be reported.

The computer software is not validated and this will be listed as a GLP exception in the final report.

5.6. Clinical Pathology Sample Collection and Analysis

Fasting Requirements:	Free access to drinking water but fasted overnight prior to blood collection.
Blood Sample Volume:	Hematology: 0.5 to 1mL<br>Coagulation: 1.2mL<br>Clinical Chemistry: 0.8 to 1.3mL
Site of Blood Sample Collection:	Vena cava after carbon dioxide inhalation
Anticoagulant:	Hematology: K_2EDTA<br>Coagulation: Sodium Citrate<br>Clinical Chemistry: Serum Separator
Sample Disposition:	All samples will be disposed of per Testing Facility SOP.

The following clinical pathology tests will be conducted on available samples and as survival allows.

Sample Type	Number of Animals	Intervals	Parameters Evaluated
**Hematology**	All	Termination	• leukocyte count (total and absolute differential)<br>• erythrocyte count<br>• hemoglobin<br>• hematocrit<br>• mean corpuscular hemoglobin, mean corpuscular volume, mean corpuscular hemoglobin concentration (calculated)<br>• absolute reticulocytes<br>• platelet count<br>• RDW<br>• blood smear preserve and stain*

Sample Type	Number of Animals	Intervals	Parameters Evaluated
			*blood smear review may be performed on select animals per Testing Facility SOP.
**Coagulation**	All	Termination	• prothrombin time<br>• activated partial thromboplastin time<br>• fibrinogen
**Clinical Chemistry**	All	Termination	• alkaline phosphatase<br>• total bilirubin (with direct bilirubin if total bilirubin exceeds 1 mg/dL)<br>• aspartate aminotransferase<br>• alanine aminotransferase<br>• urea nitrogen<br>• creatinine<br>• total protein<br>• albumin<br>• globulin and A/G (albumin/globulin) ratio (calculated)<br>• glucose<br>• total cholesterol<br>• triglycerides<br>• electrolytes (sodium, potassium, chloride)<br>• calcium<br>• phosphorus<br>• sample quality

5.7. Serum Sample Collection and Analysis

5.7.1. Sample Collection

Group(s)	Animals/Sex/Interval	Interval
All	All	Termination

Fasting Requirements: The animals will not be fasted before blood collection unless concurrent with fasting for other procedures.

Collection Site: Vena cava after carbon dioxide inhalation

Whole Blood Volume/Sample: Blood: 0.6 to 1 mL

Anticoagulant: Serum separator

Whole Blood Storage: Room Temperature

Additional Container Label Requirements:	Testing Facility study number, relative study day, animal number, and the date and time interval of collection.
Sample Requirements:	Serum: 3 aliquots, 100 µL each. If the total volume of serum is less than 300 µL, then lower equal aliquots are acceptable.
Special Requirements:	The serum may be initially stored on dry ice.
Final Storage Temperature:	Processed samples will be placed on dry ice or in frozen (-60 to -90°C) storage within 60 minutes of centrifugation.

5.7.2. Sample Handling and Analysis

5.7.2.1. Sample Handling

Shipping Conditions:	Frozen on dry ice
Primary Contact for Sample Shipment:	Erik Lykken<br>Attn: Gray Lab<br>6000 Harry Hines Blvd<br>NA02.508<br>University of Texas Southwestern Medical Center<br>Dallas, TX 75390<br>Tel: 214-648-0671<br>E-mail: Erik.Lykken@UTSouthwestern.edu
Shipping Details:	Samples will be shipped on Monday through Wednesday for next day delivery. The primary contact will be notified prior to each shipment.

5.7.2.2. Serum Analysis

Analysis Performed by:	The analytical work will be conducted by a Sponsor-contracted laboratory, the Laboratory of Dr. Steven Gray, using an analytical method to be added by amendment if sample analysis is conducted. Samples may be retained for possible future analysis.
Regulatory Requirements:	The work performed in conjunction with this study will not be conducted in compliance with GLPs. A final report will be prepared and submitted to the Sponsor and Test Facility for inclusion as an appendix in the main study final report., if sample analysis is conducted.

Sample Disposition: Following analysis, any residual samples or backup samples will be retained by the Test Facility (The Laboratory of Dr. Steven Gray) as per their respective SOPs. Any unshipped back up samples remaining at Testing Facility will be archived by Testing Facility.

6. ANIMAL DISPOSITION AND POSTMORTEM STUDY EVALUATIONS

6.1. Animal Disposition

Moribundity: Moribund animals will be subject to Testing Facility SOP criteria and procedures. If possible, the samples below will be collected from animals euthanized *in extremis*, following veterinary consultation. A veterinary consultation is not required if the samples are collected following anesthesia or euthanasia. Blood collection methods utilized for animals euthanized *in extremis* may include suitable methods other than those presented in the respective blood collection section(s) of this protocol.

Sample Type	Groups	Volume	Anticoagulant
Hematology	All	1mL	K_2EDTA
Coagulation		1.2mL	Citrate
Clinical Chemistry		1.3mL	Serum Separator
Serum	All		
CSF	All		

Necropsy examinations will be performed seven days a week. Animals that are found dead or euthanized *in extremis* after regular working hours will be refrigerated overnight and necropsies performed at the start of the next day. Any animal found dead or euthanized *in extremis* will undergo a gross necropsy examination and macroscopic observations will be recorded. Protocol designated tissues will be saved from these animals.

Method of Euthanasia: Euthanasia will be by carbon dioxide inhalation followed by an Testing Facility SOP approved method to ensure death, e.g. exsanguination.

Disposition of Animals Not Assigned: Extra animals obtained for this study, but not placed on study, will be either transferred to a Testing Facility stock or training colony, or euthanized and discarded. The final disposition of each animal will be documented in the study records.

Disposition of Study Animals: All main study animals will be euthanized and subjected to a complete necropsy examination, performed under procedures approved by a veterinary pathologist in accordance with Testing Facility SOP.

6.2. Postmortem Evaluations

Necropsy Schedule: Terminal: Day 8, Day 29, and Day 91

External Evaluations: The animals will be examined carefully for external abnormalities including palpable masses.

Macroscopic Evaluations: The skin will be reflected from a ventral midline incision, and any subcutaneous masses will be identified and correlated with antemortem findings. The abdominal, thoracic, and cranial cavities will be examined for abnormalities and the organs removed, examined, and, where required, placed in the appropriate fixative.

Tissue Fixation: All tissues will be placed in a neutral buffered formalin, except that the eyes, including the optic nerves, and testes will be fixed using a modified Davidson's fixative[6]. Eyes and testes will be placed into formalin after fixation. Formalin will be infused into the lung via the trachea and into the urinary bladder.

Organ Weights: Body weight and the organ weights identified in the organ list table will be recorded for all animals at the scheduled necropsies, and appropriate organ weight ratios will be calculated (relative to body and brain weights). Paired organs will be weighed together. Organs will not be weighed for animals found dead or euthanized *in extremis*.

A combined weight of the thyroid gland with parathyroid glands will be obtained, and the right mandibular/sublingual salivary glands will be weighed together. The thyroid/parathyroid gland and pituitary gland will be weighed following fixation.

Microscopic Evaluation: Microscopic examination of fixed hematoxylin and eosin-stained paraffin sections will be performed on sections of tissues and from the groups and/or intervals identified in the following table and all animals found dead or euthanized *in extremis*.

Handling of Specific Tissues: Bone marrow smears will not be prepared for animals found dead but will be prepared for animals euthanized *in extremis* (with the exception of euthanized animals requiring refrigeration prior to necropsy).

[6] Latendresse JR, Warbrittion AR, Jonassen H, Creasy DM. Fixation of testes and eyes using a modified Davidson's fluid: comparison with Bouin's fluid and conventional Davidson's fluid. Toxicol Pathol. 2002 Jul Aug;30(4):524 33.

The spinal cord and DRG (cervical, lumbar, thoracic) will be collected in situ. The samples will be decalcified and embedded in paraffin with cross-sections that include the spinal cord, nerve roots, and DRG.

Additional Testing: Evaluation of the bone marrow smears will be performed at the discretion of the Study Director and/or Sponsor.

The pathologist may use special stains and techniques as needed to aid in the diagnosis of specific lesions. If after routine sectioning, a tissue is missed, one recut will be requested either by re-sectioning the tissue in the block or ordering a wet tissue recut. If the tissue is still missing, the block will not be re-sectioned unless the missing tissue is determined to be a potential target organ. In this case, the tissue will be re-sectioned until located or until it is determined that it is not present in the block or in wet tissue. Non-protocol-required tissues that are coincidentally within the plane of a protocol-required tissue will be examined by the study pathologist, and any findings that impact the interpretation of the study will be documented. In some instances, tissues may be collected and processed to paraffin block or glass slide stage prior to defining the tissues required for microscopic examination (e.g., intermediate and/or recovery groups). In these cases, only protocol-required tissues will be examined microscopically and other tissues present on the slide will not be examined.

Digital images for illustrative purposes may be taken. These images will not be used for data generation or interpretation, and will not be archived or included in the final report.

Pathology Evaluation: In the unlikely event that an Testing Facility pathologist cannot be scheduled for the pathology evaluation due to logistics/timing, the pathology evaluation may be outsourced (by protocol amendment) to an external ACVP-certified veterinary pathologist.

6.3. qPCR and RT-qPCR Sample Collection and Analysis

qPCR Sample Collection: Each organ/tissue sample will be divided in half; one part used for DNA purification and the other part used for RNA purification. When possible, duplicate samples will be collected (4 total per tissue/organ) as long as each sample is ≥10 mg.

Samples of the organs or tissues collected for qPCR will be placed in labeled 2-mL microfuge tubes (one tube/sample), snap-frozen into liquid nitrogen, and then stored at -60°C to -90°C until shipped to the Sponsor designated laboratory for analysis. Samples collected for RT-qPCR analysis of the transgene mRNA will be completely immersed

into labeled 2 mL microfuge tubes prefilled with 1.5 mL RNALater. Samples will be stored refrigerated (2 to 8ºC) for up to 24 hours and then stored frozen (-60 to -90ºC) with the RNALater removed until shipped to the Sponsor designated laboratory for analysis.

The samples for DNA analysis will be collected using strict aseptic techniques and disposable instruments for each tissue or organ. Care will be given to ensure that cross contamination between tissues does not occur. Gloves will be changed between collection and dissection of each tissue for PCR analysis. In addition, non-disposable instruments will be wiped down with a 10% bleach solution, rinsed with water followed by a wipe down of 100% ethanol between groups. The cutting board will be wiped down with a 10% bleach solution, rinsed with water followed by a wipe down of 100% ethanol between each of the specified organs or sterile trays may be used. The tissues for PCR analysis will be collected prior to sample collection for any other parameters. Samples for qPCR and RT-qPCR will not collected for animals found dead or euthanized *in extremis*.

qPCR and RT-qPCR Sample analysis: If deemed necessary by the sponsor, qPCR and/or RT-qPCR analysis will be conducted. Purified DNA will be used as template for qPCR to detect the transgene in each sample relative to a genomic DNA reference, with a sensitivity of ≤50 copies of vector DNA per microgram of rat genomic DNA. Purified RNA will be used for cDNA synthesis, and the cDNA of the optimized human CLN7 will be quantified relative to endogenous rat GAPDH cDNA and rat CLN7 cDNA levels. Only tissues found positive for vector presence at ≥ 50 double-stranded copy of vector DNA per 1 µg rat DNA will be tested for gene expression by RNA analysis.

6.3.1. Sample Handling and Analysis

6.3.1.1. Sample Handling

Shipping Conditions: Frozen on dry ice

Primary Contact for Sample Shipment: Erik Lykken

Attn: Gray Lab

6000 Harry Hines Blvd

NA02.508

University of Texas Southwestern Medical Center

Dallas, TX 75390

Tel: 214-648-0671
E-mail: Erik.Lykken@UTSouthwestern.edu

Shipping Details: Samples will be shipped on Monday through Wednesday for next day delivery. The primary contact will be notified prior to each shipment.

6.3.1.2. qPCR and RT-qPCR Sample Analysis

Analysis Performed by: If needed, the analytical work will be conducted by a Sponsor-contracted laboratory, the Laboratory of Dr. Steven Gray, using an analytical method to be added by amendment if sample analysis is conducted. Samples may be retained for possible future analysis.

Regulatory Requirements: The work performed in conjunction with this study will not be conducted in compliance with GLPs. A final report will be prepared and submitted to the Sponsor and Test Facility for inclusion as an appendix in the main study final report, if sample analysis is conducted.

Sample Disposition: Following analysis, any residual samples or backup samples will be retained by the Test Facility (The Laboratory of Dr. Steven Gray) as per their respective SOPs. Any unshipped back up samples remaining at Testing Facility will be archived by Testing Facility.

6.4. Organs or Tissues to be Weighed, Preserved, and Microscopically Examined

Organ	Weigh	Collect at Necropsy	Histology Processing	Histopathology Evaluation Groups[2] All Groups	Biodistribution/Gene Expression All Groups
Animal ID	-	X	-	-	
Artery, aorta	-	X	-	-	
Body cavity, nasal	-	X	-	-	
Bone marrow	-	X	-	-	
Bone marrow smear	-	X[1]	-	-	
Bone, femur (one)	-	X	-	-	
Bone, sternum	-	X	-	-	
Brain- at least 3 coronal cross sections (hindbrain including brainstem and cerebellum, midbrain, and forebrain)	X	X	X	X	X
Cervix	-	X	-	-	
Epididymis (both)	X	X	-	-	
Esophagus	-	X	-	-	
Eye (both)	-	X	-	-	
Gland, adrenal (both)	X	X	-	-	
Gland, clitoral (both)	-	X	-	-	

Organ	Weigh	Collect at Necropsy	Histology Processing	Histopathology Evaluation Groups[2] All Groups	Biodistribution/Gene Expression All Groups
Gland, lacrimal (both, extra-orbital)	-	X	-	-	
Gland, Harderian	-	X (both)	-	-	
Gland, mammary	-	X	-	-	
Gland, parathyroid (both)	-	X	-	-	
Gland, pituitary	X	X	-	-	
Gland, preputial (both)	-	X	-	-	
Gland, prostate	X	X	-	-	
Gland, salivary	X (right mandibular/ sublingual salivary glands)	X (both) sub-mandibular, sublingual, parotid	-	-	
Gland, seminal vesicle (both)	-	X	-	-	
Gland, thyroid (both)	X	X	-	-	
Gland, Zymbal's (both)	-	X	-	-	
Gut-associated lymphoid tissue	-	X	-	-	
Heart	X	X	X	X	X
Joint, femorotibial (one)	-	X	-	-	-
Kidney (both)	X	X	X	X	X
Large intestine, cecum	-	X	-	-	-
Large intestine, colon	-	X	-	-	-
Large intestine, rectum	-	X	-	-	-
Larynx	-	X	-	-	-
Liver	X	X	X	X	X
Lung	X	X	X	X	X
Lymph node, mandibular	-	X (both)	X (one)	X (one)	X
Lymph node, mesenteric	-	X	X	X	X
Macroscopic abnormalities	-	X	X	X	-
Muscle, skeletal (bicep femoris and gastrocnemius)	-	X	X	X	X
Nasopharynx	-	X	-	-	-
Nerve, optic (both)	-	X	-	-	-
Nerve, sciatic	-	X (both)	-	-	-
Ovary (both)	X	X	X	X	X
Oviduct (both)	X	X	-	-	-
Pancreas	-	X	-	-	-
Skin	-	X	-	-	-
Small intestine, duodenum	-	X	-	-	-
Small intestine, ileum	-	X	-	-	-
Small intestine, jejunum	-	X	-	-	-

Organ	Weigh	Collect at Necropsy	Histology Processing	Histopathology Evaluation Groups[2] All Groups	Biodistribution/Gene Expression All Groups
Spinal cord, cervical (to include DRG and nerve roots)	-	X	X	X	X
Spinal cord, thoracic (to include DRG and nerve roots)	-	X	X	X	X
Spinal cord, lumbar (to include DRG and nerve roots)	-	X	X	X	X
Spleen	X	X	X	X	-
Stomach	-	X	-	-	-
Testis (both)	X	X	X	X	X
Thymus	X	X	-	-	-
Tongue	-	X	-	-	-
Trachea	-	X	-	-	-
Ureter (both)	-	X	-	-	-
Urinary bladder	-	X	-	-	-
Uterus	X	X	-	-	-
Vagina	-	X	-	-	-

X = Procedure to be conducted; - = Not applicable.

[1] Two bone marrow smears will be collected from the femur at scheduled and unscheduled necropsies (for possible examination). Smears will not be collected from animals that are found dead or from animals that were euthanized moribund and then stored in the refrigerator prior to necropsy. Bone marrow smears are allowed to air dry and are not fixed in formalin.

[2] At the discretion of the Study Pathologist, findings for extraneous tissues (non-protocol tissues that may be present on a slide as a result of collection of protocol tissues) will also be recorded when observed.

6.5. Cerebrospinal Fluid (CSF) Sample Collection and Analysis

6.5.1. Sample Collection

Frequency: All animals at termination and animals euthanized *in extremis*

CSF Volume/Sample: Max attainable

Anticoagulant: None

Additional Container Label Requirements: Testing Facility study number, relative study day, animal number, and the date and time interval of collection.

Sample Requirements: 2 approximately equal aliquots

Final Storage Temperature:	Frozen (-60 to -90°C)

6.5.2. Sample Handling and Analysis

6.5.2.1. Sample Handling

Shipping Conditions:	Frozen on dry ice
Primary Contact for Sample Shipment:	Erik Lykken, PhD<br>Attn: Gray Lab<br>6000 Harry Hines Blvd<br>NA02.508<br>University of Texas Southwestern Medical Center<br>Dallas, TX 75390<br>Tel: 214-648-0671<br>E-mail: Erik.Lykken@UTSouthwestern.edu
Shipping Details:	Samples will be shipped on Monday through Wednesday for next day delivery. The primary contact will be notified prior to each shipment.

6.5.2.2. CSF Analysis

Analysis Performed by:	If needed, the analytical work will be conducted by a Sponsor-contracted laboratory, the Laboratory of Dr. Steven Gray, using an analytical method to be added by amendment if sample analysis is conducted. Samples may be retained for possible future analysis.
Regulatory Requirements:	The work performed in conjunction with this study will not be conducted in compliance with GLPs. A final report will be prepared and submitted to the Sponsor and Test Facility for inclusion as an appendix in the main study final report, if sample analysis is conducted.
Sample Disposition:	Following analysis, any residual samples or backup samples will be retained by the Test Facility (The Laboratory of Dr. Steven Gray) as per their respective SOPs. Any unshipped back up samples remaining at Testing Facility will be archived by Testing Facility.

7. STATISTICS

The following presents a proposed statistical analysis plan. Statistical plans are data dependent, and this analysis plan may require modification if standard data assumptions are not met. Other conceptually equivalent statistical testing routines may also be employed at the discretion of the statistician. The actual analysis plan will be documented in the final report.

The raw data will be tabulated within each time interval, and the appropriate summary statistics will be calculated for each endpoint, sex, and group. For each endpoint, treatment groups will be compared to the control group using the analysis outlined below. Data for some endpoints, as indicated, will be transformed by either a log or rank transformation prior to conducting the specified analysis.

7.1. Statistical Comparisons

Control Group	Comparison Group(s)
1	2, 3, 4

7.2. Group Pair-wise Comparison (General ANOVA)

Endpoints:

- Body Weight and Body Weight Change
- Food Consumption
- Hematology
- Coagulation
- Clinical Chemistry
- Organ Weights
  - Absolute Weights
  - Relative to Body and Brain Weights
- Urinalysis
  - Urine Volume
  - Specific Gravity
  - pH

Description: The experimental unit for the analysis of food consumption will be cage, while for all other endpoints the experimental unit will be the individual animal. Food consumption will be calculated as described in Testing Facility SOP.

If the control group has a sample size less than three, no inferential statistics will be calculated. If a particular endpoint and/or parameter within a given collection interval have the same value across all experimental units, no inferential statistics will be calculated.

Otherwise, for endpoints and/or parameters where all groups with sample sizes of three or greater are included, the system will test the normality of the residuals and homogeneity of variances to see whether the data is approximately normal or whether a log transformation or rank transformation should be used. Levene's test[7] will be used to assess

[7]Milliken GA, Johnson DE., Analysis of messy data. London: Chapman and Hall; 1992.

homogeneity of group variances and Shapiro-Wilk's[8] test will be used to test the normality of the residuals.

On the raw data, if Levene's test is not significant (p≥0.01) and Shapiro-Wilk's test is not significant (p≥0.01), then a normal distribution will be used. If either the Levene's test is significant (p<0.01) or Shapiro-Wilk's test is significant (p<0.01), normality and homogeneity of variances will be tested with a log transformation used on the data.

On the log transformed data, if Levene's test is not significant (p≥0.01) and Shapiro-Wilk's test is not significant (p≥0.01), then a log normal distribution will be used. If either the Levene's test is significant (p<0.01) or Shapiro-Wilk's test is significant (p<0.01), then a rank transformation will be used on the data.

Raw or Log Transformed data:

A one way analysis of variance will be used to test each endpoint for the effects of treatment.[9]

If the treatment effect is significant (p<0.05), linear contrasts will be constructed for a Dunnett's pair-wise comparison of treatment groups as described above.

Rank transformed data:

A Kruskal-Wallis test will be used to test each endpoint for the effects of treatment.[9]

If the treatment effect is significant (p<0.05), a non-parametric Dunn's pair-wise comparison test of each treatment group with the control group.

Results of all pair-wise comparisons will be reported at the 0.05 and 0.01 significance levels. All endpoints will be analyzed using two-tailed tests unless indicated otherwise.

8. STUDY REPORTS

Progress/Study Updates:	As Requested
Terminal (Overall) Report:	Unaudited report followed by Audited report

---

[8]Royston, J. P., "Approximating the Shapiro-Wilk W Test for Nonnormality." Statistics and Computing 2:117–119; 1992.
[9]Zar JH., Biostatistical analysis. 4th ed. New Jersey: Prentice Hall; 1999.

Report Format: Comprehensive

Tabulated Summary: Unaudited tabulated summary will be compiled from the report, in accordance with ICH guidelines, for inclusion in the Common Technical Document (CTD).

Copies Issued: 1 Regulatory Compliant PDF

Electronic Data: Electronic datasets created following the Standard for Exchange of Nonclinical Data Implementation Guide (SENDIG)[10] will be provided after study finalization. When work in support of this study is conducted at a Test Site (*i.e.*, Bioanalysis, TK modeling, etc.), an electronic version of all data should be provided to Testing Facility. Failure to do so may result in lengthened SEND dataset completion timelines.

Special Considerations: A signed version of the pathology contributing scientist report (CSR) will be issued with or subsequent to the issuance of the audited draft report. Until the signed pathology CSR is issued, the unsigned pathology CSR will be marked 'Not Intended for Regulatory Submission' and will be listed on the Statement of Compliance in the audited draft report relating to the unsigned nature of this CSR. After the pathology CSR is signed, any subsequent revisions will be made in the form of an amended CSR, which will delineate the changes, as well as the reasons for those changes.

Six months after issuance of the draft report (overall report), if no requested revisions or instructions to finalize have been communicated by the Sponsor, the draft report may be issued as a final report, signed by the Study Director, and submitted to the Sponsor. Any modifications or changes to the draft report requested six months after issuance of the draft will be performed at additional cost to the Sponsor.

9. DATA AND SPECIMEN RETENTION

Materials: All raw data, documentation, records, protocol, specimens (or samples if archived), and the final report.

Storage Location: Testing Facility or an Testing Facility contracted archive facility.

Length of Retention: One year following issuance of the audited draft report.

[10] Standard for Exchange of Nonclinical Data Implementation Guide: Nonclinical Studies, CDISC Standard for Exchange of Nonclinical Data Team.

<u>Disposal</u>:	The Sponsor will be contacted annually by Testing Facility archive staff regarding the retained material and will be responsible for the incurred costs for the return, disposal, or continued storage of any study generated material retained after that time.
<u>Special Considerations</u>:	Data generated at the Sponsor's designated lab will be retained by that facility for at least the period specified above.<br><br>It is the responsibility of the Sponsor to notify the Study Director of any data generated from tests outside of the scope of this protocol using samples/specimens shipped back to the Sponsor for possible inclusion into the final study report.<br><br>Data/specimens shipped to the Sponsor or Sponsor-designated location (excluding wet specimens obtained from blood, urine/feces and/or biological fluids), will be archived by the Sponsor.

10. APPROVAL

10.1. Sponsor Approval

6 September 2018
Date of Sponsor Approval

10.2. Study Director Approval

07SEP2018

Sarah Davis, BS, LATG
Study Director

Date

10.3. Testing Facility Management Approval

Chris Pagan for

7 Sept 2018

Mark Johnson, MS
Senior Director, Surgery

Date

DEVIATIONS

All deviations that occurred during the study have been authorized/acknowledged by the Study Director, assessed for impact, and documented in the study records. All study plan deviations are listed below.

None of the deviations were considered to have impacted the overall integrity of the study or the interpretation of the study results and conclusions.

The following planned deviations occurred as the result of an intended study modifications.

Postmortem and Pathology

- At the time of trimming, the spinal cord was opened up to remove bilateral DRG's (to include nerve roots, when possible) from the cervical, thoracic, and lumbar regions of the spinal column. The DRG's were each be submitted for processing in individual tissue cassettes and left and right orientation maintained. Spinal cord sections adjacent to the harvested DRG's were trimmed and submitted for processing.

The following unplanned deviations occurred as the result of an unintended study modifications.

In-Life Observations, Measurements, and Evaluations

- During pretreatment several animals received food in progress before Day 1 instead of weekly food consumption starting on Day 1 as stated in protocol.
- On Week 2, the weekly body weights and detailed clinical observations were not conducted for several Day 8 necropsy animals.
- On Week 9, the weekly body weights and detailed clinical observations for 16 animals were not conducted.

Laboratory Evaluations

- At terminal collection, the volume of blood obtained, 0.4 mL for clinical chemistry and 0.4 mL of serum, for Group 2 females (Animal No. 2513 and 2511, respectively) were insufficient for analysis.
- At terminal collection, a Serum sample from 1 Group 2 female (Animal No. 2513) was unable to be obtained.

Postmortem and Pathology

- At terminal necropsy, due to insufficient CSF volumes the technician was unable to collect two approximately equal aliquots for 1 Group 2 Female (Animal No. 2507) and at the time of frozen inventory the sample of CSF could not be found and was not available for analysis.
- At terminal necropsy, 1 Group 3 female (Animal No. 3506) was documented for having a RT-qPCR spinal cord thoracic A and B sample being collection. At the time of transfer no tissues were present in the vials and was not available for analysis.
- At terminal necropsy, the biceps femoris Sample A for 1 Group 4 female (Animal No. 4511) and the mandibular lymph node sample for 1 Group 4 male (Animal No. 4012) for

RT-PCR were placed in liquid nitrogen rather than refrigerated for up to 24 hours prior to frozen storage (-60ºC to -90ºC) with RNALater removed.

- At terminal necropsy, the Brain samples 1 and 2 were collected and combined under Brain Sample for DNA and RNA purification collections for several animals (Animal No. 2503, 3004, 4002, and 4505); therefore, no samples for Brain sample 2 were collected and not available for evaluation.
- At terminal necropsy, the qPCR tissues for several animals (Animal No. 1515, 2515, 3014, and 4015) were not transferred to frozen storage. Samples were found in dry ice container 6 days later and placed immediately on dry ice and laced in frozen storage (-60ºC to -90ºC).
- At terminal necropsy, only 1 bone smear sample was obtained for 1 Group 4 male (Animal No. 4003), short the protocol-specified 2 samples.
- At the time of tissue archival, the paraffin block 4 (Dorsal Root Ganglia) for 1 Group 4 female (Animal 4509) could not be located and was not available to be submitted to records management.

Appendix 2
Test Article Characterization

The Certificate of Analysis was not received.

Appendix 3
Record of Animal Fate and Disposition

Abbreviations
Term – Terminal necropsy

2759-001
A Single Dose Toxicity Study of Test Article Administered by Intrathecal Injection in Rats

Record of Animal Fate and Disposition

Sex: Male Day(s): 1 → 91 Relative to Start Date

Vehicle	Fate	Removal Day
1001	Term	91
1002	Term	91
1003	Term	91
1004	Term	91
1005	Term	91
1006	Term	8
1007	Term	8
1008	Term	8
1009	Term	8
1010	Term	8
1011	Term	29
1012	Term	29
1013	Term	29
1014	Term	29
1015	Term	29

2759-001
A Single Dose Toxicity Study of Test Article Administered by Intrathecal Injection in Rats

Record of Animal Fate and Disposition

Sex: Male Day(s): 1 → 91 Relative to Start Date

Low Dose	Fate	Removal Day
2001	Term	91
2002	Term	91
2003	Term	91
2004	Term	91
2005	Term	91
2006	Term	8
2007	Term	8
2008	Term	8
2009	Term	8
2010	Term	8
2011	Term	29
2012	Term	29
2013	Term	29
2014	Term	29
2015	Term	29

2759-001
A Single Dose Toxicity Study of Test Article Administered by Intrathecal Injection in Rats

Record of Animal Fate and Disposition

Sex: Male Day(s): 1 → 91 Relative to Start Date

Mid Dose	Fate	Removal Day
3001	Term	91
3002	Term	91
3003	Term	91
3004	Term	91
3005	Term	91
3006	Term	8
3007	Term	8
3008	Term	8
3009	Term	8
3010	Term	8
3011	Term	29
3012	Term	29
3013	Term	29
3014	Term	29
3015	Term	29

2759-001
A Single Dose Toxicity Study of Test Article Administered by Intrathecal Injection in Rats

Record of Animal Fate and Disposition

Sex: Male Day(s): 1 → 91 Relative to Start Date

High Dose	Fate	Removal Day
4001	Term	91
4002	Term	91
4003	Term	91
4004	Term	91
4005	Term	91
4006	Term	8
4007	Term	8
4008	Term	8
4009	Term	8
4010	Term	8
4011	Term	29
4012	Term	29
4013	Term	29
4014	Term	29
4015	Term	29

2759-001
A Single Dose Toxicity Study of Test Article Administered by Intrathecal Injection in Rats

Record of Animal Fate and Disposition

Sex: Female Day(s): 1 → 91 Relative to Start Date

Vehicle	Fate	Removal Day
1501	Term	91
1502	Term	91
1503	Term	91
1504	Term	91
1505	Term	91
1506	Term	8
1507	Term	8
1508	Term	8
1509	Term	8
1510	Term	8
1511	Term	29
1512	Term	29
1513	Term	29
1514	Term	29
1515	Term	29

2759-001
A Single Dose Toxicity Study of Test Article Administered by Intrathecal Injection in Rats

Record of Animal Fate and Disposition

Sex: Female Day(s): 1 → 91 Relative to Start Date

Low Dose	Fate	Removal Day
2501	Term	91
2502	Term	91
2503	Term	91
2504	Term	91
2505	Term	91
2506	Term	8
2507	Term	8
2508	Term	8
2509	Term	8
2510	Term	8
2511	Term	29
2512	Term	29
2513	Term	29
2514	Term	29
2515	Term	29

2759-001
A Single Dose Toxicity Study of Test Article Administered by Intrathecal Injection in Rats

Record of Animal Fate and Disposition

Sex: Female Day(s): 1 → 91 Relative to Start Date

Mid Dose	Fate	Removal Day
3501	Term	91
3502	Term	91
3503	Term	91
3504	Term	91
3505	Term	91
3506	Term	8
3507	Term	8
3508	Term	8
3509	Term	8
3510	Term	8
3511	Term	29
3512	Term	29
3513	Term	29
3514	Term	29
3515	Term	29

2759-001
A Single Dose Toxicity Study of Test Article Administered by Intrathecal Injection in Rats

Record of Animal Fate and Disposition

Sex: Female Day(s): 1 → 91 Relative to Start Date

High Dose	Fate	Removal Day
4501	Term	91
4502	Term	91
4503	Term	91
4504	Term	91
4505	Term	91
4506	Term	8
4507	Term	8
4508	Term	8
4509	Term	8
4510	Term	8
4511	Term	29
4512	Term	29
4513	Term	29
4514	Term	29
4515	Term	29

Appendix 4
Individual Detailed Clinical Observations

On occasion, clinical findings may have been observed more than once during the interval and were recorded accordingly. The individual clinical observations table of this appendix reports the findings observed, not the number of times observed.

2759-001
A Single Dose Toxicity Study of Test Article Administered by Intrathecal Injection in Rats

Individual Detailed Clinical Observations

Sex: Male	Animal	Observation Type: Routine	From Week -1 (Start Date) to 13 (Start Date)
Vehicle	1001	Normal	-1, 1, 2, 3
		Hair sparse, Face	4, 5, 6, 7, 8, 10, 11, 12, 13
		Scabbed area, Face	6, 7, 8, 13
	1002	Tail bent	-1, 1, 2, 3, 4, 5, 6, 7, 8, 10, 11, 12, 13
		Hair sparse, Face	8, 10, 11, 12, 13
		Scabbed area, Face	7, 11, 12, 13
	1003	Normal	-1, 1, 2, 3, 4
		Tail bent	5, 6, 7, 8, 9, 10, 11, 12, 13
		Hair discolored, Cervical region, Red	12, 13
	1004	Normal	-1, 1, 2, 3, 4, 5, 6, 7, 8
		Hair discolored, Face, Brown	9, 10, 11, 12, 13
	1005	Normal	-1, 1, 2, 3, 4, 5, 6, 7, 8, 9, 10, 11, 12, 13
	1006	Normal	-1, 1
	1007	Normal	-1, 1
	1008	Normal	-1, 1, 2
	1009	Normal	-1, 1, 2
	1010	Normal	-1, 1, 2
	1011	Normal	-1, 1, 2, 3, 4, 5
	1012	Normal	-1, 1, 2, 3, 4, 5
	1013	Normal	-1, 1, 2, 3, 4, 5
	1014	Normal	-1, 1, 2, 3
		Hair sparse, Face	5
		Scabbed area, Face	4
	1015	Normal	-1, 1, 2, 3, 4, 5
Low Dose	2001	Normal	-1, 1, 2, 3, 4, 5, 6, 7, 8
		Ear/portion of ear missing, Ear/left	12, 13
		Hair sparse, Face	10, 11, 12, 13
	2002	Normal	-1, 1, 2, 3, 4, 5, 7, 8, 10, 11, 12, 13
		Scabbed area, Face	6

Values=Interval seen

2759-001
A Single Dose Toxicity Study of Test Article Administered by Intrathecal Injection in Rats

Individual Detailed Clinical Observations

Sex: Male	Animal	Observation Type: Routine	From Week -1 (Start Date) to 13 (Start Date)
Low Dose	2003	Normal	-1, 1, 2, 3, 4, 5, 6, 7, 8, 10, 11, 12, 13
		Hair sparse, Face	13
	2004	Normal	-1, 1, 2, 3, 4
		Hair sparse, Face	5, 6, 7, 8, 9, 10, 11, 12, 13
		Scabbed area, Face	7, 8, 9, 10, 11, 12, 13
	2005	Normal	-1, 1, 2, 12, 13
		Loss of skin elasticity	3
		Scabbed area, Tail	4, 5, 6, 7, 8, 9, 10, 11
	2006	Normal	-1, 1
	2007	Normal	-1, 1
	2008	Normal	-1, 1
	2009	Normal	-1, 1, 2
	2010	Normal	-1, 1
		Ear/portion of ear missing, Ear/right	2
	2011	Normal	-1, 1, 2, 3, 4, 5
	2012	Normal	-1, 1, 2, 3, 4, 5
	2013	Normal	-1, 1, 2, 3, 4, 5
	2014	Normal	-1, 1, 2, 3, 4
		Hair sparse, Face	5
	2015	Normal	-1, 1, 2, 3, 4, 5
Mid Dose	3001	Normal	-1, 1, 2, 3, 4, 5, 6, 7, 8, 10, 11, 12, 13
	3002	Normal	-1, 1, 2, 3
		Hair sparse, Face	4, 5, 6, 7, 8, 10, 11, 12, 13
		Scabbed area, Face	4, 5, 6, 7, 8, 10, 11, 12, 13
	3003	Normal	-1, 1, 2, 3, 4, 5, 6, 7, 8, 10, 11, 12, 13
		Loss of skin elasticity, Entire body	9
	3004	Normal	-1, 1, 2, 3, 4, 5, 6, 7, 8, 9
		Hair discolored, Cervical region, Red	10, 11, 12, 13
	3005	Normal	-1, 1, 2, 3, 4, 5, 6, 7, 8, 9, 10, 11, 12, 13

Values=Interval seen

2759-001
A Single Dose Toxicity Study of Test Article Administered by Intrathecal Injection in Rats

Individual Detailed Clinical Observations

Sex: Male	Animal	Observation Type: Routine	From Week -1 (Start Date) to 13 (Start Date)
Mid Dose	3006	Normal	-1, 1
	3007	Normal	-1, 1
	3008	Normal	-1, 1, 2
	3009	Normal	-1, 1, 2
	3010	Normal	-1, 1, 2
	3011	Normal	-1, 1, 2
		Ear/portion of ear missing, Ear/left	3, 4, 5
	3012	Normal	-1, 1, 2, 3, 4, 5
	3013	Normal	-1, 1, 2
		Ear/portion of ear missing, Ear/right	3, 4, 5
	3014	Normal	-1, 1, 2, 3, 4, 5
	3015	Normal	-1, 1, 2, 3, 4, 5
High Dose	4001	Normal	-1, 1, 2, 3, 4
		Ear/portion of ear missing, Ear/left	7, 8, 9, 10, 11, 12, 13
		Hair sparse, Face	5, 6, 7, 8, 9, 10, 11, 12, 13
		Scabbed area, Face	9, 10, 11, 12, 13
	4002	Normal	-1, 1, 2, 3, 4, 5, 6, 7, 8, 9, 10
		Hair discolored, Face, Brown	11, 12, 13
	4003	Normal	-1, 1, 2, 3, 4, 5, 6, 7, 8, 9, 10, 11, 12, 13
	4004	Normal	-1, 1, 2, 3, 4, 5, 6, 7, 8, 9, 10, 11, 12, 13
	4005	Normal	-1, 1, 2, 3, 4, 5, 6, 7, 8, 9, 10
		Hair discolored, Face, Red	11, 12, 13
	4006	Normal	-1, 1, 2
	4007	Normal	-1, 1, 2
	4008	Normal	-1, 1, 2
	4009	Normal	-1, 1, 2
	4010	Normal	-1, 1, 2
	4011	Normal	-1, 1, 2, 3, 4, 5
	4012	Normal	-1, 1, 2, 3, 4, 5

Values=Interval seen

2759-001
A Single Dose Toxicity Study of Test Article Administered by Intrathecal Injection in Rats

Individual Detailed Clinical Observations

Sex: Male	Animal	Observation Type: Routine	From Week -1 (Start Date) to 13 (Start Date)
High Dose	4013	Normal	-1, 1, 2
		Hair sparse, Face	3, 4, 5
		Scabbed area, Face	5
	4014	Normal	-1, 1, 2, 3, 4, 5
	4015	Normal	-1, 1, 2
		Hair sparse, Face	3, 4, 5
		Scabbed area, Face	5

Values=Interval seen

2759-001
A Single Dose Toxicity Study of Test Article Administered by Intrathecal Injection in Rats

Individual Detailed Clinical Observations

Sex: Female	Animal	Observation Type: Routine	From Week -1 (Start Date) to 13 (Start Date)
Vehicle	1501	Normal	-1, 1, 2, 3, 4, 5, 6, 7, 8, 10, 11, 12, 13
	1502	Normal	-1, 1, 2, 3, 4, 5, 6, 8, 10, 11, 12, 13
		Hair sparse, Face	13
		Hair sparse, Lumbar region	7
	1503	Normal	-1, 1, 2, 3, 4, 5, 6, 7, 8, 10, 11, 12, 13
	1504	Normal	-1, 1, 2, 3, 4, 5, 6
		Hair discolored, Cervical region, Red	7, 8, 9, 10, 11, 12, 13
		Hair discolored, Face, Red	9, 10, 11, 12, 13
		Loss of skin elasticity	7
	1505	Normal	-1, 1, 2, 3, 4, 5, 6, 8, 9, 10, 11, 12, 13
		Loss of skin elasticity	7
	1506	Normal	-1, 1
	1507	Normal	-1, 1
	1508	Normal	-1, 1
	1509	Normal	-1, 1, 2
	1510	Normal	-1, 1, 2
	1511	Normal	-1, 1, 2, 3, 4, 5
	1512	Normal	-1, 1, 2, 3, 4, 5
	1513	Normal	-1, 1, 2, 3, 4, 5
	1514	Normal	-1, 1, 2, 3, 4, 5
	1515	Normal	-1, 1, 2, 3, 4, 5
Low Dose	2501	Normal	-1, 1, 2, 3, 4, 5, 6, 7, 8
		Hair discolored, Face, Red	10, 11, 12, 13
	2502	Normal	-1, 1, 2, 3, 4, 5, 6, 7, 8, 13
		Hair discolored, Cervical region, Red	10, 11, 12, 13
	2503	Normal	-1, 1, 2, 3, 4, 5, 6, 7, 8, 9
		Hair discolored, Cervical region, Red	10, 11, 12, 13
	2504	Normal	-1, 1, 2, 3, 4, 5, 6, 7, 8, 9, 10, 11
		Hair discolored, Cervical region, Red	12, 13

Values=Interval seen

2759-001
A Single Dose Toxicity Study of Test Article Administered by Intrathecal Injection in Rats

Individual Detailed Clinical Observations

Sex: Female	Animal	Observation Type: Routine	From Week -1 (Start Date) to 13 (Start Date)
Low Dose	2505	Normal	-1, 1, 2, 3, 4, 5, 6, 7, 8, 9
		Hair discolored, Face, Brown	10, 11, 12, 13
	2506	Normal	-1, 1
	2507	Normal	-1, 1
	2508	Normal	-1, 1, 2
	2509	Normal	-1, 1, 2
	2510	Normal	-1, 1, 2
	2511	Normal	-1, 1, 2, 3, 4, 5
	2512	Normal	-1, 1, 2, 3, 4, 5
	2513	Normal	-1, 1, 2, 3, 4, 5
	2514	Normal	-1, 1, 2, 3, 4, 5
	2515	Normal	-1, 1, 2, 3, 4, 5
Mid Dose	3501	Normal	-1, 1, 2, 3, 4, 5, 7, 8
		Hair discolored, Cervical region, Red	12, 13
		Hair sparse, Face	6, 10, 11, 12, 13
		Nodule, Abdominal region, 5-20 mm	10, 11, 12, 13
		Scabbed area, Face	6
	3502	Normal	-1, 1, 2, 3, 4, 5, 6, 7, 8, 10, 11
		Hair discolored, Cervical region, Red	12, 13
	3503	Normal	-1, 1, 2, 3, 5, 6, 7, 8, 9, 10, 11, 12, 13
		Vocalization	4
	3504	Normal	-1, 1, 2, 3, 4, 5, 6, 7, 8, 12, 13
		Hair discolored, Cervical region, Red	9, 10, 11
		Scabbed area, Ear/right	9
	3505	Normal	-1, 1, 2, 3, 4, 5, 6, 7, 8, 9, 10, 11, 12, 13
	3506	Normal	-1, 1
	3507	Normal	-1, 1
	3508	Normal	-1, 1
		Hair sparse, Cervical region	2

Values=Interval seen

2759-001
A Single Dose Toxicity Study of Test Article Administered by Intrathecal Injection in Rats

Individual Detailed Clinical Observations

Sex: Female	Animal	Observation Type: Routine	From Week -1 (Start Date) to 13 (Start Date)
Mid Dose	3509	Normal	-1, 1, 2
	3510	Normal	-1, 1, 2
	3511	Normal	-1, 1, 2, 3, 4, 5
	3512	Normal	-1, 1, 2, 3, 4, 5
	3513	Normal	-1, 1, 2, 3, 4, 5
	3514	Normal	-1, 1, 2, 3, 4, 5
	3515	Normal	-1, 1, 3, 4
		Hair discolored, Face, Red	5
		Scabbed area, Lumbar region	2
High Dose	4501	Normal	-1, 1, 2, 3, 4, 5, 6, 7
		Stereotypy, Straub tail	8, 10, 11, 12, 13
	4502	Normal	-1, 1, 2, 3, 4, 5, 6, 7, 8, 10, 11, 12, 13
	4503	Normal	-1, 1, 2, 3, 4, 5, 6, 7, 8, 10, 11
		Vocalization	9
		Hair discolored, Cervical region, Red	12, 13
	4504	Normal	-1, 1, 2, 4, 5, 6, 7, 8, 9, 10, 11, 12, 13
		Scabbed area, Thoracic region	3
	4505	Normal	-1, 1, 2, 3, 4, 5, 6, 7, 8, 9, 10, 11
		Hair discolored, Cervical region, Red	12, 13
	4506	Normal	-1, 1
	4507	Normal	-1, 1
	4508	Normal	-1, 1, 2
	4509	Normal	-1, 1, 2
	4510	Normal	-1, 1, 2
	4511	Normal	-1, 1, 2, 3, 4, 5
	4512	Normal	-1, 1, 2, 3, 4, 5
	4513	Normal	-1, 1, 2, 3, 4, 5
	4514	Normal	-1, 1, 2, 3, 4, 5
	4515	Normal	-1, 1, 2, 3, 4

Values=Interval seen

2759-001
A Single Dose Toxicity Study of Test Article Administered by Intrathecal Injection in Rats

Individual Detailed Clinical Observations

Sex: Female	Animal	Observation Type: Routine	From Week -1 (Start Date) to 13 (Start Date)
High Dose	4515	Hair discolored, Face, Red	5

Values=Interval seen

2759-001
A Single Dose Toxicity Study of Test Article Administered by Intrathecal Injection in Rats

Individual Detailed Clinical Observations

Sex: Male	Animal	Observation Type: Unscheduled	From Week -1 (Start Date) to 13 (Start Date)
Vehicle	1001	Hair sparse, Face	6
		Scabbed area, Face	6
	1002	Tail bent	6
		Hair sparse, Face	6
		Scabbed area, Face	6
Mid Dose	3002	Hair sparse, Face	6
		Scabbed area, Face	6
	3011	Ear/portion of ear missing, Ear/left	3

Values=Interval seen

2759-001
A Single Dose Toxicity Study of Test Article Administered by Intrathecal Injection in Rats

Individual Detailed Clinical Observations

Sex: Female	Animal	Observation Type: Unscheduled	From Week -1 (Start Date) to 13 (Start Date)
Mid Dose	3501	Swelling, Thoracic region	8
	3504	Discharge, Tail, Red	1
		Swelling, Tail	1

Values=Interval seen

Appendix 5
Individual Body Weight Values

2759-001
A Single Dose Toxicity Study of Test Article Administered by Intrathecal Injection in Rats

Individual Body Weight Values

Sex: Male Body Weight (g)

Vehicle	Day(s) Relative to Start Date													
	-1	1	8	15	22	29	36	43	50	60	64	71	78	85
1001	226	234	286	333	369	401	409	442	454	-	435	483	499	508
1002	255	260	330	374	413	451	471	487	509	-	481	526	541	561
1003	290	291	334	370	397	424	450	477	491	509	513	529	534	547
1004	285	289	339	371	405	426	440	459	477	495	497	500	510	518
1005	279	279	332	367	393	414	432	461	488	495	503	512	516	536
1006	275	286	330[a]	-	-	-	-	-	-	-	-	-	-	-
1007	267	275	306[a]	-	-	-	-	-	-	-	-	-	-	-
1008	278	280	323	-	-	-	-	-	-	-	-	-	-	-
1009	282	284	334	-	-	-	-	-	-	-	-	-	-	-
1010	293	289	351	-	-	-	-	-	-	-	-	-	-	-
1011	313	323	375	414	450	456	-	-	-	-	-	-	-	-
1012	285	298	358	407	450	451	-	-	-	-	-	-	-	-
1013	293	303	356	393	434	437	-	-	-	-	-	-	-	-
1014	306	311	363	397	434	432	-	-	-	-	-	-	-	-
1015	297	304	372	410	455	461	-	-	-	-	-	-	-	-

[a] Body weight recorded was fasted, necropsy weight.

2759-001
A Single Dose Toxicity Study of Test Article Administered by Intrathecal Injection in Rats

Individual Body Weight Values

Sex: Male Body Weight (g)

Vehicle	Day(s) Relative to Start<br>91
1001	490
1002	543
1003	530
1004	508
1005	525
1006	-
1007	-
1008	-
1009	-
1010	-
1011	-
1012	-
1013	-
1014	-
1015	-

2759-001
A Single Dose Toxicity Study of Test Article Administered by Intrathecal Injection in Rats

Individual Body Weight Values

Sex: Male Body Weight (g)

Low Dose	Day(s) Relative to Start Date													
	-1	1	8	15	22	29	36	43	50	57	64	71	78	85
2001	245	249	294	333	358	379	398	422	440	-	475	486	501	513
2002	259	267	318	366	397	431	457	483	503	-	543	551	577	601
2003	233	239	290	328	363	391	410	435	439	-	437	439	452	466
2004	275	282	351	268	386	433	473	496	523	541	557	578	591	578
2005	253	268	325	254	362	392	411	418	434	445	458	475	486	500
2006	272	277	304[a]	-	-	-	-	-	-	-	-	-	-	-
2007	259	270	303[a]	-	-	-	-	-	-	-	-	-	-	-
2008	283	292	334[a]	-	-	-	-	-	-	-	-	-	-	-
2009	242	246	301	-	-	-	-	-	-	-	-	-	-	-
2010	254	259	318	-	-	-	-	-	-	-	-	-	-	-
2011	305	314	359	406	445	443	-	-	-	-	-	-	-	-
2012	286	300	358	393	438	446	-	-	-	-	-	-	-	-
2013	305	309	372	408	443	456	-	-	-	-	-	-	-	-
2014	294	300	346	384	410	411	-	-	-	-	-	-	-	-
2015	313	320	375	424	460	459	-	-	-	-	-	-	-	-

[a] Body weight recorded was fasted, necropsy weight.

2759-001
A Single Dose Toxicity Study of Test Article Administered by Intrathecal Injection in Rats

Individual Body Weight Values

Sex: Male Body Weight (g)

Low Dose	Day(s) Relative to Start<br>91
2001	498
2002	581
2003	430
2004	559
2005	477
2006	-
2007	-
2008	-
2009	-
2010	-
2011	-
2012	-
2013	-
2014	-
2015	-

2759-001
A Single Dose Toxicity Study of Test Article Administered by Intrathecal Injection in Rats

Individual Body Weight Values

Sex: Male Body Weight (g)

Mid Dose	Day(s) Relative to Start Date													
	-1	1	8	15	22	29	36	43	50	60	64	71	78	85
3001	237	240	295	332	363	395	424	447	463	-	431	489	507	524
3002	257	265	323	365	405	431	453	481	495	-	472	524	547	562
3003	297	305	358	413	458	497	513	529	553	513	569	592	603	617
3004	261	271	319	365	392	428	457	478	505	463	517	538	548	570
3005	300	306	357	397	439	474	497	520	551	517	573	593	620	638
3006	278	292	324 [a]	-	-	-	-	-	-	-	-	-	-	-
3007	273	282	314 [a]	-	-	-	-	-	-	-	-	-	-	-
3008	290	294	352	-	-	-	-	-	-	-	-	-	-	-
3009	280	282	331	-	-	-	-	-	-	-	-	-	-	-
3010	278	280	328	-	-	-	-	-	-	-	-	-	-	-
3011	301	308	351	381	411	406	-	-	-	-	-	-	-	-
3012	281	284	326	366	388	384	-	-	-	-	-	-	-	-
3013	328	336	399	448	486	488	-	-	-	-	-	-	-	-
3014	261	281	348	415	465	468	-	-	-	-	-	-	-	-
3015	234	246	297	333	371	378	-	-	-	-	-	-	-	-

[a] Body weight recorded was fasted, necropsy weight.

2759-001
A Single Dose Toxicity Study of Test Article Administered by Intrathecal Injection in Rats

Individual Body Weight Values

Sex: Male Body Weight (g)

Mid Dose	Day(s) Relative to Start<br>91
3001	503
3002	538
3003	605
3004	561
3005	625
3006	-
3007	-
3008	-
3009	-
3010	-
3011	-
3012	-
3013	-
3014	-
3015	-

2759-001
A Single Dose Toxicity Study of Test Article Administered by Intrathecal Injection in Rats

Individual Body Weight Values

Sex: Male Body Weight (g)

High Dose	Day(s) Relative to Start Date													
	-1	1	8	15	22	29	36	43	50	57	60	64	71	78
4001	315	312	381	437	480	518	548	569	591	-	609	618	641	648
4002	288	286	342	380	411	423	444	466	490	-	487	496	507	508
4003	258	264	311	344	377	397	417	433	449	-	466	472	484	493
4004	268	283	340	384	441	480	515	533	583	600	-	626	648	669
4005	254	263	318	360	399	425	458	480	510	532	-	551	566	576
4006	275	275	306	-	-	-	-	-	-	-	-	-	-	-
4007	310	308	345	-	-	-	-	-	-	-	-	-	-	-
4008	286	291	331	-	-	-	-	-	-	-	-	-	-	-
4009	268	274	333	-	-	-	-	-	-	-	-	-	-	-
4010	256	261	314	-	-	-	-	-	-	-	-	-	-	-
4011	311	320	365	401	450	443	-	-	-	-	-	-	-	-
4012	321	331	416	493	558	579	-	-	-	-	-	-	-	-
4013	267	277	336	383	414	421	-	-	-	-	-	-	-	-
4014	258	268	327	371	409	417	-	-	-	-	-	-	-	-
4015	250	261	307	341	372	367	-	-	-	-	-	-	-	-

2759-001
A Single Dose Toxicity Study of Test Article Administered by Intrathecal Injection in Rats

Individual Body Weight Values

Sex: Male Body Weight (g)

High Dose	Day(s) Relative to Start Date	
	85	91
4001	667	648
4002	536	514
4003	505	500
4004	686	674
4005	593	581
4006	-	-
4007	-	-
4008	-	-
4009	-	-
4010	-	-
4011	-	-
4012	-	-
4013	-	-
4014	-	-
4015	-	-

2759-001
A Single Dose Toxicity Study of Test Article Administered by Intrathecal Injection in Rats

Individual Body Weight Values

Sex: Female Body Weight (g)

Vehicle	Day(s) Relative to Start Date													
	-1	1	8	15	22	29	36	43	50	57	64	71	78	85
1501	179	177	207	223	246	267	270	266	287	-	299	285	291	282
1502	174	178	208	231	246	254	264	275	279	-	289	293	302	299
1503	171	170	194	222	243	262	274	286	292	-	304	309	317	322
1504	176	185	198	217	219	225	228	175	239	241	248	249	252	248
1505	196	207	223	239	247	267	284	207	284	303	313	330	328	328
1506	184	192	191 [a]	-	-	-	-	-	-	-	-	-	-	-
1507	202	201	196 [a]	-	-	-	-	-	-	-	-	-	-	-
1508	169	174	173 [a]	-	-	-	-	-	-	-	-	-	-	-
1509	189	200	215	-	-	-	-	-	-	-	-	-	-	-
1510	186	187	200	-	-	-	-	-	-	-	-	-	-	-
1511	195	194	225	229	253	252	-	-	-	-	-	-	-	-
1512	192	191	224	239	260	259	-	-	-	-	-	-	-	-
1513	183	180	208	226	243	228	-	-	-	-	-	-	-	-
1514	192	202	221	238	260	256	-	-	-	-	-	-	-	-
1515	188	183	209	227	237	230	-	-	-	-	-	-	-	-

[a] Body weight recorded was fasted, necropsy weight.

2759-001
A Single Dose Toxicity Study of Test Article Administered by Intrathecal Injection in Rats

Individual Body Weight Values

Sex: Female Body Weight (g)

Vehicle	Day(s) Relative to Start 91
1501	286
1502	294
1503	300
1504	247
1505	307
1506	-
1507	-
1508	-
1509	-
1510	-
1511	-
1512	-
1513	-
1514	-
1515	-

2759-001
A Single Dose Toxicity Study of Test Article Administered by Intrathecal Injection in Rats

Individual Body Weight Values

Sex: Female Body Weight (g)

Low Dose	Day(s) Relative to Start Date													
	-1	1	8	15	22	29	36	43	50	60	64	71	78	85
2501	173	174	203	223	235	249	243	256	276	-	262	291	300	318
2502	168	169	200	217	231	237	244	252	264	-	256	293	296	310
2503	188	185	203	224	228	237	249	250	259	255	261	268	277	271
2504	183	182	191	205	200	218	232	228	225	246	238	245	243	252
2505	200	201	222	240	240	256	271	268	265	286	280	284	291	298
2506	193	193	206[a]	-	-	-	-	-	-	-	-	-	-	-
2507	207	208	216[a]	-	-	-	-	-	-	-	-	-	-	-
2508	188	189	200	-	-	-	-	-	-	-	-	-	-	-
2509	180	180	185	-	-	-	-	-	-	-	-	-	-	-
2510	189	186	198	-	-	-	-	-	-	-	-	-	-	-
2511	189	193	205	220	229	219	-	-	-	-	-	-	-	-
2512	198	200	232	252	258	253	-	-	-	-	-	-	-	-
2513	173	174	191	199	209	205	-	-	-	-	-	-	-	-
2514	198	195	216	236	253	229	-	-	-	-	-	-	-	-
2515	176	179	202	215	226	221	-	-	-	-	-	-	-	-

[a] Body weight recorded was fasted, necropsy weight.

2759-001
A Single Dose Toxicity Study of Test Article Administered by Intrathecal Injection in Rats

Individual Body Weight Values

Sex: Female Body Weight (g)

Low Dose	Day(s) Relative to Start<br>91
2501	294
2502	291
2503	266
2504	248
2505	289
2506	-
2507	-
2508	-
2509	-
2510	-
2511	-
2512	-
2513	-
2514	-
2515	-

2759-001
A Single Dose Toxicity Study of Test Article Administered by Intrathecal Injection in Rats

Individual Body Weight Values

Sex: Female Body Weight (g)

Mid Dose	Day(s) Relative to Start Date													
	-1	1	8	15	22	29	36	43	50	57	64	71	78	85
3501	165	169	199	215	232	231	262	272	271	-	281	283	290	298
3502	178	183	206	235	255	271	273	271	292	-	309	306	313	323
3503	182	190	212	232	232	241	244	250	259	273	267	272	280	278
3504	191	203	215	229	242	253	261	253	268	272	282	270	273	279
3505	180	182	206	221	238	238	246	257	267	266	268	269	273	277
3506	191	198	204 [a]	-	-	-	-	-	-	-	-	-	-	-
3507	191	187	193 [a]	-	-	-	-	-	-	-	-	-	-	-
3508	183	185	193	-	-	-	-	-	-	-	-	-	-	-
3509	187	191	206	-	-	-	-	-	-	-	-	-	-	-
3510	183	192	204	-	-	-	-	-	-	-	-	-	-	-
3511	189	189	212	219	237	240	-	-	-	-	-	-	-	-
3512	171	164	191	198	206	198	-	-	-	-	-	-	-	-
3513	190	188	214	219	239	235	-	-	-	-	-	-	-	-
3514	170	176	194	210	219	207	-	-	-	-	-	-	-	-
3515	201	197	234	252	267	255	-	-	-	-	-	-	-	-

[a] Body weight recorded was fasted, necropsy weight.

2759-001
A Single Dose Toxicity Study of Test Article Administered by Intrathecal Injection in Rats

Individual Body Weight Values

Sex: Female Body Weight (g)

Mid Dose	Day(s) Relative to Start 91
3501	284
3502	309
3503	271
3504	265
3505	269
3506	-
3507	-
3508	-
3509	-
3510	-
3511	-
3512	-
3513	-
3514	-
3515	-

2759-001
A Single Dose Toxicity Study of Test Article Administered by Intrathecal Injection in Rats

Individual Body Weight Values

Sex: Female Body Weight (g)

High Dose	Day(s) Relative to Start Date													
	-1	1	8	15	22	29	36	43	50	60	64	71	78	85
4501	172	178	209	223	241	260	258	287	275	-	270	283	300	329
4502	186	189	215	235	255	278	289	305	308	-	297	302	338	358
4503	186	190	210	229	249	268	272	273	295	297	295	296	302	308
4504	193	196	219	238	261	275	293	300	314	323	326	326	342	343
4505	188	186	211	241	250	253	273	277	285	298	295	296	304	308
4506	176	185	176[a]	-	-	-	-	-	-	-	-	-	-	-
4507	180	183	169[a]	-	-	-	-	-	-	-	-	-	-	-
4508	189	187	199	-	-	-	-	-	-	-	-	-	-	-
4509	199	197	210	-	-	-	-	-	-	-	-	-	-	-
4510	202	201	207	-	-	-	-	-	-	-	-	-	-	-
4511	191	197	233	255	263	250	-	-	-	-	-	-	-	-
4512	185	190	208	228	251	238	-	-	-	-	-	-	-	-
4513	177	182	202	218	230	213	-	-	-	-	-	-	-	-
4514	193	195	215	231	261	257	-	-	-	-	-	-	-	-
4515	172	171	191	200	195	200	-	-	-	-	-	-	-	-

[a] Body weight recorded was fasted, necropsy weight.

2759-001
A Single Dose Toxicity Study of Test Article Administered by Intrathecal Injection in Rats

Individual Body Weight Values

Sex: Female Body Weight (g)

High Dose	Day(s) Relative to Start<br>91
4501	308
4502	337
4503	287
4504	331
4505	294
4506	-
4507	-
4508	-
4509	-
4510	-
4511	-
4512	-
4513	-
4514	-
4515	-

Appendix 6
Individual Body Weight Change Values

2759-001
A Single Dose Toxicity Study of Test Article Administered by Intrathecal Injection in Rats

Individual Body Weight Change Values, g

Sex: Male Body Weight Change

Vehicle	Day(s) Relative to Start Date													
	-1 → 1	1 → 8	8 → 15	15 → 22	22 → 29	29 → 36	36 → 43	43 → 50	50 → 60	60 → 64	50 → 64	64 → 71	71 → 78	78 → 85
1001	8	52	47	36	32	8	33	12	-	-	-19	48	16	9
1002	5	70	44	39	38	20	16	22	-	-	-28	45	15	20
1003	1	43	36	27	27	26	27	14	18	4	-	16	5	13
1004	4	50	32	34	21	14	19	18	18	2	-	3	10	8
1005	0	53	35	26	21	18	29	27	7	8	-	9	4	20
1006	11	44[a]	-	-	-	-	-	-	-	-	-	-	-	-
1007	8	31[a]	-	-	-	-	-	-	-	-	-	-	-	-
1008	2	43	-	-	-	-	-	-	-	-	-	-	-	-
1009	2	50	-	-	-	-	-	-	-	-	-	-	-	-
1010	-4	62	-	-	-	-	-	-	-	-	-	-	-	-
1011	10	52	39	36	6	-	-	-	-	-	-	-	-	-
1012	13	60	49	43	1	-	-	-	-	-	-	-	-	-
1013	10	53	37	41	3	-	-	-	-	-	-	-	-	-
1014	5	52	34	37	-2	-	-	-	-	-	-	-	-	-
1015	7	68	38	45	6	-	-	-	-	-	-	-	-	-

[a] Day 8 body weight recorded was fasted, necropsy weight.

2759-001
A Single Dose Toxicity Study of Test Article Administered by Intrathecal Injection in Rats

Individual Body Weight Change Values, g

Sex: Male Body Weight Change

Vehicle	Day(s) Relative to Start<br>85 → 91
1001	-18
1002	-18
1003	-17
1004	-10
1005	-11
1006	-
1007	-
1008	-
1009	-
1010	-
1011	-
1012	-
1013	-
1014	-
1015	-

2759-001
A Single Dose Toxicity Study of Test Article Administered by Intrathecal Injection in Rats

Individual Body Weight Change Values, g

Sex: Male Body Weight Change

Low Dose	Day(s) Relative to Start Date												
	-1 → 1	1 → 8	8 → 15	15 → 22	22 → 29	29 → 36	36 → 43	43 → 50	50 → 57	57 → 64	50 → 64	64 → 71	71 → 78	78 → 85
2001	4	45	39	25	21	19	24	18	-	-	35	11	15	12
2002	8	51	48	31	34	26	26	20	-	-	40	8	26	24
2003	6	51	38	35	28	19	25	4	-	-	-2	2	13	14
2004	7	69	-83	118	47	40	23	27	18	16	-	21	13	-13
2005	15	57	-71	108	30	19	7	16	11	13	-	17	11	14
2006	5	27[a]	-	-	-	-	-	-	-	-	-	-	-	-
2007	11	33[a]	-	-	-	-	-	-	-	-	-	-	-	-
2008	9	42[a]	-	-	-	-	-	-	-	-	-	-	-	-
2009	4	55	-	-	-	-	-	-	-	-	-	-	-	-
2010	5	59	-	-	-	-	-	-	-	-	-	-	-	-
2011	9	45	47	39	-2	-	-	-	-	-	-	-	-	-
2012	14	58	35	45	8	-	-	-	-	-	-	-	-	-
2013	4	63	36	35	13	-	-	-	-	-	-	-	-	-
2014	6	46	38	26	1	-	-	-	-	-	-	-	-	-
2015	7	55	49	36	-1	-	-	-	-	-	-	-	-	-

[a] Day 8 body weight recorded was fasted, necropsy weight.

2759-001
A Single Dose Toxicity Study of Test Article Administered by Intrathecal Injection in Rats

Individual Body Weight Change Values, g

Sex: Male Body Weight Change

Low Dose	Day(s) Relative to Start
	85 → 91
2001	-15
2002	-20
2003	-36
2004	-19
2005	-23
2006	-
2007	-
2008	-
2009	-
2010	-
2011	-
2012	-
2013	-
2014	-
2015	-

2759-001
A Single Dose Toxicity Study of Test Article Administered by Intrathecal Injection in Rats

Individual Body Weight Change Values, g

Sex: Male Body Weight Change

Mid Dose	Day(s) Relative to Start Date													
	-1 → 1	1 → 8	8 → 15	15 → 22	22 → 29	29 → 36	36 → 43	43 → 50	50 → 60	60 → 64	50 → 64	64 → 71	71 → 78	78 → 85
3001	3	55	37	31	32	29	23	16	-	-	-32	58	18	17
3002	8	58	42	40	26	22	28	14	-	-	-23	52	23	15
3003	8	53	55	45	39	16	16	24	-40	56	-	23	11	14
3004	10	48	46	27	36	29	21	27	-42	54	-	21	10	22
3005	6	51	40	42	35	23	23	31	-34	56	-	20	27	18
3006	14	32[a]	-	-	-	-	-	-	-	-	-	-	-	-
3007	9	32[a]	-	-	-	-	-	-	-	-	-	-	-	-
3008	4	58	-	-	-	-	-	-	-	-	-	-	-	-
3009	2	49	-	-	-	-	-	-	-	-	-	-	-	-
3010	2	48	-	-	-	-	-	-	-	-	-	-	-	-
3011	7	43	30	30	-5	-	-	-	-	-	-	-	-	-
3012	3	42	40	22	-4	-	-	-	-	-	-	-	-	-
3013	8	63	49	38	2	-	-	-	-	-	-	-	-	-
3014	20	67	67	50	3	-	-	-	-	-	-	-	-	-
3015	12	51	36	38	7	-	-	-	-	-	-	-	-	-

[a] Day 8 body weight recorded was fasted, necropsy weight.

2759-001
A Single Dose Toxicity Study of Test Article Administered by Intrathecal Injection in Rats

Individual Body Weight Change Values, g

Sex: Male Body Weight Change

Mid Dose	Day(s) Relative to Start<br>85 → 91
3001	-21
3002	-24
3003	-12
3004	-9
3005	-13
3006	-
3007	-
3008	-
3009	-
3010	-
3011	-
3012	-
3013	-
3014	-
3015	-

2759-001
A Single Dose Toxicity Study of Test Article Administered by Intrathecal Injection in Rats

Individual Body Weight Change Values, g

Sex: Male Body Weight Change

High Dose	Day(s) Relative to Start Date													
	-1 → 1	1 → 8	8 → 15	15 → 22	22 → 29	29 → 36	36 → 43	43 → 50	50 → 57	50 → 60	60 → 64	57 → 64	64 → 71	71 → 78
4001	-3	69	56	43	38	30	21	22	-	18	9	-	23	7
4002	-2	56	38	31	12	21	22	24	-	-3	9	-	11	1
4003	6	47	33	33	20	20	16	16	-	17	6	-	12	9
4004	15	57	44	57	39	35	18	50	17	-	-	26	22	21
4005	9	55	42	39	26	33	22	30	22	-	-	19	15	10
4006	0	31	-	-	-	-	-	-	-	-	-	-	-	-
4007	-2	37	-	-	-	-	-	-	-	-	-	-	-	-
4008	5	40	-	-	-	-	-	-	-	-	-	-	-	-
4009	6	59	-	-	-	-	-	-	-	-	-	-	-	-
4010	5	53	-	-	-	-	-	-	-	-	-	-	-	-
4011	9	45	36	49	-7	-	-	-	-	-	-	-	-	-
4012	10	85	77	65	21	-	-	-	-	-	-	-	-	-
4013	10	59	47	31	7	-	-	-	-	-	-	-	-	-
4014	10	59	44	38	8	-	-	-	-	-	-	-	-	-
4015	11	46	34	31	-5	-	-	-	-	-	-	-	-	-

2759-001
A Single Dose Toxicity Study of Test Article Administered by Intrathecal Injection in Rats

Individual Body Weight Change Values, g

Sex: Male Body Weight Change

High Dose	Day(s) Relative to Start Date	
	78 → 85	85 → 91
4001	19	-19
4002	28	-22
4003	12	-5
4004	17	-12
4005	17	-12
4006	-	-
4007	-	-
4008	-	-
4009	-	-
4010	-	-
4011	-	-
4012	-	-
4013	-	-
4014	-	-
4015	-	-

2759-001
A Single Dose Toxicity Study of Test Article Administered by Intrathecal Injection in Rats

Individual Body Weight Change Values, g

Sex: Female Body Weight Change

Vehicle	Day(s) Relative to Start Date													
	-1 → 1	1 → 8	8 → 15	15 → 22	22 → 29	29 → 36	36 → 43	43 → 50	50 → 57	57 → 64	50 → 64	64 → 71	71 → 78	78 → 85
1501	-2	30	16	23	21	3	-4	21	-	-	12	-14	6	-9
1502	4	30	23	15	8	10	11	4	-	-	10	4	9	-3
1503	-1	24	28	21	19	12	12	6	-	-	12	5	8	5
1504	9	13	19	2	6	3	-53	64	2	7	-	1	3	-4
1505	11	16	16	8	20	17	-77	77	19	10	-	17	-2	0
1506	8	-1[a]	-	-	-	-	-	-	-	-	-	-	-	-
1507	-1	-5[a]	-	-	-	-	-	-	-	-	-	-	-	-
1508	5	-1[a]	-	-	-	-	-	-	-	-	-	-	-	-
1509	11	15	-	-	-	-	-	-	-	-	-	-	-	-
1510	1	13	-	-	-	-	-	-	-	-	-	-	-	-
1511	-1	31	4	24	-1	-	-	-	-	-	-	-	-	-
1512	-1	33	15	21	-1	-	-	-	-	-	-	-	-	-
1513	-3	28	18	17	-15	-	-	-	-	-	-	-	-	-
1514	10	19	17	22	-4	-	-	-	-	-	-	-	-	-
1515	-5	26	18	10	-7	-	-	-	-	-	-	-	-	-

[a] Day 8 body weight recorded was fasted, necropsy weight.

2759-001
A Single Dose Toxicity Study of Test Article Administered by Intrathecal Injection in Rats

Individual Body Weight Change Values, g

Sex: Female Body Weight Change

Vehicle	Day(s) Relative to Start<br>85 → 91
1501	4
1502	-5
1503	-22
1504	-1
1505	-21
1506	-
1507	-
1508	-
1509	-
1510	-
1511	-
1512	-
1513	-
1514	-
1515	-

2759-001
A Single Dose Toxicity Study of Test Article Administered by Intrathecal Injection in Rats

Individual Body Weight Change Values, g

Sex: Female Body Weight Change

Low Dose	Day(s) Relative to Start Date												
	-1 → 1	1 → 8	8 → 15	15 → 22	22 → 29	29 → 36	36 → 43	43 → 50	50 → 60	60 → 64	50 → 64	64 → 71	71 → 78	78 → 85
2501	1	29	20	12	14	-6	13	20	-	-	-14	29	9	18
2502	1	31	17	14	6	7	8	12	-	-	-8	37	3	14
2503	-3	18	21	4	9	12	1	9	-4	6	-	7	9	-6
2504	-1	9	14	-5	18	14	-4	-3	21	-8	-	7	-2	9
2505	1	21	18	0	16	15	-3	-3	21	-6	-	4	7	7
2506	0	13[a]	-	-	-	-	-	-	-	-	-	-	-	-
2507	1	8[a]	-	-	-	-	-	-	-	-	-	-	-	-
2508	1	11	-	-	-	-	-	-	-	-	-	-	-	-
2509	0	5	-	-	-	-	-	-	-	-	-	-	-	-
2510	-3	12	-	-	-	-	-	-	-	-	-	-	-	-
2511	4	12	15	9	-10	-	-	-	-	-	-	-	-	-
2512	2	32	20	6	-5	-	-	-	-	-	-	-	-	-
2513	1	17	8	10	-4	-	-	-	-	-	-	-	-	-
2514	-3	21	20	17	-24	-	-	-	-	-	-	-	-	-
2515	3	23	13	11	-5	-	-	-	-	-	-	-	-	-

[a] Day 8 body weight recorded was fasted, necropsy weight.

2759-001
A Single Dose Toxicity Study of Test Article Administered by Intrathecal Injection in Rats

Individual Body Weight Change Values, g

Sex: Female Body Weight Change

Low Dose	Day(s) Relative to Start<br>85 → 91
2501	-24
2502	-19
2503	-5
2504	-4
2505	-9
2506	-
2507	-
2508	-
2509	-
2510	-
2511	-
2512	-
2513	-
2514	-
2515	-

2759-001
A Single Dose Toxicity Study of Test Article Administered by Intrathecal Injection in Rats

Individual Body Weight Change Values, g

Sex: Female Body Weight Change

Mid Dose	Day(s) Relative to Start Date													
	-1 → 1	1 → 8	8 → 15	15 → 22	22 → 29	29 → 36	36 → 43	43 → 50	50 → 57	57 → 64	50 → 64	64 → 71	71 → 78	78 → 85
3501	4	30	16	17	-1	31	10	-1	-	-	10	2	7	8
3502	5	23	29	20	16	2	-2	21	-	-	17	-3	7	10
3503	8	22	20	0	9	3	6	9	14	-6	-	5	8	-2
3504	12	12	14	13	11	8	-8	15	4	10	-	-12	3	6
3505	2	24	15	17	0	8	11	10	-1	2	-	1	4	4
3506	7	6[a]	-	-	-	-	-	-	-	-	-	-	-	-
3507	-4	6[a]	-	-	-	-	-	-	-	-	-	-	-	-
3508	2	8	-	-	-	-	-	-	-	-	-	-	-	-
3509	4	15	-	-	-	-	-	-	-	-	-	-	-	-
3510	9	12	-	-	-	-	-	-	-	-	-	-	-	-
3511	0	23	7	18	3	-	-	-	-	-	-	-	-	-
3512	-7	27	7	8	-8	-	-	-	-	-	-	-	-	-
3513	-2	26	5	20	-4	-	-	-	-	-	-	-	-	-
3514	6	18	16	9	-12	-	-	-	-	-	-	-	-	-
3515	-4	37	18	15	-12	-	-	-	-	-	-	-	-	-

[a] Day 8 body weight recorded was fasted, necropsy weight.

2759-001
A Single Dose Toxicity Study of Test Article Administered by Intrathecal Injection in Rats

Individual Body Weight Change Values, g

Sex: Female Body Weight Change

Mid Dose	Day(s) Relative to Start<br>85 → 91
3501	-14
3502	-14
3503	-7
3504	-14
3505	-8
3506	-
3507	-
3508	-
3509	-
3510	-
3511	-
3512	-
3513	-
3514	-
3515	-

2759-001
A Single Dose Toxicity Study of Test Article Administered by Intrathecal Injection in Rats

Individual Body Weight Change Values, g

Sex: Female Body Weight Change

High Dose	Day(s) Relative to Start Date													
	-1 → 1	1 → 8	8 → 15	15 → 22	22 → 29	29 → 36	36 → 43	43 → 50	50 → 60	60 → 64	50 → 64	64 → 71	71 → 78	78 → 85
4501	6	31	14	18	19	-2	29	-12	-	-	-5	13	17	29
4502	3	26	20	20	23	11	16	3	-	-	-11	5	36	20
4503	4	20	19	20	19	4	1	22	2	-2	-	1	6	6
4504	3	23	19	23	14	18	7	14	9	3	-	0	16	1
4505	-2	25	30	9	3	20	4	8	13	-3	-	1	8	4
4506	9	-9[a]	-	-	-	-	-	-	-	-	-	-	-	-
4507	3	-14[a]	-	-	-	-	-	-	-	-	-	-	-	-
4508	-2	12	-	-	-	-	-	-	-	-	-	-	-	-
4509	-2	13	-	-	-	-	-	-	-	-	-	-	-	-
4510	-1	6	-	-	-	-	-	-	-	-	-	-	-	-
4511	6	36	22	8	-13	-	-	-	-	-	-	-	-	-
4512	5	18	20	23	-13	-	-	-	-	-	-	-	-	-
4513	5	20	16	12	-17	-	-	-	-	-	-	-	-	-
4514	2	20	16	30	-4	-	-	-	-	-	-	-	-	-
4515	-1	20	9	-5	5	-	-	-	-	-	-	-	-	-

[a] Day 8 body weight recorded was fasted, necropsy weight.

2759-001
A Single Dose Toxicity Study of Test Article Administered by Intrathecal Injection in Rats

Individual Body Weight Change Values, g

Sex: Female Body Weight Change

High Dose	Day(s) Relative to Start
	85 → 91
4501	-21
4502	-21
4503	-21
4504	-12
4505	-14
4506	-
4507	-
4508	-
4509	-
4510	-
4511	-
4512	-
4513	-
4514	-
4515	-

Appendix 7
Individual Caged Food Consumption Values

2759-001
A Single Dose Toxicity Study of Test Article Administered by Intrathecal Injection in Rats

Individual Caged Food Consumption Values

Sex: Male Daily Food Consumption/Animal (g)

Vehicle	No. in Cage	Week(s) Relative to Animal Start Date						
		1	1 → 2	2 → 3	4	3 → 4	4 → 5	5 → 6
1	2	-	25	26	-	23	25	24
2	3	-	25	26	-	26	26	27
3	2	30	-	-	-	-	-	-
4	3	30	-	-	-	-	-	-
5	3	-	29	29	31	29	-	-
6	2	-	29	29	28	30	-	-

2759-001
A Single Dose Toxicity Study of Test Article Administered by Intrathecal Injection in Rats

Individual Caged Food Consumption Values

Sex: Male Daily Food Consumption/Animal (g)

Vehicle	No. in Cage	Week(s) Relative to Animal Start Date						
		6 → 7	7 → 8	8 → 9	9 → 10	10 → 11	11 → 12	13
1	2	25	25	26	23	26	26	25
2	3	27	28	28	28	27	28	28
3	2	-	-	-	-	-	-	-
4	3	-	-	-	-	-	-	-
5	3	-	-	-	-	-	-	-
6	2	-	-	-	-	-	-	-

2759-001
A Single Dose Toxicity Study of Test Article Administered by Intrathecal Injection in Rats

Individual Caged Food Consumption Values

Sex: Male Daily Food Consumption/Animal (g)

Vehicle	No. in Cage	Week(s) Relative to Animal Start Date<br>12 → 13
1	2	26
2	3	28
3	2	-
4	3	-
5	3	-
6	2	-

2759-001
A Single Dose Toxicity Study of Test Article Administered by Intrathecal Injection in Rats

Individual Caged Food Consumption Values

Sex: Male Daily Food Consumption/Animal (g)

Low Dose	No. in Cage	Week(s) Relative to Animal Start Date						
		1	1 → 2	2 → 3	4	3 → 4	4 → 5	5 → 6
13	3	-	23	33	-	23	28	24
14	2	-	30	14	-	33	31	30
15	3	28	-	-	-	-	-	-
16	2	29	-	-	-	-	-	-
17	3	-	29	29	30	29	-	-
18	2	-	29	29	28	29	-	-

2759-001
A Single Dose Toxicity Study of Test Article Administered by Intrathecal Injection in Rats

Individual Caged Food Consumption Values

Sex: Male Daily Food Consumption/Animal (g)

Low Dose	No. in Cage	Week(s) Relative to Animal Start Date						
		6 → 7	7 → 8	8 → 9	9 → 10	10 → 11	11 → 12	13
13	3	25	24	26	26	26	26	27
14	2	30	29	30	31	30	30	28
15	3	-	-	-	-	-	-	-
16	2	-	-	-	-	-	-	-
17	3	-	-	-	-	-	-	-
18	2	-	-	-	-	-	-	-

2759-001
A Single Dose Toxicity Study of Test Article Administered by Intrathecal Injection in Rats

Individual Caged Food Consumption Values

Sex: Male Daily Food Consumption/Animal (g)

Low Dose	No. in Cage	Week(s) Relative to Animal Start Date<br>12 → 13
13	3	26
14	2	25
15	3	-
16	2	-
17	3	-
18	2	-

2759-001
A Single Dose Toxicity Study of Test Article Administered by Intrathecal Injection in Rats

Individual Caged Food Consumption Values

Sex: Male Daily Food Consumption/Animal (g)

Mid Dose	No. in Cage	Week(s) Relative to Animal Start Date						
		1	1 → 2	2 → 3	4	3 → 4	4 → 5	5 → 6
25	2	-	26	27	-	27	27	28
26	3	-	27	28	-	28	29	29
27	2	29	-	-	-	-	-	-
28	3	29	-	-	-	-	-	-
29	3	-	27	28	27	28	-	-
30	2	-	27	29	29	28	-	-

2759-001
A Single Dose Toxicity Study of Test Article Administered by Intrathecal Injection in Rats

Individual Caged Food Consumption Values

Sex: Male Daily Food Consumption/Animal (g)

Mid Dose	No. in Cage	Week(s) Relative to Animal Start Date						
		6 → 7	7 → 8	8 → 9	9 → 10	10 → 11	11 → 12	13
25	2	28	27	28	25	29	30	29
26	3	29	29	29	25	31	30	31
27	2	-	-	-	-	-	-	-
28	3	-	-	-	-	-	-	-
29	3	-	-	-	-	-	-	-
30	2	-	-	-	-	-	-	-

2759-001
A Single Dose Toxicity Study of Test Article Administered by Intrathecal Injection in Rats

Individual Caged Food Consumption Values

Sex: Male Daily Food Consumption/Animal (g)

Mid Dose	No. in Cage	Week(s) Relative to Animal Start Date<br>12 → 13
25	2	29
26	3	31
27	2	-
28	3	-
29	3	-
30	2	-

2759-001
A Single Dose Toxicity Study of Test Article Administered by Intrathecal Injection in Rats

Individual Caged Food Consumption Values

Sex: Male Daily Food Consumption/Animal (g)

High Dose	No. in Cage	Week(s) Relative to Animal Start Date						
		1	1 → 2	2 → 3	4	3 → 4	4 → 5	5 → 6
37	3	-	26	27	-	26	27	27
38	2	-	27	27	-	31	30	32
39	3	28	-	-	-	-	-	-
40	2	29	-	-	-	-	-	-
41	2	-	33	36	38	37	-	-
42	3	-	27	28	27	27	-	-

2759-001
A Single Dose Toxicity Study of Test Article Administered by Intrathecal Injection in Rats

Individual Caged Food Consumption Values

Sex: Male Daily Food Consumption/Animal (g)

High Dose	No. in Cage	Week(s) Relative to Animal Start Date						
		6 → 7	7 → 8	8 → 9	9 → 10	10 → 11	11 → 12	13
37	3	27	28	27	29	27	27	27
38	2	32	32	33	32	32	33	32
39	3	-	-	-	-	-	-	-
40	2	-	-	-	-	-	-	-
41	2	-	-	-	-	-	-	-
42	3	-	-	-	-	-	-	-

2759-001
A Single Dose Toxicity Study of Test Article Administered by Intrathecal Injection in Rats

Individual Caged Food Consumption Values

Sex: Male Daily Food Consumption/Animal (g)

High Dose	No. in Cage	Week(s) Relative to Animal Start Date<br>12 → 13
37	3	27
38	2	31
39	3	-
40	2	-
41	2	-
42	3	-

2759-001
A Single Dose Toxicity Study of Test Article Administered by Intrathecal Injection in Rats

Individual Caged Food Consumption Values

Sex: Female Daily Food Consumption/Animal (g)

Vehicle	No. in Cage	Week(s) Relative to Animal Start Date						
		1	1 → 2	2 → 3	4	3 → 4	4 → 5	5 → 6
7	3	-	18	20	-	20	20	19
8	2	-	18	19	-	18	19	19
9	3	19	-	-	-	-	-	-
10	2	19	-	-	-	-	-	-
11	2	-	20	20	20	21	-	-
12	3	-	17	19	19	18	-	-

2759-001
A Single Dose Toxicity Study of Test Article Administered by Intrathecal Injection in Rats

Individual Caged Food Consumption Values

Sex: Female Daily Food Consumption/Animal (g)

Vehicle	No. in Cage	Week(s) Relative to Animal Start Date						
		6 → 7	7 → 8	8 → 9	9 → 10	10 → 11	11 → 12	13
7	3	19	18	19	19	17	18	20
8	2	7	23	21	19	18	20	17
9	3	-	-	-	-	-	-	-
10	2	-	-	-	-	-	-	-
11	2	-	-	-	-	-	-	-
12	3	-	-	-	-	-	-	-

2759-001
A Single Dose Toxicity Study of Test Article Administered by Intrathecal Injection in Rats

Individual Caged Food Consumption Values

Sex: Female Daily Food Consumption/Animal (g)

Vehicle	No. in Cage	Week(s) Relative to Animal Start Date
		12 → 13
7	3	17
8	2	17
9	3	-
10	2	-
11	2	-
12	3	-

2759-001
A Single Dose Toxicity Study of Test Article Administered by Intrathecal Injection in Rats

Individual Caged Food Consumption Values

Sex: Female Daily Food Consumption/Animal (g)

Low Dose	No. in Cage	Week(s) Relative to Animal Start Date						
		1	1 → 2	2 → 3	4	3 → 4	4 → 5	5 → 6
19	2	-	17	6	-	19	19	19
20	3	-	18	20	-	18	21	21
21	2	19	-	-	-	-	-	-
22	3	17	-	-	-	-	-	-
23	3	-	16	17	16	17	-	-
24	2	-	18	18	18	25	-	-

2759-001
A Single Dose Toxicity Study of Test Article Administered by Intrathecal Injection in Rats

Individual Caged Food Consumption Values

Sex: Female Daily Food Consumption/Animal (g)

Low Dose	No. in Cage	Week(s) Relative to Animal Start Date						
		6 → 7	7 → 8	8 → 9	9 → 10	10 → 11	11 → 12	13
19	2	9 !	24	21	18	21	20	19
20	3	19	19	22	19	19	20	20
21	2	-	-	-	-	-	-	-
22	3	-	-	-	-	-	-	-
23	3	-	-	-	-	-	-	-
24	2	-	-	-	-	-	-	-

! = Result Comment

2759-001
A Single Dose Toxicity Study of Test Article Administered by Intrathecal Injection in Rats

Individual Caged Food Consumption Values

Sex: Female Daily Food Consumption/Animal (g)

Low Dose	No. in Cage	Week(s) Relative to Animal Start Date
		12 → 13
19	2	20
20	3	21
21	2	-
22	3	-
23	3	-
24	2	-

2759-001
A Single Dose Toxicity Study of Test Article Administered by Intrathecal Injection in Rats

Individual Caged Food Consumption Values

Sex: Female Daily Food Consumption/Animal (g)

Mid Dose	No. in Cage	Week(s) Relative to Animal Start Date						
		1	1 → 2	2 → 3	4	3 → 4	4 → 5	5 → 6
31	2	-	18	19	-	20	20	21
32	3	-	18	19	-	18	18	20
33	2	18	-	-	-	-	-	-
34	3	20	-	-	-	-	-	-
35	3	-	18	17	17	18	-	-
36	2	-	19	20	18	19	-	-

2759-001
A Single Dose Toxicity Study of Test Article Administered by Intrathecal Injection in Rats

Individual Caged Food Consumption Values

Sex: Female Daily Food Consumption/Animal (g)

Mid Dose	No. in Cage	Week(s) Relative to Animal Start Date						
		6 → 7	7 → 8	8 → 9	9 → 10	10 → 11	11 → 12	13
31	2	19	18	20	19	18	19	22
32	3	19	19	20	19	18	19	20
33	2	-	-	-	-	-	-	-
34	3	-	-	-	-	-	-	-
35	3	-	-	-	-	-	-	-
36	2	-	-	-	-	-	-	-

2759-001
A Single Dose Toxicity Study of Test Article Administered by Intrathecal Injection in Rats

Individual Caged Food Consumption Values

Sex: Female Daily Food Consumption/Animal (g)

Mid Dose	No. in Cage	Week(s) Relative to Animal Start Date<br>12 → 13
31	2	20
32	3	18
33	2	-
34	3	-
35	3	-
36	2	-

2759-001
A Single Dose Toxicity Study of Test Article Administered by Intrathecal Injection in Rats

Individual Caged Food Consumption Values

Sex: Female Daily Food Consumption/Animal (g)

High Dose	No. in Cage	Week(s) Relative to Animal Start Date						
		1	1 → 2	2 → 3	4	3 → 4	4 → 5	5 → 6
43	2	-	19	21	-	20	21	20
44	3	-	18	21	-	20	21	20
45	2	16	-	-	-	-	-	-
46	3	19	-	-	-	-	-	-
47	3	-	18	20	16	19	-	-
48	2	-	17	19	19	19	-	-

2759-001
A Single Dose Toxicity Study of Test Article Administered by Intrathecal Injection in Rats

Individual Caged Food Consumption Values

Sex: Female Daily Food Consumption/Animal (g)

High Dose	No. in Cage	Week(s) Relative to Animal Start Date						
		6 → 7	7 → 8	8 → 9	9 → 10	10 → 11	11 → 12	13
43	2	23	20	21	19	8	28	22
44	3	20	20	20	19	20	19	18
45	2	-	-	-	-	-	-	-
46	3	-	-	-	-	-	-	-
47	3	-	-	-	-	-	-	-
48	2	-	-	-	-	-	-	-

2759-001
A Single Dose Toxicity Study of Test Article Administered by Intrathecal Injection in Rats

Individual Caged Food Consumption Values

Sex: Female Daily Food Consumption/Animal (g)

High Dose	No. in Cage	Week(s) Relative to Animal Start Date<br>12 → 13
43	2	26
44	3	20
45	2	-
46	3	-
47	3	-
48	2	-

2759-001
A Single Dose Toxicity Study of Test Article Administered by Intrathecal Injection in Rats

Individual Caged Food Consumption Values

Key Page

Cage Contents

Cage Number	Animal Numbers	Cage Number	Animal Numbers
1	1001, 1002	2	1003, 1004, 1005
3	1006, 1007	4	1008, 1009, 1010
5	1011, 1012, 1013	6	1014, 1015
7	1501, 1502, 1503	8	1504, 1505
9	1506, 1507, 1508	10	1509, 1510
11	1511, 1512	12	1513, 1514, 1515
13	2001, 2002, 2003	14	2004, 2005
15	2006, 2007, 2008	16	2009, 2010
17	2011, 2012, 2013	18	2014, 2015
19	2501, 2502	20	2503, 2504, 2505
21	2506, 2507	22	2508, 2509, 2510
23	2511, 2512, 2513	24	2514, 2515
25	3001, 3002	26	3003, 3004, 3005
27	3006, 3007	28	3008, 3009, 3010
29	3011, 3012, 3013	30	3014, 3015
31	3501, 3502	32	3503, 3504, 3505
33	3506, 3507	34	3508, 3509, 3510
35	3511, 3512, 3513	36	3514, 3515
37	4001, 4002, 4003	38	4004, 4005
39	4006, 4007, 4008	40	4009, 4010
41	4011, 4012	42	4013, 4014, 4015
43	4501, 4502	44	4503, 4504, 4505
45	4506, 4507	46	4508, 4509, 4510
47	4511, 4512, 4513	48	4514, 4515

Appendix 8
Individual Rotating Rod Values

2759-001
A Single Dose Toxicity Study of Test Article Administered by Intrathecal Injection in Rats

Individual Rotating Rod Values - Male

Fall Time (sec)

Vehicle	Day -1 (PRDO)				Day 28				Day 90			
	Trial Number				Trial Number				Trial Number			
	1	2	3	**Average**	1	2	3	**Average**	1	2	3	**Average**
1001	47.80	66.75	36.86	**50.47**	-	-	-	**-**	51.11	38.30	42.78	**44.06**
1002	45.76	25.62	51.20	**40.86**	-	-	-	**-**	8.22	4.85	9.67	**7.58**
1003	21.68	17.13	37.22	**25.34**	-	-	-	**-**	35.62	33.03	27.92	**32.19**
1004	39.91	5.63	9.49	**18.34**	-	-	-	**-**	2.36	4.93	2.14	**3.14**
1005	71.95	21.76	57.61	**50.44**	-	-	-	**-**	78.56	49.61	35.32	**54.50**
1011	40.76	29.52	49.90	**40.06**	50.34	68.56	80.29	**66.40**	-	-	-	**-**
1012	56.33	20.87	55.51	**44.24**	67.30	30.55	46.13	**47.99**	-	-	-	**-**
1013	35.69	92.91	92.67	**73.76**	59.11	48.49	96.22	**67.94**	-	-	-	**-**
1014	39.89	25.58	60.47	**41.98**	82.74	69.61	36.69	**63.01**	-	-	-	**-**
1015	46.49	42.50	70.95	**53.31**	99.39	58.30	44.87	**67.52**	-	-	-	**-**

2759-001
A Single Dose Toxicity Study of Test Article Administered by Intrathecal Injection in Rats

Individual Rotating Rod Values - Male

Fall Time (sec)

Low Dose	Day -1 (PRDO)				Day 28				Day 90			
	Trial Number				Trial Number				Trial Number			
	1	2	3	**Average**	1	2	3	**Average**	1	2	3	**Average**
2001	44.29	77.24	45.62	**55.72**	-	-	-	**-**	32.34	7.24	69.67	**36.42**
2002	59.75	78.95	86.94	**75.21**	-	-	-	**-**	28.21	37.52	25.15	**30.29**
2003	54.09	77.33	47.74	**59.72**	-	-	-	**-**	20.31	10.61	8.69	**13.20**
2004	45.55	14.52	61.23	**40.43**	-	-	-	**-**	87.47	51.37	29.86	**56.23**
2005	109.54	118.47	95.86	**107.96**	-	-	-	**-**	82.18	67.07	36.30	**61.85**
2011	55.15	79.23	50.94	**61.77**	42.51	73.91	72.48	**62.97**	-	-	-	**-**
2012	31.68	49.48	67.77	**49.64**	41.19	80.25	26.05	**49.16**	-	-	-	**-**
2013	65.72	11.86	18.50	**32.03**	74.16	33.62	77.31	**61.70**	-	-	-	**-**
2014	43.24	36.52	52.60	**44.12**	69.41	82.42	83.82	**78.55**	-	-	-	**-**
2015	49.89	41.39	20.92	**37.40**	68.91	68.78	71.25	**69.65**	-	-	-	**-**

2759-001
A Single Dose Toxicity Study of Test Article Administered by Intrathecal Injection in Rats

Individual Rotating Rod Values - Male

Fall Time (sec)

Mid Dose	Day -1 (PRDO)				Day 28				Day 90			
	Trial Number				Trial Number				Trial Number			
	1	2	3	**Average**	1	2	3	**Average**	1	2	3	**Average**
3001	95.80	54.34	91.18	**80.44**	-	-	-	**-**	79.98	55.37	140.03	**91.79**
3002	62.24	1.01	47.02	**36.76**	-	-	-	**-**	46.65	62.04	51.14	**53.28**
3003	22.20	46.13	31.26	**33.20**	-	-	-	**-**	4.73	3.60	5.54	**4.62**
3004	49.31	18.38	11.62	**26.44**	-	-	-	**-**	50.73	37.28	48.69	**45.57**
3005	54.21	46.24	33.76	**44.74**	-	-	-	**-**	46.01	3.17	4.06	**17.75**
3011	48.03	19.81	18.60	**28.81**	21.51	79.30	33.57	**44.79**	-	-	-	**-**
3012	82.52	56.85	72.37	**70.58**	63.85	39.87	87.59	**63.77**	-	-	-	**-**
3013	31.79	29.55	34.48	**31.94**	28.10	26.27	62.60	**38.99**	-	-	-	**-**
3014	47.24	82.63	59.09	**62.99**	11.51	6.29	11.37	**9.72**	-	-	-	**-**
3015	46.66	56.94	52.11	**51.90**	25.26	74.41	52.00	**50.56**	-	-	-	**-**

2759-001
A Single Dose Toxicity Study of Test Article Administered by Intrathecal Injection in Rats

Individual Rotating Rod Values - Male

Fall Time (sec)

High Dose	Day -1 (PRDO)				Day 28				Day 90			
	Trial Number				Trial Number				Trial Number			
	1	2	3	**Average**	1	2	3	**Average**	1	2	3	**Average**
4001	10.72	26.19	26.82	**21.24**	-	-	-	**-**	52.18	70.36	35.96	**52.83**
4002	48.25	20.75	74.76	**47.92**	-	-	-	**-**	28.41	56.01	53.27	**45.90**
4003	64.68	81.60	82.73	**76.34**	-	-	-	**-**	34.07	25.12	36.75	**31.98**
4004	74.07	13.31	16.79	**34.72**	-	-	-	**-**	36.91	35.83	36.19	**36.31**
4005	59.97	80.31	44.55	**61.61**	-	-	-	**-**	38.55	47.33	51.75	**45.88**
4011	33.06	40.36	48.86	**40.76**	53.98	62.70	79.97	**65.55**	-	-	-	**-**
4012	37.46	12.53	12.43	**20.81**	22.65	6.88	7.66	**12.40**	-	-	-	**-**
4013	110.50	22.67	107.50	**80.22**	8.00	81.23	49.94	**46.39**	-	-	-	**-**
4014	43.28	60.97	71.81	**58.69**	76.64	29.94	20.28	**42.29**	-	-	-	**-**
4015	66.99	82.29	100.34	**83.21**	103.40	58.42	60.95	**74.26**	-	-	-	**-**

2759-001
A Single Dose Toxicity Study of Test Article Administered by Intrathecal Injection in Rats

Individual Rotating Rod Values - Female

Fall Time (sec)

Vehicle	Day -1 (PRDO)				Day 28				Day 90			
	Trial Number				Trial Number				Trial Number			
	1	2	3	**Average**	1	2	3	**Average**	1	2	3	**Average**
1501	105.10	125.36	117.59	**116.02**	-	-	-	**-**	81.42	81.25	84.21	**82.29**
1502	93.24	38.10	53.85	**61.73**	-	-	-	**-**	70.40	31.90	35.87	**46.06**
1503	20.30	97.30	24.84	**47.48**	-	-	-	**-**	62.92	61.26	32.35	**52.18**
1504	67.66	46.69	62.90	**59.08**	-	-	-	**-**	72.92	102.91	77.84	**84.56**
1505	12.61	66.38	72.96	**50.65**	-	-	-	**-**	64.41	28.53	42.48	**45.14**
1511	89.93	71.20	62.12	**74.42**	22.84	115.39	11.39	**49.87**	-	-	-	**-**
1512	39.03	77.75	19.59	**45.46**	104.63	74.66	71.15	**83.48**	-	-	-	**-**
1513	62.43	96.08	106.92	**88.48**	74.34	70.08	69.19	**71.20**	-	-	-	**-**
1514	113.07	173.13	154.24	**146.81**	36.54	50.11	124.33	**70.33**	-	-	-	**-**
1515	112.84	145.99	130.32	**129.72**	48.44	74.18	27.61	**50.08**	-	-	-	**-**

2759-001
A Single Dose Toxicity Study of Test Article Administered by Intrathecal Injection in Rats

Individual Rotating Rod Values - Female

Fall Time (sec)

Low Dose	Day -1 (PRDO)				Day 28				Day 90			
	Trial Number				Trial Number				Trial Number			
	1	2	3	**Average**	1	2	3	**Average**	1	2	3	**Average**
2501	67.05	95.21	34.82	**65.69**	-	-	-	**-**	75.94	84.41	106.97	**89.11**
2502	103.57	41.31	18.21	**54.36**	-	-	-	**-**	38.80	76.16	28.86	**47.94**
2503	13.31	66.33	89.84	**56.49**	-	-	-	**-**	55.46	63.01	63.57	**60.68**
2504	94.54	54.99	45.68	**65.07**	-	-	-	**-**	96.33	61.34	131.54	**96.40**
2505	52.32	103.16	56.72	**70.73**	-	-	-	**-**	126.77	135.53	88.56	**116.95**
2511	73.37	47.13	74.44	**64.98**	73.01	108.28	109.87	**97.05**	-	-	-	**-**
2512	57.47	74.62	70.20	**67.43**	71.47	62.60	65.36	**66.48**	-	-	-	**-**
2513	72.71	55.35	28.31	**52.12**	88.77	39.92	105.77	**78.15**	-	-	-	**-**
2514	110.92	124.83	55.36	**97.04**	73.98	57.55	69.89	**67.14**	-	-	-	**-**
2515	50.67	35.74	143.01	**76.47**	132.20	165.61	168.96	**155.59**	-	-	-	**-**

2759-001
A Single Dose Toxicity Study of Test Article Administered by Intrathecal Injection in Rats

Individual Rotating Rod Values - Female

Fall Time (sec)

Mid Dose	Day -1 (PRDO)				Day 28				Day 90			
	Trial Number				Trial Number				Trial Number			
	1	2	3	**Average**	1	2	3	**Average**	1	2	3	**Average**
3501	115.21	48.27	27.08	**63.52**	-	-	-	**-**	42.39	70.14	66.47	**59.67**
3502	32.12	25.91	118.14	**58.72**	-	-	-	**-**	38.27	47.88	26.49	**37.55**
3503	88.58	89.86	103.35	**93.93**	-	-	-	**-**	108.47	84.93	43.09	**78.83**
3504	48.91	30.53	9.33	**29.59**	-	-	-	**-**	12.04	9.00	7.66	**9.57**
3505	63.81	88.91	107.94	**86.89**	-	-	-	**-**	40.54	47.89	51.84	**46.76**
3511	16.01	49.14	50.86	**38.67**	53.57	47.02	52.31	**50.97**	-	-	-	**-**
3512	14.32	97.08	76.24	**62.55**	98.81	81.40	109.58	**96.60**	-	-	-	**-**
3513	85.66	100.22	119.01	**101.63**	46.30	49.47	63.71	**53.16**	-	-	-	**-**
3514	103.44	51.75	113.54	**89.58**	61.29	82.15	125.41	**89.62**	-	-	-	**-**
3515	82.85	51.62	48.05	**60.84**	52.51	70.01	64.24	**62.25**	-	-	-	**-**

2759-001
A Single Dose Toxicity Study of Test Article Administered by Intrathecal Injection in Rats

Individual Rotating Rod Values - Female

Fall Time (sec)

High Dose	Day -1 (PRDO)				Day 28				Day 90			
	Trial Number				Trial Number				Trial Number			
	1	2	3	**Average**	1	2	3	**Average**	1	2	3	**Average**
4501	59.59	62.53	28.77	**50.30**	-	-	-	**-**	21.44	28.67	17.25	**22.45**
4502	87.17	115.72	66.52	**89.80**	-	-	-	**-**	74.59	90.62	63.07	**76.09**
4503	70.98	66.68	70.43	**69.36**	-	-	-	**-**	115.08	125.19	154.13	**131.47**
4504	38.81	101.42	39.80	**60.01**	-	-	-	**-**	61.68	36.94	77.22	**58.61**
4505	44.90	91.07	24.71	**53.56**	-	-	-	**-**	68.17	114.68	103.21	**95.35**
4511	15.34	89.08	43.95	**49.46**	81.84	81.11	74.01	**78.99**	-	-	-	**-**
4512	34.26	92.42	78.45	**68.38**	63.52	88.45	137.98	**96.65**	-	-	-	**-**
4513	92.51	127.80	50.17	**90.16**	107.30	94.44	66.82	**89.52**	-	-	-	**-**
4514	73.13	95.80	126.24	**98.39**	94.82	84.77	113.27	**97.62**	-	-	-	**-**
4515	65.65	157.55	65.58	**96.26**	96.94	25.43	32.67	**51.68**	-	-	-	**-**

Appendix 9
Clinical Pathology Report

FINAL REPORT

Study Phase: Clinical Pathology

Testing Facility Study No. 2759-001

Sponsor Reference No. CLN7-001

TESTING FACILITY:
Charles River Laboratories, Inc.

TABLE OF CONTENTS

	Page
TABLE OF CONTENTS	2
REPORT TEXT	3
REPORT APPROVAL	3
1. SUMMARY	4
2. MATERIALS AND METHODS	4
2.1. Group Assignments	4
2.2. Hematology, Coagulation, and Clinical Chemistry	4
2.3. Data Collection and Analysis Software	5
3. RESULTS AND DISCUSSION	6
3.1. Hematology	6
3.2. Coagulation	6
3.3. Clinical Chemistry	7
TABLE	8
1. Summary of Hematology Values	8
2. Summary of Coagulation Values	24
3. Summary of Clinical Chemistry Values	30
APPENDIX	48
1. Individual Hematology Values	48
2. Individual Coagulation Values	90
3. Individual Clinical Chemistry Values	100

REPORT APPROVAL

DocuSigned by:

Caitlyn Carter

Signer Name: Caitlyn Carter
Signing Reason: I approve this document
Signing Time: 18-Mar-2020 | 16:29:33 EDT
48CE3A2EA7874707B68B816148DE0E56

Caitlyn Carter, DVM, DACVP
Clinical Pathologist

1. SUMMARY

This study was conducted for Foundation for Batten Hope to further characterize the toxicity, biodistribution, and gene expression of the test article, scAAV9/JeT-hCLN7opt-SV40pA. The test article is being developed for treatment of the neurodegenerative Batten Disease (CLN7 subtype).

Single intrathecal injections of scAAV9/JeT-hCLN7opt-SV40pA to Sprague Dawley rats at Low Dose ($5x10^{11}$ vg/animal), Mid Dose ($2x10^{12}$ vg/animal) or High Dose ($6x10^{12}$ vg/animal) levels on Day 1 with an observation period through Day 91 resulted in test article-related increases in lymphocyte counts in both sexes at the Mid and High dose levels with concomitant increases in total leukocyte counts, which tended to partially resolve by the end of the observation period except in females at the High Dose levels. Additionally, males administered the High Dose level had a test article-related increase in fibrinogen concentration that had resolved by the end of the observation period. Males administered Mid and High Dose levels and females administered Low, Mid, and High Dose levels also had test article-related increases in globulin concentrations, which had partially to fully resolved by the end of the observation period at the Low and Mid Dose levels but persisted in both sexes at the High Dose level. These changes were collectively suggestive of a minor immune or inflammatory stimulus, which lacked definitive microscopic correlates.

Males administered the Low, Mid, and High Dose levels also had test article-related decreases in triglyceride concentrations that had resolved by the end of the observation period and lacked microscopic correlates.

2. MATERIALS AND METHODS

2.1. Group Assignments

Animals were assigned to the study as indicated in Text Table 1.

Text Table 1
Group Assignments

Group No.	Dose Level (vg/animal)	No. of Animals					
		Necropsy Day 8		Necropsy Day 29		Necropsy Day 91	
		M	F	M	F	M	F
1	0	5	5	5	5	5	5
2	$5x10^{11}$	5	5	5	5	5	5
3	$2x10^{12}$	5	5	5	5	5	5
4	$6x10^{12}$	5	5	5	5	5	5

M - Male
F - Female

2.2. Hematology, Coagulation, and Clinical Chemistry

Clinical pathology evaluations were conducted on all animals at the interval specified in Text Table 2. The animals had access to drinking water but were fasted overnight prior to scheduled sample collection. Blood samples (approximately 2.5 mL to 3.5 mL) were collected via the vena cava after carbon dioxide inhalation. The samples were collected into tubes containing K_2EDTA for evaluation of hematology parameters and sodium citrate for evaluation of coagulation parameters. Blood samples were collected into serum separator tubes without anticoagulant, allowed to clot at controlled room temperature, and processed to serum for evaluation of clinical chemistry parameters. On occasion, blood samples were clotted.

Text Table 2
Clinical Pathology Sample Collection

Panel	Collection Interval	Group 1	Group 2	Group 3	Group 4
Hematology	Terminal	X	X	X	X
Coagulation	Terminal	X	X	X	X
Clinical Chemistry	Terminal	X	X	X	X

X - samples were collected

2.3. Data Collection and Analysis Software

Data Collection and Analysis Software is listed in Text Table 3.

Text Table 3
Data Collection and Analysis Software

Hematology	Advia 2120i v6.9
Coagulation	STA Compact Max v103.04 or STA-R Evolution v3.04
Clinical Chemistry	AU5800 v05.03

3. RESULTS AND DISCUSSION

For the purpose of this report, treated animals' values were compared to control animals' values. Fold change (x) in clinical pathology parameters was determined by comparing the scAAV9/JeT-hCLN7opt-SV40pA group mean to the respective control group mean unless otherwise noted.

3.1. Hematology

(Table 1 and Appendix 1)

On Days 8, 29, and 91 in both sexes at the Mid Dose and High Dose there were minimal to mild increases in mean lymphocyte counts with concomitant increases in mean total leukocyte counts, as illustrated in Text Table 4. These changes tended to be most pronounced on Day 29, except in High Dose females that had the most pronounced change on Day 91. These changes were considered test article-related. Along with increases in fibrinogen concentrations (see Section 3.2) and globulin concentrations (see Section 3.3), these changes were collectively suggestive of a minor immune or inflammatory stimulus, which lacked definitive microscopic correlates.

Text Table 4
scAAV9/JeT-hCLN7opt-SV40pA-Related Hematology Changes

Group / Dose Level / Sex	2 Low Dose M	2 Low Dose F	3 Mid Dose M	3 Mid Dose F	4 High Dose M	4 High Dose F
**Lymphocytes**						
Day 8	–	–	1.24x	1.18x	1.18x	1.16x
Day 29	–	–	1.54x	1.33x	1.71x	1.20x
Day 91	–	–	1.20x	1.30x	1.25x	1.67x
**Total Leukocytes**						
Day 8	–	–	1.21x	1.18x	1.13x	1.15x
Day 29	–	–	1.48x	1.26x	1.60x	1.15x
Day 91	–	–	1.10x	1.25x	1.17x	1.60x

M = Males F = Females
A dash (—) indicates absence of a test article-related change. Numerical values indicate fold (x) change of the treated group mean value relative to the concurrent control group mean value.

All other fluctuations among individual and mean hematology values, regardless of statistical significance, were not considered test article-related due to one or more of the following: negligible magnitude, inconsistent direction of change, lack of a dose-responsive pattern, and/or relation to expected values for biologic and procedure-related variation.

3.2. Coagulation

(Table 2 and Appendix 2)

On Day 29 in males at the High Dose there was a minimal increase in mean fibrinogen concentration (1.25x), which had resolved by Day 91. This increase in fibrinogen concentration was considered test article-related and suggestive of a minor immune or inflammatory stimulus as previously discussed.

All other fluctuations among individual and mean coagulation values, regardless of statistical significance, were not considered test article-related due to one or more of the following: negligible magnitude, inconsistent direction of change, lack of a dose-responsive pattern, and/or relation to expected values for biologic and procedure-related variation.

3.3. Clinical Chemistry

(Table 3 and Appendix 3)

As illustrated in Text Table 5, on Day 29 in males at the Mid and High Dose levels and females at all dose levels there were minimal increases in mean globulin concentrations. These changes had partially to fully resolved by Day 91 at the Low and Mid Dose levels, but persisted at the High Dose. These changes were considered test article-related and suggestive of a minor immune or inflammatory stimulus, as previously discussed.

On Day 8 males at all dose levels had mild decreases in mean triglyceride concentrations, which had partially resolved by Day 29 and fully resolved by Day 91. These decreases in triglyceride concentrations were considered test article-related. No microscopic correlates were observed.

Text Table 5
scAAV9/JeT-hCLN7opt-SV40pA-Related Clinical Chemistry Changes

Group	2		3		4	
Dose Level	Low Dose		Mid Dose		High Dose	
Sex	M	F	M	F	M	F
**Globulin**						
Day 8	–	–	–	–	–	–
Day 29	–	1.10x	1.12x	1.10x	1.08x	1.09x
Day 91	–	1.05x	1.07x	–	1.09x	1.08x
**Triglycerides**						
Day 8	0.53x	–	0.57x	–	0.50x	–
Day 29	0.77x	–	0.82x	–	0.82x	–
Day 91	–	–	–	–	–	–

M = Males F = Females
A dash (—) indicates absence of a test article-related change. Numerical values indicate fold (x) change of the treated group mean value relative to the concurrent control group mean value.

All other fluctuations among individual and mean clinical chemistry values, regardless of statistical significance, were not considered test article-related due to one or more of the following: negligible magnitude, inconsistent direction of change, lack of a dose-responsive pattern, and/or relation to expected values for biologic and procedure-related variation.

Table 1
Summary of Hematology Values

Abbreviations for Hematology Parameters

MCV	-	Mean Corpuscular Volume
MCH	-	Mean Corpuscular Hemoglobin
MCHC	-	Mean Corpuscular Hemoglobin Concentration
RDW	-	Red Blood Cell Distribution Width

2759-001
A Single Dose Toxicity Study of Test Article Administered by Intrathecal Injection in Rats

Summary of Hematology Values

Sex: Male<br>Day(s) Relative to Start Date			Vehicle		Low Dose		Mid Dose		High Dose	
Leukocytes (10^3 cells/ µL)	8 [g]	Mean	13.33		14.76		16.21		15.07	
		SD	0.726		2.936		1.535		2.586	
		N	5		5		5		5	
	29 [g]	Mean	11.64		11.25		17.22	a	18.59	b
		SD	1.161		1.999		3.296		3.895	
		N	5		5		5		5	
	91 [g]	Mean	11.74		12.20		12.94		13.78	
		SD	2.112		1.685		0.947		1.660	
		N	5		5		5		5	
Erythrocytes (10^6 cells/ µL)	8 [g]	Mean	7.860		7.794		7.900		8.400	
		SD	0.4043		0.6143		0.3870		0.1500	
		N	5		5		5		5	
	29 [g]	Mean	9.246		9.114		8.866		8.448	
		SD	0.3568		0.3258		0.6760		0.4688	
		N	5		5		5		5	
	91 [g]	Mean	9.270		9.960		9.684		9.494	
		SD	0.3205		0.7675		0.3128		0.1412	
		N	5		5		5		5	
Hemoglobin (g/dL)	8 [g]	Mean	15.90		15.86		15.78		17.04	a
		SD	0.711		0.961		0.536		0.288	
		N	5		5		5		5	

[g] - Anova & Dunnett: a = p < 0.05; b = p < 0.01

2759-001
A Single Dose Toxicity Study of Test Article Administered by Intrathecal Injection in Rats

Summary of Hematology Values

Sex: Male<br>Day(s) Relative to Start Date			Vehicle	Low Dose	Mid Dose	High Dose
Hemoglobin (g/dL)	29 [g]	Mean	17.90	17.46	16.86	16.62 a
		SD	1.000	0.483	0.404	0.626
		N	5	5	5	5
	91 [g]	Mean	16.44	17.24	17.20	17.18
		SD	0.555	1.163	0.640	0.642
		N	5	5	5	5
Hematocrit (%)	8 [g]	Mean	54.64	53.36	52.18	55.28
		SD	2.797	3.310	1.659	0.915
		N	5	5	5	5
	29 [g]	Mean	56.78	55.92	53.14	51.92 a
		SD	3.600	2.128	1.787	2.524
		N	5	5	5	5
	91 [g]	Mean	53.68	54.90	55.66	54.62
		SD	2.564	4.445	2.311	2.146
		N	5	5	5	5
MCV (fL)	8 [g]	Mean	69.58	68.64	66.10	65.86
		SD	2.303	3.272	1.836	1.401
		N	5	5	5	5
	29 [g]	Mean	61.38	61.38	60.14	61.48
		SD	2.776	1.018	3.402	1.404
		N	5	5	5	5

[g] - Anova & Dunnett: a = $p < 0.05$

2759-001
A Single Dose Toxicity Study of Test Article Administered by Intrathecal Injection in Rats

Summary of Hematology Values

Sex: Male	Day(s) Relative to Start Date		Vehicle	Low Dose	Mid Dose	High Dose
MCV (fL)	91 [g]	Mean	57.92	55.12	57.50	57.52
		SD	1.983	0.638	1.961	1.659
		N	5	5	5	5
MCH (pg)	8 [g]	Mean	20.20	20.40	20.02	20.30
		SD	0.292	0.791	0.432	0.505
		N	5	5	5	5
	29 [g]	Mean	19.36	19.12	19.06	19.68
		SD	0.826	0.563	1.115	0.563
		N	5	5	5	5
	91 [g]	Mean	17.72	17.32	17.76	18.10
		SD	0.319	0.363	0.513	0.515
		N	5	5	5	5
MCHC (g/dL)	8 [g]	Mean	29.08	29.74	30.28 b	30.84 b
		SD	0.701	0.757	0.259	0.313
		N	5	5	5	5
	29 [g]	Mean	31.56	31.18	31.70	32.02
		SD	0.650	0.760	0.400	0.665
		N	5	5	5	5
	91 [g]	Mean	30.66	31.42	30.86	31.48 a
		SD	0.594	0.638	0.305	0.179
		N	5	5	5	5

[g] - Anova & Dunnett: a = p < 0.05; b = p < 0.01

2759-001
A Single Dose Toxicity Study of Test Article Administered by Intrathecal Injection in Rats

Summary of Hematology Values

Sex: Male	Day(s) Relative to Start Date		Vehicle	Low Dose	Mid Dose	High Dose
Platelets (10^3 cells/ µL)	8 [g]	Mean	1272.2	1372.4	1333.6	1260.4
		SD	137.34	129.99	77.73	137.56
		N	5	5	5	5
	29 [g]	Mean	966.6	1114.0	1143.4	1029.8
		SD	254.64	111.60	117.41	88.88
		N	5	5	5	5
	91 [g1]	Mean	960.4	1122.2	1196.2	1072.6
		SD	151.32	186.32	25.92	83.88
		N	5	5	5	5
Absolute Reticulocyte (10^3 cells/ µL)	8 [g]	Mean	430.18	441.62	442.88	432.52
		SD	29.615	29.113	72.551	66.198
		N	5	5	5	5
	29 [g]	Mean	241.28	229.14	229.86	227.40
		SD	40.906	49.361	57.482	21.198
		N	5	5	5	5
	91 [g]	Mean	173.76	186.62	193.58	224.66 b
		SD	16.971	20.152	25.652	23.925
		N	5	5	5	5
Neutrophils (10^3 cells/ µL)	8 [g]	Mean	1.682	1.540	1.794	1.324
		SD	0.3209	0.3286	0.1405	0.3589
		N	5	5	5	5

[g] - Anova & Dunnett: b = p < 0.01
[g1] - Kruskal-Wallis & Dunn

2759-001
A Single Dose Toxicity Study of Test Article Administered by Intrathecal Injection in Rats

Summary of Hematology Values

Sex: Male	Day(s) Relative to Start Date		Vehicle	Low Dose	Mid Dose	High Dose
Neutrophils (10^3 cells/ µL)	29 [g]	Mean	1.564	1.234	1.686	1.402
		SD	0.3174	0.2955	0.9141	0.5822
		N	5	5	5	5
	91 [g1]	Mean	2.304	1.314	1.622	1.926
		SD	1.4466	0.2900	0.6211	0.7255
		N	5	5	5	5
Lymphocytes (10^3 cells/ µL)	8 [g]	Mean	11.062	12.534	13.758	13.070
		SD	0.6637	2.6235	1.4903	2.1338
		N	5	5	5	5
	29 [g]	Mean	9.598	9.482	14.766 b	16.376 b
		SD	0.8871	1.8609	2.4378	3.5459
		N	5	5	5	5
	91 [g]	Mean	8.810	10.310	10.592	11.054
		SD	1.5301	1.3765	0.5542	1.1584
		N	5	5	5	5
Monocytes (10^3 cells/ µL)	8 [g]	Mean	0.360	0.424	0.392	0.400
		SD	0.0794	0.2156	0.1130	0.1329
		N	5	5	5	5
	29 [g]	Mean	0.194	0.300	0.338	0.350
		SD	0.0859	0.0866	0.1114	0.0875
		N	5	5	5	5

[g] - Anova & Dunnett: b = p < 0.01
[g1] - Anova & Dunnett(Log)

2759-001
A Single Dose Toxicity Study of Test Article Administered by Intrathecal Injection in Rats

Summary of Hematology Values

Sex: Male	Day(s) Relative to Start Date		Vehicle	Low Dose	Mid Dose	High Dose
Monocytes (10^3 cells/ µL)	91 [g]	Mean	0.326	0.258	0.402	0.394
		SD	0.0702	0.0792	0.1973	0.1633
		N	5	5	5	5
Eosinophils (10^3 cells/ µL)	8 [g]	Mean	0.060	0.064	0.068	0.066
		SD	0.0187	0.0195	0.0268	0.0270
		N	5	5	5	5
	29 [g]	Mean	0.132	0.044 a	0.106	0.076
		SD	0.0536	0.0207	0.0607	0.0321
		N	5	5	5	5
	91 [g]	Mean	0.094	0.144	0.122	0.122
		SD	0.0167	0.0577	0.0363	0.0449
		N	5	5	5	5
Basophils (10^3 cells/ µL)	8 [g]	Mean	0.066	0.068	0.072	0.074
		SD	0.0195	0.0295	0.0179	0.0241
		N	5	5	5	5
	29 [g]	Mean	0.060	0.040	0.078	0.090
		SD	0.0100	0.0122	0.0303	0.0274
		N	5	5	5	5
	91 [g]	Mean	0.060	0.046	0.052	0.066
		SD	0.0212	0.0152	0.0217	0.0089
		N	5	5	5	5

[g] - Anova & Dunnett: a = p < 0.05

2759-001
A Single Dose Toxicity Study of Test Article Administered by Intrathecal Injection in Rats

Summary of Hematology Values

Sex: Male<br>Day(s) Relative to Start Date			Vehicle	Low Dose	Mid Dose	High Dose
Other Cells (10^3 cells/ µL)	8 [g]	Mean	0.094	0.134	0.132	0.130
		SD	0.0288	0.0513	0.0356	0.0469
		N	5	5	5	5
	29 [g]	Mean	0.096	0.146	0.246 a	0.302 b
		SD	0.0297	0.0647	0.0820	0.1008
		N	5	5	5	5
	91 [g]	Mean	0.146	0.124	0.156	0.210
		SD	0.0695	0.0639	0.0607	0.0636
		N	5	5	5	5
RDW (%)	8 [g]	Mean	13.26	13.28	13.48	12.66
		SD	0.462	0.295	0.676	0.541
		N	5	5	5	5
	29 [g]	Mean	12.26	11.88	12.24	11.96
		SD	0.451	0.502	0.422	0.219
		N	5	5	5	5
	91 [g]	Mean	12.70	12.74	13.64	12.80
		SD	0.354	0.472	0.820	0.745
		N	5	5	5	5

[g] - Anova & Dunnett: a = p < 0.05; b = p < 0.01

2759-001
A Single Dose Toxicity Study of Test Article Administered by Intrathecal Injection in Rats

Summary of Hematology Values

Sex: Female<br>Day(s) Relative to Start Date			Vehicle	Low Dose	Mid Dose	High Dose
Leukocytes (10^3 cells/ µL)	8 [g]	Mean	10.38	10.34	12.24	11.89
		SD	1.937	1.746	1.969	4.357
		N	5	5	5	5
	29 [g]	Mean	10.55	11.77	13.25	12.17
		SD	4.059	1.764	3.998	1.960
		N	5	5	5	5
	91 [g1]	Mean	8.39	8.50	10.47	13.39 a
		SD	2.364	0.387	1.678	4.254
		N	5	5	5	5
Erythrocytes (10^6 cells/ µL)	8 [g]	Mean	8.080	7.836	8.202	8.488
		SD	0.2451	0.4978	0.4045	0.4711
		N	5	5	5	5
	29 [g]	Mean	9.024	8.702	8.812	8.752
		SD	0.5333	0.6657	0.5630	0.0716
		N	5	5	5	5
	91 [g]	Mean	9.100	8.482	8.694	8.768
		SD	0.6463	0.3477	0.8037	0.5758
		N	5	5	5	5
Hemoglobin (g/dL)	8 [g]	Mean	16.18	15.82	16.08	16.88
		SD	0.390	0.740	0.512	1.110
		N	5	5	5	5

[g] - Anova & Dunnett
[g1] - Anova & Dunnett(Log): a = p < 0.05

2759-001
A Single Dose Toxicity Study of Test Article Administered by Intrathecal Injection in Rats

Summary of Hematology Values

Sex: Female	Day(s) Relative to Start Date		Vehicle	Low Dose	Mid Dose	High Dose
Hemoglobin (g/dL)	29 [g]	Mean	17.44	16.66	16.52	16.54
		SD	1.001	0.956	0.712	0.279
		N	5	5	5	5
	91 [g]	Mean	16.92	16.32	16.60	16.58
		SD	0.531	0.998	1.693	1.033
		N	5	5	5	5
Hematocrit (%)	8 [g]	Mean	52.28	51.42	51.08	52.92
		SD	2.190	2.770	0.947	2.930
		N	5	5	5	5
	29 [g]	Mean	54.22	51.76	51.04	50.76
		SD	3.461	4.050	2.669	0.730
		N	5	5	5	5
	91 [g]	Mean	53.56	51.60	52.64	51.70
		SD	2.499	1.710	5.525	4.501
		N	5	5	5	5
MCV (fL)	8 [g]	Mean	64.72	65.70	62.34	62.38
		SD	2.846	1.065	2.337	2.778
		N	5	5	5	5
	29 [g]	Mean	60.10	59.48	57.94	57.98
		SD	2.372	1.874	1.837	0.602
		N	5	5	5	5

[g] - Anova & Dunnett

2759-001
A Single Dose Toxicity Study of Test Article Administered by Intrathecal Injection in Rats

Summary of Hematology Values

Sex: Female	Day(s) Relative to Start Date		Vehicle	Low Dose	Mid Dose	High Dose
MCV (fL)	91 [g]	Mean	59.00	60.90	60.54	58.90
		SD	3.109	1.313	2.786	1.465
		N	5	5	5	5
MCH (pg)	8 [g]	Mean	20.00	20.20	19.64	19.90
		SD	0.524	0.557	0.673	0.539
		N	5	5	5	5
	29 [g]	Mean	19.36	19.18	18.80	18.88
		SD	0.518	0.716	0.644	0.327
		N	5	5	5	5
	91 [g]	Mean	18.66	19.22	19.10	18.90
		SD	1.163	0.482	0.970	0.367
		N	5	5	5	5
MCHC (g/dL)	8 [g]	Mean	30.94	30.78	31.52	31.90
		SD	0.764	0.370	0.563	0.930
		N	5	5	5	5
	29 [g]	Mean	32.18	32.24	32.40	32.56
		SD	0.657	0.847	0.354	0.594
		N	5	5	5	5
	91 [g]	Mean	31.64	31.58	31.54	32.12
		SD	0.757	0.887	0.288	0.983
		N	5	5	5	5

[g] - Anova & Dunnett

2759-001
A Single Dose Toxicity Study of Test Article Administered by Intrathecal Injection in Rats

Summary of Hematology Values

Sex: Female<br>Day(s) Relative to Start Date			Vehicle	Low Dose	Mid Dose	High Dose
Platelets (10^3 cells/ µL)	8 [g]	Mean	1266.6	1126.8	1290.6	1140.6
		SD	150.35	150.25	130.56	102.16
		N	5	5	5	5
	29 [g1]	Mean	981.0	925.8	1042.2	1030.4
		SD	75.05	338.43	98.73	99.94
		N	5	5	5	5
	91 [g]	Mean	959.8	957.2	934.0	854.0
		SD	205.12	159.20	191.24	144.46
		N	5	5	5	5
Absolute Reticulocyte (10^3 cells/ µL)	8 [g]	Mean	276.58	271.76	255.60	228.68
		SD	43.596	53.896	31.181	56.954
		N	5	5	5	5
	29 [g]	Mean	215.02	217.80	223.88	204.12
		SD	29.311	33.443	39.149	36.470
		N	5	5	5	5
	91 [g]	Mean	171.08	192.04	221.50	177.86
		SD	48.296	27.511	48.805	14.964
		N	5	5	5	5
Neutrophils (10^3 cells/ µL)	8 [g]	Mean	0.842	0.700	0.964	0.806
		SD	0.2282	0.2137	0.2463	0.2420
		N	5	5	5	5

[g] - Anova & Dunnett
[g1] - Kruskal-Wallis & Dunn

2759-001
A Single Dose Toxicity Study of Test Article Administered by Intrathecal Injection in Rats

Summary of Hematology Values

Sex: Female	Day(s) Relative to Start Date		Vehicle	Low Dose	Mid Dose	High Dose
Neutrophils (10^3 cells/ µL)	29 [g]	Mean	1.134	0.886	0.896	0.788
		SD	0.5638	0.2111	0.3230	0.2704
		N	5	5	5	5
	91 [g]	Mean	0.882	0.824	0.858	0.962
		SD	0.3714	0.5714	0.3987	0.3358
		N	5	5	5	5
Lymphocytes (10^3 cells/ µL)	8 [g]	Mean	9.070	9.146	10.742	10.558
		SD	1.6969	1.4685	1.6943	3.9443
		N	5	5	5	5
	29 [g]	Mean	8.938	10.412	11.856	10.720
		SD	3.3951	1.6040	3.9092	1.6650
		N	5	5	5	5
	91 [g]	Mean	7.102	7.260	9.256	11.874 a
		SD	1.8556	0.5778	1.5897	3.9501
		N	5	5	5	5
Monocytes (10^3 cells/ µL)	8 [g]	Mean	0.226	0.296	0.286	0.302
		SD	0.0709	0.1708	0.0907	0.2141
		N	5	5	5	5
	29 [g]	Mean	0.246	0.188	0.214	0.398
		SD	0.1576	0.0427	0.0313	0.0829
		N	5	5	5	5

[g] - Anova & Dunnett: a = $p < 0.05$

2759-001
A Single Dose Toxicity Study of Test Article Administered by Intrathecal Injection in Rats

Summary of Hematology Values

Sex: Female	Day(s) Relative to Start Date		Vehicle	Low Dose	Mid Dose	High Dose
Monocytes (10^3 cells/ µL)	91 [g]	Mean	0.208	0.226	0.162	0.312
		SD	0.0887	0.1045	0.0740	0.1335
		N	5	5	5	5
Eosinophils (10^3 cells/ µL)	8 [g]	Mean	0.076	0.064	0.078	0.056
		SD	0.0270	0.0321	0.0415	0.0167
		N	5	5	5	5
	29 [g1]	Mean	0.050	0.106	0.046	0.062
		SD	0.0158	0.1101	0.0230	0.0205
		N	5	5	5	5
	91 [g]	Mean	0.072	0.050	0.058	0.062
		SD	0.0239	0.0071	0.0217	0.0259
		N	5	5	5	5
Basophils (10^3 cells/ µL)	8 [g1]	Mean	0.046	0.034	0.050	0.060
		SD	0.0167	0.0089	0.0100	0.0346
		N	5	5	5	5
	29 [g1]	Mean	0.052	0.048	0.058	0.054
		SD	0.0179	0.0179	0.0402	0.0219
		N	5	5	5	5
	91 [g]	Mean	0.030	0.030	0.042	0.058
		SD	0.0100	0.0071	0.0217	0.0286
		N	5	5	5	5

[g] - Anova & Dunnett
[g1] - Anova & Dunnett(Log)

2759-001
A Single Dose Toxicity Study of Test Article Administered by Intrathecal Injection in Rats

Summary of Hematology Values

Sex: Female<br>Day(s) Relative to Start Date			Vehicle	Low Dose	Mid Dose	High Dose
Other Cells (10^3 cells/ µL)	8 [g]	Mean	0.126	0.100	0.124	0.112
		SD	0.0577	0.0394	0.0114	0.0455
		N	5	5	5	5
	29 [g]	Mean	0.136	0.134	0.182	0.152
		SD	0.1060	0.0673	0.0746	0.0756
		N	5	5	5	5
	91 [g1]	Mean	0.096	0.106	0.094	0.122
		SD	0.0643	0.0207	0.0344	0.0669
		N	5	5	5	5
RDW (%)	8 [g]	Mean	11.74	11.60	12.00	11.48
		SD	0.416	0.235	0.406	0.205
		N	5	5	5	5
	29 [g]	Mean	11.52	11.84	12.08	12.06
		SD	0.550	0.493	0.653	0.467
		N	5	5	5	5
	91 [g]	Mean	11.72	11.50	12.08	12.08
		SD	0.370	0.374	0.295	0.576
		N	5	5	5	5

[g] - Anova & Dunnett
[g1] - Anova & Dunnett(Log)

Table 2
Summary of Coagulation Values

Abbreviation for Coagulation Parameters

APTT - Activated Partial Thromboplastin Time

2759-001
A Single Dose Toxicity Study of Test Article Administered by Intrathecal Injection in Rats

Summary of Coagulation Values

Sex: Male<br>Day(s) Relative to Start Date			Vehicle	Low Dose	Mid Dose	High Dose
APTT (sec)	8 [g]	Mean	14.83	14.78	16.32	14.76
		SD	2.722	1.132	1.076	0.152
		N	4	5	5	5
	29 [g]	Mean	17.33	16.74	18.14	15.42
		SD	2.219	1.450	1.831	1.268
		N	4	5	5	5
	91 [g]	Mean	15.94	17.30	16.10	16.70
		SD	1.698	2.521	2.423	2.563
		N	5	5	5	5
Prothrombin Time (sec)	8 [g1]	Mean	16.94	18.50 b	17.72 a	17.22
		SD	0.167	0.908	0.164	0.311
		N	5	5	5	5
	29 [g]	Mean	16.98	16.30	16.92	16.22
		SD	0.377	0.406	0.873	0.864
		N	4	5	5	5
	91 [g]	Mean	17.48	17.28	17.90	17.00
		SD	0.638	0.536	1.373	0.762
		N	5	5	5	5
Fibrinogen (mg/dL)	8 [g]	Mean	333.8	311.4	320.4	315.2
		SD	32.96	38.94	16.10	13.55
		N	4	5	5	5

[g] - Anova & Dunnett
[g1] - Kruskal-Wallis & Dunn: a = p < 0.05; b = p < 0.01

2759-001
A Single Dose Toxicity Study of Test Article Administered by Intrathecal Injection in Rats

Summary of Coagulation Values

Sex: Male	Day(s) Relative to Start Date		Vehicle	Low Dose	Mid Dose	High Dose
Fibrinogen (mg/dL)	29 [g]	Mean	277.0	312.0	323.4 a	345.8 b
		SD	20.78	16.23	26.35	25.92
		N	4	5	5	5
	91 [g]	Mean	331.4	312.6	338.2	364.0
		SD	56.97	22.38	25.96	29.09
		N	5	5	5	5

[g] - Anova & Dunnett: a = p < 0.05; b = p < 0.01

2759-001
A Single Dose Toxicity Study of Test Article Administered by Intrathecal Injection in Rats

Summary of Coagulation Values

Sex: Female	Day(s) Relative to Start Date		Vehicle	Low Dose	Mid Dose	High Dose
APTT (sec)	8 [g]	Mean	14.42	13.85	12.22	13.64
		SD	2.087	1.406	1.983	1.630
		N	5	4	5	5
	29 [g]	Mean	16.26	16.42	16.06	17.66
		SD	1.095	1.777	1.873	1.799
		N	5	5	5	5
	91 [g]	Mean	16.58	16.10	16.38	15.17
		SD	2.768	3.434	2.928	1.159
		N	4	5	5	3
Prothrombin Time (sec)	8 [g]	Mean	17.48	17.36	17.20	16.64
		SD	0.934	1.159	0.332	0.451
		N	5	5	5	5
	29 [g]	Mean	17.22	16.60	16.88	17.12
		SD	0.311	0.863	1.001	0.572
		N	5	5	5	5
	91 [g]	Mean	17.15	16.74	17.02	16.23
		SD	0.526	0.477	0.988	0.462
		N	4	5	5	3
Fibrinogen (mg/dL)	8 [g]	Mean	275.0	291.8	284.0	279.4
		SD	26.11	26.83	11.40	27.41
		N	5	5	5	5

[g] - Anova & Dunnett

2759-001
A Single Dose Toxicity Study of Test Article Administered by Intrathecal Injection in Rats

Summary of Coagulation Values

Sex: Female<br>Day(s) Relative to Start Date			Vehicle	Low Dose	Mid Dose	High Dose
Fibrinogen (mg/dL)	29 [g]	Mean	282.0	244.4	238.0	246.8
		SD	50.49	31.58	28.25	25.71
		N	5	5	5	5
	91 [g1]	Mean	240.8	218.8	220.4	228.0
		SD	21.61	5.07	7.92	23.39
		N	4	5	5	3

[g] - Anova & Dunnett
[g1] - Kruskal-Wallis & Dunn

Table 3
Summary of Clinical Chemistry Values

Abbreviations for Clinical Chemistry Parameters

AST - Aspartate Aminotransferase
ALT - Alanine Aminotransferase
ALP - Alkaline Phosphatase

2759-001
A Single Dose Toxicity Study of Test Article Administered by Intrathecal Injection in Rats

Summary of Clinical Chemistry Values

Sex: Male<br>Day(s) Relative to Start Date			Vehicle	Low Dose	Mid Dose	High Dose
Sodium (mEq/L)	8 [g]	Mean	141.2	143.4 a	141.6	143.4 a
		SD	0.45	1.82	0.89	1.14
		N	5	5	5	5
	29 [g]	Mean	142.6	142.4	141.6	141.6
		SD	1.52	1.82	1.67	0.89
		N	5	5	5	5
	91 [g]	Mean	141.6	143.6	140.8	141.6
		SD	1.14	3.05	3.11	1.95
		N	5	5	5	5
Potassium (mEq/L)	8 [g]	Mean	10.24	8.66	9.46	10.58
		SD	1.412	1.601	1.455	1.021
		N	5	5	5	5
	29 [g]	Mean	11.40	11.90	10.36	10.04
		SD	1.483	1.746	1.484	1.322
		N	5	5	5	5
	91 [g]	Mean	12.16	12.16	13.34	10.22
		SD	1.381	2.961	3.429	1.217
		N	5	5	5	5
Chloride (mEq/L)	8 [g]	Mean	100.0	101.6	100.2	100.2
		SD	2.45	3.05	1.30	1.10
		N	5	5	5	5

[g] - Anova & Dunnett: a = p < 0.05

2759-001
A Single Dose Toxicity Study of Test Article Administered by Intrathecal Injection in Rats

Summary of Clinical Chemistry Values

Sex: Male<br>Day(s) Relative to Start Date			Vehicle	Low Dose	Mid Dose	High Dose
Chloride (mEq/L)	29 [g]	Mean	101.0	100.0	99.8	99.0
		SD	2.45	1.87	1.30	1.73
		N	5	5	5	5
	91 [g]	Mean	99.6	100.2	98.4	98.6
		SD	2.07	1.48	1.52	1.14
		N	5	5	5	5
Calcium (mg/dL)	8 [g1]	Mean	13.58	13.18	13.04	12.76
		SD	1.180	0.785	0.251	0.428
		N	5	5	5	5
	29 [g1]	Mean	12.72	12.88	12.92	13.12
		SD	0.901	0.497	0.415	0.268
		N	5	5	5	5
	91 [g]	Mean	12.18	12.76	13.38 b	13.18 b
		SD	0.327	0.410	0.563	0.487
		N	5	5	5	5
Phosphorus (mg/dL)	8 [g]	Mean	14.74	13.46	14.44	15.22
		SD	0.684	1.081	1.152	1.283
		N	5	5	5	5
	29 [g]	Mean	13.18	14.18	13.56	13.00
		SD	0.983	1.501	1.264	0.316
		N	5	5	5	5

[g] - Anova & Dunnett: b = p < 0.01
[g1] - Kruskal-Wallis & Dunn

2759-001
A Single Dose Toxicity Study of Test Article Administered by Intrathecal Injection in Rats

Summary of Clinical Chemistry Values

Sex: Male Day(s) Relative to Start Date			Vehicle	Low Dose	Mid Dose	High Dose
Phosphorus (mg/dL)	91 [g]	Mean	13.44	13.54	14.22	12.12
		SD	0.826	1.387	1.951	0.981
		N	5	5	5	5
ALP (U/L)	8 [g]	Mean	387.0	317.4	246.0	306.8
		SD	84.64	123.95	39.52	48.43
		N	5	5	5	5
	29 [g]	Mean	186.4	158.8	180.6	190.8
		SD	28.01	58.95	55.63	55.57
		N	5	5	5	5
	91 [g]	Mean	108.4	105.0	80.8	97.6
		SD	12.20	29.82	8.58	25.12
		N	5	5	5	5
Total Bilirubin (mg/dL)	8 [g1]	Mean	0.12	0.10	0.10	0.10
		SD	0.045	0.000	0.000	0.000
		N	5	5	5	5
	29 [g1]	Mean	0.12	0.12	0.10	0.10
		SD	0.045	0.045	0.000	0.000
		N	5	5	5	5
	91 [g1]	Mean	0.14	0.10	0.12	0.12
		SD	0.055	0.000	0.045	0.045
		N	5	5	5	5

[g] - Anova & Dunnett
[g1] - Kruskal-Wallis & Dunn

2759-001
A Single Dose Toxicity Study of Test Article Administered by Intrathecal Injection in Rats

Summary of Clinical Chemistry Values

Sex: Male<br>Day(s) Relative to Start Date			Vehicle	Low Dose	Mid Dose	High Dose
AST (U/L)	8 [g]	Mean	89.0	78.6	74.2	82.6
		SD	7.68	16.50	3.35	9.32
		N	5	5	5	5
	29 [g1]	Mean	86.0	75.8	85.6	82.2
		SD	10.07	15.25	10.48	16.78
		N	5	5	5	5
	91 [g1]	Mean	194.4	99.2	131.6	115.8
		SD	74.42	6.76	35.71	76.40
		N	5	5	5	5
ALT (U/L)	8 [g2]	Mean	54.4	52.0	43.6	49.8
		SD	10.43	21.26	4.83	7.60
		N	5	5	5	5
	29 [g1]	Mean	40.2	33.2	38.4	35.6
		SD	10.23	5.45	8.35	8.96
		N	5	5	5	5
	91 [g]	Mean	139.8	52.6	77.4	73.0
		SD	79.23	8.05	41.06	63.19
		N	5	5	5	5
Urea Nitrogen (mg/dL)	8 [g2]	Mean	20.0	17.8	15.0 b	14.0 b
		SD	4.30	1.64	1.22	1.00
		N	5	5	5	5

[g] - Kruskal-Wallis & Dunn
[g1] - Anova & Dunnett
[g2] - Anova & Dunnett(Log): b = p < 0.01

2759-001
A Single Dose Toxicity Study of Test Article Administered by Intrathecal Injection in Rats

Summary of Clinical Chemistry Values

Sex: Male<br>Day(s) Relative to Start Date			Vehicle	Low Dose	Mid Dose	High Dose
Urea Nitrogen (mg/dL)	29 [g]	Mean	15.0	14.0	15.4	16.0
		SD	2.45	1.87	1.67	1.22
		N	5	5	5	5
	91 [g1]	Mean	19.6	15.8	17.2	16.6
		SD	7.57	0.84	1.64	1.14
		N	5	5	5	5
Creatinine (mg/dL)	8 [g1]	Mean	0.46	0.38	0.38	0.38
		SD	0.089	0.045	0.045	0.045
		N	5	5	5	5
	29 [g]	Mean	0.44	0.40	0.44	0.48
		SD	0.055	0.071	0.055	0.045
		N	5	5	5	5
	91 [g1]	Mean	0.54	0.52	0.50	0.52
		SD	0.055	0.045	0.000	0.045
		N	5	5	5	5
Total Protein (g/dL)	8 [g]	Mean	6.48	6.00	6.26	6.30
		SD	0.589	0.255	0.230	0.141
		N	5	5	5	5
	29 [g]	Mean	6.48	6.66	6.84	6.68
		SD	0.228	0.219	0.321	0.228
		N	5	5	5	5

[g] - Anova & Dunnett
[g1] - Kruskal-Wallis & Dunn

2759-001
A Single Dose Toxicity Study of Test Article Administered by Intrathecal Injection in Rats

Summary of Clinical Chemistry Values

Sex: Male<br>Day(s) Relative to Start Date			Vehicle	Low Dose	Mid Dose	High Dose
Total Protein (g/dL)	91 [g]	Mean	6.78	6.80	7.12	7.18 a
		SD	0.205	0.100	0.164	0.311
		N	5	5	5	5
Albumin (g/dL)	8 [g]	Mean	3.52	3.34	3.34	3.40
		SD	0.179	0.241	0.055	0.100
		N	5	5	5	5
	29 [g]	Mean	3.34	3.46	3.32	3.30
		SD	0.055	0.134	0.164	0.071
		N	5	5	5	5
	91 [g]	Mean	3.30	3.40	3.40	3.40
		SD	0.100	0.071	0.071	0.158
		N	5	5	5	5
Globulin (g/dL)	8 [g]	Mean	2.96	2.66	2.92	2.90
		SD	0.439	0.089	0.228	0.071
		N	5	5	5	5
	29 [g]	Mean	3.14	3.20	3.52 b	3.38
		SD	0.207	0.141	0.179	0.164
		N	5	5	5	5
	91 [g]	Mean	3.48	3.40	3.72	3.78
		SD	0.239	0.100	0.192	0.259
		N	5	5	5	5

[g] - Anova & Dunnett: a = p < 0.05; b = p < 0.01

2759-001
A Single Dose Toxicity Study of Test Article Administered by Intrathecal Injection in Rats

Summary of Clinical Chemistry Values

Sex: Male<br>Day(s) Relative to Start Date			Vehicle	Low Dose	Mid Dose	High Dose
Albumin/ Globulin	8 [g]	Mean	1.20	1.26	1.15	1.17
		SD	0.132	0.100	0.094	0.037
		N	5	5	5	5
	29 [g]	Mean	1.07	1.08	0.94 b	0.98 a
		SD	0.071	0.053	0.032	0.033
		N	5	5	5	5
	91 [g]	Mean	0.95	1.00	0.92	0.90
		SD	0.084	0.042	0.060	0.072
		N	5	5	5	5
Triglyceride (mg/dL)	8 [g1]	Mean	116.0	61.6 b	66.4 b	57.6 b
		SD	34.18	8.68	9.10	15.66
		N	5	5	5	5
	29 [g]	Mean	84.4	65.2	69.4	69.2
		SD	13.35	15.02	21.05	43.09
		N	5	5	5	5
	91 [g]	Mean	82.4	73.2	117.8	77.6
		SD	23.56	22.88	50.05	11.97
		N	5	5	5	5
Cholesterol (mg/dL)	8 [g]	Mean	67.2	69.8	81.2	74.2
		SD	3.96	12.64	17.05	11.65
		N	5	5	5	5

[g] - Anova & Dunnett: a = p < 0.05; b = p < 0.01
[g1] - Anova & Dunnett(Log): b = p < 0.01

2759-001
A Single Dose Toxicity Study of Test Article Administered by Intrathecal Injection in Rats

Summary of Clinical Chemistry Values

Sex: Male<br>Day(s) Relative to Start Date			Vehicle	Low Dose	Mid Dose	High Dose
Cholesterol (mg/dL)	29 [g]	Mean	50.0	72.0	63.4	71.6
		SD	11.98	16.17	5.94	14.91
		N	5	5	5	5
	91 [g]	Mean	74.8	78.0	74.0	82.4
		SD	21.81	16.39	6.40	18.73
		N	5	5	5	5
Glucose (mg/dL)	8 [g]	Mean	410.8	350.2	293.2	181.2
		SD	223.05	64.12	118.01	81.77
		N	5	5	5	5
	29 [g1]	Mean	362.4	339.4	351.0	364.2
		SD	233.92	85.96	119.50	102.25
		N	5	5	5	5
	91 [g]	Mean	315.2	314.2	403.2	423.2
		SD	90.05	73.49	106.35	71.84
		N	5	5	5	5

[g] - Anova & Dunnett
[g1] - Anova & Dunnett(Log)

2759-001
A Single Dose Toxicity Study of Test Article Administered by Intrathecal Injection in Rats

Summary of Clinical Chemistry Values

Sex: Female<br>Day(s) Relative to Start Date			Vehicle	Low Dose	Mid Dose	High Dose
Sodium (mEq/L)	8 [g]	Mean	141.4	139.4	140.0	139.6
		SD	1.34	1.52	1.58	1.52
		N	5	5	5	5
	29 [g]	Mean	140.0	139.0	138.8	136.8
		SD	2.24	4.06	2.59	3.03
		N	5	5	5	5
	91 [g]	Mean	140.2	139.8	141.6	139.0
		SD	2.77	0.84	1.95	2.00
		N	5	5	5	5
Potassium (mEq/L)	8 [g]	Mean	10.26	11.28	10.40	11.52
		SD	0.669	1.585	0.678	0.576
		N	5	5	5	5
	29 [g]	Mean	12.02	12.68	12.56	13.64
		SD	2.283	3.981	2.012	1.729
		N	5	5	5	5
	91 [g]	Mean	13.72	11.72	12.52	12.08
		SD	1.890	1.110	2.086	4.091
		N	5	5	5	5
Chloride (mEq/L)	8 [g]	Mean	102.0	101.0	102.2	101.4
		SD	2.12	1.58	1.10	0.89
		N	5	5	5	5

[g] - Anova & Dunnett

2759-001
A Single Dose Toxicity Study of Test Article Administered by Intrathecal Injection in Rats

Summary of Clinical Chemistry Values

Sex: Female	Day(s) Relative to Start Date		Vehicle	Low Dose	Mid Dose	High Dose
Chloride (mEq/L)	29 [g]	Mean	102.2	100.2	100.6	102.2
		SD	1.64	1.10	1.14	2.28
		N	5	5	5	5
	91 [g]	Mean	101.8	98.8	101.6	100.0
		SD	2.49	2.17	0.89	2.24
		N	5	5	5	5
Calcium (mg/dL)	8 [g]	Mean	12.88	13.18	12.44	12.90
		SD	0.476	0.370	0.462	0.886
		N	5	5	5	5
	29 [g]	Mean	12.62	12.92	13.12	12.64
		SD	0.349	0.383	0.630	0.513
		N	5	5	5	5
	91 [g]	Mean	12.78	13.08	13.14	13.32
		SD	0.497	0.576	0.586	0.593
		N	5	5	5	5
Phosphorus (mg/dL)	8 [g]	Mean	13.82	14.20	14.00	14.28
		SD	0.887	1.687	0.495	1.232
		N	5	5	5	5
	29 [g1]	Mean	13.52	14.68	13.52	12.98
		SD	1.374	3.031	1.089	0.769
		N	5	5	5	5

[g] - Anova & Dunnett
[g1] - Kruskal-Wallis & Dunn

2759-001
A Single Dose Toxicity Study of Test Article Administered by Intrathecal Injection in Rats

Summary of Clinical Chemistry Values

Sex: Female	Day(s) Relative to Start Date		Vehicle	Low Dose	Mid Dose	High Dose
Phosphorus (mg/dL)	91 [g]	Mean	13.36	13.56	12.66	12.26
		SD	1.001	2.532	2.186	2.802
		N	5	5	5	5
ALP (U/L)	8 [g]	Mean	186.4	173.0	186.2	168.8
		SD	40.32	15.35	38.82	22.38
		N	5	5	5	5
	29 [g]	Mean	149.2	101.8	123.6	91.6 a
		SD	47.04	24.54	24.42	15.47
		N	5	5	5	5
	91 [g]	Mean	52.0	44.2	48.2	38.0
		SD	13.11	10.99	15.87	7.07
		N	5	5	5	5
Total Bilirubin (mg/dL)	8 [g1]	Mean	0.10	0.10	0.10	0.12
		SD	0.000	0.000	0.000	0.045
		N	5	5	5	5
	29 [g1]	Mean	0.16	0.18	0.16	0.20
		SD	0.055	0.045	0.055	0.000
		N	5	5	5	5
	91 [g1]	Mean	0.14	0.20	0.18	0.16
		SD	0.055	0.000	0.045	0.055
		N	5	5	5	5

[g] - Anova & Dunnett: a = p < 0.05
[g1] - Kruskal-Wallis & Dunn

2759-001
A Single Dose Toxicity Study of Test Article Administered by Intrathecal Injection in Rats

Summary of Clinical Chemistry Values

Sex: Female<br>Day(s) Relative to Start Date			Vehicle	Low Dose	Mid Dose	High Dose
AST (U/L)	8 [g]	Mean	67.0	82.0	75.6	70.6
		SD	7.48	13.78	3.36	7.80
		N	5	5	5	5
	29 [g1]	Mean	85.6	122.6	76.6	75.6
		SD	21.89	89.32	10.50	16.23
		N	5	5	5	5
	91 [g2]	Mean	98.8	154.8	78.0	103.6
		SD	28.40	84.15	21.31	36.20
		N	5	5	5	5
ALT (U/L)	8 [g2]	Mean	36.8	42.4	49.0	38.6
		SD	12.44	13.90	12.71	16.83
		N	5	5	5	5
	29 [g1]	Mean	57.8	46.6	30.8	28.6 a
		SD	45.39	22.94	8.17	4.51
		N	5	5	5	5
	91 [g2]	Mean	57.8	57.6	39.6	49.6
		SD	18.46	21.24	15.52	8.44
		N	5	5	5	5
Urea Nitrogen (mg/dL)	8 [g2]	Mean	17.0	18.0	16.8	15.4
		SD	3.16	4.58	3.96	1.82
		N	5	5	5	5

[g] - Anova & Dunnett(Log)
[g1] - Kruskal-Wallis & Dunn: a = p < 0.05
[g2] - Anova & Dunnett

2759-001
A Single Dose Toxicity Study of Test Article Administered by Intrathecal Injection in Rats

Summary of Clinical Chemistry Values

Sex: Female<br>Day(s) Relative to Start Date			Vehicle	Low Dose	Mid Dose	High Dose
Urea Nitrogen (mg/dL)	29 [g]	Mean	17.8	15.4	14.8	16.8
		SD	2.59	1.14	3.42	1.48
		N	5	5	5	5
	91 [g]	Mean	15.4	16.0	15.4	16.0
		SD	2.30	4.12	1.52	1.58
		N	5	5	5	5
Creatinine (mg/dL)	8 [g]	Mean	0.38	0.44	0.32	0.40
		SD	0.084	0.055	0.045	0.071
		N	5	5	5	5
	29 [g]	Mean	0.58	0.52	0.58	0.52
		SD	0.084	0.084	0.084	0.045
		N	5	5	5	5
	91 [g]	Mean	0.58	0.58	0.54	0.56
		SD	0.045	0.084	0.055	0.055
		N	5	5	5	5
Total Protein (g/dL)	8 [g]	Mean	6.38	6.80	6.44	6.82
		SD	0.110	0.447	0.114	0.390
		N	5	5	5	5
	29 [g]	Mean	7.16	7.84 a	7.82 a	7.64
		SD	0.378	0.261	0.342	0.351
		N	5	5	5	5

[g] - Anova & Dunnett: a = $p < 0.05$

2759-001
A Single Dose Toxicity Study of Test Article Administered by Intrathecal Injection in Rats

Summary of Clinical Chemistry Values

Sex: Female	Day(s) Relative to Start Date		Vehicle	Low Dose	Mid Dose	High Dose
Total Protein (g/dL)	91 [g]	Mean	7.54	8.16	7.82	8.12
		SD	0.513	0.669	0.920	0.614
		N	5	5	5	5
Albumin (g/dL)	8 [g]	Mean	3.62	3.78	3.58	3.78
		SD	0.192	0.164	0.148	0.130
		N	5	5	5	5
	29 [g]	Mean	3.82	4.18	4.16	4.00
		SD	0.259	0.217	0.207	0.141
		N	5	5	5	5
	91 [g]	Mean	4.00	4.44	4.16	4.30
		SD	0.255	0.313	0.541	0.430
		N	5	5	5	5
Globulin (g/dL)	8 [g]	Mean	2.76	3.02	2.86	3.04
		SD	0.114	0.319	0.207	0.344
		N	5	5	5	5
	29 [g]	Mean	3.34	3.66 a	3.66 a	3.64 a
		SD	0.152	0.055	0.182	0.270
		N	5	5	5	5
	91 [g]	Mean	3.54	3.72	3.66	3.82
		SD	0.261	0.390	0.416	0.259
		N	5	5	5	5

[g] - Anova & Dunnett: a = p < 0.05

2759-001
A Single Dose Toxicity Study of Test Article Administered by Intrathecal Injection in Rats

Summary of Clinical Chemistry Values

Sex: Female<br>Day(s) Relative to Start Date			Vehicle	Low Dose	Mid Dose	High Dose
Albumin/ Globulin	8 [g]	Mean	1.32	1.26	1.26	1.26
		SD	0.122	0.110	0.141	0.151
		N	5	5	5	5
	29 [g]	Mean	1.14	1.14	1.14	1.10
		SD	0.057	0.048	0.053	0.083
		N	5	5	5	5
	91 [g]	Mean	1.13	1.20	1.14	1.13
		SD	0.020	0.073	0.075	0.091
		N	5	5	5	5
Triglyceride (mg/dL)	8 [g]	Mean	45.2	58.0	49.8	46.6
		SD	9.93	5.10	5.26	17.04
		N	5	5	5	5
	29 [g]	Mean	56.0	53.6	58.0	54.2
		SD	14.09	18.35	12.81	8.26
		N	5	5	5	5
	91 [g]	Mean	60.2	66.6	60.0	93.2
		SD	8.90	18.09	8.25	40.69
		N	5	5	5	5
Cholesterol (mg/dL)	8 [g]	Mean	67.4	77.6	71.0	76.4
		SD	8.32	10.88	18.59	22.03
		N	5	5	5	5

[g] - Anova & Dunnett

2759-001
A Single Dose Toxicity Study of Test Article Administered by Intrathecal Injection in Rats

Summary of Clinical Chemistry Values

Sex: Female<br>Day(s) Relative to Start Date			Vehicle	Low Dose	Mid Dose	High Dose
Cholesterol (mg/dL)	29 [g]	Mean	64.2	76.6	68.0	64.6
		SD	9.18	24.61	21.82	12.58
		N	5	5	5	5
	91 [g]	Mean	72.6	81.6	81.0	78.4
		SD	9.53	18.08	20.80	23.60
		N	5	5	5	5
Glucose (mg/dL)	8 [g]	Mean	302.4	341.4	237.2	211.4
		SD	86.79	145.33	111.56	165.79
		N	5	5	5	5
	29 [g]	Mean	217.2	229.8	210.2	181.4
		SD	67.64	112.84	72.19	75.95
		N	5	5	5	5
	91 [g]	Mean	190.6	244.0	226.0	245.8
		SD	80.27	99.46	61.40	86.29
		N	5	5	5	5

[g] - Anova & Dunnett

Appendix 1
Individual Hematology Values

Manual Evaluation – Blood Cell Morphology:

All Parameters:

Multi-species Ranges:	0	-	Does not meet reporting criteria

Echinocyte (Burr Cells):

Multi-species Ranges:	3+	-	≥100 cells

Acanthocyte, Spherocyte, Schistocyte, Keratocyte or Blister Cell, and Howell-Jolly Bodies:

Multi-species Ranges:	1+	-	2-5 cells
	2+	-	6-10 cells
	3+	-	≥11 cells

Polychromasia:

Multi-species Ranges:	1+	-	4-12 cells
	2+	-	13-25 cells
	3+	-	≥26 cells

Basophilic Stippling, Pappenheimer Bodies, Heinz Bodies, Eccentrocytes, and Ghost Cells:

Multi-species Ranges:	1+	-	1-3 cells
	2+	-	4-10 cells
	3+	-	≥11 cells

Unclassified Poikilocytosis:

Multi-species Ranges:	1+	-	3-10 cells
	2+	-	11-20 cells
	3+	-	≥21 cells

Codes for Individual Hematology Values

MCV	-	Mean Corpuscular Volume
MCH	-	Mean Corpuscular Hemoglobin
MCHC	-	Mean Corpuscular Hemoglobin Concentration
RDW	-	Red Blood Cell Distribution Width

Blood Cell Morphology Parameters

RBC	-	Red blood cell = Erythrocyte
Poik	-	Poikilocytes
NCC	-	Neutrophil Cytoplasmic Change
CAC	-	Consistent with automated count

2759-001
A Single Dose Toxicity Study of Test Article Administered by Intrathecal Injection in Rats

Individual Hematology Values

Sex: Male

Vehicle	Day(s) Relative to Start Date	Leukocytes (10^3 cells/ µL)	Erythrocytes (10^6 cells/ µL)	Hemoglobin (g/dL)	Hematocrit (%)	MCV (fL)	MCH (pg)
1001	91	13.3	9.50	16.7	55.0	57.9	17.5
1002	91	10.7	9.29	16.9	56.8	61.1	18.2
1003	91	10.0	9.66	16.9	54.4	56.3	17.5
1004	91	10.1	8.93	16.0	51.8	58.1	17.9
1005	91	14.7	8.97	15.7	50.4	56.2	17.5
1006	8	13.0	7.31	15.1	52.8	72.3	20.6
1007	8	12.4	8.38	17.0	58.8	70.2	20.2
1008	8	13.2	7.78	15.6	51.5	66.2	20.1
1009	8	13.9	8.10	16.1	55.5	68.6	19.8
1010	8	14.2	7.73	15.7	54.6	70.6	20.3
1011	29	10.8	8.76	16.2	50.5	57.6	18.5
1012	29	10.5	9.43	18.6	59.6	63.2	19.7
1013	29	12.9	8.98	18.5	58.1	64.7	20.6
1014	29	11.1	9.59	18.4	57.4	59.9	19.2
1015	29	12.9	9.47	17.8	58.3	61.5	18.8

2759-001
A Single Dose Toxicity Study of Test Article Administered by Intrathecal Injection in Rats

Individual Hematology Values

Sex: Male

Vehicle	Day(s) Relative to Start Date	MCHC (g/dL)	Platelets (10^3 cells/ µL)	Absolute Reticulocyte (10^3 cells/ µL)	Neutrophils (10^3 cells/ µL)	Lymphocytes (10^3 cells/ µL)	Monocytes (10^3 cells/ µL)
1001	91	30.3	880	173.7	1.73	11.04	0.31
1002	91	29.8	1067	198.1	0.94	9.02	0.41
1003	91	31.1	836	151.0	2.13	7.31	0.22
1004	91	30.9	846	177.5	1.96	7.43	0.36
1005	91	31.2	1173	168.5	4.76	9.25	0.33
1006	8	28.5	1310	475.8	1.43	10.82	0.45
1007	8	28.9	1033	396.6	1.78	10.05	0.39
1008	8	30.3	1294	423.7	1.41	11.18	0.40
1009	8	28.9	1372	416.4	1.60	11.73	0.31
1010	8	28.8	1352	438.4	2.19	11.53	0.25
1011	29	32.1	1168	170.9	1.12	9.23	0.16
1012	29	31.2	1185	246.0	1.39	8.74	0.13
1013	29	31.8	921	257.3	1.92	10.41	0.34
1014	29	32.1	557	255.4	1.61	8.93	0.20
1015	29	30.6	1002	276.8	1.78	10.68	0.14

2759-001
A Single Dose Toxicity Study of Test Article Administered by Intrathecal Injection in Rats

Individual Hematology Values

Sex: Male

Vehicle	Day(s) Relative to Start Date	Eosinophils (10^3 cells/ µL)	Basophils (10^3 cells/ µL)	Other Cells (10^3 cells/ µL)	RDW (%)
1001	91	0.09	0.06	0.05	12.7
1002	91	0.09	0.06	0.15	12.1
1003	91	0.07	0.06	0.17	13.0
1004	91	0.11	0.03	0.24	12.8
1005	91	0.11	0.09	0.12	12.9
1006	8	0.03	0.08	0.13	13.0
1007	8	0.08	0.04	0.06	12.9
1008	8	0.06	0.06	0.07	13.9
1009	8	0.07	0.09	0.11	13.6
1010	8	0.06	0.06	0.10	12.9
1011	29	0.14	0.06	0.14	11.9
1012	29	0.11	0.05	0.07	12.9
1013	29	0.08	0.07	0.11	11.8
1014	29	0.22	0.05	0.09	12.2
1015	29	0.11	0.07	0.07	12.5

2759-001
A Single Dose Toxicity Study of Test Article Administered by Intrathecal Injection in Rats

Individual Hematology Values

Sex: Male

Low Dose	Day(s) Relative to Start Date	Leukocytes (10^3 cells/ µL)	Erythrocytes (10^6 cells/ µL)	Hemoglobin (g/dL)	Hematocrit (%)	MCV (fL)	MCH (pg)
2001	91	13.3	10.95	19.0	61.2	55.9	17.3
2002	91	13.9	10.06	17.0	54.7	54.4	16.9
2003	91	10.3	9.77	16.9	54.3	55.6	17.3
2004	91	13.1	8.83	15.8	48.7	55.1	17.9
2005	91	10.5	10.19	17.5	55.6	54.6	17.2
2006	8	13.9	8.33	16.7	58.1	69.8	20.0
2007	8	13.7	6.97	15.1	51.3	73.6	21.7
2008	8	18.9	7.46	15.2	50.9	68.3	20.4
2009	8	11.1	7.76	15.2	50.9	65.6	19.6
2010	8	16.1	8.45	17.1	55.6	65.9	20.3
2011	29	8.7	9.56	18.1	59.5	62.3	18.9
2012	29	14.2	8.98	17.3	55.6	61.9	19.2
2013	29	10.6	9.21	16.9	55.2	60.0	18.3
2014	29	11.2	9.15	17.8	55.5	60.6	19.4
2015	29	11.6	8.67	17.2	53.8	62.1	19.8

2759-001
A Single Dose Toxicity Study of Test Article Administered by Intrathecal Injection in Rats

Individual Hematology Values

Sex: Male

Low Dose	Day(s) Relative to Start Date	MCHC (g/dL)	Platelets (10^3 cells/ µL)	Absolute Reticulocyte (10^3 cells/ µL)	Neutrophils (10^3 cells/ µL)	Lymphocytes (10^3 cells/ µL)	Monocytes (10^3 cells/ µL)
2001	91	31.0	958	194.3	1.42	11.20	0.33
2002	91	31.1	1365	215.6	1.59	11.73	0.23
2003	91	31.0	1048	186.9	0.84	9.11	0.14
2004	91	32.5	1274	172.8	1.46	10.92	0.33
2005	91	31.5	966	163.5	1.26	8.59	0.26
2006	8	28.7	1404	406.0	1.20	12.00	0.53
2007	8	29.5	1313	448.7	1.31	11.97	0.22
2008	8	29.8	1576	435.8	1.68	16.26	0.75
2009	8	29.9	1340	431.9	1.48	9.03	0.33
2010	8	30.8	1229	485.7	2.03	13.41	0.29
2011	29	30.3	1152	294.0	1.49	6.88	0.18
2012	29	31.1	1202	229.3	1.57	12.00	0.35
2013	29	30.6	1054	250.7	1.10	8.94	0.27
2014	29	32.0	949	160.1	1.16	9.44	0.41
2015	29	31.9	1213	211.6	0.85	10.15	0.29

2759-001
A Single Dose Toxicity Study of Test Article Administered by Intrathecal Injection in Rats

Individual Hematology Values

Sex: Male

Low Dose	Day(s) Relative to Start Date	Eosinophils (10^3 cells/ µL)	Basophils (10^3 cells/ µL)	Other Cells (10^3 cells/ µL)	RDW (%)
2001	91	0.12	0.06	0.11	12.2
2002	91	0.18	0.06	0.07	13.5
2003	91	0.06	0.03	0.08	12.7
2004	91	0.15	0.05	0.23	12.6
2005	91	0.21	0.03	0.13	12.7
2006	8	0.05	0.06	0.09	13.7
2007	8	0.07	0.06	0.13	13.2
2008	8	0.04	0.05	0.13	13.4
2009	8	0.09	0.05	0.10	13.2
2010	8	0.07	0.12	0.22	12.9
2011	29	0.05	0.04	0.06	12.0
2012	29	0.06	0.06	0.17	11.7
2013	29	0.01	0.03	0.18	12.7
2014	29	0.04	0.04	0.10	11.5
2015	29	0.06	0.03	0.22	11.5

2759-001
A Single Dose Toxicity Study of Test Article Administered by Intrathecal Injection in Rats

Individual Hematology Values

Sex: Male

Mid Dose	Day(s) Relative to Start Date	Leukocytes (10^3 cells/ µL)	Erythrocytes (10^6 cells/ µL)	Hemoglobin (g/dL)	Hematocrit (%)	MCV (fL)	MCH (pg)
3001	91	13.1	9.90	18.1	58.8	59.4	18.3
3002	91	12.5	9.57	16.4	52.5	54.9	17.2
3003	91	12.3	9.66	17.1	55.9	57.9	17.6
3004	91	12.4	9.24	16.9	54.7	59.2	18.3
3005	91	14.5	10.05	17.5	56.4	56.1	17.4
3006	8	18.0	7.65	15.4	51.1	66.8	20.2
3007	8	17.1	7.46	15.4	51.5	69.0	20.7
3008	8	14.1	8.48	16.7	55.1	65.0	19.7
3009	8	16.6	7.97	15.7	51.9	65.2	19.7
3010	8	15.2	7.94	15.7	51.3	64.5	19.8
3011	29	16.8	9.60	17.3	54.3	56.6	18.0
3012	29	15.8	9.32	17.1	54.5	58.5	18.4
3013	29	17.2	8.61	17.0	54.4	63.1	19.7
3014	29	13.7	7.86	16.3	50.6	64.4	20.7
3015	29	22.6	8.94	16.6	51.9	58.1	18.5

2759-001
A Single Dose Toxicity Study of Test Article Administered by Intrathecal Injection in Rats

Individual Hematology Values

Sex: Male

Mid Dose	Day(s) Relative to Start Date	MCHC (g/dL)	Platelets (10^3 cells/ µL)	Absolute Reticulocyte (10^3 cells/ µL)	Neutrophils (10^3 cells/ µL)	Lymphocytes (10^3 cells/ µL)	Monocytes (10^3 cells/ µL)
3001	91	30.7	1210	203.5	1.36	11.30	0.26
3002	91	31.3	1211	160.3	1.54	9.97	0.60
3003	91	30.5	1183	173.3	1.44	10.09	0.34
3004	91	30.8	1157	209.8	1.08	10.77	0.19
3005	91	31.0	1220	221.0	2.69	10.83	0.62
3006	8	30.2	1429	394.8	1.98	15.26	0.50
3007	8	30.0	1385	424.9	1.61	14.86	0.35
3008	8	30.3	1241	557.0	1.71	11.61	0.52
3009	8	30.2	1341	372.8	1.83	14.12	0.33
3010	8	30.7	1272	464.9	1.84	12.94	0.26
3011	29	31.8	1225	192.6	1.36	14.89	0.20
3012	29	31.4	1201	302.5	1.85	13.17	0.48
3013	29	31.2	1243	281.1	1.05	15.62	0.29
3014	29	32.2	966	177.2	0.97	11.89	0.30
3015	29	31.9	1082	195.9	3.20	18.26	0.42

2759-001
A Single Dose Toxicity Study of Test Article Administered by Intrathecal Injection in Rats

Individual Hematology Values

Sex: Male

Mid Dose	Day(s) Relative to Start Date	Eosinophils (10^3 cells/ µL)	Basophils (10^3 cells/ µL)	Other Cells (10^3 cells/ µL)	RDW (%)
3001	91	0.08	0.04	0.08	12.4
3002	91	0.12	0.05	0.18	14.4
3003	91	0.11	0.04	0.24	14.3
3004	91	0.12	0.04	0.16	13.8
3005	91	0.18	0.09	0.12	13.3
3006	8	0.08	0.09	0.10	12.6
3007	8	0.04	0.06	0.16	13.1
3008	8	0.08	0.05	0.17	14.2
3009	8	0.10	0.09	0.14	14.1
3010	8	0.04	0.07	0.09	13.4
3011	29	0.07	0.05	0.23	11.5
3012	29	0.07	0.07	0.18	12.3
3013	29	0.05	0.07	0.17	12.5
3014	29	0.15	0.07	0.28	12.5
3015	29	0.19	0.13	0.37	12.4

2759-001
A Single Dose Toxicity Study of Test Article Administered by Intrathecal Injection in Rats

Individual Hematology Values

Sex: Male

High Dose	Day(s) Relative to Start Date	Leukocytes (10^3 cells/ µL)	Erythrocytes (10^6 cells/ µL)	Hemoglobin (g/dL)	Hematocrit (%)	MCV (fL)	MCH (pg)
4001	91	15.8	9.56	16.9	53.8	56.3	17.7
4002	91	11.8	9.39	17.1	53.8	57.3	18.2
4003	91	15.2	9.66	17.7	56.5	58.5	18.3
4004	91	13.3	9.55	17.9	57.1	59.8	18.8
4005	91	12.8	9.31	16.3	51.9	55.7	17.5
4006	8	18.3	8.49	16.7	54.4	64.1	19.7
4007	8	16.0	8.28	16.8	54.2	65.5	20.3
4008	8	11.7	8.25	17.2	55.9	67.8	20.8
4009	8	13.3	8.61	17.1	56.2	65.3	19.9
4010	8	16.1	8.37	17.4	55.7	66.6	20.8
4011	29	12.7	9.06	17.3	55.1	60.8	19.1
4012	29	17.0	8.23	16.6	52.5	63.8	20.1
4013	29	19.8	8.33	16.0	50.3	60.4	19.2
4014	29	22.9	7.86	16.0	48.6	61.8	20.4
4015	29	20.5	8.76	17.2	53.1	60.6	19.6

2759-001
A Single Dose Toxicity Study of Test Article Administered by Intrathecal Injection in Rats

Individual Hematology Values

Sex: Male

High Dose	Day(s) Relative to Start Date	MCHC (g/dL)	Platelets (10^3 cells/ µL)	Absolute Reticulocyte (10^3 cells/ µL)	Neutrophils (10^3 cells/ µL)	Lymphocytes (10^3 cells/ µL)	Monocytes (10^3 cells/ µL)
4001	91	31.4	1031	202.4	3.20	11.47	0.52
4002	91	31.8	1049	234.0	1.66	9.70	0.14
4003	91	31.4	978	210.9	1.71	12.71	0.32
4004	91	31.4	1198	262.1	1.37	11.12	0.48
4005	91	31.4	1107	213.9	1.69	10.27	0.51
4006	8	30.7	1316	338.6	1.64	15.69	0.55
4007	8	31.0	1410	424.1	1.28	14.20	0.36
4008	8	30.7	1041	416.8	0.84	10.35	0.30
4009	8	30.5	1236	518.2	1.15	11.52	0.26
4010	8	31.3	1299	464.9	1.71	13.59	0.53
4011	29	31.4	928	228.7	0.87	11.07	0.39
4012	29	31.6	988	263.1	0.90	15.33	0.21
4013	29	31.7	1121	218.0	2.30	16.66	0.42
4014	29	33.0	1126	208.5	1.42	20.54	0.41
4015	29	32.4	986	218.7	1.52	18.28	0.32

2759-001
A Single Dose Toxicity Study of Test Article Administered by Intrathecal Injection in Rats

Individual Hematology Values

Sex: Male

High Dose	Day(s) Relative to Start Date	Eosinophils (10^3 cells/ µL)	Basophils (10^3 cells/ µL)	Other Cells (10^3 cells/ µL)	RDW (%)
4001	91	0.18	0.08	0.30	13.8
4002	91	0.10	0.06	0.16	12.8
4003	91	0.16	0.07	0.25	11.7
4004	91	0.09	0.06	0.15	12.8
4005	91	0.08	0.06	0.19	12.9
4006	8	0.10	0.09	0.18	12.2
4007	8	0.05	0.06	0.08	12.4
4008	8	0.08	0.04	0.08	12.3
4009	8	0.07	0.10	0.16	13.5
4010	8	0.03	0.08	0.15	12.9
4011	29	0.04	0.06	0.31	12.1
4012	29	0.05	0.09	0.46	12.1
4013	29	0.09	0.10	0.20	12.1
4014	29	0.12	0.13	0.31	11.9
4015	29	0.08	0.07	0.23	11.6

2759-001
A Single Dose Toxicity Study of Test Article Administered by Intrathecal Injection in Rats

Individual Blood Cell Morphology

Sex: Male

Low Dose	Day(s) Relative to Start Date	Blood Cell Morphology	Nucleated RBC	Polychrom-asia	Heinz Bodies	Basophilic Stippling	Pappenheimer Bodies
2001	91	Findings	0	0	0	0	0
2002	91	Findings	0	0	0	0	0
2005	91	No Finding	0	0	0	0	0

2759-001
A Single Dose Toxicity Study of Test Article Administered by Intrathecal Injection in Rats

Individual Blood Cell Morphology

Sex: Male

Low Dose	Day(s) Relative to Start Date	Howell-Jolly Bodies	Echinocyte (Burr Cells)	Spherocyte	Schistocyte	Acanthocyte	Keratocyte, Blister Cell
2001	91	0	0	0	0	3+	0
2002	91	0	0	0	0	3+	0
2005	91	0	0	0	0	0	0

2759-001
A Single Dose Toxicity Study of Test Article Administered by Intrathecal Injection in Rats

Individual Blood Cell Morphology

Sex: Male

Low Dose	Day(s) Relative to Start Date	Eccentrocyte	Unclassified Poik	Ghost Cells	Rouleaux	RBC Agg-lutination	NCC
2001	91	0	0	0	0	0	0
2002	91	0	0	0	0	0	0
2005	91	0	0	0	0	0	0

2759-001
A Single Dose Toxicity Study of Test Article Administered by Intrathecal Injection in Rats

Individual Blood Cell Morphology

Sex: Male

Low Dose	Day(s) Relative to Start Date	Smudge Cells	Pyknotic Cells	Reactive Lymphocyte	Reactive Monocytes	Leukocyte A-gglutination	Platelet Es-timate Count
2001	91	0	0	0	0	0	CAC
2002	91	0	0	0	0	0	CAC
2005	91	0	0	0	0	0	CAC

2759-001
A Single Dose Toxicity Study of Test Article Administered by Intrathecal Injection in Rats

Individual Blood Cell Morphology

Sex: Male

Low Dose	Day(s) Relative to Start Date	Platelet Clumps	Large Platelets
2001	91	Present	0
2002	91	0	0
2005	91	0	0

2759-001
A Single Dose Toxicity Study of Test Article Administered by Intrathecal Injection in Rats

Individual Blood Cell Morphology

Sex: Male

Mid Dose	Day(s) Relative to Start Date	Blood Cell Morphology	Nucleated RBC	Polychrom-asia	Heinz Bodies	Basophilic Stippling	Pappenheimer Bodies
3005	91	Findings	0	1+	0	0	0

2759-001
A Single Dose Toxicity Study of Test Article Administered by Intrathecal Injection in Rats

Individual Blood Cell Morphology

Sex: Male

Mid Dose	Day(s) Relative to Start Date	Howell-Jolly Bodies	Echinocyte (Burr Cells)	Spherocyte	Schistocyte	Acanthocyte	Keratocyte, Blister Cell
3005	91	0	0	0	0	3+	0

2759-001
A Single Dose Toxicity Study of Test Article Administered by Intrathecal Injection in Rats

Individual Blood Cell Morphology

Sex: Male

Mid Dose	Day(s) Relative to Start Date	Eccentrocyte	Unclassified Poik	Ghost Cells	Rouleaux	RBC Agg-lutination	NCC
3005	91	0	0	0	0	0	0

2759-001
A Single Dose Toxicity Study of Test Article Administered by Intrathecal Injection in Rats

Individual Blood Cell Morphology

Sex: Male

Mid Dose	Day(s) Relative to Start Date	Smudge Cells	Pyknotic Cells	Reactive Lymphocyte	Reactive Monocytes	Leukocyte A-gglutination	Platelet Es-timate Count
3005	91	0	0	0	0	0	CAC

2759-001
A Single Dose Toxicity Study of Test Article Administered by Intrathecal Injection in Rats

Individual Blood Cell Morphology

Sex: Male

Mid Dose	Day(s) Relative to Start Date	Platelet Clumps	Large Platelets
3005	91	Present	0

2759-001
A Single Dose Toxicity Study of Test Article Administered by Intrathecal Injection in Rats

Individual Hematology Values

Sex: Female

Vehicle	Day(s) Relative to Start Date	Leukocytes (10^3 cells/ µL)	Erythrocytes (10^6 cells/ µL)	Hemoglobin (g/dL)	Hematocrit (%)	MCV (fL)	MCH (pg)
1501	91	6.0	8.95	17.4	56.2	62.8	19.5
1502	91	6.2	9.95	16.9	55.2	55.5	17.0
1503	91	9.8	9.45	17.5	54.6	57.8	18.5
1504	91	11.6	8.93	16.3	51.1	57.2	18.3
1505	91	8.4	8.22	16.5	50.7	61.7	20.0
1506	8	12.3	7.81	16.0	53.0	67.9	20.4
1507	8	9.9	8.31	16.6	54.3	65.3	20.0
1508	8	8.4	8.20	16.6	54.2	66.1	20.2
1509	8	8.8	7.82	15.9	50.1	64.0	20.3
1510	8	12.5	8.26	15.8	49.8	60.3	19.1
1511	29	8.1	9.48	18.2	57.3	60.4	19.2
1512	29	9.4	8.84	17.6	56.3	63.7	20.0
1513	29	5.8	8.18	15.9	48.9	59.8	19.4
1514	29	15.4	9.41	18.4	56.0	59.5	19.6
1515	29	14.1	9.21	17.1	52.6	57.1	18.6

2759-001
A Single Dose Toxicity Study of Test Article Administered by Intrathecal Injection in Rats

Individual Hematology Values

Sex: Female

Vehicle	Day(s) Relative to Start Date	MCHC (g/dL)	Platelets (10^3 cells/ µL)	Absolute Reticulocyte (10^3 cells/ µL)	Neutrophils (10^3 cells/ µL)	Lymphocytes (10^3 cells/ µL)	Monocytes (10^3 cells/ µL)
1501	91	31.0	968	249.9	0.56	5.22	0.12
1502	91	30.7	1143	141.2	0.57	5.30	0.15
1503	91	32.0	694	160.1	0.95	8.44	0.20
1504	91	32.0	822	125.8	1.47	9.38	0.35
1505	91	32.5	1172	178.4	0.86	7.17	0.22
1506	8	30.1	1234	309.1	1.16	10.65	0.23
1507	8	30.6	1043	255.6	0.85	8.70	0.12
1508	8	30.5	1258	324.9	0.65	7.39	0.20
1509	8	31.7	1353	278.1	0.60	7.58	0.29
1510	8	31.8	1445	215.2	0.95	11.03	0.29
1511	29	31.7	1011	184.5	0.87	6.95	0.14
1512	29	31.3	857	211.8	0.53	8.51	0.19
1513	29	32.5	1058	249.5	0.99	4.58	0.10
1514	29	32.9	999	189.1	2.03	12.46	0.49
1515	29	32.5	980	240.2	1.25	12.19	0.31

2759-001
A Single Dose Toxicity Study of Test Article Administered by Intrathecal Injection in Rats

Individual Hematology Values

Sex: Female

Vehicle	Day(s) Relative to Start Date	Eosinophils (10^3 cells/ µL)	Basophils (10^3 cells/ µL)	Other Cells (10^3 cells/ µL)	RDW (%)
1501	91	0.06	0.02	0.06	11.3
1502	91	0.04	0.04	0.06	11.5
1503	91	0.07	0.03	0.07	12.0
1504	91	0.10	0.04	0.21	11.6
1505	91	0.09	0.02	0.08	12.2
1506	8	0.11	0.04	0.11	12.3
1507	8	0.04	0.06	0.09	11.9
1508	8	0.09	0.02	0.06	11.2
1509	8	0.08	0.05	0.20	11.5
1510	8	0.06	0.06	0.17	11.8
1511	29	0.06	0.04	0.06	10.9
1512	29	0.03	0.07	0.08	12.3
1513	29	0.04	0.03	0.04	11.6
1514	29	0.07	0.07	0.27	11.7
1515	29	0.05	0.05	0.23	11.1

2759-001
A Single Dose Toxicity Study of Test Article Administered by Intrathecal Injection in Rats

Individual Hematology Values

Sex: Female

Low Dose	Day(s) Relative to Start Date	Leukocytes (10^3 cells/ µL)	Erythrocytes (10^6 cells/ µL)	Hemoglobin (g/dL)	Hematocrit (%)	MCV (fL)	MCH (pg)
2501	91	8.5	8.35	15.5	50.1	60.1	18.6
2502	91	8.6	8.65	17.0	53.2	61.5	19.6
2503	91	8.7	9.01	17.7	53.5	59.4	19.6
2504	91	7.8	8.19	16.0	51.4	62.8	19.5
2505	91	8.8	8.21	15.4	49.8	60.7	18.8
2506	8	11.1	7.13	14.9	47.8	67.0	20.9
2507	8	10.9	7.67	15.2	49.6	64.7	19.8
2508	8	7.3	8.46	16.5	54.5	64.5	19.5
2509	8	10.5	7.81	16.0	51.6	66.1	20.5
2510	8	11.8	8.11	16.5	53.6	66.2	20.3
2511	29	12.8	9.74	17.7	56.8	58.3	18.2
2512	29	12.6	8.16	16.3	50.2	61.5	20.0
2513	29	12.1	8.86	17.0	53.9	60.8	19.2
2514	29	12.7	8.09	15.2	46.0	56.9	18.8
2515	29	8.7	8.66	17.1	51.9	59.9	19.7

2759-001
A Single Dose Toxicity Study of Test Article Administered by Intrathecal Injection in Rats

Individual Hematology Values

Sex: Female

Low Dose	Day(s) Relative to Start Date	MCHC (g/dL)	Platelets (10^3 cells/ µL)	Absolute Reticulocyte (10^3 cells/ µL)	Neutrophils (10^3 cells/ µL)	Lymphocytes (10^3 cells/ µL)	Monocytes (10^3 cells/ µL)
2501	91	30.9	1023	209.6	0.71	7.34	0.29
2502	91	31.9	725	198.1	0.39	7.65	0.34
2503	91	33.0	891	167.9	0.47	7.96	0.11
2504	91	31.1	997	224.7	0.74	6.64	0.27
2505	91	31.0	1150	159.9	1.81	6.71	0.12
2506	8	31.2	1250	338.6	0.56	9.74	0.56
2507	8	30.6	1103	221.6	0.58	9.91	0.25
2508	8	30.3	888	231.8	0.55	6.57	0.11
2509	8	31.1	1134	246.2	0.76	9.36	0.21
2510	8	30.7	1259	320.6	1.05	10.15	0.35
2511	29	31.2	386	175.7	1.16	10.95	0.23
2512	29	32.5	950	235.7	0.74	11.45	0.18
2513	29	31.5	1325	212.0	0.79	10.80	0.17
2514	29	33.1	980	202.0	1.06	11.28	0.13
2515	29	32.9	988	263.6	0.68	7.58	0.23

2759-001
A Single Dose Toxicity Study of Test Article Administered by Intrathecal Injection in Rats

Individual Hematology Values

Sex: Female

Low Dose	Day(s) Relative to Start Date	Eosinophils (10^3 cells/ µL)	Basophils (10^3 cells/ µL)	Other Cells (10^3 cells/ µL)	RDW (%)
2501	91	0.05	0.02	0.11	11.2
2502	91	0.04	0.03	0.14	11.0
2503	91	0.05	0.04	0.09	11.7
2504	91	0.05	0.03	0.10	11.8
2505	91	0.06	0.03	0.09	11.8
2506	8	0.06	0.03	0.14	11.4
2507	8	0.05	0.04	0.10	11.8
2508	8	0.04	0.02	0.04	11.5
2509	8	0.05	0.04	0.09	11.9
2510	8	0.12	0.04	0.13	11.4
2511	29	0.30	0.05	0.08	12.7
2512	29	0.05	0.06	0.11	11.8
2513	29	0.09	0.03	0.25	11.6
2514	29	0.05	0.07	0.13	11.6
2515	29	0.04	0.03	0.10	11.5

2759-001
A Single Dose Toxicity Study of Test Article Administered by Intrathecal Injection in Rats

Individual Hematology Values

Sex: Female

Mid Dose	Day(s) Relative to Start Date	Leukocytes (10^3 cells/ µL)	Erythrocytes (10^6 cells/ µL)	Hemoglobin (g/dL)	Hematocrit (%)	MCV (fL)	MCH (pg)
3501	91	12.3	9.68	18.9	60.3	62.3	19.5
3502	91	10.0	8.60	16.2	51.6	60.0	18.9
3503	91	12.2	8.32	17.1	53.5	64.3	20.5
3504	91	9.5	7.62	14.2	44.8	58.8	18.7
3505	91	8.5	9.25	16.6	53.0	57.3	17.9
3506	8	12.1	7.66	15.3	49.8	65.0	20.0
3507	8	14.2	7.89	16.1	50.7	64.2	20.4
3508	8	9.1	8.58	16.0	50.9	59.3	18.6
3509	8	12.4	8.38	16.3	52.2	62.3	19.5
3510	8	13.5	8.50	16.7	51.8	60.9	19.7
3511	29	20.2	8.51	16.7	51.8	60.8	19.7
3512	29	10.4	9.37	17.2	53.0	56.6	18.4
3513	29	11.7	7.98	15.4	46.6	58.3	19.2
3514	29	11.0	9.04	16.3	50.7	56.1	18.1
3515	29	13.1	9.16	17.0	53.1	57.9	18.6

2759-001
A Single Dose Toxicity Study of Test Article Administered by Intrathecal Injection in Rats

Individual Hematology Values

Sex: Female

Mid Dose	Day(s) Relative to Start Date	MCHC (g/dL)	Platelets (10^3 cells/ µL)	Absolute Reticulocyte (10^3 cells/ µL)	Neutrophils (10^3 cells/ µL)	Lymphocytes (10^3 cells/ µL)	Monocytes (10^3 cells/ µL)
3501	91	31.3	755	165.3	1.15	10.66	0.22
3502	91	31.4	977	205.2	1.41	8.14	0.26
3503	91	31.9	900	281.4	0.58	11.25	0.11
3504	91	31.8	799	192.9	0.64	8.51	0.13
3505	91	31.3	1239	262.7	0.51	7.72	0.09
3506	8	30.8	1403	249.9	1.00	10.48	0.41
3507	8	31.8	1409	290.6	1.33	12.25	0.33
3508	8	31.4	1106	206.4	0.65	7.99	0.18
3509	8	31.3	1211	263.0	0.87	11.03	0.29
3510	8	32.3	1324	268.1	0.97	11.96	0.22
3511	29	32.3	944	268.5	0.78	18.72	0.24
3512	29	32.4	1002	233.5	0.49	9.40	0.18
3513	29	33.0	1116	160.6	0.82	10.31	0.24
3514	29	32.2	974	229.4	1.03	9.56	0.23
3515	29	32.1	1175	227.4	1.36	11.29	0.18

2759-001
A Single Dose Toxicity Study of Test Article Administered by Intrathecal Injection in Rats

Individual Hematology Values

Sex: Female

Mid Dose	Day(s) Relative to Start Date	Eosinophils (10^3 cells/ µL)	Basophils (10^3 cells/ µL)	Other Cells (10^3 cells/ µL)	RDW (%)
3501	91	0.08	0.07	0.08	12.4
3502	91	0.05	0.03	0.07	11.8
3503	91	0.03	0.06	0.12	11.9
3504	91	0.08	0.03	0.14	11.9
3505	91	0.05	0.02	0.06	12.4
3506	8	0.04	0.04	0.12	12.2
3507	8	0.10	0.05	0.11	12.3
3508	8	0.05	0.06	0.13	11.3
3509	8	0.06	0.04	0.12	12.0
3510	8	0.14	0.06	0.14	12.2
3511	29	0.03	0.13	0.28	13.0
3512	29	0.08	0.04	0.19	12.3
3513	29	0.02	0.04	0.22	11.5
3514	29	0.05	0.04	0.09	12.2
3515	29	0.05	0.04	0.13	11.4

2759-001
A Single Dose Toxicity Study of Test Article Administered by Intrathecal Injection in Rats

Individual Hematology Values

Sex: Female

High Dose	Day(s) Relative to Start Date	Leukocytes (10^3 cells/ µL)	Erythrocytes (10^6 cells/ µL)	Hemoglobin (g/dL)	Hematocrit (%)	MCV (fL)	MCH (pg)
4501	91	8.6	9.10	17.1	55.0	60.5	18.7
4502	91	17.2	9.54	17.7	57.1	59.8	18.6
4503	91	10.0	8.03	15.0	45.9	57.2	18.7
4504	91	18.3	8.52	16.2	49.0	57.5	19.0
4505	91	12.9	8.65	16.9	51.5	59.5	19.5
4506	8	19.0	8.42	17.5	55.7	66.1	20.8
4507	8	9.0	8.74	17.0	51.7	59.1	19.5
4508	8	7.9	7.92	15.5	50.8	64.2	19.6
4509	8	12.6	8.22	16.1	50.0	60.8	19.6
4510	8	11.1	9.14	18.3	56.4	61.7	20.0
4511	29	11.6	8.77	16.6	51.7	59.0	18.9
4512	29	11.8	8.74	17.0	50.7	58.0	19.4
4513	29	10.2	8.75	16.4	50.4	57.5	18.8
4514	29	15.5	8.85	16.4	51.2	57.8	18.5
4515	29	11.8	8.65	16.3	49.8	57.6	18.8

2759-001
A Single Dose Toxicity Study of Test Article Administered by Intrathecal Injection in Rats

Individual Hematology Values

Sex: Female

High Dose	Day(s) Relative to Start Date	MCHC (g/dL)	Platelets (10^3 cells/ µL)	Absolute Reticulocyte (10^3 cells/ µL)	Neutrophils (10^3 cells/ µL)	Lymphocytes (10^3 cells/ µL)	Monocytes (10^3 cells/ µL)
4501	91	31.0	706	169.4	1.14	7.07	0.27
4502	91	31.1	797	198.1	1.37	15.06	0.49
4503	91	32.7	968	166.2	0.72	8.94	0.15
4504	91	33.0	756	189.6	1.05	16.41	0.40
4505	91	32.8	1043	166.0	0.53	11.89	0.25
4506	8	31.5	1166	305.5	1.04	16.88	0.67
4507	8	32.9	983	178.4	0.44	8.03	0.30
4508	8	30.5	1266	260.2	0.87	6.66	0.18
4509	8	32.2	1160	230.2	0.98	11.19	0.22
4510	8	32.4	1128	169.1	0.70	10.03	0.14
4511	29	32.1	1040	145.1	0.72	10.16	0.45
4512	29	33.5	926	209.1	0.83	10.38	0.33
4513	29	32.6	986	198.5	0.37	9.32	0.30
4514	29	32.0	1193	233.3	0.93	13.61	0.50
4515	29	32.6	1007	234.6	1.09	10.13	0.41

2759-001
A Single Dose Toxicity Study of Test Article Administered by Intrathecal Injection in Rats

Individual Hematology Values

Sex: Female

High Dose	Day(s) Relative to Start Date	Eosinophils (10^3 cells/ µL)	Basophils (10^3 cells/ µL)	Other Cells (10^3 cells/ µL)	RDW (%)
4501	91	0.07	0.02	0.05	12.5
4502	91	0.03	0.08	0.12	12.7
4503	91	0.08	0.04	0.09	11.7
4504	91	0.09	0.09	0.23	12.2
4505	91	0.04	0.06	0.12	11.3
4506	8	0.06	0.12	0.19	11.8
4507	8	0.06	0.06	0.10	11.3
4508	8	0.04	0.04	0.08	11.5
4509	8	0.08	0.04	0.08	11.5
4510	8	0.04	0.04	0.11	11.3
4511	29	0.08	0.04	0.12	11.9
4512	29	0.04	0.06	0.20	11.4
4513	29	0.04	0.04	0.10	12.4
4514	29	0.07	0.09	0.26	12.0
4515	29	0.08	0.04	0.08	12.6

2759-001
A Single Dose Toxicity Study of Test Article Administered by Intrathecal Injection in Rats

Individual Blood Cell Morphology

Sex: Female

Mid Dose	Day(s) Relative to Start Date	Blood Cell Morphology	Nucleated RBC	Polychrom-asia	Heinz Bodies	Basophilic Stippling	Pappenheimer Bodies
3501	91	Findings	0	1+	0	0	0

2759-001
A Single Dose Toxicity Study of Test Article Administered by Intrathecal Injection in Rats

Individual Blood Cell Morphology

Sex: Female

Mid Dose	Day(s) Relative to Start Date	Howell-Jolly Bodies	Echinocyte (Burr Cells)	Spherocyte	Schistocyte	Acanthocyte	Keratocyte, Blister Cell
3501	91	0	0	0	0	0	0

2759-001
A Single Dose Toxicity Study of Test Article Administered by Intrathecal Injection in Rats

Individual Blood Cell Morphology

Sex: Female

Mid Dose	Day(s) Relative to Start Date	Eccentrocyte	Unclassified Poik	Ghost Cells	Rouleaux	RBC Agg-lutination	NCC
3501	91	0	0	0	0	0	0

2759-001
A Single Dose Toxicity Study of Test Article Administered by Intrathecal Injection in Rats

Individual Blood Cell Morphology

Sex: Female

Mid Dose	Day(s) Relative to Start Date	Smudge Cells	Pyknotic Cells	Reactive Lymphocyte	Reactive Monocytes	Leukocyte A-gglutination	Platelet Es-timate Count
3501	91	0	0	0	0	0	CAC

2759-001
A Single Dose Toxicity Study of Test Article Administered by Intrathecal Injection in Rats

Individual Blood Cell Morphology

Sex: Female

Mid Dose	Day(s) Relative to Start Date	Platelet Clumps	Large Platelets
3501	91	Present	0

Appendix 2
Individual Coagulation Values

Codes for Individual Coagulation Values

APTT	-	Activated Partial Thromboplastin Time
CT	-	Clotted Sample
QNS	-	Quantity Not Sufficient

2759-001
A Single Dose Toxicity Study of Test Article Administered by Intrathecal Injection in Rats

Individual Coagulation Values

Sex: Male

Vehicle	Day(s) Relative to Start Date	APTT (sec)	Prothrombin Time (sec)	Fibrinogen (mg/dL)
1001	91	17.5	17.6	270
1002	91	16.5	17.9	304
1003	91	13.8	16.6	314
1004	91	17.4	17.1	349
1005	91	14.5	18.2	420
1006	8	12.5	17.2	379
1007	8	18.7	17.0	301
1008	8	14.6	16.8	322
1009	8	13.5	16.9	333
1010	8	QNS	16.8	QNS
1011	29	14.9	16.7	299
1012	29	17.9	17.0	251
1013	29	CT	CT	CT
1014	29	20.1	17.5	271
1015	29	16.4	16.7	287

2759-001
A Single Dose Toxicity Study of Test Article Administered by Intrathecal Injection in Rats

Individual Coagulation Values

Sex: Male

Low Dose	Day(s) Relative to Start Date	APTT (sec)	Prothrombin Time (sec)	Fibrinogen (mg/dL)
2001	91	20.9	16.8	310
2002	91	14.5	17.1	345
2003	91	17.4	17.8	284
2004	91	15.4	16.8	320
2005	91	18.3	17.9	304
2006	8	16.4	17.3	367
2007	8	14.2	17.9	335
2008	8	13.5	18.7	299
2009	8	14.4	19.0	274
2010	8	15.4	19.6	282
2011	29	17.3	15.9	310
2012	29	16.7	16.2	303
2013	29	14.6	16.0	338
2014	29	18.6	16.9	295
2015	29	16.5	16.5	314

2759-001
A Single Dose Toxicity Study of Test Article Administered by Intrathecal Injection in Rats

Individual Coagulation Values

Sex: Male

Mid Dose	Day(s) Relative to Start Date	APTT (sec)	Prothrombin Time (sec)	Fibrinogen (mg/dL)
3001	91	19.0	19.8	299
3002	91	18.0	16.9	333
3003	91	14.2	18.8	370
3004	91	13.3	17.5	340
3005	91	16.0	16.5	349
3006	8	15.0	17.6	342
3007	8	16.0	17.9	316
3008	8	17.7	17.8	316
3009	8	17.1	17.8	329
3010	8	15.8	17.5	299
3011	29	20.9	18.3	297
3012	29	18.4	16.6	352
3013	29	17.7	16.9	320
3014	29	15.8	16.9	349
3015	29	17.9	15.9	299

2759-001
A Single Dose Toxicity Study of Test Article Administered by Intrathecal Injection in Rats

Individual Coagulation Values

Sex: Male

High Dose	Day(s) Relative to Start Date	APTT (sec)	Prothrombin Time (sec)	Fibrinogen (mg/dL)
4001	91	18.2	16.5	400
4002	91	14.6	17.5	362
4003	91	15.9	18.1	320
4004	91	20.4	16.4	362
4005	91	14.4	16.5	376
4006	8	14.8	17.5	318
4007	8	14.9	17.4	304
4008	8	14.5	16.7	301
4009	8	14.8	17.2	335
4010	8	14.8	17.3	318
4011	29	16.9	16.5	301
4012	29	15.1	17.4	362
4013	29	14.3	16.1	365
4014	29	14.2	16.1	349
4015	29	16.6	15.0	352

2759-001
A Single Dose Toxicity Study of Test Article Administered by Intrathecal Injection in Rats

Individual Coagulation Values

Sex: Female

Vehicle	Day(s) Relative to Start Date	APTT (sec)	Prothrombin Time (sec)	Fibrinogen (mg/dL)
1501	91	CT	CT	CT
1502	91	13.3	17.6	231
1503	91	19.5	16.6	261
1504	91	18.1	17.6	215
1505	91	15.4	16.8	256
1506	8	18.1	17.3	282
1507	8	13.1	17.1	279
1508	8	13.9	16.3	301
1509	8	13.8	18.8	282
1510	8	13.2	17.9	231
1511	29	18.2	17.7	261
1512	29	15.7	17.0	306
1513	29	15.6	17.2	247
1514	29	15.8	17.3	237
1515	29	16.0	16.9	359

2759-001
A Single Dose Toxicity Study of Test Article Administered by Intrathecal Injection in Rats

Individual Coagulation Values

Sex: Female

Low Dose	Day(s) Relative to Start Date	APTT (sec)	Prothrombin Time (sec)	Fibrinogen (mg/dL)
2501	91	21.8	17.1	215
2502	91	15.7	17.2	226
2503	91	15.5	16.6	214
2504	91	15.0	16.8	217
2505	91	12.5	16.0	222
2506	8	12.8	17.7	282
2507	8	12.5	18.6	276
2508	8	15.3	18.0	299
2509	8	14.8	16.9	335
2510	8	QNS	15.6	267
2511	29	17.7	15.2	210
2512	29	13.4	17.1	284
2513	29	17.1	17.4	271
2514	29	16.3	16.9	225
2515	29	17.6	16.4	232

2759-001
A Single Dose Toxicity Study of Test Article Administered by Intrathecal Injection in Rats

Individual Coagulation Values

Sex: Female

Mid Dose	Day(s) Relative to Start Date	APTT (sec)	Prothrombin Time (sec)	Fibrinogen (mg/dL)
3501	91	20.1	18.7	208
3502	91	16.9	16.6	219
3503	91	16.3	17.1	225
3504	91	11.9	16.3	229
3505	91	16.7	16.4	221
3506	8	13.2	17.5	281
3507	8	13.2	17.5	273
3508	8	14.0	17.1	274
3509	8	11.7	16.7	297
3510	8	9.0	17.2	295
3511	29	14.1	18.5	196
3512	29	15.9	16.5	251
3513	29	16.1	17.1	227
3514	29	15.1	15.9	245
3515	29	19.1	16.4	271

2759-001
A Single Dose Toxicity Study of Test Article Administered by Intrathecal Injection in Rats

Individual Coagulation Values

Sex: Female

High Dose	Day(s) Relative to Start Date	APTT (sec)	Prothrombin Time (sec)	Fibrinogen (mg/dL)
4501	91	CT	CT	CT
4502	91	16.4	16.5	241
4503	91	14.1	15.7	242
4504	91	CT	CT	CT
4505	91	15.0	16.5	201
4506	8	13.1	17.2	308
4507	8	11.4	16.9	234
4508	8	14.1	16.6	285
4509	8	13.7	16.5	289
4510	8	15.9	16.0	281
4511	29	17.8	17.6	273
4512	29	16.6	16.6	233
4513	29	20.6	17.5	231
4514	29	15.9	16.4	276
4515	29	17.4	17.5	221

Appendix 3
Individual Clinical Chemistry Values

Codes for Individual Clinical Chemistry Values

AST - Aspartate Aminotransferase
ALT - Alanine Aminotransferase
ALP - Alkaline Phosphatase

Hemolytic Index Result Reference Guide

0 - No Hemolysis
1 - Slight Hemolysis
2 - Hemolyzed

2759-001
A Single Dose Toxicity Study of Test Article Administered by Intrathecal Injection in Rats

Individual Clinical Chemistry Values

Sex: Male

Vehicle	Day(s) Relative to Start Date	Sodium (mEq/L)	Potassium (mEq/L)	Chloride (mEq/L)	Calcium (mg/dL)	Phosphorus (mg/dL)	ALP (U/L)	Total Bilirubin (mg/dL)	AST (U/L)	ALT (U/L)	Urea Nitrogen (mg/dL)
1001	91	142	11.4	102	12.2	12.5	108	0.2	90	71	16
1002	91	141	13.6	100	12.7	12.9	105	0.1	169	89	15
1003	91	142	10.2	98	12.1	13.2	109	0.2	180	88	18
1004	91	143	12.4	101	11.8	14.4	127	0.1	268	240	16
1005	91	140	13.2	97	12.1	14.2	93	0.1	265	211	33
1006	8	141	9.3	98	14.4	14.7	490	0.1	85	70	20
1007	8	141	12.6	103	12.8	15.4	265	0.1	84	43	16
1008	8	141	9.8	101	12.0	14.4	394	0.2	102	59	17
1009	8	141	10.4	101	13.8	13.8	432	0.1	90	49	20
1010	8	142	9.1	97	14.9	15.4	354	0.1	84	51	27
1011	29	144	9.0	99	13.6	11.5	195	0.1	79	31	14
1012	29	141	12.4	102	12.0	14.0	151	0.2	95	43	18
1013	29	144	11.2	104	12.2	13.2	194	0.1	90	48	17
1014	29	143	12.8	102	12.0	13.5	224	0.1	94	51	14
1015	29	141	11.6	98	13.8	13.7	168	0.1	72	28	12

2759-001
A Single Dose Toxicity Study of Test Article Administered by Intrathecal Injection in Rats

Individual Clinical Chemistry Values

Sex: Male

Vehicle	Day(s) Relative to Start Date	Creatinine (mg/dL)	Total Protein (g/dL)	Albumin (g/dL)	Globulin (g/dL)	Albumin/ Globulin	Triglyceride (mg/dL)	Cholesterol (mg/dL)	Glucose (mg/dL)	Hemolytic Index
1001	91	0.5	6.6	3.4	3.2	1.1	66	43	367	0
1002	91	0.6	7.0	3.4	3.6	0.9	123	66	443	0
1003	91	0.5	6.7	3.2	3.5	0.9	66	100	292	1
1004	91	0.5	6.6	3.3	3.3	1.0	80	88	255	1
1005	91	0.6	7.0	3.2	3.8	0.8	77	77	219	1
1006	8	0.4	6.4	3.4	3.0	1.1	134	64	629	0
1007	8	0.4	5.8	3.3	2.5	1.3	157	62	309	0
1008	8	0.4	6.1	3.5	2.6	1.3	74	71	82	0
1009	8	0.5	6.8	3.7	3.1	1.2	88	70	445	0
1010	8	0.6	7.3	3.7	3.6	1.0	127	69	589	0
1011	29	0.5	6.7	3.4	3.3	1.0	96	50	584	0
1012	29	0.4	6.1	3.3	2.8	1.2	68	45	209	0
1013	29	0.5	6.5	3.3	3.2	1.0	72	64	209	0
1014	29	0.4	6.5	3.4	3.1	1.1	95	58	161	0
1015	29	0.4	6.6	3.3	3.3	1.0	91	33	649	0

2759-001
A Single Dose Toxicity Study of Test Article Administered by Intrathecal Injection in Rats

Individual Clinical Chemistry Values

Sex: Male

Low Dose	Day(s) Relative to Start Date	Sodium (mEq/L)	Potassium (mEq/L)	Chloride (mEq/L)	Calcium (mg/dL)	Phosphorus (mg/dL)	ALP (U/L)	Total Bilirubin (mg/dL)	AST (U/L)	ALT (U/L)	Urea Nitrogen (mg/dL)
2001	91	142	15.0	102	12.4	13.9	91	0.1	106	58	15
2002	91	140	15.4	98	13.2	15.7	79	0.1	94	60	16
2003	91	143	11.8	100	12.4	13.4	156	0.1	93	57	17
2004	91	148	9.2	100	12.6	12.2	96	0.1	107	46	16
2005	91	145	9.4	101	13.2	12.5	103	0.1	96	42	15
2006	8	142	8.1	104	13.5	13.2	264	0.1	65	44	18
2007	8	145	6.1	98	13.7	11.8	301	0.1	69	42	19
2008	8	141	9.5	99	14.0	14.6	238	0.1	67	26	19
2009	8	145	10.0	105	12.5	14.2	535	0.1	102	74	18
2010	8	144	9.6	102	12.2	13.5	249	0.1	90	74	15
2011	29	144	14.6	103	12.9	16.4	136	0.1	61	31	12
2012	29	141	11.8	98	13.4	14.0	190	0.1	83	41	15
2013	29	144	11.0	99	13.3	14.4	168	0.1	68	34	12
2014	29	140	12.2	100	12.6	13.9	228	0.1	99	34	16
2015	29	143	9.9	100	12.2	12.2	72	0.2	68	26	15

2759-001
A Single Dose Toxicity Study of Test Article Administered by Intrathecal Injection in Rats

Individual Clinical Chemistry Values

Sex: Male

Low Dose	Day(s) Relative to Start Date	Creatinine (mg/dL)	Total Protein (g/dL)	Albumin (g/dL)	Globulin (g/dL)	Albumin/ Globulin	Triglyceride (mg/dL)	Cholesterol (mg/dL)	Glucose (mg/dL)	Hemolytic Index
2001	91	0.5	6.8	3.5	3.3	1.1	72	86	195	1
2002	91	0.5	6.7	3.4	3.3	1.0	112	72	390	0
2003	91	0.5	6.7	3.3	3.4	1.0	54	83	331	0
2004	91	0.5	6.9	3.4	3.5	1.0	69	96	305	1
2005	91	0.6	6.9	3.4	3.5	1.0	59	53	350	0
2006	8	0.4	6.1	3.5	2.6	1.3	70	68	382	0
2007	8	0.4	6.3	3.6	2.7	1.3	69	91	388	0
2008	8	0.4	6.0	3.4	2.6	1.3	64	67	383	0
2009	8	0.4	5.6	3.0	2.6	1.2	54	57	361	0
2010	8	0.3	6.0	3.2	2.8	1.1	51	66	237	0
2011	29	0.4	6.3	3.3	3.0	1.1	78	47	387	0
2012	29	0.5	6.8	3.6	3.2	1.1	77	76	409	0
2013	29	0.4	6.8	3.4	3.4	1.0	72	84	408	0
2014	29	0.3	6.8	3.6	3.2	1.1	44	66	231	2
2015	29	0.4	6.6	3.4	3.2	1.1	55	87	262	1

2759-001
A Single Dose Toxicity Study of Test Article Administered by Intrathecal Injection in Rats

Individual Clinical Chemistry Values

Sex: Male

Mid Dose	Day(s) Relative to Start Date	Sodium (mEq/L)	Potassium (mEq/L)	Chloride (mEq/L)	Calcium (mg/dL)	Phosphorus (mg/dL)	ALP (U/L)	Total Bilirubin (mg/dL)	AST (U/L)	ALT (U/L)	Urea Nitrogen (mg/dL)
3001	91	137	18.4	100	13.1	16.6	67	0.2	166	104	19
3002	91	142	11.8	100	12.8	13.3	81	0.1	104	38	18
3003	91	143	11.4	97	14.2	13.8	80	0.1	109	55	18
3004	91	138	15.2	97	13.1	15.7	88	0.1	175	136	15
3005	91	144	9.9	98	13.7	11.7	88	0.1	104	54	16
3006	8	143	7.8	99	13.4	13.3	218	0.1	71	38	16
3007	8	142	9.0	102	13.2	15.3	239	0.1	71	40	15
3008	8	141	11.8	101	12.8	16.0	303	0.1	79	48	13
3009	8	141	9.4	100	12.9	13.9	266	0.1	75	49	16
3010	8	141	9.3	99	12.9	13.7	204	0.1	75	43	15
3011	29	142	9.9	99	12.7	12.1	123	0.1	72	29	14
3012	29	141	12.8	102	12.4	15.3	149	0.1	91	45	15
3013	29	139	10.6	99	12.9	14.1	158	0.1	79	31	18
3014	29	143	9.5	99	13.5	13.7	262	0.1	99	48	16
3015	29	143	9.0	100	13.1	12.6	211	0.1	87	39	14

2759-001
A Single Dose Toxicity Study of Test Article Administered by Intrathecal Injection in Rats

Individual Clinical Chemistry Values

Sex: Male

Mid Dose	Day(s) Relative to Start Date	Creatinine (mg/dL)	Total Protein (g/dL)	Albumin (g/dL)	Globulin (g/dL)	Albumin/ Globulin	Triglyceride (mg/dL)	Cholesterol (mg/dL)	Glucose (mg/dL)	Hemolytic Index
3001	91	0.5	7.1	3.4	3.7	0.9	81	69	265	1
3002	91	0.5	7.0	3.5	3.5	1.0	69	74	394	0
3003	91	0.5	7.4	3.4	4.0	0.9	110	77	528	0
3004	91	0.5	7.0	3.4	3.6	0.9	134	83	343	0
3005	91	0.5	7.1	3.3	3.8	0.9	195	67	486	0
3006	8	0.4	6.2	3.4	2.8	1.2	62	70	448	0
3007	8	0.3	5.9	3.3	2.6	1.3	55	68	325	0
3008	8	0.4	6.5	3.3	3.2	1.0	65	109	122	0
3009	8	0.4	6.3	3.3	3.0	1.1	79	73	260	0
3010	8	0.4	6.4	3.4	3.0	1.1	71	86	311	0
3011	29	0.4	7.3	3.6	3.7	1.0	82	71	368	0
3012	29	0.4	7.0	3.3	3.7	0.9	61	57	162	1
3013	29	0.4	6.6	3.2	3.4	0.9	100	58	483	0
3014	29	0.5	6.5	3.2	3.3	1.0	51	67	409	0
3015	29	0.5	6.8	3.3	3.5	0.9	53	64	333	0

2759-001
A Single Dose Toxicity Study of Test Article Administered by Intrathecal Injection in Rats

Individual Clinical Chemistry Values

Sex: Male

High Dose	Day(s) Relative to Start Date	Sodium (mEq/L)	Potassium (mEq/L)	Chloride (mEq/L)	Calcium (mg/dL)	Phosphorus (mg/dL)	ALP (U/L)	Total Bilirubin (mg/dL)	AST (U/L)	ALT (U/L)	Urea Nitrogen (mg/dL)
4001	91	143	8.6	99	12.8	11.1	78	0.1	66	36	18
4002	91	141	10.6	99	12.7	12.1	120	0.2	251	178	17
4003	91	139	11.8	98	13.9	12.6	128	0.1	93	87	17
4004	91	144	10.6	100	13.1	13.5	90	0.1	92	29	16
4005	91	141	9.5	97	13.4	11.3	72	0.1	77	35	15
4006	8	145	9.3	99	12.3	14.1	361	0.1	86	45	15
4007	8	143	12.0	100	12.4	15.1	274	0.1	87	41	13
4008	8	144	11.0	100	13.2	17.4	278	0.1	88	50	15
4009	8	143	10.6	102	13.2	14.5	263	0.1	66	52	14
4010	8	142	10.0	100	12.7	15.0	358	0.1	86	61	13
4011	29	141	10.8	100	13.1	13.5	127	0.1	71	30	15
4012	29	141	11.0	96	13.3	12.8	168	0.1	66	23	16
4013	29	143	8.6	99	13.1	12.7	242	0.1	77	38	18
4014	29	142	8.6	100	13.4	12.9	256	0.1	89	43	16
4015	29	141	11.2	100	12.7	13.1	161	0.1	108	44	15

2759-001
A Single Dose Toxicity Study of Test Article Administered by Intrathecal Injection in Rats

Individual Clinical Chemistry Values

Sex: Male

High Dose	Day(s) Relative to Start Date	Creatinine (mg/dL)	Total Protein (g/dL)	Albumin (g/dL)	Globulin (g/dL)	Albumin/ Globulin	Triglyceride (mg/dL)	Cholesterol (mg/dL)	Glucose (mg/dL)	Hemolytic Index
4001	91	0.5	7.4	3.3	4.1	0.8	90	109	373	0
4002	91	0.5	6.9	3.2	3.7	0.9	59	93	367	1
4003	91	0.6	6.9	3.4	3.5	1.0	86	66	545	0
4004	91	0.5	7.1	3.5	3.6	1.0	76	65	418	0
4005	91	0.5	7.6	3.6	4.0	0.9	77	79	413	0
4006	8	0.4	6.5	3.5	3.0	1.2	71	73	126	0
4007	8	0.4	6.4	3.5	2.9	1.2	73	57	110	0
4008	8	0.4	6.2	3.4	2.8	1.2	36	84	140	0
4009	8	0.4	6.2	3.3	2.9	1.1	60	86	303	0
4010	8	0.3	6.2	3.3	2.9	1.1	48	71	227	0
4011	29	0.4	6.3	3.2	3.1	1.0	66	58	343	0
4012	29	0.5	6.7	3.3	3.4	1.0	143	97	522	0
4013	29	0.5	6.7	3.3	3.4	1.0	39	65	333	0
4014	29	0.5	6.8	3.3	3.5	0.9	60	70	382	0
4015	29	0.5	6.9	3.4	3.5	1.0	38	68	241	0

2759-001
A Single Dose Toxicity Study of Test Article Administered by Intrathecal Injection in Rats

Individual Clinical Chemistry Values

Sex: Female

Vehicle	Day(s) Relative to Start Date	Sodium (mEq/L)	Potassium (mEq/L)	Chloride (mEq/L)	Calcium (mg/dL)	Phosphorus (mg/dL)	ALP (U/L)	Total Bilirubin (mg/dL)	AST (U/L)	ALT (U/L)	Urea Nitrogen (mg/dL)
1501	91	144	13.2	106	12.4	13.0	29	0.1	88	50	16
1502	91	141	16.0	102	13.1	13.4	61	0.2	73	53	19
1503	91	137	15.4	100	12.5	15.0	57	0.1	141	81	13
1504	91	138	12.2	101	12.4	13.1	59	0.2	114	71	15
1505	91	141	11.8	100	13.5	12.3	54	0.1	78	34	14
1506	8	140	10.6	103	13.0	13.8	192	0.1	64	30	15
1507	8	143	10.8	105	12.6	13.9	141	0.1	74	35	16
1508	8	142	10.4	100	13.3	15.1	150	0.1	60	26	14
1509	8	140	10.4	100	13.3	13.7	234	0.1	76	58	18
1510	8	142	9.1	102	12.2	12.6	215	0.1	61	35	22
1511	29	137	15.6	100	13.0	15.0	230	0.2	72	37	14
1512	29	140	11.8	104	12.3	13.2	150	0.1	75	38	17
1513	29	141	9.5	103	12.2	11.6	114	0.1	69	37	21
1514	29	143	12.4	103	12.8	14.7	128	0.2	122	139	19
1515	29	139	10.8	101	12.8	13.1	124	0.2	90	38	18

2759-001
A Single Dose Toxicity Study of Test Article Administered by Intrathecal Injection in Rats

Individual Clinical Chemistry Values

Sex: Female

Vehicle	Day(s) Relative to Start Date	Creatinine (mg/dL)	Total Protein (g/dL)	Albumin (g/dL)	Globulin (g/dL)	Albumin/ Globulin	Triglyceride (mg/dL)	Cholesterol (mg/dL)	Glucose (mg/dL)	Hemolytic Index
1501	91	0.6	7.2	3.8	3.4	1.1	67	70	176	0
1502	91	0.6	8.1	4.3	3.8	1.1	62	80	116	0
1503	91	0.6	8.0	4.2	3.8	1.1	68	84	134	0
1504	91	0.5	6.9	3.7	3.2	1.2	58	69	208	0
1505	91	0.6	7.5	4.0	3.5	1.1	46	60	319	0
1506	8	0.3	6.4	3.7	2.7	1.4	61	65	236	0
1507	8	0.4	6.4	3.6	2.8	1.3	34	59	311	0
1508	8	0.3	6.5	3.9	2.6	1.5	46	71	237	0
1509	8	0.5	6.4	3.5	2.9	1.2	44	62	447	0
1510	8	0.4	6.2	3.4	2.8	1.2	41	80	281	0
1511	29	0.7	7.3	3.9	3.4	1.1	71	70	287	0
1512	29	0.6	6.6	3.5	3.1	1.1	70	77	241	0
1513	29	0.5	7.0	3.6	3.4	1.1	44	60	256	0
1514	29	0.6	7.3	4.0	3.3	1.2	54	60	115	0
1515	29	0.5	7.6	4.1	3.5	1.2	41	54	187	0

2759-001
A Single Dose Toxicity Study of Test Article Administered by Intrathecal Injection in Rats

Individual Clinical Chemistry Values

Sex: Female

Low Dose	Day(s) Relative to Start Date	Sodium (mEq/L)	Potassium (mEq/L)	Chloride (mEq/L)	Calcium (mg/dL)	Phosphorus (mg/dL)	ALP (U/L)	Total Bilirubin (mg/dL)	AST (U/L)	ALT (U/L)	Urea Nitrogen (mg/dL)
2501	91	141	12.0	100	13.8	16.4	55	0.2	88	38	12
2502	91	140	12.0	100	13.2	15.7	38	0.2	90	38	15
2503	91	139	13.2	97	12.4	13.6	33	0.2	168	79	23
2504	91	140	11.2	101	12.6	11.5	57	0.2	293	81	15
2505	91	139	10.2	96	13.4	10.6	38	0.2	135	52	15
2506	8	141	10.4	102	13.2	13.0	153	0.1	64	33	19
2507	8	138	11.8	100	12.9	12.9	182	0.1	95	56	25
2508	8	139	12.2	103	13.1	16.7	177	0.1	87	58	18
2509	8	141	9.0	99	13.8	13.2	191	0.1	71	38	15
2510	8	138	13.0	101	12.9	15.2	162	0.1	93	27	13
2511	29	134	18.2	99	12.5	17.6	84	0.2	280	87	17
2512	29	143	8.8	100	12.9	11.3	122	0.2	67	43	14
2513	29	136	15.2	102	13.4	17.4	72	0.2	108	35	15
2514	29	143	9.4	100	12.6	11.7	130	0.1	75	32	15
2515	29	139	11.8	100	13.2	15.4	101	0.2	83	36	16

2759-001
A Single Dose Toxicity Study of Test Article Administered by Intrathecal Injection in Rats

Individual Clinical Chemistry Values

Sex: Female

Low Dose	Day(s) Relative to Start Date	Creatinine (mg/dL)	Total Protein (g/dL)	Albumin (g/dL)	Globulin (g/dL)	Albumin/ Globulin	Triglyceride (mg/dL)	Cholesterol (mg/dL)	Glucose (mg/dL)	Hemolytic Index
2501	91	0.5	8.2	4.6	3.6	1.3	96	80	318	0
2502	91	0.5	7.7	4.3	3.4	1.3	67	78	210	0
2503	91	0.6	7.9	4.3	3.6	1.2	66	74	96	0
2504	91	0.6	7.7	4.1	3.6	1.1	49	64	247	0
2505	91	0.7	9.3	4.9	4.4	1.1	55	112	349	0
2506	8	0.4	6.3	3.7	2.6	1.4	61	72	445	0
2507	8	0.4	6.5	3.7	2.8	1.3	63	61	470	0
2508	8	0.5	7.1	3.9	3.2	1.2	53	84	135	0
2509	8	0.5	7.4	4.0	3.4	1.2	61	85	413	0
2510	8	0.4	6.7	3.6	3.1	1.2	52	86	244	0
2511	29	0.6	8.2	4.5	3.7	1.2	59	54	84	0
2512	29	0.5	7.5	3.9	3.6	1.1	77	110	339	0
2513	29	0.4	7.9	4.2	3.7	1.1	62	80	310	2
2514	29	0.5	7.9	4.2	3.7	1.1	37	88	280	0
2515	29	0.6	7.7	4.1	3.6	1.1	33	51	136	0

2759-001
A Single Dose Toxicity Study of Test Article Administered by Intrathecal Injection in Rats

Individual Clinical Chemistry Values

Sex: Female

Mid Dose	Day(s) Relative to Start Date	Sodium (mEq/L)	Potassium (mEq/L)	Chloride (mEq/L)	Calcium (mg/dL)	Phosphorus (mg/dL)	ALP (U/L)	Total Bilirubin (mg/dL)	AST (U/L)	ALT (U/L)	Urea Nitrogen (mg/dL)
3501	91	143	12.4	103	13.3	16.5	30	0.2	113	63	18
3502	91	141	11.0	101	14.0	11.4	33	0.2	80	48	15
3503	91	139	14.4	102	12.5	12.2	60	0.1	57	31	15
3504	91	144	10.0	101	13.2	12.0	65	0.2	73	29	14
3505	91	141	14.8	101	12.7	11.2	53	0.2	67	27	15
3506	8	138	9.6	101	13.1	14.3	249	0.1	77	38	23
3507	8	140	11.2	103	12.5	14.0	198	0.1	76	34	16
3508	8	141	10.8	103	11.8	13.3	167	0.1	76	51	13
3509	8	142	9.8	101	12.4	13.8	163	0.1	70	59	18
3510	8	139	10.6	103	12.4	14.6	154	0.1	79	63	14
3511	29	136	15.4	101	12.3	12.9	130	0.2	95	40	19
3512	29	142	10.6	101	13.7	15.2	92	0.2	74	32	14
3513	29	137	12.0	100	13.0	12.5	150	0.2	74	37	14
3514	29	141	11.0	102	13.8	13.0	105	0.1	71	24	17
3515	29	138	13.8	99	12.8	14.0	141	0.1	69	21	10

2759-001
A Single Dose Toxicity Study of Test Article Administered by Intrathecal Injection in Rats

Individual Clinical Chemistry Values

Sex: Female

Mid Dose	Day(s) Relative to Start Date	Creatinine (mg/dL)	Total Protein (g/dL)	Albumin (g/dL)	Globulin (g/dL)	Albumin/ Globulin	Triglyceride (mg/dL)	Cholesterol (mg/dL)	Glucose (mg/dL)	Hemolytic Index
3501	91	0.5	7.5	4.1	3.4	1.2	66	73	137	0
3502	91	0.6	9.1	5.0	4.1	1.2	66	107	291	0
3503	91	0.5	6.6	3.5	3.1	1.1	66	75	236	0
3504	91	0.5	7.7	4.0	3.7	1.1	50	96	270	0
3505	91	0.6	8.2	4.2	4.0	1.1	52	54	196	0
3506	8	0.4	6.3	3.6	2.7	1.3	43	68	394	0
3507	8	0.3	6.4	3.8	2.6	1.5	47	49	157	0
3508	8	0.3	6.5	3.6	2.9	1.2	52	59	175	0
3509	8	0.3	6.6	3.5	3.1	1.1	57	95	316	0
3510	8	0.3	6.4	3.4	3.0	1.1	50	84	144	0
3511	29	0.5	7.4	4.0	3.4	1.2	68	53	113	0
3512	29	0.6	8.0	4.3	3.7	1.2	60	102	250	0
3513	29	0.5	8.1	4.2	3.9	1.1	42	77	164	0
3514	29	0.7	8.1	4.4	3.7	1.2	48	59	296	0
3515	29	0.6	7.5	3.9	3.6	1.1	72	49	228	0

2759-001
A Single Dose Toxicity Study of Test Article Administered by Intrathecal Injection in Rats

Individual Clinical Chemistry Values

Sex: Female

High Dose	Day(s) Relative to Start Date	Sodium (mEq/L)	Potassium (mEq/L)	Chloride (mEq/L)	Calcium (mg/dL)	Phosphorus (mg/dL)	ALP (U/L)	Total Bilirubin (mg/dL)	AST (U/L)	ALT (U/L)	Urea Nitrogen (mg/dL)
4501	91	140	11.8	99	13.7	15.0	47	0.1	71	45	18
4502	91	136	18.8	104	13.6	15.5	35	0.2	93	60	17
4503	91	141	8.5	99	13.9	10.1	41	0.2	77	39	16
4504	91	140	9.1	99	12.9	9.5	39	0.1	117	48	15
4505	91	138	12.2	99	12.5	11.2	28	0.2	160	56	14
4506	8	140	11.8	102	13.5	15.1	194	0.1	80	29	16
4507	8	141	10.8	101	11.7	12.4	156	0.1	59	21	18
4508	8	137	12.0	100	14.0	13.7	164	0.1	74	46	15
4509	8	140	11.0	102	12.6	14.8	141	0.1	68	64	15
4510	8	140	12.0	102	12.7	15.4	189	0.2	72	33	13
4511	29	138	12.2	101	13.5	13.2	115	0.2	70	29	17
4512	29	132	16.0	99	12.2	12.7	88	0.2	103	35	17
4513	29	136	13.6	103	12.4	13.4	94	0.2	68	23	19
4514	29	140	14.6	105	12.7	13.8	89	0.2	76	30	15
4515	29	138	11.8	103	12.4	11.8	72	0.2	61	26	16

2759-001
A Single Dose Toxicity Study of Test Article Administered by Intrathecal Injection in Rats

Individual Clinical Chemistry Values

Sex: Female

High Dose	Day(s) Relative to Start Date	Creatinine (mg/dL)	Total Protein (g/dL)	Albumin (g/dL)	Globulin (g/dL)	Albumin/ Globulin	Triglyceride (mg/dL)	Cholesterol (mg/dL)	Glucose (mg/dL)	Hemolytic Index
4501	91	0.5	8.6	4.7	3.9	1.2	158	49	227	0
4502	91	0.6	7.8	4.2	3.6	1.2	97	77	174	0
4503	91	0.6	8.9	4.8	4.1	1.2	70	115	308	0
4504	91	0.5	7.4	3.9	3.5	1.1	91	77	360	0
4505	91	0.6	7.9	3.9	4.0	1.0	50	74	160	0
4506	8	0.4	6.8	3.9	2.9	1.3	50	76	248	0
4507	8	0.3	6.2	3.7	2.5	1.5	37	56	165	0
4508	8	0.5	7.1	3.8	3.3	1.2	75	74	481	0
4509	8	0.4	6.8	3.6	3.2	1.1	34	113	78	0
4510	8	0.4	7.2	3.9	3.3	1.2	37	63	85	0
4511	29	0.6	7.8	4.0	3.8	1.1	58	81	306	0
4512	29	0.5	8.1	4.2	3.9	1.1	54	50	114	0
4513	29	0.5	7.7	4.0	3.7	1.1	46	54	128	0
4514	29	0.5	7.4	3.8	3.6	1.1	47	69	188	1
4515	29	0.5	7.2	4.0	3.2	1.3	66	69	171	0

Appendix 10
Pathology Report

FINAL REPORT

Study Phase: Pathology

Test Site Reference No. 20181811

Testing Facility Study No. 2759-001

Sponsor Reference No. CLN7-001

TEST SITE:
Charles River Laboratories, Inc.
4025 Stirrup Creek Drive, Suite 150
Durham, NC 27703
United States

TESTING FACILITY:
Charles River Laboratories, Inc.
54943 North Main Street
Mattawan, MI 49071
United States

TABLE OF CONTENTS

LIST OF TABLES ... 3
LIST OF APPENDICES ... 3
QUALITY ASSURANCE STATEMENT ... 4
COMPLIANCE STATEMENT AND REPORT APPROVAL ... 5
1. SUMMARY ... 6
2. RESPONSIBLE PERSONNEL ... 6
3. INTRODUCTION ... 7
4. MATERIALS AND METHODS ... 7
4.1. Computerized Systems ... 8
4.2. Disposition of Study Materials ... 8
5. RESULTS AND DISCUSSIONS ... 8
5.1. Mortality ... 8
5.2. Gross (Macroscopic) Pathology ... 8
5.2.1. Terminal Euthanasia Animals (Day 8) ... 8
5.2.2. Terminal Euthanasia Animals (Day 29) ... 8
5.2.3. Terminal Euthanasia Animals (Day 91) ... 8
5.3. Organ Weights ... 9
5.3.1. Terminal Euthanasia Animals (Day 8) ... 9
5.3.2. Terminal Euthanasia Animals (Day 29) ... 9
5.3.3. Terminal Euthanasia Animals (Day 91) ... 9
5.4. Histopathology ... 10
5.4.1. Terminal Euthanasia (Day 8) ... 10
5.4.2. Terminal Euthanasia (Day 29) ... 12
5.4.3. Terminal Euthanasia (Day 91) ... 12
6. CONCLUSIONS ... 13
7. REFERENCES ... 14

LIST OF TABLES

Table 1 Summary of Macroscopic Pathology Findings (Day 8) 15
Table 2 Summary of Macroscopic Pathology Findings (Day 29) 17
Table 3 Summary of Macroscopic Pathology Findings (Day 91) 20
Table 4 Summary of Organ Weight Values (Day 8) 23
Table 5 Summary of Organ Weight Values (Day 29) 36
Table 6 Summary of Organ Weight Values (Day 91) 49
Table 7 Summary of Microscopic Pathology Findings (Day 8) 62
Table 8 Summary of Microscopic Pathology Findings (Day 29) 71
Table 9 Summary of Microscopic Pathology Findings (Day 91) 79

LIST OF APPENDICES

Appendix 1 Deviations 86
Appendix 2 Individual Organ Weight Values (Day 8) 88
Appendix 3 Individual Organ Weight Values (Day 29) 137
Appendix 4 Individual Organ Weight Values (Day 91) 186
Appendix 5 Individual Animal Data Macroscopic and Microscopic Pathology Findings (Day 8) 235
Appendix 6 Individual Animal Data Macroscopic and Microscopic Pathology Findings (Day 29) 276
Appendix 7 Individual Animal Data Macroscopic and Microscopic Pathology Findings (Day 91) 317

QUALITY ASSURANCE STATEMENT

Study Number: 2759-001

This phase has been audited by Quality Assurance in accordance with the applicable Good Laboratory Practice regulations. Reports were submitted in accordance with standard operating procedures as follows:

QA INSPECTION DATES

Date(s) of Audit	Phase(s) Audited	Dates Findings Submitted to: Principal Investigator	Principal Investigator Management	Study Director	Study Director Management
08-Mar-2019 - 09-Mar-2019	Phase Report - Pathology	11-Mar-2019	11-Mar-2019	11-Mar-2019	11-Mar-2019
28-May-2019	Phase Report - Pathology	28-May-2019	28-May-2019	28-May-2019	28-May-2019
02-Mar-2020	Final Phase Report - Pathology	02-Mar-2020	02-Mar-2020	02-Mar-2020	02-Mar-2020

Process-based inspections relevant to this study were conducted according to a predetermined schedule. The outcome of each inspection was reported to Management and, where relevant for processes seen as part of a study, the Study Director.

Facilities relevant to this study are included in Charles River's annual facility inspection programme. The outcome of each inspection is reported to Management.

DocuSigned by:

Enosha Simmons

Signer Name: Enosha Simmons
Signing Reason: I approve this document
Signing Time: 05-Mar-2020 | 13:30:36 EST
4CCF1BF93FB74CD4B33DC7AD5C1B296E

Enosha Simmons
Quality Assurance Auditor

COMPLIANCE STATEMENT AND REPORT APPROVAL

The pathology phase of this study conducted in the USA was performed in accordance with the U.S. Department of Health and Human Services, Food and Drug Administration, United States Code of Federal Regulations, Title 21, Part 58: Good Laboratory Practice for Nonclinical Laboratory Studies and as accepted by Regulatory Authorities throughout the European Union (OECD Principles of Good Laboratory Practice), Japan (MHLW), and other countries that are signatories to the OECD Mutual Acceptance of Data Agreement.

This phase of the study was conducted in accordance with the procedures described herein. All deviations (if any) authorized/acknowledged by the Study Director are documented in the Study Records. The report represents an accurate and complete record of the results obtained for this study phase.

There were no deviations from the above regulations that affected the overall integrity of this study phase or the interpretation of the phase results and conclusions.

DocuSigned by:
Odete R. Mendes

Signer Name: Odete R. Mendes
Signing Reason: I approve this document
Signing Time: 05-Mar-2020 | 13:32:54 EST
32FB759FC80E43D7A5A36F632DA48E05

Odete R. Mendes, DVM, PhD, DACVP, DABT
Principal Investigator, Anatomic Pathology

1. SUMMARY

The objective of this study was to further characterize the toxicity, biodistribution and gene expression of scAAV9/JeT-hCLN7opt-SV40pA.

The test article, scAAV9/JeT-hCLN7opt-SV40pA, or vehicle were administered by intrathecal injection (IT) to Sprague-Dawley rats once on Day 1 at doses of 0, $5x10^{11}$, $2x10^{12}$ or $6x10^{12}$ vg/animal with an observation period of up to 91 days. There were 4 groups of 5 animals/sex/group at all dose levels scheduled for euthanasia on Day 8, Day 29 or Day 91 of study. Necropsies were performed and organ weights were collected as were all tissues required for microscopic evaluation at all dose levels.

There were no main study unscheduled deaths. There were no test article related macroscopic (gross) observations. There were no toxicologically relevant organ weight changes observed on Days 8 and Day 29. On Day 91, increased thymus weights were observed in males in a dose dependent manner that were statistically significant at $\geq 6x10^{12}$ vg/animal. Increased thymus weights had no histopathological correlate and were considered to be of little toxicological significance.

On Day 8, splenic marginal zone depletion was observed in females at $6x10^{12}$ vg/animal. This transient finding was potentially secondary to stress associated with the test article administration procedure and therefore had limited toxicological relevance. Also limited to Day 8, were focal gliosis and perivascular infiltrates observed in the lumbar and/or the thoracic spinal cord in individual females treated at $2x10^{12}$ vg/animal. These were considered local reactivity to the administration procedure and were not considered to be directly attributable to test article.

Perivascular mononuclear infiltrates in the right and/or left lumbar ganglia were observed with a comparable incidence across treatment groups, and their incidence and severity decreased by Day 91. They are attributed to the administration procedure and not directly to a test article effect.

In conclusion, intrathecal administration of scAAV9/JeT-hCLN7opt-SV40pA once on Day 1 to rats at doses of 0, $5x10^{11}$, $2x10^{12}$ or $6x10^{12}$ vg/animal with an observation period of up to 91 days resulted in no unscheduled deaths. There were no test article related macroscopic findings. Increased thymus weights were significant in males at $6x10^{12}$ vg/animal however, without a histopathological correlate, they were considered to have little toxicological significance. There were no microscopic changes directly attributable to the administration of scAAV9/JeT-hCLN7opt-SV40pA.

2. RESPONSIBLE PERSONNEL

Principal Investigator,<br>Anatomic Pathology	Odete R. Mendes, DVM, PhD, DACVP, DABT<br>Charles River Laboratories, Inc.<br>Durham, North Carolina

Test Site Management | Danielle L. Brown, DVM, MS, DACVP, DABT, FIATP
Charles River Laboratories, Inc.
Durham, North Carolina

3. INTRODUCTION

This report presents the pathology findings in Sprague Dawley rats assigned to Study No. 2759-001. The objective of this study was to further characterize the toxicity, biodistribution, and gene expression of the test article. The test article was being developed for treatment of the neurodegenerative Batten Disease (CLN7 subtype).

The study was sponsored by Foundation for Batten Hope, Dallas, TX. Sarah Davis, BS, LATG served as the Study Director.

4. MATERIALS AND METHODS

Experimental procedures applicable to pathology investigations are summarized in Text Table 1. Deviations to the pathology procedures performed by the Test Site are listed in Appendix 1.

Text Table 1
Experimental Design

Group	Treatment	Dose Level (vg/animal)	Dose Volume (µL)	Dose Concentration (vg/ µL)	Number of Animals: Necropsy Day 8 M	Necropsy Day 8 F	Necropsy Day 29 M	Necropsy Day 29 F	Necropsy Day 91 M	Necropsy Day 91 F
1	Vehicle	0	60 µL	0	5	5	5	5	5	5
2	Test Article Low Dose	$5x10^{11}$	20 µL	$2.5x10^{10}$	5	5	5	5	5	5
3	Test Article Middle Dose	$2x10^{12}$	20 µL	$1x10^{11}$	5	5	5	5	5	5
4	Test Article High Dose	$6x10^{12}$	60 µL	$1x10^{11}$	5	5	5	5	5	5

All main study animals were submitted for necropsy on Day 8, Day 29 or Day 91 (Terminal Euthanasia). Necropsies were performed and organ weights were collected by Charles River Laboratories, Inc., Mattawan, MI personnel. Statistical analysis of organ weight data was performed by the Testing Facility. Tissues required for microscopic evaluation which included thymus added by amendment, were trimmed, processed routinely, embedded in paraffin, and stained with hematoxylin and eosin by Charles River Laboratories, Inc., Mattawan, MI. Microscopic evaluation was conducted by the Principal Investigator, a board-certified veterinary pathologist, on all protocol-specified tissues from all animals that were evaluated by light microscopy.

Tissues that were supposed to be microscopically evaluated per protocol but were not available on the slide (and therefore not evaluated) are listed in the Individual Animal Data of the pathology report as not present. These missing tissues did not affect the outcome or

interpretation of the pathology portion of the study because the number of tissues examined from each treatment group was sufficient for interpretation.

4.1. Computerized Systems

Critical computerized systems used in the study by the Test Site are listed in Text Table 2.

Text Table 2
Computerized Systems

System Name	Version No.	Description of Data Collected and/or Analyzed
Provantis	9	Histopathology
Deviation Information Library	2.1	Deviations (if any)
Share Document Management System	1.0	Reporting
DocuSign	19	Collection of Part 11 compliant signature

4.2. Disposition of Study Materials

All study-specific raw data, pathology materials, and documentation and Final Report generated from this study phase are to be sent to Charles River Laboratories, Inc., Mattawan, MI for archiving. Study materials will be retained for a period of 1 year following issue of the audited Draft Report. Electronic Provantis data generated by the Test Site will be archived, and the software and hardware required to produce it in a readable form will be maintained and available. The electronic Provantis data will be archived in MPI Research, Mattawan, MI. Report files stored on the SHARE Document Management System (SDMS) will be archived in Charles River Laboratories, Inc., Wilmington, MA.

5. RESULTS AND DISCUSSIONS

5.1. Mortality

There were no main study unscheduled deaths during this study.

5.2. Gross (Macroscopic) Pathology

5.2.1. Terminal Euthanasia Animals (Day 8)

(Table 1 and Appendix 5)

No gross (macroscopic) findings were noted for male or female animals at this interval.

5.2.2. Terminal Euthanasia Animals (Day 29)

(Table 2 and Appendix 6)

No test article-related gross findings were noted. The gross findings observed were considered incidental, of the nature commonly observed in this strain and age of rat and/or were of similar incidence in control and treated animals and, therefore, were considered unrelated to administration of scAAV9/JeT-hCLN7opt-SV40pA.

5.2.3. Terminal Euthanasia Animals (Day 91)

(Table 3 and Appendix 7)

No test article-related gross findings were noted. The gross findings observed were considered incidental, of the nature commonly observed in this strain and age of rat and/or were of similar incidence in control and treated animals and, therefore, were considered unrelated to administration of scAAV9/JeT-hCLN7opt-SV40pA.

5.3. Organ Weights

5.3.1. Terminal Euthanasia Animals (Day 8)

(Table 4 and Appendix 2)

No test article-related organ weight changes were noted. There were isolated organ weight values that were different from their respective controls. There were, however, no patterns, trends, or correlating data to suggest these values were toxicologically relevant. Thus, the organ weight differences observed were considered incidental and unrelated to administration of scAAV9/JeT-hCLN7opt-SV40pA.

5.3.2. Terminal Euthanasia Animals (Day 29)

(Table 5 and Appendix 3)

Potential test article-related organ weight changes were noted at the end of Day 29 and are summarized in Text Table 3.

Text Table 3
Summary of Organ Weight Data – Terminal Euthanasia (Day 29)

	Males		
**Group**	**2**	**3**	**4**
**Dose (vg/day)**	**$5x10^{11}$**	**$2x10^{12}$**	**$6x10^{12}$**
**No. Animals per Group**	**5**	**5**	**5**
**Thyroid (No. Weighed)[a]**	**5**	**5**	**5**
Absolute value	-11.7	**-22.5**	**-22.3**
% of body weight	-12.7	**-20.0**	**-23.1**
% of brain weight	-11.3	**-21.7**	**-23.4**

[a] All values expressed as percent difference of control group means.
Based upon statistical analysis of group means, values highlighted in bold are significantly different from control group – P ≤ 0.01; refer to data tables for actual significance levels and tests used.

Decreased thyroid weights in male animals were statistically significant at ≥ $2x10^{12}$ vg/animal. As per protocol, the thyroid gland was not evaluated microscopically.

No other test article-related organ weight changes were noted. There were other isolated organ weight values that were statistically different from their respective controls. There were, however, no patterns, trends, or correlating data to suggest these values were toxicologically relevant. Thus, other organ weight differences observed were considered incidental and unrelated to administration of scAAV9/JeT-hCLN7opt-SV40pA.

5.3.3. Terminal Euthanasia Animals (Day 91)

(Table 6 and Appendix 4)

Potential test article-related organ weight changes noted at the Day 91 terminal euthanasia and potential test article-related organ weight changes noted at the Day 29 terminal euthanasia that were not observed at the end of Day 91 are summarized in Text Table 4.

Text Table 4
Summary of Organ Weight Data – Terminal Euthanasia (Day 91)

	Males		
**Group**	**2**	**3**	**4**
**Dose (vg/animal)**	**$5x10^{11}$**	**$2x10^{12}$**	**$6x10^{12}$**
**No. Animals per Group**	**5**	**5**	**5**
**Thymus (No. Weighed)[a]**	**5**	**5**	**5**
Absolute value	13.0	46.9	**64.6**
% of body weight	14.6	36.2	**56.0**
% of brain weight	16.2	52.7	**70.1**
**Thyroid (No. Weighed)**	**5**	**5**	**5**
Absolute value	-7.5	15.6	8.1
% of body weight	-6.9	6.8	9.4
% of brain weight	-6.0	19.2	10.4

[a] All values expressed as percent difference of control group means.
Based upon statistical analysis of group means, values highlighted in bold are significantly different from control group – P ≤ 0.05; refer to data tables for actual significance levels and tests used.

Increased thymus weights were observed in males in a dose dependent manner and became statistically significant at the highest dose. Increased thymus weights had no histopathological correlate and were considered to be of little toxicological significance.

Potential dose dependent decreases in thyroid weight that were observed in males on Day 29 were not observed on Day 91 and actual increases in thyroid weights were observed at $6x10^{12}$ vg/animal. These data, together with the small magnitude and transiency of the changes observed, support an unlikely relationship to treatment and no toxicological significance.

A statistical significant increase was observed for the absolute spleen of females treated at $6x10^{12}$ vg/animal. Spleen weight increases were of small magnitude, were not in a dose dependent manner and did not have a relevant correlating microscopic effect and were thus considered within expected biological variability for this organ and not related to test article administration.

No other test article-related organ weight changes were noted. There were other isolated organ weight values that were statistically different from their respective controls. There were, however, no patterns, trends, or correlating data to suggest these values were toxicologically relevant. Thus, other organ weight differences observed were considered incidental and unrelated to administration of scAAV9/JeT-hCLN7opt-SV40pA.

5.4. Histopathology

5.4.1. Terminal Euthanasia (Day 8)

(Table 7 and Appendix 5)

Microscopic findings are summarized in Text Table 5.

Text Table 5
Summary of Microscopic Findings – Terminal Euthanasia (Day 8)

	Males				Females			
**Group**	1	**2**	**3**	**4**	**1**	**2**	**3**	**4**
**Dose (vg/animal)**	**0**	**5x10^{11}**	**2x10^{12}**	**6x10^{12}**	**0**	**5x10^{11}**	**2x10^{12}**	**6x10^{12}**
**No. Animals Examined**	**5**	**5**	**5**	**5**	**5**	**5**	**5**	**5**
**Dorsal Root Ganglia, Lumbar right (No. Examined)**	(5)	(5)	(5)	(5)	(5)	(5)	(5)	(5)
Infiltration, mononuclear perivascular								
Minimal	0	0	1	1	1	1	1	0
**Dorsal Root Ganglia, Lumbar left (No. Examined)**	(5)	(5)	(5)	(5)	(4)	(5)	(5)	(5)
Infiltration, mononuclear perivascular								
Minimal	1	1	0	1	0	1	0	1
**Spleen (No. Examined)**	(5)	(5)	(5)	(5)	(5)	(5)	(5)	(5)
Depletion, marginal zone								
Minimal	0	0	0	0	0	0	0	3
Mild	0	0	0	0	0	0	0	1
Marked	0	0	0	0	0	0	0	1
**Spinal Cord, Lumbar (No. Examined)**	(5)	(5)	(5)	(5)	(5)	(5)	(5)	(5)
Gliosis								
Minimal	0	0	0	0	0	0	2	0
Infiltration, mononuclear perivascular								
Minimal	0	0	0	0	0	0	2	0
**Spinal Cord, Thoracic (No. Examined)**	(5)	(5)	(5)	(5)	(5)	(5)	(5)	(5)
Gliosis								
Minimal	0	0	0	0	0	0	1	0
Infiltration, mononuclear perivascular								
Minimal	0	0	0	0	0	0	1	0

Minimal to marked depletion of the splenic marginal zone was observed in females treated at 6x10^{12} vg/animal. Decrease in B lymphocytes (main component) of the marginal zone have been associated with stress related response in rodent preclinical studies.[1] Additionally, this finding was not observed at latter time points and was interpreted likely a transient secondary effect to test article administration and therefore of limited toxicological relevance.

Minimal perivascular mononuclear infiltrates in the right and/or left lumbar ganglia were observed with a comparable incidence across treatment groups, these were attributed to the administration procedure and not directly to test article administration.

Likewise, focal minimal gliosis and minimal perivascular infiltrates were observed in the lumbar and/or the thoracic spinal cord in individual females treated at 2x10^{12} vg/animal. These were secondary reactivity to the administration procedure and were not considered to be directly attributable to a test article effect.

Other microscopic findings observed were considered incidental, of the nature commonly observed in this strain and age of rat, and/or were of similar incidence and severity in control and

treated animals and, therefore, were considered unrelated to administration of scAAV9/JeT-hCLN7opt-SV40pA.

5.4.2. Terminal Euthanasia (Day 29)

(Table 8 and Appendix 6)

Microscopic findings noted at the end of Day 29 and are summarized in Text Table 6.

Text Table 6
Summary of Microscopic Findings – Terminal Euthanasia (Day 29)

	Males				Females			
**Group**	1	**2**	**3**	**4**	**1**	**2**	**3**	**4**
**Dose (vg/animal)**	**0**	**$5x10^{11}$**	**$2x10^{12}$**	**$6x10^{12}$**	**0**	**$5x10^{11}$**	**$2x10^{12}$**	**$6x10^{12}$**
**No. Animals Examined**	**5**	**5**	**5**	**5**	**5**	**5**	**5**	**5**
**Dorsal Root Ganglia, Lumbar right (No. Examined)**	(5)	(5)	(5)	(5)	(5)	(5)	(5)	(5)
Infiltration, mononuclear perivascular								
Minimal	0	1	1	2	1	1	0	1
Mild	0	1	0	0	0	0	0	0
**Dorsal Root Ganglia, Lumbar left (No. Examined)**	(5)	(5)	(5)	(5)	(5)	(5)	(5)	(5)
Infiltration, mononuclear perivascular								
Minimal	0	1	1	1	0	1	2	2

Minimal perivascular mononuclear infiltrates in the right and/or left lumbar ganglia were observed with an overall comparable incidence across treatment groups and were attributed to the administration procedure and not directly to test article administration. In the groups administered the test article, there was a potential very slight trend, that had no clear dose response, for increased finding incidence and severity that could possibly be attributable to reactivity to the presence of the test article.

Other microscopic findings observed were considered incidental, of the nature commonly observed in this strain and age of rat, and/or were of similar incidence and severity in control and treated animals and, therefore, were considered unrelated to administration of scAAV9/JeT-hCLN7opt-SV40pA.

5.4.3. Terminal Euthanasia (Day 91)

(Table 9 and Appendix 7)

Microscopic findings noted at the end of Day 91 and are summarized in Text Table 7.

Text Table 7
Summary of Microscopic Findings – Terminal Euthanasia (Day 91)

	Males				Females			
**Group**	1	**2**	**3**	**4**	**1**	**2**	**3**	**4**
**Dose (vg/animal)**	**0**	**$5x10^{11}$**	**$2x10^{12}$**	**$6x10^{12}$**	**0**	**$5x10^{11}$**	**$2x10^{12}$**	**$6x10^{12}$**
**No. Animals Examined**	**5**	**5**	**5**	**5**	**5**	**5**	**5**	**5**
**Dorsal Root Ganglia, Lumbar right (No. Examined)**	(5)	(5)	(5)	(5)	(5)	(5)	(5)	(5)
Infiltration, mononuclear perivascular								
Minimal	0	0	0	0	0	0	3	0
**Dorsal Root Ganglia, Lumbar left (No. Examined)**	(5)	(5)	(5)	(5)	(5)	(5)	(5)	(5)
Infiltration, mononuclear perivascular								
Minimal	0	1	0	0	1	0	2	0

Minimal perivascular mononuclear infiltrates in the right and/or left lumbar ganglia were observed with an overall comparable incidence across treatment groups, that decreased in severity and incidence by Day 91. These were attributed to the administration procedure and not directly to test article administration.

Other microscopic findings observed were considered incidental, of the nature commonly observed in this strain and age of rat, and/or were of similar incidence and severity in control and treated animals and, therefore, were considered unrelated to administration of scAAV9/JeT-hCLN7opt-SV40pA.

6. CONCLUSIONS

Intrathecal administration of scAAV9/JeT-hCLN7opt-SV40pA once on Day 1 to rats at doses of 0, $5x10^{11}$, $2x10^{12}$ or $6x10^{12}$ vg/animal with an observation period of up to 91 days resulted in no unscheduled deaths. There were no test article related macroscopic findings. Increased thymus weights were significant in males at $6x10^{12}$ vg/animal however, without a histopathological correlate, they were considered to have little toxicological significance. There were no microscopic changes directly attributable to the administration of scAAV9/JeT-hCLN7opt-SV40pA.

7. REFERENCES

1 Everds NE, Snyder PW, Bailey KL, Bolon B, Creasy DM, Foley GL, Rosol TJ, Sellers T., Interpreting stress responses during routine toxicity studies: a review of the biology, impact, and assessment., Toxicol Pathol. 2013;41(4):560-614.

Table 1
Summary of Macroscopic Pathology Findings (Day 8)

2759-001
A Single Dose Toxicity Study of Test Article Administered by Intrathecal Injection in Rats

Summary of Macroscopic Observations

No Data Found

Table 2
Summary of Macroscopic Pathology Findings (Day 29)

2759-001
A Single Dose Toxicity Study of Test Article Administered by Intrathecal Injection in Rats

Summary of Macroscopic Observations

Sex: Male	Vehicle	Low Dose	Mid Dose	High Dose
	SS	SS	SS	SS
Number of Animals:	5	5	5	5
**liver**				
Submitted	5	5	5	5
focus/foci, red	1	-	-	-
**skin**				
Submitted	5	5	5	5
abrasion/scab	-	-	-	1

2759-001
A Single Dose Toxicity Study of Test Article Administered by Intrathecal Injection in Rats

Summary of Macroscopic Observations

Sex: Female	Vehicle	Low Dose	Mid Dose	High Dose
	SS	SS	SS	SS
Number of Animals:	5	5	5	5
**liver**				
Submitted	5	5	5	5
**skin**				
Submitted	5	5	5	5

Table 3
Summary of Macroscopic Pathology Findings (Day 91)

2759-001
A Single Dose Toxicity Study of Test Article Administered by Intrathecal Injection in Rats

Summary of Macroscopic Observations

Sex: Male	Vehicle SS	Low Dose SS	Mid Dose SS	High Dose SS
Number of Animals:	5	5	5	5
**kidneys**				
Submitted	5	5	5	5
irregular surface	1	-	-	-
.... approximately 50% affected	1	-	-	-

2759-001
A Single Dose Toxicity Study of Test Article Administered by Intrathecal Injection in Rats

Summary of Macroscopic Observations

Sex: Female	Vehicle	Low Dose	Mid Dose	High Dose
	SS	SS	SS	SS
Number of Animals:	5	5	5	5
**kidneys**				
Submitted	5	5	5	5

Table 4
Summary of Organ Weight Values (Day 8)

2759-001
A Single Dose Toxicity Study of Test Article Administered by Intrathecal Injection in Rats

Summary of Organ Weight Values
Day 8

Day: 8 Relative to Start Date

Sex: Male		Vehicle	Low Dose	Mid Dose	High Dose
Body [g] Weight (g)	Mean	321.8	306.6	320.2	310.4
	SD	11.76	16.53	11.50	15.37
	N	5	5	5	5
	%Diff	-	-4.7	-0.5	-3.5
Brain [g] (g)	Mean	1.9250	1.9042	1.9590	1.9598
	SD	0.14321	0.05512	0.05030	0.09907
	N	5	5	5	5
	%Diff	-	-1.1	1.8	1.8
Brain/BWt [g] (%)	Mean	0.59829	0.62181	0.61271	0.63176
	SD	0.041531	0.019527	0.033746	0.026256
	N	5	5	5	5
	%Diff	-	3.9	2.4	5.6
Adrenal [g] glands (g)	Mean	0.0584	0.0626	0.0666	0.0608
	SD	0.01555	0.01909	0.01252	0.01207
	N	5	5	5	5
	%Diff	-	7.2	14.0	4.1
Adrenal gl/ [g] BWt (%)	Mean	0.01827	0.02025	0.02073	0.01950
	SD	0.005454	0.005149	0.003249	0.003207
	N	5	5	5	5
	%Diff	-	10.8	13.4	6.7
Adrenal gl/ [g] BrWt (ratio)	Mean	0.03087	0.03276	0.03407	0.03101
	SD	0.010703	0.009334	0.006905	0.005806
	N	5	5	5	5
	%Diff	-	6.1	10.4	0.5
Epididymides [g] (g)	Mean	0.7040	0.6774	0.7456	0.7116
	SD	0.07069	0.05369	0.14500	0.06497
	N	5	5	5	5
	%Diff	-	-3.8	5.9	1.1
Epididymides [g1] /BWt (%)	Mean	0.21938	0.22088	0.23265	0.22961
	SD	0.027291	0.011846	0.043025	0.023469
	N	5	5	5	5
	%Diff	-	0.7	6.0	4.7

[g] - Anova & Dunnett
[g1] - Kruskal-Wallis & Dunn

2759-001
A Single Dose Toxicity Study of Test Article Administered by Intrathecal Injection in Rats

Summary of Organ Weight Values
Day 8

Day: 8 Relative to Start Date

Sex: Male		Vehicle	Low Dose	Mid Dose	High Dose
Epididymides [g] /BrWt (ratio)	Mean	0.36707	0.35544	0.38045	0.36482
	SD	0.042936	0.020845	0.073322	0.049635
	N	5	5	5	5
	%Diff	-	-3.2	3.6	-0.6
Heart [g] (g)	Mean	1.3744	1.3384	1.4394	1.5150
	SD	0.22698	0.25882	0.21808	0.31696
	N	5	5	5	5
	%Diff	-	-2.6	4.7	10.2
Heart/BWt [g] (%)	Mean	0.42804	0.43471	0.44934	0.48612
	SD	0.076194	0.066392	0.064680	0.085220
	N	5	5	5	5
	%Diff	-	1.6	5.0	13.6
Heart/BrWt [g] (ratio)	Mean	0.71219	0.70116	0.73641	0.77022
	SD	0.085336	0.121792	0.121296	0.135657
	N	5	5	5	5
	%Diff	-	-1.5	3.4	8.1
Kidneys [g] (g)	Mean	3.1488	2.7654	2.9886	2.8942
	SD	0.21421	0.34834	0.14406	0.28079
	N	5	5	5	5
	%Diff	-	-12.2	-5.1	-8.1
Kidneys/BWt [g] (%)	Mean	0.97818	0.89955	0.93353	0.93088
	SD	0.049613	0.069633	0.036884	0.049584
	N	5	5	5	5
	%Diff	-	-8.0	-4.6	-4.8
Kidneys/BrWt [g] (ratio)	Mean	1.63823	1.44960	1.52640	1.47748
	SD	0.086479	0.147780	0.085331	0.129823
	N	5	5	5	5
	%Diff	-	-11.5	-6.8	-9.8
Liver [g] (g)	Mean	14.7970	12.4866 a	13.7358	12.9754
	SD	1.10214	1.01091	1.14122	1.37132
	N	5	5	5	5
	%Diff	-	-15.6	-7.2	-12.3

[g] - Anova & Dunnett: a = p < 0.05

2759-001
A Single Dose Toxicity Study of Test Article Administered by Intrathecal Injection in Rats

Summary of Organ Weight Values
Day 8

Day: 8 Relative to Start Date

Sex: Male		Vehicle	Low Dose	Mid Dose	High Dose
Liver/BWt [g] (%)	Mean	4.59699	4.07372 a	4.28599	4.17153
	SD	0.271405	0.276635	0.250328	0.250522
	N	5	5	5	5
	%Diff	-	-11.4	-6.8	-9.3
Liver/BrWt [g] (ratio)	Mean	7.72367	6.55271 a	7.02330	6.62163 a
	SD	0.818068	0.414568	0.715186	0.614430
	N	5	5	5	5
	%Diff	-	-15.2	-9.1	-14.3
Lung [g] (g)	Mean	1.6148	1.6796	1.7722	1.7322
	SD	0.16046	0.30544	0.11333	0.21244
	N	5	5	5	5
	%Diff	-	4.0	9.7	7.3
Lung/BWt [g] (%)	Mean	0.50198	0.54718	0.55393	0.55648
	SD	0.048270	0.092405	0.038710	0.042788
	N	5	5	5	5
	%Diff	-	9.0	10.3	10.9
Lung/BrWt [g] (ratio)	Mean	0.83956	0.88058	0.90474	0.88284
	SD	0.066310	0.148320	0.054754	0.086066
	N	5	5	5	5
	%Diff	-	4.9	7.8	5.2
Pituitary [g] (fixed) (g)	Mean	0.01570	0.01478	0.01486	0.01450
	SD	0.002559	0.000820	0.001163	0.002266
	N	5	5	5	5
	%Diff	-	-5.9	-5.4	-7.6
Pituitary [g] (fixed)/BWt (%)	Mean	0.00489	0.00483	0.00464	0.00468
	SD	0.000874	0.000249	0.000257	0.000768
	N	5	5	5	5
	%Diff	-	-1.4	-5.2	-4.4
Pituitary [g] (fixed)/BrWt (ratio)	Mean	0.00815	0.00776	0.00760	0.00738
	SD	0.001145	0.000287	0.000755	0.000993
	N	5	5	5	5
	%Diff	-	-4.8	-6.8	-9.4

[g] - Anova & Dunnett: a = p < 0.05

2759-001
A Single Dose Toxicity Study of Test Article Administered by Intrathecal Injection in Rats

Summary of Organ Weight Values
Day 8

Day: 8 Relative to Start Date

Sex: Male		Vehicle	Low Dose	Mid Dose	High Dose
Prostate [g] gland (g)	Mean	0.6970	0.6400	0.6894	0.6834
	SD	0.13127	0.08620	0.19504	0.09921
	N	5	5	5	5
	%Diff	-	-8.2	-1.1	-2.0
Prostate [g] gland/BWt (%)	Mean	0.21768	0.20879	0.21483	0.22056
	SD	0.046926	0.026595	0.058632	0.033378
	N	5	5	5	5
	%Diff	-	-4.1	-1.3	1.3
Prostate [g] gland/BrWt (ratio)	Mean	0.36559	0.33593	0.35144	0.34987
	SD	0.087511	0.042385	0.096141	0.057349
	N	5	5	5	5
	%Diff	-	-8.1	-3.9	-4.3
Sal, mand/ [g] sub., rt (g)	Mean	0.2936	0.2726	0.3172	0.3024
	SD	0.02323	0.03706	0.02307	0.02386
	N	5	5	5	5
	%Diff	-	-7.2	8.0	3.0
Sal gl mand/ [g] sub., rt/BWt (%)	Mean	0.09152	0.08864	0.09916	0.09737
	SD	0.010361	0.007746	0.007996	0.004918
	N	5	5	5	5
	%Diff	-	-3.1	8.3	6.4
Sal gl mand/ [g] sub.,rt/BrWt (ratio)	Mean	0.15356	0.14287	0.16184	0.15455
	SD	0.021315	0.016044	0.009429	0.013311
	N	5	5	5	5
	%Diff	-	-7.0	5.4	0.6
Spleen [g] (g)	Mean	0.8182	0.8146	0.8498	0.8972
	SD	0.09743	0.11673	0.02588	0.09855
	N	5	5	5	5
	%Diff	-	-0.4	3.9	9.7
Spleen/BWt [g] (%)	Mean	0.25455	0.26557	0.26560	0.28909
	SD	0.031840	0.034098	0.010662	0.028318
	N	5	5	5	5
	%Diff	-	4.3	4.3	13.6

[g] - Anova & Dunnett

2759-001
A Single Dose Toxicity Study of Test Article Administered by Intrathecal Injection in Rats

Summary of Organ Weight Values
Day 8

Day: 8 Relative to Start Date

Sex: Male		Vehicle	Low Dose	Mid Dose	High Dose
Spleen/BrWt [g] (ratio)	Mean	0.42548	0.42776	0.43415	0.45884
	SD	0.042343	0.059126	0.021018	0.056306
	N	5	5	5	5
	%Diff	-	0.5	2.0	7.8
Testes [g] (g)	Mean	3.1754	3.1834	3.3376	3.2362
	SD	0.27554	0.43164	0.22360	0.12564
	N	5	5	5	5
	%Diff	-	0.3	5.1	1.9
Testes/BWt [g] (%)	Mean	0.98717	1.03523	1.04283	1.04514
	SD	0.085863	0.088275	0.069564	0.073849
	N	5	5	5	5
	%Diff	-	4.9	5.6	5.9
Testes/BrWt [g] (ratio)	Mean	1.64870	1.66911	1.70382	1.65486
	SD	0.042206	0.191492	0.108965	0.108654
	N	5	5	5	5
	%Diff	-	1.2	3.3	0.4
Thymus [g] (g)	Mean	0.6942	0.6364	0.6846	0.6746
	SD	0.17365	0.21561	0.07953	0.17988
	N	5	5	5	5
	%Diff	-	-8.3	-1.4	-2.8
Thymus/BWt [g] (%)	Mean	0.21480	0.20517	0.21455	0.21684
	SD	0.047341	0.056819	0.030529	0.054558
	N	5	5	5	5
	%Diff	-	-4.5	-0.1	1.0
Thymus/BrWt [g] (ratio)	Mean	0.36275	0.33232	0.34935	0.34202
	SD	0.095791	0.104471	0.039050	0.079316
	N	5	5	5	5
	%Diff	-	-8.4	-3.7	-5.7
Thyroid/para [g] (fixed) (g)	Mean	0.02548	0.02202	0.02342	0.02216
	SD	0.002662	0.002121	0.003149	0.004787
	N	5	5	5	5
	%Diff	-	-13.6	-8.1	-13.0

[g] - Anova & Dunnett

2759-001
A Single Dose Toxicity Study of Test Article Administered by Intrathecal Injection in Rats

Summary of Organ Weight Values
Day 8

Day: 8 Relative to Start Date

Sex: Male		Vehicle	Low Dose	Mid Dose	High Dose
Thyroid/para [g] (fixed)/BWt (%)	Mean	0.00794	0.00718	0.00734	0.00718
	SD	0.001024	0.000511	0.001173	0.001737
	N	5	5	5	5
	%Diff	-	-9.6	-7.5	-9.6
Thyroid/para [g] (fixed)/BrWt (ratio)	Mean	0.01331	0.01155	0.01195	0.01139
	SD	0.001825	0.000828	0.001542	0.002789
	N	5	5	5	5
	%Diff	-	-13.2	-10.2	-14.4

[g] - Anova & Dunnett

2759-001
A Single Dose Toxicity Study of Test Article Administered by Intrathecal Injection in Rats

Summary of Organ Weight Values
Day 8

Day: 8 Relative to Start Date

Sex: Female		Vehicle	Low Dose	Mid Dose	High Dose
Body [g] Weight (g)	Mean	191.4	196.0	193.8	185.6
	SD	12.30	15.22	6.83	12.40
	N	5	5	5	5
	%Diff	-	2.4	1.3	-3.0
Brain [g] (g)	Mean	1.8246	1.8668	1.8400	1.8338
	SD	0.07151	0.02067	0.07937	0.12045
	N	5	5	5	5
	%Diff	-	2.3	0.8	0.5
Brain/BWt [g] (%)	Mean	0.95775	0.95700	0.95019	0.98859
	SD	0.092418	0.074080	0.048469	0.033125
	N	5	5	5	5
	%Diff	-	-0.1	-0.8	3.2
Adrenal [g] glands (g)	Mean	0.0664	0.0572	0.0714	0.0648
	SD	0.01199	0.00572	0.01659	0.01238
	N	5	5	5	5
	%Diff	-	-13.9	7.5	-2.4
Adrenal gl/ [g] BWt (%)	Mean	0.03488	0.02929	0.03693	0.03478
	SD	0.007186	0.003355	0.008987	0.005066
	N	5	5	5	5
	%Diff	-	-16.0	5.9	-0.3
Adrenal gl/ [g] BrWt (ratio)	Mean	0.03644	0.03064	0.03873	0.03526
	SD	0.006716	0.003062	0.008094	0.005591
	N	5	5	5	5
	%Diff	-	-15.9	6.3	-3.2
Heart [g] (g)	Mean	0.9740	0.8442	0.8326	0.9424
	SD	0.18517	0.05959	0.08349	0.13109
	N	5	5	5	5
	%Diff	-	-13.3	-14.5	-3.2
Heart/BWt [g1] (%)	Mean	0.51426	0.43213	0.43016	0.50844
	SD	0.130481	0.036902	0.046904	0.069265
	N	5	5	5	5
	%Diff	-	-16.0	-16.4	-1.1

[g] - Anova & Dunnett
[g1] - Anova & Dunnett(Log)

2759-001
A Single Dose Toxicity Study of Test Article Administered by Intrathecal Injection in Rats

Summary of Organ Weight Values
Day 8

Day: 8 Relative to Start Date

Sex: Female		Vehicle	Low Dose	Mid Dose	High Dose
Heart/BrWt [g] (ratio)	Mean	0.53214	0.45218	0.45240	0.51358
	SD	0.084997	0.030694	0.038741	0.060370
	N	5	5	5	5
	%Diff	-	-15.0	-15.0	-3.5
Kidneys [g] (g)	Mean	1.8720	1.8946	1.8834	1.8892
	SD	0.10707	0.15867	0.04244	0.11721
	N	5	5	5	5
	%Diff	-	1.2	0.6	0.9
Kidneys/BWt [g] (%)	Mean	0.98051	0.96823	0.97289	1.01866
	SD	0.072091	0.068824	0.043118	0.036504
	N	5	5	5	5
	%Diff	-	-1.3	-0.8	3.9
Kidneys/BrWt [g] (ratio)	Mean	1.02665	1.01456	1.02464	1.03164
	SD	0.059284	0.078791	0.035498	0.057641
	N	5	5	5	5
	%Diff	-	-1.2	-0.2	0.5
Liver [g] (g)	Mean	8.1428	7.7658	8.3960	7.9046
	SD	0.59964	0.59726	0.31692	0.84024
	N	5	5	5	5
	%Diff	-	-4.6	3.1	-2.9
Liver/BWt [g] (%)	Mean	4.26014	3.96755	4.33906	4.25027
	SD	0.290120	0.232501	0.270728	0.184457
	N	5	5	5	5
	%Diff	-	-6.9	1.9	-0.2
Liver/BrWt [g] (ratio)	Mean	4.46613	4.15992	4.57173	4.30621
	SD	0.337197	0.314507	0.303256	0.300857
	N	5	5	5	5
	%Diff	-	-6.9	2.4	-3.6
Lung [g] (g)	Mean	1.2312	1.2368	1.2016	1.2882
	SD	0.14462	0.13521	0.18374	0.15127
	N	5	5	5	5
	%Diff	-	0.5	-2.4	4.6

[g] - Anova & Dunnett

2759-001
A Single Dose Toxicity Study of Test Article Administered by Intrathecal Injection in Rats

Summary of Organ Weight Values
Day 8

Day: 8 Relative to Start Date

Sex: Female		Vehicle	Low Dose	Mid Dose	High Dose
Lung/BWt [g] (%)	Mean	0.64237	0.63212	0.62062	0.69316
	SD	0.047285	0.061549	0.097857	0.053389
	N	5	5	5	5
	%Diff	-	-1.6	-3.4	7.9
Lung/BrWt [g1] (ratio)	Mean	0.67574	0.66315	0.65236	0.70065
	SD	0.084828	0.079772	0.086770	0.038560
	N	5	5	5	5
	%Diff	-	-1.9	-3.5	3.7
Ovaries [g] (g)	Mean	0.0880	0.0676	0.0844	0.0726
	SD	0.01762	0.01260	0.01623	0.01668
	N	5	5	5	5
	%Diff	-	-23.2	-4.1	-17.5
Ovaries/BWt [g] (%)	Mean	0.04658	0.03464	0.04368	0.03910
	SD	0.012563	0.006885	0.008983	0.008451
	N	5	5	5	5
	%Diff	-	-25.6	-6.2	-16.1
Ovaries/BrWt [g] (ratio)	Mean	0.04807	0.03618	0.04605	0.03951
	SD	0.008244	0.006543	0.009752	0.008158
	N	5	5	5	5
	%Diff	-	-24.7	-4.2	-17.8
Oviducts [g] (g)	Mean	0.0206	0.0236	0.0270	0.0210
	SD	0.00581	0.00167	0.00752	0.00245
	N	5	5	5	5
	%Diff	-	14.6	31.1	1.9
Oviducts/BWt [g] (%)	Mean	0.01077	0.01210	0.01397	0.01130
	SD	0.002961	0.001313	0.004026	0.000853
	N	5	5	5	5
	%Diff	-	12.4	29.7	4.9
Oviducts/ [g] BrWt (ratio)	Mean	0.01133	0.01264	0.01467	0.01143
	SD	0.003274	0.000797	0.003881	0.000773
	N	5	5	5	5
	%Diff	-	11.6	29.5	0.9

[g] - Anova & Dunnett
[g1] - Anova & Dunnett(Log)

2759-001
A Single Dose Toxicity Study of Test Article Administered by Intrathecal Injection in Rats

Summary of Organ Weight Values
Day 8

Day: 8 Relative to Start Date

Sex: Female		Vehicle	Low Dose	Mid Dose	High Dose
Pituitary [g] (fixed) (g)	Mean	0.01734	0.01740	0.01752	0.01732
	SD	0.001992	0.002529	0.002044	0.003518
	N	5	5	5	5
	%Diff	-	0.3	1.0	-0.1
Pituitary [g] (fixed)/BWt (%)	Mean	0.00910	0.00892	0.00902	0.00928
	SD	0.001216	0.001500	0.000828	0.001408
	N	5	5	5	5
	%Diff	-	-1.9	-0.8	2.1
Pituitary [g] (fixed)/BrWt (ratio)	Mean	0.00952	0.00931	0.00951	0.00942
	SD	0.001269	0.001274	0.000928	0.001657
	N	5	5	5	5
	%Diff	-	-2.2	-0.1	-1.1
Sal, mand/ [g1] sub., rt (g)	Mean	0.2412	0.1878	0.1960	0.1882
	SD	0.12072	0.01462	0.01639	0.03306
	N	5	5	5	5
	%Diff	-	-22.1	-18.7	-22.0
Sal gl mand/ [g1] sub., rt/BWt (%)	Mean	0.12584	0.09606	0.10114	0.10088
	SD	0.060836	0.007667	0.007650	0.012167
	N	5	5	5	5
	%Diff	-	-23.7	-19.6	-19.8
Sal gl mand/ [g1] sub.,rt/BrWt (ratio)	Mean	0.13398	0.10062	0.10669	0.10234
	SD	0.072694	0.008182	0.010005	0.015035
	N	5	5	5	5
	%Diff	-	-24.9	-20.4	-23.6
Spleen [g] (g)	Mean	0.4628	0.4872	0.5172	0.4608
	SD	0.03819	0.09443	0.05676	0.04473
	N	5	5	5	5
	%Diff	-	5.3	11.8	-0.4
Spleen/BWt [g] (%)	Mean	0.24205	0.24714	0.26706	0.24914
	SD	0.017421	0.030136	0.029980	0.029329
	N	5	5	5	5
	%Diff	-	2.1	10.3	2.9

[g] - Anova & Dunnett
[g1] - Kruskal-Wallis & Dunn

2759-001
A Single Dose Toxicity Study of Test Article Administered by Intrathecal Injection in Rats

Summary of Organ Weight Values
Day 8

Day: 8 Relative to Start Date

Sex: Female		Vehicle	Low Dose	Mid Dose	High Dose
Spleen/BrWt [g] (ratio)	Mean	0.25413	0.26082	0.28125	0.25176
	SD	0.025916	0.049169	0.030142	0.025319
	N	5	5	5	5
	%Diff	-	2.6	10.7	-0.9
Thymus [g] (g)	Mean	0.5890	0.5122	0.5634	0.5590
	SD	0.13383	0.08400	0.12405	0.12014
	N	5	5	5	5
	%Diff	-	-13.0	-4.3	-5.1
Thymus/BWt [g] (%)	Mean	0.30865	0.26280	0.28993	0.30008
	SD	0.071288	0.047582	0.059201	0.053628
	N	5	5	5	5
	%Diff	-	-14.9	-6.1	-2.8
Thymus/BrWt [g] (ratio)	Mean	0.32417	0.27447	0.30613	0.30406
	SD	0.082442	0.045507	0.064732	0.056652
	N	5	5	5	5
	%Diff	-	-15.3	-5.6	-6.2
Thyroid/para [g1] (fixed) (g)	Mean	0.01982	0.01934	0.02112	0.01978
	SD	0.002629	0.001210	0.005396	0.003235
	N	5	5	5	5
	%Diff	-	-2.4	6.6	-0.2
Thyroid/para [g] (fixed)/BWt (%)	Mean	0.01040	0.00992	0.01092	0.01067
	SD	0.001547	0.001054	0.002889	0.001602
	N	5	5	5	5
	%Diff	-	-4.6	5.0	2.6
Thyroid/para [g2] (fixed)/BrWt (ratio)	Mean	0.01087	0.01036	0.01152	0.01076
	SD	0.001440	0.000701	0.003084	0.001343
	N	5	5	5	5
	%Diff	-	-4.7	6.0	-1.0
Uterus w/ [g1] cervix (g)	Mean	0.4918	0.4990	0.5222	0.4080
	SD	0.21611	0.21754	0.21041	0.02732
	N	5	5	5	5
	%Diff	-	1.5	6.2	-17.0

[g] - Anova & Dunnett
[g1] - Kruskal-Wallis & Dunn
[g2] - Anova & Dunnett(Log)

2759-001
A Single Dose Toxicity Study of Test Article Administered by Intrathecal Injection in Rats

Summary of Organ Weight Values
Day 8

Day: 8 Relative to Start Date

Sex: Female		Vehicle	Low Dose	Mid Dose	High Dose
Uterus w/ [g] cervix/BWt (%)	Mean	0.25730	0.25446	0.27043	0.22033
	SD	0.110911	0.103182	0.110807	0.016149
	N	5	5	5	5
	%Diff	-	-1.1	5.1	-14.4
Uterus w/ [g1] cervix/BrWt (ratio)	Mean	0.27186	0.26827	0.28342	0.22270
	SD	0.129218	0.120568	0.110140	0.011086
	N	5	5	5	5
	%Diff	-	-1.3	4.3	-18.1

[g] - Anova & Dunnett(Log)
[g1] - Kruskal-Wallis & Dunn

Table 5
Summary of Organ Weight Values (Day 29)

2759-001
A Single Dose Toxicity Study of Test Article Administered by Intrathecal Injection in Rats

Summary of Organ Weight Values
Day 29

Day: 29 Relative to Start Date

Sex: Male		Vehicle	Low Dose	Mid Dose	High Dose
Body [g] Weight (g)	Mean	424.8	428.4	412.8	430.6
	SD	20.04	17.90	48.14	76.17
	N	5	5	5	5
	%Diff	-	0.8	-2.8	1.4
Brain [g] (g)	Mean	2.0812	2.0652	2.0534	2.1064
	SD	0.08752	0.13752	0.06137	0.05044
	N	5	5	5	5
	%Diff	-	-0.8	-1.3	1.2
Brain/BWt [g] (%)	Mean	0.49010	0.48192	0.50267	0.49986
	SD	0.009551	0.020201	0.057696	0.078669
	N	5	5	5	5
	%Diff	-	-1.7	2.6	2.0
Adrenal [g] glands (g)	Mean	0.0794	0.0772	0.0678	0.0766
	SD	0.00733	0.01156	0.01033	0.01540
	N	5	5	5	5
	%Diff	-	-2.8	-14.6	-3.5
Adrenal gl/ [g] BWt (%)	Mean	0.01873	0.01802	0.01664	0.01777
	SD	0.001992	0.002575	0.003383	0.001492
	N	5	5	5	5
	%Diff	-	-3.8	-11.2	-5.1
Adrenal gl/ [g] BrWt (ratio)	Mean	0.03817	0.03746	0.03298	0.03631
	SD	0.003528	0.005664	0.004490	0.006847
	N	5	5	5	5
	%Diff	-	-1.9	-13.6	-4.9
Epididymides [g] (g)	Mean	1.4058	1.2644 a	1.2596 a	1.2984
	SD	0.08152	0.06512	0.07181	0.09127
	N	5	5	5	5
	%Diff	-	-10.1	-10.4	-7.6
Epididymides [g] /BWt (%)	Mean	0.33211	0.29561	0.30753	0.30680
	SD	0.033894	0.020357	0.029315	0.043296
	N	5	5	5	5
	%Diff	-	-11.0	-7.4	-7.6

[g] - Anova & Dunnett: a = $p < 0.05$

2759-001
A Single Dose Toxicity Study of Test Article Administered by Intrathecal Injection in Rats

Summary of Organ Weight Values
Day 29

Day: 29 Relative to Start Date

Sex: Male		Vehicle	Low Dose	Mid Dose	High Dose
Epididymides [g] /BrWt (ratio)	Mean	0.67702	0.61541	0.61377	0.61603
	SD	0.059205	0.064925	0.037939	0.032922
	N	5	5	5	5
	%Diff	-	-9.1	-9.3	-9.0
Heart [g] (g)	Mean	1.5930	1.6760	1.5262	1.8700
	SD	0.15541	0.17503	0.21268	0.30028
	N	5	5	5	5
	%Diff	-	5.2	-4.2	17.4
Heart/BWt [g] (%)	Mean	0.37563	0.39176	0.37003	0.43751
	SD	0.039439	0.042916	0.031163	0.054140
	N	5	5	5	5
	%Diff	-	4.3	-1.5	16.5
Heart/BrWt [g] (ratio)	Mean	0.76592	0.81423	0.74411	0.88602
	SD	0.072395	0.094042	0.111070	0.126166
	N	5	5	5	5
	%Diff	-	6.3	-2.8	15.7
Kidneys [g] (g)	Mean	3.6898	3.6490	3.4558	3.6642
	SD	0.27064	0.46621	0.54630	0.39657
	N	5	5	5	5
	%Diff	-	-1.1	-6.3	-0.7
Kidneys/BWt [g] (%)	Mean	0.87031	0.85062	0.83583	0.86075
	SD	0.079110	0.090002	0.063285	0.091558
	N	5	5	5	5
	%Diff	-	-2.3	-4.0	-1.1
Kidneys/BrWt [g] (ratio)	Mean	1.77467	1.76782	1.68648	1.73948
	SD	0.140931	0.199601	0.293295	0.181950
	N	5	5	5	5
	%Diff	-	-0.4	-5.0	-2.0
Liver [g] (g)	Mean	16.9814	15.8602	14.5980	15.5666
	SD	0.61408	1.80177	1.98181	3.49767
	N	5	5	5	5
	%Diff	-	-6.6	-14.0	-8.3

[g] - Anova & Dunnett

2759-001
A Single Dose Toxicity Study of Test Article Administered by Intrathecal Injection in Rats

Summary of Organ Weight Values
Day 29

Day: 29 Relative to Start Date

Sex: Male		Vehicle	Low Dose	Mid Dose	High Dose
Liver/BWt [g] (%)	Mean	4.00154	3.69754	3.53469 a	3.59563 a
	SD	0.157552	0.335785	0.183720	0.178573
	N	5	5	5	5
	%Diff	-	-7.6	-11.7	-10.1
Liver/BrWt [g] (ratio)	Mean	8.16498	7.69109	7.12001	7.38358
	SD	0.282185	0.847655	1.058682	1.580477
	N	5	5	5	5
	%Diff	-	-5.8	-12.8	-9.6
Lung [g] (g)	Mean	1.7852	1.6752	1.8804	2.1218
	SD	0.23102	0.18829	0.37795	0.34342
	N	5	5	5	5
	%Diff	-	-6.2	5.3	18.9
Lung/BWt [g] (%)	Mean	0.42113	0.39187	0.45251	0.50630
	SD	0.058512	0.050332	0.043541	0.135770
	N	5	5	5	5
	%Diff	-	-6.9	7.5	20.2
Lung/BrWt [g] (ratio)	Mean	0.85859	0.81172	0.91549	1.00580
	SD	0.111318	0.083884	0.179776	0.149872
	N	5	5	5	5
	%Diff	-	-5.5	6.6	17.1
Pituitary [g] (fixed) (g)	Mean	0.01826	0.01852	0.01648	0.01580
	SD	0.001313	0.003093	0.002359	0.001756
	N	5	5	5	5
	%Diff	-	1.4	-9.7	-13.5
Pituitary [g] (fixed)/BWt (%)	Mean	0.00431	0.00431	0.00400	0.00374
	SD	0.000418	0.000593	0.000400	0.000616
	N	5	5	5	5
	%Diff	-	0.0	-7.2	-13.2
Pituitary [g] (fixed)/BrWt (ratio)	Mean	0.00879	0.00892	0.00804	0.00751
	SD	0.000712	0.000915	0.001262	0.000927
	N	5	5	5	5
	%Diff	-	1.6	-8.5	-14.5

[g] - Anova & Dunnett: a = p < 0.05

2759-001
A Single Dose Toxicity Study of Test Article Administered by Intrathecal Injection in Rats

Summary of Organ Weight Values
Day 29

Day: 29 Relative to Start Date

Sex: Male		Vehicle	Low Dose	Mid Dose	High Dose
Prostate [g] gland (g)	Mean	1.1866	1.2762	1.2976	1.2570
	SD	0.19398	0.12599	0.24517	0.09633
	N	5	5	5	5
	%Diff	-	7.6	9.4	5.9
Prostate [g] gland/BWt (%)	Mean	0.28095	0.29907	0.31690	0.29816
	SD	0.056625	0.040740	0.070375	0.049038
	N	5	5	5	5
	%Diff	-	6.4	12.8	6.1
Prostate [g] gland/BrWt (ratio)	Mean	0.57228	0.62161	0.63415	0.59680
	SD	0.108277	0.089767	0.132262	0.044350
	N	5	5	5	5
	%Diff	-	8.6	10.8	4.3
Sal, mand/ [g] sub., rt (g)	Mean	0.3962	0.3690	0.3356	0.3556
	SD	0.03285	0.04515	0.02895	0.04449
	N	5	5	5	5
	%Diff	-	-6.9	-15.3	-10.2
Sal gl mand/ [g] sub., rt/BWt (%)	Mean	0.09367	0.08625	0.08172	0.08421
	SD	0.011774	0.011112	0.006413	0.014935
	N	5	5	5	5
	%Diff	-	-7.9	-12.8	-10.1
Sal gl mand/ [g] sub.,rt/BrWt (ratio)	Mean	0.19092	0.17857	0.16373	0.16891
	SD	0.021378	0.017637	0.017428	0.021617
	N	5	5	5	5
	%Diff	-	-6.5	-14.2	-11.5
Spleen [g1] (g)	Mean	0.8170	0.8076	1.0530	0.9064
	SD	0.07550	0.14213	0.27262	0.12972
	N	5	5	5	5
	%Diff	-	-1.2	28.9	10.9
Spleen/BWt [g] (%)	Mean	0.19293	0.18973	0.25449 a	0.21235
	SD	0.022513	0.041844	0.049945	0.024464
	N	5	5	5	5
	%Diff	-	-1.7	31.9	10.1

[g] - Anova & Dunnett: a = p < 0.05
[g1] - Anova & Dunnett(Log)

2759-001
A Single Dose Toxicity Study of Test Article Administered by Intrathecal Injection in Rats

Summary of Organ Weight Values
Day 29

Day: 29 Relative to Start Date

Sex: Male		Vehicle	Low Dose	Mid Dose	High Dose
Spleen/BrWt [g] (ratio)	Mean	0.39331	0.39357	0.51463	0.43004
	SD	0.041463	0.084214	0.143131	0.058209
	N	5	5	5	5
	%Diff	-	0.1	30.8	9.3
Testes [g1] (g)	Mean	3.6934	3.5542	3.6848	3.6902
	SD	0.34075	0.26375	0.26910	0.21554
	N	5	5	5	5
	%Diff	-	-3.8	-0.2	-0.1
Testes/BWt [g1] (%)	Mean	0.87094	0.82942	0.90098	0.87158
	SD	0.089134	0.046405	0.110961	0.108891
	N	5	5	5	5
	%Diff	-	-4.8	3.4	0.1
Testes/BrWt [g1] (ratio)	Mean	1.77498	1.72565	1.79681	1.75339
	SD	0.152396	0.154120	0.155997	0.122632
	N	5	5	5	5
	%Diff	-	-2.8	1.2	-1.2
Thymus [g1] (g)	Mean	0.4624	0.5054	0.5980	0.5778
	SD	0.11724	0.09868	0.19374	0.09141
	N	5	5	5	5
	%Diff	-	9.3	29.3	25.0
Thymus/BWt [g1] (%)	Mean	0.10857	0.11840	0.14312	0.13633
	SD	0.024950	0.025174	0.033642	0.026082
	N	5	5	5	5
	%Diff	-	9.1	31.8	25.6
Thymus/BrWt (ratio)	Mean	0.22153	0.24596	0.29108	0.27427
	SD	0.050453	0.053612	0.091575	0.042494
	N	5	5	5	5
	%Diff	-	11.0	31.4	23.8
Thyroid/para [g1] (fixed) (g)	Mean	0.03464	0.03058	0.02686 b	0.02692 b
	SD	0.002601	0.002794	0.003652	0.003552
	N	5	5	5	5
	%Diff	-	-11.7	-22.5	-22.3

[g] - Anova & Dunnett(Log)
[g1] - Anova & Dunnett: b = $p < 0.01$

2759-001
A Single Dose Toxicity Study of Test Article Administered by Intrathecal Injection in Rats

Summary of Organ Weight Values
Day 29

Day: 29 Relative to Start Date

Sex: Male		Vehicle	Low Dose	Mid Dose	High Dose
Thyroid/para [g] (fixed)/BWt (%)	Mean	0.00818	0.00714	0.00654 b	0.00629 b
	SD	0.000884	0.000587	0.000959	0.000377
	N	5	5	5	5
	%Diff	-	-12.7	-20.0	-23.1
Thyroid/para [g] (fixed)/BrWt (ratio)	Mean	0.01669	0.01481	0.01307 b	0.01279 b
	SD	0.001804	0.000898	0.001644	0.001671
	N	5	5	5	5
	%Diff	-	-11.3	-21.7	-23.4

[g] - Anova & Dunnett: b = p < 0.01

2759-001
A Single Dose Toxicity Study of Test Article Administered by Intrathecal Injection in Rats

Summary of Organ Weight Values
Day 29

Day: 29 Relative to Start Date

Sex: Female		Vehicle	Low Dose	Mid Dose	High Dose
Body [g] Weight (g)	Mean	239.0	215.4	217.0	222.0
	SD	16.45	16.77	23.47	24.81
	N	5	5	5	5
	%Diff	-	-9.9	-9.2	-7.1
Brain [g] (g)	Mean	1.9694	1.8742	1.8726	1.9056
	SD	0.09438	0.06797	0.12063	0.06379
	N	5	5	5	5
	%Diff	-	-4.8	-4.9	-3.2
Brain/BWt [g] (%)	Mean	0.82597	0.87276	0.86659	0.86708
	SD	0.047198	0.047690	0.045652	0.102031
	N	5	5	5	5
	%Diff	-	5.7	4.9	5.0
Adrenal [g] glands (g)	Mean	0.0744	0.0700	0.0676	0.0916
	SD	0.01178	0.01373	0.01041	0.02130
	N	5	5	5	5
	%Diff	-	-5.9	-9.1	23.1
Adrenal gl/ [g] BWt (%)	Mean	0.03133	0.03277	0.03143	0.04101
	SD	0.005776	0.007451	0.005532	0.006780
	N	5	5	5	5
	%Diff	-	4.6	0.3	30.9
Adrenal gl/ [g] BrWt (ratio)	Mean	0.03802	0.03755	0.03621	0.04803
	SD	0.007321	0.008509	0.005920	0.010668
	N	5	5	5	5
	%Diff	-	-1.2	-4.7	26.3
Heart [g] (g)	Mean	0.9096	0.8448	0.8920	1.0042
	SD	0.10339	0.07755	0.10544	0.14268
	N	5	5	5	5
	%Diff	-	-7.1	-1.9	10.4
Heart/BWt [g] (%)	Mean	0.38398	0.39333	0.41086	0.45191
	SD	0.067911	0.037789	0.013184	0.033544
	N	5	5	5	5
	%Diff	-	2.4	7.0	17.7

[g] - Anova & Dunnett

2759-001
A Single Dose Toxicity Study of Test Article Administered by Intrathecal Injection in Rats

Summary of Organ Weight Values
Day 29

Day: 29 Relative to Start Date

Sex: Female		Vehicle	Low Dose	Mid Dose	High Dose
Heart/BrWt [g] (ratio)	Mean	0.46330	0.45101	0.47521	0.52667
	SD	0.063675	0.042176	0.030470	0.068543
	N	5	5	5	5
	%Diff	-	-2.7	2.6	13.7
Kidneys [g] (g)	Mean	1.8350	1.8626	1.8092	1.9058
	SD	0.12585	0.15893	0.27377	0.20671
	N	5	5	5	5
	%Diff	-	1.5	-1.4	3.9
Kidneys/BWt [g] (%)	Mean	0.76920	0.86549 a	0.83080	0.85929 a
	SD	0.052960	0.054644	0.046818	0.033846
	N	5	5	5	5
	%Diff	-	12.5	8.0	11.7
Kidneys/BrWt [g] (ratio)	Mean	0.93253	0.99419	0.96285	1.00043
	SD	0.064350	0.084530	0.100455	0.106865
	N	5	5	5	5
	%Diff	-	6.6	3.3	7.3
Liver [g1] (g)	Mean	8.4688	8.3660	8.3644	8.4880
	SD	0.52046	0.71902	1.22966	1.64545
	N	5	5	5	5
	%Diff	-	-1.2	-1.2	0.2
Liver/BWt [g] (%)	Mean	3.55444	3.89165	3.84988	3.79882
	SD	0.294742	0.321773	0.318121	0.383506
	N	5	5	5	5
	%Diff	-	9.5	8.3	6.9
Liver/BrWt [g] (ratio)	Mean	4.30228	4.46600	4.45678	4.45777
	SD	0.228742	0.392010	0.494012	0.877861
	N	5	5	5	5
	%Diff	-	3.8	3.6	3.6
Lung [g2] (g)	Mean	1.4840	1.2412	1.2112	1.2232
	SD	0.35286	0.10872	0.19733	0.06566
	N	5	5	5	5
	%Diff	-	-16.4	-18.4	-17.6

[g] - Anova & Dunnett: a = p < 0.05
[g1] - Kruskal-Wallis & Dunn
[g2] - Anova & Dunnett(Log)

2759-001
A Single Dose Toxicity Study of Test Article Administered by Intrathecal Injection in Rats

Summary of Organ Weight Values
Day 29

Day: 29 Relative to Start Date

Sex: Female		Vehicle	Low Dose	Mid Dose	High Dose
Lung/BWt [g] (%)	Mean	0.61849	0.57610	0.56105	0.55435
	SD	0.123603	0.016295	0.096499	0.040592
	N	5	5	5	5
	%Diff	-	-6.9	-9.3	-10.4
Lung/BrWt [g1] (ratio)	Mean	0.75038	0.66179	0.64715	0.64227
	SD	0.154013	0.043332	0.100871	0.036181
	N	5	5	5	5
	%Diff	-	-11.8	-13.8	-14.4
Ovaries [g] (g)	Mean	0.0844	0.0790	0.0692	0.0918
	SD	0.01788	0.01602	0.02195	0.03667
	N	5	5	5	5
	%Diff	-	-6.4	-18.0	8.8
Ovaries/BWt [g] (%)	Mean	0.03575	0.03706	0.03162	0.04123
	SD	0.009406	0.008541	0.007832	0.014895
	N	5	5	5	5
	%Diff	-	3.7	-11.6	15.3
Ovaries/BrWt [g] (ratio)	Mean	0.04296	0.04239	0.03667	0.04797
	SD	0.009659	0.009612	0.009917	0.018926
	N	5	5	5	5
	%Diff	-	-1.3	-14.6	11.7
Oviducts [g2] (g)	Mean	0.0258	0.0276	0.0274	0.0284
	SD	0.01314	0.00456	0.00508	0.00594
	N	5	5	5	5
	%Diff	-	7.0	6.2	10.1
Oviducts/BWt (%)	Mean	0.01098	0.01295	0.01271	0.01269
	SD	0.006226	0.002694	0.002542	0.001489
	N	5	5	5	5
	%Diff	-	17.9	15.8	15.6
Oviducts/ [g2] BrWt (ratio)	Mean	0.01319	0.01481	0.01467	0.01493
	SD	0.007044	0.002884	0.002813	0.003249
	N	5	5	5	5
	%Diff	-	12.2	11.2	13.2

[g] - Anova & Dunnett
[g1] - Kruskal-Wallis & Dunn
[g2] - Anova & Dunnett(Log)

2759-001
A Single Dose Toxicity Study of Test Article Administered by Intrathecal Injection in Rats

Summary of Organ Weight Values
Day 29

Day: 29 Relative to Start Date

Sex: Female		Vehicle	Low Dose	Mid Dose	High Dose
Pituitary [g] (fixed) (g)	Mean	0.01828	0.01998	0.01636	0.01772
	SD	0.002025	0.001506	0.003709	0.003458
	N	5	5	5	5
	%Diff	-	9.3	-10.5	-3.1
Pituitary [g] (fixed)/BWt (%)	Mean	0.00772	0.00932	0.00763	0.00795
	SD	0.001337	0.001016	0.002140	0.000971
	N	5	5	5	5
	%Diff	-	20.8	-1.1	3.0
Pituitary [g] (fixed)/BrWt (ratio)	Mean	0.00931	0.01067	0.00876	0.00928
	SD	0.001221	0.000903	0.002096	0.001643
	N	5	5	5	5
	%Diff	-	14.7	-5.9	-0.3
Sal, mand/ [g] sub., rt (g)	Mean	0.2278	0.2040	0.1728	0.1922
	SD	0.05139	0.03090	0.05213	0.06563
	N	5	5	5	5
	%Diff	-	-10.4	-24.1	-15.6
Sal gl mand/ [g] sub., rt/BWt (%)	Mean	0.09526	0.09554	0.08060	0.08531
	SD	0.019489	0.018216	0.024590	0.026535
	N	5	5	5	5
	%Diff	-	0.3	-15.4	-10.5
Sal gl mand/ [g] sub.,rt/BrWt (ratio)	Mean	0.11588	0.10937	0.09307	0.10100
	SD	0.027197	0.020035	0.029131	0.034642
	N	5	5	5	5
	%Diff	-	-5.6	-19.7	-12.8
Spleen [g] (g)	Mean	0.5366	0.5058	0.5566	0.6330
	SD	0.07379	0.05054	0.15856	0.08507
	N	5	5	5	5
	%Diff	-	-5.7	3.7	18.0
Spleen/BWt [g] (%)	Mean	0.22589	0.23585	0.25354	0.28583
	SD	0.037756	0.027947	0.052836	0.029633
	N	5	5	5	5
	%Diff	-	4.4	12.2	26.5

[g] - Anova & Dunnett

2759-001
A Single Dose Toxicity Study of Test Article Administered by Intrathecal Injection in Rats

Summary of Organ Weight Values
Day 29

Day: 29 Relative to Start Date

Sex: Female		Vehicle	Low Dose	Mid Dose	High Dose
Spleen/BrWt [g] (ratio)	Mean	0.27355	0.27058	0.29524	0.33191
	SD	0.044595	0.033330	0.074004	0.040895
	N	5	5	5	5
	%Diff	-	-1.1	7.9	21.3
Thymus [g] (g)	Mean	0.4512	0.3686	0.3476	0.4470
	SD	0.08675	0.07555	0.09032	0.18239
	N	5	5	5	5
	%Diff	-	-18.3	-23.0	-0.9
Thymus/BWt [g] (%)	Mean	0.18792	0.17170	0.16056	0.19801
	SD	0.029214	0.037128	0.037330	0.062958
	N	5	5	5	5
	%Diff	-	-8.6	-14.6	5.4
Thymus/BrWt [g] (ratio)	Mean	0.22906	0.19683	0.18590	0.23545
	SD	0.043509	0.040869	0.047051	0.097880
	N	5	5	5	5
	%Diff	-	-14.1	-18.8	2.8
Thyroid/para [g] (fixed) (g)	Mean	0.02030	0.02036	0.02124	0.01968
	SD	0.003706	0.002610	0.002028	0.002149
	N	5	5	5	5
	%Diff	-	0.3	4.6	-3.1
Thyroid/para [g] (fixed)/BWt (%)	Mean	0.00848	0.00956	0.00990	0.00887
	SD	0.001273	0.001805	0.001551	0.000231
	N	5	5	5	5
	%Diff	-	12.8	16.8	4.6
Thyroid/para [g] (fixed)/BrWt (ratio)	Mean	0.01029	0.01091	0.01138	0.01033
	SD	0.001663	0.001719	0.001346	0.001146
	N	5	5	5	5
	%Diff	-	6.1	10.7	0.5
Uterus w/ [g] cervix (g)	Mean	0.5414	0.5708	0.5760	0.5394
	SD	0.09542	0.09218	0.16120	0.06286
	N	5	5	5	5
	%Diff	-	5.4	6.4	-0.4

[g] - Anova & Dunnett

2759-001
A Single Dose Toxicity Study of Test Article Administered by Intrathecal Injection in Rats

Summary of Organ Weight Values
Day 29

Day: 29 Relative to Start Date

Sex: Female		Vehicle	Low Dose	Mid Dose	High Dose
Uterus w/ [g] cervix/BWt (%)	Mean	0.22805	0.26549	0.26999	0.24475
	SD	0.047512	0.042177	0.095123	0.034244
	N	5	5	5	5
	%Diff	-	16.4	18.4	7.3
Uterus w/ [g] cervix/BrWt (ratio)	Mean	0.27613	0.30467	0.31008	0.28298
	SD	0.055558	0.047706	0.099569	0.030952
	N	5	5	5	5
	%Diff	-	10.3	12.3	2.5

[g] - Anova & Dunnett

Table 6
Summary of Organ Weight Values (Day 91)

2759-001
A Single Dose Toxicity Study of Test Article Administered by Intrathecal Injection in Rats

Summary of Organ Weight Values
Day 91

Day: 91 Relative to Start Date

Sex: Male		Vehicle	Low Dose	Mid Dose	High Dose
Body [g] Weight (g)	Mean	508.2	500.4	551.0	534.0
	SD	20.45	55.76	50.14	132.37
	N	5	5	5	5
	%Diff	-	-1.5	8.4	5.1
Brain [g] (g)	Mean	2.1732	2.1428	2.1038	2.1218
	SD	0.12996	0.08089	0.05134	0.06196
	N	5	5	5	5
	%Diff	-	-1.4	-3.2	-2.4
Brain/BWt [g1] (%)	Mean	0.42858	0.43242	0.38477	0.42392
	SD	0.036599	0.049288	0.041431	0.138586
	N	5	5	5	5
	%Diff	-	0.9	-10.2	-1.1
Adrenal [g] glands (g)	Mean	0.0684	0.0644	0.0704	0.0682
	SD	0.01609	0.01521	0.01592	0.01165
	N	5	5	5	5
	%Diff	-	-5.8	2.9	-0.3
Adrenal gl/ [g] BWt (%)	Mean	0.01343	0.01275	0.01304	0.01322
	SD	0.002919	0.001706	0.004107	0.002814
	N	5	5	5	5
	%Diff	-	-5.1	-2.9	-1.5
Adrenal gl/ [g] BrWt (ratio)	Mean	0.03192	0.03011	0.03336	0.03220
	SD	0.009501	0.007471	0.006840	0.005873
	N	5	5	5	5
	%Diff	-	-5.7	4.5	0.9
Epididymides [g] (g)	Mean	1.5482	1.5656	1.5358	1.4766
	SD	0.16960	0.05978	0.08808	0.08587
	N	5	5	5	5
	%Diff	-	1.1	-0.8	-4.6
Epididymides [g] /BWt (%)	Mean	0.30483	0.31568	0.28096	0.29061
	SD	0.032541	0.033072	0.033704	0.071974
	N	5	5	5	5
	%Diff	-	3.6	-7.8	-4.7

[g] - Anova & Dunnett
[g1] - Anova & Dunnett(Log)

2759-001
A Single Dose Toxicity Study of Test Article Administered by Intrathecal Injection in Rats

Summary of Organ Weight Values
Day 91

Day: 91 Relative to Start Date

Sex: Male		Vehicle	Low Dose	Mid Dose	High Dose
Epididymides [g] /BrWt (ratio)	Mean	0.71776	0.73114	0.72995	0.69699
	SD	0.120357	0.030310	0.035999	0.055127
	N	5	5	5	5
	%Diff	-	1.9	1.7	-2.9
Heart [g] (g)	Mean	1.6768	1.8614	1.9036	2.0814
	SD	0.13245	0.25011	0.22000	0.33516
	N	5	5	5	5
	%Diff	-	11.0	13.5	24.1
Heart/BWt [g] (%)	Mean	0.33035	0.37288	0.34617	0.40038
	SD	0.029647	0.040620	0.033627	0.062450
	N	5	5	5	5
	%Diff	-	12.9	4.8	21.2
Heart/BrWt [g] (ratio)	Mean	0.77573	0.87012	0.90694	0.98185
	SD	0.098348	0.127309	0.123553	0.161885
	N	5	5	5	5
	%Diff	-	12.2	16.9	26.6
Kidneys [g] (g)	Mean	3.8454	3.6118	4.0230	3.8416
	SD	0.13649	0.51406	0.62132	0.40699
	N	5	5	5	5
	%Diff	-	-6.1	4.6	-0.1
Kidneys/BWt [g1] (%)	Mean	0.75716	0.72011	0.73159	0.75886
	SD	0.026784	0.033184	0.097832	0.227445
	N	5	5	5	5
	%Diff	-	-4.9	-3.4	0.2
Kidneys/BrWt [g] (ratio)	Mean	1.77363	1.68631	1.91387	1.80877
	SD	0.106193	0.242608	0.305429	0.165194
	N	5	5	5	5
	%Diff	-	-4.9	7.9	2.0
Liver [g] (g)	Mean	15.2390	15.3124	17.1628	17.5508
	SD	0.44115	2.87372	1.57775	2.31615
	N	5	5	5	5
	%Diff	-	0.5	12.6	15.2

[g] - Anova & Dunnett
[g1] - Kruskal-Wallis & Dunn

2759-001
A Single Dose Toxicity Study of Test Article Administered by Intrathecal Injection in Rats

Summary of Organ Weight Values
Day 91

Day: 91 Relative to Start Date

Sex: Male		Vehicle	Low Dose	Mid Dose	High Dose
Liver/BWt [g] (%)	Mean	2.99991	3.04019	3.12305	3.44683
	SD	0.050338	0.236575	0.251397	0.968213
	N	5	5	5	5
	%Diff	-	1.3	4.1	14.9
Liver/BrWt [g1] (ratio)	Mean	7.04028	7.16064	8.16865	8.26825
	SD	0.605413	1.429703	0.853643	1.058828
	N	5	5	5	5
	%Diff	-	1.7	16.0	17.4
Lung [g2] (g)	Mean	1.8658	2.1060	2.2704	2.0682
	SD	0.12817	0.27309	0.53860	0.39574
	N	5	5	5	5
	%Diff	-	12.9	21.7	10.8
Lung/BWt [g1] (%)	Mean	0.36752	0.42116	0.41067	0.40595
	SD	0.027236	0.034994	0.074719	0.111184
	N	5	5	5	5
	%Diff	-	14.6	11.7	10.5
Lung/BrWt [g2] (ratio)	Mean	0.86108	0.98337	1.08292	0.97656
	SD	0.079163	0.131459	0.277082	0.194776
	N	5	5	5	5
	%Diff	-	14.2	25.8	13.4
Pituitary [g1] (fixed) (g)	Mean	0.01802	0.01654	0.01784	0.01622
	SD	0.001134	0.002339	0.001481	0.002139
	N	5	5	5	5
	%Diff	-	-8.2	-1.0	-10.0
Pituitary [g] (fixed)/BWt (%)	Mean	0.00355	0.00330	0.00325	0.00327
	SD	0.000319	0.000299	0.000228	0.001279
	N	5	5	5	5
	%Diff	-	-7.1	-8.7	-8.0
Pituitary [g1] (fixed)/BrWt (ratio)	Mean	0.00830	0.00772	0.00849	0.00763
	SD	0.000451	0.001115	0.000829	0.000813
	N	5	5	5	5
	%Diff	-	-7.0	2.3	-8.1

[g] - Kruskal-Wallis & Dunn
[g1] - Anova & Dunnett
[g2] - Anova & Dunnett(Log)

2759-001
A Single Dose Toxicity Study of Test Article Administered by Intrathecal Injection in Rats

Summary of Organ Weight Values
Day 91

Day: 91 Relative to Start Date

Sex: Male		Vehicle	Low Dose	Mid Dose	High Dose
Prostate [g] gland (g)	Mean	1.6784	1.3944	1.5052	1.5304
	SD	0.23836	0.20146	0.30886	0.28067
	N	5	5	5	5
	%Diff	-	-16.9	-10.3	-8.8
Prostate [g] gland/BWt (%)	Mean	0.33187	0.28077	0.27567	0.29537
	SD	0.057681	0.046175	0.064562	0.057875
	N	5	5	5	5
	%Diff	-	-15.4	-16.9	-11.0
Prostate [g] gland/BrWt (ratio)	Mean	0.77251	0.64885	0.71391	0.72198
	SD	0.098936	0.072419	0.133755	0.135186
	N	5	5	5	5
	%Diff	-	-16.0	-7.6	-6.5
Sal, mand/ [g] sub., rt (g)	Mean	0.3566	0.3576	0.4096	0.3880
	SD	0.01936	0.02903	0.08325	0.07150
	N	5	5	5	5
	%Diff	-	0.3	14.9	8.8
Sal gl mand/ [g1] sub., rt/BWt (%)	Mean	0.07031	0.07189	0.07543	0.07934
	SD	0.005688	0.006951	0.018717	0.039000
	N	5	5	5	5
	%Diff	-	2.2	7.3	12.8
Sal gl mand/ [g] sub.,rt/BrWt (ratio)	Mean	0.16480	0.16699	0.19445	0.18239
	SD	0.016507	0.014067	0.038246	0.029834
	N	5	5	5	5
	%Diff	-	1.3	18.0	10.7
Spleen [g] (g)	Mean	0.8580	0.8326	0.8836	1.0106
	SD	0.15733	0.07911	0.07597	0.05000
	N	5	5	5	5
	%Diff	-	-3.0	3.0	17.8
Spleen/BWt [g1] (%)	Mean	0.16895	0.16676	0.16173	0.20216
	SD	0.030605	0.008467	0.023922	0.068593
	N	5	5	5	5
	%Diff	-	-1.3	-4.3	19.7

[g] - Anova & Dunnett
[g1] - Anova & Dunnett(Log)

2759-001
A Single Dose Toxicity Study of Test Article Administered by Intrathecal Injection in Rats

Summary of Organ Weight Values
Day 91

Day: 91 Relative to Start Date

Sex: Male		Vehicle	Low Dose	Mid Dose	High Dose
Spleen/BrWt [g] (ratio)	Mean	0.39739	0.38912	0.41961	0.47609
	SD	0.084486	0.041297	0.028138	0.011588
	N	5	5	5	5
	%Diff	-	-2.1	5.6	19.8
Testes [g] (g)	Mean	3.6970	3.6838	3.6718	3.4820
	SD	0.04958	0.08258	0.30679	0.24092
	N	5	5	5	5
	%Diff	-	-0.4	-0.7	-5.8
Testes/BWt [g1] (%)	Mean	0.72862	0.74394	0.66849	0.69060
	SD	0.036452	0.089026	0.051406	0.202527
	N	5	5	5	5
	%Diff	-	2.1	-8.3	-5.2
Testes/BrWt [g] (ratio)	Mean	1.70526	1.72110	1.74672	1.64307
	SD	0.086322	0.075038	0.159070	0.136964
	N	5	5	5	5
	%Diff	-	0.9	2.4	-3.6
Thymus [g] (g)	Mean	0.2446	0.2764	0.3592	0.4026 a
	SD	0.05111	0.08809	0.06843	0.11032
	N	5	5	5	5
	%Diff	-	13.0	46.9	64.6
Thymus/BWt [g] (%)	Mean	0.04827	0.05532	0.06576	0.07531 a
	SD	0.010682	0.018076	0.014221	0.006341
	N	5	5	5	5
	%Diff	-	14.6	36.2	56.0
Thymus/BrWt [g] (ratio)	Mean	0.11193	0.13002	0.17097	0.19041 a
	SD	0.018511	0.044300	0.034049	0.054243
	N	5	5	5	5
	%Diff	-	16.2	52.7	70.1
Thyroid/para [g] (fixed) (g)	Mean	0.02832	0.02620	0.03274	0.03060
	SD	0.007585	0.006719	0.005847	0.004552
	N	5	5	5	5
	%Diff	-	-7.5	15.6	8.1

[g] - Anova & Dunnett: a = p < 0.05
[g1] - Anova & Dunnett(Log)

2759-001
A Single Dose Toxicity Study of Test Article Administered by Intrathecal Injection in Rats

Summary of Organ Weight Values
Day 91

Day: 91 Relative to Start Date

Sex: Male		Vehicle	Low Dose	Mid Dose	High Dose
Thyroid/para [g] (fixed)/BWt (%)	Mean	0.00558	0.00519	0.00596	0.00610
	SD	0.001479	0.000888	0.001030	0.002108
	N	5	5	5	5
	%Diff	-	-6.9	6.8	9.4
Thyroid/para [g] (fixed)/BrWt (ratio)	Mean	0.01305	0.01226	0.01555	0.01440
	SD	0.003425	0.003376	0.002642	0.001872
	N	5	5	5	5
	%Diff	-	-6.0	19.2	10.4

[g] - Anova & Dunnett

2759-001
A Single Dose Toxicity Study of Test Article Administered by Intrathecal Injection in Rats

Summary of Organ Weight Values
Day 91

Day: 91 Relative to Start Date

Sex: Female		Vehicle	Low Dose	Mid Dose	High Dose
Body [g] Weight (g)	Mean	279.4	269.4	268.0	301.6
	SD	24.36	20.34	16.93	22.55
	N	5	5	5	5
	%Diff	-	-3.6	-4.1	7.9
Brain [g1] (g)	Mean	1.9340	1.9702	1.9778	1.9656
	SD	0.07522	0.11679	0.08732	0.12458
	N	5	5	5	5
	%Diff	-	1.9	2.3	1.6
Brain/BWt [g] (%)	Mean	0.69638	0.73385	0.74064	0.65500
	SD	0.064947	0.056481	0.059909	0.067556
	N	5	5	5	5
	%Diff	-	5.4	6.4	-5.9
Adrenal [g] glands (g)	Mean	0.0692	0.0686	0.0892	0.0866
	SD	0.00471	0.02314	0.01069	0.01718
	N	5	5	5	5
	%Diff	-	-0.9	28.9	25.1
Adrenal gl/ [g] BWt (%)	Mean	0.02484	0.02549	0.03330	0.02887
	SD	0.001450	0.008270	0.003612	0.006116
	N	5	5	5	5
	%Diff	-	2.6	34.1	16.3
Adrenal gl/ [g1] BrWt (ratio)	Mean	0.03578	0.03531	0.04507	0.04417
	SD	0.002024	0.013299	0.004585	0.009263
	N	5	5	5	5
	%Diff	-	-1.3	26.0	23.5
Heart [g] (g)	Mean	1.1088	1.0782	1.2174	1.2134
	SD	0.14393	0.17517	0.15510	0.14135
	N	5	5	5	5
	%Diff	-	-2.8	9.8	9.4
Heart/BWt [g] (%)	Mean	0.39558	0.39860	0.45386	0.40209
	SD	0.019795	0.041390	0.046784	0.033933
	N	5	5	5	5
	%Diff	-	0.8	14.7	1.6

[g] - Anova & Dunnett
[g1] - Kruskal-Wallis & Dunn

2759-001
A Single Dose Toxicity Study of Test Article Administered by Intrathecal Injection in Rats

Summary of Organ Weight Values
Day 91

Day: 91 Relative to Start Date

Sex: Female		Vehicle	Low Dose	Mid Dose	High Dose
Heart/BrWt [g] (ratio)	Mean	0.57314	0.54834	0.61596	0.61857
	SD	0.070273	0.094040	0.077994	0.076488
	N	5	5	5	5
	%Diff	-	-4.3	7.5	7.9
Kidneys [g] (g)	Mean	2.0970	2.0984	2.2794	2.2132
	SD	0.20841	0.20257	0.18649	0.16172
	N	5	5	5	5
	%Diff	-	0.1	8.7	5.5
Kidneys/BWt [g] (%)	Mean	0.75043	0.77912	0.85187 a	0.73477
	SD	0.031474	0.048731	0.070786	0.041229
	N	5	5	5	5
	%Diff	-	3.8	13.5	-2.1
Kidneys/BrWt [g] (ratio)	Mean	1.08369	1.06542	1.15272	1.12883
	SD	0.092010	0.087166	0.083214	0.101166
	N	5	5	5	5
	%Diff	-	-1.7	6.4	4.2
Liver [g] (g)	Mean	9.6278	9.0286	9.1702	10.2726
	SD	1.38025	1.05431	0.68846	1.42472
	N	5	5	5	5
	%Diff	-	-6.2	-4.8	6.7
Liver/BWt [g] (%)	Mean	3.45445	3.34374	3.42146	3.40889
	SD	0.454562	0.164389	0.135833	0.398309
	N	5	5	5	5
	%Diff	-	-3.2	-1.0	-1.3
Liver/BrWt [g] (ratio)	Mean	4.96843	4.58494	4.64585	5.24076
	SD	0.586933	0.503257	0.441351	0.801606
	N	5	5	5	5
	%Diff	-	-7.7	-6.5	5.5
Lung [g] (g)	Mean	1.4068	1.3680	1.4428	1.4982
	SD	0.14535	0.17428	0.11743	0.17678
	N	5	5	5	5
	%Diff	-	-2.8	2.6	6.5

[g] - Anova & Dunnett: a = p < 0.05

2759-001
A Single Dose Toxicity Study of Test Article Administered by Intrathecal Injection in Rats

Summary of Organ Weight Values
Day 91

Day: 91 Relative to Start Date

Sex: Female		Vehicle	Low Dose	Mid Dose	High Dose
Lung/BWt [g] (%)	Mean	0.50421	0.50921	0.54032	0.50110
	SD	0.038092	0.067727	0.057271	0.087730
	N	5	5	5	5
	%Diff	-	1.0	7.2	-0.6
Lung/BrWt [g] (ratio)	Mean	0.72748	0.69479	0.72876	0.76582
	SD	0.071245	0.083475	0.033356	0.110981
	N	5	5	5	5
	%Diff	-	-4.5	0.2	5.3
Ovaries [g] (g)	Mean	0.0868	0.0770	0.0896	0.1008
	SD	0.03065	0.01483	0.01778	0.00363
	N	5	5	5	5
	%Diff	-	-11.3	3.2	16.1
Ovaries/BWt [g] (%)	Mean	0.03114	0.02864	0.03372	0.03360
	SD	0.010404	0.005201	0.007554	0.003069
	N	5	5	5	5
	%Diff	-	-8.0	8.3	7.9
Ovaries/BrWt [g] (ratio)	Mean	0.04482	0.03888	0.04508	0.05144
	SD	0.015287	0.005157	0.007282	0.003585
	N	5	5	5	5
	%Diff	-	-13.2	0.6	14.8
Oviducts [g] (g)	Mean	0.0302	0.0238	0.0320	0.0334
	SD	0.01038	0.00955	0.00529	0.01014
	N	5	5	5	5
	%Diff	-	-21.2	6.0	10.6
Oviducts/BWt [g] (%)	Mean	0.01073	0.00907	0.01203	0.01126
	SD	0.003253	0.004469	0.002459	0.003903
	N	5	5	5	5
	%Diff	-	-15.4	12.2	5.0
Oviducts/ [g] BrWt (ratio)	Mean	0.01571	0.01223	0.01618	0.01698
	SD	0.005609	0.005330	0.002600	0.005156
	N	5	5	5	5
	%Diff	-	-22.2	3.0	8.0

[g] - Anova & Dunnett

2759-001
A Single Dose Toxicity Study of Test Article Administered by Intrathecal Injection in Rats

Summary of Organ Weight Values
Day 91

Day: 91 Relative to Start Date

Sex: Female		Vehicle	Low Dose	Mid Dose	High Dose
Pituitary [g] (fixed) (g)	Mean	0.02224	0.02104	0.02556	0.02484
	SD	0.004459	0.005414	0.004433	0.001982
	N	5	5	5	5
	%Diff	-	-5.4	14.9	11.7
Pituitary [g] (fixed)/BWt (%)	Mean	0.00794	0.00786	0.00951	0.00827
	SD	0.001264	0.002143	0.001198	0.000853
	N	5	5	5	5
	%Diff	-	-1.0	19.7	4.1
Pituitary [g] (fixed)/BrWt (ratio)	Mean	0.01148	0.01072	0.01301	0.01269
	SD	0.002128	0.002893	0.002773	0.001447
	N	5	5	5	5
	%Diff	-	-6.6	13.3	10.6
Sal, mand/ [g] sub., rt (g)	Mean	0.2134	0.2100	0.2288	0.2338
	SD	0.02142	0.03040	0.03050	0.04503
	N	5	5	5	5
	%Diff	-	-1.6	7.2	9.6
Sal gl mand/ [g] sub., rt/BWt (%)	Mean	0.07655	0.07789	0.08567	0.07799
	SD	0.006669	0.008621	0.013178	0.017359
	N	5	5	5	5
	%Diff	-	1.8	11.9	1.9
Sal gl mand/ [g] sub.,rt/BrWt (ratio)	Mean	0.11017	0.10649	0.11588	0.11988
	SD	0.007415	0.013104	0.016458	0.026555
	N	5	5	5	5
	%Diff	-	-3.3	5.2	8.8
Spleen [g] (g)	Mean	0.5186	0.5074	0.5834	0.6432 a
	SD	0.05136	0.08911	0.07097	0.08159
	N	5	5	5	5
	%Diff	-	-2.2	12.5	24.0
Spleen/BWt [g] (%)	Mean	0.18600	0.18823	0.21860	0.21308
	SD	0.015170	0.029146	0.031303	0.020803
	N	5	5	5	5
	%Diff	-	1.2	17.5	14.6

[g] - Anova & Dunnett: a = $p < 0.05$

2759-001
A Single Dose Toxicity Study of Test Article Administered by Intrathecal Injection in Rats

Summary of Organ Weight Values
Day 91

Day: 91 Relative to Start Date

Sex: Female		Vehicle	Low Dose	Mid Dose	High Dose
Spleen/BrWt [g] (ratio)	Mean	0.26797	0.25652	0.29435	0.32886
	SD	0.022367	0.033563	0.025377	0.051517
	N	5	5	5	5
	%Diff	-	-4.3	9.8	22.7
Thymus [g1] (g)	Mean	0.3020	0.2810	0.2938	0.3246
	SD	0.06525	0.06778	0.02879	0.09255
	N	5	5	5	5
	%Diff	-	-7.0	-2.7	7.5
Thymus/BWt [g1] (%)	Mean	0.10784	0.10350	0.10954	0.10646
	SD	0.019946	0.019507	0.006775	0.024071
	N	5	5	5	5
	%Diff	-	-4.0	1.6	-1.3
Thymus/BrWt (ratio)	Mean	0.15658	0.14302	0.14888	0.16651
	SD	0.036496	0.036170	0.017614	0.052524
	N	5	5	5	5
	%Diff	-	-8.7	-4.9	6.3
Thyroid/para [g1] (fixed) (g)	Mean	0.02200	0.02138	0.02096	0.02656
	SD	0.004147	0.002491	0.002082	0.003196
	N	5	5	5	5
	%Diff	-	-2.8	-4.7	20.7
Thyroid/para [g1] (fixed)/BWt (%)	Mean	0.00793	0.00793	0.00784	0.00882
	SD	0.001663	0.000594	0.000831	0.000952
	N	5	5	5	5
	%Diff	-	0.0	-1.2	11.2
Thyroid/para [g1] (fixed)/BrWt (ratio)	Mean	0.01134	0.01088	0.01058	0.01360
	SD	0.001771	0.001432	0.000685	0.002204
	N	5	5	5	5
	%Diff	-	-4.1	-6.7	19.9
Uterus w/ [g] cervix (g)	Mean	0.5562	0.6014	0.8336	0.6446
	SD	0.05261	0.16852	0.29609	0.08454
	N	5	5	5	5
	%Diff	-	8.1	49.9	15.9

[g] - Anova & Dunnett(Log)
[g1] - Anova & Dunnett

2759-001
A Single Dose Toxicity Study of Test Article Administered by Intrathecal Injection in Rats

Summary of Organ Weight Values
Day 91

Day: 91 Relative to Start Date

Sex: Female		Vehicle	Low Dose	Mid Dose	High Dose
Uterus w/ [g] cervix/BWt (%)	Mean	0.20062	0.22243	0.31484	0.21360
	SD	0.028173	0.055693	0.122672	0.020472
	N	5	5	5	5
	%Diff	-	10.9	56.9	6.5
Uterus w/ [g] cervix/BrWt (ratio)	Mean	0.28802	0.30737	0.42294	0.32980
	SD	0.030522	0.091937	0.150586	0.054956
	N	5	5	5	5
	%Diff	-	6.7	46.8	14.5

[g] - Anova & Dunnett(Log)

Table 7
Summary of Microscopic Pathology Findings (Day 8)

2759-001 - Summary of Microscopic Observations, Day 8

Sex: Male	Vehicle SS	Low Dose SS	Mid Dose SS	High Dose SS
Number of Animals:	5	5	5	5
**brain**				
Examined	5	5	5	5
No Visible Lesions	5	5	5	5
**dorsal root ganglia lumbar, right**				
Examined	5	5	5	5
No Visible Lesions	5	5	4	4
infiltration, mononuclear cell, perivascular	0	0	1	1
.... minimal	0	0	1	1
**dorsal root ganglia, cervical, right**				
Examined	5	5	5	5
No Visible Lesions	5	5	5	5
**dorsal root ganglia, thoracic, right**				
Examined	5	5	5	5
No Visible Lesions	5	5	5	5
**heart**				
Examined	5	5	5	5
No Visible Lesions	5	5	5	4
cardiomyopathy	0	0	0	1
.... minimal	0	0	0	1
**kidneys**				
Examined	5	5	5	5
No Visible Lesions	4	3	4	5
infiltration, mononuclear cell	1	1	0	0
.... minimal	1	1	0	0
mineralization	0	1	1	0
.... minimal	0	1	1	0
**liver**				
Examined	5	5	5	5
No Visible Lesions	4	3	0	3
hemorrhage	0	0	1	0
.... minimal	0	0	1	0
infiltration, mononuclear cell	1	2	4	2
.... minimal	1	2	4	2

2759-001 - Summary of Microscopic Observations, Day 8

Sex: Male	Vehicle	Low Dose	Mid Dose	High Dose
	SS	SS	SS	SS
Number of Animals:	5	5	5	5
**lung**				
Examined	5	5	5	5
No Visible Lesions	4	2	0	5
hemorrhage	1	0	0	0
.... minimal	1	0	0	0
infiltration, mononuclear cell	0	3	4	0
.... minimal	0	3	4	0
macrophages, alveolar	0	0	0	0
.... minimal	0	0	0	0
infiltration, mixed cell, perivascular	0	0	1	0
.... minimal	0	0	1	0
**lymph node, mandibular**				
Examined	5	4	5	5
No Visible Lesions	5	4	5	5
**lymph node, mesenteric**				
Examined	5	5	5	5
No Visible Lesions	5	5	5	5
**skeletal muscle, biceps femoris**				
Examined	5	5	5	5
No Visible Lesions	5	5	5	5
**skeletal muscle, gastrocnemius**				
Examined	5	5	5	5
No Visible Lesions	5	5	5	5
**spinal cord, cervical**				
Examined	5	5	5	5
No Visible Lesions	5	5	5	5
**spinal cord, lumbar**				
Examined	5	5	5	5
No Visible Lesions	5	5	5	5
gliosis	0	0	0	0
.... minimal	0	0	0	0
infiltration, mononuclear cell, perivascular	0	0	0	0
.... minimal	0	0	0	0

2759-001 - Summary of Microscopic Observations, Day 8

Sex: Male	Vehicle	Low Dose	Mid Dose	High Dose
	SS	SS	SS	SS
Number of Animals:	5	5	5	5
**spinal cord, thoracic**				
Examined	5	5	5	5
No Visible Lesions	5	5	5	5
gliosis	0	0	0	0
.... minimal	0	0	0	0
infiltration, mononuclear cell, perivascular	0	0	0	0
.... minimal	0	0	0	0
**spleen**				
Examined	5	5	5	5
No Visible Lesions	5	5	5	5
depletion, lymphoid, marginal zone	0	0	0	0
.... minimal	0	0	0	0
.... mild	0	0	0	0
.... marked	0	0	0	0
hyperplasia, lymphoid, follicular	0	0	0	0
.... minimal	0	0	0	0
**testes**				
Examined	5	5	5	5
No Visible Lesions	5	5	5	5
**thymus**				
Examined	4	5	5	5
No Visible Lesions	4	4	4	4
hemorrhage	0	1	1	1
.... minimal	0	1	1	1
**dorsal root ganglia, cervical, left**				
Examined	5	5	5	5
No Visible Lesions	5	5	5	5
**dorsal root ganglia, thoracic, left**				
Examined	5	5	5	5
No Visible Lesions	5	5	5	5
**dorsal root ganglia, lumbar, left**				
Examined	5	5	5	5
No Visible Lesions	4	4	5	4

2759-001 - Summary of Microscopic Observations, Day 8

Sex: Male	Vehicle<br>SS	Low Dose<br>SS	Mid Dose<br>SS	High Dose<br>SS
Number of Animals:	5	5	5	5
**dorsal root ganglia, lumbar, left (Continued...)**				
infiltration, mononuclear cell, perivascular	1	1	0	1
.... minimal	1	1	0	1

2759-001 - Summary of Microscopic Observations, Day 8

Sex: Female	Vehicle SS	Low Dose SS	Mid Dose SS	High Dose SS
Number of Animals:	5	5	5	5
**brain**				
Examined	5	5	5	5
No Visible Lesions	5	5	5	5
**dorsal root ganglia lumbar, right**				
Examined	5	5	5	5
No Visible Lesions	4	4	4	5
infiltration, mononuclear cell, perivascular	1	1	1	0
.... minimal	1	1	1	0
**dorsal root ganglia, cervical, right**				
Examined	5	5	5	5
No Visible Lesions	5	5	5	5
**dorsal root ganglia, thoracic, right**				
Examined	5	4	5	5
No Visible Lesions	5	4	5	5
**heart**				
Examined	5	5	5	5
No Visible Lesions	5	5	5	5
cardiomyopathy	0	0	0	0
.... minimal	0	0	0	0
**kidneys**				
Examined	5	5	5	5
No Visible Lesions	2	5	4	3
infiltration, mononuclear cell	1	0	0	1
.... minimal	1	0	0	1
mineralization	2	0	1	1
.... minimal	2	0	1	1
**liver**				
Examined	5	5	5	5
No Visible Lesions	2	3	3	5
hemorrhage	0	0	0	0
.... minimal	0	0	0	0
infiltration, mononuclear cell	3	2	2	0
.... minimal	3	2	2	0

2759-001 - Summary of Microscopic Observations, Day 8

Sex: Female	Vehicle	Low Dose	Mid Dose	High Dose
	SS	SS	SS	SS
Number of Animals:	5	5	5	5
**lung**				
Examined	5	5	5	5
No Visible Lesions	3	2	5	4
hemorrhage	0	0	0	0
.... minimal	0	0	0	0
infiltration, mononuclear cell	1	3	0	1
.... minimal	1	3	0	1
macrophages, alveolar	1	0	0	0
.... minimal	1	0	0	0
infiltration, mixed cell, perivascular	0	0	0	0
.... minimal	0	0	0	0
**lymph node, mandibular**				
Examined	5	5	5	5
No Visible Lesions	5	5	5	5
**lymph node, mesenteric**				
Examined	5	5	5	5
No Visible Lesions	5	5	5	5
**ovaries**				
Examined	5	5	5	5
No Visible Lesions	5	5	5	5
**skeletal muscle, biceps femoris**				
Examined	5	5	5	5
No Visible Lesions	5	5	5	5
**skeletal muscle, gastrocnemius**				
Examined	5	5	5	5
No Visible Lesions	5	5	5	5
**spinal cord, cervical**				
Examined	5	5	5	5
No Visible Lesions	5	5	5	5
**spinal cord, lumbar**				
Examined	5	5	5	5
No Visible Lesions	5	5	3	5
gliosis	0	0	2	0
.... minimal	0	0	2	0

2759-001 - Summary of Microscopic Observations, Day 8

Sex: Female	Vehicle SS	Low Dose SS	Mid Dose SS	High Dose SS
Number of Animals:	5	5	5	5
**spinal cord, lumbar (Continued...)**				
infiltration, mononuclear cell, perivascular	0	0	2	0
.... minimal	0	0	2	0
**spinal cord, thoracic**				
Examined	5	5	5	5
No Visible Lesions	5	5	4	5
gliosis	0	0	1	0
.... minimal	0	0	1	0
infiltration, mononuclear cell, perivascular	0	0	1	0
.... minimal	0	0	1	0
**spleen**				
Examined	5	5	5	5
No Visible Lesions	3	5	4	0
depletion, lymphoid, marginal zone	0	0	0	5
.... minimal	0	0	0	3
.... mild	0	0	0	1
.... marked	0	0	0	1
hyperplasia, lymphoid, follicular	2	0	1	0
.... minimal	2	0	1	0
**thymus**				
Examined	5	5	5	5
No Visible Lesions	5	2	5	5
hemorrhage	0	3	0	0
.... minimal	0	3	0	0
**dorsal root ganglia, cervical, left**				
Examined	5	5	5	4
No Visible Lesions	5	5	5	4
**dorsal root ganglia, thoracic, left**				
Examined	5	3	5	5
No Visible Lesions	5	3	5	5
**dorsal root ganglia, lumbar, left**				
Examined	4	5	5	5
No Visible Lesions	4	4	5	4

2759-001 - Summary of Microscopic Observations, Day 8

Sex: Female	Vehicle	Low Dose	Mid Dose	High Dose
	SS	SS	SS	SS
Number of Animals:	5	5	5	5
**dorsal root ganglia, lumbar, left (Continued...)**				
infiltration, mononuclear cell, perivascular	0	1	0	1
.... minimal	0	1	0	1

Table 8
Summary of Microscopic Pathology Findings (Day 29)

2759-001 - Summary of Microscopic Observations, Day 29

Sex: Male	Vehicle	Low Dose	Mid Dose	High Dose
	SS	SS	SS	SS
Number of Animals:	5	5	5	5
**brain**				
Examined	5	5	5	5
No Visible Lesions	5	5	5	5
**dorsal root ganglia lumbar, right**				
Examined	5	5	5	5
No Visible Lesions	5	3	4	3
infiltration, mononuclear cell, perivascular	0	2	1	2
.... minimal	0	1	1	2
.... mild	0	1	0	0
**dorsal root ganglia, cervical, right**				
Examined	5	5	5	5
No Visible Lesions	5	5	5	5
**dorsal root ganglia, thoracic, right**				
Examined	5	5	5	5
No Visible Lesions	5	5	5	5
**heart**				
Examined	5	5	5	5
No Visible Lesions	4	3	1	5
cardiomyopathy	1	2	4	0
.... minimal	1	2	4	0
infiltration, mononuclear cell	0	0	0	0
.... minimal	0	0	0	0
**kidneys**				
Examined	5	5	5	5
No Visible Lesions	5	5	4	3
cast/casts	0	0	0	0
.... minimal	0	0	0	0
cyst	0	0	0	0
.... minimal	0	0	0	0
infiltration, mononuclear cell	0	0	0	1
.... minimal	0	0	0	1
mineralization	0	0	1	1
.... minimal	0	0	1	1

2759-001 - Summary of Microscopic Observations, Day 29

Sex: Male	Vehicle	Low Dose	Mid Dose	High Dose
	SS	SS	SS	SS
Number of Animals:	5	5	5	5
**liver**				
Examined	5	5	5	5
No Visible Lesions	3	0	3	1
infiltration, mononuclear cell	1	5	2	4
.... minimal	1	5	2	4
congestion	1	0	0	0
.... minimal	1	0	0	0
**lung**				
Examined	5	5	5	5
No Visible Lesions	3	3	3	4
hemorrhage	1	0	0	0
.... minimal	0	0	0	0
.... mild	1	0	0	0
infiltration, mononuclear cell	1	2	2	1
.... minimal	1	2	2	1
**lymph node, mandibular**				
Examined	5	5	5	5
No Visible Lesions	5	5	5	5
**lymph node, mesenteric**				
Examined	5	5	5	5
No Visible Lesions	4	5	5	4
erythrocytosis/erythrophagocytosis, sinus	1	0	0	0
.... minimal	1	0	0	0
infiltration, mast cell	0	0	0	1
.... minimal	0	0	0	1
**skeletal muscle, biceps femoris**				
Examined	5	5	5	5
No Visible Lesions	5	5	5	5
degeneration	0	0	0	0
.... minimal	0	0	0	0
**skeletal muscle, gastrocnemius**				
Examined	5	5	5	5
No Visible Lesions	5	5	4	5
degeneration	0	0	1	0

2759-001 - Summary of Microscopic Observations, Day 29

Sex: Male	Vehicle	Low Dose	Mid Dose	High Dose
	SS	SS	SS	SS
Number of Animals:	5	5	5	5
**skeletal muscle, gastrocnemius (Continued...)**				
.... minimal	0	0	1	0
**skin**				
Examined	0	0	0	1
hyperkeratosis	-	-	-	1
.... minimal	-	-	-	1
inflammation	-	-	-	1
.... minimal	-	-	-	1
**spinal cord, cervical**				
Examined	5	5	5	5
No Visible Lesions	5	5	5	5
**spinal cord, lumbar**				
Examined	5	5	5	5
No Visible Lesions	5	5	5	5
**spinal cord, thoracic**				
Examined	5	5	5	5
No Visible Lesions	5	5	5	5
**spleen**				
Examined	5	5	5	5
No Visible Lesions	2	4	3	3
hyperplasia, lymphoid, follicular	3	1	2	2
.... minimal	3	1	2	2
**testes**				
Examined	5	5	5	5
No Visible Lesions	5	5	5	5
**thymus**				
Examined	5	5	5	5
No Visible Lesions	5	4	5	3
hemorrhage	0	1	0	2
.... minimal	0	1	0	2
**dorsal root ganglia, cervical, left**				
Examined	5	5	5	5
No Visible Lesions	5	5	5	5

2759-001 - Summary of Microscopic Observations, Day 29

Sex: Male	Vehicle SS	Low Dose SS	Mid Dose SS	High Dose SS
Number of Animals:	5	5	5	5
**dorsal root ganglia, thoracic, left**				
Examined	5	4	5	5
No Visible Lesions	5	4	5	5
**dorsal root ganglia, lumbar, left**				
Examined	5	5	5	5
No Visible Lesions	5	4	4	4
infiltration, mononuclear cell, perivascular	0	1	1	1
.... minimal	0	1	1	1

2759-001 - Summary of Microscopic Observations, Day 29

Sex: Female	Vehicle	Low Dose	Mid Dose	High Dose
	SS	SS	SS	SS
Number of Animals:	5	5	5	5
**brain**				
Examined	5	5	5	5
No Visible Lesions	5	5	5	5
**dorsal root ganglia lumbar, right**				
Examined	5	5	5	5
No Visible Lesions	4	4	5	4
infiltration, mononuclear cell, perivascular	1	1	0	1
.... minimal	1	1	0	1
.... mild	0	0	0	0
**dorsal root ganglia, cervical, right**				
Examined	5	5	5	5
No Visible Lesions	5	5	5	5
**dorsal root ganglia, thoracic, right**				
Examined	5	5	5	5
No Visible Lesions	5	5	5	5
**heart**				
Examined	5	5	5	5
No Visible Lesions	5	4	5	3
cardiomyopathy	0	1	0	1
.... minimal	0	1	0	1
infiltration, mononuclear cell	0	0	0	1
.... minimal	0	0	0	1
**kidneys**				
Examined	5	5	5	5
No Visible Lesions	4	4	3	1
cast/casts	0	1	0	0
.... minimal	0	1	0	0
cyst	1	0	0	0
.... minimal	1	0	0	0
infiltration, mononuclear cell	0	1	0	1
.... minimal	0	1	0	1
mineralization	0	0	2	3
.... minimal	0	0	2	3

2759-001 - Summary of Microscopic Observations, Day 29

Sex: Female	Vehicle SS	Low Dose SS	Mid Dose SS	High Dose SS
Number of Animals:	5	5	5	5
**liver**				
Examined	5	5	5	5
No Visible Lesions	1	4	3	3
infiltration, mononuclear cell	4	1	2	2
.... minimal	4	1	2	2
congestion	0	0	0	0
.... minimal	0	0	0	0
**lung**				
Examined	5	5	5	5
No Visible Lesions	2	5	5	4
hemorrhage	0	0	0	1
.... minimal	0	0	0	1
.... mild	0	0	0	0
infiltration, mononuclear cell	3	0	0	0
.... minimal	3	0	0	0
**lymph node, mandibular**				
Examined	5	5	5	5
No Visible Lesions	5	5	5	5
**lymph node, mesenteric**				
Examined	5	5	5	5
No Visible Lesions	5	5	5	5
erythrocytosis/erythrophagocytosis, sinus	0	0	0	0
.... minimal	0	0	0	0
infiltration, mast cell	0	0	0	0
.... minimal	0	0	0	0
**ovaries**				
Examined	5	5	5	5
No Visible Lesions	5	5	5	5
**skeletal muscle, biceps femoris**				
Examined	5	5	5	5
No Visible Lesions	5	5	4	5
degeneration	0	0	1	0
.... minimal	0	0	1	0

2759-001 - Summary of Microscopic Observations, Day 29

Sex: Female	Vehicle SS	Low Dose SS	Mid Dose SS	High Dose SS
Number of Animals:	5	5	5	5
**skeletal muscle, gastrocnemius**				
Examined	5	5	5	5
No Visible Lesions	5	5	5	4
degeneration	0	0	0	1
.... minimal	0	0	0	1
**spinal cord, cervical**				
Examined	5	5	5	5
No Visible Lesions	5	5	5	5
**spinal cord, lumbar**				
Examined	5	5	5	5
No Visible Lesions	5	5	5	5
**spinal cord, thoracic**				
Examined	5	5	5	5
No Visible Lesions	5	5	5	5
**spleen**				
Examined	5	5	5	5
No Visible Lesions	4	3	3	2
hyperplasia, lymphoid, follicular	1	2	2	3
.... minimal	1	2	2	3
**thymus**				
Examined	5	5	5	5
No Visible Lesions	4	5	5	5
hemorrhage	1	0	0	0
.... minimal	1	0	0	0
**dorsal root ganglia, cervical, left**				
Examined	5	5	5	5
No Visible Lesions	5	5	5	5
**dorsal root ganglia, thoracic, left**				
Examined	5	5	5	5
No Visible Lesions	5	5	5	5
**dorsal root ganglia, lumbar, left**				
Examined	5	5	5	5
No Visible Lesions	5	4	3	3
infiltration, mononuclear cell, perivascular	0	1	2	2
.... minimal	0	1	2	2

Table 9
Summary of Microscopic Pathology Findings (Day 91)

2759-001 - Summary of Microscopic Observations, Day 91

Sex: Male	Vehicle	Low Dose	Mid Dose	High Dose
	SS	SS	SS	SS
Number of Animals:	5	5	5	5
**brain**				
Examined	5	5	5	5
No Visible Lesions	5	5	5	5
**dorsal root ganglia lumbar, right**				
Examined	5	5	5	5
No Visible Lesions	5	5	5	5
infiltration, mononuclear cell, perivascular	0	0	0	0
.... minimal	0	0	0	0
**dorsal root ganglia, cervical, right**				
Examined	5	5	5	5
No Visible Lesions	5	5	5	5
**dorsal root ganglia, thoracic, right**				
Examined	5	5	5	5
No Visible Lesions	5	5	5	5
**heart**				
Examined	5	5	5	5
No Visible Lesions	3	1	3	2
cardiomyopathy	2	3	2	3
.... minimal	2	3	1	3
.... mild	0	0	1	0
fibrosis	0	1	0	0
.... minimal	0	1	0	0
**kidneys**				
Examined	5	5	5	5
No Visible Lesions	4	5	5	5
infiltration, mononuclear cell	0	0	0	0
.... minimal	0	0	0	0
inflammation, subacute/chronic	1	0	0	0
.... moderate	1	0	0	0
mineralization	0	0	0	0
.... minimal	0	0	0	0
**liver**				
Examined	5	5	5	5

2759-001 - Summary of Microscopic Observations, Day 91

Sex: Male	Vehicle	Low Dose	Mid Dose	High Dose
	SS	SS	SS	SS
Number of Animals:	5	5	5	5
**liver (Continued...)**				
No Visible Lesions	1	0	0	1
infiltration, mononuclear cell	4	5	5	4
.... minimal	4	5	5	4
**lung**				
Examined	5	5	5	5
No Visible Lesions	3	3	3	4
infiltration, mononuclear cell	2	2	2	1
.... minimal	2	2	2	1
macrophages, alveolar	0	0	0	0
.... minimal	0	0	0	0
**lymph node, mandibular**				
Examined	5	5	5	5
No Visible Lesions	5	5	5	5
**lymph node, mesenteric**				
Examined	5	5	5	5
No Visible Lesions	5	5	5	5
**skeletal muscle, biceps femoris**				
Examined	5	5	5	5
No Visible Lesions	5	5	5	5
**skeletal muscle, gastrocnemius**				
Examined	5	5	5	5
No Visible Lesions	5	5	5	4
degeneration	0	0	0	1
.... minimal	0	0	0	1
**spinal cord, cervical**				
Examined	5	5	5	5
No Visible Lesions	5	5	5	5
**spinal cord, lumbar**				
Examined	5	5	5	5
No Visible Lesions	5	5	5	5
**spinal cord, thoracic**				
Examined	5	5	5	5
No Visible Lesions	5	5	5	5

2759-001 - Summary of Microscopic Observations, Day 91

Sex: Male	Vehicle	Low Dose	Mid Dose	High Dose
	SS	SS	SS	SS
Number of Animals:	5	5	5	5
**spleen**				
Examined	5	5	5	5
No Visible Lesions	5	4	5	4
hyperplasia, lymphoid, follicular	0	1	0	1
.... minimal	0	1	0	1
**testes**				
Examined	5	5	5	5
No Visible Lesions	5	5	5	5
**thymus**				
Examined	5	5	5	5
No Visible Lesions	4	2	4	5
hemorrhage	1	3	1	0
.... minimal	1	3	1	0
**dorsal root ganglia, cervical, left**				
Examined	5	5	5	5
No Visible Lesions	5	5	5	5
**dorsal root ganglia, thoracic, left**				
Examined	5	5	5	5
No Visible Lesions	5	5	5	5
**dorsal root ganglia, lumbar, left**				
Examined	5	5	5	5
No Visible Lesions	5	4	5	5
infiltration, mononuclear cell, perivascular	0	1	0	0
.... minimal	0	1	0	0

2759-001 - Summary of Microscopic Observations, Day 91

Sex: Female	Vehicle	Low Dose	Mid Dose	High Dose
	SS	SS	SS	SS
Number of Animals:	5	5	5	5
**brain**				
Examined	5	5	5	5
No Visible Lesions	5	5	5	5
**dorsal root ganglia lumbar, right**				
Examined	5	5	5	5
No Visible Lesions	5	5	2	5
infiltration, mononuclear cell, perivascular	0	0	3	0
.... minimal	0	0	3	0
**dorsal root ganglia, cervical, right**				
Examined	5	5	5	5
No Visible Lesions	5	5	5	5
**dorsal root ganglia, thoracic, right**				
Examined	5	5	5	5
No Visible Lesions	5	5	5	5
**heart**				
Examined	5	5	5	5
No Visible Lesions	5	5	5	5
cardiomyopathy	0	0	0	0
.... minimal	0	0	0	0
.... mild	0	0	0	0
fibrosis	0	0	0	0
.... minimal	0	0	0	0
**kidneys**				
Examined	5	5	5	5
No Visible Lesions	4	5	1	4
infiltration, mononuclear cell	0	0	1	0
.... minimal	0	0	1	0
inflammation, subacute/chronic	0	0	0	0
.... moderate	0	0	0	0
mineralization	1	0	3	1
.... minimal	1	0	3	1
**liver**				
Examined	5	5	5	5

2759-001 - Summary of Microscopic Observations, Day 91

Sex: Female	Vehicle SS	Low Dose SS	Mid Dose SS	High Dose SS
Number of Animals:	5	5	5	5
**liver (Continued...)**				
No Visible Lesions	1	4	3	1
infiltration, mononuclear cell	4	1	2	4
.... minimal	4	1	2	4
**lung**				
Examined	5	5	5	5
No Visible Lesions	4	5	5	2
infiltration, mononuclear cell	1	0	0	2
.... minimal	1	0	0	2
macrophages, alveolar	0	0	0	1
.... minimal	0	0	0	1
**lymph node, mandibular**				
Examined	5	5	5	5
No Visible Lesions	5	5	5	5
**lymph node, mesenteric**				
Examined	5	5	5	5
No Visible Lesions	5	5	5	5
**ovaries**				
Examined	5	5	5	5
No Visible Lesions	5	5	5	5
**skeletal muscle, biceps femoris**				
Examined	5	5	5	5
No Visible Lesions	5	5	5	5
**skeletal muscle, gastrocnemius**				
Examined	5	5	5	5
No Visible Lesions	5	5	5	5
degeneration	0	0	0	0
.... minimal	0	0	0	0
**spinal cord, cervical**				
Examined	5	5	5	5
No Visible Lesions	5	5	5	5
**spinal cord, lumbar**				
Examined	5	5	5	5
No Visible Lesions	5	5	5	5

2759-001 - Summary of Microscopic Observations, Day 91

Sex: Female	Vehicle SS	Low Dose SS	Mid Dose SS	High Dose SS
Number of Animals:	5	5	5	5
**spinal cord, thoracic**				
Examined	5	5	5	5
No Visible Lesions	5	5	5	5
**spleen**				
Examined	5	5	5	5
No Visible Lesions	5	4	4	4
hyperplasia, lymphoid, follicular	0	1	1	1
.... minimal	0	1	1	1
**thymus**				
Examined	5	5	5	5
No Visible Lesions	5	4	4	5
hemorrhage	0	1	1	0
.... minimal	0	1	1	0
**dorsal root ganglia, cervical, left**				
Examined	5	5	5	5
No Visible Lesions	5	5	5	5
**dorsal root ganglia, thoracic, left**				
Examined	5	5	5	5
No Visible Lesions	5	5	5	5
**dorsal root ganglia, lumbar, left**				
Examined	5	5	5	5
No Visible Lesions	4	5	3	5
infiltration, mononuclear cell, perivascular	1	0	2	0
.... minimal	1	0	2	0

Appendix 1
Deviations

DEVIATIONS

All deviations (if any) that occurred during this study phase have been acknowledged by the Study Director, assessed for impact, and documented in the study records. All protocol deviations and those SOP deviations regarded as significant are listed below. None of the deviations were considered to have impacted the overall integrity of the study or the interpretation of the study results and conclusions.

- There were no deviations to report for this study phase.

Appendix 2
Individual Organ Weight Values (Day 8)

2759-001
A Single Dose Toxicity Study of Test Article Administered by Intrathecal Injection in Rats

Individual Organ Weight Values
Day 8

Sex: Male   Day: 8 Relative to Start Date

Vehicle	Body Weight (g)	Brain (g)	Brain/BWt (%)	Adrenal glands (g)	Adrenal gl/ BWt (%)	Adrenal gl/ BrWt (ratio)	Epididymides (g)
1006	330	1.941	0.5882	0.041	0.0124	0.0211	0.600
1007	306	1.696	0.5542	0.083	0.0271	0.0489	0.719
1008	316	2.030	0.6424	0.051	0.0161	0.0251	0.792
1009	321	2.058	0.6411	0.060	0.0187	0.0292	0.729
1010	336	1.900	0.5655	0.057	0.0170	0.0300	0.680

2759-001
A Single Dose Toxicity Study of Test Article Administered by Intrathecal Injection in Rats

Individual Organ Weight Values
Day 8

Sex: Male Day: 8 Relative to Start Date

Vehicle	Epididymides /BWt (%)	Epididymides /BrWt (ratio)	Heart (g)	Heart/BWt (%)	Heart/BrWt (ratio)	Kidneys (g)	Kidneys/BWt (%)
1006	0.1818	0.3091	1.254	0.3800	0.6461	3.415	1.0348
1007	0.2350	0.4239	1.202	0.3928	0.7087	2.820	0.9216
1008	0.2506	0.3901	1.725	0.5459	0.8498	3.216	1.0177
1009	0.2271	0.3542	1.482	0.4617	0.7201	3.151	0.9816
1010	0.2024	0.3579	1.209	0.3598	0.6363	3.142	0.9351

2759-001
A Single Dose Toxicity Study of Test Article Administered by Intrathecal Injection in Rats

Individual Organ Weight Values
Day 8

Sex: Male Day: 8 Relative to Start Date

Vehicle	Kidneys/BrWt (ratio)	Liver (g)	Liver/BWt (%)	Liver/BrWt (ratio)	Lung (g)	Lung/BWt (%)	Lung/BrWt (ratio)
1006	1.7594	15.311	4.6397	7.8882	1.818	0.5509	0.9366
1007	1.6627	14.150	4.6242	8.3432	1.446	0.4725	0.8526
1008	1.5842	15.147	4.7934	7.4616	1.734	0.5487	0.8542
1009	1.5311	13.269	4.1336	6.4475	1.598	0.4978	0.7765
1010	1.6537	16.108	4.7940	8.4779	1.478	0.4399	0.7779

2759-001
A Single Dose Toxicity Study of Test Article Administered by Intrathecal Injection in Rats

Individual Organ Weight Values
Day 8

Sex: Male Day: 8 Relative to Start Date

Vehicle	Pituitary (fixed) (g)	Pituitary (fixed)/BWt (%)	Pituitary (fixed)/BrWt (ratio)	Prostate gland (g)	Prostate gland/BWt (%)	Prostate gland/BrWt (ratio)	Sal, mand/ sub., rt (g)
1006	0.0162	0.0049	0.0083	0.488	0.1479	0.2514	0.281
1007	0.0149	0.0049	0.0088	0.838	0.2739	0.4941	0.324
1008	0.0186	0.0059	0.0092	0.770	0.2437	0.3793	0.289
1009	0.0170	0.0053	0.0083	0.691	0.2153	0.3358	0.309
1010	0.0118	0.0035	0.0062	0.698	0.2077	0.3674	0.265

2759-001
A Single Dose Toxicity Study of Test Article Administered by Intrathecal Injection in Rats

Individual Organ Weight Values
Day 8

Sex: Male Day: 8 Relative to Start Date

Vehicle	Sal gl mand/ sub., rt/BWt (%)	Sal gl mand/ sub.,rt/BrWt (ratio)	Spleen (g)	Spleen/BWt (%)	Spleen/BrWt (ratio)	Testes (g)	Testes/BWt (%)
1006	0.0852	0.1448	0.763	0.2312	0.3931	3.120	0.9455
1007	0.1059	0.1910	0.775	0.2533	0.4570	2.777	0.9075
1008	0.0915	0.1424	0.779	0.2465	0.3837	3.492	1.1051
1009	0.0963	0.1501	0.992	0.3090	0.4820	3.371	1.0502
1010	0.0789	0.1395	0.782	0.2327	0.4116	3.117	0.9277

2759-001
A Single Dose Toxicity Study of Test Article Administered by Intrathecal Injection in Rats

Individual Organ Weight Values
Day 8

Sex: Male Day: 8 Relative to Start Date

Vehicle	Testes/BrWt (ratio)	Thymus (g)	Thymus/BWt (%)	Thymus/BrWt (ratio)	Thyroid/para (fixed) (g)	Thyroid/para (fixed)/BWt (%)	Thyroid/para (fixed)/BrWt (ratio)
1006	1.6074	0.675	0.2045	0.3478	0.0227	0.0069	0.0117
1007	1.6374	0.622	0.2033	0.3667	0.0267	0.0087	0.0157
1008	1.7202	0.507	0.1604	0.2498	0.0294	0.0093	0.0145
1009	1.6380	0.690	0.2150	0.3353	0.0236	0.0074	0.0115
1010	1.6405	0.977	0.2908	0.5142	0.0250	0.0074	0.0132

2759-001
A Single Dose Toxicity Study of Test Article Administered by Intrathecal Injection in Rats

Individual Organ Weight Values
Day 8

Sex: Male Day: 8 Relative to Start Date

Low Dose	Body Weight (g)	Brain (g)	Brain/BWt (%)	Adrenal glands (g)	Adrenal gl/ BWt (%)	Adrenal gl/ BrWt (ratio)	Epididymides (g)
2006	304	1.915	0.6299	0.049	0.0161	0.0256	0.727
2007	303	1.918	0.6330	0.049	0.0162	0.0255	0.641
2008	334	1.961	0.5871	0.092	0.0275	0.0469	0.730
2009	289	1.812	0.6270	0.051	0.0176	0.0281	0.607
2010	303	1.915	0.6320	0.072	0.0238	0.0376	0.682

2759-001
A Single Dose Toxicity Study of Test Article Administered by Intrathecal Injection in Rats

Individual Organ Weight Values
Day 8

Sex: Male Day: 8 Relative to Start Date

Low Dose	Epididymides /BWt (%)	Epididymides /BrWt (ratio)	Heart (g)	Heart/BWt (%)	Heart/BrWt (ratio)	Kidneys (g)	Kidneys/BWt (%)
2006	0.2391	0.3796	1.105	0.3635	0.5770	2.545	0.8372
2007	0.2116	0.3342	1.263	0.4168	0.6585	2.830	0.9340
2008	0.2186	0.3723	1.685	0.5045	0.8593	3.286	0.9838
2009	0.2100	0.3350	1.111	0.3844	0.6131	2.365	0.8183
2010	0.2251	0.3561	1.528	0.5043	0.7979	2.801	0.9244

2759-001
A Single Dose Toxicity Study of Test Article Administered by Intrathecal Injection in Rats

Individual Organ Weight Values
Day 8

Sex: Male Day: 8 Relative to Start Date

Low Dose	Kidneys/BrWt (ratio)	Liver (g)	Liver/BWt (%)	Liver/BrWt (ratio)	Lung (g)	Lung/BWt (%)	Lung/BrWt (ratio)
2006	1.3290	11.583	3.8102	6.0486	1.560	0.5132	0.8146
2007	1.4755	13.541	4.4690	7.0600	1.477	0.4875	0.7701
2008	1.6757	13.178	3.9455	6.7200	1.835	0.5494	0.9357
2009	1.3052	11.251	3.8931	6.2092	1.389	0.4806	0.7666
2010	1.4627	12.880	4.2508	6.7258	2.137	0.7053	1.1159

2759-001
A Single Dose Toxicity Study of Test Article Administered by Intrathecal Injection in Rats

Individual Organ Weight Values
Day 8

Sex: Male Day: 8 Relative to Start Date

Low Dose	Pituitary (fixed) (g)	Pituitary (fixed)/BWt (%)	Pituitary (fixed)/BrWt (ratio)	Prostate gland (g)	Prostate gland/BWt (%)	Prostate gland/BrWt (ratio)	Sal, mand/ sub., rt (g)
2006	0.0145	0.0048	0.0076	0.769	0.2530	0.4016	0.248
2007	0.0158	0.0052	0.0082	0.548	0.1809	0.2857	0.274
2008	0.0152	0.0046	0.0078	0.675	0.2021	0.3442	0.328
2009	0.0136	0.0047	0.0075	0.584	0.2021	0.3223	0.231
2010	0.0148	0.0049	0.0077	0.624	0.2059	0.3258	0.282

2759-001
A Single Dose Toxicity Study of Test Article Administered by Intrathecal Injection in Rats

Individual Organ Weight Values
Day 8

Sex: Male Day: 8 Relative to Start Date

Low Dose	Sal gl mand/ sub., rt/BWt (%)	Sal gl mand/ sub.,rt/BrWt (ratio)	Spleen (g)	Spleen/BWt (%)	Spleen/BrWt (ratio)	Testes (g)	Testes/BWt (%)
2006	0.0816	0.1295	0.622	0.2046	0.3248	3.003	0.9878
2007	0.0904	0.1429	0.856	0.2825	0.4463	2.901	0.9574
2008	0.0982	0.1673	0.935	0.2799	0.4768	3.879	1.1614
2009	0.0799	0.1275	0.811	0.2806	0.4476	2.820	0.9758
2010	0.0931	0.1473	0.849	0.2802	0.4433	3.314	1.0937

2759-001
A Single Dose Toxicity Study of Test Article Administered by Intrathecal Injection in Rats

Individual Organ Weight Values
Day 8

Sex: Male Day: 8 Relative to Start Date

Low Dose	Testes/BrWt (ratio)	Thymus (g)	Thymus/BWt (%)	Thymus/BrWt (ratio)	Thyroid/para (fixed) (g)	Thyroid/para (fixed)/BWt (%)	Thyroid/para (fixed)/BrWt (ratio)
2006	1.5681	0.545	0.1793	0.2846	0.0218	0.0072	0.0114
2007	1.5125	0.582	0.1921	0.3034	0.0225	0.0074	0.0117
2008	1.9781	0.998	0.2988	0.5089	0.0237	0.0071	0.0121
2009	1.5563	0.427	0.1478	0.2357	0.0185	0.0064	0.0102
2010	1.7305	0.630	0.2079	0.3290	0.0236	0.0078	0.0123

2759-001
A Single Dose Toxicity Study of Test Article Administered by Intrathecal Injection in Rats

Individual Organ Weight Values
Day 8

Sex: Male Day: 8 Relative to Start Date

Mid Dose	Body Weight (g)	Brain (g)	Brain/BWt (%)	Adrenal glands (g)	Adrenal gl/ BWt (%)	Adrenal gl/ BrWt (ratio)	Epididymides (g)
3006	324	1.945	0.6003	0.075	0.0231	0.0386	0.616
3007	314	1.928	0.6140	0.051	0.0162	0.0265	0.570
3008	338	1.902	0.5627	0.083	0.0246	0.0436	0.898
3009	317	2.026	0.6391	0.063	0.0199	0.0311	0.850
3010	308	1.994	0.6474	0.061	0.0198	0.0306	0.794

2759-001
A Single Dose Toxicity Study of Test Article Administered by Intrathecal Injection in Rats

Individual Organ Weight Values
Day 8

Sex: Male Day: 8 Relative to Start Date

Mid Dose	Epididymides /BWt (%)	Epididymides /BrWt (ratio)	Heart (g)	Heart/BWt (%)	Heart/BrWt (ratio)	Kidneys (g)	Kidneys/BWt (%)
3006	0.1901	0.3167	1.720	0.5309	0.8843	2.994	0.9241
3007	0.1815	0.2956	1.354	0.4312	0.7023	2.760	0.8790
3008	0.2657	0.4721	1.513	0.4476	0.7955	3.160	0.9349
3009	0.2681	0.4195	1.128	0.3558	0.5568	3.013	0.9505
3010	0.2578	0.3982	1.482	0.4812	0.7432	3.016	0.9792

2759-001
A Single Dose Toxicity Study of Test Article Administered by Intrathecal Injection in Rats

Individual Organ Weight Values
Day 8

Sex: Male Day: 8 Relative to Start Date

Mid Dose	Kidneys/BrWt (ratio)	Liver (g)	Liver/BWt (%)	Liver/BrWt (ratio)	Lung (g)	Lung/BWt (%)	Lung/BrWt (ratio)
3006	1.5393	15.183	4.6861	7.8062	1.617	0.4991	0.8314
3007	1.4315	13.171	4.1946	6.8314	1.762	0.5611	0.9139
3008	1.6614	14.740	4.3609	7.7497	1.830	0.5414	0.9621
3009	1.4872	12.856	4.0555	6.3455	1.921	0.6060	0.9482
3010	1.5125	12.729	4.1328	6.3837	1.731	0.5620	0.8681

2759-001
A Single Dose Toxicity Study of Test Article Administered by Intrathecal Injection in Rats

Individual Organ Weight Values
Day 8

Sex: Male Day: 8 Relative to Start Date

Mid Dose	Pituitary (fixed) (g)	Pituitary (fixed)/BWt (%)	Pituitary (fixed)/BrWt (ratio)	Prostate gland (g)	Prostate gland/BWt (%)	Prostate gland/BrWt (ratio)	Sal, mand/ sub., rt (g)
3006	0.0145	0.0045	0.0075	0.563	0.1738	0.2895	0.301
3007	0.0155	0.0049	0.0080	0.524	0.1669	0.2718	0.291
3008	0.0164	0.0049	0.0086	0.827	0.2447	0.4348	0.322
3009	0.0146	0.0046	0.0072	0.964	0.3041	0.4758	0.351
3010	0.0133	0.0043	0.0067	0.569	0.1847	0.2854	0.321

2759-001
A Single Dose Toxicity Study of Test Article Administered by Intrathecal Injection in Rats

Individual Organ Weight Values
Day 8

Sex: Male Day: 8 Relative to Start Date

Mid Dose	Sal gl mand/ sub., rt/BWt (%)	Sal gl mand/ sub.,rt/BrWt (ratio)	Spleen (g)	Spleen/BWt (%)	Spleen/BrWt (ratio)	Testes (g)	Testes/BWt (%)
3006	0.0929	0.1548	0.838	0.2586	0.4308	3.169	0.9781
3007	0.0927	0.1509	0.885	0.2818	0.4590	3.037	0.9672
3008	0.0953	0.1693	0.859	0.2541	0.4516	3.535	1.0459
3009	0.1107	0.1732	0.852	0.2688	0.4205	3.525	1.1120
3010	0.1042	0.1610	0.815	0.2646	0.4087	3.422	1.1110

2759-001
A Single Dose Toxicity Study of Test Article Administered by Intrathecal Injection in Rats

Individual Organ Weight Values
Day 8

Sex: Male Day: 8 Relative to Start Date

Mid Dose	Testes/BrWt (ratio)	Thymus (g)	Thymus/BWt (%)	Thymus/BrWt (ratio)	Thyroid/para (fixed) (g)	Thyroid/para (fixed)/BWt (%)	Thyroid/para (fixed)/BrWt (ratio)
3006	1.6293	0.636	0.1963	0.3270	0.0256	0.0079	0.0132
3007	1.5752	0.792	0.2522	0.4108	0.0249	0.0079	0.0129
3008	1.8586	0.585	0.1731	0.3076	0.0183	0.0054	0.0096
3009	1.7399	0.723	0.2281	0.3569	0.0225	0.0071	0.0111
3010	1.7161	0.687	0.2231	0.3445	0.0258	0.0084	0.0129

2759-001
A Single Dose Toxicity Study of Test Article Administered by Intrathecal Injection in Rats

Individual Organ Weight Values
Day 8

Sex: Male Day: 8 Relative to Start Date

High Dose	Body Weight (g)	Brain (g)	Brain/BWt (%)	Adrenal glands (g)	Adrenal gl/ BWt (%)	Adrenal gl/ BrWt (ratio)	Epididymides (g)
4006	293	1.967	0.6713	0.043	0.0147	0.0219	0.627
4007	329	2.018	0.6134	0.069	0.0210	0.0342	0.732
4008	318	2.043	0.6425	0.062	0.0195	0.0303	0.670
4009	316	1.980	0.6266	0.074	0.0234	0.0374	0.733
4010	296	1.791	0.6051	0.056	0.0189	0.0313	0.796

2759-001
A Single Dose Toxicity Study of Test Article Administered by Intrathecal Injection in Rats

Individual Organ Weight Values
Day 8

Sex: Male Day: 8 Relative to Start Date

High Dose	Epididymides /BWt (%)	Epididymides /BrWt (ratio)	Heart (g)	Heart/BWt (%)	Heart/BrWt (ratio)	Kidneys (g)	Kidneys/BWt (%)
4006	0.2140	0.3188	1.293	0.4413	0.6573	2.532	0.8642
4007	0.2225	0.3627	1.772	0.5386	0.8781	3.280	0.9970
4008	0.2107	0.3279	1.940	0.6101	0.9496	2.892	0.9094
4009	0.2320	0.3702	1.292	0.4089	0.6525	3.016	0.9544
4010	0.2689	0.4444	1.278	0.4318	0.7136	2.751	0.9294

2759-001
A Single Dose Toxicity Study of Test Article Administered by Intrathecal Injection in Rats

Individual Organ Weight Values
Day 8

Sex: Male Day: 8 Relative to Start Date

High Dose	Kidneys/BrWt (ratio)	Liver (g)	Liver/BWt (%)	Liver/BrWt (ratio)	Lung (g)	Lung/BWt (%)	Lung/BrWt (ratio)
4006	1.2872	11.134	3.8000	5.6604	1.474	0.5031	0.7494
4007	1.6254	14.767	4.4884	7.3176	1.938	0.5891	0.9604
4008	1.4156	13.242	4.1642	6.4816	1.824	0.5736	0.8928
4009	1.5232	13.510	4.2753	6.8232	1.889	0.5978	0.9540
4010	1.5360	12.224	4.1297	6.8252	1.536	0.5189	0.8576

2759-001
A Single Dose Toxicity Study of Test Article Administered by Intrathecal Injection in Rats

Individual Organ Weight Values
Day 8

Sex: Male   Day: 8 Relative to Start Date

High Dose	Pituitary (fixed) (g)	Pituitary (fixed)/BWt (%)	Pituitary (fixed)/BrWt (ratio)	Prostate gland (g)	Prostate gland/BWt (%)	Prostate gland/BrWt (ratio)	Sal, mand/ sub., rt (g)
4006	0.0169	0.0058	0.0086	0.595	0.2031	0.3025	0.269
4007	0.0152	0.0046	0.0075	0.628	0.1909	0.3112	0.327
4008	0.0133	0.0042	0.0065	0.820	0.2579	0.4014	0.294
4009	0.0159	0.0050	0.0080	0.617	0.1953	0.3116	0.324
4010	0.0112	0.0038	0.0063	0.757	0.2557	0.4227	0.298

2759-001
A Single Dose Toxicity Study of Test Article Administered by Intrathecal Injection in Rats

Individual Organ Weight Values
Day 8

Sex: Male Day: 8 Relative to Start Date

High Dose	Sal gl mand/ sub., rt/BWt (%)	Sal gl mand/ sub.,rt/BrWt (ratio)	Spleen (g)	Spleen/BWt (%)	Spleen/BrWt (ratio)	Testes (g)	Testes/BWt (%)
4006	0.0918	0.1368	0.828	0.2826	0.4209	3.252	1.1099
4007	0.0994	0.1620	1.032	0.3137	0.5114	3.088	0.9386
4008	0.0925	0.1439	0.913	0.2871	0.4469	3.421	1.0758
4009	0.1025	0.1636	0.778	0.2462	0.3929	3.158	0.9994
4010	0.1007	0.1664	0.935	0.3159	0.5221	3.262	1.1020

2759-001
A Single Dose Toxicity Study of Test Article Administered by Intrathecal Injection in Rats

Individual Organ Weight Values
Day 8

Sex: Male Day: 8 Relative to Start Date

High Dose	Testes/BrWt (ratio)	Thymus (g)	Thymus/BWt (%)	Thymus/BrWt (ratio)	Thyroid/para (fixed) (g)	Thyroid/para (fixed)/BWt (%)	Thyroid/para (fixed)/BrWt (ratio)
4006	1.6533	0.729	0.2488	0.3706	0.0258	0.0088	0.0131
4007	1.5302	0.777	0.2362	0.3850	0.0249	0.0076	0.0123
4008	1.6745	0.890	0.2799	0.4356	0.0148	0.0047	0.0072
4009	1.5949	0.501	0.1585	0.2530	0.0198	0.0063	0.0100
4010	1.8213	0.476	0.1608	0.2658	0.0255	0.0086	0.0142

2759-001
A Single Dose Toxicity Study of Test Article Administered by Intrathecal Injection in Rats

Individual Organ Weight Values
Day 8

Sex: Female Day: 8 Relative to Start Date

Vehicle	Body Weight (g)	Brain (g)	Brain/BWt (%)	Adrenal glands (g)	Adrenal gl/ BWt (%)	Adrenal gl/ BrWt (ratio)	Heart (g)
1506	191	1.888	0.9885	0.051	0.0267	0.0270	0.925
1507	196	1.731	0.8832	0.057	0.0291	0.0329	0.836
1508	173	1.904	1.1006	0.073	0.0422	0.0383	1.287
1509	207	1.809	0.8739	0.071	0.0343	0.0392	0.981
1510	190	1.791	0.9426	0.080	0.0421	0.0447	0.841

2759-001
A Single Dose Toxicity Study of Test Article Administered by Intrathecal Injection in Rats

Individual Organ Weight Values
Day 8

Sex: Female Day: 8 Relative to Start Date

Vehicle	Heart/BWt (%)	Heart/BrWt (ratio)	Kidneys (g)	Kidneys/BWt (%)	Kidneys/BrWt (ratio)	Liver (g)	Liver/BWt (%)
1506	0.4843	0.4899	2.013	1.0539	1.0662	8.773	4.5932
1507	0.4265	0.4830	1.874	0.9561	1.0826	8.072	4.1184
1508	0.7439	0.6759	1.834	1.0601	0.9632	7.789	4.5023
1509	0.4739	0.5423	1.917	0.9261	1.0597	8.707	4.2063
1510	0.4426	0.4696	1.722	0.9063	0.9615	7.373	3.8805

2759-001
A Single Dose Toxicity Study of Test Article Administered by Intrathecal Injection in Rats

Individual Organ Weight Values
Day 8

Sex: Female Day: 8 Relative to Start Date

Vehicle	Liver/BrWt (ratio)	Lung (g)	Lung/BWt (%)	Lung/BrWt (ratio)	Ovaries (g)	Ovaries/BWt (%)	Ovaries/BrWt (ratio)
1506	4.6467	1.263	0.6613	0.6690	0.085	0.0445	0.0450
1507	4.6632	1.188	0.6061	0.6863	0.071	0.0362	0.0410
1508	4.0909	1.119	0.6468	0.5877	0.116	0.0671	0.0609
1509	4.8132	1.467	0.7087	0.8109	0.076	0.0367	0.0420
1510	4.1167	1.119	0.5889	0.6248	0.092	0.0484	0.0514

2759-001
A Single Dose Toxicity Study of Test Article Administered by Intrathecal Injection in Rats

Individual Organ Weight Values
Day 8

Sex: Female Day: 8 Relative to Start Date

Vehicle	Oviducts (g)	Oviducts/BWt (%)	Oviducts/ BrWt (ratio)	Pituitary (fixed) (g)	Pituitary (fixed)/BWt (%)	Pituitary (fixed)/BrWt (ratio)	Sal, mand/ sub., rt (g)
1506	0.012	0.0063	0.0064	0.0185	0.0097	0.0098	0.148
1507	0.018	0.0092	0.0104	0.0201	0.0103	0.0116	0.453
1508	0.022	0.0127	0.0116	0.0171	0.0099	0.0090	0.208
1509	0.027	0.0130	0.0149	0.0153	0.0074	0.0085	0.199
1510	0.024	0.0126	0.0134	0.0157	0.0083	0.0088	0.198

2759-001
A Single Dose Toxicity Study of Test Article Administered by Intrathecal Injection in Rats

Individual Organ Weight Values
Day 8

Sex: Female Day: 8 Relative to Start Date

Vehicle	Sal gl mand/ sub., rt/BWt (%)	Sal gl mand/ sub.,rt/BrWt (ratio)	Spleen (g)	Spleen/BWt (%)	Spleen/BrWt (ratio)	Thymus (g)	Thymus/BWt (%)
1506	0.0775	0.0784	0.467	0.2445	0.2474	0.552	0.2890
1507	0.2311	0.2617	0.497	0.2536	0.2871	0.801	0.4087
1508	0.1202	0.1092	0.447	0.2584	0.2348	0.607	0.3509
1509	0.0961	0.1100	0.497	0.2401	0.2747	0.549	0.2652
1510	0.1042	0.1106	0.406	0.2137	0.2267	0.436	0.2295

2759-001
A Single Dose Toxicity Study of Test Article Administered by Intrathecal Injection in Rats

Individual Organ Weight Values
Day 8

Sex: Female Day: 8 Relative to Start Date

Vehicle	Thymus/BrWt (ratio)	Thyroid/para (fixed) (g)	Thyroid/para (fixed)/BWt (%)	Thyroid/para (fixed)/BrWt (ratio)	Uterus w/ cervix (g)	Uterus w/ cervix/BWt (%)	Uterus w/ cervix/BrWt (ratio)
1506	0.2924	0.0234	0.0123	0.0124	0.392	0.2052	0.2076
1507	0.4627	0.0216	0.0110	0.0125	0.852	0.4347	0.4922
1508	0.3188	0.0192	0.0111	0.0101	0.499	0.2884	0.2621
1509	0.3035	0.0176	0.0085	0.0097	0.433	0.2092	0.2394
1510	0.2434	0.0173	0.0091	0.0097	0.283	0.1489	0.1580

2759-001
A Single Dose Toxicity Study of Test Article Administered by Intrathecal Injection in Rats

Individual Organ Weight Values
Day 8

Sex: Female Day: 8 Relative to Start Date

Low Dose	Body Weight (g)	Brain (g)	Brain/BWt (%)	Adrenal glands (g)	Adrenal gl/ BWt (%)	Adrenal gl/ BrWt (ratio)	Heart (g)
2506	206	1.835	0.8908	0.059	0.0286	0.0322	0.828
2507	216	1.884	0.8722	0.054	0.0250	0.0287	0.872
2508	192	1.877	0.9776	0.066	0.0344	0.0352	0.933
2509	177	1.857	1.0492	0.051	0.0288	0.0275	0.805
2510	189	1.881	0.9952	0.056	0.0296	0.0298	0.783

2759-001
A Single Dose Toxicity Study of Test Article Administered by Intrathecal Injection in Rats

Individual Organ Weight Values
Day 8

Sex: Female Day: 8 Relative to Start Date

Low Dose	Heart/BWt (%)	Heart/BrWt (ratio)	Kidneys (g)	Kidneys/BWt (%)	Kidneys/BrWt (ratio)	Liver (g)	Liver/BWt (%)
2506	0.4019	0.4512	1.809	0.8782	0.9858	7.864	3.8175
2507	0.4037	0.4628	2.080	0.9630	1.1040	8.388	3.8833
2508	0.4859	0.4971	2.052	1.0688	1.0932	8.274	4.3094
2509	0.4548	0.4335	1.742	0.9842	0.9381	7.244	4.0927
2510	0.4143	0.4163	1.790	0.9471	0.9516	7.059	3.7349

2759-001
A Single Dose Toxicity Study of Test Article Administered by Intrathecal Injection in Rats

Individual Organ Weight Values
Day 8

Sex: Female Day: 8 Relative to Start Date

Low Dose	Liver/BrWt (ratio)	Lung (g)	Lung/BWt (%)	Lung/BrWt (ratio)	Ovaries (g)	Ovaries/BWt (%)	Ovaries/BrWt (ratio)
2506	4.2856	1.474	0.7155	0.8033	0.057	0.0277	0.0311
2507	4.4522	1.212	0.5611	0.6433	0.071	0.0329	0.0377
2508	4.4081	1.163	0.6057	0.6196	0.088	0.0458	0.0469
2509	3.9009	1.192	0.6734	0.6419	0.063	0.0356	0.0339
2510	3.7528	1.143	0.6048	0.6077	0.059	0.0312	0.0314

2759-001
A Single Dose Toxicity Study of Test Article Administered by Intrathecal Injection in Rats

Individual Organ Weight Values
Day 8

Sex: Female Day: 8 Relative to Start Date

Low Dose	Oviducts (g)	Oviducts/BWt (%)	Oviducts/ BrWt (ratio)	Pituitary (fixed) (g)	Pituitary (fixed)/BWt (%)	Pituitary (fixed)/BrWt (ratio)	Sal, mand/ sub., rt (g)
2506	0.021	0.0102	0.0114	0.0147	0.0071	0.0080	0.203
2507	0.025	0.0116	0.0133	0.0174	0.0081	0.0092	0.181
2508	0.025	0.0130	0.0133	0.0194	0.0101	0.0103	0.197
2509	0.024	0.0136	0.0129	0.0151	0.0085	0.0081	0.166
2510	0.023	0.0122	0.0122	0.0204	0.0108	0.0108	0.192

2759-001
A Single Dose Toxicity Study of Test Article Administered by Intrathecal Injection in Rats

Individual Organ Weight Values
Day 8

Sex: Female Day: 8 Relative to Start Date

Low Dose	Sal gl mand/ sub., rt/BWt (%)	Sal gl mand/ sub.,rt/BrWt (ratio)	Spleen (g)	Spleen/BWt (%)	Spleen/BrWt (ratio)	Thymus (g)	Thymus/BWt (%)
2506	0.0985	0.1106	0.476	0.2311	0.2594	0.563	0.2733
2507	0.0838	0.0961	0.647	0.2995	0.3434	0.410	0.1898
2508	0.1026	0.1050	0.467	0.2432	0.2488	0.601	0.3130
2509	0.0938	0.0894	0.397	0.2243	0.2138	0.436	0.2463
2510	0.1016	0.1021	0.449	0.2376	0.2387	0.551	0.2915

2759-001
A Single Dose Toxicity Study of Test Article Administered by Intrathecal Injection in Rats

Individual Organ Weight Values
Day 8

Sex: Female Day: 8 Relative to Start Date

Low Dose	Thymus/BrWt (ratio)	Thyroid/para (fixed) (g)	Thyroid/para (fixed)/BWt (%)	Thyroid/para (fixed)/BrWt (ratio)	Uterus w/ cervix (g)	Uterus w/ cervix/BWt (%)	Uterus w/ cervix/BrWt (ratio)
2506	0.3068	0.0198	0.0096	0.0108	0.879	0.4267	0.4790
2507	0.2176	0.0197	0.0091	0.0105	0.333	0.1542	0.1768
2508	0.3202	0.0173	0.0090	0.0092	0.391	0.2036	0.2083
2509	0.2348	0.0205	0.0116	0.0110	0.441	0.2492	0.2375
2510	0.2929	0.0194	0.0103	0.0103	0.451	0.2386	0.2398

2759-001
A Single Dose Toxicity Study of Test Article Administered by Intrathecal Injection in Rats

Individual Organ Weight Values
Day 8

Sex: Female Day: 8 Relative to Start Date

Mid Dose	Body Weight (g)	Brain (g)	Brain/BWt (%)	Adrenal glands (g)	Adrenal gl/ BWt (%)	Adrenal gl/ BrWt (ratio)	Heart (g)
3506	204	1.865	0.9142	0.066	0.0324	0.0354	0.760
3507	193	1.882	0.9751	0.055	0.0285	0.0292	0.851
3508	185	1.793	0.9692	0.072	0.0389	0.0402	0.755
3509	195	1.729	0.8867	0.065	0.0333	0.0376	0.837
3510	192	1.931	1.0057	0.099	0.0516	0.0513	0.960

2759-001
A Single Dose Toxicity Study of Test Article Administered by Intrathecal Injection in Rats

Individual Organ Weight Values
Day 8

Sex: Female Day: 8 Relative to Start Date

Mid Dose	Heart/BWt (%)	Heart/BrWt (ratio)	Kidneys (g)	Kidneys/BWt (%)	Kidneys/BrWt (ratio)	Liver (g)	Liver/BWt (%)
3506	0.3725	0.4075	1.848	0.9059	0.9909	7.872	3.8588
3507	0.4409	0.4522	1.944	1.0073	1.0329	8.548	4.4290
3508	0.4081	0.4211	1.854	1.0022	1.0340	8.355	4.5162
3509	0.4292	0.4841	1.859	0.9533	1.0752	8.695	4.4590
3510	0.5000	0.4972	1.912	0.9958	0.9902	8.510	4.4323

2759-001
A Single Dose Toxicity Study of Test Article Administered by Intrathecal Injection in Rats

Individual Organ Weight Values
Day 8

Sex: Female Day: 8 Relative to Start Date

Mid Dose	Liver/BrWt (ratio)	Lung (g)	Lung/BWt (%)	Lung/BrWt (ratio)	Ovaries (g)	Ovaries/BWt (%)	Ovaries/BrWt (ratio)
3506	4.2209	1.120	0.5490	0.6005	0.070	0.0343	0.0375
3507	4.5420	1.109	0.5746	0.5893	0.065	0.0337	0.0345
3508	4.6598	1.059	0.5724	0.5906	0.088	0.0476	0.0491
3509	5.0289	1.203	0.6169	0.6958	0.100	0.0513	0.0578
3510	4.4070	1.517	0.7901	0.7856	0.099	0.0516	0.0513

2759-001
A Single Dose Toxicity Study of Test Article Administered by Intrathecal Injection in Rats

Individual Organ Weight Values
Day 8

Sex: Female Day: 8 Relative to Start Date

Mid Dose	Oviducts (g)	Oviducts/BWt (%)	Oviducts/ BrWt (ratio)	Pituitary (fixed) (g)	Pituitary (fixed)/BWt (%)	Pituitary (fixed)/BrWt (ratio)	Sal, mand/ sub., rt (g)
3506	0.025	0.0123	0.0134	0.0198	0.0097	0.0106	0.215
3507	0.017	0.0088	0.0090	0.0172	0.0089	0.0091	0.170
3508	0.028	0.0151	0.0156	0.0146	0.0079	0.0081	0.197
3509	0.027	0.0138	0.0156	0.0169	0.0087	0.0098	0.196
3510	0.038	0.0198	0.0197	0.0191	0.0099	0.0099	0.202

2759-001
A Single Dose Toxicity Study of Test Article Administered by Intrathecal Injection in Rats

Individual Organ Weight Values
Day 8

Sex: Female Day: 8 Relative to Start Date

Mid Dose	Sal gl mand/ sub., rt/BWt (%)	Sal gl mand/ sub.,rt/BrWt (ratio)	Spleen (g)	Spleen/BWt (%)	Spleen/BrWt (ratio)	Thymus (g)	Thymus/BWt (%)
3506	0.1054	0.1153	0.566	0.2775	0.3035	0.647	0.3172
3507	0.0881	0.0903	0.558	0.2891	0.2965	0.730	0.3782
3508	0.1065	0.1099	0.551	0.2978	0.3073	0.436	0.2357
3509	0.1005	0.1134	0.449	0.2303	0.2597	0.541	0.2774
3510	0.1052	0.1046	0.462	0.2406	0.2393	0.463	0.2411

2759-001
A Single Dose Toxicity Study of Test Article Administered by Intrathecal Injection in Rats

Individual Organ Weight Values
Day 8

Sex: Female Day: 8 Relative to Start Date

Mid Dose	Thymus/BrWt (ratio)	Thyroid/para (fixed) (g)	Thyroid/para (fixed)/BWt (%)	Thyroid/para (fixed)/BrWt (ratio)	Uterus w/ cervix (g)	Uterus w/ cervix/BWt (%)	Uterus w/ cervix/BrWt (ratio)
3506	0.3469	0.0257	0.0126	0.0138	0.377	0.1848	0.2021
3507	0.3879	0.0156	0.0081	0.0083	0.891	0.4617	0.4734
3508	0.2432	0.0280	0.0151	0.0156	0.493	0.2665	0.2750
3509	0.3129	0.0183	0.0094	0.0106	0.436	0.2236	0.2522
3510	0.2398	0.0180	0.0094	0.0093	0.414	0.2156	0.2144

2759-001
A Single Dose Toxicity Study of Test Article Administered by Intrathecal Injection in Rats

Individual Organ Weight Values
Day 8

Sex: Female Day: 8 Relative to Start Date

High Dose	Body Weight (g)	Brain (g)	Brain/BWt (%)	Adrenal glands (g)	Adrenal gl/ BWt (%)	Adrenal gl/ BrWt (ratio)	Heart (g)
4506	176	1.769	1.0051	0.059	0.0335	0.0334	1.066
4507	169	1.684	0.9964	0.055	0.0325	0.0327	0.798
4508	191	1.909	0.9995	0.054	0.0283	0.0283	0.886
4509	197	1.993	1.0117	0.081	0.0411	0.0406	1.096
4510	195	1.814	0.9303	0.075	0.0385	0.0413	0.866

2759-001
A Single Dose Toxicity Study of Test Article Administered by Intrathecal Injection in Rats

Individual Organ Weight Values
Day 8

Sex: Female Day: 8 Relative to Start Date

High Dose	Heart/BWt (%)	Heart/BrWt (ratio)	Kidneys (g)	Kidneys/BWt (%)	Kidneys/BrWt (ratio)	Liver (g)	Liver/BWt (%)
4506	0.6057	0.6026	1.883	1.0699	1.0644	7.049	4.0051
4507	0.4722	0.4739	1.714	1.0142	1.0178	6.973	4.1260
4508	0.4639	0.4641	1.861	0.9743	0.9749	8.175	4.2801
4509	0.5563	0.5499	1.967	0.9985	0.9870	8.645	4.3883
4510	0.4441	0.4774	2.021	1.0364	1.1141	8.681	4.4518

2759-001
A Single Dose Toxicity Study of Test Article Administered by Intrathecal Injection in Rats

Individual Organ Weight Values
Day 8

Sex: Female Day: 8 Relative to Start Date

High Dose	Liver/BrWt (ratio)	Lung (g)	Lung/BWt (%)	Lung/BrWt (ratio)	Ovaries (g)	Ovaries/BWt (%)	Ovaries/BrWt (ratio)
4506	3.9847	1.249	0.7097	0.7060	0.077	0.0438	0.0435
4507	4.1407	1.094	0.6473	0.6496	0.055	0.0325	0.0327
4508	4.2823	1.393	0.7293	0.7297	0.099	0.0518	0.0519
4509	4.3377	1.481	0.7518	0.7431	0.066	0.0335	0.0331
4510	4.7856	1.224	0.6277	0.6748	0.066	0.0338	0.0364

2759-001
A Single Dose Toxicity Study of Test Article Administered by Intrathecal Injection in Rats

Individual Organ Weight Values
Day 8

Sex: Female Day: 8 Relative to Start Date

High Dose	Oviducts (g)	Oviducts/BWt (%)	Oviducts/ BrWt (ratio)	Pituitary (fixed) (g)	Pituitary (fixed)/BWt (%)	Pituitary (fixed)/BrWt (ratio)	Sal, mand/ sub., rt (g)
4506	0.021	0.0119	0.0119	0.0151	0.0086	0.0085	0.176
4507	0.017	0.0101	0.0101	0.0142	0.0084	0.0084	0.142
4508	0.023	0.0120	0.0120	0.0150	0.0079	0.0079	0.184
4509	0.023	0.0117	0.0115	0.0209	0.0106	0.0105	0.212
4510	0.021	0.0108	0.0116	0.0214	0.0110	0.0118	0.227

2759-001
A Single Dose Toxicity Study of Test Article Administered by Intrathecal Injection in Rats

Individual Organ Weight Values
Day 8

Sex: Female Day: 8 Relative to Start Date

High Dose	Sal gl mand/ sub., rt/BWt (%)	Sal gl mand/ sub.,rt/BrWt (ratio)	Spleen (g)	Spleen/BWt (%)	Spleen/BrWt (ratio)	Thymus (g)	Thymus/BWt (%)
4506	0.1000	0.0995	0.519	0.2949	0.2934	0.576	0.3273
4507	0.0840	0.0843	0.423	0.2503	0.2512	0.432	0.2556
4508	0.0963	0.0964	0.434	0.2272	0.2273	0.442	0.2314
4509	0.1076	0.1064	0.499	0.2533	0.2504	0.703	0.3569
4510	0.1164	0.1251	0.429	0.2200	0.2365	0.642	0.3292

2759-001
A Single Dose Toxicity Study of Test Article Administered by Intrathecal Injection in Rats

Individual Organ Weight Values
Day 8

Sex: Female Day: 8 Relative to Start Date

High Dose	Thymus/BrWt (ratio)	Thyroid/para (fixed) (g)	Thyroid/para (fixed)/BWt (%)	Thyroid/para (fixed)/BrWt (ratio)	Uterus w/ cervix (g)	Uterus w/ cervix/BWt (%)	Uterus w/ cervix/BrWt (ratio)
4506	0.3256	0.0181	0.0103	0.0102	0.398	0.2261	0.2250
4507	0.2565	0.0190	0.0112	0.0113	0.392	0.2320	0.2328
4508	0.2315	0.0226	0.0118	0.0118	0.445	0.2330	0.2331
4509	0.3527	0.0235	0.0119	0.0118	0.427	0.2168	0.2142
4510	0.3539	0.0157	0.0081	0.0087	0.378	0.1938	0.2084

Appendix 3
Individual Organ Weight Values (Day 29)

2759-001
A Single Dose Toxicity Study of Test Article Administered by Intrathecal Injection in Rats

Individual Organ Weight Values
Day 29

Sex: Male Day: 29 Relative to Start Date

Vehicle	Body Weight (g)	Brain (g)	Brain/BWt (%)	Adrenal glands (g)	Adrenal gl/ BWt (%)	Adrenal gl/ BrWt (ratio)	Epididymides (g)
1011	443	2.155	0.4865	0.084	0.0190	0.0390	1.364
1012	440	2.169	0.4930	0.085	0.0193	0.0392	1.407
1013	428	2.034	0.4752	0.068	0.0159	0.0334	1.301
1014	420	2.090	0.4976	0.076	0.0181	0.0364	1.438
1015	393	1.958	0.4982	0.084	0.0214	0.0429	1.519

2759-001
A Single Dose Toxicity Study of Test Article Administered by Intrathecal Injection in Rats

Individual Organ Weight Values
Day 29

Sex: Male Day: 29 Relative to Start Date

Vehicle	Epididymides /BWt (%)	Epididymides /BrWt (ratio)	Heart (g)	Heart/BWt (%)	Heart/BrWt (ratio)	Kidneys (g)	Kidneys/BWt (%)
1011	0.3079	0.6329	1.477	0.3334	0.6854	3.968	0.8957
1012	0.3198	0.6487	1.850	0.4205	0.8529	3.771	0.8570
1013	0.3040	0.6396	1.491	0.3484	0.7330	3.320	0.7757
1014	0.3424	0.6880	1.519	0.3617	0.7268	3.505	0.8345
1015	0.3865	0.7758	1.628	0.4142	0.8315	3.885	0.9885

2759-001
A Single Dose Toxicity Study of Test Article Administered by Intrathecal Injection in Rats

Individual Organ Weight Values
Day 29

Sex: Male Day: 29 Relative to Start Date

Vehicle	Kidneys/BrWt (ratio)	Liver (g)	Liver/BWt (%)	Liver/BrWt (ratio)	Lung (g)	Lung/BWt (%)	Lung/BrWt (ratio)
1011	1.8413	17.065	3.8521	7.9188	1.629	0.3677	0.7559
1012	1.7386	17.975	4.0852	8.2872	2.141	0.4866	0.9871
1013	1.6323	16.862	3.9397	8.2901	1.651	0.3857	0.8117
1014	1.6770	16.359	3.8950	7.8273	1.607	0.3826	0.7689
1015	1.9842	16.646	4.2356	8.5015	1.898	0.4830	0.9694

2759-001
A Single Dose Toxicity Study of Test Article Administered by Intrathecal Injection in Rats

Individual Organ Weight Values
Day 29

Sex: Male Day: 29 Relative to Start Date

Vehicle	Pituitary (fixed) (g)	Pituitary (fixed)/BWt (%)	Pituitary (fixed)/BrWt (ratio)	Prostate gland (g)	Prostate gland/BWt (%)	Prostate gland/BrWt (ratio)	Sal, mand/ sub., rt (g)
1011	0.0171	0.0039	0.0079	1.295	0.2923	0.6009	0.354
1012	0.0188	0.0043	0.0087	1.034	0.2350	0.4767	0.403
1013	0.0169	0.0039	0.0083	0.966	0.2257	0.4749	0.372
1014	0.0201	0.0048	0.0096	1.193	0.2840	0.5708	0.418
1015	0.0184	0.0047	0.0094	1.445	0.3677	0.7380	0.434

2759-001
A Single Dose Toxicity Study of Test Article Administered by Intrathecal Injection in Rats

Individual Organ Weight Values
Day 29

Sex: Male Day: 29 Relative to Start Date

Vehicle	Sal gl mand/ sub., rt/BWt (%)	Sal gl mand/ sub.,rt/BrWt (ratio)	Spleen (g)	Spleen/BWt (%)	Spleen/BrWt (ratio)	Testes (g)	Testes/BWt (%)
1011	0.0799	0.1643	0.703	0.1587	0.3262	3.733	0.8427
1012	0.0916	0.1858	0.900	0.2045	0.4149	4.087	0.9289
1013	0.0869	0.1829	0.786	0.1836	0.3864	3.149	0.7357
1014	0.0995	0.2000	0.844	0.2010	0.4038	3.697	0.8802
1015	0.1104	0.2217	0.852	0.2168	0.4351	3.801	0.9672

2759-001
A Single Dose Toxicity Study of Test Article Administered by Intrathecal Injection in Rats

Individual Organ Weight Values
Day 29

Sex: Male Day: 29 Relative to Start Date

Vehicle	Testes/BrWt (ratio)	Thymus (g)	Thymus/BWt (%)	Thymus/BrWt (ratio)	Thyroid/para (fixed) (g)	Thyroid/para (fixed)/BWt (%)	Thyroid/para (fixed)/BrWt (ratio)
1011	1.7323	0.410	0.0926	0.1903	0.0352	0.0079	0.0163
1012	1.8843	0.668	0.1518	0.3080	0.0334	0.0076	0.0154
1013	1.5482	0.446	0.1042	0.2193	0.0360	0.0084	0.0177
1014	1.7689	0.381	0.0907	0.1823	0.0309	0.0074	0.0148
1015	1.9413	0.407	0.1036	0.2079	0.0377	0.0096	0.0193

2759-001
A Single Dose Toxicity Study of Test Article Administered by Intrathecal Injection in Rats

Individual Organ Weight Values
Day 29

Sex: Male Day: 29 Relative to Start Date

Low Dose	Body Weight (g)	Brain (g)	Brain/BWt (%)	Adrenal glands (g)	Adrenal gl/ BWt (%)	Adrenal gl/ BrWt (ratio)	Epididymides (g)
2011	429	1.952	0.4550	0.088	0.0205	0.0451	1.376
2012	433	2.039	0.4709	0.068	0.0157	0.0333	1.261
2013	438	2.147	0.4902	0.066	0.0151	0.0307	1.234
2014	398	1.930	0.4849	0.073	0.0183	0.0378	1.242
2015	444	2.258	0.5086	0.091	0.0205	0.0403	1.209

2759-001
A Single Dose Toxicity Study of Test Article Administered by Intrathecal Injection in Rats

Individual Organ Weight Values
Day 29

Sex: Male Day: 29 Relative to Start Date

Low Dose	Epididymides /BWt (%)	Epididymides /BrWt (ratio)	Heart (g)	Heart/BWt (%)	Heart/BrWt (ratio)	Kidneys (g)	Kidneys/BWt (%)
2011	0.3207	0.7049	1.593	0.3713	0.8161	3.623	0.8445
2012	0.2912	0.6184	1.664	0.3843	0.8161	3.627	0.8376
2013	0.2817	0.5748	1.964	0.4484	0.9148	4.394	1.0032
2014	0.3121	0.6435	1.663	0.4178	0.8617	3.113	0.7822
2015	0.2723	0.5354	1.496	0.3369	0.6625	3.488	0.7856

2759-001
A Single Dose Toxicity Study of Test Article Administered by Intrathecal Injection in Rats

Individual Organ Weight Values
Day 29

Sex: Male Day: 29 Relative to Start Date

Low Dose	Kidneys/BrWt (ratio)	Liver (g)	Liver/BWt (%)	Liver/BrWt (ratio)	Lung (g)	Lung/BWt (%)	Lung/BrWt (ratio)
2011	1.8560	16.499	3.8459	8.4524	1.433	0.3340	0.7341
2012	1.7788	16.010	3.6975	7.8519	1.549	0.3577	0.7597
2013	2.0466	18.300	4.1781	8.5235	1.682	0.3840	0.7834
2014	1.6130	13.412	3.3698	6.9492	1.826	0.4588	0.9461
2015	1.5447	15.080	3.3964	6.6785	1.886	0.4248	0.8353

2759-001
A Single Dose Toxicity Study of Test Article Administered by Intrathecal Injection in Rats

Individual Organ Weight Values
Day 29

Sex: Male Day: 29 Relative to Start Date

Low Dose	Pituitary (fixed) (g)	Pituitary (fixed)/BWt (%)	Pituitary (fixed)/BrWt (ratio)	Prostate gland (g)	Prostate gland/BWt (%)	Prostate gland/BrWt (ratio)	Sal, mand/ sub., rt (g)
2011	0.0156	0.0036	0.0080	1.336	0.3114	0.6844	0.307
2012	0.0178	0.0041	0.0087	1.136	0.2624	0.5571	0.337
2013	0.0213	0.0049	0.0099	1.167	0.2664	0.5435	0.396
2014	0.0157	0.0039	0.0081	1.443	0.3626	0.7477	0.390
2015	0.0222	0.0050	0.0098	1.299	0.2926	0.5753	0.415

2759-001
A Single Dose Toxicity Study of Test Article Administered by Intrathecal Injection in Rats

Individual Organ Weight Values
Day 29

Sex: Male Day: 29 Relative to Start Date

Low Dose	Sal gl mand/ sub., rt/BWt (%)	Sal gl mand/ sub.,rt/BrWt (ratio)	Spleen (g)	Spleen/BWt (%)	Spleen/BrWt (ratio)	Testes (g)	Testes/BWt (%)
2011	0.0716	0.1573	0.710	0.1655	0.3637	3.783	0.8818
2012	0.0778	0.1653	0.689	0.1591	0.3379	3.546	0.8189
2013	0.0904	0.1844	0.835	0.1906	0.3889	3.822	0.8726
2014	0.0980	0.2021	1.041	0.2616	0.5394	3.178	0.7985
2015	0.0935	0.1838	0.763	0.1718	0.3379	3.442	0.7752

2759-001
A Single Dose Toxicity Study of Test Article Administered by Intrathecal Injection in Rats

Individual Organ Weight Values
Day 29

Sex: Male Day: 29 Relative to Start Date

Low Dose	Testes/BrWt (ratio)	Thymus (g)	Thymus/BWt (%)	Thymus/BrWt (ratio)	Thyroid/para (fixed) (g)	Thyroid/para (fixed)/BWt (%)	Thyroid/para (fixed)/BrWt (ratio)
2011	1.9380	0.386	0.0900	0.1977	0.0275	0.0064	0.0141
2012	1.7391	0.641	0.1480	0.3144	0.0317	0.0073	0.0155
2013	1.7802	0.480	0.1096	0.2236	0.0292	0.0067	0.0136
2014	1.6466	0.563	0.1415	0.2917	0.0297	0.0075	0.0154
2015	1.5244	0.457	0.1029	0.2024	0.0348	0.0078	0.0154

2759-001
A Single Dose Toxicity Study of Test Article Administered by Intrathecal Injection in Rats

Individual Organ Weight Values
Day 29

Sex: Male Day: 29 Relative to Start Date

Mid Dose	Body Weight (g)	Brain (g)	Brain/BWt (%)	Adrenal glands (g)	Adrenal gl/ BWt (%)	Adrenal gl/ BrWt (ratio)	Epididymides (g)
3011	396	2.102	0.5308	0.086	0.0217	0.0409	1.335
3012	372	2.086	0.5608	0.066	0.0177	0.0316	1.212
3013	470	1.996	0.4247	0.063	0.0134	0.0316	1.337
3014	458	2.105	0.4596	0.063	0.0138	0.0299	1.231
3015	368	1.978	0.5375	0.061	0.0166	0.0308	1.183

2759-001
A Single Dose Toxicity Study of Test Article Administered by Intrathecal Injection in Rats

Individual Organ Weight Values
Day 29

Sex: Male Day: 29 Relative to Start Date

Mid Dose	Epididymides /BWt (%)	Epididymides /BrWt (ratio)	Heart (g)	Heart/BWt (%)	Heart/BrWt (ratio)	Kidneys (g)	Kidneys/BWt (%)
3011	0.3371	0.6351	1.592	0.4020	0.7574	3.321	0.8386
3012	0.3258	0.5810	1.380	0.3710	0.6616	2.981	0.8013
3013	0.2845	0.6698	1.857	0.3951	0.9304	4.372	0.9302
3014	0.2688	0.5848	1.486	0.3245	0.7059	3.481	0.7600
3015	0.3215	0.5981	1.316	0.3576	0.6653	3.124	0.8489

2759-001
A Single Dose Toxicity Study of Test Article Administered by Intrathecal Injection in Rats

Individual Organ Weight Values
Day 29

Sex: Male Day: 29 Relative to Start Date

Mid Dose	Kidneys/BrWt (ratio)	Liver (g)	Liver/BWt (%)	Liver/BrWt (ratio)	Lung (g)	Lung/BWt (%)	Lung/BrWt (ratio)
3011	1.5799	13.614	3.4379	6.4767	1.671	0.4220	0.7950
3012	1.4291	13.438	3.6124	6.4420	1.639	0.4406	0.7857
3013	2.1904	17.830	3.7936	8.9329	2.157	0.4589	1.0807
3014	1.6537	15.140	3.3057	7.1924	2.402	0.5245	1.1411
3015	1.5794	12.968	3.5239	6.5561	1.533	0.4166	0.7750

2759-001
A Single Dose Toxicity Study of Test Article Administered by Intrathecal Injection in Rats

Individual Organ Weight Values
Day 29

Sex: Male Day: 29 Relative to Start Date

Mid Dose	Pituitary (fixed) (g)	Pituitary (fixed)/BWt (%)	Pituitary (fixed)/BrWt (ratio)	Prostate gland (g)	Prostate gland/BWt (%)	Prostate gland/BrWt (ratio)	Sal, mand/ sub., rt (g)
3011	0.0142	0.0036	0.0068	1.382	0.3490	0.6575	0.318
3012	0.0167	0.0045	0.0080	0.904	0.2430	0.4334	0.322
3013	0.0202	0.0043	0.0101	1.403	0.2985	0.7029	0.387
3014	0.0166	0.0036	0.0079	1.247	0.2723	0.5924	0.328
3015	0.0147	0.0040	0.0074	1.552	0.4217	0.7846	0.323

2759-001
A Single Dose Toxicity Study of Test Article Administered by Intrathecal Injection in Rats

Individual Organ Weight Values
Day 29

Sex: Male Day: 29 Relative to Start Date

Mid Dose	Sal gl mand/ sub., rt/BWt (%)	Sal gl mand/ sub.,rt/BrWt (ratio)	Spleen (g)	Spleen/BWt (%)	Spleen/BrWt (ratio)	Testes (g)	Testes/BWt (%)
3011	0.0803	0.1513	0.908	0.2293	0.4320	3.983	1.0058
3012	0.0866	0.1544	1.045	0.2809	0.5010	3.310	0.8898
3013	0.0823	0.1939	1.527	0.3249	0.7650	3.855	0.8202
3014	0.0716	0.1558	0.893	0.1950	0.4242	3.522	0.7690
3015	0.0878	0.1633	0.892	0.2424	0.4510	3.754	1.0201

2759-001
A Single Dose Toxicity Study of Test Article Administered by Intrathecal Injection in Rats

Individual Organ Weight Values
Day 29

Sex: Male Day: 29 Relative to Start Date

Mid Dose	Testes/BrWt (ratio)	Thymus (g)	Thymus/BWt (%)	Thymus/BrWt (ratio)	Thyroid/para (fixed) (g)	Thyroid/para (fixed)/BWt (%)	Thyroid/para (fixed)/BrWt (ratio)
3011	1.8949	0.426	0.1076	0.2027	0.0298	0.0075	0.0142
3012	1.5868	0.488	0.1312	0.2339	0.0283	0.0076	0.0136
3013	1.9314	0.656	0.1396	0.3287	0.0292	0.0062	0.0146
3014	1.6732	0.910	0.1987	0.4323	0.0262	0.0057	0.0124
3015	1.8979	0.510	0.1386	0.2578	0.0208	0.0057	0.0105

2759-001
A Single Dose Toxicity Study of Test Article Administered by Intrathecal Injection in Rats

Individual Organ Weight Values
Day 29

Sex: Male Day: 29 Relative to Start Date

High Dose	Body Weight (g)	Brain (g)	Brain/BWt (%)	Adrenal glands (g)	Adrenal gl/ BWt (%)	Adrenal gl/ BrWt (ratio)	Epididymides (g)
4011	429	2.113	0.4925	0.083	0.0193	0.0393	1.291
4012	558	2.153	0.3858	0.100	0.0179	0.0464	1.423
4013	405	2.052	0.5067	0.062	0.0153	0.0302	1.168
4014	406	2.057	0.5067	0.073	0.0180	0.0355	1.287
4015	355	2.157	0.6076	0.065	0.0183	0.0301	1.323

2759-001
A Single Dose Toxicity Study of Test Article Administered by Intrathecal Injection in Rats

Individual Organ Weight Values
Day 29

Sex: Male Day: 29 Relative to Start Date

High Dose	Epididymides /BWt (%)	Epididymides /BrWt (ratio)	Heart (g)	Heart/BWt (%)	Heart/BrWt (ratio)	Kidneys (g)	Kidneys/BWt (%)
4011	0.3009	0.6110	2.023	0.4716	0.9574	3.606	0.8406
4012	0.2550	0.6609	2.302	0.4125	1.0692	4.201	0.7529
4013	0.2884	0.5692	1.592	0.3931	0.7758	3.243	0.8007
4014	0.3170	0.6257	1.602	0.3946	0.7788	3.917	0.9648
4015	0.3727	0.6134	1.831	0.5158	0.8489	3.354	0.9448

2759-001
A Single Dose Toxicity Study of Test Article Administered by Intrathecal Injection in Rats

Individual Organ Weight Values
Day 29

Sex: Male Day: 29 Relative to Start Date

High Dose	Kidneys/BrWt (ratio)	Liver (g)	Liver/BWt (%)	Liver/BrWt (ratio)	Lung (g)	Lung/BWt (%)	Lung/BrWt (ratio)
4011	1.7066	14.669	3.4193	6.9423	2.035	0.4744	0.9631
4012	1.9512	21.576	3.8667	10.0214	2.183	0.3912	1.0139
4013	1.5804	14.210	3.5086	6.9250	2.226	0.5496	1.0848
4014	1.9042	14.939	3.6796	7.2625	1.610	0.3966	0.7827
4015	1.5549	12.439	3.5039	5.7668	2.555	0.7197	1.1845

2759-001
A Single Dose Toxicity Study of Test Article Administered by Intrathecal Injection in Rats

Individual Organ Weight Values
Day 29

Sex: Male Day: 29 Relative to Start Date

High Dose	Pituitary (fixed) (g)	Pituitary (fixed)/BWt (%)	Pituitary (fixed)/BrWt (ratio)	Prostate gland (g)	Prostate gland/BWt (%)	Prostate gland/BrWt (ratio)	Sal, mand/ sub., rt (g)
4011	0.0177	0.0041	0.0084	1.410	0.3287	0.6673	0.425
4012	0.0152	0.0027	0.0071	1.237	0.2217	0.5745	0.342
4013	0.0150	0.0037	0.0073	1.144	0.2825	0.5575	0.315
4014	0.0175	0.0043	0.0085	1.260	0.3103	0.6125	0.372
4015	0.0136	0.0038	0.0063	1.234	0.3476	0.5721	0.324

2759-001
A Single Dose Toxicity Study of Test Article Administered by Intrathecal Injection in Rats

Individual Organ Weight Values
Day 29

Sex: Male Day: 29 Relative to Start Date

High Dose	Sal gl mand/ sub., rt/BWt (%)	Sal gl mand/ sub.,rt/BrWt (ratio)	Spleen (g)	Spleen/BWt (%)	Spleen/BrWt (ratio)	Testes (g)	Testes/BWt (%)
4011	0.0991	0.2011	0.872	0.2033	0.4127	3.547	0.8268
4012	0.0613	0.1588	1.092	0.1957	0.5072	3.914	0.7014
4013	0.0778	0.1535	0.754	0.1862	0.3674	3.712	0.9165
4014	0.0916	0.1808	0.972	0.2394	0.4725	3.873	0.9539
4015	0.0913	0.1502	0.842	0.2372	0.3904	3.405	0.9592

2759-001
A Single Dose Toxicity Study of Test Article Administered by Intrathecal Injection in Rats

Individual Organ Weight Values
Day 29

Sex: Male Day: 29 Relative to Start Date

High Dose	Testes/BrWt (ratio)	Thymus (g)	Thymus/BWt (%)	Thymus/BrWt (ratio)	Thyroid/para (fixed) (g)	Thyroid/para (fixed)/BWt (%)	Thyroid/para (fixed)/BrWt (ratio)
4011	1.6787	0.445	0.1037	0.2106	0.0267	0.0062	0.0126
4012	1.8179	0.676	0.1211	0.3140	0.0321	0.0058	0.0149
4013	1.8090	0.534	0.1319	0.2602	0.0260	0.0064	0.0127
4014	1.8828	0.641	0.1579	0.3116	0.0276	0.0068	0.0134
4015	1.5786	0.593	0.1670	0.2749	0.0222	0.0063	0.0103

2759-001
A Single Dose Toxicity Study of Test Article Administered by Intrathecal Injection in Rats

Individual Organ Weight Values
Day 29

Sex: Female Day: 29 Relative to Start Date

Vehicle	Body Weight (g)	Brain (g)	Brain/BWt (%)	Adrenal glands (g)	Adrenal gl/ BWt (%)	Adrenal gl/ BrWt (ratio)	Heart (g)
1511	251	2.132	0.8494	0.060	0.0239	0.0281	0.874
1512	251	1.921	0.7653	0.090	0.0359	0.0469	0.812
1513	220	1.914	0.8700	0.076	0.0345	0.0397	0.921
1514	251	1.972	0.7857	0.066	0.0263	0.0335	0.860
1515	222	1.908	0.8595	0.080	0.0360	0.0419	1.081

2759-001
A Single Dose Toxicity Study of Test Article Administered by Intrathecal Injection in Rats

Individual Organ Weight Values
Day 29

Sex: Female Day: 29 Relative to Start Date

Vehicle	Heart/BWt (%)	Heart/BrWt (ratio)	Kidneys (g)	Kidneys/BWt (%)	Kidneys/BrWt (ratio)	Liver (g)	Liver/BWt (%)
1511	0.3482	0.4099	1.919	0.7645	0.9001	9.071	3.6139
1512	0.3235	0.4227	1.956	0.7793	1.0182	8.577	3.4171
1513	0.4186	0.4812	1.874	0.8518	0.9791	8.817	4.0077
1514	0.3426	0.4361	1.785	0.7112	0.9052	8.057	3.2100
1515	0.4869	0.5666	1.641	0.7392	0.8601	7.822	3.5234

2759-001
A Single Dose Toxicity Study of Test Article Administered by Intrathecal Injection in Rats

Individual Organ Weight Values
Day 29

Sex: Female Day: 29 Relative to Start Date

Vehicle	Liver/BrWt (ratio)	Lung (g)	Lung/BWt (%)	Lung/BrWt (ratio)	Ovaries (g)	Ovaries/BWt (%)	Ovaries/BrWt (ratio)
1511	4.2547	1.973	0.7861	0.9254	0.082	0.0327	0.0385
1512	4.4649	1.726	0.6876	0.8985	0.055	0.0219	0.0286
1513	4.6066	1.166	0.5300	0.6092	0.101	0.0459	0.0528
1514	4.0857	1.194	0.4757	0.6055	0.089	0.0355	0.0451
1515	4.0996	1.361	0.6131	0.7133	0.095	0.0428	0.0498

2759-001
A Single Dose Toxicity Study of Test Article Administered by Intrathecal Injection in Rats

Individual Organ Weight Values
Day 29

Sex: Female Day: 29 Relative to Start Date

Vehicle	Oviducts (g)	Oviducts/BWt (%)	Oviducts/ BrWt (ratio)	Pituitary (fixed) (g)	Pituitary (fixed)/BWt (%)	Pituitary (fixed)/BrWt (ratio)	Sal, mand/ sub., rt (g)
1511	0.022	0.0088	0.0103	0.0175	0.0070	0.0082	0.237
1512	0.022	0.0088	0.0115	0.0153	0.0061	0.0080	0.308
1513	0.017	0.0077	0.0089	0.0206	0.0094	0.0108	0.228
1514	0.019	0.0076	0.0096	0.0185	0.0074	0.0094	0.179
1515	0.049	0.0221	0.0257	0.0195	0.0088	0.0102	0.187

2759-001
A Single Dose Toxicity Study of Test Article Administered by Intrathecal Injection in Rats

Individual Organ Weight Values
Day 29

Sex: Female Day: 29 Relative to Start Date

Vehicle	Sal gl mand/ sub., rt/BWt (%)	Sal gl mand/ sub.,rt/BrWt (ratio)	Spleen (g)	Spleen/BWt (%)	Spleen/BrWt (ratio)	Thymus (g)	Thymus/BWt (%)
1511	0.0944	0.1112	0.507	0.2020	0.2378	0.478	0.1904
1512	0.1227	0.1603	0.627	0.2498	0.3264	0.523	0.2084
1513	0.1036	0.1191	0.576	0.2618	0.3009	0.302	0.1373
1514	0.0713	0.0908	0.431	0.1717	0.2186	0.495	0.1972
1515	0.0842	0.0980	0.542	0.2441	0.2841	0.458	0.2063

2759-001
A Single Dose Toxicity Study of Test Article Administered by Intrathecal Injection in Rats

Individual Organ Weight Values
Day 29

Sex: Female Day: 29 Relative to Start Date

Vehicle	Thymus/BrWt (ratio)	Thyroid/para (fixed) (g)	Thyroid/para (fixed)/BWt (%)	Thyroid/para (fixed)/BrWt (ratio)	Uterus w/ cervix (g)	Uterus w/ cervix/BWt (%)	Uterus w/ cervix/BrWt (ratio)
1511	0.2242	0.0245	0.0098	0.0115	0.503	0.2004	0.2359
1512	0.2723	0.0238	0.0095	0.0124	0.622	0.2478	0.3238
1513	0.1578	0.0195	0.0089	0.0102	0.488	0.2218	0.2550
1514	0.2510	0.0174	0.0069	0.0088	0.434	0.1729	0.2201
1515	0.2400	0.0163	0.0073	0.0085	0.660	0.2973	0.3459

2759-001
A Single Dose Toxicity Study of Test Article Administered by Intrathecal Injection in Rats

Individual Organ Weight Values
Day 29

Sex: Female Day: 29 Relative to Start Date

Low Dose	Body Weight (g)	Brain (g)	Brain/BWt (%)	Adrenal glands (g)	Adrenal gl/ BWt (%)	Adrenal gl/ BrWt (ratio)	Heart (g)
2511	212	1.910	0.9009	0.062	0.0292	0.0325	0.879
2512	241	1.975	0.8195	0.058	0.0241	0.0294	0.826
2513	195	1.832	0.9395	0.066	0.0338	0.0360	0.780
2514	219	1.847	0.8434	0.071	0.0324	0.0384	0.962
2515	210	1.807	0.8605	0.093	0.0443	0.0515	0.777

2759-001
A Single Dose Toxicity Study of Test Article Administered by Intrathecal Injection in Rats

Individual Organ Weight Values
Day 29

Sex: Female Day: 29 Relative to Start Date

Low Dose	Heart/BWt (%)	Heart/BrWt (ratio)	Kidneys (g)	Kidneys/BWt (%)	Kidneys/BrWt (ratio)	Liver (g)	Liver/BWt (%)
2511	0.4146	0.4602	1.825	0.8608	0.9555	8.738	4.1217
2512	0.3427	0.4182	1.996	0.8282	1.0106	8.629	3.5805
2513	0.4000	0.4258	1.622	0.8318	0.8854	7.251	3.7185
2514	0.4393	0.5208	1.853	0.8461	1.0032	8.105	3.7009
2515	0.3700	0.4300	2.017	0.9605	1.1162	9.107	4.3367

2759-001
A Single Dose Toxicity Study of Test Article Administered by Intrathecal Injection in Rats

Individual Organ Weight Values
Day 29

Sex: Female Day: 29 Relative to Start Date

Low Dose	Liver/BrWt (ratio)	Lung (g)	Lung/BWt (%)	Lung/BrWt (ratio)	Ovaries (g)	Ovaries/BWt (%)	Ovaries/BrWt (ratio)
2511	4.5749	1.172	0.5528	0.6136	0.077	0.0363	0.0403
2512	4.3691	1.423	0.5905	0.7205	0.055	0.0228	0.0278
2513	3.9580	1.145	0.5872	0.6250	0.077	0.0395	0.0420
2514	4.3882	1.238	0.5653	0.6703	0.098	0.0447	0.0531
2515	5.0398	1.228	0.5848	0.6796	0.088	0.0419	0.0487

2759-001
A Single Dose Toxicity Study of Test Article Administered by Intrathecal Injection in Rats

Individual Organ Weight Values
Day 29

Sex: Female Day: 29 Relative to Start Date

Low Dose	Oviducts (g)	Oviducts/BWt (%)	Oviducts/ BrWt (ratio)	Pituitary (fixed) (g)	Pituitary (fixed)/BWt (%)	Pituitary (fixed)/BrWt (ratio)	Sal, mand/ sub., rt (g)
2511	0.025	0.0118	0.0131	0.0219	0.0103	0.0115	0.187
2512	0.021	0.0087	0.0106	0.0184	0.0076	0.0093	0.169
2513	0.029	0.0149	0.0158	0.0189	0.0097	0.0103	0.200
2514	0.032	0.0146	0.0173	0.0212	0.0097	0.0115	0.213
2515	0.031	0.0148	0.0172	0.0195	0.0093	0.0108	0.251

2759-001
A Single Dose Toxicity Study of Test Article Administered by Intrathecal Injection in Rats

Individual Organ Weight Values
Day 29

Sex: Female Day: 29 Relative to Start Date

Low Dose	Sal gl mand/ sub., rt/BWt (%)	Sal gl mand/ sub.,rt/BrWt (ratio)	Spleen (g)	Spleen/BWt (%)	Spleen/BrWt (ratio)	Thymus (g)	Thymus/BWt (%)
2511	0.0882	0.0979	0.479	0.2259	0.2508	0.277	0.1307
2512	0.0701	0.0856	0.467	0.1938	0.2365	0.404	0.1676
2513	0.1026	0.1092	0.464	0.2379	0.2533	0.423	0.2169
2514	0.0973	0.1153	0.575	0.2626	0.3113	0.441	0.2014
2515	0.1195	0.1389	0.544	0.2590	0.3011	0.298	0.1419

2759-001
A Single Dose Toxicity Study of Test Article Administered by Intrathecal Injection in Rats

Individual Organ Weight Values
Day 29

Sex: Female Day: 29 Relative to Start Date

Low Dose	Thymus/BrWt (ratio)	Thyroid/para (fixed) (g)	Thyroid/para (fixed)/BWt (%)	Thyroid/para (fixed)/BrWt (ratio)	Uterus w/ cervix (g)	Uterus w/ cervix/BWt (%)	Uterus w/ cervix/BrWt (ratio)
2511	0.1450	0.0208	0.0098	0.0109	0.430	0.2028	0.2251
2512	0.2046	0.0163	0.0068	0.0083	0.678	0.2813	0.3433
2513	0.2309	0.0218	0.0112	0.0119	0.620	0.3179	0.3384
2514	0.2388	0.0197	0.0090	0.0107	0.555	0.2534	0.3005
2515	0.1649	0.0232	0.0110	0.0128	0.571	0.2719	0.3160

2759-001
A Single Dose Toxicity Study of Test Article Administered by Intrathecal Injection in Rats

Individual Organ Weight Values
Day 29

Sex: Female Day: 29 Relative to Start Date

Mid Dose	Body Weight (g)	Brain (g)	Brain/BWt (%)	Adrenal glands (g)	Adrenal gl/ BWt (%)	Adrenal gl/ BrWt (ratio)	Heart (g)
3511	230	1.905	0.8283	0.055	0.0239	0.0289	0.904
3512	189	1.721	0.9106	0.066	0.0349	0.0383	0.766
3513	226	1.885	0.8341	0.084	0.0372	0.0446	0.969
3514	196	1.807	0.9219	0.066	0.0337	0.0365	0.806
3515	244	2.045	0.8381	0.067	0.0275	0.0328	1.015

2759-001
A Single Dose Toxicity Study of Test Article Administered by Intrathecal Injection in Rats

Individual Organ Weight Values
Day 29

Sex: Female Day: 29 Relative to Start Date

Mid Dose	Heart/BWt (%)	Heart/BrWt (ratio)	Kidneys (g)	Kidneys/BWt (%)	Kidneys/BrWt (ratio)	Liver (g)	Liver/BWt (%)
3511	0.3930	0.4745	1.985	0.8630	1.0420	8.192	3.5617
3512	0.4053	0.4451	1.534	0.8116	0.8913	7.299	3.8619
3513	0.4288	0.5141	1.983	0.8774	1.0520	9.872	4.3681
3514	0.4112	0.4460	1.489	0.7597	0.8240	7.095	3.6199
3515	0.4160	0.4963	2.055	0.8422	1.0049	9.364	3.8377

2759-001
A Single Dose Toxicity Study of Test Article Administered by Intrathecal Injection in Rats

Individual Organ Weight Values
Day 29

Sex: Female Day: 29 Relative to Start Date

Mid Dose	Liver/BrWt (ratio)	Lung (g)	Lung/BWt (%)	Lung/BrWt (ratio)	Ovaries (g)	Ovaries/BWt (%)	Ovaries/BrWt (ratio)
3511	4.3003	1.201	0.5222	0.6304	0.086	0.0374	0.0451
3512	4.2411	1.346	0.7122	0.7821	0.063	0.0333	0.0366
3513	5.2371	1.074	0.4752	0.5698	0.049	0.0217	0.0260
3514	3.9264	0.974	0.4969	0.5390	0.050	0.0255	0.0277
3515	4.5790	1.461	0.5988	0.7144	0.098	0.0402	0.0479

2759-001
A Single Dose Toxicity Study of Test Article Administered by Intrathecal Injection in Rats

Individual Organ Weight Values
Day 29

Sex: Female Day: 29 Relative to Start Date

Mid Dose	Oviducts (g)	Oviducts/BWt (%)	Oviducts/BrWt (ratio)	Pituitary (fixed) (g)	Pituitary (fixed)/BWt (%)	Pituitary (fixed)/BrWt (ratio)	Sal, mand/ sub., rt (g)
3511	0.022	0.0096	0.0115	0.0142	0.0062	0.0075	0.186
3512	0.030	0.0159	0.0174	0.0125	0.0066	0.0073	0.173
3513	0.033	0.0146	0.0175	0.0167	0.0074	0.0089	0.247
3514	0.022	0.0112	0.0122	0.0223	0.0114	0.0123	0.155
3515	0.030	0.0123	0.0147	0.0161	0.0066	0.0079	0.103

2759-001
A Single Dose Toxicity Study of Test Article Administered by Intrathecal Injection in Rats

Individual Organ Weight Values
Day 29

Sex: Female Day: 29 Relative to Start Date

Mid Dose	Sal gl mand/ sub., rt/BWt (%)	Sal gl mand/ sub.,rt/BrWt (ratio)	Spleen (g)	Spleen/BWt (%)	Spleen/BrWt (ratio)	Thymus (g)	Thymus/BWt (%)
3511	0.0809	0.0976	0.776	0.3374	0.4073	0.508	0.2209
3512	0.0915	0.1005	0.424	0.2243	0.2464	0.308	0.1630
3513	0.1093	0.1310	0.569	0.2518	0.3019	0.324	0.1434
3514	0.0791	0.0858	0.385	0.1964	0.2131	0.304	0.1551
3515	0.0422	0.0504	0.629	0.2578	0.3076	0.294	0.1205

2759-001
A Single Dose Toxicity Study of Test Article Administered by Intrathecal Injection in Rats

Individual Organ Weight Values
Day 29

Sex: Female Day: 29 Relative to Start Date

Mid Dose	Thymus/BrWt (ratio)	Thyroid/para (fixed) (g)	Thyroid/para (fixed)/BWt (%)	Thyroid/para (fixed)/BrWt (ratio)	Uterus w/ cervix (g)	Uterus w/ cervix/BWt (%)	Uterus w/ cervix/BrWt (ratio)
3511	0.2667	0.0234	0.0102	0.0123	0.597	0.2596	0.3134
3512	0.1790	0.0208	0.0110	0.0121	0.817	0.4323	0.4747
3513	0.1719	0.0183	0.0081	0.0097	0.421	0.1863	0.2233
3514	0.1682	0.0229	0.0117	0.0127	0.434	0.2214	0.2402
3515	0.1438	0.0208	0.0085	0.0102	0.611	0.2504	0.2988

2759-001
A Single Dose Toxicity Study of Test Article Administered by Intrathecal Injection in Rats

Individual Organ Weight Values
Day 29

Sex: Female Day: 29 Relative to Start Date

High Dose	Body Weight (g)	Brain (g)	Brain/BWt (%)	Adrenal glands (g)	Adrenal gl/ BWt (%)	Adrenal gl/ BrWt (ratio)	Heart (g)
4511	240	1.999	0.8329	0.121	0.0504	0.0605	1.199
4512	229	1.821	0.7952	0.103	0.0450	0.0566	1.023
4513	204	1.890	0.9265	0.082	0.0402	0.0434	0.959
4514	248	1.901	0.7665	0.087	0.0351	0.0458	1.036
4515	189	1.917	1.0143	0.065	0.0344	0.0339	0.804

2759-001
A Single Dose Toxicity Study of Test Article Administered by Intrathecal Injection in Rats

Individual Organ Weight Values
Day 29

Sex: Female Day: 29 Relative to Start Date

High Dose	Heart/BWt (%)	Heart/BrWt (ratio)	Kidneys (g)	Kidneys/BWt (%)	Kidneys/BrWt (ratio)	Liver (g)	Liver/BWt (%)
4511	0.4996	0.5998	2.124	0.8850	1.0625	9.428	3.9283
4512	0.4467	0.5618	2.011	0.8782	1.1043	9.409	4.1087
4513	0.4701	0.5074	1.691	0.8289	0.8947	6.505	3.1887
4514	0.4177	0.5450	2.025	0.8165	1.0652	10.155	4.0948
4515	0.4254	0.4194	1.678	0.8878	0.8753	6.943	3.6735

2759-001
A Single Dose Toxicity Study of Test Article Administered by Intrathecal Injection in Rats

Individual Organ Weight Values
Day 29

Sex: Female Day: 29 Relative to Start Date

High Dose	Liver/BrWt (ratio)	Lung (g)	Lung/BWt (%)	Lung/BrWt (ratio)	Ovaries (g)	Ovaries/BWt (%)	Ovaries/BrWt (ratio)
4511	4.7164	1.266	0.5275	0.6333	0.101	0.0421	0.0505
4512	5.1669	1.206	0.5266	0.6623	0.042	0.0183	0.0231
4513	3.4418	1.232	0.6039	0.6519	0.109	0.0534	0.0577
4514	5.3419	1.291	0.5206	0.6791	0.137	0.0552	0.0721
4515	3.6218	1.121	0.5931	0.5848	0.070	0.0370	0.0365

2759-001
A Single Dose Toxicity Study of Test Article Administered by Intrathecal Injection in Rats

Individual Organ Weight Values
Day 29

Sex: Female Day: 29 Relative to Start Date

High Dose	Oviducts (g)	Oviducts/BWt (%)	Oviducts/ BrWt (ratio)	Pituitary (fixed) (g)	Pituitary (fixed)/BWt (%)	Pituitary (fixed)/BrWt (ratio)	Sal, mand/ sub., rt (g)
4511	0.029	0.0121	0.0145	0.0230	0.0096	0.0115	0.227
4512	0.031	0.0135	0.0170	0.0183	0.0080	0.0100	0.216
4513	0.026	0.0127	0.0138	0.0146	0.0072	0.0077	0.222
4514	0.036	0.0145	0.0189	0.0181	0.0073	0.0095	0.221
4515	0.020	0.0106	0.0104	0.0146	0.0077	0.0076	0.075

2759-001
A Single Dose Toxicity Study of Test Article Administered by Intrathecal Injection in Rats

Individual Organ Weight Values
Day 29

Sex: Female Day: 29 Relative to Start Date

High Dose	Sal gl mand/ sub., rt/BWt (%)	Sal gl mand/ sub.,rt/BrWt (ratio)	Spleen (g)	Spleen/BWt (%)	Spleen/BrWt (ratio)	Thymus (g)	Thymus/BWt (%)
4511	0.0946	0.1136	0.680	0.2833	0.3402	0.357	0.1488
4512	0.0943	0.1186	0.545	0.2380	0.2993	0.475	0.2074
4513	0.1088	0.1175	0.648	0.3176	0.3429	0.354	0.1735
4514	0.0891	0.1163	0.742	0.2992	0.3903	0.752	0.3032
4515	0.0397	0.0391	0.550	0.2910	0.2869	0.297	0.1571

2759-001
A Single Dose Toxicity Study of Test Article Administered by Intrathecal Injection in Rats

Individual Organ Weight Values
Day 29

Sex: Female Day: 29 Relative to Start Date

High Dose	Thymus/BrWt (ratio)	Thyroid/para (fixed) (g)	Thyroid/para (fixed)/BWt (%)	Thyroid/para (fixed)/BrWt (ratio)	Uterus w/ cervix (g)	Uterus w/ cervix/BWt (%)	Uterus w/ cervix/BrWt (ratio)
4511	0.1786	0.0207	0.0086	0.0104	0.541	0.2254	0.2706
4512	0.2608	0.0198	0.0086	0.0109	0.481	0.2100	0.2641
4513	0.1873	0.0186	0.0091	0.0098	0.480	0.2353	0.2540
4514	0.3956	0.0225	0.0091	0.0118	0.630	0.2540	0.3314
4515	0.1549	0.0168	0.0089	0.0088	0.565	0.2989	0.2947

Appendix 4
Individual Organ Weight Values (Day 91)

2759-001
A Single Dose Toxicity Study of Test Article Administered by Intrathecal Injection in Rats

Individual Organ Weight Values
Day 91

Sex: Male Day: 91 Relative to Start Date

Vehicle	Body Weight (g)	Brain (g)	Brain/BWt (%)	Adrenal glands (g)	Adrenal gl/ BWt (%)	Adrenal gl/ BrWt (ratio)	Epididymides (g)
1001	482	2.147	0.4454	0.074	0.0154	0.0345	1.619
1002	534	1.988	0.3723	0.092	0.0172	0.0463	1.798
1003	518	2.275	0.4392	0.058	0.0112	0.0255	1.347
1004	494	2.318	0.4692	0.050	0.0101	0.0216	1.493
1005	513	2.138	0.4168	0.068	0.0133	0.0318	1.484

2759-001
A Single Dose Toxicity Study of Test Article Administered by Intrathecal Injection in Rats

Individual Organ Weight Values
Day 91

Sex: Male Day: 91 Relative to Start Date

Vehicle	Epididymides /BWt (%)	Epididymides /BrWt (ratio)	Heart (g)	Heart/BWt (%)	Heart/BrWt (ratio)	Kidneys (g)	Kidneys/BWt (%)
1001	0.3359	0.7541	1.827	0.3790	0.8510	3.629	0.7529
1002	0.3367	0.9044	1.791	0.3354	0.9009	3.810	0.7135
1003	0.2600	0.5921	1.671	0.3226	0.7345	3.962	0.7649
1004	0.3022	0.6441	1.524	0.3085	0.6575	3.870	0.7834
1005	0.2893	0.6941	1.571	0.3062	0.7348	3.956	0.7712

2759-001
A Single Dose Toxicity Study of Test Article Administered by Intrathecal Injection in Rats

Individual Organ Weight Values
Day 91

Sex: Male Day: 91 Relative to Start Date

Vehicle	Kidneys/BrWt (ratio)	Liver (g)	Liver/BWt (%)	Liver/BrWt (ratio)	Lung (g)	Lung/BWt (%)	Lung/BrWt (ratio)
1001	1.6903	14.808	3.0722	6.8971	1.793	0.3720	0.8351
1002	1.9165	15.864	2.9708	7.9799	1.793	0.3358	0.9019
1003	1.7415	15.218	2.9378	6.6892	1.816	0.3506	0.7982
1004	1.6695	14.847	3.0055	6.4051	1.834	0.3713	0.7912
1005	1.8503	15.458	3.0133	7.2301	2.093	0.4080	0.9790

2759-001
A Single Dose Toxicity Study of Test Article Administered by Intrathecal Injection in Rats

Individual Organ Weight Values
Day 91

Sex: Male Day: 91 Relative to Start Date

Vehicle	Pituitary (fixed) (g)	Pituitary (fixed)/BWt (%)	Pituitary (fixed)/BrWt (ratio)	Prostate gland (g)	Prostate gland/BWt (%)	Prostate gland/BrWt (ratio)	Sal, mand/ sub., rt (g)
1001	0.0186	0.0039	0.0087	1.806	0.3747	0.8412	0.379
1002	0.0161	0.0030	0.0081	1.450	0.2715	0.7294	0.367
1003	0.0186	0.0036	0.0082	1.404	0.2710	0.6171	0.327
1004	0.0179	0.0036	0.0077	1.947	0.3941	0.8399	0.357
1005	0.0189	0.0037	0.0088	1.785	0.3480	0.8349	0.353

2759-001
A Single Dose Toxicity Study of Test Article Administered by Intrathecal Injection in Rats

Individual Organ Weight Values
Day 91

Sex: Male Day: 91 Relative to Start Date

Vehicle	Sal gl mand/ sub., rt/BWt (%)	Sal gl mand/ sub.,rt/BrWt (ratio)	Spleen (g)	Spleen/BWt (%)	Spleen/BrWt (ratio)	Testes (g)	Testes/BWt (%)
1001	0.0786	0.1765	0.814	0.1689	0.3791	3.734	0.7747
1002	0.0687	0.1846	0.879	0.1646	0.4422	3.631	0.6800
1003	0.0631	0.1437	0.663	0.1280	0.2914	3.745	0.7230
1004	0.0723	0.1540	0.835	0.1690	0.3602	3.716	0.7522
1005	0.0688	0.1651	1.099	0.2142	0.5140	3.659	0.7133

2759-001
A Single Dose Toxicity Study of Test Article Administered by Intrathecal Injection in Rats

Individual Organ Weight Values
Day 91

Sex: Male Day: 91 Relative to Start Date

Vehicle	Testes/BrWt (ratio)	Thymus (g)	Thymus/BWt (%)	Thymus/BrWt (ratio)	Thyroid/para (fixed) (g)	Thyroid/para (fixed)/BWt (%)	Thyroid/para (fixed)/BrWt (ratio)
1001	1.7392	0.203	0.0421	0.0946	0.0311	0.0065	0.0145
1002	1.8265	0.183	0.0343	0.0921	0.0294	0.0055	0.0148
1003	1.6462	0.257	0.0496	0.1130	0.0384	0.0074	0.0169
1004	1.6031	0.308	0.0623	0.1329	0.0247	0.0050	0.0107
1005	1.7114	0.272	0.0530	0.1272	0.0180	0.0035	0.0084

2759-001
A Single Dose Toxicity Study of Test Article Administered by Intrathecal Injection in Rats

Individual Organ Weight Values
Day 91

Sex: Male Day: 91 Relative to Start Date

Low Dose	Body Weight (g)	Brain (g)	Brain/BWt (%)	Adrenal glands (g)	Adrenal gl/ BWt (%)	Adrenal gl/ BrWt (ratio)	Epididymides (g)
2001	484	2.117	0.4374	0.053	0.0110	0.0250	1.617
2002	568	2.064	0.3634	0.087	0.0153	0.0422	1.514
2003	436	2.076	0.4761	0.050	0.0115	0.0241	1.530
2004	548	2.220	0.4051	0.072	0.0131	0.0324	1.643
2005	466	2.237	0.4800	0.060	0.0129	0.0268	1.524

2759-001
A Single Dose Toxicity Study of Test Article Administered by Intrathecal Injection in Rats

Individual Organ Weight Values
Day 91

Sex: Male Day: 91 Relative to Start Date

Low Dose	Epididymides /BWt (%)	Epididymides /BrWt (ratio)	Heart (g)	Heart/BWt (%)	Heart/BrWt (ratio)	Kidneys (g)	Kidneys/BWt (%)
2001	0.3341	0.7638	1.504	0.3107	0.7104	3.285	0.6787
2002	0.2665	0.7335	2.164	0.3810	1.0484	4.195	0.7386
2003	0.3509	0.7370	1.847	0.4236	0.8897	3.007	0.6897
2004	0.2998	0.7401	2.015	0.3677	0.9077	4.089	0.7462
2005	0.3270	0.6813	1.777	0.3813	0.7944	3.483	0.7474

2759-001
A Single Dose Toxicity Study of Test Article Administered by Intrathecal Injection in Rats

Individual Organ Weight Values
Day 91

Sex: Male Day: 91 Relative to Start Date

Low Dose	Kidneys/BrWt (ratio)	Liver (g)	Liver/BWt (%)	Liver/BrWt (ratio)	Lung (g)	Lung/BWt (%)	Lung/BrWt (ratio)
2001	1.5517	14.626	3.0219	6.9088	1.991	0.4114	0.9405
2002	2.0325	19.161	3.3734	9.2834	2.459	0.4329	1.1914
2003	1.4485	12.403	2.8447	5.9745	1.723	0.3952	0.8300
2004	1.8419	17.337	3.1637	7.8095	2.138	0.3901	0.9631
2005	1.5570	13.035	2.7972	5.8270	2.219	0.4762	0.9920

2759-001
A Single Dose Toxicity Study of Test Article Administered by Intrathecal Injection in Rats

Individual Organ Weight Values
Day 91

Sex: Male Day: 91 Relative to Start Date

Low Dose	Pituitary (fixed) (g)	Pituitary (fixed)/BWt (%)	Pituitary (fixed)/BrWt (ratio)	Prostate gland (g)	Prostate gland/BWt (%)	Prostate gland/BrWt (ratio)	Sal, mand/ sub., rt (g)
2001	0.0168	0.0035	0.0079	1.448	0.2992	0.6840	0.314
2002	0.0191	0.0034	0.0093	1.225	0.2157	0.5935	0.388
2003	0.0127	0.0029	0.0061	1.143	0.2622	0.5506	0.344
2004	0.0170	0.0031	0.0077	1.569	0.2863	0.7068	0.370
2005	0.0171	0.0037	0.0076	1.587	0.3406	0.7094	0.372

2759-001
A Single Dose Toxicity Study of Test Article Administered by Intrathecal Injection in Rats

Individual Organ Weight Values
Day 91

Sex: Male Day: 91 Relative to Start Date

Low Dose	Sal gl mand/ sub., rt/BWt (%)	Sal gl mand/ sub.,rt/BrWt (ratio)	Spleen (g)	Spleen/BWt (%)	Spleen/BrWt (ratio)	Testes (g)	Testes/BWt (%)
2001	0.0649	0.1483	0.872	0.1802	0.4119	3.682	0.7607
2002	0.0683	0.1880	0.903	0.1590	0.4375	3.595	0.6329
2003	0.0789	0.1657	0.740	0.1697	0.3565	3.774	0.8656
2004	0.0675	0.1667	0.894	0.1631	0.4027	3.759	0.6859
2005	0.0798	0.1663	0.754	0.1618	0.3371	3.609	0.7745

2759-001
A Single Dose Toxicity Study of Test Article Administered by Intrathecal Injection in Rats

Individual Organ Weight Values
Day 91

Sex: Male Day: 91 Relative to Start Date

Low Dose	Testes/BrWt (ratio)	Thymus (g)	Thymus/BWt (%)	Thymus/BrWt (ratio)	Thyroid/para (fixed) (g)	Thyroid/para (fixed)/BWt (%)	Thyroid/para (fixed)/BrWt (ratio)
2001	1.7393	0.233	0.0481	0.1101	0.0252	0.0052	0.0119
2002	1.7418	0.353	0.0621	0.1710	0.0370	0.0065	0.0179
2003	1.8179	0.350	0.0803	0.1686	0.0184	0.0042	0.0089
2004	1.6932	0.301	0.0549	0.1356	0.0250	0.0046	0.0113
2005	1.6133	0.145	0.0311	0.0648	0.0254	0.0055	0.0114

2759-001
A Single Dose Toxicity Study of Test Article Administered by Intrathecal Injection in Rats

Individual Organ Weight Values
Day 91

Sex: Male Day: 91 Relative to Start Date

Mid Dose	Body Weight (g)	Brain (g)	Brain/BWt (%)	Adrenal glands (g)	Adrenal gl/ BWt (%)	Adrenal gl/ BrWt (ratio)	Epididymides (g)
3001	487	2.159	0.4433	0.096	0.0197	0.0445	1.525
3002	523	2.056	0.3931	0.064	0.0122	0.0311	1.544
3003	587	2.090	0.3560	0.056	0.0095	0.0268	1.430
3004	545	2.157	0.3958	0.075	0.0138	0.0348	1.673
3005	613	2.057	0.3356	0.061	0.0100	0.0297	1.507

2759-001
A Single Dose Toxicity Study of Test Article Administered by Intrathecal Injection in Rats

Individual Organ Weight Values
Day 91

Sex: Male Day: 91 Relative to Start Date

Mid Dose	Epididymides /BWt (%)	Epididymides /BrWt (ratio)	Heart (g)	Heart/BWt (%)	Heart/BrWt (ratio)	Kidneys (g)	Kidneys/BWt (%)
3001	0.3131	0.7063	1.810	0.3717	0.8384	3.592	0.7376
3002	0.2952	0.7510	1.955	0.3738	0.9509	3.845	0.7352
3003	0.2436	0.6842	1.885	0.3211	0.9019	5.105	0.8697
3004	0.3070	0.7756	1.634	0.2998	0.7575	3.935	0.7220
3005	0.2458	0.7326	2.234	0.3644	1.0860	3.638	0.5935

2759-001
A Single Dose Toxicity Study of Test Article Administered by Intrathecal Injection in Rats

Individual Organ Weight Values
Day 91

Sex: Male Day: 91 Relative to Start Date

Mid Dose	Kidneys/BrWt (ratio)	Liver (g)	Liver/BWt (%)	Liver/BrWt (ratio)	Lung (g)	Lung/BWt (%)	Lung/BrWt (ratio)
3001	1.6637	14.994	3.0789	6.9449	1.974	0.4053	0.9143
3002	1.8701	17.631	3.3711	8.5754	2.280	0.4359	1.1089
3003	2.4426	19.359	3.2980	9.2627	1.920	0.3271	0.9187
3004	1.8243	17.125	3.1422	7.9393	1.978	0.3629	0.9170
3005	1.7686	16.705	2.7251	8.1211	3.200	0.5220	1.5557

2759-001
A Single Dose Toxicity Study of Test Article Administered by Intrathecal Injection in Rats

Individual Organ Weight Values
Day 91

Sex: Male Day: 91 Relative to Start Date

Mid Dose	Pituitary (fixed) (g)	Pituitary (fixed)/BWt (%)	Pituitary (fixed)/BrWt (ratio)	Prostate gland (g)	Prostate gland/BWt (%)	Prostate gland/BrWt (ratio)	Sal, mand/ sub., rt (g)
3001	0.0152	0.0031	0.0070	1.499	0.3078	0.6943	0.405
3002	0.0186	0.0036	0.0090	1.326	0.2535	0.6449	0.464
3003	0.0186	0.0032	0.0089	1.230	0.2095	0.5885	0.419
3004	0.0185	0.0034	0.0086	2.025	0.3716	0.9388	0.487
3005	0.0183	0.0030	0.0089	1.446	0.2359	0.7030	0.273

2759-001
A Single Dose Toxicity Study of Test Article Administered by Intrathecal Injection in Rats

Individual Organ Weight Values
Day 91

Sex: Male Day: 91 Relative to Start Date

Mid Dose	Sal gl mand/ sub., rt/BWt (%)	Sal gl mand/ sub.,rt/BrWt (ratio)	Spleen (g)	Spleen/BWt (%)	Spleen/BrWt (ratio)	Testes (g)	Testes/BWt (%)
3001	0.0832	0.1876	0.976	0.2004	0.4521	3.323	0.6823
3002	0.0887	0.2257	0.770	0.1472	0.3745	3.604	0.6891
3003	0.0714	0.2005	0.878	0.1496	0.4201	4.151	0.7072
3004	0.0894	0.2258	0.922	0.1692	0.4274	3.737	0.6857
3005	0.0445	0.1327	0.872	0.1423	0.4239	3.544	0.5781

2759-001
A Single Dose Toxicity Study of Test Article Administered by Intrathecal Injection in Rats

Individual Organ Weight Values
Day 91

Sex: Male Day: 91 Relative to Start Date

Mid Dose	Testes/BrWt (ratio)	Thymus (g)	Thymus/BWt (%)	Thymus/BrWt (ratio)	Thyroid/para (fixed) (g)	Thyroid/para (fixed)/BWt (%)	Thyroid/para (fixed)/BrWt (ratio)
3001	1.5391	0.391	0.0803	0.1811	0.0285	0.0059	0.0132
3002	1.7529	0.384	0.0734	0.1868	0.0287	0.0055	0.0140
3003	1.9861	0.257	0.0438	0.1230	0.0294	0.0050	0.0141
3004	1.7325	0.329	0.0604	0.1525	0.0420	0.0077	0.0195
3005	1.7229	0.435	0.0710	0.2115	0.0351	0.0057	0.0171

2759-001
A Single Dose Toxicity Study of Test Article Administered by Intrathecal Injection in Rats

Individual Organ Weight Values
Day 91

Sex: Male Day: 91 Relative to Start Date

High Dose	Body Weight (g)	Brain (g)	Brain/BWt (%)	Adrenal glands (g)	Adrenal gl/ BWt (%)	Adrenal gl/ BrWt (ratio)	Epididymides (g)
4001	634	2.174	0.3429	0.069	0.0109	0.0317	1.451
4002	331	2.190	0.6616	0.059	0.0178	0.0269	1.349
4003	483	2.040	0.4224	0.063	0.0130	0.0309	1.477
4004	658	2.086	0.3170	0.088	0.0134	0.0422	1.574
4005	564	2.119	0.3757	0.062	0.0110	0.0293	1.532

2759-001
A Single Dose Toxicity Study of Test Article Administered by Intrathecal Injection in Rats

Individual Organ Weight Values
Day 91

Sex: Male Day: 91 Relative to Start Date

High Dose	Epididymides /BWt (%)	Epididymides /BrWt (ratio)	Heart (g)	Heart/BWt (%)	Heart/BrWt (ratio)	Kidneys (g)	Kidneys/BWt (%)
4001	0.2289	0.6674	2.317	0.3655	1.0658	4.252	0.6707
4002	0.4076	0.6160	1.694	0.5118	0.7735	3.853	1.1640
4003	0.3058	0.7240	1.805	0.3737	0.8848	3.189	0.6602
4004	0.2392	0.7546	2.490	0.3784	1.1937	4.101	0.6233
4005	0.2716	0.7230	2.101	0.3725	0.9915	3.813	0.6761

2759-001
A Single Dose Toxicity Study of Test Article Administered by Intrathecal Injection in Rats

Individual Organ Weight Values
Day 91

Sex: Male Day: 91 Relative to Start Date

High Dose	Kidneys/BrWt (ratio)	Liver (g)	Liver/BWt (%)	Liver/BrWt (ratio)	Lung (g)	Lung/BWt (%)	Lung/BrWt (ratio)
4001	1.9558	18.486	2.9158	8.5032	1.822	0.2874	0.8381
4002	1.7594	17.113	5.1701	7.8142	1.777	0.5369	0.8114
4003	1.5632	14.114	2.9222	6.9186	2.019	0.4180	0.9897
4004	1.9660	20.480	3.1125	9.8178	1.970	0.2994	0.9444
4005	1.7994	17.561	3.1137	8.2874	2.753	0.4881	1.2992

2759-001
A Single Dose Toxicity Study of Test Article Administered by Intrathecal Injection in Rats

Individual Organ Weight Values
Day 91

Sex: Male Day: 91 Relative to Start Date

High Dose	Pituitary (fixed) (g)	Pituitary (fixed)/BWt (%)	Pituitary (fixed)/BrWt (ratio)	Prostate gland (g)	Prostate gland/BWt (%)	Prostate gland/BrWt (ratio)	Sal, mand/ sub., rt (g)
4001	0.0179	0.0028	0.0082	1.534	0.2420	0.7056	0.373
4002	0.0181	0.0055	0.0083	1.238	0.3740	0.5653	0.493
4003	0.0138	0.0029	0.0068	1.285	0.2660	0.6299	0.295
4004	0.0140	0.0021	0.0067	1.682	0.2556	0.8063	0.406
4005	0.0173	0.0031	0.0082	1.913	0.3392	0.9028	0.373

2759-001
A Single Dose Toxicity Study of Test Article Administered by Intrathecal Injection in Rats

Individual Organ Weight Values
Day 91

Sex: Male Day: 91 Relative to Start Date

High Dose	Sal gl mand/ sub., rt/BWt (%)	Sal gl mand/ sub.,rt/BrWt (ratio)	Spleen (g)	Spleen/BWt (%)	Spleen/BrWt (ratio)	Testes (g)	Testes/BWt (%)
4001	0.0588	0.1716	1.036	0.1634	0.4765	3.199	0.5046
4002	0.1489	0.2251	1.065	0.3218	0.4863	3.407	1.0293
4003	0.0611	0.1446	0.931	0.1928	0.4564	3.350	0.6936
4004	0.0617	0.1946	1.005	0.1527	0.4818	3.794	0.5766
4005	0.0661	0.1760	1.016	0.1801	0.4795	3.660	0.6489

2759-001
A Single Dose Toxicity Study of Test Article Administered by Intrathecal Injection in Rats

Individual Organ Weight Values
Day 91

Sex: Male Day: 91 Relative to Start Date

High Dose	Testes/BrWt (ratio)	Thymus (g)	Thymus/BWt (%)	Thymus/BrWt (ratio)	Thyroid/para (fixed) (g)	Thyroid/para (fixed)/BWt (%)	Thyroid/para (fixed)/BrWt (ratio)
4001	1.4715	0.438	0.0691	0.2015	0.0380	0.0060	0.0175
4002	1.5557	0.248	0.0749	0.1132	0.0318	0.0096	0.0145
4003	1.6422	0.380	0.0787	0.1863	0.0288	0.0060	0.0141
4004	1.8188	0.554	0.0842	0.2656	0.0276	0.0042	0.0132
4005	1.7272	0.393	0.0697	0.1855	0.0268	0.0048	0.0126

2759-001
A Single Dose Toxicity Study of Test Article Administered by Intrathecal Injection in Rats

Individual Organ Weight Values
Day 91

Sex: Female Day: 91 Relative to Start Date

Vehicle	Body Weight (g)	Brain (g)	Brain/BWt (%)	Adrenal glands (g)	Adrenal gl/ BWt (%)	Adrenal gl/ BrWt (ratio)	Heart (g)
1501	284	1.858	0.6542	0.071	0.0250	0.0382	1.104
1502	285	2.052	0.7200	0.075	0.0263	0.0365	1.162
1503	294	1.959	0.6663	0.069	0.0235	0.0352	1.215
1504	237	1.895	0.7996	0.062	0.0262	0.0327	0.863
1505	297	1.906	0.6418	0.069	0.0232	0.0362	1.200

2759-001
A Single Dose Toxicity Study of Test Article Administered by Intrathecal Injection in Rats

Individual Organ Weight Values
Day 91

Sex: Female Day: 91 Relative to Start Date

Vehicle	Heart/BWt (%)	Heart/BrWt (ratio)	Kidneys (g)	Kidneys/BWt (%)	Kidneys/BrWt (ratio)	Liver (g)	Liver/BWt (%)
1501	0.3887	0.5942	1.988	0.7000	1.0700	9.652	3.3986
1502	0.4077	0.5663	2.211	0.7758	1.0775	11.195	3.9281
1503	0.4133	0.6202	2.287	0.7779	1.1674	10.781	3.6670
1504	0.3641	0.4554	1.783	0.7523	0.9409	8.439	3.5608
1505	0.4040	0.6296	2.216	0.7461	1.1626	8.072	2.7178

2759-001
A Single Dose Toxicity Study of Test Article Administered by Intrathecal Injection in Rats

Individual Organ Weight Values
Day 91

Sex: Female Day: 91 Relative to Start Date

Vehicle	Liver/BrWt (ratio)	Lung (g)	Lung/BWt (%)	Lung/BrWt (ratio)	Ovaries (g)	Ovaries/BWt (%)	Ovaries/BrWt (ratio)
1501	5.1948	1.369	0.4820	0.7368	0.083	0.0292	0.0447
1502	5.4557	1.519	0.5330	0.7403	0.117	0.0411	0.0570
1503	5.5033	1.317	0.4480	0.6723	0.043	0.0146	0.0219
1504	4.4533	1.238	0.5224	0.6533	0.076	0.0321	0.0401
1505	4.2350	1.591	0.5357	0.8347	0.115	0.0387	0.0603

2759-001
A Single Dose Toxicity Study of Test Article Administered by Intrathecal Injection in Rats

Individual Organ Weight Values
Day 91

Sex: Female Day: 91 Relative to Start Date

Vehicle	Oviducts (g)	Oviducts/BWt (%)	Oviducts/ BrWt (ratio)	Pituitary (fixed) (g)	Pituitary (fixed)/BWt (%)	Pituitary (fixed)/BrWt (ratio)	Sal, mand/ sub., rt (g)
1501	0.039	0.0137	0.0210	0.0178	0.0063	0.0096	0.192
1502	0.018	0.0063	0.0088	0.0226	0.0079	0.0110	0.242
1503	0.041	0.0139	0.0209	0.0289	0.0098	0.0148	0.221
1504	0.021	0.0089	0.0111	0.0185	0.0078	0.0098	0.192
1505	0.032	0.0108	0.0168	0.0234	0.0079	0.0123	0.220

2759-001
A Single Dose Toxicity Study of Test Article Administered by Intrathecal Injection in Rats

Individual Organ Weight Values
Day 91

Sex: Female Day: 91 Relative to Start Date

Vehicle	Sal gl mand/ sub., rt/BWt (%)	Sal gl mand/ sub.,rt/BrWt (ratio)	Spleen (g)	Spleen/BWt (%)	Spleen/BrWt (ratio)	Thymus (g)	Thymus/BWt (%)
1501	0.0676	0.1033	0.454	0.1599	0.2443	0.395	0.1391
1502	0.0849	0.1179	0.543	0.1905	0.2646	0.316	0.1109
1503	0.0752	0.1128	0.558	0.1898	0.2848	0.254	0.0864
1504	0.0810	0.1013	0.473	0.1996	0.2496	0.227	0.0958
1505	0.0741	0.1154	0.565	0.1902	0.2964	0.318	0.1071

2759-001
A Single Dose Toxicity Study of Test Article Administered by Intrathecal Injection in Rats

Individual Organ Weight Values
Day 91

Sex: Female Day: 91 Relative to Start Date

Vehicle	Thymus/BrWt (ratio)	Thyroid/para (fixed) (g)	Thyroid/para (fixed)/BWt (%)	Thyroid/para (fixed)/BrWt (ratio)	Uterus w/ cervix (g)	Uterus w/ cervix/BWt (%)	Uterus w/ cervix/BrWt (ratio)
1501	0.2126	0.0186	0.0065	0.0100	0.612	0.2155	0.3294
1502	0.1540	0.0287	0.0101	0.0140	0.595	0.2088	0.2900
1503	0.1297	0.0185	0.0063	0.0094	0.477	0.1622	0.2435
1504	0.1198	0.0219	0.0092	0.0116	0.554	0.2338	0.2923
1505	0.1668	0.0223	0.0075	0.0117	0.543	0.1828	0.2849

2759-001
A Single Dose Toxicity Study of Test Article Administered by Intrathecal Injection in Rats

Individual Organ Weight Values
Day 91

Sex: Female Day: 91 Relative to Start Date

Low Dose	Body Weight (g)	Brain (g)	Brain/BWt (%)	Adrenal glands (g)	Adrenal gl/ BWt (%)	Adrenal gl/ BrWt (ratio)	Heart (g)
2501	286	1.867	0.6528	0.099	0.0346	0.0530	1.275
2502	284	2.165	0.7623	0.046	0.0162	0.0212	1.169
2503	260	1.967	0.7565	0.045	0.0173	0.0229	0.873
2504	238	1.894	0.7958	0.073	0.0307	0.0385	0.914
2505	279	1.958	0.7018	0.080	0.0287	0.0409	1.160

2759-001
A Single Dose Toxicity Study of Test Article Administered by Intrathecal Injection in Rats

Individual Organ Weight Values
Day 91

Sex: Female Day: 91 Relative to Start Date

Low Dose	Heart/BWt (%)	Heart/BrWt (ratio)	Kidneys (g)	Kidneys/BWt (%)	Kidneys/BrWt (ratio)	Liver (g)	Liver/BWt (%)
2501	0.4458	0.6829	2.226	0.7783	1.1923	9.849	3.4437
2502	0.4116	0.5400	2.376	0.8366	1.0975	10.036	3.5338
2503	0.3358	0.4438	1.883	0.7242	0.9573	8.120	3.1231
2504	0.3840	0.4826	1.947	0.8181	1.0280	7.700	3.2353
2505	0.4158	0.5924	2.060	0.7384	1.0521	9.438	3.3828

2759-001
A Single Dose Toxicity Study of Test Article Administered by Intrathecal Injection in Rats

Individual Organ Weight Values
Day 91

Sex: Female Day: 91 Relative to Start Date

Low Dose	Liver/BrWt (ratio)	Lung (g)	Lung/BWt (%)	Lung/BrWt (ratio)	Ovaries (g)	Ovaries/BWt (%)	Ovaries/BrWt (ratio)
2501	5.2753	1.292	0.4517	0.6920	0.067	0.0234	0.0359
2502	4.6356	1.496	0.5268	0.6910	0.103	0.0363	0.0476
2503	4.1281	1.110	0.4269	0.5643	0.069	0.0265	0.0351
2504	4.0655	1.395	0.5861	0.7365	0.075	0.0315	0.0396
2505	4.8202	1.547	0.5545	0.7901	0.071	0.0254	0.0363

2759-001
A Single Dose Toxicity Study of Test Article Administered by Intrathecal Injection in Rats

Individual Organ Weight Values
Day 91

Sex: Female Day: 91 Relative to Start Date

Low Dose	Oviducts (g)	Oviducts/BWt (%)	Oviducts/ BrWt (ratio)	Pituitary (fixed) (g)	Pituitary (fixed)/BWt (%)	Pituitary (fixed)/BrWt (ratio)	Sal, mand/ sub., rt (g)
2501	0.024	0.0084	0.0129	0.0173	0.0060	0.0093	0.231
2502	0.018	0.0063	0.0083	0.0186	0.0065	0.0086	0.251
2503	0.021	0.0081	0.0107	0.0165	0.0063	0.0084	0.203
2504	0.040	0.0168	0.0211	0.0233	0.0098	0.0123	0.185
2505	0.016	0.0057	0.0082	0.0295	0.0106	0.0151	0.180

2759-001
A Single Dose Toxicity Study of Test Article Administered by Intrathecal Injection in Rats

Individual Organ Weight Values
Day 91

Sex: Female Day: 91 Relative to Start Date

Low Dose	Sal gl mand/ sub., rt/BWt (%)	Sal gl mand/ sub.,rt/BrWt (ratio)	Spleen (g)	Spleen/BWt (%)	Spleen/BrWt (ratio)	Thymus (g)	Thymus/BWt (%)
2501	0.0808	0.1237	0.469	0.1640	0.2512	0.325	0.1136
2502	0.0884	0.1159	0.622	0.2190	0.2873	0.275	0.0968
2503	0.0781	0.1032	0.575	0.2212	0.2923	0.237	0.0912
2504	0.0777	0.0977	0.402	0.1689	0.2122	0.199	0.0836
2505	0.0645	0.0919	0.469	0.1681	0.2395	0.369	0.1323

2759-001
A Single Dose Toxicity Study of Test Article Administered by Intrathecal Injection in Rats

Individual Organ Weight Values
Day 91

Sex: Female Day: 91 Relative to Start Date

Low Dose	Thymus/BrWt (ratio)	Thyroid/para (fixed) (g)	Thyroid/para (fixed)/BWt (%)	Thyroid/para (fixed)/BrWt (ratio)	Uterus w/ cervix (g)	Uterus w/ cervix/BWt (%)	Uterus w/ cervix/BrWt (ratio)
2501	0.1741	0.0250	0.0087	0.0134	0.671	0.2346	0.3594
2502	0.1270	0.0229	0.0081	0.0106	0.444	0.1563	0.2051
2503	0.1205	0.0193	0.0074	0.0098	0.619	0.2381	0.3147
2504	0.1051	0.0194	0.0082	0.0102	0.435	0.1828	0.2297
2505	0.1885	0.0203	0.0073	0.0104	0.838	0.3004	0.4280

2759-001
A Single Dose Toxicity Study of Test Article Administered by Intrathecal Injection in Rats

Individual Organ Weight Values
Day 91

Sex: Female Day: 91 Relative to Start Date

Mid Dose	Body Weight (g)	Brain (g)	Brain/BWt (%)	Adrenal glands (g)	Adrenal gl/ BWt (%)	Adrenal gl/ BrWt (ratio)	Heart (g)
3501	272	2.112	0.7765	0.105	0.0386	0.0497	1.390
3502	296	1.878	0.6345	0.091	0.0307	0.0485	1.322
3503	259	2.004	0.7737	0.082	0.0317	0.0409	1.060
3504	257	1.946	0.7572	0.091	0.0354	0.0468	1.266
3505	256	1.949	0.7613	0.077	0.0301	0.0395	1.049

2759-001
A Single Dose Toxicity Study of Test Article Administered by Intrathecal Injection in Rats

Individual Organ Weight Values
Day 91

Sex: Female Day: 91 Relative to Start Date

Mid Dose	Heart/BWt (%)	Heart/BrWt (ratio)	Kidneys (g)	Kidneys/BWt (%)	Kidneys/BrWt (ratio)	Liver (g)	Liver/BWt (%)
3501	0.5110	0.6581	2.516	0.9250	1.1913	9.623	3.5379
3502	0.4466	0.7039	2.297	0.7760	1.2231	10.062	3.3993
3503	0.4093	0.5289	2.190	0.8456	1.0928	8.323	3.2135
3504	0.4926	0.6506	2.372	0.9230	1.2189	8.757	3.4074
3505	0.4098	0.5382	2.022	0.7898	1.0375	9.086	3.5492

2759-001
A Single Dose Toxicity Study of Test Article Administered by Intrathecal Injection in Rats

Individual Organ Weight Values
Day 91

Sex: Female Day: 91 Relative to Start Date

Mid Dose	Liver/BrWt (ratio)	Lung (g)	Lung/BWt (%)	Lung/BrWt (ratio)	Ovaries (g)	Ovaries/BWt (%)	Ovaries/BrWt (ratio)
3501	4.5563	1.591	0.5849	0.7533	0.111	0.0408	0.0526
3502	5.3578	1.339	0.4524	0.7130	0.062	0.0209	0.0330
3503	4.1532	1.539	0.5942	0.7680	0.096	0.0371	0.0479
3504	4.5000	1.413	0.5498	0.7261	0.088	0.0342	0.0452
3505	4.6619	1.332	0.5203	0.6834	0.091	0.0355	0.0467

2759-001
A Single Dose Toxicity Study of Test Article Administered by Intrathecal Injection in Rats

Individual Organ Weight Values
Day 91

Sex: Female Day: 91 Relative to Start Date

Mid Dose	Oviducts (g)	Oviducts/BWt (%)	Oviducts/ BrWt (ratio)	Pituitary (fixed) (g)	Pituitary (fixed)/BWt (%)	Pituitary (fixed)/BrWt (ratio)	Sal, mand/ sub., rt (g)
3501	0.031	0.0114	0.0147	0.0221	0.0081	0.0105	0.237
3502	0.027	0.0091	0.0144	0.0329	0.0111	0.0175	0.223
3503	0.037	0.0143	0.0185	0.0228	0.0088	0.0114	0.195
3504	0.027	0.0105	0.0139	0.0265	0.0103	0.0136	0.276
3505	0.038	0.0148	0.0195	0.0235	0.0092	0.0121	0.213

2759-001
A Single Dose Toxicity Study of Test Article Administered by Intrathecal Injection in Rats

Individual Organ Weight Values
Day 91

Sex: Female Day: 91 Relative to Start Date

Mid Dose	Sal gl mand/ sub., rt/BWt (%)	Sal gl mand/ sub.,rt/BrWt (ratio)	Spleen (g)	Spleen/BWt (%)	Spleen/BrWt (ratio)	Thymus (g)	Thymus/BWt (%)
3501	0.0871	0.1122	0.661	0.2430	0.3130	0.293	0.1077
3502	0.0753	0.1187	0.521	0.1760	0.2774	0.333	0.1125
3503	0.0753	0.0973	0.659	0.2544	0.3288	0.310	0.1197
3504	0.1074	0.1418	0.524	0.2039	0.2693	0.263	0.1023
3505	0.0832	0.1093	0.552	0.2156	0.2832	0.270	0.1055

2759-001
A Single Dose Toxicity Study of Test Article Administered by Intrathecal Injection in Rats

Individual Organ Weight Values
Day 91

Sex: Female Day: 91 Relative to Start Date

Mid Dose	Thymus/BrWt (ratio)	Thyroid/para (fixed) (g)	Thyroid/para (fixed)/BWt (%)	Thyroid/para (fixed)/BrWt (ratio)	Uterus w/ cervix (g)	Uterus w/ cervix/BWt (%)	Uterus w/ cervix/BrWt (ratio)
3501	0.1387	0.0244	0.0090	0.0116	0.511	0.1879	0.2420
3502	0.1773	0.0201	0.0068	0.0107	0.674	0.2277	0.3589
3503	0.1547	0.0207	0.0080	0.0103	1.222	0.4718	0.6098
3504	0.1351	0.0188	0.0073	0.0097	0.699	0.2720	0.3592
3505	0.1385	0.0208	0.0081	0.0107	1.062	0.4148	0.5449

2759-001
A Single Dose Toxicity Study of Test Article Administered by Intrathecal Injection in Rats

Individual Organ Weight Values
Day 91

Sex: Female Day: 91 Relative to Start Date

High Dose	Body Weight (g)	Brain (g)	Brain/BWt (%)	Adrenal glands (g)	Adrenal gl/ BWt (%)	Adrenal gl/ BrWt (ratio)	Heart (g)
4501	298	2.186	0.7336	0.084	0.0282	0.0384	1.293
4502	329	1.904	0.5787	0.103	0.0313	0.0541	1.349
4503	276	1.911	0.6924	0.081	0.0293	0.0424	1.169
4504	320	1.888	0.5900	0.062	0.0194	0.0328	1.267
4505	285	1.939	0.6804	0.103	0.0361	0.0531	0.989

2759-001
A Single Dose Toxicity Study of Test Article Administered by Intrathecal Injection in Rats

Individual Organ Weight Values
Day 91

Sex: Female Day: 91 Relative to Start Date

High Dose	Heart/BWt (%)	Heart/BrWt (ratio)	Kidneys (g)	Kidneys/BWt (%)	Kidneys/BrWt (ratio)	Liver (g)	Liver/BWt (%)
4501	0.4339	0.5915	2.287	0.7674	1.0462	10.286	3.4517
4502	0.4100	0.7085	2.405	0.7310	1.2631	12.587	3.8258
4503	0.4236	0.6117	2.167	0.7851	1.1340	9.487	3.4373
4504	0.3959	0.6711	2.238	0.6994	1.1854	8.810	2.7531
4505	0.3470	0.5101	1.969	0.6909	1.0155	10.193	3.5765

2759-001
A Single Dose Toxicity Study of Test Article Administered by Intrathecal Injection in Rats

Individual Organ Weight Values
Day 91

Sex: Female Day: 91 Relative to Start Date

High Dose	Liver/BrWt (ratio)	Lung (g)	Lung/BWt (%)	Lung/BrWt (ratio)	Ovaries (g)	Ovaries/BWt (%)	Ovaries/BrWt (ratio)
4501	4.7054	1.350	0.4530	0.6176	0.101	0.0339	0.0462
4502	6.6108	1.467	0.4459	0.7705	0.095	0.0289	0.0499
4503	4.9644	1.569	0.5685	0.8210	0.102	0.0370	0.0534
4504	4.6663	1.339	0.4184	0.7092	0.105	0.0328	0.0556
4505	5.2568	1.766	0.6196	0.9108	0.101	0.0354	0.0521

2759-001
A Single Dose Toxicity Study of Test Article Administered by Intrathecal Injection in Rats

Individual Organ Weight Values
Day 91

Sex: Female Day: 91 Relative to Start Date

High Dose	Oviducts (g)	Oviducts/BWt (%)	Oviducts/ BrWt (ratio)	Pituitary (fixed) (g)	Pituitary (fixed)/BWt (%)	Pituitary (fixed)/BrWt (ratio)	Sal, mand/ sub., rt (g)
4501	0.036	0.0121	0.0165	0.0239	0.0080	0.0109	0.182
4502	0.034	0.0103	0.0179	0.0268	0.0081	0.0141	0.232
4503	0.040	0.0145	0.0209	0.0269	0.0097	0.0141	0.215
4504	0.016	0.0050	0.0085	0.0243	0.0076	0.0129	0.235
4505	0.041	0.0144	0.0211	0.0223	0.0078	0.0115	0.305

2759-001
A Single Dose Toxicity Study of Test Article Administered by Intrathecal Injection in Rats

Individual Organ Weight Values
Day 91

Sex: Female Day: 91 Relative to Start Date

High Dose	Sal gl mand/ sub., rt/BWt (%)	Sal gl mand/ sub.,rt/BrWt (ratio)	Spleen (g)	Spleen/BWt (%)	Spleen/BrWt (ratio)	Thymus (g)	Thymus/BWt (%)
4501	0.0611	0.0833	0.595	0.1997	0.2722	0.271	0.0909
4502	0.0705	0.1218	0.705	0.2143	0.3703	0.404	0.1228
4503	0.0779	0.1125	0.522	0.1891	0.2732	0.204	0.0739
4504	0.0734	0.1245	0.699	0.2184	0.3702	0.427	0.1334
4505	0.1070	0.1573	0.695	0.2439	0.3584	0.317	0.1112

2759-001
A Single Dose Toxicity Study of Test Article Administered by Intrathecal Injection in Rats

Individual Organ Weight Values
Day 91

Sex: Female Day: 91 Relative to Start Date

High Dose	Thymus/BrWt (ratio)	Thyroid/para (fixed) (g)	Thyroid/para (fixed)/BWt (%)	Thyroid/para (fixed)/BrWt (ratio)	Uterus w/ cervix (g)	Uterus w/ cervix/BWt (%)	Uterus w/ cervix/BrWt (ratio)
4501	0.1240	0.0233	0.0078	0.0107	0.599	0.2010	0.2740
4502	0.2122	0.0258	0.0078	0.0136	0.646	0.1964	0.3393
4503	0.1068	0.0251	0.0091	0.0131	0.605	0.2192	0.3166
4504	0.2262	0.0318	0.0099	0.0168	0.790	0.2469	0.4184
4505	0.1635	0.0268	0.0094	0.0138	0.583	0.2046	0.3007

Appendix 5
Individual Animal Data Macroscopic and Microscopic Pathology Findings (Day 8)

2759-001 - Individual Animal Data Record: Pathology, Day 8

Animal: 1006 Sex: Male

Dose: Vehicle

Study Day (Week) of Death: 8 (2)

Gross Pathology Observations [Correlation]:

No observations found

Any remaining protocol required tissues, which have been examined, have no visible lesions

Gross Pathology - The following Tissues were Not Examined:

None

Histopathology Animal Details:

No animal details found

Histopathology Observations [Correlation]:

liver : infiltration, mononuclear cell; multifocal, minimal

Any remaining protocol required tissues, which have been examined, have no visible lesions

Histopathology - The following Tissues were Not Examined:

None

2759-001 - Individual Animal Data Record: Pathology, Day 8

Animal: 1007 Sex: Male

Dose: Vehicle

Study Day (Week) of Death: 8 (2)

Gross Pathology Observations [Correlation]:

No observations found

Any remaining protocol required tissues, which have been examined, have no visible lesions

Gross Pathology - The following Tissues were Not Examined:

None

Histopathology Animal Details:

No animal details found

Histopathology Observations [Correlation]:

lung : hemorrhage; multifocal, minimal

Any remaining protocol required tissues, which have been examined, have no visible lesions

Histopathology - The following Tissues were Not Examined:

thymus - Not In Plane Of Section

2759-001 - Individual Animal Data Record: Pathology, Day 8

Animal: 1008 Sex: Male

Dose: Vehicle

Study Day (Week) of Death: 8 (2)

Gross Pathology Observations [Correlation]:

No observations found

Any remaining protocol required tissues, which have been examined, have no visible lesions

Gross Pathology - The following Tissues were Not Examined:

None

Histopathology Animal Details:

No animal details found

Histopathology Observations [Correlation]:

kidneys : infiltration, mononuclear cell; multifocal, minimal

Any remaining protocol required tissues, which have been examined, have no visible lesions

Histopathology - The following Tissues were Not Examined:

None

2759-001 - Individual Animal Data Record: Pathology, Day 8

Animal: 1009 Sex: Male

Dose: Vehicle

Study Day (Week) of Death: 8 (2)

Gross Pathology Observations [Correlation]:

No observations found

Any remaining protocol required tissues, which have been examined, have no visible lesions

Gross Pathology - The following Tissues were Not Examined:

None

Histopathology Animal Details:

No animal details found

Histopathology Observations [Correlation]:

dorsal root ganglia, lumbar, left : infiltration, mononuclear cell, perivascular; focal, minimal

Any remaining protocol required tissues, which have been examined, have no visible lesions

Histopathology - The following Tissues were Not Examined:

None

2759-001 - Individual Animal Data Record: Pathology, Day 8

Animal: 1010 Sex: Male

Dose: Vehicle

Study Day (Week) of Death: 8 (2)

Gross Pathology Observations [Correlation]:

No observations found

Any remaining protocol required tissues, which have been examined, have no visible lesions

Gross Pathology - The following Tissues were Not Examined:

None

Histopathology Animal Details:

No animal details found

Histopathology Observations [Correlation]:

No observations found

Any remaining protocol required tissues, which have been examined, have no visible lesions

Histopathology - The following Tissues were Not Examined:

None

2759-001 - Individual Animal Data Record: Pathology, Day 8

Animal: 1506 Sex: Female

Dose: Vehicle

Study Day (Week) of Death: 8 (2)

Gross Pathology Observations [Correlation]:

No observations found

Any remaining protocol required tissues, which have been examined, have no visible lesions

Gross Pathology - The following Tissues were Not Examined:

None

Histopathology Animal Details:

No animal details found

Histopathology Observations [Correlation]:

liver : infiltration, mononuclear cell; multifocal, minimal

Any remaining protocol required tissues, which have been examined, have no visible lesions

Histopathology - The following Tissues were Not Examined:

dorsal root ganglia, lumbar, left - Not In Plane Of Section

2759-001 - Individual Animal Data Record: Pathology, Day 8

Animal: 1507 Sex: Female

Dose: Vehicle

Study Day (Week) of Death: 8 (2)

Gross Pathology Observations [Correlation]:

No observations found

Any remaining protocol required tissues, which have been examined, have no visible lesions

Gross Pathology - The following Tissues were Not Examined:

None

Histopathology Animal Details:

No animal details found

Histopathology Observations [Correlation]:

dorsal root ganglia lumbar, right : infiltration, mononuclear cell, perivascular; focal, minimal
kidneys : mineralization; multifocal, minimal
liver : infiltration, mononuclear cell; multifocal, minimal
lung : infiltration, mononuclear cell; focal, minimal

Any remaining protocol required tissues, which have been examined, have no visible lesions

Histopathology - The following Tissues were Not Examined:

None

2759-001 - Individual Animal Data Record: Pathology, Day 8

Animal: 1508 Sex: Female

Dose: Vehicle

Study Day (Week) of Death: 8 (2)

Gross Pathology Observations [Correlation]:

No observations found

Any remaining protocol required tissues, which have been examined, have no visible lesions

Gross Pathology - The following Tissues were Not Examined:

None

Histopathology Animal Details:

No animal details found

Histopathology Observations [Correlation]:

liver : infiltration, mononuclear cell; multifocal, minimal

Any remaining protocol required tissues, which have been examined, have no visible lesions

Histopathology - The following Tissues were Not Examined:

None

2759-001 - Individual Animal Data Record: Pathology, Day 8

Animal: 1509 Sex: Female

Dose: Vehicle

Study Day (Week) of Death: 8 (2)

Gross Pathology Observations [Correlation]:

No observations found

Any remaining protocol required tissues, which have been examined, have no visible lesions

Gross Pathology - The following Tissues were Not Examined:

None

Histopathology Animal Details:

No animal details found

Histopathology Observations [Correlation]:

kidneys : mineralization; focal, minimal
lung : macrophages, alveolar; focal, minimal
spleen : hyperplasia, lymphoid, follicular; multifocal, minimal

Any remaining protocol required tissues, which have been examined, have no visible lesions

Histopathology - The following Tissues were Not Examined:

None

2759-001 - Individual Animal Data Record: Pathology, Day 8

Animal: 1510 Sex: Female

Dose: Vehicle

Study Day (Week) of Death: 8 (2)

Gross Pathology Observations [Correlation]:

No observations found

Any remaining protocol required tissues, which have been examined, have no visible lesions

Gross Pathology - The following Tissues were Not Examined:

None

Histopathology Animal Details:

No animal details found

Histopathology Observations [Correlation]:

kidneys : infiltration, mononuclear cell; focal, minimal
spleen : hyperplasia, lymphoid, follicular; multifocal, minimal

Any remaining protocol required tissues, which have been examined, have no visible lesions

Histopathology - The following Tissues were Not Examined:

None

2759-001 - Individual Animal Data Record: Pathology, Day 8

Animal: 2006 Sex: Male

Dose: Low Dose

Study Day (Week) of Death: 8 (2)

Gross Pathology Observations [Correlation]:

No observations found

Any remaining protocol required tissues, which have been examined, have no visible lesions

Gross Pathology - The following Tissues were Not Examined:

None

Histopathology Animal Details:

No animal details found

Histopathology Observations [Correlation]:

kidneys : mineralization; multifocal, minimal
lung : infiltration, mononuclear cell; minimal
thymus : hemorrhage; focal, minimal

Any remaining protocol required tissues, which have been examined, have no visible lesions

Histopathology - The following Tissues were Not Examined:

None

2759-001 - Individual Animal Data Record: Pathology, Day 8

Animal: 2007 Sex: Male

Dose: Low Dose

Study Day (Week) of Death: 8 (2)

Gross Pathology Observations [Correlation]:

No observations found

Any remaining protocol required tissues, which have been examined, have no visible lesions

Gross Pathology - The following Tissues were Not Examined:

None

Histopathology Animal Details:

No animal details found

Histopathology Observations [Correlation]:

liver : infiltration, mononuclear cell; focal, minimal
lung : infiltration, mononuclear cell; focal, minimal

Any remaining protocol required tissues, which have been examined, have no visible lesions

Histopathology - The following Tissues were Not Examined:

None

2759-001 - Individual Animal Data Record: Pathology, Day 8

Animal: 2008 Sex: Male

Dose: Low Dose

Study Day (Week) of Death: 8 (2)

Gross Pathology Observations [Correlation]:

No observations found

Any remaining protocol required tissues, which have been examined, have no visible lesions

Gross Pathology - The following Tissues were Not Examined:

None

Histopathology Animal Details:

No animal details found

Histopathology Observations [Correlation]:

kidneys : infiltration, mononuclear cell; focal, minimal
lung : infiltration, mononuclear cell; multifocal, minimal

Any remaining protocol required tissues, which have been examined, have no visible lesions

Histopathology - The following Tissues were Not Examined:

lymph node, mandibular - Not In Plane Of Section

2759-001 - Individual Animal Data Record: Pathology, Day 8

Animal: 2009 Sex: Male

Dose: Low Dose

Study Day (Week) of Death: 8 (2)

Gross Pathology Observations [Correlation]:

No observations found

Any remaining protocol required tissues, which have been examined, have no visible lesions

Gross Pathology - The following Tissues were Not Examined:

None

Histopathology Animal Details:

No animal details found

Histopathology Observations [Correlation]:

liver : infiltration, mononuclear cell; multifocal, minimal

Any remaining protocol required tissues, which have been examined, have no visible lesions

Histopathology - The following Tissues were Not Examined:

None

2759-001 - Individual Animal Data Record: Pathology, Day 8

Animal: 2010 Sex: Male

Dose: Low Dose

Study Day (Week) of Death: 8 (2)

Gross Pathology Observations [Correlation]:

No observations found

Any remaining protocol required tissues, which have been examined, have no visible lesions

Gross Pathology - The following Tissues were Not Examined:

None

Histopathology Animal Details:

No animal details found

Histopathology Observations [Correlation]:

dorsal root ganglia, lumbar, left : infiltration, mononuclear cell, perivascular; multifocal, minimal

Any remaining protocol required tissues, which have been examined, have no visible lesions

Histopathology - The following Tissues were Not Examined:

None

2759-001 - Individual Animal Data Record: Pathology, Day 8

Animal: 2506 Sex: Female

Dose: Low Dose

Study Day (Week) of Death: 8 (2)

Gross Pathology Observations [Correlation]:

No observations found

Any remaining protocol required tissues, which have been examined, have no visible lesions

Gross Pathology - The following Tissues were Not Examined:

None

Histopathology Animal Details:

No animal details found

Histopathology Observations [Correlation]:

lung : infiltration, mononuclear cell; multifocal, minimal

Any remaining protocol required tissues, which have been examined, have no visible lesions

Histopathology - The following Tissues were Not Examined:

dorsal root ganglia, thoracic, left - Not In Plane Of Section

2759-001 - Individual Animal Data Record: Pathology, Day 8

Animal: 2507 Sex: Female

Dose: Low Dose

Study Day (Week) of Death: 8 (2)

Gross Pathology Observations [Correlation]:

No observations found

Any remaining protocol required tissues, which have been examined, have no visible lesions

Gross Pathology - The following Tissues were Not Examined:

None

Histopathology Animal Details:

No animal details found

Histopathology Observations [Correlation]:

liver : infiltration, mononuclear cell; multifocal, minimal
lung : infiltration, mononuclear cell; focal, minimal
thymus : hemorrhage; multifocal, minimal
dorsal root ganglia, lumbar, left : infiltration, mononuclear cell, perivascular; focal, minimal

Any remaining protocol required tissues, which have been examined, have no visible lesions

Histopathology - The following Tissues were Not Examined:

dorsal root ganglia, thoracic, right - Not In Plane Of Section

2759-001 - Individual Animal Data Record: Pathology, Day 8

Animal: 2508 Sex: Female

Dose: Low Dose

Study Day (Week) of Death: 8 (2)

Gross Pathology Observations [Correlation]:

No observations found

Any remaining protocol required tissues, which have been examined, have no visible lesions

Gross Pathology - The following Tissues were Not Examined:

None

Histopathology Animal Details:

No animal details found

Histopathology Observations [Correlation]:

thymus : hemorrhage; multifocal, minimal

Any remaining protocol required tissues, which have been examined, have no visible lesions

Histopathology - The following Tissues were Not Examined:

dorsal root ganglia, thoracic, left - Not In Plane Of Section

2759-001 - Individual Animal Data Record: Pathology, Day 8

Animal: 2509 Sex: Female

Dose: Low Dose

Study Day (Week) of Death: 8 (2)

Gross Pathology Observations [Correlation]:

No observations found

Any remaining protocol required tissues, which have been examined, have no visible lesions

Gross Pathology - The following Tissues were Not Examined:

None

Histopathology Animal Details:

No animal details found

Histopathology Observations [Correlation]:

lung : infiltration, mononuclear cell; focal, minimal
thymus : hemorrhage; multifocal, minimal

Any remaining protocol required tissues, which have been examined, have no visible lesions

Histopathology - The following Tissues were Not Examined:

None

2759-001 - Individual Animal Data Record: Pathology, Day 8

Animal: 2510 Sex: Female
Dose: Low Dose
Study Day (Week) of Death: 8 (2)

Gross Pathology Observations [Correlation]:

No observations found

Any remaining protocol required tissues, which have been examined, have no visible lesions

Gross Pathology - The following Tissues were Not Examined:

None

Histopathology Animal Details:

No animal details found

Histopathology Observations [Correlation]:

dorsal root ganglia lumbar, right : infiltration, mononuclear cell, perivascular; focal, minimal
liver : infiltration, mononuclear cell; focal, minimal

Any remaining protocol required tissues, which have been examined, have no visible lesions

Histopathology - The following Tissues were Not Examined:

None

2759-001 - Individual Animal Data Record: Pathology, Day 8

Animal: 3006 Sex: Male

Dose: Mid Dose

Study Day (Week) of Death: 8 (2)

Gross Pathology Observations [Correlation]:

No observations found

Any remaining protocol required tissues, which have been examined, have no visible lesions

Gross Pathology - The following Tissues were Not Examined:

None

Histopathology Animal Details:

No animal details found

Histopathology Observations [Correlation]:

liver : hemorrhage; multifocal, minimal
lung : infiltration, mononuclear cell; multifocal, minimal

Any remaining protocol required tissues, which have been examined, have no visible lesions

Histopathology - The following Tissues were Not Examined:

None

2759-001 - Individual Animal Data Record: Pathology, Day 8

Animal: 3007 Sex: Male

Dose: Mid Dose

Study Day (Week) of Death: 8 (2)

Gross Pathology Observations [Correlation]:

No observations found

Any remaining protocol required tissues, which have been examined, have no visible lesions

Gross Pathology - The following Tissues were Not Examined:

None

Histopathology Animal Details:

No animal details found

Histopathology Observations [Correlation]:

kidneys : mineralization; multifocal, minimal
liver : infiltration, mononuclear cell; multifocal, minimal
lung : infiltration, mononuclear cell; focal, minimal

Any remaining protocol required tissues, which have been examined, have no visible lesions

Histopathology - The following Tissues were Not Examined:

None

2759-001 - Individual Animal Data Record: Pathology, Day 8

Animal: 3008 Sex: Male

Dose: Mid Dose

Study Day (Week) of Death: 8 (2)

Gross Pathology Observations [Correlation]:

No observations found

Any remaining protocol required tissues, which have been examined, have no visible lesions

Gross Pathology - The following Tissues were Not Examined:

None

Histopathology Animal Details:

No animal details found

Histopathology Observations [Correlation]:

liver : infiltration, mononuclear cell; multifocal, minimal
lung : infiltration, mixed cell, perivascular; focal, minimal

Any remaining protocol required tissues, which have been examined, have no visible lesions

Histopathology - The following Tissues were Not Examined:

None

2759-001 - Individual Animal Data Record: Pathology, Day 8

Animal: 3009 Sex: Male

Dose: Mid Dose

Study Day (Week) of Death: 8 (2)

Gross Pathology Observations [Correlation]:

No observations found

Any remaining protocol required tissues, which have been examined, have no visible lesions

Gross Pathology - The following Tissues were Not Examined:

None

Histopathology Animal Details:

No animal details found

Histopathology Observations [Correlation]:

liver : infiltration, mononuclear cell; multifocal, minimal
lung : infiltration, mononuclear cell; multifocal, minimal

Any remaining protocol required tissues, which have been examined, have no visible lesions

Histopathology - The following Tissues were Not Examined:

None

2759-001 - Individual Animal Data Record: Pathology, Day 8

Animal: 3010 Sex: Male

Dose: Mid Dose

Study Day (Week) of Death: 8 (2)

Gross Pathology Observations [Correlation]:

No observations found

Any remaining protocol required tissues, which have been examined, have no visible lesions

Gross Pathology - The following Tissues were Not Examined:

None

Histopathology Animal Details:

No animal details found

Histopathology Observations [Correlation]:

dorsal root ganglia lumbar, right : infiltration, mononuclear cell, perivascular; focal, minimal
liver : infiltration, mononuclear cell; multifocal, minimal
lung : infiltration, mononuclear cell; minimal
thymus : hemorrhage; multifocal, minimal

Any remaining protocol required tissues, which have been examined, have no visible lesions

Histopathology - The following Tissues were Not Examined:

None

2759-001 - Individual Animal Data Record: Pathology, Day 8

Animal: 3506 Sex: Female

Dose: Mid Dose

Study Day (Week) of Death: 8 (2)

Gross Pathology Observations [Correlation]:

No observations found

Any remaining protocol required tissues, which have been examined, have no visible lesions

Gross Pathology - The following Tissues were Not Examined:

None

Histopathology Animal Details:

No animal details found

Histopathology Observations [Correlation]:

liver : infiltration, mononuclear cell; focal, minimal

Any remaining protocol required tissues, which have been examined, have no visible lesions

Histopathology - The following Tissues were Not Examined:

None

2759-001 - Individual Animal Data Record: Pathology, Day 8

Animal: 3507 Sex: Female

Dose: Mid Dose

Study Day (Week) of Death: 8 (2)

Gross Pathology Observations [Correlation]:

No observations found

Any remaining protocol required tissues, which have been examined, have no visible lesions

Gross Pathology - The following Tissues were Not Examined:

None

Histopathology Animal Details:

No animal details found

Histopathology Observations [Correlation]:

liver : infiltration, mononuclear cell; multifocal, minimal

Any remaining protocol required tissues, which have been examined, have no visible lesions

Histopathology - The following Tissues were Not Examined:

None

2759-001 - Individual Animal Data Record: Pathology, Day 8

Animal: 3508 Sex: Female

Dose: Mid Dose

Study Day (Week) of Death: 8 (2)

Gross Pathology Observations [Correlation]:

No observations found

Any remaining protocol required tissues, which have been examined, have no visible lesions

Gross Pathology - The following Tissues were Not Examined:

None

Histopathology Animal Details:

No animal details found

Histopathology Observations [Correlation]:

dorsal root ganglia lumbar, right : infiltration, mononuclear cell, perivascular; focal, minimal
kidneys : mineralization; focal, minimal

Any remaining protocol required tissues, which have been examined, have no visible lesions

Histopathology - The following Tissues were Not Examined:

None

2759-001 - Individual Animal Data Record: Pathology, Day 8

Animal: 3509 Sex: Female

Dose: Mid Dose

Study Day (Week) of Death: 8 (2)

Gross Pathology Observations [Correlation]:

No observations found

Any remaining protocol required tissues, which have been examined, have no visible lesions

Gross Pathology - The following Tissues were Not Examined:

None

Histopathology Animal Details:

No animal details found

Histopathology Observations [Correlation]:

spinal cord, lumbar : gliosis; focal, minimal
spinal cord, lumbar : infiltration, mononuclear cell, perivascular; multifocal, minimal
spinal cord, thoracic : gliosis; multifocal, minimal
spinal cord, thoracic : infiltration, mononuclear cell, perivascular; multifocal, minimal

Any remaining protocol required tissues, which have been examined, have no visible lesions

Histopathology - The following Tissues were Not Examined:

None

2759-001 - Individual Animal Data Record: Pathology, Day 8

Animal: 3510 Sex: Female

Dose: Mid Dose

Study Day (Week) of Death: 8 (2)

Gross Pathology Observations [Correlation]:

No observations found

Any remaining protocol required tissues, which have been examined, have no visible lesions

Gross Pathology - The following Tissues were Not Examined:

None

Histopathology Animal Details:

No animal details found

Histopathology Observations [Correlation]:

spinal cord, lumbar : gliosis; focal, minimal
spinal cord, lumbar : infiltration, mononuclear cell, perivascular; multifocal, minimal
spleen : hyperplasia, lymphoid, follicular; multifocal, minimal

Any remaining protocol required tissues, which have been examined, have no visible lesions

Histopathology - The following Tissues were Not Examined:

None

2759-001 - Individual Animal Data Record: Pathology, Day 8

Animal: 4006 Sex: Male

Dose: High Dose

Study Day (Week) of Death: 8 (2)

Gross Pathology Observations [Correlation]:

No observations found

Any remaining protocol required tissues, which have been examined, have no visible lesions

Gross Pathology - The following Tissues were Not Examined:

None

Histopathology Animal Details:

No animal details found

Histopathology Observations [Correlation]:

dorsal root ganglia, lumbar, left : infiltration, mononuclear cell, perivascular; focal, minimal

Any remaining protocol required tissues, which have been examined, have no visible lesions

Histopathology - The following Tissues were Not Examined:

None

2759-001 - Individual Animal Data Record: Pathology, Day 8

Animal: 4007 Sex: Male

Dose: High Dose

Study Day (Week) of Death: 8 (2)

Gross Pathology Observations [Correlation]:

No observations found

Any remaining protocol required tissues, which have been examined, have no visible lesions

Gross Pathology - The following Tissues were Not Examined:

None

Histopathology Animal Details:

No animal details found

Histopathology Observations [Correlation]:

liver : infiltration, mononuclear cell; multifocal, minimal

Any remaining protocol required tissues, which have been examined, have no visible lesions

Histopathology - The following Tissues were Not Examined:

None

2759-001 - Individual Animal Data Record: Pathology, Day 8

Animal: 4008 Sex: Male

Dose: High Dose

Study Day (Week) of Death: 8 (2)

Gross Pathology Observations [Correlation]:

No observations found

Any remaining protocol required tissues, which have been examined, have no visible lesions

Gross Pathology - The following Tissues were Not Examined:

None

Histopathology Animal Details:

No animal details found

Histopathology Observations [Correlation]:

No observations found

Any remaining protocol required tissues, which have been examined, have no visible lesions

Histopathology - The following Tissues were Not Examined:

None

2759-001 - Individual Animal Data Record: Pathology, Day 8

Animal: 4009 Sex: Male

Dose: High Dose

Study Day (Week) of Death: 8 (2)

Gross Pathology Observations [Correlation]:

No observations found

Any remaining protocol required tissues, which have been examined, have no visible lesions

Gross Pathology - The following Tissues were Not Examined:

None

Histopathology Animal Details:

No animal details found

Histopathology Observations [Correlation]:

heart : cardiomyopathy; focal, minimal
thymus : hemorrhage; multifocal, minimal

Any remaining protocol required tissues, which have been examined, have no visible lesions

Histopathology - The following Tissues were Not Examined:

None

2759-001 - Individual Animal Data Record: Pathology, Day 8

Animal: 4010 Sex: Male

Dose: High Dose

Study Day (Week) of Death: 8 (2)

Gross Pathology Observations [Correlation]:

No observations found

Any remaining protocol required tissues, which have been examined, have no visible lesions

Gross Pathology - The following Tissues were Not Examined:

None

Histopathology Animal Details:

No animal details found

Histopathology Observations [Correlation]:

dorsal root ganglia lumbar, right : infiltration, mononuclear cell, perivascular; focal, minimal
liver : infiltration, mononuclear cell; multifocal, minimal

Any remaining protocol required tissues, which have been examined, have no visible lesions

Histopathology - The following Tissues were Not Examined:

None

2759-001 - Individual Animal Data Record: Pathology, Day 8

Animal: 4506 Sex: Female

Dose: High Dose

Study Day (Week) of Death: 8 (2)

Gross Pathology Observations [Correlation]:

No observations found

Any remaining protocol required tissues, which have been examined, have no visible lesions

Gross Pathology - The following Tissues were Not Examined:

None

Histopathology Animal Details:

No animal details found

Histopathology Observations [Correlation]:

spleen : depletion, lymphoid, marginal zone; multifocal, minimal

Any remaining protocol required tissues, which have been examined, have no visible lesions

Histopathology - The following Tissues were Not Examined:

None

2759-001 - Individual Animal Data Record: Pathology, Day 8

Animal: 4507 Sex: Female

Dose: High Dose

Study Day (Week) of Death: 8 (2)

Gross Pathology Observations [Correlation]:

No observations found

Any remaining protocol required tissues, which have been examined, have no visible lesions

Gross Pathology - The following Tissues were Not Examined:

None

Histopathology Animal Details:

No animal details found

Histopathology Observations [Correlation]:

kidneys : infiltration, mononuclear cell; focal, minimal
lung : infiltration, mononuclear cell; multifocal, minimal
spleen : depletion, lymphoid, marginal zone; multifocal, minimal

Any remaining protocol required tissues, which have been examined, have no visible lesions

Histopathology - The following Tissues were Not Examined:

None

2759-001 - Individual Animal Data Record: Pathology, Day 8

Animal: 4508 Sex: Female

Dose: High Dose

Study Day (Week) of Death: 8 (2)

Gross Pathology Observations [Correlation]:

No observations found

Any remaining protocol required tissues, which have been examined, have no visible lesions

Gross Pathology - The following Tissues were Not Examined:

None

Histopathology Animal Details:

No animal details found

Histopathology Observations [Correlation]:

kidneys : mineralization; multifocal, minimal
spleen : depletion, lymphoid, marginal zone; multifocal, mild

Any remaining protocol required tissues, which have been examined, have no visible lesions

Histopathology - The following Tissues were Not Examined:

None

2759-001 - Individual Animal Data Record: Pathology, Day 8

Animal: 4509 Sex: Female

Dose: High Dose

Study Day (Week) of Death: 8 (2)

Gross Pathology Observations [Correlation]:

No observations found

Any remaining protocol required tissues, which have been examined, have no visible lesions

Gross Pathology - The following Tissues were Not Examined:

None

Histopathology Animal Details:

No animal details found

Histopathology Observations [Correlation]:

spleen : depletion, lymphoid, marginal zone; multifocal, minimal
dorsal root ganglia, lumbar, left : infiltration, mononuclear cell, perivascular; focal, minimal

Any remaining protocol required tissues, which have been examined, have no visible lesions

Histopathology - The following Tissues were Not Examined:

dorsal root ganglia, cervical, left - Not In Plane Of Section

2759-001 - Individual Animal Data Record: Pathology, Day 8

Animal: 4510 Sex: Female

Dose: High Dose

Study Day (Week) of Death: 8 (2)

Gross Pathology Observations [Correlation]:

No observations found

Any remaining protocol required tissues, which have been examined, have no visible lesions

Gross Pathology - The following Tissues were Not Examined:

None

Histopathology Animal Details:

No animal details found

Histopathology Observations [Correlation]:

spleen : depletion, lymphoid, marginal zone; multifocal, marked

Any remaining protocol required tissues, which have been examined, have no visible lesions

Histopathology - The following Tissues were Not Examined:

None

Appendix 6
Individual Animal Data Macroscopic and Microscopic Pathology Findings (Day 29)

2759-001 - Individual Animal Data Record: Pathology, Day 29

Animal: 1011 Sex: Male

Dose: Vehicle

Study Day (Week) of Death: 29 (5)

Gross Pathology Observations [Correlation]:

No observations found

Any remaining protocol required tissues, which have been examined, have no visible lesions

Gross Pathology - The following Tissues were Not Examined:

None

Histopathology Animal Details:

No animal details found

Histopathology Observations [Correlation]:

spleen : hyperplasia, lymphoid, follicular; multifocal, minimal : (comment) Presence of follicular organization and activity.

Any remaining protocol required tissues, which have been examined, have no visible lesions

Histopathology - The following Tissues were Not Examined:

None

2759-001 - Individual Animal Data Record: Pathology, Day 29

Animal: 1012 Sex: Male

Dose: Vehicle

Study Day (Week) of Death: 29 (5)

Gross Pathology Observations [Correlation]:

liver : focus/foci, red; median lobe : (comment) 0.5 cm in diameter. (TGL)

Any remaining protocol required tissues, which have been examined, have no visible lesions

Gross Pathology - The following Tissues were Not Examined:

None

Histopathology Animal Details:

No animal details found

Histopathology Observations [Correlation]:

liver : congestion; focal, minimal [liver : focus/foci, red; median lobe : (comment) 0.5 cm in diameter. (G)]
lung : hemorrhage; multifocal, mild
spleen : hyperplasia, lymphoid, follicular; multifocal, minimal

Any remaining protocol required tissues, which have been examined, have no visible lesions

Histopathology - The following Tissues were Not Examined:

None

2759-001 - Individual Animal Data Record: Pathology, Day 29

Animal: 1013 Sex: Male

Dose: Vehicle

Study Day (Week) of Death: 29 (5)

Gross Pathology Observations [Correlation]:

No observations found

Any remaining protocol required tissues, which have been examined, have no visible lesions

Gross Pathology - The following Tissues were Not Examined:

None

Histopathology Animal Details:

No animal details found

Histopathology Observations [Correlation]:

heart : cardiomyopathy; focal, minimal
liver : infiltration, mononuclear cell; multifocal, minimal
lung : infiltration, mononuclear cell; multifocal, minimal

Any remaining protocol required tissues, which have been examined, have no visible lesions

Histopathology - The following Tissues were Not Examined:

None

2759-001 - Individual Animal Data Record: Pathology, Day 29

Animal: 1014 Sex: Male

Dose: Vehicle

Study Day (Week) of Death: 29 (5)

Gross Pathology Observations [Correlation]:

No observations found

Any remaining protocol required tissues, which have been examined, have no visible lesions

Gross Pathology - The following Tissues were Not Examined:

None

Histopathology Animal Details:

No animal details found

Histopathology Observations [Correlation]:

No observations found

Any remaining protocol required tissues, which have been examined, have no visible lesions

Histopathology - The following Tissues were Not Examined:

None

2759-001 - Individual Animal Data Record: Pathology, Day 29

Animal: 1015 Sex: Male

Dose: Vehicle

Study Day (Week) of Death: 29 (5)

Gross Pathology Observations [Correlation]:

No observations found

Any remaining protocol required tissues, which have been examined, have no visible lesions

Gross Pathology - The following Tissues were Not Examined:

None

Histopathology Animal Details:

No animal details found

Histopathology Observations [Correlation]:

lymph node, mesenteric : erythrocytosis/erythrophagocytosis, sinus; multifocal, minimal
spleen : hyperplasia, lymphoid, follicular; multifocal, minimal

Any remaining protocol required tissues, which have been examined, have no visible lesions

Histopathology - The following Tissues were Not Examined:

None

2759-001 - Individual Animal Data Record: Pathology, Day 29

Animal: 1511 Sex: Female

Dose: Vehicle

Study Day (Week) of Death: 29 (5)

Gross Pathology Observations [Correlation]:

No observations found

Any remaining protocol required tissues, which have been examined, have no visible lesions

Gross Pathology - The following Tissues were Not Examined:

None

Histopathology Animal Details:

No animal details found

Histopathology Observations [Correlation]:

dorsal root ganglia lumbar, right : infiltration, mononuclear cell, perivascular; focal, minimal
liver : infiltration, mononuclear cell; multifocal, minimal
lung : infiltration, mononuclear cell; focal, minimal
spleen : hyperplasia, lymphoid, follicular; multifocal, minimal
thymus : hemorrhage; multifocal, minimal

Any remaining protocol required tissues, which have been examined, have no visible lesions

Histopathology - The following Tissues were Not Examined:

None

2759-001 - Individual Animal Data Record: Pathology, Day 29

Animal: 1512 Sex: Female

Dose: Vehicle

Study Day (Week) of Death: 29 (5)

Gross Pathology Observations [Correlation]:

No observations found

Any remaining protocol required tissues, which have been examined, have no visible lesions

Gross Pathology - The following Tissues were Not Examined:

None

Histopathology Animal Details:

No animal details found

Histopathology Observations [Correlation]:

liver : infiltration, mononuclear cell; focal, minimal
lung : infiltration, mononuclear cell; focal, minimal

Any remaining protocol required tissues, which have been examined, have no visible lesions

Histopathology - The following Tissues were Not Examined:

None

2759-001 - Individual Animal Data Record: Pathology, Day 29

Animal: 1513 Sex: Female

Dose: Vehicle

Study Day (Week) of Death: 29 (5)

Gross Pathology Observations [Correlation]:

No observations found

Any remaining protocol required tissues, which have been examined, have no visible lesions

Gross Pathology - The following Tissues were Not Examined:

None

Histopathology Animal Details:

No animal details found

Histopathology Observations [Correlation]:

No observations found

Any remaining protocol required tissues, which have been examined, have no visible lesions

Histopathology - The following Tissues were Not Examined:

None

2759-001 - Individual Animal Data Record: Pathology, Day 29

Animal: 1514 Sex: Female

Dose: Vehicle

Study Day (Week) of Death: 29 (5)

Gross Pathology Observations [Correlation]:

No observations found

Any remaining protocol required tissues, which have been examined, have no visible lesions

Gross Pathology - The following Tissues were Not Examined:

None

Histopathology Animal Details:

No animal details found

Histopathology Observations [Correlation]:

kidneys : cyst; unilateral, focal, minimal
liver : infiltration, mononuclear cell; focal, minimal
lung : infiltration, mononuclear cell; focal, minimal

Any remaining protocol required tissues, which have been examined, have no visible lesions

Histopathology - The following Tissues were Not Examined:

None

2759-001 - Individual Animal Data Record: Pathology, Day 29

Animal: 1515 Sex: Female

Dose: Vehicle

Study Day (Week) of Death: 29 (5)

Gross Pathology Observations [Correlation]:

No observations found

Any remaining protocol required tissues, which have been examined, have no visible lesions

Gross Pathology - The following Tissues were Not Examined:

None

Histopathology Animal Details:

No animal details found

Histopathology Observations [Correlation]:

liver : infiltration, mononuclear cell; multifocal, minimal

Any remaining protocol required tissues, which have been examined, have no visible lesions

Histopathology - The following Tissues were Not Examined:

None

2759-001 - Individual Animal Data Record: Pathology, Day 29

Animal: 2011 Sex: Male

Dose: Low Dose

Study Day (Week) of Death: 29 (5)

Gross Pathology Observations [Correlation]:

No observations found

Any remaining protocol required tissues, which have been examined, have no visible lesions

Gross Pathology - The following Tissues were Not Examined:

None

Histopathology Animal Details:

No animal details found

Histopathology Observations [Correlation]:

liver : infiltration, mononuclear cell; focal, minimal

Any remaining protocol required tissues, which have been examined, have no visible lesions

Histopathology - The following Tissues were Not Examined:

None

2759-001 - Individual Animal Data Record: Pathology, Day 29

Animal: 2012 Sex: Male

Dose: Low Dose

Study Day (Week) of Death: 29 (5)

Gross Pathology Observations [Correlation]:

No observations found

Any remaining protocol required tissues, which have been examined, have no visible lesions

Gross Pathology - The following Tissues were Not Examined:

None

Histopathology Animal Details:

No animal details found

Histopathology Observations [Correlation]:

dorsal root ganglia lumbar, right : infiltration, mononuclear cell, perivascular; focal, minimal
heart : cardiomyopathy; focal, minimal
liver : infiltration, mononuclear cell; focal, minimal
lung : infiltration, mononuclear cell; focal, minimal
spleen : hyperplasia, lymphoid, follicular; multifocal, minimal

Any remaining protocol required tissues, which have been examined, have no visible lesions

Histopathology - The following Tissues were Not Examined:

None

2759-001 - Individual Animal Data Record: Pathology, Day 29

Animal: 2013 Sex: Male

Dose: Low Dose

Study Day (Week) of Death: 29 (5)

Gross Pathology Observations [Correlation]:

No observations found

Any remaining protocol required tissues, which have been examined, have no visible lesions

Gross Pathology - The following Tissues were Not Examined:

None

Histopathology Animal Details:

No animal details found

Histopathology Observations [Correlation]:

dorsal root ganglia lumbar, right : infiltration, mononuclear cell, perivascular; focal, mild
heart : cardiomyopathy; focal, minimal
liver : infiltration, mononuclear cell; multifocal, minimal

Any remaining protocol required tissues, which have been examined, have no visible lesions

Histopathology - The following Tissues were Not Examined:

None

2759-001 - Individual Animal Data Record: Pathology, Day 29

Animal: 2014 Sex: Male

Dose: Low Dose

Study Day (Week) of Death: 29 (5)

Gross Pathology Observations [Correlation]:

No observations found

Any remaining protocol required tissues, which have been examined, have no visible lesions

Gross Pathology - The following Tissues were Not Examined:

None

Histopathology Animal Details:

No animal details found

Histopathology Observations [Correlation]:

liver : infiltration, mononuclear cell; focal, minimal
thymus : hemorrhage; multifocal, minimal
dorsal root ganglia, lumbar, left : infiltration, mononuclear cell, perivascular; focal, minimal

Any remaining protocol required tissues, which have been examined, have no visible lesions

Histopathology - The following Tissues were Not Examined:

dorsal root ganglia, thoracic, left - Not In Plane Of Section

2759-001 - Individual Animal Data Record: Pathology, Day 29

Animal: 2015 Sex: Male

Dose: Low Dose

Study Day (Week) of Death: 29 (5)

Gross Pathology Observations [Correlation]:

No observations found

Any remaining protocol required tissues, which have been examined, have no visible lesions

Gross Pathology - The following Tissues were Not Examined:

None

Histopathology Animal Details:

No animal details found

Histopathology Observations [Correlation]:

liver : infiltration, mononuclear cell; focal, minimal
lung : infiltration, mononuclear cell; focal, minimal

Any remaining protocol required tissues, which have been examined, have no visible lesions

Histopathology - The following Tissues were Not Examined:

None

2759-001 - Individual Animal Data Record: Pathology, Day 29

Animal: 2511 Sex: Female

Dose: Low Dose

Study Day (Week) of Death: 29 (5)

Gross Pathology Observations [Correlation]:

No observations found

Any remaining protocol required tissues, which have been examined, have no visible lesions

Gross Pathology - The following Tissues were Not Examined:

None

Histopathology Animal Details:

No animal details found

Histopathology Observations [Correlation]:

liver : infiltration, mononuclear cell; multifocal, minimal
spleen : hyperplasia, lymphoid, follicular; multifocal, minimal

Any remaining protocol required tissues, which have been examined, have no visible lesions

Histopathology - The following Tissues were Not Examined:

None

2759-001 - Individual Animal Data Record: Pathology, Day 29

Animal: 2512 Sex: Female

Dose: Low Dose

Study Day (Week) of Death: 29 (5)

Gross Pathology Observations [Correlation]:

No observations found

Any remaining protocol required tissues, which have been examined, have no visible lesions

Gross Pathology - The following Tissues were Not Examined:

None

Histopathology Animal Details:

No animal details found

Histopathology Observations [Correlation]:

dorsal root ganglia lumbar, right : infiltration, mononuclear cell, perivascular; focal, minimal

Any remaining protocol required tissues, which have been examined, have no visible lesions

Histopathology - The following Tissues were Not Examined:

None

2759-001 - Individual Animal Data Record: Pathology, Day 29

Animal: 2513 Sex: Female

Dose: Low Dose

Study Day (Week) of Death: 29 (5)

Gross Pathology Observations [Correlation]:

No observations found

Any remaining protocol required tissues, which have been examined, have no visible lesions

Gross Pathology - The following Tissues were Not Examined:

None

Histopathology Animal Details:

No animal details found

Histopathology Observations [Correlation]:

heart : cardiomyopathy; focal, minimal
kidneys : cast/casts; focal, minimal : (comment) Hyalin cast with hyalin material around it.
kidneys : infiltration, mononuclear cell; focal, minimal
spleen : hyperplasia, lymphoid, follicular; multifocal, minimal
dorsal root ganglia, lumbar, left : infiltration, mononuclear cell, perivascular; focal, minimal

Any remaining protocol required tissues, which have been examined, have no visible lesions

Histopathology - The following Tissues were Not Examined:

None

2759-001 - Individual Animal Data Record: Pathology, Day 29

Animal: 2514 Sex: Female

Dose: Low Dose

Study Day (Week) of Death: 29 (5)

Gross Pathology Observations [Correlation]:

No observations found

Any remaining protocol required tissues, which have been examined, have no visible lesions

Gross Pathology - The following Tissues were Not Examined:

None

Histopathology Animal Details:

No animal details found

Histopathology Observations [Correlation]:

No observations found

Any remaining protocol required tissues, which have been examined, have no visible lesions

Histopathology - The following Tissues were Not Examined:

None

2759-001 - Individual Animal Data Record: Pathology, Day 29

Animal: 2515 Sex: Female

Dose: Low Dose

Study Day (Week) of Death: 29 (5)

Gross Pathology Observations [Correlation]:

No observations found

Any remaining protocol required tissues, which have been examined, have no visible lesions

Gross Pathology - The following Tissues were Not Examined:

None

Histopathology Animal Details:

No animal details found

Histopathology Observations [Correlation]:

No observations found

Any remaining protocol required tissues, which have been examined, have no visible lesions

Histopathology - The following Tissues were Not Examined:

None

2759-001 - Individual Animal Data Record: Pathology, Day 29

Animal: 3011 Sex: Male

Dose: Mid Dose

Study Day (Week) of Death: 29 (5)

Gross Pathology Observations [Correlation]:

No observations found

Any remaining protocol required tissues, which have been examined, have no visible lesions

Gross Pathology - The following Tissues were Not Examined:

None

Histopathology Animal Details:

No animal details found

Histopathology Observations [Correlation]:

heart : cardiomyopathy; focal, minimal
liver : infiltration, mononuclear cell; focal, minimal

Any remaining protocol required tissues, which have been examined, have no visible lesions

Histopathology - The following Tissues were Not Examined:

None

2759-001 - Individual Animal Data Record: Pathology, Day 29

Animal: 3012 Sex: Male

Dose: Mid Dose

Study Day (Week) of Death: 29 (5)

Gross Pathology Observations [Correlation]:

No observations found

Any remaining protocol required tissues, which have been examined, have no visible lesions

Gross Pathology - The following Tissues were Not Examined:

None

Histopathology Animal Details:

No animal details found

Histopathology Observations [Correlation]:

heart : cardiomyopathy; focal, minimal
liver : infiltration, mononuclear cell; multifocal, minimal

Any remaining protocol required tissues, which have been examined, have no visible lesions

Histopathology - The following Tissues were Not Examined:

None

2759-001 - Individual Animal Data Record: Pathology, Day 29

Animal: 3013 Sex: Male

Dose: Mid Dose

Study Day (Week) of Death: 29 (5)

Gross Pathology Observations [Correlation]:

No observations found

Any remaining protocol required tissues, which have been examined, have no visible lesions

Gross Pathology - The following Tissues were Not Examined:

None

Histopathology Animal Details:

No animal details found

Histopathology Observations [Correlation]:

heart : cardiomyopathy; multifocal, minimal

Any remaining protocol required tissues, which have been examined, have no visible lesions

Histopathology - The following Tissues were Not Examined:

None

2759-001 - Individual Animal Data Record: Pathology, Day 29

Animal: 3014 Sex: Male

Dose: Mid Dose

Study Day (Week) of Death: 29 (5)

Gross Pathology Observations [Correlation]:

No observations found

Any remaining protocol required tissues, which have been examined, have no visible lesions

Gross Pathology - The following Tissues were Not Examined:

None

Histopathology Animal Details:

No animal details found

Histopathology Observations [Correlation]:

dorsal root ganglia lumbar, right : infiltration, mononuclear cell, perivascular; focal, minimal
kidneys : mineralization; multifocal, minimal
lung : infiltration, mononuclear cell; focal, minimal
skeletal muscle, gastrocnemius : degeneration; focal, minimal
spleen : hyperplasia, lymphoid, follicular; multifocal, minimal
dorsal root ganglia, lumbar, left : infiltration, mononuclear cell, perivascular; focal, minimal

Any remaining protocol required tissues, which have been examined, have no visible lesions

Histopathology - The following Tissues were Not Examined:

None

2759-001 - Individual Animal Data Record: Pathology, Day 29

Animal: 3015 Sex: Male

Dose: Mid Dose

Study Day (Week) of Death: 29 (5)

Gross Pathology Observations [Correlation]:

No observations found

Any remaining protocol required tissues, which have been examined, have no visible lesions

Gross Pathology - The following Tissues were Not Examined:

None

Histopathology Animal Details:

No animal details found

Histopathology Observations [Correlation]:

heart : cardiomyopathy; focal, minimal
lung : infiltration, mononuclear cell; focal, minimal
spleen : hyperplasia, lymphoid, follicular; multifocal, minimal

Any remaining protocol required tissues, which have been examined, have no visible lesions

Histopathology - The following Tissues were Not Examined:

None

2759-001 - Individual Animal Data Record: Pathology, Day 29

Animal: 3511 Sex: Female

Dose: Mid Dose

Study Day (Week) of Death: 29 (5)

Gross Pathology Observations [Correlation]:

No observations found

Any remaining protocol required tissues, which have been examined, have no visible lesions

Gross Pathology - The following Tissues were Not Examined:

None

Histopathology Animal Details:

No animal details found

Histopathology Observations [Correlation]:

liver : infiltration, mononuclear cell; multifocal, minimal

Any remaining protocol required tissues, which have been examined, have no visible lesions

Histopathology - The following Tissues were Not Examined:

None

2759-001 - Individual Animal Data Record: Pathology, Day 29

Animal: 3512 Sex: Female

Dose: Mid Dose

Study Day (Week) of Death: 29 (5)

Gross Pathology Observations [Correlation]:

No observations found

Any remaining protocol required tissues, which have been examined, have no visible lesions

Gross Pathology - The following Tissues were Not Examined:

None

Histopathology Animal Details:

No animal details found

Histopathology Observations [Correlation]:

spleen : hyperplasia, lymphoid, follicular; multifocal, minimal

Any remaining protocol required tissues, which have been examined, have no visible lesions

Histopathology - The following Tissues were Not Examined:

None

2759-001 - Individual Animal Data Record: Pathology, Day 29

Animal: 3513 Sex: Female

Dose: Mid Dose

Study Day (Week) of Death: 29 (5)

Gross Pathology Observations [Correlation]:

No observations found

Any remaining protocol required tissues, which have been examined, have no visible lesions

Gross Pathology - The following Tissues were Not Examined:

None

Histopathology Animal Details:

No animal details found

Histopathology Observations [Correlation]:

skeletal muscle, biceps femoris : degeneration; focal, minimal
spleen : hyperplasia, lymphoid, follicular; multifocal, minimal

Any remaining protocol required tissues, which have been examined, have no visible lesions

Histopathology - The following Tissues were Not Examined:

None

2759-001 - Individual Animal Data Record: Pathology, Day 29

Animal: 3514 Sex: Female

Dose: Mid Dose

Study Day (Week) of Death: 29 (5)

Gross Pathology Observations [Correlation]:

No observations found

Any remaining protocol required tissues, which have been examined, have no visible lesions

Gross Pathology - The following Tissues were Not Examined:

None

Histopathology Animal Details:

No animal details found

Histopathology Observations [Correlation]:

kidneys : mineralization; multifocal, minimal
dorsal root ganglia, lumbar, left : infiltration, mononuclear cell, perivascular; focal, minimal

Any remaining protocol required tissues, which have been examined, have no visible lesions

Histopathology - The following Tissues were Not Examined:

None

2759-001 - Individual Animal Data Record: Pathology, Day 29

Animal: 3515 Sex: Female

Dose: Mid Dose

Study Day (Week) of Death: 29 (5)

Gross Pathology Observations [Correlation]:

No observations found

Any remaining protocol required tissues, which have been examined, have no visible lesions

Gross Pathology - The following Tissues were Not Examined:

None

Histopathology Animal Details:

No animal details found

Histopathology Observations [Correlation]:

kidneys : mineralization; multifocal, minimal
liver : infiltration, mononuclear cell; focal, minimal
dorsal root ganglia, lumbar, left : infiltration, mononuclear cell, perivascular; focal, minimal

Any remaining protocol required tissues, which have been examined, have no visible lesions

Histopathology - The following Tissues were Not Examined:

None

2759-001 - Individual Animal Data Record: Pathology, Day 29

Animal: 4011 Sex: Male

Dose: High Dose

Study Day (Week) of Death: 29 (5)

Gross Pathology Observations [Correlation]:

No observations found

Any remaining protocol required tissues, which have been examined, have no visible lesions

Gross Pathology - The following Tissues were Not Examined:

None

Histopathology Animal Details:

No animal details found

Histopathology Observations [Correlation]:

dorsal root ganglia lumbar, right : infiltration, mononuclear cell, perivascular; focal, minimal
liver : infiltration, mononuclear cell; multifocal, minimal

Any remaining protocol required tissues, which have been examined, have no visible lesions

Histopathology - The following Tissues were Not Examined:

None

2759-001 - Individual Animal Data Record: Pathology, Day 29

Animal: 4012 Sex: Male

Dose: High Dose

Study Day (Week) of Death: 29 (5)

Gross Pathology Observations [Correlation]:

No observations found

Any remaining protocol required tissues, which have been examined, have no visible lesions

Gross Pathology - The following Tissues were Not Examined:

None

Histopathology Animal Details:

No animal details found

Histopathology Observations [Correlation]:

kidneys : infiltration, mononuclear cell; focal, minimal
thymus : hemorrhage; multifocal, minimal

Any remaining protocol required tissues, which have been examined, have no visible lesions

Histopathology - The following Tissues were Not Examined:

None

2759-001 - Individual Animal Data Record: Pathology, Day 29

Animal: 4013 Sex: Male

Dose: High Dose

Study Day (Week) of Death: 29 (5)

Gross Pathology Observations [Correlation]:

skin : abrasion/scab; left lateral head; right lateral head (TGL) [Scabbed area, Face (C)]

Any remaining protocol required tissues, which have been examined, have no visible lesions

Gross Pathology - The following Tissues were Not Examined:

None

Histopathology Animal Details:

No animal details found

Histopathology Observations [Correlation]:

liver : infiltration, mononuclear cell; multifocal, minimal
lymph node, mesenteric : infiltration, mast cell; multifocal, minimal
skin : hyperkeratosis; focal, minimal [skin : abrasion/scab; left lateral head; right lateral head (G)]
skin : inflammation; focal, minimal

Any remaining protocol required tissues, which have been examined, have no visible lesions

Histopathology - The following Tissues were Not Examined:

None

2759-001 - Individual Animal Data Record: Pathology, Day 29

Animal: 4014 Sex: Male

Dose: High Dose

Study Day (Week) of Death: 29 (5)

Gross Pathology Observations [Correlation]:

No observations found

Any remaining protocol required tissues, which have been examined, have no visible lesions

Gross Pathology - The following Tissues were Not Examined:

None

Histopathology Animal Details:

No animal details found

Histopathology Observations [Correlation]:

kidneys : mineralization; multifocal, minimal
liver : infiltration, mononuclear cell; multifocal, minimal
lung : infiltration, mononuclear cell; multifocal, minimal
spleen : hyperplasia, lymphoid, follicular; multifocal, minimal
thymus : hemorrhage; multifocal, minimal

Any remaining protocol required tissues, which have been examined, have no visible lesions

Histopathology - The following Tissues were Not Examined:

None

2759-001 - Individual Animal Data Record: Pathology, Day 29

Animal: 4015 Sex: Male

Dose: High Dose

Study Day (Week) of Death: 29 (5)

Gross Pathology Observations [Correlation]:

No observations found

Any remaining protocol required tissues, which have been examined, have no visible lesions

Gross Pathology - The following Tissues were Not Examined:

None

Histopathology Animal Details:

No animal details found

Histopathology Observations [Correlation]:

dorsal root ganglia lumbar, right : infiltration, mononuclear cell, perivascular; focal, minimal
liver : infiltration, mononuclear cell; multifocal, minimal
spleen : hyperplasia, lymphoid, follicular; multifocal, minimal
dorsal root ganglia, lumbar, left : infiltration, mononuclear cell, perivascular; focal, minimal

Any remaining protocol required tissues, which have been examined, have no visible lesions

Histopathology - The following Tissues were Not Examined:

None

2759-001 - Individual Animal Data Record: Pathology, Day 29

Animal: 4511 Sex: Female

Dose: High Dose

Study Day (Week) of Death: 29 (5)

Gross Pathology Observations [Correlation]:

No observations found

Any remaining protocol required tissues, which have been examined, have no visible lesions

Gross Pathology - The following Tissues were Not Examined:

None

Histopathology Animal Details:

No animal details found

Histopathology Observations [Correlation]:

heart : cardiomyopathy; focal, minimal
kidneys : mineralization; focal, minimal
spleen : hyperplasia, lymphoid, follicular; multifocal, minimal

Any remaining protocol required tissues, which have been examined, have no visible lesions

Histopathology - The following Tissues were Not Examined:

None

2759-001 - Individual Animal Data Record: Pathology, Day 29

Animal: 4512 Sex: Female

Dose: High Dose

Study Day (Week) of Death: 29 (5)

Gross Pathology Observations [Correlation]:

No observations found

Any remaining protocol required tissues, which have been examined, have no visible lesions

Gross Pathology - The following Tissues were Not Examined:

None

Histopathology Animal Details:

No animal details found

Histopathology Observations [Correlation]:

dorsal root ganglia lumbar, right : infiltration, mononuclear cell, perivascular; focal, minimal
heart : infiltration, mononuclear cell; focal, minimal : (comment) Observed in the pericardial fat.
kidneys : infiltration, mononuclear cell; multifocal, minimal
spleen : hyperplasia, lymphoid, follicular; multifocal, minimal

Any remaining protocol required tissues, which have been examined, have no visible lesions

Histopathology - The following Tissues were Not Examined:

None

2759-001 - Individual Animal Data Record: Pathology, Day 29

Animal: 4513 Sex: Female

Dose: High Dose

Study Day (Week) of Death: 29 (5)

Gross Pathology Observations [Correlation]:

No observations found

Any remaining protocol required tissues, which have been examined, have no visible lesions

Gross Pathology - The following Tissues were Not Examined:

None

Histopathology Animal Details:

No animal details found

Histopathology Observations [Correlation]:

kidneys : mineralization; multifocal, minimal

Any remaining protocol required tissues, which have been examined, have no visible lesions

Histopathology - The following Tissues were Not Examined:

None

2759-001 - Individual Animal Data Record: Pathology, Day 29

Animal: 4514 Sex: Female

Dose: High Dose

Study Day (Week) of Death: 29 (5)

Gross Pathology Observations [Correlation]:

No observations found

Any remaining protocol required tissues, which have been examined, have no visible lesions

Gross Pathology - The following Tissues were Not Examined:

None

Histopathology Animal Details:

No animal details found

Histopathology Observations [Correlation]:

liver : infiltration, mononuclear cell; multifocal, minimal
lung : hemorrhage; focal, minimal
spleen : hyperplasia, lymphoid, follicular; multifocal, minimal
dorsal root ganglia, lumbar, left : infiltration, mononuclear cell, perivascular; focal, minimal

Any remaining protocol required tissues, which have been examined, have no visible lesions

Histopathology - The following Tissues were Not Examined:

None

2759-001 - Individual Animal Data Record: Pathology, Day 29

Animal: 4515 Sex: Female

Dose: High Dose

Study Day (Week) of Death: 29 (5)

Gross Pathology Observations [Correlation]:

No observations found

Any remaining protocol required tissues, which have been examined, have no visible lesions

Gross Pathology - The following Tissues were Not Examined:

None

Histopathology Animal Details:

No animal details found

Histopathology Observations [Correlation]:

kidneys : mineralization; multifocal, minimal
liver : infiltration, mononuclear cell; multifocal, minimal
skeletal muscle, gastrocnemius : degeneration; focal, minimal
dorsal root ganglia, lumbar, left : infiltration, mononuclear cell, perivascular; focal, minimal

Any remaining protocol required tissues, which have been examined, have no visible lesions

Histopathology - The following Tissues were Not Examined:

None

Appendix 7
Individual Animal Data Macroscopic and Microscopic Pathology Findings (Day 91)

2759-001 - Individual Animal Data Record: Pathology, Day 91

Animal: 1001 Sex: Male

Dose: Vehicle

Study Day (Week) of Death: 91 (13)

Gross Pathology Observations [Correlation]:

No observations found

Any remaining protocol required tissues, which have been examined, have no visible lesions

Gross Pathology - The following Tissues were Not Examined:

None

Histopathology Animal Details:

No animal details found

Histopathology Observations [Correlation]:

No observations found

Any remaining protocol required tissues, which have been examined, have no visible lesions

Histopathology - The following Tissues were Not Examined:

None

2759-001 - Individual Animal Data Record: Pathology, Day 91

Animal: 1002 Sex: Male

Dose: Vehicle

Study Day (Week) of Death: 91 (13)

Gross Pathology Observations [Correlation]:

No observations found

Any remaining protocol required tissues, which have been examined, have no visible lesions

Gross Pathology - The following Tissues were Not Examined:

None

Histopathology Animal Details:

No animal details found

Histopathology Observations [Correlation]:

heart : cardiomyopathy; multifocal, minimal
liver : infiltration, mononuclear cell; multifocal, minimal

Any remaining protocol required tissues, which have been examined, have no visible lesions

Histopathology - The following Tissues were Not Examined:

None

2759-001 - Individual Animal Data Record: Pathology, Day 91

Animal: 1003 Sex: Male

Dose: Vehicle

Study Day (Week) of Death: 91 (13)

Gross Pathology Observations [Correlation]:

No observations found

Any remaining protocol required tissues, which have been examined, have no visible lesions

Gross Pathology - The following Tissues were Not Examined:

None

Histopathology Animal Details:

No animal details found

Histopathology Observations [Correlation]:

liver : infiltration, mononuclear cell; multifocal, minimal

Any remaining protocol required tissues, which have been examined, have no visible lesions

Histopathology - The following Tissues were Not Examined:

None

2759-001 - Individual Animal Data Record: Pathology, Day 91

Animal: 1004 Sex: Male

Dose: Vehicle

Study Day (Week) of Death: 91 (13)

Gross Pathology Observations [Correlation]:

No observations found

Any remaining protocol required tissues, which have been examined, have no visible lesions

Gross Pathology - The following Tissues were Not Examined:

None

Histopathology Animal Details:

No animal details found

Histopathology Observations [Correlation]:

heart : cardiomyopathy; focal, minimal
liver : infiltration, mononuclear cell; focal, minimal
lung : infiltration, mononuclear cell; focal, minimal

Any remaining protocol required tissues, which have been examined, have no visible lesions

Histopathology - The following Tissues were Not Examined:

None

2759-001 - Individual Animal Data Record: Pathology, Day 91

Animal: 1005 Sex: Male

Dose: Vehicle

Study Day (Week) of Death: 91 (13)

Gross Pathology Observations [Correlation]:

kidneys : irregular surface; bilateral, approximately 50% affected (TGL)

Any remaining protocol required tissues, which have been examined, have no visible lesions

Gross Pathology - The following Tissues were Not Examined:

None

Histopathology Animal Details:

No animal details found

Histopathology Observations [Correlation]:

kidneys : inflammation, subacute/chronic; unilateral, moderate : (comment) Inflammation composed mainly of mononuclear cells is present in the cortex and expands into the pelvic space. Presence of cell debris, fibrous connective tissues and tubular cast are observed. [kidneys : irregular surface; bilateral, approximately 50% affected (G)]
liver : infiltration, mononuclear cell; multifocal, minimal
lung : infiltration, mononuclear cell; focal, minimal
thymus : hemorrhage; multifocal, minimal

Any remaining protocol required tissues, which have been examined, have no visible lesions

Histopathology - The following Tissues were Not Examined:

None

2759-001 - Individual Animal Data Record: Pathology, Day 91

Animal: 1501 Sex: Female

Dose: Vehicle

Study Day (Week) of Death: 91 (13)

Gross Pathology Observations [Correlation]:

No observations found

Any remaining protocol required tissues, which have been examined, have no visible lesions

Gross Pathology - The following Tissues were Not Examined:

None

Histopathology Animal Details:

No animal details found

Histopathology Observations [Correlation]:

liver : infiltration, mononuclear cell; focal, minimal

Any remaining protocol required tissues, which have been examined, have no visible lesions

Histopathology - The following Tissues were Not Examined:

None

2759-001 - Individual Animal Data Record: Pathology, Day 91

Animal: 1502 Sex: Female

Dose: Vehicle

Study Day (Week) of Death: 91 (13)

Gross Pathology Observations [Correlation]:

No observations found

Any remaining protocol required tissues, which have been examined, have no visible lesions

Gross Pathology - The following Tissues were Not Examined:

None

Histopathology Animal Details:

No animal details found

Histopathology Observations [Correlation]:

liver : infiltration, mononuclear cell; focal, minimal

Any remaining protocol required tissues, which have been examined, have no visible lesions

Histopathology - The following Tissues were Not Examined:

None

2759-001 - Individual Animal Data Record: Pathology, Day 91

Animal: 1503 Sex: Female

Dose: Vehicle

Study Day (Week) of Death: 91 (13)

Gross Pathology Observations [Correlation]:

No observations found

Any remaining protocol required tissues, which have been examined, have no visible lesions

Gross Pathology - The following Tissues were Not Examined:

None

Histopathology Animal Details:

No animal details found

Histopathology Observations [Correlation]:

kidneys : mineralization; multifocal, minimal

Any remaining protocol required tissues, which have been examined, have no visible lesions

Histopathology - The following Tissues were Not Examined:

None

2759-001 - Individual Animal Data Record: Pathology, Day 91

Animal: 1504 Sex: Female

Dose: Vehicle

Study Day (Week) of Death: 91 (13)

Gross Pathology Observations [Correlation]:

No observations found

Any remaining protocol required tissues, which have been examined, have no visible lesions

Gross Pathology - The following Tissues were Not Examined:

None

Histopathology Animal Details:

No animal details found

Histopathology Observations [Correlation]:

liver : infiltration, mononuclear cell; multifocal, minimal
lung : infiltration, mononuclear cell; focal, minimal

Any remaining protocol required tissues, which have been examined, have no visible lesions

Histopathology - The following Tissues were Not Examined:

None

2759-001 - Individual Animal Data Record: Pathology, Day 91

Animal: 1505 Sex: Female

Dose: Vehicle

Study Day (Week) of Death: 91 (13)

Gross Pathology Observations [Correlation]:

No observations found

Any remaining protocol required tissues, which have been examined, have no visible lesions

Gross Pathology - The following Tissues were Not Examined:

None

Histopathology Animal Details:

No animal details found

Histopathology Observations [Correlation]:

liver : infiltration, mononuclear cell; multifocal, minimal
dorsal root ganglia, lumbar, left : infiltration, mononuclear cell, perivascular; focal, minimal

Any remaining protocol required tissues, which have been examined, have no visible lesions

Histopathology - The following Tissues were Not Examined:

None

2759-001 - Individual Animal Data Record: Pathology, Day 91

Animal: 2001 Sex: Male

Dose: Low Dose

Study Day (Week) of Death: 91 (13)

Gross Pathology Observations [Correlation]:

No observations found

Any remaining protocol required tissues, which have been examined, have no visible lesions

Gross Pathology - The following Tissues were Not Examined:

None

Histopathology Animal Details:

No animal details found

Histopathology Observations [Correlation]:

heart : fibrosis; focal, minimal
liver : infiltration, mononuclear cell; multifocal, minimal
thymus : hemorrhage; focal, minimal

Any remaining protocol required tissues, which have been examined, have no visible lesions

Histopathology - The following Tissues were Not Examined:

None

2759-001 - Individual Animal Data Record: Pathology, Day 91

Animal: 2002 Sex: Male

Dose: Low Dose

Study Day (Week) of Death: 91 (13)

Gross Pathology Observations [Correlation]:

No observations found

Any remaining protocol required tissues, which have been examined, have no visible lesions

Gross Pathology - The following Tissues were Not Examined:

None

Histopathology Animal Details:

No animal details found

Histopathology Observations [Correlation]:

heart : cardiomyopathy; multifocal, minimal
liver : infiltration, mononuclear cell; multifocal, minimal
lung : infiltration, mononuclear cell; focal, minimal

Any remaining protocol required tissues, which have been examined, have no visible lesions

Histopathology - The following Tissues were Not Examined:

None

2759-001 - Individual Animal Data Record: Pathology, Day 91

Animal: 2003 Sex: Male

Dose: Low Dose

Study Day (Week) of Death: 91 (13)

Gross Pathology Observations [Correlation]:

No observations found

Any remaining protocol required tissues, which have been examined, have no visible lesions

Gross Pathology - The following Tissues were Not Examined:

None

Histopathology Animal Details:

No animal details found

Histopathology Observations [Correlation]:

liver : infiltration, mononuclear cell; multifocal, minimal
spleen : hyperplasia, lymphoid, follicular; multifocal, minimal
thymus : hemorrhage; multifocal, minimal

Any remaining protocol required tissues, which have been examined, have no visible lesions

Histopathology - The following Tissues were Not Examined:

None

2759-001 - Individual Animal Data Record: Pathology, Day 91

Animal: 2004 Sex: Male

Dose: Low Dose

Study Day (Week) of Death: 91 (13)

Gross Pathology Observations [Correlation]:

No observations found

Any remaining protocol required tissues, which have been examined, have no visible lesions

Gross Pathology - The following Tissues were Not Examined:

None

Histopathology Animal Details:

No animal details found

Histopathology Observations [Correlation]:

heart : cardiomyopathy; focal, minimal
liver : infiltration, mononuclear cell; multifocal, minimal

Any remaining protocol required tissues, which have been examined, have no visible lesions

Histopathology - The following Tissues were Not Examined:

None

2759-001 - Individual Animal Data Record: Pathology, Day 91

Animal: 2005 Sex: Male

Dose: Low Dose

Study Day (Week) of Death: 91 (13)

Gross Pathology Observations [Correlation]:

No observations found

Any remaining protocol required tissues, which have been examined, have no visible lesions

Gross Pathology - The following Tissues were Not Examined:

None

Histopathology Animal Details:

No animal details found

Histopathology Observations [Correlation]:

heart : cardiomyopathy; multifocal, minimal
liver : infiltration, mononuclear cell; multifocal, minimal
lung : infiltration, mononuclear cell; multifocal, minimal
thymus : hemorrhage; multifocal, minimal
dorsal root ganglia, lumbar, left : infiltration, mononuclear cell, perivascular; focal, minimal

Any remaining protocol required tissues, which have been examined, have no visible lesions

Histopathology - The following Tissues were Not Examined:

None

2759-001 - Individual Animal Data Record: Pathology, Day 91

Animal: 2501 Sex: Female

Dose: Low Dose

Study Day (Week) of Death: 91 (13)

Gross Pathology Observations [Correlation]:

No observations found

Any remaining protocol required tissues, which have been examined, have no visible lesions

Gross Pathology - The following Tissues were Not Examined:

None

Histopathology Animal Details:

No animal details found

Histopathology Observations [Correlation]:

spleen : hyperplasia, lymphoid, follicular; multifocal, minimal

Any remaining protocol required tissues, which have been examined, have no visible lesions

Histopathology - The following Tissues were Not Examined:

None

2759-001 - Individual Animal Data Record: Pathology, Day 91

Animal: 2502 Sex: Female

Dose: Low Dose

Study Day (Week) of Death: 91 (13)

Gross Pathology Observations [Correlation]:

No observations found

Any remaining protocol required tissues, which have been examined, have no visible lesions

Gross Pathology - The following Tissues were Not Examined:

None

Histopathology Animal Details:

No animal details found

Histopathology Observations [Correlation]:

No observations found

Any remaining protocol required tissues, which have been examined, have no visible lesions

Histopathology - The following Tissues were Not Examined:

None

2759-001 - Individual Animal Data Record: Pathology, Day 91

Animal: 2503 Sex: Female

Dose: Low Dose

Study Day (Week) of Death: 91 (13)

Gross Pathology Observations [Correlation]:

No observations found

Any remaining protocol required tissues, which have been examined, have no visible lesions

Gross Pathology - The following Tissues were Not Examined:

None

Histopathology Animal Details:

No animal details found

Histopathology Observations [Correlation]:

thymus : hemorrhage; multifocal, minimal

Any remaining protocol required tissues, which have been examined, have no visible lesions

Histopathology - The following Tissues were Not Examined:

None

2759-001 - Individual Animal Data Record: Pathology, Day 91

Animal: 2504 Sex: Female

Dose: Low Dose

Study Day (Week) of Death: 91 (13)

Gross Pathology Observations [Correlation]:

No observations found

Any remaining protocol required tissues, which have been examined, have no visible lesions

Gross Pathology - The following Tissues were Not Examined:

None

Histopathology Animal Details:

No animal details found

Histopathology Observations [Correlation]:

liver : infiltration, mononuclear cell; focal, minimal

Any remaining protocol required tissues, which have been examined, have no visible lesions

Histopathology - The following Tissues were Not Examined:

None

2759-001 - Individual Animal Data Record: Pathology, Day 91

Animal: 2505 Sex: Female

Dose: Low Dose

Study Day (Week) of Death: 91 (13)

Gross Pathology Observations [Correlation]:

No observations found

Any remaining protocol required tissues, which have been examined, have no visible lesions

Gross Pathology - The following Tissues were Not Examined:

None

Histopathology Animal Details:

No animal details found

Histopathology Observations [Correlation]:

No observations found

Any remaining protocol required tissues, which have been examined, have no visible lesions

Histopathology - The following Tissues were Not Examined:

None

2759-001 - Individual Animal Data Record: Pathology, Day 91

Animal: 3001 Sex: Male

Dose: Mid Dose

Study Day (Week) of Death: 91 (13)

Gross Pathology Observations [Correlation]:

No observations found

Any remaining protocol required tissues, which have been examined, have no visible lesions

Gross Pathology - The following Tissues were Not Examined:

None

Histopathology Animal Details:

No animal details found

Histopathology Observations [Correlation]:

heart : cardiomyopathy; focal, minimal
liver : infiltration, mononuclear cell; multifocal, minimal

Any remaining protocol required tissues, which have been examined, have no visible lesions

Histopathology - The following Tissues were Not Examined:

None

2759-001 - Individual Animal Data Record: Pathology, Day 91

Animal: 3002 Sex: Male

Dose: Mid Dose

Study Day (Week) of Death: 91 (13)

Gross Pathology Observations [Correlation]:

No observations found

Any remaining protocol required tissues, which have been examined, have no visible lesions

Gross Pathology - The following Tissues were Not Examined:

None

Histopathology Animal Details:

No animal details found

Histopathology Observations [Correlation]:

heart : cardiomyopathy; focal, mild
liver : infiltration, mononuclear cell; multifocal, minimal
lung : infiltration, mononuclear cell; focal, minimal

Any remaining protocol required tissues, which have been examined, have no visible lesions

Histopathology - The following Tissues were Not Examined:

None

2759-001 - Individual Animal Data Record: Pathology, Day 91

Animal: 3003 Sex: Male

Dose: Mid Dose

Study Day (Week) of Death: 91 (13)

Gross Pathology Observations [Correlation]:

No observations found

Any remaining protocol required tissues, which have been examined, have no visible lesions

Gross Pathology - The following Tissues were Not Examined:

None

Histopathology Animal Details:

No animal details found

Histopathology Observations [Correlation]:

liver : infiltration, mononuclear cell; multifocal, minimal

Any remaining protocol required tissues, which have been examined, have no visible lesions

Histopathology - The following Tissues were Not Examined:

None

2759-001 - Individual Animal Data Record: Pathology, Day 91

Animal: 3004 Sex: Male

Dose: Mid Dose

Study Day (Week) of Death: 91 (13)

Gross Pathology Observations [Correlation]:

No observations found

Any remaining protocol required tissues, which have been examined, have no visible lesions

Gross Pathology - The following Tissues were Not Examined:

None

Histopathology Animal Details:

No animal details found

Histopathology Observations [Correlation]:

liver : infiltration, mononuclear cell; multifocal, minimal
lung : infiltration, mononuclear cell; focal, minimal
thymus : hemorrhage; multifocal, minimal

Any remaining protocol required tissues, which have been examined, have no visible lesions

Histopathology - The following Tissues were Not Examined:

None

2759-001 - Individual Animal Data Record: Pathology, Day 91

Animal: 3005 Sex: Male

Dose: Mid Dose

Study Day (Week) of Death: 91 (13)

Gross Pathology Observations [Correlation]:

No observations found

Any remaining protocol required tissues, which have been examined, have no visible lesions

Gross Pathology - The following Tissues were Not Examined:

None

Histopathology Animal Details:

No animal details found

Histopathology Observations [Correlation]:

liver : infiltration, mononuclear cell; multifocal, minimal

Any remaining protocol required tissues, which have been examined, have no visible lesions

Histopathology - The following Tissues were Not Examined:

None

2759-001 - Individual Animal Data Record: Pathology, Day 91

Animal: 3501 Sex: Female

Dose: Mid Dose

Study Day (Week) of Death: 91 (13)

Gross Pathology Observations [Correlation]:

No observations found

Any remaining protocol required tissues, which have been examined, have no visible lesions

Gross Pathology - The following Tissues were Not Examined:

None

Histopathology Animal Details:

No animal details found

Histopathology Observations [Correlation]:

kidneys : mineralization; multifocal, minimal
liver : infiltration, mononuclear cell; multifocal, minimal
spleen : hyperplasia, lymphoid, follicular; multifocal, minimal

Any remaining protocol required tissues, which have been examined, have no visible lesions

Histopathology - The following Tissues were Not Examined:

None

2759-001 - Individual Animal Data Record: Pathology, Day 91

Animal: 3502 Sex: Female

Dose: Mid Dose

Study Day (Week) of Death: 91 (13)

Gross Pathology Observations [Correlation]:

No observations found

Any remaining protocol required tissues, which have been examined, have no visible lesions

Gross Pathology - The following Tissues were Not Examined:

None

Histopathology Animal Details:

No animal details found

Histopathology Observations [Correlation]:

kidneys : mineralization; multifocal, minimal
thymus : hemorrhage; multifocal, minimal

Any remaining protocol required tissues, which have been examined, have no visible lesions

Histopathology - The following Tissues were Not Examined:

None

2759-001 - Individual Animal Data Record: Pathology, Day 91

Animal: 3503 Sex: Female

Dose: Mid Dose

Study Day (Week) of Death: 91 (13)

Gross Pathology Observations [Correlation]:

No observations found

Any remaining protocol required tissues, which have been examined, have no visible lesions

Gross Pathology - The following Tissues were Not Examined:

None

Histopathology Animal Details:

No animal details found

Histopathology Observations [Correlation]:

dorsal root ganglia lumbar, right : infiltration, mononuclear cell, perivascular; focal, minimal
kidneys : infiltration, mononuclear cell; focal, minimal
dorsal root ganglia, lumbar, left : infiltration, mononuclear cell, perivascular; focal, minimal

Any remaining protocol required tissues, which have been examined, have no visible lesions

Histopathology - The following Tissues were Not Examined:

None

2759-001 - Individual Animal Data Record: Pathology, Day 91

Animal: 3504 Sex: Female

Dose: Mid Dose

Study Day (Week) of Death: 91 (13)

Gross Pathology Observations [Correlation]:

No observations found

Any remaining protocol required tissues, which have been examined, have no visible lesions

Gross Pathology - The following Tissues were Not Examined:

None

Histopathology Animal Details:

No animal details found

Histopathology Observations [Correlation]:

dorsal root ganglia lumbar, right : infiltration, mononuclear cell, perivascular; focal, minimal
kidneys : mineralization; multifocal, minimal
liver : infiltration, mononuclear cell; focal, minimal
dorsal root ganglia, lumbar, left : infiltration, mononuclear cell, perivascular; multifocal, minimal

Any remaining protocol required tissues, which have been examined, have no visible lesions

Histopathology - The following Tissues were Not Examined:

None

2759-001 - Individual Animal Data Record: Pathology, Day 91

Animal: 3505 Sex: Female

Dose: Mid Dose

Study Day (Week) of Death: 91 (13)

Gross Pathology Observations [Correlation]:

No observations found

Any remaining protocol required tissues, which have been examined, have no visible lesions

Gross Pathology - The following Tissues were Not Examined:

None

Histopathology Animal Details:

No animal details found

Histopathology Observations [Correlation]:

dorsal root ganglia lumbar, right : infiltration, mononuclear cell, perivascular; focal, minimal

Any remaining protocol required tissues, which have been examined, have no visible lesions

Histopathology - The following Tissues were Not Examined:

None

2759-001 - Individual Animal Data Record: Pathology, Day 91

Animal: 4001 Sex: Male

Dose: High Dose

Study Day (Week) of Death: 91 (13)

Gross Pathology Observations [Correlation]:

No observations found

Any remaining protocol required tissues, which have been examined, have no visible lesions

Gross Pathology - The following Tissues were Not Examined:

None

Histopathology Animal Details:

No animal details found

Histopathology Observations [Correlation]:

liver : infiltration, mononuclear cell; multifocal, minimal

Any remaining protocol required tissues, which have been examined, have no visible lesions

Histopathology - The following Tissues were Not Examined:

None

2759-001 - Individual Animal Data Record: Pathology, Day 91

Animal: 4002 Sex: Male
Dose: High Dose
Study Day (Week) of Death: 91 (13)

Gross Pathology Observations [Correlation]:

No observations found

Any remaining protocol required tissues, which have been examined, have no visible lesions

Gross Pathology - The following Tissues were Not Examined:

None

Histopathology Animal Details:

No animal details found

Histopathology Observations [Correlation]:

heart : cardiomyopathy; focal, minimal
liver : infiltration, mononuclear cell; multifocal, minimal

Any remaining protocol required tissues, which have been examined, have no visible lesions

Histopathology - The following Tissues were Not Examined:

None

2759-001 - Individual Animal Data Record: Pathology, Day 91

Animal: 4003 Sex: Male

Dose: High Dose

Study Day (Week) of Death: 91 (13)

Gross Pathology Observations [Correlation]:

No observations found

Any remaining protocol required tissues, which have been examined, have no visible lesions

Gross Pathology - The following Tissues were Not Examined:

None

Histopathology Animal Details:

No animal details found

Histopathology Observations [Correlation]:

heart : cardiomyopathy; focal, minimal
liver : infiltration, mononuclear cell; focal, minimal
lung : infiltration, mononuclear cell; multifocal, minimal

Any remaining protocol required tissues, which have been examined, have no visible lesions

Histopathology - The following Tissues were Not Examined:

None

2759-001 - Individual Animal Data Record: Pathology, Day 91

Animal: 4004 Sex: Male

Dose: High Dose

Study Day (Week) of Death: 91 (13)

Gross Pathology Observations [Correlation]:

No observations found

Any remaining protocol required tissues, which have been examined, have no visible lesions

Gross Pathology - The following Tissues were Not Examined:

None

Histopathology Animal Details:

No animal details found

Histopathology Observations [Correlation]:

heart : cardiomyopathy; multifocal, minimal
liver : infiltration, mononuclear cell; multifocal, minimal

Any remaining protocol required tissues, which have been examined, have no visible lesions

Histopathology - The following Tissues were Not Examined:

None

2759-001 - Individual Animal Data Record: Pathology, Day 91

Animal: 4005 Sex: Male

Dose: High Dose

Study Day (Week) of Death: 91 (13)

Gross Pathology Observations [Correlation]:

No observations found

Any remaining protocol required tissues, which have been examined, have no visible lesions

Gross Pathology - The following Tissues were Not Examined:

None

Histopathology Animal Details:

No animal details found

Histopathology Observations [Correlation]:

skeletal muscle, gastrocnemius : degeneration; focal, minimal
spleen : hyperplasia, lymphoid, follicular; multifocal, minimal

Any remaining protocol required tissues, which have been examined, have no visible lesions

Histopathology - The following Tissues were Not Examined:

None

2759-001 - Individual Animal Data Record: Pathology, Day 91

Animal: 4501 Sex: Female

Dose: High Dose

Study Day (Week) of Death: 91 (13)

Gross Pathology Observations [Correlation]:

No observations found

Any remaining protocol required tissues, which have been examined, have no visible lesions

Gross Pathology - The following Tissues were Not Examined:

None

Histopathology Animal Details:

No animal details found

Histopathology Observations [Correlation]:

No observations found

Any remaining protocol required tissues, which have been examined, have no visible lesions

Histopathology - The following Tissues were Not Examined:

None

2759-001 - Individual Animal Data Record: Pathology, Day 91

Animal: 4502 Sex: Female

Dose: High Dose

Study Day (Week) of Death: 91 (13)

Gross Pathology Observations [Correlation]:

No observations found

Any remaining protocol required tissues, which have been examined, have no visible lesions

Gross Pathology - The following Tissues were Not Examined:

None

Histopathology Animal Details:

No animal details found

Histopathology Observations [Correlation]:

kidneys : mineralization; multifocal, minimal
liver : infiltration, mononuclear cell; multifocal, minimal
lung : macrophages, alveolar; minimal : (comment) Foamy.

Any remaining protocol required tissues, which have been examined, have no visible lesions

Histopathology - The following Tissues were Not Examined:

None

2759-001 - Individual Animal Data Record: Pathology, Day 91

Animal: 4503 Sex: Female

Dose: High Dose

Study Day (Week) of Death: 91 (13)

Gross Pathology Observations [Correlation]:

No observations found

Any remaining protocol required tissues, which have been examined, have no visible lesions

Gross Pathology - The following Tissues were Not Examined:

None

Histopathology Animal Details:

No animal details found

Histopathology Observations [Correlation]:

liver : infiltration, mononuclear cell; focal, minimal
lung : infiltration, mononuclear cell; focal, minimal

Any remaining protocol required tissues, which have been examined, have no visible lesions

Histopathology - The following Tissues were Not Examined:

None

2759-001 - Individual Animal Data Record: Pathology, Day 91

Animal: 4504 Sex: Female

Dose: High Dose

Study Day (Week) of Death: 91 (13)

Gross Pathology Observations [Correlation]:

No observations found

Any remaining protocol required tissues, which have been examined, have no visible lesions

Gross Pathology - The following Tissues were Not Examined:

None

Histopathology Animal Details:

No animal details found

Histopathology Observations [Correlation]:

liver : infiltration, mononuclear cell; focal, minimal
spleen : hyperplasia, lymphoid, follicular; multifocal, minimal

Any remaining protocol required tissues, which have been examined, have no visible lesions

Histopathology - The following Tissues were Not Examined:

None

2759-001 - Individual Animal Data Record: Pathology, Day 91

Animal: 4505 Sex: Female

Dose: High Dose

Study Day (Week) of Death: 91 (13)

Gross Pathology Observations [Correlation]:

No observations found

Any remaining protocol required tissues, which have been examined, have no visible lesions

Gross Pathology - The following Tissues were Not Examined:

None

Histopathology Animal Details:

No animal details found

Histopathology Observations [Correlation]:

liver : infiltration, mononuclear cell; multifocal, minimal
lung : infiltration, mononuclear cell; focal, minimal

Any remaining protocol required tissues, which have been examined, have no visible lesions

Histopathology - The following Tissues were Not Examined:

None

Appendix 11
Biodistribution Analysis Report

REPORT

Biodistribution Study of AAV9/CLN7 in Normal WT rats

Work conducted at University of Texas Southwestern Medical Center in the laboratory of Dr. Steven Gray
Report prepared by Xin Chen, Steven Gray, and Violeta Zaric on 02/07/2020

Personnel and Role

- Xin Chen: DNA preparation, data compilation, and report preparation.
- Steven Gray: Supervisor and report preparation.
- Thomas Dong: DNA preparation.
- Frances Shaffo: DNA preparation.
- Violeta Zaric: qPCR analysis of DNA samples and report preparation.

I certify that, to the best of my knowledge, the information in this report is correct and a true representation of the work carried out.

	2/7/2020
Xin Chen	Date
	2/12/2020
Steven Gray	Date
	2/10/2020
Frances Shaffo	Date
	02/10/2020
Thomas Dong	Date
	02/12/2020
Violeta Zaric	Date

Table of Contents

1. OBJECTIVE 4
2. ABBREVIATIONS 4
3. MATERIALS AND METHODS 4
4. RESULTS AND DISCUSSION 5
5. CONCLUSIONS 6
6. REFERENCES 7
7. APPENDICES 16

List of Tables and Figures

Table 1. Experimental Design 5
Table 2. Raw data vg/rat diploid genome 14

Figure 1. AAV9/CLN7 biodistribution in normal rats. 5

1. OBJECTIVE

The objective for this study was to characterize the biodistribution pattern of Self-complementary Adeno-Associated Virus serotype 9 (scAAV9)/Jet-hCLN7opt-SV40pA (AAV9/CLN7 in short), following a single lumbar intrathecal (IT) injection in wild-type (WT) CD [Crl:CD(SD)] rats.

2. ABBREVIATIONS

AAV	Adeno-Associated Virus
CLN7	Neuronal ceroid lipofuscinosis-7
DNA	Deoxyribonucleic acid
GAN	Giant axonal neuropathy
IT	Lumbar intrathecal
JeT	Synthetic JeT promoter
PBS	Phosphate-buffered saline
qPCR	Quantitative PCR
Sc	Self-complementary
spA	Synthetic polyadenylation signal
SV40pA	Simian virus 40 polyadenylation signal
vg	Vector genomes
WT	Wild type

3. MATERIALS AND METHODS

3.1 AAV/CLN7 Vector Production

AAV/CLN7 vector in Phosphate-buffered saline (PBS), 5% Sorbitol, pH 7.4 containing 0.001% F-68 was produced by the Vigene Biosciences, Inc., Rockville, Maryland. The final vector product was prepared at a titer of 1×10^{14} vector genomes (vg) /mL (COA in the appendices).

3.2 Animal Studies

Animal study was performed by Charles River Laboratories, Inc. Mattawan MI. Male and female CD rats were randomized into cohorts, with 5 males and 5 females per cohort, and dosed as shown in Table 1. At the initiation of dosing, the animals assigned to study were approximately 8 to 9 weeks old and weighed between 165 g and 328 g. AAV9/CLN7 vector was injected IT once on day 1 in each animal by a qualified laboratory technician, in a volume of 20 or 60 µL, and a final dose of 5×10^{11}, 2×10^{12}, or 6×10^{12} vg/rat. Rats were sacrificed on day 8, 29, or 91, and tissues were collected frozen and sent to Dr. Gray's laboratory on dry ice for DNA purification and qPCR evaluation.

3.3 Biodistribution Analysis

Total genomic DNA was purified from tissue samples collected at necropsy day 29, using a Qiagen Qiacube HT. qPCR was used to determine the quantity of the CLN7 transgene per diploid rat genome. Qualification of the qPCR assay, as well as a detailed report of the standard operating procedures and qPCR study findings, is attached in the references.

Table 1. Experimental Design

Group No.	Dose Level (vg/animal)	Dose Concentration (vg/µL)	Dose Volume (µL)	No. of Animals					
				Necropsy Day 8		Necropsy Day 29		Necropsy Day 91	
				Male	Female	Male	Female	Male	Female
1	0	0	60	5	5	5	5	5	5
2	5×10^{11}	2.5×10^{10}	20	5	5	5	5	5	5
3	2×10^{12}	1×10^{11}	20	5	5	5	5	5	5
4	6×10^{12}	1×10^{11}	60	5	5	5	5	5	5

4. RESULTS AND DISCUSSION

An IT study was conducted in WT rats with each animal receiving a single injection of AAV9/CLN7 vector at a dose of 5×10^{11}, 2×10^{12}, or 6×10^{12} vg. Genomic DNA was purified from the samples collected at necropsy day 29. CLN7 vector biodistribution was quantified by qPCR and provided in Figure 1. IT delivery of AAV9/CLN7 vector results in dose dependent increase of CLN7 vector DNA across the central nervous system (brain and spinal cord) and peripheral organs (heart, lung, liver, kidney, ovary, and testes). The CLN7 vector DNA is concentrated closest to the injection site in the spinal cord and detected at lower levels in multiple brain regions. In the peripheral organs, similar high amounts of CLN7 DNA persist in liver and heart and to the less extent in testes, ovary, lung, and kidney. The pattern of CLN7 biodistribution in this study is consistent with that expected from AAV9 and observed in a previous study from Dr. Gray's laboratory where a similar vector, scAAV9/JeT-hGANopt-spA, was injected IT to WT rats at a dose of 6.6×10^{11} vg per rat (Gray lab, unpublished findings). Collectively, IT delivery of AAV9/CLN7 results in broad CLN7 biodistribution across rat body.

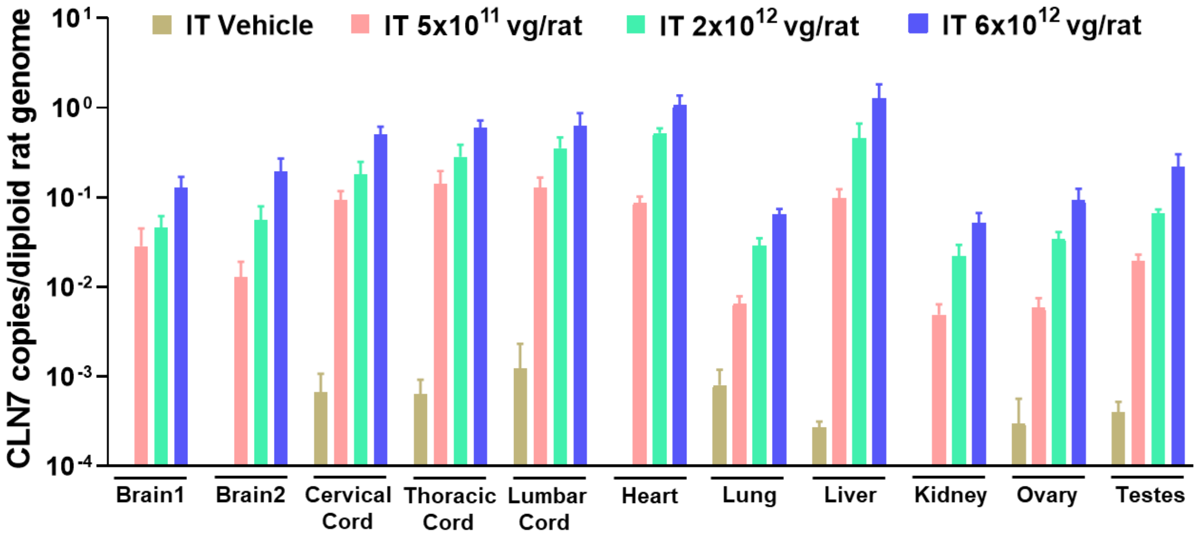

Figure 1. AAV9/CLN7 biodistribution in normal rats.

Normal rats received a single IT injection of scAAV9/JeT-hCLN7opt at a dose of 5×10^{11}, 2×10^{12}, or 6×10^{12} vg/rat. Genomic DNA was purified from the samples collected at necropsy day 29, and CLN7 vector biodistribution across the central nervous and peripheral organs was quantified by qPCR. Results were presented as Mean ± SEM.

5. CONCLUSIONS

The AAV9 vector genome DNA biodistribution pattern should be dependent on the capsid, regardless of the DNA cargo that it carries, which is further supported by the similar pattern of DNA biodistribution from this study using scAAV9/Jet-hCLN7opt-SV40pA as that expected for AAV9, and consistent with a previous similar study using scAAV9/JeT-hGANopt-spA from the Gray laboratory. The exceptions to this would be if an immune response led to viral clearance, or if there was toxicity against the expressed transgene that led to cell death and loss of viral genomes. In this study, the biodistribution pattern of AAV9/CLN7 at multiple doses (5×10^{11}, 2×10^{12}, or 6×10^{12} vg/rat) after IT injection in WT rats was determined. It was concluded that AAV9 delivered IT can achieve broad distribution across the nervous system and peripheral organs, although the level of gene transfer in the brain is sub-saturating with a minority of cells receiving the transgene. Thus, this biodistribution study is considered to portray the normal biodistribution pattern expected for an AAV9 vector in rats with vector biodistribution increasing linearly with dose. These results do not suggest any loss of vector due to cellular toxicity.

6. REFERENCES

6.1 Standard Operating Procedure

6.1.1 DNA Extraction of Total DNA from Animal Tissues using the Qiagen Qiacube HT.

Prepared by Xin Chen and Steven Gray, UTSW Medical Center
Version: 07-10-2017

Overview
Tissue samples are lysed, then automated extraction of total DNA is carried out using a Qiagen Qiacube HT. Special care is taken to avoid cross-contamination of samples. This protocol is based on the Qiagen's protocol to do the DNeasy Kit purification on the Qiagen Qiacube HT, and Qiagen's protocol is followed exactly unless otherwise specified. The DNA is eluted in Buffer EB (10 mM Tris, pH 8.5) rather than Buffer AE, to avoid potential inhibition of downstream PCR applications.

Protocol

1. The work area and all pipettes are wiped down with DNA-away solution. Filtered pipette tips are used in all manipulations. Clean gloves and appropriate PPE are worn during sample manipulations.
2. Cut up to 5mg tissue (up to 3mg spleen, about the size of the tissue from ear punched mice) pieces and place in a S-block on dry ice.
3. Make Proteinase K and buffer ATL mix in a ratio of 1:11
   a. One full S-block = 3 mL Proteinase K + 33 mL ATL buffer (Note: This amount is enough for running the plate once again if needed.)
4. To each tissue sample add:
   a. 400 µL buffer mix for Heart, Liver, Lung, Kidney Spleen, Gonads, Triceps, and Brain; or
   b. 300 µL buffer mix for sciatic, LC, CC, and DRG
5. Cover with TempPlateR EXT Sealing Foil.
6. Place S-block on a preheated Thermomixer at 56°C with shaking 800 rpm overnight.
7. Remove plate from Thermomixer and allow samples to cool to RT.
8. Check the stickiness of the samples especially for spleen, brain, heart, and triceps. Remove the clump if needed to prevent clogging the columns on Qiacube HT.
9. Turn on the instrument and the computer.
10. Launch the Qiacube HT Software.
11. On desktop, select the "**DNeasy…V5_dk_02202017.QSP**" run file from "**File**" or "**Recent**" tab.
12. Select the "**WIZARD Hat,**" select number of columns being run, enter lot numbers for Reagent and Plasticware kits (optional), and either click "**Next**" to go through each of the protocol steps or "**Jump to End**."
13. Load Adapters and Plasticware:
    a. Riser Block (Right/Elution side of sink)
    b. Channeling block holder and adapter (Left/Waste side of sink.)
    c. Carriage (must be pushed all the way to the LEFT over the LEFT side of the sink.)
    d. QIAamp 96 plate (taped off if applicable)
    e. Elution plate (Place on the instrument with the lid and leave on until prompted during pre-run checklist to remove. Plate must be pushed all the way to the LEFT to elute properly.)

   f. Tips (with lids on until prompted during pre-run checklist to remove.)
   g. To reset number of tips:
      i. Right click over a tip position.
      ii. Then select one of the options depending on what you are loading.
14. Load all buffers
   a. AW1
   b. 100% Ethanol
   c. AW2
   d. EB
   e. AL/EtOH
15. Load the S-Block with 220 µL of each sample onto deck position B1.
16. Press "**Play**."
17. At the end of the run when doing another run immediately following:
   a. Remove elution plate and cover with lid (after setting up for next run cap the columns.)
   b. Remove and discard the QIAamp 96 plate and any empty tip racks.
   c. Remove only the channeling block adapter (not the holder) from the waste sink and either swap for clean one or clean the one removed using DI water to rinse followed by 70% ethanol and allow to dry before next use.)
18. Clean the instrument at the end of the day use the "**Waste Pail.**" Running "**UV**" light after is optional.

Materials:

- QIAamp 96 DNA QCHT kit (cat no. 51331)
- QCHT plasticware (cat no. 950067)
- DNAaway solution (Molecular BioProducts and Cat #7010)
- Buffer EB (Qiagen, cat #19086)
- TempPlateR EXT Sealing Foil (cat no. 2998-7100)
- S-block (Qiagen, cat # 19585)
- Buffer AL (cat no. 19075)
- 100% Ethanol

6.1.2 Quantification of hCLN7opt in rat gDNA samples.

Prepared by Violeta Zaric, Xin Chen, and Steven Gray, UTSW Medical Center
Version: **02-06-2020**

A. Overview

This protocol is designed to use quantitative PCR (qPCR) to determine the double-stranded copies of the hCLN7opt DNA present in a purified genomic DNA sample. This SOP has been optimized and validated for use with rat genomic DNA. The total amount of sample DNA (host genomes) is determined by SYBR green qPCR analysis with primers specific to Rattus norvegicus lamin B2, and the copies of hCLN7opt DNA within each sample is determined by SYBR green qPCR analysis with primers specific for hCLN7opt.

B. Quantification of hCLN7 DNA in gDNA sample

1. *Make plasmid DNA standards*

Dilute plasmid DNA (pSJG-JeT-hCLN7opt-SV40pA) from Maxi-prep to 1×10^8 double-stranded copies/ µL stock in 10 mM Tris-EDTA buffer pH 8 (Invitrogen Cat# 9858) in 1.5 mL siliconized tubes (Fisher Cat# 02681331). Make a first dilution 1:20 to 1×10^7 copies following by serial 1:10 dilutions to 10 copies. Make 2 serial 1:2 dilutions from 10 copies to 5 copies, and 4 serial 1:2 dilutions from 100 copies to 6.25 copies as indicated. All dilutions are prepared with 10 mM Tris prepared from 1M Tris pH 8 (Invitrogen Cat# AM9855G) with UltraPure DNase/RNase-free distilled water (Invitrogen Cat# 10977-015). Vortex and spin briefly in every step.

1×10^7 copies/reaction — 10 µL of 10^8 copies/µL stock + 190 µL of 10 mM Tris
10 µL ↓
1×10^6 copies/reaction — + 90 µL of 10 mM Tris
10 µL ↓
1×10^5 copies/reaction — + 90 µL of 10 mM Tris
10 µL ↓
1×10^4 copies/reaction — + 90 µL of 10 mM Tris
10 µL ↓
1×10^3 copies/reaction — + 90 µL of 10 mM Tris
10 µL ↓
1×10^2 copies/reaction — + 90 µL of 10 mM Tris
10 µL ↓
10 copies/reaction — + 90 µL of 10 mM Tris
50 uL ↓
5 copies/reaction — + 50 µL of 10 mM Tris
50 µL ↓
2.5 copies/reaction — + 50 µL of 10 mM Tris

50 copies/reaction — 50 µL of 100 copies + 50 µL of 10 mM Tris
50 µL ↓
25 copies/reaction — + 50 µL of 10 mM Tris
50 µL ↓
12.5 copies/reaction — + 50 µL of 10 mM Tris
50 µL ↓
6.25 copies/reaction — + 50 µL of 10 mM Tris

2. *Prepare the qPCR SYBR master reactions*
   1) Set up gDNA samples as follows.

2× SYBR master mix	10 µL (Roche Cat# 04887352001)
Forward Primer (20 µM)	0.5 µL (ttcctcggcattctgaacat, IDT, Inc)
Reverse Primer (20 µM)	0.5 µL (gtcgatgcttcctcgaagtt, IDT, Inc)

   a. Pipet 11 µL of master mix into each well intended for gDNA samples.
   b. Add 9 µL of sample gDNA to the well.

   2) Set up standard plasmid DNA as follows.

2× SYBR mater mix	10 µL (Roche Cat# 04887352001)
Forward Primer (20 µM)	0.5 µL (ttcctcggcattctgaacat, Sigma)
Reverse Primer (20 µM)	0.5 µL (gtcgatgcttcctcgaagtt, Sigma)
H_2O	7 µL (Teknova Cat# W3440)

   a. Pipet 18 µL of master mix into each well intended for standard curve samples.
   b. Add 2 µL of plasmid DNA standard to the appropriate well.
   c. Add H_2O as no template control.
   d. Seal the plate with the seal for qPCR usage. (Roche Cat# 04-729-692-001)
   e. Spin down the plate @ 1800 rpm for 10 sec.
   f. Cycle in the Roche LightCycler480.

C. Quantification of Rattus norvegicus lamin B2

1. *Make genomic DNA standards*
   1) Measure the rat liver or rat kidney gDNA concentration using the LVis plate with the CLARIOstar plate reader (BMG LABTECH). Adjust the concentration to 60 ng/µL as the highest dilution stock.
   2) Make 6 serial 1:4 dilutions with 10 mM Tris from 60 ng/µL to 0.059 ng/µL.

2. *Preparation of gDNA samples for qPCR run*
   1) Dilute gDNA 1:10 with 10 mM Tris in a 96-well plate.
   2) Mix samples by pipetting up and down, then spin at 1800 rpm for 10 sec.

3. *Prepare the qPCR SYBR master reactions*
   1) Set up standard gDNA and gDNA samples as follows.

2× SYBR mater mix	5 µL (Roche Cat# 04887352001)
Forward Primer (20 µM)	0.25 µL (cctctcgggtacacagttcc, Sigma)
Reverse Primer (20 µM)	0.25 µL (gggcagcaagtctacaaagc, Sigma)
H_2O	2.5 µL (PCR water Teknova Cat# W3440)

   a. Pipet 8 µL of master mix into each well intended for gDNA samples.
   b. Add 2 µL of gDNA standard or prediluted gDNA samples
   c. Add H_2O as no template control.
   d. Seal the plate with the seal for qPCR usage. (Roche Cat#04-729-692-001)
   e. Spin down the plate @ 1800 rpm for 10 sec.
   f. Cycle in the Roche LightCycler480.

D. Running Cycles

hCLN7opt

	**Target (^{0}C)**	**Time (hh:mm:ss)**	**Cycle**	**Acquisition Mode**	**Ramp Rate**	**Acquisitions**	**Detect Mode**
Denature	95	0:10:00	1	none	4.8		SyBr green I/HRM Dye
Amplification	95	0:00:15	55	none	4.8		
	60	0:00:10		none	2.5		
	72	0:00:10		single	4.8		
Melt	95	0:00:05	1	none	4.8		
	65	0:01:00		none	2.5		
	95			continuous	0.11	5/^{0}C	
Cool	40	0:00:10	1	none	2.5		

Rattus norvegicus lamin B2

	**Target (^{0}C)**	**Time (hh:mm:ss)**	**Cycle**	**Acquisition Mode**	**Ramp Rate**	**Acquisitions**	**Detect Mode**
Denature	95	0:10:00	1	none	4.8		SyBr green I/HRM Dye
Amplification	95	0:00:10	45	none	4.8		
	60	0:00:10		none	2.5		
	72	0:00:10		single	4.8		
Melt	95	0:00:05	1	none	4.8		
	65	0:01:00		none	2.5		
	95			continuous	0.11	5/^{0}C	
Cool	40	0:00:10	1	none	2.5		

E. Analysis

Use Ab Quant/ 2nd derivative max in LightCycler 480 v 1.5 software to calculate the number of genomes per sample relative to the plasmid DNA standard curve or the amount of host DNA in ng relative to the rat gDNA standard. Use Tm calling as a quality control to check whether the specific product was amplified.

Calculation of number of copies of viral genome/µL

$$= \frac{\textit{Copy of virus genome as double strand DNA relative to the copy number of plasmid DNA standard}}{2}$$

- *Calculation of* ***genome copies*** *and amount (µg) of host DNA relative to the ng amount of host DNA standard*

This calculation assumes that the average weight of a bp of a double strand DNA is 620 g/mol, thus the molecular weight of DNA of 3 billion bps in a haploid cell is 1.85×10^{12} g/mol. The quantity of DNA in each cell contains (1.85×10^{12} g/mol)×(1 mole/6.022×10^{23} molecules)×2=6 pg of diploid DNA, so 1 pg of DNA contains 0.167 double strand copies=0.334 single-stranded copies of DNA, and 1 ng=334 single-stranded copies.

Number of rat lamin b2 genome copies = $\frac{ng*334\ (single\ stranded\ copies=genome)}{dilution\ \ factor*2}$

Conversion from ng/µL to µg/µL amount of host DNA= ng/µL of host DNA ×1000

- *Calculation of number of copies of Viral genomes normalized to number of copies of genome host*

$$=\frac{Copies\ of\ viral\ genome/\mu L}{Copies\ of\ genome\ DNA\ in\ host/\mu L}$$

- Calculation of number of copies of Viral genomes normalized to amount of host DNA host (µg)

$$=\frac{Copies\ of\ viral\ genome/\mu L}{amount\ of\ DNA\ in\ host\ (\mu g)/\mu L}$$

6.1.3 Validation studies

A. Validation Studies of JeT-hCLN7opt-SV40pA detection in no matrix.

Overview

Previous studies validated the use of CLN7opt 3F and 3R primers to detect the CLN7opt DNA with adequate sensitivity and reproducibility, in no matrix, a matrix of mouse genomic DNA, or a matrix of non-human primate genomic DNA. Lower limits of detection (LLOD) and quantification (LLOQ) were defined.

B. Validation Studies to Detect pSJG-JeT-hCLN7opt-SV40pA in a rat genomic DNA matrix.

Overview

The plasmid, pSJG-JeT-hCLN7opt-SV40pA, was run within a matrix of rat genomic DNA in 5 SYBR reactions using the CLN7opt primer set 3 to validate whether the PCR efficiency and lower detection threshold was maintained within the genomic DNA matrix.

Plasmid dilutions in gDNA matrix

The plasmid was diluted from 1×10^7 copies to 2.5 copies in all runs, and 2 replicates of serial dilution were used in all 42 independent runs. All of the dilutions were carried out in a matrix of 200 ng rat genomic DNA. 2nd derivative max and fit points algorithm were applied on the analysis. As with the plasmid DNA alone amplifications, Tm analysis was an accurate predictor of product purity visualized by gel electrophoresis. Among these 42 runs, the lower limit of detection (LLOD) was 10 copies of plasmid. 100% of replicates at or above 10 copies were successfully detected. Eight out of 10 replicates amplified 5 starting copies of template, 6 out of 10 replicates amplified 2 copies and 7 out of the 10 replicates amplified 1 copy. The efficiency was between 1.97 to 2 using the 2nd derivative max algorithm.

The variability of the LLOQ for detection of the CLN7opt plasmid sequence in the matrix of 200 ng rat genomic DNA was 28 to 76.7 copies detected with an input of 50 copies, 10.3 to 37.9 copies detected at 25 copies level, 8.57 to 27.7 copies at 10 copies, 1.93 to 18.7 at 5 copies, 2.34 to 14.1 copies at 2 copies and 0.00000000146 to 4.56 copies at 1 copy level in SYBR reaction with CLN7opt primer set 1.

Results

Our qPCR validation studies detected the plasmid pSJG-JeT-hCLN7opt-SV40pA at comparable efficiencies, LLOD, and LLOQ whether plasmid DNA alone was measured, or whether the plasmid DNA was detected in a matrix of rat genomic DNA. Overall, our LLOD was 100% sensitive to detect 10 copies of CLN7opt sequence in 200 ng of rat genomic DNA (50 copies/ug), *which conforms with FDA guidelines on conducting vector genome biodistribution studies*. This plasmid is our production plasmid to make the proposed clinical vector scAAV9/JeT-hCLN7opt. We conclude that these PCR conditions can be used to detect the CLN7opt viral genome in rat genomic DNA samples.

6.1.4 DETECTION OF CLN7opt GENOMIC DNA FROM CRL STUDY 2759-001

Methods and Scoring Criteria

DNA was purified from all tissue samples. When possible, 200 ng was used as template in the genomic DNA qPCR reactions but in some cases the DNA concentration was too low and the maximum volume of DNA (<200 ng) was used. The amount of genomic DNA used as template is noted in on the attached excel file. All reactions were run at least in duplicate.

The PCR products were scored as positive only if the Tm of the final PCR product was correct and if the crossing point (Cp) was <40 cycles. Any reaction that did not meet these criteria was scored as zero.

Results

Following the above quality control criteria, in the vehicle-injected gDNA rat samples we had a false positive rate of 0.7% (4/570 samples) to detect hCLN7opt. Some of the vehicle-injection cohorts only had an N=2, which did not negatively impact the study.

Conclusion

hCLN7opt was dose dependently distributed across the CNS and also to peripheral organs following intrathecal AAV9-mediated gene transfer in rats (see raw data vg/rat diploid genome in Figure 1 below).

Table 2. Raw data vg/rat diploid genome

Rat ID	Brain 1	Brain 2	CC	TC	LC	Heart	Lung	Liver	Kidney	Ovary	Testes
1011	0.0000	0.0000					0.0017	0.0002	0.0002		0.0008
1012			0.0006				0.0000	0.0003	0.0004		0.0005
1013				0.0007		0.0000	0.0000	0.0004	0.0000		0.0002
1014	0.0000				0.0045	0.0000	0.0000	0.0002	0.0000		0.0001
1015		0.0000				0.0000	0.0035	0.0001	0.0000		0.0004
1511			0.0000		0.0000	0.0000	0.0001	0.0003	0.0000	0.0011	
1512						0.0000	0.0002	0.0004	0.0000	0.0001	
1513					0.0002	0.0001			0.0000	0.0000	
1514		0.0000	0.0014	0.0011			0.0009		0.0001	0.0000	
1515				0.0001	0.0002		0.0008				
2011	0.0097	0.0217	0.1670	0.0653		0.1782	0.0038	0.1321	0.0050		0.0142
2012	0.1553		0.2366	0.2349	0.1746	0.0984	0.0070	0.0984	0.0007		0.0292
2013		0.0049	0.0587	0.0345	0.1110	0.0587	0.0059	0.0235	0.0060		0.0182
2014	0.0415	0.0525	0.0623	0.1649	0.3539	0.0879	0.0070	0.0410	0.0040		
2015	0.0126		0.1551	0.6029	0.1973	0.1511	0.0036	0.0510	0.0081		0.0166
2511	0.0027	0.0026	0.0224	0.0502	0.0338	0.0398	0.0170	0.0860	0.0017	0.0027	
2512	0.0038	0.0111	0.0265	0.0521	0.1695	0.0534	0.0031	0.0461	0.0017	0.0026	
2513	0.0029	0.0006	0.0132	0.0149	0.0217	0.0884	0.0031	0.1084	0.0149	0.0054	
2514	0.0058	0.0032	0.0676	0.1047	0.0129	0.0419	0.0100	0.0876	0.0021	0.0072	
2515	0.0224	0.0073	0.1273	0.0890	0.0918	0.0794	0.0048	0.3037		0.0114	
3011	0.0594	0.2385	0.3331		0.7081	0.5639	0.0150	0.1902	0.0454		0.0693
3012	0.0168	0.0138	0.3236	0.3436	0.4370	0.8010	0.0166	0.1458	0.0105		0.0857
3013	0.0767	0.0247	0.1180	0.3132	1.1398	0.3669	0.0162	0.0850	0.0069		0.0537
3014	0.0148	0.0154	0.0152	0.0075	0.0175		0.0356	2.0698			0.0555
3015	0.0283	0.0470	0.1128	0.2430	0.4509	0.2920	0.0299				
3511	0.0194	0.0070	0.0407	0.1538	0.0987	0.3661	0.0254	0.1514	0.0637	0.0270	

3512	0.0173	0.0271	0.0876	0.0577	0.1387	0.6401	0.0807	0.4947	0.0145	0.0480	
3513	0.0128	0.0191	0.0213	0.0406	0.0506	0.2889	0.0236	0.4459	0.0063	0.0142	
3514	0.0422	0.0339	0.0656	0.3508	0.0703	0.5589	0.0104	0.3413	0.0220	0.0480	
3515	0.1727	0.1326	0.6956	1.0400	0.4294	0.8233	0.0328	0.2141	0.0067	0.0355	
4011	0.3426	0.4842	0.5845	0.6250	0.8260		0.0300	0.4326	0.1175		0.2632
4012	0.0413	0.1270	0.1069	0.1337	0.1912	0.6093	0.0667	0.5074	0.0399		0.3393
4013		0.1016	0.0543	0.0654	0.0910	1.2233	0.0693				
4014		0.0949	0.7902	0.6956	0.4770	0.5964	0.0378				
4015	0.0961	0.6850	0.4848	0.6229	0.5169		0.0742				0.0581
4511	0.0322	0.0272	0.1217	0.1480	0.0829	2.7656	0.1125	1.0313	0.0173	0.0562	
4512		0.0708	0.4359	1.0783	0.6622	0.4014	0.0416	0.5291	0.0082	0.0480	
4513	0.0950	0.0632	1.2251	1.1929	2.3989	0.7297	0.0259	4.6061	0.0889	0.0371	
4514	0.1984	0.1026	0.4554	0.8305		1.1228	0.0631	0.4124	0.0341	0.2097	
4515	0.0912		0.7548	0.5722	0.4441		0.1184	1.3111	0.0579	0.1115	

7. APPENDICES

7.1 Test Article Certificate of Analysis

12111 Parklawn Drive
Rockville, MD 20852
(301)251-6638
www.vigenebio.com

Vigene Biosciences
Excellence in Gene Delivery

PRODUCT INFORMATION & CERTIFICATE OF ANALYSIS

PRODUCT INFORMATION

Tox Lot scAAV9-CLN7 virus production and purification

Date: September 8th 2018

SHELF LIFE: 2 years from date of receipt under proper storage conditions

SHIPMENT SPECIFICATION & HANDLING INSTRUCTION

Quantity	Description	Volume/titer
**50**	**AAV9/CLN7**	**200µl X 50<br>1.09+14GC /mL**

FORMULATION BUFFER

PBS, 5% Sorbitol, pH 7.4 containing 0.001%F-68

Test	Specification	Result	Unit
Viral genome titer (qPCR)	≥1E+14 GC/mL	1.09E+14 (±1.76E+13)	GC/mL
Residual Host Cell Protein	Report result	<LOD	ng/mL
Residual HCD	Report result	369±96	ng/mL
Residual Benzonase	Report result	<LOD (0.7)	ng/mL
Endotoxin	<10	2.7±0.8	ng/mL
pH	7.4±0.3	7.4	
Appearance	Clear	Clear	

Senior PD Director: MAN-SHIOW JIANG, PH.D.
